# Compliance to playpen usages to enhance parental supervision of under-five children in rural community of Bangladesh

**DOI:** 10.1371/journal.pone.0264902

**Published:** 2022-05-09

**Authors:** Md. Al-Amin Bhuiyan, Priyanka Agrawal, Olakunle Alonge, Zobaer Alam, Lamisa Ashraf, Shirin Wadhwaniya, Md. Abu Talab, Qingfeng Li, Abdulgafoor M. Bachani, Fazlur Rahman, Aminur Rahman

**Affiliations:** 1 Centre for Injury Prevention and Research, Bangladesh (CIPRB), Mohakhali, Dhaka, Bangladesh; 2 Department of International Health, Johns Hopkins Bloomberg School of Public Health, Baltimore, Maryland, United States of America; The University of Hong Kong, CHINA

## Abstract

**Introduction:**

In Bangladesh, injury is one of the leading causes of death and morbidity in children. All children under 5 years of age are at high risk for drowning though the risks are highest when children first learn to walk and crawl while they do not understand the danger of water. The Centre for Injury Prevention and Research, Bangladesh (CIPRB) in collaboration with Johns Hopkins International Injury Research Unit (*JH-IIRU*) has been implementing two drowning prevention interventions, providing playpens and community day care centres (anchal), or both in three rural sub-districts of Bangladesh under Saving of Lives from Drowning (SoLiD) project in Bangladesh. In CIPRB intervention areas, wooden playpens were distributed among the children nine months to three years at household (HH) level.

**Objective:**

The aim of this study was to explore and understand the acceptability and perceptions of parents towards playpen and its relevance for drowning and injury related mortality and morbidity prevention.

**Methods:**

Anchal mothers (‘anchal maa’ in Bangla) distributed 30,553 playpens and collected compliance information at the HH level using a structured questionnaire. 1600 trained anchal maas collected data via face to face interviews from May 2014 to November 2015. Playpen compliance visits were conducted periodically on the second and seventh days and every two months after delivering the playpen. Data were entered using standard data entry formats and analyzed using SPSS software version 23.

**Results:**

Parents reported that playpen is a safe place and protects children from drowning and other injuries. During compliance data collection, anchal maa founds that 71.8% of all children were using playpen and 93.7% (of 71.8%) children were playing inside the playpen while mothers were busy with their household chores like cooking, washing dishes and clothes, taking care of their poultry and domestic animals etc. 95.7% parents reported playpen is being used for keeping the child safe. On an average, the children were placed two to six times per day in a playpen. 99.1% of the children who reported using a playpen did not get any injuries (falls, cuts and bruises) while using the playpen. Satisfaction level with the playpen intervention among mothers was 90.5%. Some respondents suggested improving the playpen utilization by providing toys, adding wheels for ease of mobility, and increasing the height.

**Conclusion:**

The playpens were found to be well accepted and utilized for the children, especially when mothers were busy with their household chores.

## Background

In Bangladesh, an estimated 30,000 children under the age of 17 years die each year from injuries; 60% of these injuries are due to drowning [1]. Forty percent of all childhood mortality for children 1 to 4 years of age was due to drowning as per the 2011 Bangladesh Demographic and Health Survey [2]. A peak is seen among children one year of age when infants begin to explore their surroundings using their newly gained skills of mobility and tend to venture away from parental supervision [3].

Most deaths happen very close to home, with three quarters of all child drownings taking place in water less than 20 meters from the house. Almost all fatal drownings occur during the day, particularly between the hours of 9:00 am and 1:00 pm when parents and caregivers are busy with household chores or are working outside of the home [1]. Past research has also found that the rate of drowning and other injuries is much higher among children who live in rural areas than those residing in towns and cities where water hazards are sparse [4]. Additionally, inadequate supervision, young age, male gender, nearby water bodies, lack of physical barriers and fencing, and lack of swimming skills add to the risk of childhood drowning [5–8]. Absence of safety awareness, risky behavior around the water bodies, and underestimated perceived risk are also considered as important risk factors [9–11]. Two prevention tools, door barriers and playpens, were piloted in rural areas of Bangladesh as potential interventions to reduce drowning among children under 18 months of age [6, 7]. Both the interventions showed promising results in aiding supervision of the children; however, caregivers preferred playpens to door barriers [7]. Other studies also explored the option of community daycare centres (anchal) and found that children who were enrolled in the anchal program were 80% less likely to drown than those who were not enrolled [7, 12].

Playpens are being used as a child supervision aid for mothers for decades and are now a common household feature around the world [13]. Playpens (sometimes called play yards) offer a safe place where caregivers can keep their babies alone for a short period of time [14, 15]. Playpens are used to retain a young child fairly comfortably as they either asleep or stay awake, while minimizing potential hazards during these times of limited supervision, without impairing the parent or guardian’s access to the child should it be necessary [16]. A pilot study carried out in Matlab showed that caregivers at the time of an assessment appeared to choose to use the playpen and highlighted strong adoption as well as potential to enhance parental supervision practices [7].

The John Hopkins International Injury Research Unit (JH-IIRU), in collaboration with the Centre for Injury Prevention and Research, Bangladesh (CIPRB) and the International Centre for Diarrhoeal Disease Research, Bangladesh (icddr,b) implemented two drowning prevention interventions, the playpens and anchal services, or both, to families of children nine to 36 months of age to assess their large-scale effectiveness in reducing drowning and other injury deaths. The anchals are a secured room with doors and window, clean and carpeted floors, adequate light and ventilation that provide a safe space for 20 to 30 children per anchal to be supervised by and engaged in age-based singing, dancing and learning activities by an anchal mother and assistant, away from a water body. The playpens serve as barriers and assist parental supervision efforts, especially during critical times when fathers are away at work and mothers are busy with household chores. Given that the playpen acts as an adjunct to parental supervision, this study describes the compliance of users and caretakers in the usage of playpen.

## Methodology

The Saving of Lives from drowning project was implemented from 2013–2015 in seven sub-districts of rural Bangladesh. A baseline census was conducted in 2013 across the seven rural sub-districts to identify children eligible for enrollment in the anchal and playpen interventions. The baseline census also collected information on socio-demographic characteristics as well as birth and death history, and detailed injury profile if applicable. This paper is based on a sub-set of the population in the sub-districts Raiganj, Sherpur and Manohardi, that received playpens and were under the management of CIPRB. 19 unions were randomly chosen from three sub-districts and all the villages of those unions were covered in the study. CIPRB introduced the playpen intervention in the second year of the project to all pre-identified children between the ages of nine and 36 months. In addition, any new children migrating into the area and present during the playpen delivery were also included (Table 1).

**Table 1:** Target population for playpen intervention in the baseline assessment.

Sub-district	Number of villages	Number of unions	Total population	Total number of Households	Total number of children in 9–36 months of age
**Raiganj**	75	3	103,745	26,271	8,007
**Sherpur**	102	7	227,364	57,758	11,313
**Manohardi**	125	9	203,112	47,583	11,233
**Total**	**302**	**19**	**534,221**	**131,612**	**30,553**

In addition to baseline data collection, surveillance was also conducted to capture the injury profile of children enrolled in the study over a two-year time period. To assess the use of the playpen intervention in the home setting, compliance data collection using face-to-face interviews by trained anchal maas. Each interview took approximately 40–50 minutes. Prior to handing a parent the playpen, detailed instructions were provided on the correct set up and use of the playpen (Annex: 1). After determining identification and enrollment of participants into the study, an intervention worker introduced the playpen to the parent/caregiver and assisted them in the set up as well as provided instructions on usage. The parents/caregivers were visited by the intervention worker on days two and seven from the day the intervention was provided to support, educate and encourage utilization. Thereafter, anchal maas made home visits every two months to assess utilization of the playpen. All data were recorded on paper-based compliance forms.

Each household in the study area was given a unique household identification number and each household member was given a unique personal ID. The compliance data form (Annex: 5) collected basic demographics of the child, and direct observation of the child with respect to playpen use, current location, condition, and use of playpen, as well as past history of playpen usage. The child’s injury history with respect to playpen use as well as satisfaction of parent/caregiver in the use of playpen were also noted.

To maintain confidentiality of the participants, hard copies of data were kept securely locked in a cabinet in the office of the local co-investigators. Access to the information was limited only to study personnel involved in the actual follow-up and the workers were required to keep the data in their possession during transportation to and from field sites to offices. The paper-based data sheets were returned to the local principal investigators upon completion of the follow-up. The hard copies of the forms were transported in secured bags and were only accessed at the field office or at the central office.

To maintain the quality of data, 10% of compliance forms were re-observed by trained supervisors, another 10% of the collected data were re-checked, and 2% of the households were re-interviewed by the supervisors. All data were re-checked for inconsistencies. Also, coordinators were asked to re-check the data for inconsistencies. For inconsistent data, the concerned anchal maa was asked to revisit that household to collect the correct compliance information.

A data entry program using SQL Server 2008 was developed for data entry. Double entry was done for 5% of the compliance forms in each round of visit. Once data entry was completed, all personal identifiers were removed from the data sets and only the redacted versions of the data were made available for coding and analysis. The personal identifiers and their links (i.e., individual/household id) were kept secured in a password protected computer that only the PI or his designee had access to. All related information retrieved from the primary database was de-identified and transferred to SPSS version 23 for analysis. To analyze the compliance of playpen users, standard descriptive statistics were used to report socio-demographic characteristics and playpen use in counts and frequencies.

The data was retained according to the organization’s policy. Written informed consent was obtained from all children’s parents and/or caregivers. The households and study participants were informed that the data may be used for scientific research undertaken by the study team. The Institutional Review Board of the Johns Hopkins Bloomberg School of Public Health (JHSPH), and the Ethics Review Committees of the International Center for Diarrhoeal Disease Research, Bangladesh (icddr,b), and Center for Injury Prevention and Research, Bangladesh (CIPRB), approved this study (JHSPH IRB 00004746) under the study Saving of Lives from Drowning (SoLiD), Bangladesh.

## Results

A total of 30,553 playpens were distributed among children 9–36 months of age in the three sub-districts under the CIPRB management. Forty-six percent of all playpens were distributed in Sherpur Sadar of Sherpur district, followed by Manohardi (35.4%) of Narshingdi district and Raiganj (18.9%) of Sirajganj district (Table 2).

**Table 2:** Socio-demographic characteristics among the playpen recipients.

Characteristics	*Frequency*	Percentage%
	*N = 30*,*553*	
**CHILD**		
**Sex**		
Boy	15,302	50.1
Girl	15,251	49.9
**Age group (months)**		
9–18	8,846	29.0
19–24	4,917	16.1
25–36	16,790	55.0
**HEAD OF HOUSEHOLD**		
**Level of Education**		
No education	16,766	54.9
Primary	9,183	30.1
Secondary	3,330	10.9
A level and above	1,274	4.2
**Occupation**		
Agriculture	5,138	16.8
Business	1,256	4.1
Skilled labor (Professional)	2,104	6.9
Unskilled/domestic (Unskilled)	284	0.9
Rickshaw/bus (Transport worker)	406	1.3
Students	7,461	24.4
Retired/unemployed/ housewife	10,392	34.0
Not applicable AND/OR others	3,512	11.5
**Marital Status**		
Married	16,639	54.5
Never married	4,886	16.0
Widowed/Divorced/Separated/Others	9,028	29.5
**Wealth quantile**		
Lowest	6,541	21.4
Low	7,250	23.7
Middle	5,397	17.7
High	6,214	20.3
Highest	5,151	16.9
**Sub-district**		
Raiganj	5,787	18.9
Sherpur	13,947	45.6
Manohardi	10,819	35.4

Forty-five percent (n = 13,763) of the children who received a playpen were 9 to 24 months of age while the rest (n = 16,790, 55%) were between 25–36 months old at the time they received the playpen. Around 55% (54.9%, n = 16,766) of the household heads had no education and around 30.1% had received at least primary level education. More than half (n = 16,639, 54.5%) of the heads of households were married, and 34% were either retired, unemployed, or housewives (n = 10,392) (Table 2). Detailed socio-demographic characteristics of the study population have been described in previous work [17].

### Direct observations by anchal maas

**Fig 1:**
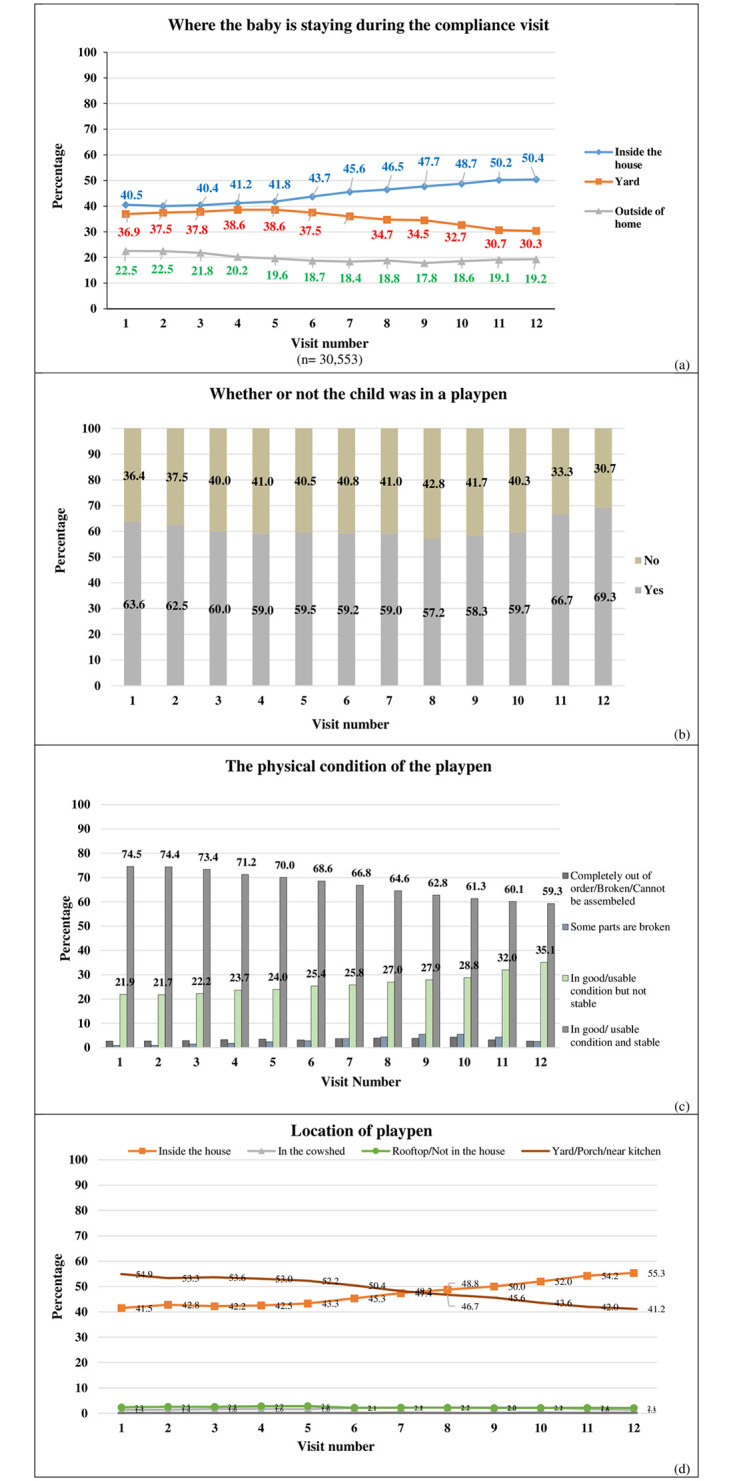


During the study period, every child who received a playpen had around 12 compliance visits. Looking at the aggregate data across the 12 compliance visits, 44.5% of the children were found either inside the house and around 35.6% children were in a yard near the house at the time of the visit. Of the children that were observed during a compliance visit, 61.1% children on average, were inside the playpen (inside the house or in a yard) during a compliance visit. Of those who were inside the playpen, 85.8% children on average were found playing while a small percent of the children was sleeping or crying (Table 3). Most playpens were placed either inside the house or in the yard/porch or near the kitchen by the child’s caregivers. As the study progressed, with each compliance visit, more playpens were observed inside the homes while a declining trend was seen in playpens lying in the yard (Fig 1). Most playpens were found to be in good condition, were usable and stable through the 12^th^ visit, however the stability declined slightly with usage. Around 95.1% caregivers/parents on average used the playpens for their child’s safety but some of them had repurposed the playpen for storing household goods or to put chicken/goat inside it (Fig 1).

**Table 3:** Child activities during playpen compliance visit.

Visit number	Sleeping		Playing		Crying		Others	
	n	%	n	%	n	%	N	%
1	1,075	7.1	13,136	87.3	795	5.3	47	0.3
2	944	7.1	11,529	87.0	745	5.6	32	0.2
3	908	7.4	10,563	86.5	707	5.8	39	0.3
4	892	7.7	9,964	86.1	689	6.0	32	0.3
5	862	8.2	9,039	85.5	628	5.9	46	0.4
6	800	8.9	7,697	85.2	490	5.4	49	0.5
7	808	10.8	6,198	83.0	422	5.7	36	0.5
8	567	10.9	4,372	83.8	258	4.9	23	0.4
9	372	10.8	2,896	84.0	170	4.9	8	0.2
10	213	11.3	1,577	83.7	89	4.7	5	0.3
11	104	10.2	868	85.0	45	4.4	4	0.4
12	55	8.8	554	88.6	16	2.6	-	-

### Self-reported utilization of playpens by the caregivers and/or users

**Fig 2:**
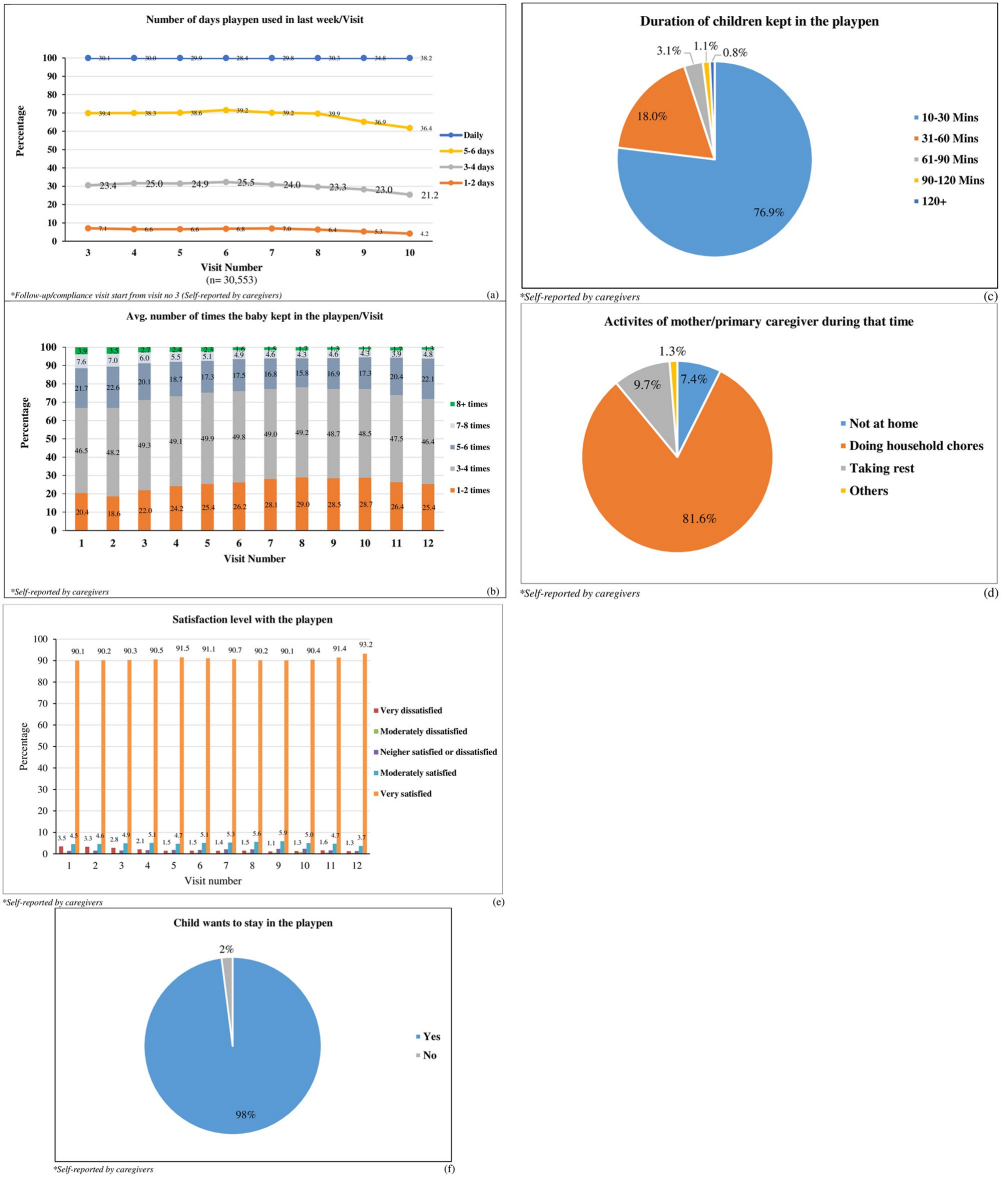


Around 30.7% of caregivers reported daily use of the playpen for their children and an upward trend was seen in its daily usage as the study progressed. Almost 38% of all caregivers used the playpen 5–6 times per week. On average, 48.5% caregivers used the playpens 3–4 times a day for varied durations (Fig 2). Caregivers reported that less than one percent of the children got playpen related injuries such as falls, cut, bruises or scratches. 98.8% caregivers reported no difficulties in using the playpens. As the playpens were made of wooden material and were heavy after set up, some parents had trouble moving them while some had issues with initial set up. Less than 1% of the parents thought it did not have enough space for their children. Almost all users (99%) reported that the playpens were convenient to use and 98% children want to stay in the playpen (Fig 2). Most of the caregivers reported in every follow-up visit that their child wanted to stay inside the playpen while they were busy with their regular/daily household activities. Most children who received a playpen were also enrolled at anchal centres; 4.2% (on average) children were not enrolled in the anchals due to their young age and remoteness to the center. Majority of the caregivers (90.8%) reported that they were very satisfied with the playpens and 4.9% were moderately satisfied with the playpen intervention (Fig 2).

Parents expressed that a child were more likely to stay in the playpen if (s)he had a toy to play with, a snack to eat or if they could easily see their mother from the playpen. A few caregivers additionally found that children were more likely to stay in the playpen if another child or a sibling accompanied them in the playpen. Many respondents addressed that playpen was better than other methods of supervision (such as bell in the waist, fasten with rope and door barrier) within the home settings that were available to them.

## Discussion

Throughout the study, children aged nine to 36 months of age received a total of 30,553 playpens in the three sub-districts of rural Bangladesh [17]. During the compliance visits, approximately 80% of all children who had a playpen were found either inside the house or in a nearby yard at the time of the visit. Two-third of the children on average were observed inside the playpen across the 12 rounds of compliance visits. Most of the caregivers/parents used the playpens for their child’s safety. This study found that almost half of all caregivers used their playpens 3 to 4 times a day for their kids. Most of the children who received a playpen were also enrolled at anchal centres. Satisfaction with the use of the playpen was very high among the caregivers. This compliance study revealed that when playpens were provided to households in rural Bangladesh, there was sustained usage of a playpen as a supervisory aid for young children. Parents of children who use playpens have been found to have more time to carry out other chores and income earning activities while their children are in the playpen [18].

There were some reports of minor injuries while using playpens. It’s interesting to note that past work has shown that playpens assist in reducing injury risks, if manufacturing guidelines are from relevant agencies, such as the U.S Consumer Health Security Commission for the US are followed [19]. There is evidence to show that playpen interventions and similar barrier strategies are an effective method to prevent drowning including other injuries, however these studies are limited to high income countries [14]. Similar drowning prevention strategies in LMICs like Bangladesh have only been implemented at a smaller scale and there is lack of evidence to suggest the robustness of the intervention due to lack of direct continuous observation of playpen usage and translation into injury prevention including drowning [20, 21]. The safety recommendation is that playpens are not often used when a child can easily climb out and/or when he or she reaches a height of 34 inches (86 centimeters) or weighs 30 pounds (14 kilograms) [22].

While use of playpens in drowning and injury prevention has been recommended by the WHO and other researchers, to our knowledge, this project was the largest-scale study to procure and distribute locally manufactured playpens and follow young children to generate population-level, reliable estimates of compliance of playpen use among caregivers [5, 23, 24]. Findings indicate that the playpen intervention was acceptable in use among the community members. However, playpens were not shown to be effective in reducing drowning and/or injury deaths among young children when compared to historical cohorts [25]. Even though the study showed that parents self-reported the use of playpen 3–4 times a day, it was difficult to capture the amount of time a child actually spent in the playpen. On direct observation, only 12.7% (n = 3,877) children were witnessed inside the playpens through all 10 follow-up visits [20, 21, 25]. The multiple rounds of data collection may have been subjected to reporting bias as respondents could have been accustomed to the questions.

As Bangladesh has a huge number of rivers, ponds and plenty of ditches, fencing of all water bodies to prevent child drowning in rural Bangladesh is probably not feasible and cost-effective. Health education programs, swimming and water safety lessons have shown an estimated prevention of almost 85% drowning deaths among the larger populations are not appropriate and effective strategies for under-5 children (21). Drowning along with other injuries are not generally associated with a complete lack of adult supervision, rather, with a momentary lapse in supervision (22). Considering all individual and environmental factors, adult supervision and playpens are reasonable substitutes for fencing in the Bangladesh context for preventing drowning among children along with other injuries.

A Baby WASH pilot study in rural Zambia, sub-Saharan Africa, engaged the community to design a community-built play yard and qualitatively explored its use and appeal. Reports from caregivers suggest that the community-built play-yard acts as a supervision tool for caregivers and protects IYC from ingesting soil and livestock feces. Barriers to intervention use included caregivers’ WASH beliefs and practices, community reactions, and play-yard maintenance [26]. This also highlights the collateral benefits of the use of a playpen–cleanliness, less risk of infection due to limited movement, and better hygiene.

## Conclusion

This is the first large-scale study on the feasibility and adaption of playpens in a low- and middle-income setting. The main contributions of playpen intervention were to analyze the acceptability and feasibility of playpen intervention in Bangladesh’s rural community. The study also assessed the level and trend of playpen intervention compliance and identified the factors influencing playpen usage.

In rural Bangladesh, playpens provide a convenient, realistic, and suitable alternative to constant adult supervision. The caregivers are reasonably compliant and accept the intervention. To reduce the number of playpen-related injuries, more attention should be paid to the design and construction of these devices. Healthcare experts, childcare providers, parents, and other child carers should all be aware of the product’s usage guidelines. Furthermore, efficacy studies are required to assess the influence of playpens on particular death rates for injured children under the age of five.

## Supporting information

S1 File (HTM)

S2 File (HTM)

S3 File (PDF)

S1 Data (RAR)
