# Supplementary material for: Compliance to playpen usages to enhance parental supervision of under-five children in rural community of Bangladesh
Source: PLoS One. 2022 May 9;17(5):e0264902. doi: 10.1371/journal.pone.0264902 (PMC9084520; doi:10.1371/journal.pone.0264902)
Supplement: S1 File — (HTM) [file pone.0264902.s001.htm]

DrPlayPenCompliance\_1st Visit\_10March2022.htm


GET  
  FILE='D:\CreCHeS\JHU\_SoLiD 2 Paper\Playpen\Manuscript\Journal\PlosOne\_Playpen\Review-7\DrPlayPenCompliance\_1st Visit\_10March2022.sav'.  
DATASET NAME DataSet1 WINDOW=FRONT.  
FREQUENCIES VARIABLES=Dist\_1 Upazila\_1 Unions\_1 Village\_1 Bari HHNo Intcode Result MSlNo CareSl  
    VisitNo VDate\_1 BDate ChAge StTime EnTime ChSex MoSl W05a W05b W06 W07 W08 W09 W10 W11 W12a W12b  
    W12c W12cAge W12d W12dAge W12e W12eAge W12f W12fAge W12g W12gAge W12h W12hAge W12i W12iAge W12j  
    W12jAge W12k W12kAge W12X W12x1 W12xAge W13 W14 W15 W16 W17a W17b W17c W17d W17e W17f W17g W17h  
    W17i W17x W17x1 W18 W19 W20 W21 W22a W22b W22c W22d W22e W22f W22x W22x1 W23 W24 W24a W25 W26 W27  
    A10 A11 A12P Org Unique MSlNo\_InjID InjSlNo InjMorb Dist Upazila Unions Village ResID HeadNo VDate  
    A03 Age\_group DOB Sex SESindex Age\_Cat  
  /ORDER=ANALYSIS.

  

# **Frequencies**

  

NotesNotes, table, 0 levels of column headers and 2 levels of row headers, table with 3 columns and 14 rows

|  |  |  |
| --- | --- | --- |
| Output Created | | 10-MAR-2022 12:05:49 |
| Comments | |  |
| Input | Data | D:\CreCHeS\JHU\_SoLiD 2 Paper\Playpen\Manuscript\Journal\PlosOne\_Playpen\Review-7\DrPlayPenCompliance\_1st Visit\_10March2022.sav |
|  | Active Dataset | DataSet1 |
|  | Filter | <none> |
|  | Weight | <none> |
|  | Split File | <none> |
|  | N of Rows in Working Data File | 30553 |
| Missing Value Handling | Definition of Missing | User-defined missing values are treated as missing. |
|  | Cases Used | Statistics are based on all cases with valid data. |
| Syntax | | FREQUENCIES VARIABLES=Dist\_1 Upazila\_1 Unions\_1 Village\_1 Bari HHNo Intcode Result MSlNo CareSl  VisitNo VDate\_1 BDate ChAge StTime EnTime ChSex MoSl W05a W05b W06 W07 W08 W09 W10 W11 W12a W12b  W12c W12cAge W12d W12dAge W12e W12eAge W12f W12fAge W12g W12gAge W12h W12hAge W12i W12iAge W12j  W12jAge W12k W12kAge W12X W12x1 W12xAge W13 W14 W15 W16 W17a W17b W17c W17d W17e W17f W17g W17h  W17i W17x W17x1 W18 W19 W20 W21 W22a W22b W22c W22d W22e W22f W22x W22x1 W23 W24 W24a W25 W26 W27  A10 A11 A12P Org Unique MSlNo\_InjID InjSlNo InjMorb Dist Upazila Unions Village ResID HeadNo VDate  A03 Age\_group DOB Sex SESindex Age\_Cat  /ORDER=ANALYSIS. |
| Resources | Processor Time | 00:00:00.56 |
|  | Elapsed Time | 00:00:00.58 |
|  |  |  |

[DataSet1] D:\CreCHeS\JHU\_SoLiD 2 Paper\Playpen\Manuscript\Journal\PlosOne\_Playpen\Review-7\DrPlayPenCompliance\_1st Visit\_10March2022.sav

  

StatisticsStatistics, table, 1 levels of column headers and 2 levels of row headers, table with 105 columns and 4 rows

|  |  |  |  |  |  |  |  |  |  |  |  |  |  |  |  |  |  |  |  |  |  |  |  |  |  |  |  |  |  |  |  |  |  |  |  |  |  |  |  |  |  |  |  |  |  |  |  |  |  |  |  |  |  |  |  |  |  |  |  |  |  |  |  |  |  |  |  |  |  |  |  |  |  |  |  |  |  |  |  |  |  |  |  |  |  |  |  |  |  |  |  |  |  |  |  |  |  |  |  |  |  |  |  |  |
| --- | --- | --- | --- | --- | --- | --- | --- | --- | --- | --- | --- | --- | --- | --- | --- | --- | --- | --- | --- | --- | --- | --- | --- | --- | --- | --- | --- | --- | --- | --- | --- | --- | --- | --- | --- | --- | --- | --- | --- | --- | --- | --- | --- | --- | --- | --- | --- | --- | --- | --- | --- | --- | --- | --- | --- | --- | --- | --- | --- | --- | --- | --- | --- | --- | --- | --- | --- | --- | --- | --- | --- | --- | --- | --- | --- | --- | --- | --- | --- | --- | --- | --- | --- | --- | --- | --- | --- | --- | --- | --- | --- | --- | --- | --- | --- | --- | --- | --- | --- | --- | --- | --- | --- | --- |
|  | | District code | Upazila code | Unions code | Village code | Bari number | Identification number | Interviewer code | Interview result code | Serial # of HH members | Caregiver's serial number | Visit number | Visit date | Date of birth of individual | Child age | Interview start time | Interview end time | Sex of the individual | mother's serial number | relationship of primary caregiver with child | Age of the primary caregiver | Where the baby stay at the time of visit | Whether the child was in the playpen | What the child doing inside the playpen | Location of playpen | The physical condition of the playpen | What is the playpen being used | No one | Mother/primary caregiver | Paternal grandmother | Age of grandmother | paternal grandfather | Age of paternal grandfather | Father | Age of father | Maternal grandfather | Age of Maternal grandfather | Maternal grandmother | Age of Maternal grandmother | Sibling | Age of sibling | Cousin | Age of Cousin | Aunt/uncle | Age of Aunt/uncle | Neighbour | Age of Neighbour | Others | Other specify | Age of other person | Activites of mother/primary caregiver during that time | Whether the mother used the plapen since the intervention worker's last visit | Number of days that the plapen used in last week | Average number of times the baby kept in the palpen | Wash dishes/cloth | Taking care of the poultry/domestic animals | Collect fuel/water/irrigation work | Child Care-Bathing,teaching,feeding,taking to school | Care of other HH members | Other HH work e.g.Cleaning the HH | Working in others house | Leisure activities-chatting,sleeping,taking rest etc. | Cooking | Others | Other(Specify) | How long the child kept in the playpen during last use of the playpen | Whether the child got any injuries(fell out.cut,bruise)while the playpen being u | Describe in detail how the child got injured | Any difficulties faces during using the playpen | Inconvenient | Child does not want to stay in the playpen | Child goes to creche | Have a caregiver to look after the child | Don't think it is necessary | Have other methods of supervision(specify) | Others | Other specify | Satisfaction level with the playpen intervention | Any suggestions to improve the playpen intervention | Code of suggestion | Currently participate in any income generating activities | Number of hours per day devote to income generating activities | Numder of hours per day devote to household chores | Marital status | Education of the individual | Primary occupation | Organization | Unique Person Identifier | Serial # of HH member | Injury serial number | Injury event | District code | Upazila code | Unions code | Village code | Respondent id number | Household headâ€™s serial number | Visit date | Mother's serial number of the individual | Age group | Date of birth | Sex of the individual | 5 quantiles of econ | Age category |
| N | Valid | 30553 | 30553 | 30553 | 30553 | 30553 | 30553 | 30553 | 30553 | 30553 | 30 | 30553 | 30553 | 30553 | 30553 | 30553 | 30553 | 30553 | 30553 | 1057 | 1057 | 30553 | 23670 | 15053 | 30553 | 30553 | 30553 | 30553 | 30553 | 30553 | 30553 | 30553 | 30553 | 30553 | 30553 | 30553 | 30553 | 30553 | 30553 | 30553 | 261 | 30553 | 22 | 78 | 78 | 309 | 309 | 951 | 0 | 951 | 9193 | 30553 | 29209 | 29209 | 30553 | 30553 | 30553 | 30553 | 30553 | 30553 | 30553 | 30553 | 30553 | 30553 | 30553 | 29209 | 29209 | 30553 | 30553 | 30553 | 30553 | 30553 | 30553 | 30553 | 30553 | 30553 | 30553 | 30553 | 30553 | 30553 | 30553 | 30553 | 30476 | 30553 | 30553 | 30553 | 1121 | 30553 | 30553 | 30553 | 1121 | 1121 | 1121 | 1121 | 30553 | 30553 | 30553 | 1121 | 1121 | 1121 | 1121 | 1121 | 1121 | 30553 |
|  | Missing | 0 | 0 | 0 | 0 | 0 | 0 | 0 | 0 | 0 | 30523 | 0 | 0 | 0 | 0 | 0 | 0 | 0 | 0 | 29496 | 29496 | 0 | 6883 | 15500 | 0 | 0 | 0 | 0 | 0 | 0 | 0 | 0 | 0 | 0 | 0 | 0 | 0 | 0 | 0 | 0 | 30292 | 0 | 30531 | 30475 | 30475 | 30244 | 30244 | 29602 | 30553 | 29602 | 21360 | 0 | 1344 | 1344 | 0 | 0 | 0 | 0 | 0 | 0 | 0 | 0 | 0 | 0 | 0 | 1344 | 1344 | 0 | 0 | 0 | 0 | 0 | 0 | 0 | 0 | 0 | 0 | 0 | 0 | 0 | 0 | 0 | 77 | 0 | 0 | 0 | 29432 | 0 | 0 | 0 | 29432 | 29432 | 29432 | 29432 | 0 | 0 | 0 | 29432 | 29432 | 29432 | 29432 | 29432 | 29432 | 0 |
|  |  |  |  |  |  |  |  |  |  |  |  |  |  |  |  |  |  |  |  |  |  |  |  |  |  |  |  |  |  |  |  |  |  |  |  |  |  |  |  |  |  |  |  |  |  |  |  |  |  |  |  |  |  |  |  |  |  |  |  |  |  |  |  |  |  |  |  |  |  |  |  |  |  |  |  |  |  |  |  |  |  |  |  |  |  |  |  |  |  |  |  |  |  |  |  |  |  |  |  |  |  |  |  |  |

# **Frequency Table**

  

District codeDistrict code, table, 1 levels of column headers and 2 levels of row headers, table with 6 columns and 6 rows

|  |  |  |  |  |  |
| --- | --- | --- | --- | --- | --- |
|  | | Frequency | Percent | Valid Percent | Cumulative Percent |
| Valid | Sirajganj | 5787 | 18.9 | 18.9 | 18.9 |
|  | Sherpur | 13947 | 45.6 | 45.6 | 64.6 |
|  | Narshindi | 10819 | 35.4 | 35.4 | 100.0 |
|  | Total | 30553 | 100.0 | 100.0 |  |
|  |  |  |  |  |  |

Upazila codeUpazila code, table, 1 levels of column headers and 2 levels of row headers, table with 6 columns and 6 rows

|  |  |  |  |  |  |
| --- | --- | --- | --- | --- | --- |
|  | | Frequency | Percent | Valid Percent | Cumulative Percent |
| Valid | Raiganj | 5787 | 18.9 | 18.9 | 18.9 |
|  | Sherpur Sadar | 13947 | 45.6 | 45.6 | 64.6 |
|  | Manohardi | 10819 | 35.4 | 35.4 | 100.0 |
|  | Total | 30553 | 100.0 | 100.0 |  |
|  |  |  |  |  |  |

Unions codeUnions code, table, 1 levels of column headers and 2 levels of row headers, table with 6 columns and 12 rows

|  |  |  |  |  |  |
| --- | --- | --- | --- | --- | --- |
|  | | Frequency | Percent | Valid Percent | Cumulative Percent |
| Valid | 1 | 2824 | 9.2 | 9.2 | 9.2 |
|  | 2 | 3095 | 10.1 | 10.1 | 19.4 |
|  | 3 | 3371 | 11.0 | 11.0 | 30.4 |
|  | 4 | 4852 | 15.9 | 15.9 | 46.3 |
|  | 5 | 5313 | 17.4 | 17.4 | 63.7 |
|  | 6 | 5169 | 16.9 | 16.9 | 80.6 |
|  | 7 | 3660 | 12.0 | 12.0 | 92.6 |
|  | 8 | 1297 | 4.2 | 4.2 | 96.8 |
|  | 9 | 972 | 3.2 | 3.2 | 100.0 |
|  | Total | 30553 | 100.0 | 100.0 |  |
|  |  |  |  |  |  |

Village codeVillage code, table, 1 levels of column headers and 2 levels of row headers, table with 6 columns and 35 rows

|  |  |  |  |  |  |
| --- | --- | --- | --- | --- | --- |
|  | | Frequency | Percent | Valid Percent | Cumulative Percent |
| Valid | 001 | 2936 | 9.6 | 9.6 | 9.6 |
|  | 002 | 2248 | 7.4 | 7.4 | 17.0 |
|  | 003 | 1664 | 5.4 | 5.4 | 22.4 |
|  | 004 | 1258 | 4.1 | 4.1 | 26.5 |
|  | 005 | 2301 | 7.5 | 7.5 | 34.1 |
|  | 006 | 1941 | 6.4 | 6.4 | 40.4 |
|  | 007 | 1930 | 6.3 | 6.3 | 46.7 |
|  | 008 | 2006 | 6.6 | 6.6 | 53.3 |
|  | 009 | 1755 | 5.7 | 5.7 | 59.0 |
|  | 010 | 2330 | 7.6 | 7.6 | 66.7 |
|  | 011 | 1652 | 5.4 | 5.4 | 72.1 |
|  | 012 | 1344 | 4.4 | 4.4 | 76.5 |
|  | 013 | 1754 | 5.7 | 5.7 | 82.2 |
|  | 014 | 1043 | 3.4 | 3.4 | 85.6 |
|  | 015 | 554 | 1.8 | 1.8 | 87.4 |
|  | 016 | 408 | 1.3 | 1.3 | 88.8 |
|  | 017 | 610 | 2.0 | 2.0 | 90.8 |
|  | 018 | 630 | 2.1 | 2.1 | 92.8 |
|  | 019 | 278 | .9 | .9 | 93.7 |
|  | 020 | 326 | 1.1 | 1.1 | 94.8 |
|  | 021 | 345 | 1.1 | 1.1 | 95.9 |
|  | 022 | 207 | .7 | .7 | 96.6 |
|  | 023 | 215 | .7 | .7 | 97.3 |
|  | 024 | 265 | .9 | .9 | 98.2 |
|  | 025 | 111 | .4 | .4 | 98.6 |
|  | 026 | 107 | .4 | .4 | 98.9 |
|  | 027 | 47 | .2 | .2 | 99.1 |
|  | 028 | 29 | .1 | .1 | 99.2 |
|  | 029 | 66 | .2 | .2 | 99.4 |
|  | 030 | 103 | .3 | .3 | 99.7 |
|  | 031 | 59 | .2 | .2 | 99.9 |
|  | 032 | 31 | .1 | .1 | 100.0 |
|  | Total | 30553 | 100.0 | 100.0 |  |
|  |  |  |  |  |  |

Bari numberBari number, table, 1 levels of column headers and 2 levels of row headers, table with 6 columns and 3 rows

|  |  |  |  |  |  |
| --- | --- | --- | --- | --- | --- |
|  | | Frequency | Percent | Valid Percent | Cumulative Percent |
| Valid |  | 30553 | 100.0 | 100.0 | 100.0 |
|  |  |  |  |  |  |

Identification numberIdentification number, table, 1 levels of column headers and 2 levels of row headers, table with 6 columns and 2478 rows

|  |  |  |  |  |  |
| --- | --- | --- | --- | --- | --- |
|  | | Frequency | Percent | Valid Percent | Cumulative Percent |
| Valid | 1 | 74 | .2 | .2 | .2 |
|  | 2 | 63 | .2 | .2 | .4 |
|  | 3 | 61 | .2 | .2 | .6 |
|  | 4 | 76 | .2 | .2 | .9 |
|  | 5 | 68 | .2 | .2 | 1.1 |
|  | 6 | 83 | .3 | .3 | 1.4 |
|  | 7 | 71 | .2 | .2 | 1.6 |
|  | 8 | 60 | .2 | .2 | 1.8 |
|  | 9 | 73 | .2 | .2 | 2.1 |
|  | 10 | 69 | .2 | .2 | 2.3 |
|  | 11 | 65 | .2 | .2 | 2.5 |
|  | 12 | 64 | .2 | .2 | 2.7 |
|  | 13 | 50 | .2 | .2 | 2.9 |
|  | 14 | 69 | .2 | .2 | 3.1 |
|  | 15 | 72 | .2 | .2 | 3.3 |
|  | 16 | 58 | .2 | .2 | 3.5 |
|  | 17 | 68 | .2 | .2 | 3.7 |
|  | 18 | 66 | .2 | .2 | 4.0 |
|  | 19 | 78 | .3 | .3 | 4.2 |
|  | 20 | 87 | .3 | .3 | 4.5 |
|  | 21 | 67 | .2 | .2 | 4.7 |
|  | 22 | 73 | .2 | .2 | 5.0 |
|  | 23 | 71 | .2 | .2 | 5.2 |
|  | 24 | 69 | .2 | .2 | 5.4 |
|  | 25 | 53 | .2 | .2 | 5.6 |
|  | 26 | 78 | .3 | .3 | 5.8 |
|  | 27 | 63 | .2 | .2 | 6.1 |
|  | 28 | 81 | .3 | .3 | 6.3 |
|  | 29 | 76 | .2 | .2 | 6.6 |
|  | 30 | 70 | .2 | .2 | 6.8 |
|  | 31 | 56 | .2 | .2 | 7.0 |
|  | 32 | 73 | .2 | .2 | 7.2 |
|  | 33 | 74 | .2 | .2 | 7.5 |
|  | 34 | 68 | .2 | .2 | 7.7 |
|  | 35 | 68 | .2 | .2 | 7.9 |
|  | 36 | 66 | .2 | .2 | 8.1 |
|  | 37 | 79 | .3 | .3 | 8.4 |
|  | 38 | 65 | .2 | .2 | 8.6 |
|  | 39 | 72 | .2 | .2 | 8.8 |
|  | 40 | 79 | .3 | .3 | 9.1 |
|  | 41 | 59 | .2 | .2 | 9.3 |
|  | 42 | 67 | .2 | .2 | 9.5 |
|  | 43 | 61 | .2 | .2 | 9.7 |
|  | 44 | 61 | .2 | .2 | 9.9 |
|  | 45 | 63 | .2 | .2 | 10.1 |
|  | 46 | 61 | .2 | .2 | 10.3 |
|  | 47 | 71 | .2 | .2 | 10.5 |
|  | 48 | 72 | .2 | .2 | 10.8 |
|  | 49 | 71 | .2 | .2 | 11.0 |
|  | 50 | 66 | .2 | .2 | 11.2 |
|  | 51 | 75 | .2 | .2 | 11.5 |
|  | 52 | 68 | .2 | .2 | 11.7 |
|  | 53 | 69 | .2 | .2 | 11.9 |
|  | 54 | 70 | .2 | .2 | 12.1 |
|  | 55 | 59 | .2 | .2 | 12.3 |
|  | 56 | 71 | .2 | .2 | 12.6 |
|  | 57 | 75 | .2 | .2 | 12.8 |
|  | 58 | 69 | .2 | .2 | 13.0 |
|  | 59 | 59 | .2 | .2 | 13.2 |
|  | 60 | 74 | .2 | .2 | 13.5 |
|  | 61 | 54 | .2 | .2 | 13.7 |
|  | 62 | 65 | .2 | .2 | 13.9 |
|  | 63 | 74 | .2 | .2 | 14.1 |
|  | 64 | 58 | .2 | .2 | 14.3 |
|  | 65 | 61 | .2 | .2 | 14.5 |
|  | 66 | 72 | .2 | .2 | 14.7 |
|  | 67 | 66 | .2 | .2 | 14.9 |
|  | 68 | 57 | .2 | .2 | 15.1 |
|  | 69 | 68 | .2 | .2 | 15.4 |
|  | 70 | 68 | .2 | .2 | 15.6 |
|  | 71 | 58 | .2 | .2 | 15.8 |
|  | 72 | 59 | .2 | .2 | 16.0 |
|  | 73 | 68 | .2 | .2 | 16.2 |
|  | 74 | 57 | .2 | .2 | 16.4 |
|  | 75 | 56 | .2 | .2 | 16.6 |
|  | 76 | 66 | .2 | .2 | 16.8 |
|  | 77 | 52 | .2 | .2 | 16.9 |
|  | 78 | 61 | .2 | .2 | 17.1 |
|  | 79 | 71 | .2 | .2 | 17.4 |
|  | 80 | 75 | .2 | .2 | 17.6 |
|  | 81 | 55 | .2 | .2 | 17.8 |
|  | 82 | 56 | .2 | .2 | 18.0 |
|  | 83 | 59 | .2 | .2 | 18.2 |
|  | 84 | 61 | .2 | .2 | 18.4 |
|  | 85 | 54 | .2 | .2 | 18.6 |
|  | 86 | 63 | .2 | .2 | 18.8 |
|  | 87 | 50 | .2 | .2 | 18.9 |
|  | 88 | 65 | .2 | .2 | 19.1 |
|  | 89 | 59 | .2 | .2 | 19.3 |
|  | 90 | 66 | .2 | .2 | 19.5 |
|  | 91 | 64 | .2 | .2 | 19.8 |
|  | 92 | 50 | .2 | .2 | 19.9 |
|  | 93 | 71 | .2 | .2 | 20.1 |
|  | 94 | 49 | .2 | .2 | 20.3 |
|  | 95 | 60 | .2 | .2 | 20.5 |
|  | 96 | 52 | .2 | .2 | 20.7 |
|  | 97 | 60 | .2 | .2 | 20.9 |
|  | 98 | 55 | .2 | .2 | 21.1 |
|  | 99 | 49 | .2 | .2 | 21.2 |
|  | 100 | 52 | .2 | .2 | 21.4 |
|  | 101 | 58 | .2 | .2 | 21.6 |
|  | 102 | 80 | .3 | .3 | 21.8 |
|  | 103 | 51 | .2 | .2 | 22.0 |
|  | 104 | 66 | .2 | .2 | 22.2 |
|  | 105 | 69 | .2 | .2 | 22.4 |
|  | 106 | 72 | .2 | .2 | 22.7 |
|  | 107 | 55 | .2 | .2 | 22.9 |
|  | 108 | 62 | .2 | .2 | 23.1 |
|  | 109 | 65 | .2 | .2 | 23.3 |
|  | 110 | 50 | .2 | .2 | 23.4 |
|  | 111 | 64 | .2 | .2 | 23.6 |
|  | 112 | 36 | .1 | .1 | 23.8 |
|  | 113 | 65 | .2 | .2 | 24.0 |
|  | 114 | 65 | .2 | .2 | 24.2 |
|  | 115 | 55 | .2 | .2 | 24.4 |
|  | 116 | 61 | .2 | .2 | 24.6 |
|  | 117 | 51 | .2 | .2 | 24.7 |
|  | 118 | 42 | .1 | .1 | 24.9 |
|  | 119 | 68 | .2 | .2 | 25.1 |
|  | 120 | 48 | .2 | .2 | 25.3 |
|  | 121 | 53 | .2 | .2 | 25.4 |
|  | 122 | 55 | .2 | .2 | 25.6 |
|  | 123 | 58 | .2 | .2 | 25.8 |
|  | 124 | 53 | .2 | .2 | 26.0 |
|  | 125 | 50 | .2 | .2 | 26.1 |
|  | 126 | 52 | .2 | .2 | 26.3 |
|  | 127 | 44 | .1 | .1 | 26.4 |
|  | 128 | 49 | .2 | .2 | 26.6 |
|  | 129 | 52 | .2 | .2 | 26.8 |
|  | 130 | 56 | .2 | .2 | 27.0 |
|  | 131 | 47 | .2 | .2 | 27.1 |
|  | 132 | 58 | .2 | .2 | 27.3 |
|  | 133 | 55 | .2 | .2 | 27.5 |
|  | 134 | 58 | .2 | .2 | 27.7 |
|  | 135 | 50 | .2 | .2 | 27.8 |
|  | 136 | 49 | .2 | .2 | 28.0 |
|  | 137 | 51 | .2 | .2 | 28.2 |
|  | 138 | 49 | .2 | .2 | 28.3 |
|  | 139 | 61 | .2 | .2 | 28.5 |
|  | 140 | 40 | .1 | .1 | 28.7 |
|  | 141 | 58 | .2 | .2 | 28.8 |
|  | 142 | 59 | .2 | .2 | 29.0 |
|  | 143 | 50 | .2 | .2 | 29.2 |
|  | 144 | 49 | .2 | .2 | 29.4 |
|  | 145 | 49 | .2 | .2 | 29.5 |
|  | 146 | 42 | .1 | .1 | 29.7 |
|  | 147 | 49 | .2 | .2 | 29.8 |
|  | 148 | 52 | .2 | .2 | 30.0 |
|  | 149 | 53 | .2 | .2 | 30.2 |
|  | 150 | 36 | .1 | .1 | 30.3 |
|  | 151 | 30 | .1 | .1 | 30.4 |
|  | 152 | 39 | .1 | .1 | 30.5 |
|  | 153 | 37 | .1 | .1 | 30.6 |
|  | 154 | 35 | .1 | .1 | 30.7 |
|  | 155 | 51 | .2 | .2 | 30.9 |
|  | 156 | 60 | .2 | .2 | 31.1 |
|  | 157 | 39 | .1 | .1 | 31.2 |
|  | 158 | 44 | .1 | .1 | 31.4 |
|  | 159 | 59 | .2 | .2 | 31.6 |
|  | 160 | 48 | .2 | .2 | 31.7 |
|  | 161 | 45 | .1 | .1 | 31.9 |
|  | 162 | 52 | .2 | .2 | 32.0 |
|  | 163 | 46 | .2 | .2 | 32.2 |
|  | 164 | 49 | .2 | .2 | 32.4 |
|  | 165 | 46 | .2 | .2 | 32.5 |
|  | 166 | 50 | .2 | .2 | 32.7 |
|  | 167 | 59 | .2 | .2 | 32.9 |
|  | 168 | 58 | .2 | .2 | 33.1 |
|  | 169 | 48 | .2 | .2 | 33.2 |
|  | 170 | 41 | .1 | .1 | 33.3 |
|  | 171 | 50 | .2 | .2 | 33.5 |
|  | 172 | 49 | .2 | .2 | 33.7 |
|  | 173 | 48 | .2 | .2 | 33.8 |
|  | 174 | 46 | .2 | .2 | 34.0 |
|  | 175 | 49 | .2 | .2 | 34.1 |
|  | 176 | 36 | .1 | .1 | 34.3 |
|  | 177 | 42 | .1 | .1 | 34.4 |
|  | 178 | 43 | .1 | .1 | 34.5 |
|  | 179 | 42 | .1 | .1 | 34.7 |
|  | 180 | 42 | .1 | .1 | 34.8 |
|  | 181 | 47 | .2 | .2 | 35.0 |
|  | 182 | 52 | .2 | .2 | 35.1 |
|  | 183 | 51 | .2 | .2 | 35.3 |
|  | 184 | 33 | .1 | .1 | 35.4 |
|  | 185 | 38 | .1 | .1 | 35.5 |
|  | 186 | 39 | .1 | .1 | 35.7 |
|  | 187 | 38 | .1 | .1 | 35.8 |
|  | 188 | 45 | .1 | .1 | 35.9 |
|  | 189 | 33 | .1 | .1 | 36.0 |
|  | 190 | 43 | .1 | .1 | 36.2 |
|  | 191 | 48 | .2 | .2 | 36.3 |
|  | 192 | 45 | .1 | .1 | 36.5 |
|  | 193 | 56 | .2 | .2 | 36.7 |
|  | 194 | 43 | .1 | .1 | 36.8 |
|  | 195 | 44 | .1 | .1 | 37.0 |
|  | 196 | 43 | .1 | .1 | 37.1 |
|  | 197 | 41 | .1 | .1 | 37.2 |
|  | 198 | 39 | .1 | .1 | 37.4 |
|  | 199 | 51 | .2 | .2 | 37.5 |
|  | 200 | 44 | .1 | .1 | 37.7 |
|  | 201 | 37 | .1 | .1 | 37.8 |
|  | 202 | 44 | .1 | .1 | 37.9 |
|  | 203 | 37 | .1 | .1 | 38.1 |
|  | 204 | 44 | .1 | .1 | 38.2 |
|  | 205 | 46 | .2 | .2 | 38.3 |
|  | 206 | 43 | .1 | .1 | 38.5 |
|  | 207 | 39 | .1 | .1 | 38.6 |
|  | 208 | 48 | .2 | .2 | 38.8 |
|  | 209 | 40 | .1 | .1 | 38.9 |
|  | 210 | 37 | .1 | .1 | 39.0 |
|  | 211 | 56 | .2 | .2 | 39.2 |
|  | 212 | 43 | .1 | .1 | 39.4 |
|  | 213 | 51 | .2 | .2 | 39.5 |
|  | 214 | 45 | .1 | .1 | 39.7 |
|  | 215 | 35 | .1 | .1 | 39.8 |
|  | 216 | 40 | .1 | .1 | 39.9 |
|  | 217 | 38 | .1 | .1 | 40.0 |
|  | 218 | 38 | .1 | .1 | 40.2 |
|  | 219 | 40 | .1 | .1 | 40.3 |
|  | 220 | 37 | .1 | .1 | 40.4 |
|  | 221 | 41 | .1 | .1 | 40.5 |
|  | 222 | 37 | .1 | .1 | 40.7 |
|  | 223 | 38 | .1 | .1 | 40.8 |
|  | 224 | 43 | .1 | .1 | 40.9 |
|  | 225 | 38 | .1 | .1 | 41.1 |
|  | 226 | 27 | .1 | .1 | 41.1 |
|  | 227 | 39 | .1 | .1 | 41.3 |
|  | 228 | 34 | .1 | .1 | 41.4 |
|  | 229 | 41 | .1 | .1 | 41.5 |
|  | 230 | 45 | .1 | .1 | 41.7 |
|  | 231 | 45 | .1 | .1 | 41.8 |
|  | 232 | 37 | .1 | .1 | 41.9 |
|  | 233 | 31 | .1 | .1 | 42.0 |
|  | 234 | 37 | .1 | .1 | 42.2 |
|  | 235 | 49 | .2 | .2 | 42.3 |
|  | 236 | 34 | .1 | .1 | 42.4 |
|  | 237 | 43 | .1 | .1 | 42.6 |
|  | 238 | 46 | .2 | .2 | 42.7 |
|  | 239 | 45 | .1 | .1 | 42.9 |
|  | 240 | 45 | .1 | .1 | 43.0 |
|  | 241 | 49 | .2 | .2 | 43.2 |
|  | 242 | 48 | .2 | .2 | 43.3 |
|  | 243 | 31 | .1 | .1 | 43.4 |
|  | 244 | 44 | .1 | .1 | 43.6 |
|  | 245 | 34 | .1 | .1 | 43.7 |
|  | 246 | 41 | .1 | .1 | 43.8 |
|  | 247 | 38 | .1 | .1 | 43.9 |
|  | 248 | 42 | .1 | .1 | 44.1 |
|  | 249 | 41 | .1 | .1 | 44.2 |
|  | 250 | 33 | .1 | .1 | 44.3 |
|  | 251 | 52 | .2 | .2 | 44.5 |
|  | 252 | 37 | .1 | .1 | 44.6 |
|  | 253 | 36 | .1 | .1 | 44.7 |
|  | 254 | 35 | .1 | .1 | 44.8 |
|  | 255 | 40 | .1 | .1 | 45.0 |
|  | 256 | 38 | .1 | .1 | 45.1 |
|  | 257 | 34 | .1 | .1 | 45.2 |
|  | 258 | 37 | .1 | .1 | 45.3 |
|  | 259 | 33 | .1 | .1 | 45.4 |
|  | 260 | 42 | .1 | .1 | 45.6 |
|  | 261 | 22 | .1 | .1 | 45.7 |
|  | 262 | 33 | .1 | .1 | 45.8 |
|  | 263 | 37 | .1 | .1 | 45.9 |
|  | 264 | 32 | .1 | .1 | 46.0 |
|  | 265 | 50 | .2 | .2 | 46.2 |
|  | 266 | 36 | .1 | .1 | 46.3 |
|  | 267 | 38 | .1 | .1 | 46.4 |
|  | 268 | 30 | .1 | .1 | 46.5 |
|  | 269 | 38 | .1 | .1 | 46.6 |
|  | 270 | 44 | .1 | .1 | 46.8 |
|  | 271 | 32 | .1 | .1 | 46.9 |
|  | 272 | 37 | .1 | .1 | 47.0 |
|  | 273 | 53 | .2 | .2 | 47.2 |
|  | 274 | 37 | .1 | .1 | 47.3 |
|  | 275 | 41 | .1 | .1 | 47.4 |
|  | 276 | 38 | .1 | .1 | 47.5 |
|  | 277 | 36 | .1 | .1 | 47.7 |
|  | 278 | 41 | .1 | .1 | 47.8 |
|  | 279 | 34 | .1 | .1 | 47.9 |
|  | 280 | 39 | .1 | .1 | 48.0 |
|  | 281 | 28 | .1 | .1 | 48.1 |
|  | 282 | 44 | .1 | .1 | 48.3 |
|  | 283 | 36 | .1 | .1 | 48.4 |
|  | 284 | 48 | .2 | .2 | 48.5 |
|  | 285 | 38 | .1 | .1 | 48.7 |
|  | 286 | 34 | .1 | .1 | 48.8 |
|  | 287 | 31 | .1 | .1 | 48.9 |
|  | 288 | 41 | .1 | .1 | 49.0 |
|  | 289 | 41 | .1 | .1 | 49.1 |
|  | 290 | 44 | .1 | .1 | 49.3 |
|  | 291 | 33 | .1 | .1 | 49.4 |
|  | 292 | 33 | .1 | .1 | 49.5 |
|  | 293 | 34 | .1 | .1 | 49.6 |
|  | 294 | 26 | .1 | .1 | 49.7 |
|  | 295 | 38 | .1 | .1 | 49.8 |
|  | 296 | 30 | .1 | .1 | 49.9 |
|  | 297 | 34 | .1 | .1 | 50.0 |
|  | 298 | 25 | .1 | .1 | 50.1 |
|  | 299 | 33 | .1 | .1 | 50.2 |
|  | 300 | 33 | .1 | .1 | 50.3 |
|  | 301 | 21 | .1 | .1 | 50.4 |
|  | 302 | 27 | .1 | .1 | 50.5 |
|  | 303 | 27 | .1 | .1 | 50.6 |
|  | 304 | 39 | .1 | .1 | 50.7 |
|  | 305 | 32 | .1 | .1 | 50.8 |
|  | 306 | 30 | .1 | .1 | 50.9 |
|  | 307 | 30 | .1 | .1 | 51.0 |
|  | 308 | 36 | .1 | .1 | 51.1 |
|  | 309 | 32 | .1 | .1 | 51.2 |
|  | 310 | 32 | .1 | .1 | 51.3 |
|  | 311 | 41 | .1 | .1 | 51.5 |
|  | 312 | 34 | .1 | .1 | 51.6 |
|  | 313 | 30 | .1 | .1 | 51.7 |
|  | 314 | 32 | .1 | .1 | 51.8 |
|  | 315 | 24 | .1 | .1 | 51.9 |
|  | 316 | 26 | .1 | .1 | 51.9 |
|  | 317 | 26 | .1 | .1 | 52.0 |
|  | 318 | 32 | .1 | .1 | 52.1 |
|  | 319 | 30 | .1 | .1 | 52.2 |
|  | 320 | 19 | .1 | .1 | 52.3 |
|  | 321 | 32 | .1 | .1 | 52.4 |
|  | 322 | 26 | .1 | .1 | 52.5 |
|  | 323 | 31 | .1 | .1 | 52.6 |
|  | 324 | 34 | .1 | .1 | 52.7 |
|  | 325 | 30 | .1 | .1 | 52.8 |
|  | 326 | 32 | .1 | .1 | 52.9 |
|  | 327 | 35 | .1 | .1 | 53.0 |
|  | 328 | 31 | .1 | .1 | 53.1 |
|  | 329 | 30 | .1 | .1 | 53.2 |
|  | 330 | 31 | .1 | .1 | 53.3 |
|  | 331 | 23 | .1 | .1 | 53.4 |
|  | 332 | 25 | .1 | .1 | 53.5 |
|  | 333 | 30 | .1 | .1 | 53.6 |
|  | 334 | 33 | .1 | .1 | 53.7 |
|  | 335 | 39 | .1 | .1 | 53.8 |
|  | 336 | 33 | .1 | .1 | 53.9 |
|  | 337 | 30 | .1 | .1 | 54.0 |
|  | 338 | 23 | .1 | .1 | 54.1 |
|  | 339 | 38 | .1 | .1 | 54.2 |
|  | 340 | 18 | .1 | .1 | 54.3 |
|  | 341 | 33 | .1 | .1 | 54.4 |
|  | 342 | 40 | .1 | .1 | 54.5 |
|  | 343 | 27 | .1 | .1 | 54.6 |
|  | 344 | 43 | .1 | .1 | 54.7 |
|  | 345 | 19 | .1 | .1 | 54.8 |
|  | 346 | 31 | .1 | .1 | 54.9 |
|  | 347 | 34 | .1 | .1 | 55.0 |
|  | 348 | 35 | .1 | .1 | 55.1 |
|  | 349 | 37 | .1 | .1 | 55.3 |
|  | 350 | 42 | .1 | .1 | 55.4 |
|  | 351 | 29 | .1 | .1 | 55.5 |
|  | 352 | 34 | .1 | .1 | 55.6 |
|  | 353 | 36 | .1 | .1 | 55.7 |
|  | 354 | 33 | .1 | .1 | 55.8 |
|  | 355 | 23 | .1 | .1 | 55.9 |
|  | 356 | 23 | .1 | .1 | 56.0 |
|  | 357 | 42 | .1 | .1 | 56.1 |
|  | 358 | 38 | .1 | .1 | 56.2 |
|  | 359 | 20 | .1 | .1 | 56.3 |
|  | 360 | 33 | .1 | .1 | 56.4 |
|  | 361 | 29 | .1 | .1 | 56.5 |
|  | 362 | 35 | .1 | .1 | 56.6 |
|  | 363 | 28 | .1 | .1 | 56.7 |
|  | 364 | 23 | .1 | .1 | 56.8 |
|  | 365 | 30 | .1 | .1 | 56.9 |
|  | 366 | 25 | .1 | .1 | 57.0 |
|  | 367 | 29 | .1 | .1 | 57.1 |
|  | 368 | 30 | .1 | .1 | 57.2 |
|  | 369 | 31 | .1 | .1 | 57.3 |
|  | 370 | 29 | .1 | .1 | 57.4 |
|  | 371 | 26 | .1 | .1 | 57.4 |
|  | 372 | 21 | .1 | .1 | 57.5 |
|  | 373 | 27 | .1 | .1 | 57.6 |
|  | 374 | 21 | .1 | .1 | 57.7 |
|  | 375 | 26 | .1 | .1 | 57.8 |
|  | 376 | 38 | .1 | .1 | 57.9 |
|  | 377 | 33 | .1 | .1 | 58.0 |
|  | 378 | 31 | .1 | .1 | 58.1 |
|  | 379 | 30 | .1 | .1 | 58.2 |
|  | 380 | 29 | .1 | .1 | 58.3 |
|  | 381 | 25 | .1 | .1 | 58.4 |
|  | 382 | 26 | .1 | .1 | 58.4 |
|  | 383 | 32 | .1 | .1 | 58.6 |
|  | 384 | 28 | .1 | .1 | 58.6 |
|  | 385 | 28 | .1 | .1 | 58.7 |
|  | 386 | 26 | .1 | .1 | 58.8 |
|  | 387 | 31 | .1 | .1 | 58.9 |
|  | 388 | 19 | .1 | .1 | 59.0 |
|  | 389 | 35 | .1 | .1 | 59.1 |
|  | 390 | 30 | .1 | .1 | 59.2 |
|  | 391 | 28 | .1 | .1 | 59.3 |
|  | 392 | 29 | .1 | .1 | 59.4 |
|  | 393 | 25 | .1 | .1 | 59.5 |
|  | 394 | 24 | .1 | .1 | 59.5 |
|  | 395 | 28 | .1 | .1 | 59.6 |
|  | 396 | 32 | .1 | .1 | 59.7 |
|  | 397 | 34 | .1 | .1 | 59.9 |
|  | 398 | 32 | .1 | .1 | 60.0 |
|  | 399 | 32 | .1 | .1 | 60.1 |
|  | 400 | 37 | .1 | .1 | 60.2 |
|  | 401 | 24 | .1 | .1 | 60.3 |
|  | 402 | 22 | .1 | .1 | 60.3 |
|  | 403 | 33 | .1 | .1 | 60.4 |
|  | 404 | 27 | .1 | .1 | 60.5 |
|  | 405 | 26 | .1 | .1 | 60.6 |
|  | 406 | 17 | .1 | .1 | 60.7 |
|  | 407 | 29 | .1 | .1 | 60.8 |
|  | 408 | 24 | .1 | .1 | 60.8 |
|  | 409 | 21 | .1 | .1 | 60.9 |
|  | 410 | 29 | .1 | .1 | 61.0 |
|  | 411 | 24 | .1 | .1 | 61.1 |
|  | 412 | 28 | .1 | .1 | 61.2 |
|  | 413 | 26 | .1 | .1 | 61.3 |
|  | 414 | 25 | .1 | .1 | 61.3 |
|  | 415 | 23 | .1 | .1 | 61.4 |
|  | 416 | 26 | .1 | .1 | 61.5 |
|  | 417 | 30 | .1 | .1 | 61.6 |
|  | 418 | 23 | .1 | .1 | 61.7 |
|  | 419 | 27 | .1 | .1 | 61.8 |
|  | 420 | 34 | .1 | .1 | 61.9 |
|  | 421 | 26 | .1 | .1 | 62.0 |
|  | 422 | 18 | .1 | .1 | 62.0 |
|  | 423 | 28 | .1 | .1 | 62.1 |
|  | 424 | 28 | .1 | .1 | 62.2 |
|  | 425 | 29 | .1 | .1 | 62.3 |
|  | 426 | 27 | .1 | .1 | 62.4 |
|  | 427 | 29 | .1 | .1 | 62.5 |
|  | 428 | 33 | .1 | .1 | 62.6 |
|  | 429 | 24 | .1 | .1 | 62.7 |
|  | 430 | 24 | .1 | .1 | 62.7 |
|  | 431 | 27 | .1 | .1 | 62.8 |
|  | 432 | 27 | .1 | .1 | 62.9 |
|  | 433 | 23 | .1 | .1 | 63.0 |
|  | 434 | 21 | .1 | .1 | 63.1 |
|  | 435 | 27 | .1 | .1 | 63.2 |
|  | 436 | 24 | .1 | .1 | 63.2 |
|  | 437 | 24 | .1 | .1 | 63.3 |
|  | 438 | 36 | .1 | .1 | 63.4 |
|  | 439 | 24 | .1 | .1 | 63.5 |
|  | 440 | 29 | .1 | .1 | 63.6 |
|  | 441 | 32 | .1 | .1 | 63.7 |
|  | 442 | 27 | .1 | .1 | 63.8 |
|  | 443 | 26 | .1 | .1 | 63.9 |
|  | 444 | 27 | .1 | .1 | 64.0 |
|  | 445 | 33 | .1 | .1 | 64.1 |
|  | 446 | 24 | .1 | .1 | 64.2 |
|  | 447 | 29 | .1 | .1 | 64.3 |
|  | 448 | 22 | .1 | .1 | 64.3 |
|  | 449 | 24 | .1 | .1 | 64.4 |
|  | 450 | 25 | .1 | .1 | 64.5 |
|  | 451 | 27 | .1 | .1 | 64.6 |
|  | 452 | 31 | .1 | .1 | 64.7 |
|  | 453 | 15 | .0 | .0 | 64.7 |
|  | 454 | 28 | .1 | .1 | 64.8 |
|  | 455 | 23 | .1 | .1 | 64.9 |
|  | 456 | 20 | .1 | .1 | 65.0 |
|  | 457 | 16 | .1 | .1 | 65.0 |
|  | 458 | 23 | .1 | .1 | 65.1 |
|  | 459 | 28 | .1 | .1 | 65.2 |
|  | 460 | 19 | .1 | .1 | 65.2 |
|  | 461 | 27 | .1 | .1 | 65.3 |
|  | 462 | 16 | .1 | .1 | 65.4 |
|  | 463 | 16 | .1 | .1 | 65.4 |
|  | 464 | 27 | .1 | .1 | 65.5 |
|  | 465 | 24 | .1 | .1 | 65.6 |
|  | 466 | 24 | .1 | .1 | 65.7 |
|  | 467 | 23 | .1 | .1 | 65.8 |
|  | 468 | 27 | .1 | .1 | 65.8 |
|  | 469 | 28 | .1 | .1 | 65.9 |
|  | 470 | 25 | .1 | .1 | 66.0 |
|  | 471 | 21 | .1 | .1 | 66.1 |
|  | 472 | 18 | .1 | .1 | 66.1 |
|  | 473 | 21 | .1 | .1 | 66.2 |
|  | 474 | 23 | .1 | .1 | 66.3 |
|  | 475 | 33 | .1 | .1 | 66.4 |
|  | 476 | 17 | .1 | .1 | 66.4 |
|  | 477 | 23 | .1 | .1 | 66.5 |
|  | 478 | 20 | .1 | .1 | 66.6 |
|  | 479 | 28 | .1 | .1 | 66.7 |
|  | 480 | 30 | .1 | .1 | 66.8 |
|  | 481 | 16 | .1 | .1 | 66.8 |
|  | 482 | 33 | .1 | .1 | 66.9 |
|  | 483 | 22 | .1 | .1 | 67.0 |
|  | 484 | 22 | .1 | .1 | 67.1 |
|  | 485 | 24 | .1 | .1 | 67.2 |
|  | 486 | 22 | .1 | .1 | 67.2 |
|  | 487 | 25 | .1 | .1 | 67.3 |
|  | 488 | 16 | .1 | .1 | 67.4 |
|  | 489 | 22 | .1 | .1 | 67.4 |
|  | 490 | 26 | .1 | .1 | 67.5 |
|  | 491 | 24 | .1 | .1 | 67.6 |
|  | 492 | 17 | .1 | .1 | 67.7 |
|  | 493 | 24 | .1 | .1 | 67.7 |
|  | 494 | 22 | .1 | .1 | 67.8 |
|  | 495 | 21 | .1 | .1 | 67.9 |
|  | 496 | 27 | .1 | .1 | 68.0 |
|  | 497 | 19 | .1 | .1 | 68.0 |
|  | 498 | 23 | .1 | .1 | 68.1 |
|  | 499 | 20 | .1 | .1 | 68.2 |
|  | 500 | 19 | .1 | .1 | 68.2 |
|  | 501 | 17 | .1 | .1 | 68.3 |
|  | 502 | 17 | .1 | .1 | 68.3 |
|  | 503 | 29 | .1 | .1 | 68.4 |
|  | 504 | 25 | .1 | .1 | 68.5 |
|  | 505 | 18 | .1 | .1 | 68.6 |
|  | 506 | 19 | .1 | .1 | 68.6 |
|  | 507 | 25 | .1 | .1 | 68.7 |
|  | 508 | 15 | .0 | .0 | 68.8 |
|  | 509 | 23 | .1 | .1 | 68.8 |
|  | 510 | 21 | .1 | .1 | 68.9 |
|  | 511 | 17 | .1 | .1 | 69.0 |
|  | 512 | 19 | .1 | .1 | 69.0 |
|  | 513 | 19 | .1 | .1 | 69.1 |
|  | 514 | 11 | .0 | .0 | 69.1 |
|  | 515 | 20 | .1 | .1 | 69.2 |
|  | 516 | 16 | .1 | .1 | 69.3 |
|  | 517 | 27 | .1 | .1 | 69.3 |
|  | 518 | 21 | .1 | .1 | 69.4 |
|  | 519 | 20 | .1 | .1 | 69.5 |
|  | 520 | 16 | .1 | .1 | 69.5 |
|  | 521 | 25 | .1 | .1 | 69.6 |
|  | 522 | 23 | .1 | .1 | 69.7 |
|  | 523 | 21 | .1 | .1 | 69.8 |
|  | 524 | 18 | .1 | .1 | 69.8 |
|  | 525 | 15 | .0 | .0 | 69.9 |
|  | 526 | 16 | .1 | .1 | 69.9 |
|  | 527 | 21 | .1 | .1 | 70.0 |
|  | 528 | 24 | .1 | .1 | 70.1 |
|  | 529 | 18 | .1 | .1 | 70.1 |
|  | 530 | 17 | .1 | .1 | 70.2 |
|  | 531 | 14 | .0 | .0 | 70.2 |
|  | 532 | 15 | .0 | .0 | 70.3 |
|  | 533 | 23 | .1 | .1 | 70.3 |
|  | 534 | 16 | .1 | .1 | 70.4 |
|  | 535 | 20 | .1 | .1 | 70.5 |
|  | 536 | 17 | .1 | .1 | 70.5 |
|  | 537 | 18 | .1 | .1 | 70.6 |
|  | 538 | 17 | .1 | .1 | 70.6 |
|  | 539 | 12 | .0 | .0 | 70.7 |
|  | 540 | 21 | .1 | .1 | 70.7 |
|  | 541 | 19 | .1 | .1 | 70.8 |
|  | 542 | 18 | .1 | .1 | 70.9 |
|  | 543 | 18 | .1 | .1 | 70.9 |
|  | 544 | 21 | .1 | .1 | 71.0 |
|  | 545 | 20 | .1 | .1 | 71.1 |
|  | 546 | 25 | .1 | .1 | 71.1 |
|  | 547 | 17 | .1 | .1 | 71.2 |
|  | 548 | 17 | .1 | .1 | 71.2 |
|  | 549 | 16 | .1 | .1 | 71.3 |
|  | 550 | 22 | .1 | .1 | 71.4 |
|  | 551 | 12 | .0 | .0 | 71.4 |
|  | 552 | 13 | .0 | .0 | 71.5 |
|  | 553 | 12 | .0 | .0 | 71.5 |
|  | 554 | 18 | .1 | .1 | 71.6 |
|  | 555 | 20 | .1 | .1 | 71.6 |
|  | 556 | 17 | .1 | .1 | 71.7 |
|  | 557 | 18 | .1 | .1 | 71.7 |
|  | 558 | 11 | .0 | .0 | 71.8 |
|  | 559 | 19 | .1 | .1 | 71.8 |
|  | 560 | 15 | .0 | .0 | 71.9 |
|  | 561 | 12 | .0 | .0 | 71.9 |
|  | 562 | 15 | .0 | .0 | 72.0 |
|  | 563 | 18 | .1 | .1 | 72.0 |
|  | 564 | 12 | .0 | .0 | 72.1 |
|  | 565 | 20 | .1 | .1 | 72.1 |
|  | 566 | 12 | .0 | .0 | 72.2 |
|  | 567 | 10 | .0 | .0 | 72.2 |
|  | 568 | 17 | .1 | .1 | 72.3 |
|  | 569 | 15 | .0 | .0 | 72.3 |
|  | 570 | 7 | .0 | .0 | 72.3 |
|  | 571 | 14 | .0 | .0 | 72.4 |
|  | 572 | 17 | .1 | .1 | 72.4 |
|  | 573 | 22 | .1 | .1 | 72.5 |
|  | 574 | 24 | .1 | .1 | 72.6 |
|  | 575 | 15 | .0 | .0 | 72.6 |
|  | 576 | 14 | .0 | .0 | 72.7 |
|  | 577 | 16 | .1 | .1 | 72.7 |
|  | 578 | 21 | .1 | .1 | 72.8 |
|  | 579 | 16 | .1 | .1 | 72.9 |
|  | 580 | 17 | .1 | .1 | 72.9 |
|  | 581 | 16 | .1 | .1 | 73.0 |
|  | 582 | 8 | .0 | .0 | 73.0 |
|  | 583 | 18 | .1 | .1 | 73.0 |
|  | 584 | 24 | .1 | .1 | 73.1 |
|  | 585 | 17 | .1 | .1 | 73.2 |
|  | 586 | 21 | .1 | .1 | 73.2 |
|  | 587 | 14 | .0 | .0 | 73.3 |
|  | 588 | 18 | .1 | .1 | 73.4 |
|  | 589 | 15 | .0 | .0 | 73.4 |
|  | 590 | 20 | .1 | .1 | 73.5 |
|  | 591 | 24 | .1 | .1 | 73.5 |
|  | 592 | 11 | .0 | .0 | 73.6 |
|  | 593 | 15 | .0 | .0 | 73.6 |
|  | 594 | 15 | .0 | .0 | 73.7 |
|  | 595 | 15 | .0 | .0 | 73.7 |
|  | 596 | 21 | .1 | .1 | 73.8 |
|  | 597 | 10 | .0 | .0 | 73.8 |
|  | 598 | 14 | .0 | .0 | 73.9 |
|  | 599 | 21 | .1 | .1 | 73.9 |
|  | 600 | 13 | .0 | .0 | 74.0 |
|  | 601 | 19 | .1 | .1 | 74.0 |
|  | 602 | 22 | .1 | .1 | 74.1 |
|  | 603 | 19 | .1 | .1 | 74.2 |
|  | 604 | 23 | .1 | .1 | 74.3 |
|  | 605 | 15 | .0 | .0 | 74.3 |
|  | 606 | 21 | .1 | .1 | 74.4 |
|  | 607 | 25 | .1 | .1 | 74.5 |
|  | 608 | 13 | .0 | .0 | 74.5 |
|  | 609 | 18 | .1 | .1 | 74.6 |
|  | 610 | 22 | .1 | .1 | 74.6 |
|  | 611 | 12 | .0 | .0 | 74.7 |
|  | 612 | 17 | .1 | .1 | 74.7 |
|  | 613 | 21 | .1 | .1 | 74.8 |
|  | 614 | 18 | .1 | .1 | 74.9 |
|  | 615 | 15 | .0 | .0 | 74.9 |
|  | 616 | 15 | .0 | .0 | 75.0 |
|  | 617 | 22 | .1 | .1 | 75.0 |
|  | 618 | 15 | .0 | .0 | 75.1 |
|  | 619 | 22 | .1 | .1 | 75.1 |
|  | 620 | 13 | .0 | .0 | 75.2 |
|  | 621 | 13 | .0 | .0 | 75.2 |
|  | 622 | 11 | .0 | .0 | 75.3 |
|  | 623 | 15 | .0 | .0 | 75.3 |
|  | 624 | 21 | .1 | .1 | 75.4 |
|  | 625 | 16 | .1 | .1 | 75.4 |
|  | 626 | 17 | .1 | .1 | 75.5 |
|  | 627 | 18 | .1 | .1 | 75.6 |
|  | 628 | 15 | .0 | .0 | 75.6 |
|  | 629 | 15 | .0 | .0 | 75.6 |
|  | 630 | 18 | .1 | .1 | 75.7 |
|  | 631 | 11 | .0 | .0 | 75.7 |
|  | 632 | 20 | .1 | .1 | 75.8 |
|  | 633 | 14 | .0 | .0 | 75.9 |
|  | 634 | 13 | .0 | .0 | 75.9 |
|  | 635 | 19 | .1 | .1 | 76.0 |
|  | 636 | 13 | .0 | .0 | 76.0 |
|  | 637 | 20 | .1 | .1 | 76.1 |
|  | 638 | 20 | .1 | .1 | 76.1 |
|  | 639 | 15 | .0 | .0 | 76.2 |
|  | 640 | 23 | .1 | .1 | 76.3 |
|  | 641 | 16 | .1 | .1 | 76.3 |
|  | 642 | 13 | .0 | .0 | 76.4 |
|  | 643 | 15 | .0 | .0 | 76.4 |
|  | 644 | 18 | .1 | .1 | 76.5 |
|  | 645 | 18 | .1 | .1 | 76.5 |
|  | 646 | 20 | .1 | .1 | 76.6 |
|  | 647 | 14 | .0 | .0 | 76.6 |
|  | 648 | 19 | .1 | .1 | 76.7 |
|  | 649 | 8 | .0 | .0 | 76.7 |
|  | 650 | 14 | .0 | .0 | 76.8 |
|  | 651 | 14 | .0 | .0 | 76.8 |
|  | 652 | 12 | .0 | .0 | 76.9 |
|  | 653 | 18 | .1 | .1 | 76.9 |
|  | 654 | 16 | .1 | .1 | 77.0 |
|  | 655 | 19 | .1 | .1 | 77.0 |
|  | 656 | 11 | .0 | .0 | 77.1 |
|  | 657 | 10 | .0 | .0 | 77.1 |
|  | 658 | 13 | .0 | .0 | 77.1 |
|  | 659 | 18 | .1 | .1 | 77.2 |
|  | 660 | 23 | .1 | .1 | 77.3 |
|  | 661 | 13 | .0 | .0 | 77.3 |
|  | 662 | 15 | .0 | .0 | 77.4 |
|  | 663 | 16 | .1 | .1 | 77.4 |
|  | 664 | 22 | .1 | .1 | 77.5 |
|  | 665 | 20 | .1 | .1 | 77.6 |
|  | 666 | 16 | .1 | .1 | 77.6 |
|  | 667 | 18 | .1 | .1 | 77.7 |
|  | 668 | 17 | .1 | .1 | 77.7 |
|  | 669 | 14 | .0 | .0 | 77.8 |
|  | 670 | 16 | .1 | .1 | 77.8 |
|  | 671 | 15 | .0 | .0 | 77.9 |
|  | 672 | 17 | .1 | .1 | 77.9 |
|  | 673 | 11 | .0 | .0 | 78.0 |
|  | 674 | 8 | .0 | .0 | 78.0 |
|  | 675 | 12 | .0 | .0 | 78.0 |
|  | 676 | 9 | .0 | .0 | 78.1 |
|  | 677 | 16 | .1 | .1 | 78.1 |
|  | 678 | 12 | .0 | .0 | 78.1 |
|  | 679 | 13 | .0 | .0 | 78.2 |
|  | 680 | 14 | .0 | .0 | 78.2 |
|  | 681 | 24 | .1 | .1 | 78.3 |
|  | 682 | 14 | .0 | .0 | 78.4 |
|  | 683 | 11 | .0 | .0 | 78.4 |
|  | 684 | 12 | .0 | .0 | 78.4 |
|  | 685 | 13 | .0 | .0 | 78.5 |
|  | 686 | 12 | .0 | .0 | 78.5 |
|  | 687 | 12 | .0 | .0 | 78.6 |
|  | 688 | 12 | .0 | .0 | 78.6 |
|  | 689 | 14 | .0 | .0 | 78.6 |
|  | 690 | 9 | .0 | .0 | 78.7 |
|  | 691 | 14 | .0 | .0 | 78.7 |
|  | 692 | 13 | .0 | .0 | 78.8 |
|  | 693 | 24 | .1 | .1 | 78.8 |
|  | 694 | 16 | .1 | .1 | 78.9 |
|  | 695 | 12 | .0 | .0 | 78.9 |
|  | 696 | 8 | .0 | .0 | 79.0 |
|  | 697 | 12 | .0 | .0 | 79.0 |
|  | 698 | 13 | .0 | .0 | 79.0 |
|  | 699 | 17 | .1 | .1 | 79.1 |
|  | 700 | 11 | .0 | .0 | 79.1 |
|  | 701 | 14 | .0 | .0 | 79.2 |
|  | 702 | 14 | .0 | .0 | 79.2 |
|  | 703 | 6 | .0 | .0 | 79.2 |
|  | 704 | 13 | .0 | .0 | 79.3 |
|  | 705 | 9 | .0 | .0 | 79.3 |
|  | 706 | 9 | .0 | .0 | 79.3 |
|  | 707 | 11 | .0 | .0 | 79.4 |
|  | 708 | 11 | .0 | .0 | 79.4 |
|  | 709 | 9 | .0 | .0 | 79.4 |
|  | 710 | 16 | .1 | .1 | 79.5 |
|  | 711 | 16 | .1 | .1 | 79.5 |
|  | 712 | 12 | .0 | .0 | 79.6 |
|  | 713 | 17 | .1 | .1 | 79.6 |
|  | 714 | 7 | .0 | .0 | 79.7 |
|  | 715 | 20 | .1 | .1 | 79.7 |
|  | 716 | 16 | .1 | .1 | 79.8 |
|  | 717 | 10 | .0 | .0 | 79.8 |
|  | 718 | 14 | .0 | .0 | 79.9 |
|  | 719 | 11 | .0 | .0 | 79.9 |
|  | 720 | 13 | .0 | .0 | 79.9 |
|  | 721 | 19 | .1 | .1 | 80.0 |
|  | 722 | 16 | .1 | .1 | 80.1 |
|  | 723 | 14 | .0 | .0 | 80.1 |
|  | 724 | 7 | .0 | .0 | 80.1 |
|  | 725 | 12 | .0 | .0 | 80.2 |
|  | 726 | 19 | .1 | .1 | 80.2 |
|  | 727 | 16 | .1 | .1 | 80.3 |
|  | 728 | 15 | .0 | .0 | 80.3 |
|  | 729 | 5 | .0 | .0 | 80.3 |
|  | 730 | 7 | .0 | .0 | 80.4 |
|  | 731 | 6 | .0 | .0 | 80.4 |
|  | 732 | 9 | .0 | .0 | 80.4 |
|  | 733 | 14 | .0 | .0 | 80.5 |
|  | 734 | 9 | .0 | .0 | 80.5 |
|  | 735 | 12 | .0 | .0 | 80.5 |
|  | 736 | 6 | .0 | .0 | 80.5 |
|  | 737 | 10 | .0 | .0 | 80.6 |
|  | 738 | 10 | .0 | .0 | 80.6 |
|  | 739 | 10 | .0 | .0 | 80.6 |
|  | 740 | 14 | .0 | .0 | 80.7 |
|  | 741 | 16 | .1 | .1 | 80.7 |
|  | 742 | 13 | .0 | .0 | 80.8 |
|  | 743 | 9 | .0 | .0 | 80.8 |
|  | 744 | 13 | .0 | .0 | 80.9 |
|  | 745 | 14 | .0 | .0 | 80.9 |
|  | 746 | 11 | .0 | .0 | 80.9 |
|  | 747 | 16 | .1 | .1 | 81.0 |
|  | 748 | 11 | .0 | .0 | 81.0 |
|  | 749 | 6 | .0 | .0 | 81.0 |
|  | 750 | 13 | .0 | .0 | 81.1 |
|  | 751 | 9 | .0 | .0 | 81.1 |
|  | 752 | 7 | .0 | .0 | 81.1 |
|  | 753 | 19 | .1 | .1 | 81.2 |
|  | 754 | 13 | .0 | .0 | 81.2 |
|  | 755 | 18 | .1 | .1 | 81.3 |
|  | 756 | 14 | .0 | .0 | 81.4 |
|  | 757 | 7 | .0 | .0 | 81.4 |
|  | 758 | 6 | .0 | .0 | 81.4 |
|  | 759 | 12 | .0 | .0 | 81.4 |
|  | 760 | 9 | .0 | .0 | 81.5 |
|  | 761 | 12 | .0 | .0 | 81.5 |
|  | 762 | 8 | .0 | .0 | 81.5 |
|  | 763 | 16 | .1 | .1 | 81.6 |
|  | 764 | 13 | .0 | .0 | 81.6 |
|  | 765 | 7 | .0 | .0 | 81.6 |
|  | 766 | 10 | .0 | .0 | 81.7 |
|  | 767 | 7 | .0 | .0 | 81.7 |
|  | 768 | 7 | .0 | .0 | 81.7 |
|  | 769 | 3 | .0 | .0 | 81.7 |
|  | 770 | 16 | .1 | .1 | 81.8 |
|  | 771 | 8 | .0 | .0 | 81.8 |
|  | 772 | 10 | .0 | .0 | 81.8 |
|  | 773 | 4 | .0 | .0 | 81.9 |
|  | 774 | 10 | .0 | .0 | 81.9 |
|  | 775 | 13 | .0 | .0 | 81.9 |
|  | 776 | 10 | .0 | .0 | 82.0 |
|  | 777 | 7 | .0 | .0 | 82.0 |
|  | 778 | 16 | .1 | .1 | 82.0 |
|  | 779 | 12 | .0 | .0 | 82.1 |
|  | 780 | 8 | .0 | .0 | 82.1 |
|  | 781 | 14 | .0 | .0 | 82.2 |
|  | 782 | 14 | .0 | .0 | 82.2 |
|  | 783 | 11 | .0 | .0 | 82.2 |
|  | 784 | 8 | .0 | .0 | 82.3 |
|  | 785 | 18 | .1 | .1 | 82.3 |
|  | 786 | 9 | .0 | .0 | 82.3 |
|  | 787 | 11 | .0 | .0 | 82.4 |
|  | 788 | 15 | .0 | .0 | 82.4 |
|  | 789 | 9 | .0 | .0 | 82.5 |
|  | 790 | 12 | .0 | .0 | 82.5 |
|  | 791 | 10 | .0 | .0 | 82.5 |
|  | 792 | 11 | .0 | .0 | 82.6 |
|  | 793 | 10 | .0 | .0 | 82.6 |
|  | 794 | 6 | .0 | .0 | 82.6 |
|  | 795 | 14 | .0 | .0 | 82.7 |
|  | 796 | 9 | .0 | .0 | 82.7 |
|  | 797 | 9 | .0 | .0 | 82.7 |
|  | 798 | 8 | .0 | .0 | 82.8 |
|  | 799 | 3 | .0 | .0 | 82.8 |
|  | 800 | 8 | .0 | .0 | 82.8 |
|  | 801 | 10 | .0 | .0 | 82.8 |
|  | 802 | 8 | .0 | .0 | 82.8 |
|  | 803 | 6 | .0 | .0 | 82.9 |
|  | 804 | 7 | .0 | .0 | 82.9 |
|  | 805 | 14 | .0 | .0 | 82.9 |
|  | 806 | 13 | .0 | .0 | 83.0 |
|  | 807 | 10 | .0 | .0 | 83.0 |
|  | 808 | 7 | .0 | .0 | 83.0 |
|  | 809 | 10 | .0 | .0 | 83.1 |
|  | 810 | 4 | .0 | .0 | 83.1 |
|  | 811 | 9 | .0 | .0 | 83.1 |
|  | 812 | 8 | .0 | .0 | 83.1 |
|  | 813 | 9 | .0 | .0 | 83.2 |
|  | 814 | 13 | .0 | .0 | 83.2 |
|  | 815 | 11 | .0 | .0 | 83.2 |
|  | 816 | 16 | .1 | .1 | 83.3 |
|  | 817 | 5 | .0 | .0 | 83.3 |
|  | 818 | 10 | .0 | .0 | 83.3 |
|  | 819 | 7 | .0 | .0 | 83.4 |
|  | 820 | 14 | .0 | .0 | 83.4 |
|  | 821 | 10 | .0 | .0 | 83.4 |
|  | 822 | 7 | .0 | .0 | 83.5 |
|  | 823 | 6 | .0 | .0 | 83.5 |
|  | 824 | 11 | .0 | .0 | 83.5 |
|  | 825 | 11 | .0 | .0 | 83.6 |
|  | 826 | 12 | .0 | .0 | 83.6 |
|  | 827 | 7 | .0 | .0 | 83.6 |
|  | 828 | 12 | .0 | .0 | 83.7 |
|  | 829 | 9 | .0 | .0 | 83.7 |
|  | 830 | 13 | .0 | .0 | 83.7 |
|  | 831 | 8 | .0 | .0 | 83.8 |
|  | 832 | 9 | .0 | .0 | 83.8 |
|  | 833 | 5 | .0 | .0 | 83.8 |
|  | 834 | 12 | .0 | .0 | 83.8 |
|  | 835 | 8 | .0 | .0 | 83.9 |
|  | 836 | 9 | .0 | .0 | 83.9 |
|  | 837 | 8 | .0 | .0 | 83.9 |
|  | 838 | 9 | .0 | .0 | 84.0 |
|  | 839 | 5 | .0 | .0 | 84.0 |
|  | 840 | 9 | .0 | .0 | 84.0 |
|  | 841 | 12 | .0 | .0 | 84.0 |
|  | 842 | 16 | .1 | .1 | 84.1 |
|  | 843 | 11 | .0 | .0 | 84.1 |
|  | 844 | 10 | .0 | .0 | 84.2 |
|  | 845 | 13 | .0 | .0 | 84.2 |
|  | 846 | 10 | .0 | .0 | 84.2 |
|  | 847 | 11 | .0 | .0 | 84.3 |
|  | 848 | 11 | .0 | .0 | 84.3 |
|  | 849 | 9 | .0 | .0 | 84.3 |
|  | 850 | 10 | .0 | .0 | 84.4 |
|  | 851 | 10 | .0 | .0 | 84.4 |
|  | 852 | 15 | .0 | .0 | 84.5 |
|  | 853 | 8 | .0 | .0 | 84.5 |
|  | 854 | 10 | .0 | .0 | 84.5 |
|  | 855 | 11 | .0 | .0 | 84.6 |
|  | 856 | 6 | .0 | .0 | 84.6 |
|  | 857 | 8 | .0 | .0 | 84.6 |
|  | 858 | 7 | .0 | .0 | 84.6 |
|  | 859 | 8 | .0 | .0 | 84.6 |
|  | 860 | 7 | .0 | .0 | 84.7 |
|  | 861 | 8 | .0 | .0 | 84.7 |
|  | 862 | 8 | .0 | .0 | 84.7 |
|  | 863 | 7 | .0 | .0 | 84.7 |
|  | 864 | 10 | .0 | .0 | 84.8 |
|  | 865 | 10 | .0 | .0 | 84.8 |
|  | 866 | 6 | .0 | .0 | 84.8 |
|  | 867 | 10 | .0 | .0 | 84.9 |
|  | 868 | 10 | .0 | .0 | 84.9 |
|  | 869 | 9 | .0 | .0 | 84.9 |
|  | 870 | 10 | .0 | .0 | 85.0 |
|  | 871 | 10 | .0 | .0 | 85.0 |
|  | 872 | 9 | .0 | .0 | 85.0 |
|  | 873 | 9 | .0 | .0 | 85.0 |
|  | 874 | 10 | .0 | .0 | 85.1 |
|  | 875 | 12 | .0 | .0 | 85.1 |
|  | 876 | 8 | .0 | .0 | 85.1 |
|  | 877 | 10 | .0 | .0 | 85.2 |
|  | 878 | 10 | .0 | .0 | 85.2 |
|  | 879 | 9 | .0 | .0 | 85.2 |
|  | 880 | 11 | .0 | .0 | 85.3 |
|  | 881 | 5 | .0 | .0 | 85.3 |
|  | 882 | 9 | .0 | .0 | 85.3 |
|  | 883 | 6 | .0 | .0 | 85.3 |
|  | 884 | 5 | .0 | .0 | 85.4 |
|  | 885 | 8 | .0 | .0 | 85.4 |
|  | 886 | 12 | .0 | .0 | 85.4 |
|  | 887 | 8 | .0 | .0 | 85.5 |
|  | 888 | 12 | .0 | .0 | 85.5 |
|  | 889 | 9 | .0 | .0 | 85.5 |
|  | 890 | 7 | .0 | .0 | 85.5 |
|  | 891 | 7 | .0 | .0 | 85.6 |
|  | 892 | 9 | .0 | .0 | 85.6 |
|  | 893 | 12 | .0 | .0 | 85.6 |
|  | 894 | 7 | .0 | .0 | 85.7 |
|  | 895 | 5 | .0 | .0 | 85.7 |
|  | 896 | 6 | .0 | .0 | 85.7 |
|  | 897 | 6 | .0 | .0 | 85.7 |
|  | 898 | 8 | .0 | .0 | 85.7 |
|  | 899 | 8 | .0 | .0 | 85.8 |
|  | 900 | 6 | .0 | .0 | 85.8 |
|  | 901 | 7 | .0 | .0 | 85.8 |
|  | 902 | 5 | .0 | .0 | 85.8 |
|  | 903 | 7 | .0 | .0 | 85.8 |
|  | 904 | 11 | .0 | .0 | 85.9 |
|  | 905 | 6 | .0 | .0 | 85.9 |
|  | 906 | 12 | .0 | .0 | 85.9 |
|  | 907 | 10 | .0 | .0 | 86.0 |
|  | 908 | 4 | .0 | .0 | 86.0 |
|  | 909 | 14 | .0 | .0 | 86.0 |
|  | 910 | 12 | .0 | .0 | 86.1 |
|  | 911 | 7 | .0 | .0 | 86.1 |
|  | 912 | 7 | .0 | .0 | 86.1 |
|  | 913 | 8 | .0 | .0 | 86.1 |
|  | 914 | 5 | .0 | .0 | 86.2 |
|  | 915 | 8 | .0 | .0 | 86.2 |
|  | 916 | 10 | .0 | .0 | 86.2 |
|  | 917 | 6 | .0 | .0 | 86.2 |
|  | 918 | 3 | .0 | .0 | 86.3 |
|  | 919 | 11 | .0 | .0 | 86.3 |
|  | 920 | 7 | .0 | .0 | 86.3 |
|  | 921 | 10 | .0 | .0 | 86.3 |
|  | 922 | 9 | .0 | .0 | 86.4 |
|  | 923 | 4 | .0 | .0 | 86.4 |
|  | 924 | 7 | .0 | .0 | 86.4 |
|  | 925 | 7 | .0 | .0 | 86.4 |
|  | 926 | 8 | .0 | .0 | 86.5 |
|  | 927 | 14 | .0 | .0 | 86.5 |
|  | 928 | 9 | .0 | .0 | 86.5 |
|  | 929 | 10 | .0 | .0 | 86.6 |
|  | 930 | 8 | .0 | .0 | 86.6 |
|  | 931 | 7 | .0 | .0 | 86.6 |
|  | 932 | 10 | .0 | .0 | 86.6 |
|  | 933 | 9 | .0 | .0 | 86.7 |
|  | 934 | 6 | .0 | .0 | 86.7 |
|  | 935 | 5 | .0 | .0 | 86.7 |
|  | 936 | 12 | .0 | .0 | 86.8 |
|  | 937 | 6 | .0 | .0 | 86.8 |
|  | 938 | 10 | .0 | .0 | 86.8 |
|  | 939 | 11 | .0 | .0 | 86.8 |
|  | 940 | 11 | .0 | .0 | 86.9 |
|  | 941 | 8 | .0 | .0 | 86.9 |
|  | 942 | 9 | .0 | .0 | 86.9 |
|  | 943 | 8 | .0 | .0 | 87.0 |
|  | 944 | 13 | .0 | .0 | 87.0 |
|  | 945 | 11 | .0 | .0 | 87.0 |
|  | 946 | 5 | .0 | .0 | 87.1 |
|  | 947 | 9 | .0 | .0 | 87.1 |
|  | 948 | 6 | .0 | .0 | 87.1 |
|  | 949 | 9 | .0 | .0 | 87.1 |
|  | 950 | 4 | .0 | .0 | 87.1 |
|  | 951 | 6 | .0 | .0 | 87.2 |
|  | 952 | 6 | .0 | .0 | 87.2 |
|  | 953 | 5 | .0 | .0 | 87.2 |
|  | 954 | 6 | .0 | .0 | 87.2 |
|  | 955 | 4 | .0 | .0 | 87.2 |
|  | 956 | 4 | .0 | .0 | 87.2 |
|  | 957 | 5 | .0 | .0 | 87.3 |
|  | 958 | 4 | .0 | .0 | 87.3 |
|  | 959 | 4 | .0 | .0 | 87.3 |
|  | 960 | 5 | .0 | .0 | 87.3 |
|  | 961 | 10 | .0 | .0 | 87.3 |
|  | 962 | 4 | .0 | .0 | 87.3 |
|  | 963 | 8 | .0 | .0 | 87.4 |
|  | 964 | 6 | .0 | .0 | 87.4 |
|  | 965 | 9 | .0 | .0 | 87.4 |
|  | 966 | 8 | .0 | .0 | 87.5 |
|  | 967 | 6 | .0 | .0 | 87.5 |
|  | 968 | 3 | .0 | .0 | 87.5 |
|  | 969 | 4 | .0 | .0 | 87.5 |
|  | 970 | 7 | .0 | .0 | 87.5 |
|  | 971 | 8 | .0 | .0 | 87.5 |
|  | 972 | 6 | .0 | .0 | 87.6 |
|  | 973 | 7 | .0 | .0 | 87.6 |
|  | 974 | 4 | .0 | .0 | 87.6 |
|  | 975 | 6 | .0 | .0 | 87.6 |
|  | 976 | 5 | .0 | .0 | 87.6 |
|  | 977 | 9 | .0 | .0 | 87.7 |
|  | 978 | 6 | .0 | .0 | 87.7 |
|  | 979 | 5 | .0 | .0 | 87.7 |
|  | 980 | 10 | .0 | .0 | 87.7 |
|  | 981 | 5 | .0 | .0 | 87.7 |
|  | 982 | 6 | .0 | .0 | 87.8 |
|  | 983 | 5 | .0 | .0 | 87.8 |
|  | 984 | 11 | .0 | .0 | 87.8 |
|  | 985 | 6 | .0 | .0 | 87.8 |
|  | 986 | 4 | .0 | .0 | 87.9 |
|  | 987 | 13 | .0 | .0 | 87.9 |
|  | 988 | 5 | .0 | .0 | 87.9 |
|  | 989 | 5 | .0 | .0 | 87.9 |
|  | 990 | 8 | .0 | .0 | 88.0 |
|  | 991 | 8 | .0 | .0 | 88.0 |
|  | 992 | 3 | .0 | .0 | 88.0 |
|  | 993 | 5 | .0 | .0 | 88.0 |
|  | 994 | 6 | .0 | .0 | 88.0 |
|  | 995 | 11 | .0 | .0 | 88.1 |
|  | 996 | 5 | .0 | .0 | 88.1 |
|  | 997 | 7 | .0 | .0 | 88.1 |
|  | 998 | 5 | .0 | .0 | 88.1 |
|  | 999 | 2 | .0 | .0 | 88.1 |
|  | 1000 | 4 | .0 | .0 | 88.1 |
|  | 1001 | 2 | .0 | .0 | 88.1 |
|  | 1002 | 5 | .0 | .0 | 88.2 |
|  | 1003 | 7 | .0 | .0 | 88.2 |
|  | 1004 | 9 | .0 | .0 | 88.2 |
|  | 1005 | 7 | .0 | .0 | 88.2 |
|  | 1006 | 5 | .0 | .0 | 88.3 |
|  | 1007 | 5 | .0 | .0 | 88.3 |
|  | 1008 | 3 | .0 | .0 | 88.3 |
|  | 1009 | 5 | .0 | .0 | 88.3 |
|  | 1010 | 1 | .0 | .0 | 88.3 |
|  | 1011 | 5 | .0 | .0 | 88.3 |
|  | 1012 | 11 | .0 | .0 | 88.4 |
|  | 1013 | 5 | .0 | .0 | 88.4 |
|  | 1014 | 4 | .0 | .0 | 88.4 |
|  | 1015 | 9 | .0 | .0 | 88.4 |
|  | 1016 | 6 | .0 | .0 | 88.4 |
|  | 1017 | 4 | .0 | .0 | 88.4 |
|  | 1018 | 2 | .0 | .0 | 88.4 |
|  | 1019 | 4 | .0 | .0 | 88.5 |
|  | 1020 | 2 | .0 | .0 | 88.5 |
|  | 1021 | 2 | .0 | .0 | 88.5 |
|  | 1022 | 5 | .0 | .0 | 88.5 |
|  | 1023 | 10 | .0 | .0 | 88.5 |
|  | 1024 | 4 | .0 | .0 | 88.5 |
|  | 1025 | 2 | .0 | .0 | 88.5 |
|  | 1026 | 5 | .0 | .0 | 88.6 |
|  | 1027 | 9 | .0 | .0 | 88.6 |
|  | 1028 | 6 | .0 | .0 | 88.6 |
|  | 1029 | 3 | .0 | .0 | 88.6 |
|  | 1030 | 4 | .0 | .0 | 88.6 |
|  | 1031 | 1 | .0 | .0 | 88.6 |
|  | 1032 | 3 | .0 | .0 | 88.6 |
|  | 1033 | 5 | .0 | .0 | 88.7 |
|  | 1034 | 5 | .0 | .0 | 88.7 |
|  | 1035 | 4 | .0 | .0 | 88.7 |
|  | 1036 | 6 | .0 | .0 | 88.7 |
|  | 1037 | 5 | .0 | .0 | 88.7 |
|  | 1038 | 7 | .0 | .0 | 88.8 |
|  | 1039 | 6 | .0 | .0 | 88.8 |
|  | 1040 | 7 | .0 | .0 | 88.8 |
|  | 1041 | 6 | .0 | .0 | 88.8 |
|  | 1042 | 9 | .0 | .0 | 88.8 |
|  | 1043 | 7 | .0 | .0 | 88.9 |
|  | 1044 | 9 | .0 | .0 | 88.9 |
|  | 1045 | 7 | .0 | .0 | 88.9 |
|  | 1046 | 5 | .0 | .0 | 88.9 |
|  | 1047 | 5 | .0 | .0 | 89.0 |
|  | 1048 | 4 | .0 | .0 | 89.0 |
|  | 1049 | 8 | .0 | .0 | 89.0 |
|  | 1050 | 2 | .0 | .0 | 89.0 |
|  | 1051 | 2 | .0 | .0 | 89.0 |
|  | 1052 | 6 | .0 | .0 | 89.0 |
|  | 1053 | 3 | .0 | .0 | 89.0 |
|  | 1054 | 7 | .0 | .0 | 89.1 |
|  | 1055 | 5 | .0 | .0 | 89.1 |
|  | 1056 | 8 | .0 | .0 | 89.1 |
|  | 1057 | 8 | .0 | .0 | 89.1 |
|  | 1058 | 5 | .0 | .0 | 89.1 |
|  | 1059 | 6 | .0 | .0 | 89.2 |
|  | 1060 | 2 | .0 | .0 | 89.2 |
|  | 1061 | 11 | .0 | .0 | 89.2 |
|  | 1062 | 6 | .0 | .0 | 89.2 |
|  | 1063 | 4 | .0 | .0 | 89.2 |
|  | 1064 | 5 | .0 | .0 | 89.3 |
|  | 1065 | 10 | .0 | .0 | 89.3 |
|  | 1066 | 5 | .0 | .0 | 89.3 |
|  | 1067 | 4 | .0 | .0 | 89.3 |
|  | 1068 | 7 | .0 | .0 | 89.3 |
|  | 1069 | 5 | .0 | .0 | 89.4 |
|  | 1070 | 1 | .0 | .0 | 89.4 |
|  | 1071 | 3 | .0 | .0 | 89.4 |
|  | 1073 | 4 | .0 | .0 | 89.4 |
|  | 1074 | 6 | .0 | .0 | 89.4 |
|  | 1075 | 7 | .0 | .0 | 89.4 |
|  | 1076 | 3 | .0 | .0 | 89.4 |
|  | 1077 | 13 | .0 | .0 | 89.5 |
|  | 1078 | 11 | .0 | .0 | 89.5 |
|  | 1079 | 7 | .0 | .0 | 89.5 |
|  | 1080 | 6 | .0 | .0 | 89.6 |
|  | 1081 | 5 | .0 | .0 | 89.6 |
|  | 1082 | 3 | .0 | .0 | 89.6 |
|  | 1083 | 4 | .0 | .0 | 89.6 |
|  | 1084 | 3 | .0 | .0 | 89.6 |
|  | 1085 | 5 | .0 | .0 | 89.6 |
|  | 1086 | 2 | .0 | .0 | 89.6 |
|  | 1087 | 8 | .0 | .0 | 89.7 |
|  | 1088 | 7 | .0 | .0 | 89.7 |
|  | 1089 | 5 | .0 | .0 | 89.7 |
|  | 1090 | 5 | .0 | .0 | 89.7 |
|  | 1091 | 3 | .0 | .0 | 89.7 |
|  | 1092 | 2 | .0 | .0 | 89.7 |
|  | 1093 | 2 | .0 | .0 | 89.7 |
|  | 1094 | 3 | .0 | .0 | 89.7 |
|  | 1095 | 6 | .0 | .0 | 89.8 |
|  | 1096 | 6 | .0 | .0 | 89.8 |
|  | 1097 | 7 | .0 | .0 | 89.8 |
|  | 1098 | 1 | .0 | .0 | 89.8 |
|  | 1099 | 5 | .0 | .0 | 89.8 |
|  | 1100 | 3 | .0 | .0 | 89.8 |
|  | 1101 | 3 | .0 | .0 | 89.8 |
|  | 1102 | 3 | .0 | .0 | 89.9 |
|  | 1103 | 3 | .0 | .0 | 89.9 |
|  | 1104 | 2 | .0 | .0 | 89.9 |
|  | 1105 | 5 | .0 | .0 | 89.9 |
|  | 1106 | 4 | .0 | .0 | 89.9 |
|  | 1107 | 5 | .0 | .0 | 89.9 |
|  | 1108 | 5 | .0 | .0 | 89.9 |
|  | 1109 | 6 | .0 | .0 | 89.9 |
|  | 1110 | 6 | .0 | .0 | 90.0 |
|  | 1111 | 4 | .0 | .0 | 90.0 |
|  | 1112 | 5 | .0 | .0 | 90.0 |
|  | 1113 | 4 | .0 | .0 | 90.0 |
|  | 1114 | 5 | .0 | .0 | 90.0 |
|  | 1115 | 5 | .0 | .0 | 90.0 |
|  | 1116 | 8 | .0 | .0 | 90.1 |
|  | 1117 | 5 | .0 | .0 | 90.1 |
|  | 1118 | 7 | .0 | .0 | 90.1 |
|  | 1119 | 2 | .0 | .0 | 90.1 |
|  | 1120 | 4 | .0 | .0 | 90.1 |
|  | 1121 | 6 | .0 | .0 | 90.1 |
|  | 1122 | 6 | .0 | .0 | 90.2 |
|  | 1123 | 5 | .0 | .0 | 90.2 |
|  | 1124 | 6 | .0 | .0 | 90.2 |
|  | 1125 | 5 | .0 | .0 | 90.2 |
|  | 1126 | 4 | .0 | .0 | 90.2 |
|  | 1127 | 6 | .0 | .0 | 90.3 |
|  | 1128 | 4 | .0 | .0 | 90.3 |
|  | 1129 | 3 | .0 | .0 | 90.3 |
|  | 1130 | 5 | .0 | .0 | 90.3 |
|  | 1131 | 3 | .0 | .0 | 90.3 |
|  | 1132 | 1 | .0 | .0 | 90.3 |
|  | 1133 | 8 | .0 | .0 | 90.3 |
|  | 1134 | 4 | .0 | .0 | 90.3 |
|  | 1135 | 4 | .0 | .0 | 90.4 |
|  | 1136 | 4 | .0 | .0 | 90.4 |
|  | 1137 | 7 | .0 | .0 | 90.4 |
|  | 1138 | 9 | .0 | .0 | 90.4 |
|  | 1139 | 7 | .0 | .0 | 90.4 |
|  | 1140 | 4 | .0 | .0 | 90.5 |
|  | 1141 | 7 | .0 | .0 | 90.5 |
|  | 1142 | 7 | .0 | .0 | 90.5 |
|  | 1143 | 8 | .0 | .0 | 90.5 |
|  | 1144 | 7 | .0 | .0 | 90.6 |
|  | 1145 | 2 | .0 | .0 | 90.6 |
|  | 1146 | 3 | .0 | .0 | 90.6 |
|  | 1147 | 1 | .0 | .0 | 90.6 |
|  | 1148 | 2 | .0 | .0 | 90.6 |
|  | 1150 | 2 | .0 | .0 | 90.6 |
|  | 1151 | 3 | .0 | .0 | 90.6 |
|  | 1152 | 4 | .0 | .0 | 90.6 |
|  | 1153 | 4 | .0 | .0 | 90.6 |
|  | 1154 | 1 | .0 | .0 | 90.6 |
|  | 1155 | 4 | .0 | .0 | 90.6 |
|  | 1156 | 4 | .0 | .0 | 90.7 |
|  | 1157 | 7 | .0 | .0 | 90.7 |
|  | 1158 | 4 | .0 | .0 | 90.7 |
|  | 1159 | 6 | .0 | .0 | 90.7 |
|  | 1160 | 6 | .0 | .0 | 90.7 |
|  | 1161 | 7 | .0 | .0 | 90.8 |
|  | 1162 | 10 | .0 | .0 | 90.8 |
|  | 1163 | 5 | .0 | .0 | 90.8 |
|  | 1164 | 8 | .0 | .0 | 90.8 |
|  | 1165 | 8 | .0 | .0 | 90.9 |
|  | 1166 | 2 | .0 | .0 | 90.9 |
|  | 1167 | 3 | .0 | .0 | 90.9 |
|  | 1168 | 4 | .0 | .0 | 90.9 |
|  | 1169 | 6 | .0 | .0 | 90.9 |
|  | 1170 | 5 | .0 | .0 | 90.9 |
|  | 1171 | 6 | .0 | .0 | 90.9 |
|  | 1172 | 5 | .0 | .0 | 91.0 |
|  | 1173 | 2 | .0 | .0 | 91.0 |
|  | 1174 | 4 | .0 | .0 | 91.0 |
|  | 1175 | 1 | .0 | .0 | 91.0 |
|  | 1176 | 9 | .0 | .0 | 91.0 |
|  | 1177 | 7 | .0 | .0 | 91.0 |
|  | 1178 | 5 | .0 | .0 | 91.0 |
|  | 1179 | 5 | .0 | .0 | 91.1 |
|  | 1180 | 9 | .0 | .0 | 91.1 |
|  | 1181 | 9 | .0 | .0 | 91.1 |
|  | 1182 | 6 | .0 | .0 | 91.1 |
|  | 1183 | 3 | .0 | .0 | 91.1 |
|  | 1184 | 3 | .0 | .0 | 91.2 |
|  | 1185 | 6 | .0 | .0 | 91.2 |
|  | 1186 | 6 | .0 | .0 | 91.2 |
|  | 1187 | 4 | .0 | .0 | 91.2 |
|  | 1188 | 6 | .0 | .0 | 91.2 |
|  | 1189 | 3 | .0 | .0 | 91.2 |
|  | 1190 | 10 | .0 | .0 | 91.3 |
|  | 1191 | 4 | .0 | .0 | 91.3 |
|  | 1192 | 9 | .0 | .0 | 91.3 |
|  | 1193 | 4 | .0 | .0 | 91.3 |
|  | 1194 | 2 | .0 | .0 | 91.3 |
|  | 1195 | 6 | .0 | .0 | 91.4 |
|  | 1196 | 5 | .0 | .0 | 91.4 |
|  | 1197 | 6 | .0 | .0 | 91.4 |
|  | 1198 | 5 | .0 | .0 | 91.4 |
|  | 1199 | 6 | .0 | .0 | 91.4 |
|  | 1200 | 5 | .0 | .0 | 91.4 |
|  | 1201 | 5 | .0 | .0 | 91.5 |
|  | 1202 | 9 | .0 | .0 | 91.5 |
|  | 1203 | 10 | .0 | .0 | 91.5 |
|  | 1204 | 4 | .0 | .0 | 91.5 |
|  | 1205 | 2 | .0 | .0 | 91.5 |
|  | 1206 | 3 | .0 | .0 | 91.6 |
|  | 1207 | 3 | .0 | .0 | 91.6 |
|  | 1208 | 8 | .0 | .0 | 91.6 |
|  | 1209 | 4 | .0 | .0 | 91.6 |
|  | 1210 | 6 | .0 | .0 | 91.6 |
|  | 1211 | 2 | .0 | .0 | 91.6 |
|  | 1212 | 3 | .0 | .0 | 91.6 |
|  | 1213 | 5 | .0 | .0 | 91.7 |
|  | 1214 | 1 | .0 | .0 | 91.7 |
|  | 1215 | 3 | .0 | .0 | 91.7 |
|  | 1216 | 1 | .0 | .0 | 91.7 |
|  | 1217 | 5 | .0 | .0 | 91.7 |
|  | 1218 | 5 | .0 | .0 | 91.7 |
|  | 1219 | 2 | .0 | .0 | 91.7 |
|  | 1220 | 6 | .0 | .0 | 91.7 |
|  | 1221 | 4 | .0 | .0 | 91.7 |
|  | 1222 | 2 | .0 | .0 | 91.7 |
|  | 1223 | 10 | .0 | .0 | 91.8 |
|  | 1224 | 4 | .0 | .0 | 91.8 |
|  | 1225 | 1 | .0 | .0 | 91.8 |
|  | 1226 | 4 | .0 | .0 | 91.8 |
|  | 1227 | 5 | .0 | .0 | 91.8 |
|  | 1228 | 6 | .0 | .0 | 91.8 |
|  | 1229 | 3 | .0 | .0 | 91.9 |
|  | 1230 | 5 | .0 | .0 | 91.9 |
|  | 1231 | 4 | .0 | .0 | 91.9 |
|  | 1232 | 3 | .0 | .0 | 91.9 |
|  | 1233 | 1 | .0 | .0 | 91.9 |
|  | 1234 | 4 | .0 | .0 | 91.9 |
|  | 1235 | 4 | .0 | .0 | 91.9 |
|  | 1236 | 6 | .0 | .0 | 91.9 |
|  | 1237 | 3 | .0 | .0 | 92.0 |
|  | 1238 | 2 | .0 | .0 | 92.0 |
|  | 1239 | 1 | .0 | .0 | 92.0 |
|  | 1240 | 4 | .0 | .0 | 92.0 |
|  | 1241 | 1 | .0 | .0 | 92.0 |
|  | 1242 | 5 | .0 | .0 | 92.0 |
|  | 1243 | 4 | .0 | .0 | 92.0 |
|  | 1244 | 2 | .0 | .0 | 92.0 |
|  | 1245 | 4 | .0 | .0 | 92.0 |
|  | 1246 | 5 | .0 | .0 | 92.0 |
|  | 1247 | 1 | .0 | .0 | 92.0 |
|  | 1248 | 3 | .0 | .0 | 92.1 |
|  | 1249 | 5 | .0 | .0 | 92.1 |
|  | 1250 | 2 | .0 | .0 | 92.1 |
|  | 1251 | 2 | .0 | .0 | 92.1 |
|  | 1252 | 5 | .0 | .0 | 92.1 |
|  | 1253 | 5 | .0 | .0 | 92.1 |
|  | 1254 | 4 | .0 | .0 | 92.1 |
|  | 1255 | 1 | .0 | .0 | 92.1 |
|  | 1256 | 4 | .0 | .0 | 92.2 |
|  | 1257 | 3 | .0 | .0 | 92.2 |
|  | 1258 | 3 | .0 | .0 | 92.2 |
|  | 1259 | 8 | .0 | .0 | 92.2 |
|  | 1260 | 3 | .0 | .0 | 92.2 |
|  | 1261 | 5 | .0 | .0 | 92.2 |
|  | 1262 | 2 | .0 | .0 | 92.2 |
|  | 1263 | 2 | .0 | .0 | 92.2 |
|  | 1264 | 5 | .0 | .0 | 92.3 |
|  | 1265 | 6 | .0 | .0 | 92.3 |
|  | 1266 | 3 | .0 | .0 | 92.3 |
|  | 1267 | 3 | .0 | .0 | 92.3 |
|  | 1268 | 1 | .0 | .0 | 92.3 |
|  | 1269 | 4 | .0 | .0 | 92.3 |
|  | 1270 | 7 | .0 | .0 | 92.3 |
|  | 1271 | 3 | .0 | .0 | 92.3 |
|  | 1272 | 3 | .0 | .0 | 92.4 |
|  | 1273 | 5 | .0 | .0 | 92.4 |
|  | 1274 | 6 | .0 | .0 | 92.4 |
|  | 1275 | 1 | .0 | .0 | 92.4 |
|  | 1276 | 5 | .0 | .0 | 92.4 |
|  | 1277 | 3 | .0 | .0 | 92.4 |
|  | 1278 | 4 | .0 | .0 | 92.4 |
|  | 1279 | 7 | .0 | .0 | 92.5 |
|  | 1280 | 8 | .0 | .0 | 92.5 |
|  | 1281 | 3 | .0 | .0 | 92.5 |
|  | 1282 | 3 | .0 | .0 | 92.5 |
|  | 1283 | 8 | .0 | .0 | 92.5 |
|  | 1284 | 5 | .0 | .0 | 92.5 |
|  | 1285 | 2 | .0 | .0 | 92.5 |
|  | 1286 | 7 | .0 | .0 | 92.6 |
|  | 1287 | 5 | .0 | .0 | 92.6 |
|  | 1288 | 4 | .0 | .0 | 92.6 |
|  | 1289 | 2 | .0 | .0 | 92.6 |
|  | 1291 | 5 | .0 | .0 | 92.6 |
|  | 1292 | 3 | .0 | .0 | 92.6 |
|  | 1293 | 2 | .0 | .0 | 92.6 |
|  | 1294 | 7 | .0 | .0 | 92.7 |
|  | 1295 | 4 | .0 | .0 | 92.7 |
|  | 1296 | 5 | .0 | .0 | 92.7 |
|  | 1297 | 11 | .0 | .0 | 92.7 |
|  | 1298 | 1 | .0 | .0 | 92.7 |
|  | 1299 | 1 | .0 | .0 | 92.7 |
|  | 1300 | 4 | .0 | .0 | 92.7 |
|  | 1301 | 5 | .0 | .0 | 92.8 |
|  | 1302 | 3 | .0 | .0 | 92.8 |
|  | 1303 | 2 | .0 | .0 | 92.8 |
|  | 1304 | 6 | .0 | .0 | 92.8 |
|  | 1305 | 5 | .0 | .0 | 92.8 |
|  | 1306 | 6 | .0 | .0 | 92.8 |
|  | 1307 | 7 | .0 | .0 | 92.9 |
|  | 1308 | 3 | .0 | .0 | 92.9 |
|  | 1309 | 1 | .0 | .0 | 92.9 |
|  | 1310 | 2 | .0 | .0 | 92.9 |
|  | 1311 | 5 | .0 | .0 | 92.9 |
|  | 1312 | 3 | .0 | .0 | 92.9 |
|  | 1313 | 1 | .0 | .0 | 92.9 |
|  | 1314 | 5 | .0 | .0 | 92.9 |
|  | 1315 | 1 | .0 | .0 | 92.9 |
|  | 1316 | 4 | .0 | .0 | 92.9 |
|  | 1317 | 1 | .0 | .0 | 92.9 |
|  | 1318 | 3 | .0 | .0 | 93.0 |
|  | 1319 | 6 | .0 | .0 | 93.0 |
|  | 1320 | 2 | .0 | .0 | 93.0 |
|  | 1321 | 5 | .0 | .0 | 93.0 |
|  | 1322 | 4 | .0 | .0 | 93.0 |
|  | 1323 | 2 | .0 | .0 | 93.0 |
|  | 1324 | 4 | .0 | .0 | 93.0 |
|  | 1325 | 6 | .0 | .0 | 93.0 |
|  | 1326 | 3 | .0 | .0 | 93.1 |
|  | 1327 | 2 | .0 | .0 | 93.1 |
|  | 1328 | 7 | .0 | .0 | 93.1 |
|  | 1329 | 3 | .0 | .0 | 93.1 |
|  | 1330 | 4 | .0 | .0 | 93.1 |
|  | 1331 | 4 | .0 | .0 | 93.1 |
|  | 1332 | 7 | .0 | .0 | 93.1 |
|  | 1333 | 1 | .0 | .0 | 93.1 |
|  | 1334 | 4 | .0 | .0 | 93.2 |
|  | 1335 | 3 | .0 | .0 | 93.2 |
|  | 1336 | 5 | .0 | .0 | 93.2 |
|  | 1337 | 2 | .0 | .0 | 93.2 |
|  | 1338 | 4 | .0 | .0 | 93.2 |
|  | 1339 | 2 | .0 | .0 | 93.2 |
|  | 1340 | 8 | .0 | .0 | 93.2 |
|  | 1341 | 1 | .0 | .0 | 93.2 |
|  | 1342 | 1 | .0 | .0 | 93.2 |
|  | 1343 | 2 | .0 | .0 | 93.3 |
|  | 1345 | 4 | .0 | .0 | 93.3 |
|  | 1346 | 2 | .0 | .0 | 93.3 |
|  | 1347 | 2 | .0 | .0 | 93.3 |
|  | 1348 | 2 | .0 | .0 | 93.3 |
|  | 1350 | 5 | .0 | .0 | 93.3 |
|  | 1351 | 3 | .0 | .0 | 93.3 |
|  | 1352 | 3 | .0 | .0 | 93.3 |
|  | 1353 | 1 | .0 | .0 | 93.3 |
|  | 1354 | 3 | .0 | .0 | 93.3 |
|  | 1355 | 1 | .0 | .0 | 93.3 |
|  | 1356 | 5 | .0 | .0 | 93.4 |
|  | 1358 | 7 | .0 | .0 | 93.4 |
|  | 1359 | 1 | .0 | .0 | 93.4 |
|  | 1360 | 6 | .0 | .0 | 93.4 |
|  | 1361 | 3 | .0 | .0 | 93.4 |
|  | 1362 | 7 | .0 | .0 | 93.4 |
|  | 1363 | 4 | .0 | .0 | 93.4 |
|  | 1364 | 3 | .0 | .0 | 93.5 |
|  | 1365 | 7 | .0 | .0 | 93.5 |
|  | 1366 | 5 | .0 | .0 | 93.5 |
|  | 1367 | 8 | .0 | .0 | 93.5 |
|  | 1368 | 5 | .0 | .0 | 93.5 |
|  | 1369 | 4 | .0 | .0 | 93.6 |
|  | 1370 | 1 | .0 | .0 | 93.6 |
|  | 1371 | 7 | .0 | .0 | 93.6 |
|  | 1372 | 2 | .0 | .0 | 93.6 |
|  | 1373 | 5 | .0 | .0 | 93.6 |
|  | 1374 | 2 | .0 | .0 | 93.6 |
|  | 1375 | 6 | .0 | .0 | 93.6 |
|  | 1376 | 5 | .0 | .0 | 93.6 |
|  | 1377 | 3 | .0 | .0 | 93.7 |
|  | 1378 | 1 | .0 | .0 | 93.7 |
|  | 1379 | 2 | .0 | .0 | 93.7 |
|  | 1380 | 3 | .0 | .0 | 93.7 |
|  | 1381 | 2 | .0 | .0 | 93.7 |
|  | 1382 | 5 | .0 | .0 | 93.7 |
|  | 1383 | 1 | .0 | .0 | 93.7 |
|  | 1384 | 1 | .0 | .0 | 93.7 |
|  | 1385 | 3 | .0 | .0 | 93.7 |
|  | 1386 | 3 | .0 | .0 | 93.7 |
|  | 1387 | 3 | .0 | .0 | 93.7 |
|  | 1388 | 4 | .0 | .0 | 93.7 |
|  | 1389 | 3 | .0 | .0 | 93.8 |
|  | 1390 | 1 | .0 | .0 | 93.8 |
|  | 1391 | 6 | .0 | .0 | 93.8 |
|  | 1392 | 5 | .0 | .0 | 93.8 |
|  | 1393 | 4 | .0 | .0 | 93.8 |
|  | 1394 | 6 | .0 | .0 | 93.8 |
|  | 1395 | 4 | .0 | .0 | 93.8 |
|  | 1396 | 4 | .0 | .0 | 93.9 |
|  | 1397 | 4 | .0 | .0 | 93.9 |
|  | 1398 | 7 | .0 | .0 | 93.9 |
|  | 1399 | 5 | .0 | .0 | 93.9 |
|  | 1400 | 1 | .0 | .0 | 93.9 |
|  | 1401 | 5 | .0 | .0 | 93.9 |
|  | 1402 | 2 | .0 | .0 | 93.9 |
|  | 1403 | 1 | .0 | .0 | 93.9 |
|  | 1404 | 5 | .0 | .0 | 94.0 |
|  | 1405 | 6 | .0 | .0 | 94.0 |
|  | 1406 | 5 | .0 | .0 | 94.0 |
|  | 1407 | 5 | .0 | .0 | 94.0 |
|  | 1408 | 5 | .0 | .0 | 94.0 |
|  | 1409 | 3 | .0 | .0 | 94.0 |
|  | 1410 | 2 | .0 | .0 | 94.0 |
|  | 1411 | 2 | .0 | .0 | 94.0 |
|  | 1412 | 5 | .0 | .0 | 94.1 |
|  | 1413 | 2 | .0 | .0 | 94.1 |
|  | 1414 | 6 | .0 | .0 | 94.1 |
|  | 1415 | 6 | .0 | .0 | 94.1 |
|  | 1416 | 1 | .0 | .0 | 94.1 |
|  | 1417 | 4 | .0 | .0 | 94.1 |
|  | 1418 | 4 | .0 | .0 | 94.1 |
|  | 1419 | 3 | .0 | .0 | 94.1 |
|  | 1420 | 4 | .0 | .0 | 94.2 |
|  | 1421 | 5 | .0 | .0 | 94.2 |
|  | 1422 | 2 | .0 | .0 | 94.2 |
|  | 1423 | 6 | .0 | .0 | 94.2 |
|  | 1424 | 3 | .0 | .0 | 94.2 |
|  | 1425 | 1 | .0 | .0 | 94.2 |
|  | 1426 | 2 | .0 | .0 | 94.2 |
|  | 1427 | 4 | .0 | .0 | 94.2 |
|  | 1428 | 5 | .0 | .0 | 94.2 |
|  | 1429 | 2 | .0 | .0 | 94.3 |
|  | 1430 | 3 | .0 | .0 | 94.3 |
|  | 1431 | 3 | .0 | .0 | 94.3 |
|  | 1432 | 3 | .0 | .0 | 94.3 |
|  | 1433 | 1 | .0 | .0 | 94.3 |
|  | 1434 | 6 | .0 | .0 | 94.3 |
|  | 1435 | 4 | .0 | .0 | 94.3 |
|  | 1436 | 2 | .0 | .0 | 94.3 |
|  | 1437 | 3 | .0 | .0 | 94.3 |
|  | 1438 | 3 | .0 | .0 | 94.3 |
|  | 1439 | 4 | .0 | .0 | 94.4 |
|  | 1440 | 4 | .0 | .0 | 94.4 |
|  | 1441 | 4 | .0 | .0 | 94.4 |
|  | 1442 | 5 | .0 | .0 | 94.4 |
|  | 1443 | 2 | .0 | .0 | 94.4 |
|  | 1444 | 6 | .0 | .0 | 94.4 |
|  | 1445 | 5 | .0 | .0 | 94.4 |
|  | 1446 | 2 | .0 | .0 | 94.5 |
|  | 1447 | 2 | .0 | .0 | 94.5 |
|  | 1448 | 2 | .0 | .0 | 94.5 |
|  | 1449 | 3 | .0 | .0 | 94.5 |
|  | 1450 | 2 | .0 | .0 | 94.5 |
|  | 1451 | 3 | .0 | .0 | 94.5 |
|  | 1452 | 3 | .0 | .0 | 94.5 |
|  | 1453 | 2 | .0 | .0 | 94.5 |
|  | 1454 | 4 | .0 | .0 | 94.5 |
|  | 1455 | 2 | .0 | .0 | 94.5 |
|  | 1456 | 2 | .0 | .0 | 94.5 |
|  | 1457 | 3 | .0 | .0 | 94.5 |
|  | 1458 | 1 | .0 | .0 | 94.5 |
|  | 1459 | 4 | .0 | .0 | 94.6 |
|  | 1460 | 5 | .0 | .0 | 94.6 |
|  | 1461 | 6 | .0 | .0 | 94.6 |
|  | 1462 | 6 | .0 | .0 | 94.6 |
|  | 1463 | 2 | .0 | .0 | 94.6 |
|  | 1464 | 1 | .0 | .0 | 94.6 |
|  | 1465 | 2 | .0 | .0 | 94.6 |
|  | 1466 | 1 | .0 | .0 | 94.6 |
|  | 1467 | 2 | .0 | .0 | 94.6 |
|  | 1468 | 2 | .0 | .0 | 94.6 |
|  | 1469 | 2 | .0 | .0 | 94.7 |
|  | 1471 | 2 | .0 | .0 | 94.7 |
|  | 1472 | 1 | .0 | .0 | 94.7 |
|  | 1473 | 3 | .0 | .0 | 94.7 |
|  | 1475 | 1 | .0 | .0 | 94.7 |
|  | 1476 | 4 | .0 | .0 | 94.7 |
|  | 1477 | 1 | .0 | .0 | 94.7 |
|  | 1478 | 2 | .0 | .0 | 94.7 |
|  | 1479 | 2 | .0 | .0 | 94.7 |
|  | 1480 | 3 | .0 | .0 | 94.7 |
|  | 1481 | 1 | .0 | .0 | 94.7 |
|  | 1482 | 3 | .0 | .0 | 94.7 |
|  | 1483 | 2 | .0 | .0 | 94.7 |
|  | 1484 | 2 | .0 | .0 | 94.7 |
|  | 1485 | 1 | .0 | .0 | 94.7 |
|  | 1486 | 4 | .0 | .0 | 94.8 |
|  | 1487 | 3 | .0 | .0 | 94.8 |
|  | 1488 | 3 | .0 | .0 | 94.8 |
|  | 1489 | 7 | .0 | .0 | 94.8 |
|  | 1490 | 4 | .0 | .0 | 94.8 |
|  | 1491 | 2 | .0 | .0 | 94.8 |
|  | 1492 | 3 | .0 | .0 | 94.8 |
|  | 1493 | 2 | .0 | .0 | 94.8 |
|  | 1494 | 2 | .0 | .0 | 94.8 |
|  | 1495 | 2 | .0 | .0 | 94.9 |
|  | 1496 | 3 | .0 | .0 | 94.9 |
|  | 1497 | 5 | .0 | .0 | 94.9 |
|  | 1498 | 2 | .0 | .0 | 94.9 |
|  | 1499 | 4 | .0 | .0 | 94.9 |
|  | 1500 | 2 | .0 | .0 | 94.9 |
|  | 1501 | 2 | .0 | .0 | 94.9 |
|  | 1502 | 5 | .0 | .0 | 94.9 |
|  | 1503 | 2 | .0 | .0 | 94.9 |
|  | 1504 | 2 | .0 | .0 | 94.9 |
|  | 1505 | 2 | .0 | .0 | 94.9 |
|  | 1506 | 4 | .0 | .0 | 95.0 |
|  | 1507 | 1 | .0 | .0 | 95.0 |
|  | 1508 | 2 | .0 | .0 | 95.0 |
|  | 1509 | 4 | .0 | .0 | 95.0 |
|  | 1513 | 3 | .0 | .0 | 95.0 |
|  | 1514 | 2 | .0 | .0 | 95.0 |
|  | 1515 | 3 | .0 | .0 | 95.0 |
|  | 1516 | 3 | .0 | .0 | 95.0 |
|  | 1517 | 4 | .0 | .0 | 95.0 |
|  | 1518 | 2 | .0 | .0 | 95.0 |
|  | 1519 | 6 | .0 | .0 | 95.1 |
|  | 1520 | 1 | .0 | .0 | 95.1 |
|  | 1521 | 1 | .0 | .0 | 95.1 |
|  | 1522 | 3 | .0 | .0 | 95.1 |
|  | 1523 | 2 | .0 | .0 | 95.1 |
|  | 1524 | 1 | .0 | .0 | 95.1 |
|  | 1525 | 5 | .0 | .0 | 95.1 |
|  | 1526 | 1 | .0 | .0 | 95.1 |
|  | 1527 | 3 | .0 | .0 | 95.1 |
|  | 1528 | 1 | .0 | .0 | 95.1 |
|  | 1529 | 2 | .0 | .0 | 95.1 |
|  | 1530 | 2 | .0 | .0 | 95.1 |
|  | 1531 | 5 | .0 | .0 | 95.1 |
|  | 1532 | 3 | .0 | .0 | 95.2 |
|  | 1533 | 1 | .0 | .0 | 95.2 |
|  | 1534 | 1 | .0 | .0 | 95.2 |
|  | 1536 | 3 | .0 | .0 | 95.2 |
|  | 1538 | 1 | .0 | .0 | 95.2 |
|  | 1539 | 2 | .0 | .0 | 95.2 |
|  | 1541 | 2 | .0 | .0 | 95.2 |
|  | 1542 | 1 | .0 | .0 | 95.2 |
|  | 1543 | 4 | .0 | .0 | 95.2 |
|  | 1544 | 3 | .0 | .0 | 95.2 |
|  | 1545 | 3 | .0 | .0 | 95.2 |
|  | 1546 | 1 | .0 | .0 | 95.2 |
|  | 1547 | 3 | .0 | .0 | 95.2 |
|  | 1548 | 1 | .0 | .0 | 95.2 |
|  | 1549 | 1 | .0 | .0 | 95.2 |
|  | 1550 | 7 | .0 | .0 | 95.3 |
|  | 1551 | 2 | .0 | .0 | 95.3 |
|  | 1552 | 2 | .0 | .0 | 95.3 |
|  | 1553 | 1 | .0 | .0 | 95.3 |
|  | 1554 | 1 | .0 | .0 | 95.3 |
|  | 1555 | 5 | .0 | .0 | 95.3 |
|  | 1556 | 4 | .0 | .0 | 95.3 |
|  | 1557 | 1 | .0 | .0 | 95.3 |
|  | 1558 | 8 | .0 | .0 | 95.3 |
|  | 1559 | 2 | .0 | .0 | 95.4 |
|  | 1560 | 3 | .0 | .0 | 95.4 |
|  | 1561 | 1 | .0 | .0 | 95.4 |
|  | 1562 | 2 | .0 | .0 | 95.4 |
|  | 1563 | 4 | .0 | .0 | 95.4 |
|  | 1564 | 2 | .0 | .0 | 95.4 |
|  | 1565 | 4 | .0 | .0 | 95.4 |
|  | 1566 | 1 | .0 | .0 | 95.4 |
|  | 1567 | 1 | .0 | .0 | 95.4 |
|  | 1569 | 3 | .0 | .0 | 95.4 |
|  | 1570 | 3 | .0 | .0 | 95.4 |
|  | 1572 | 2 | .0 | .0 | 95.4 |
|  | 1573 | 5 | .0 | .0 | 95.5 |
|  | 1574 | 5 | .0 | .0 | 95.5 |
|  | 1575 | 3 | .0 | .0 | 95.5 |
|  | 1576 | 4 | .0 | .0 | 95.5 |
|  | 1577 | 1 | .0 | .0 | 95.5 |
|  | 1578 | 3 | .0 | .0 | 95.5 |
|  | 1580 | 2 | .0 | .0 | 95.5 |
|  | 1581 | 1 | .0 | .0 | 95.5 |
|  | 1582 | 2 | .0 | .0 | 95.5 |
|  | 1583 | 3 | .0 | .0 | 95.5 |
|  | 1584 | 6 | .0 | .0 | 95.6 |
|  | 1585 | 2 | .0 | .0 | 95.6 |
|  | 1586 | 7 | .0 | .0 | 95.6 |
|  | 1587 | 6 | .0 | .0 | 95.6 |
|  | 1588 | 6 | .0 | .0 | 95.6 |
|  | 1589 | 2 | .0 | .0 | 95.6 |
|  | 1590 | 1 | .0 | .0 | 95.6 |
|  | 1591 | 3 | .0 | .0 | 95.6 |
|  | 1592 | 3 | .0 | .0 | 95.7 |
|  | 1593 | 5 | .0 | .0 | 95.7 |
|  | 1594 | 3 | .0 | .0 | 95.7 |
|  | 1595 | 6 | .0 | .0 | 95.7 |
|  | 1596 | 1 | .0 | .0 | 95.7 |
|  | 1597 | 3 | .0 | .0 | 95.7 |
|  | 1598 | 5 | .0 | .0 | 95.7 |
|  | 1599 | 1 | .0 | .0 | 95.7 |
|  | 1600 | 2 | .0 | .0 | 95.7 |
|  | 1601 | 3 | .0 | .0 | 95.7 |
|  | 1602 | 4 | .0 | .0 | 95.8 |
|  | 1603 | 2 | .0 | .0 | 95.8 |
|  | 1605 | 1 | .0 | .0 | 95.8 |
|  | 1606 | 1 | .0 | .0 | 95.8 |
|  | 1607 | 3 | .0 | .0 | 95.8 |
|  | 1608 | 2 | .0 | .0 | 95.8 |
|  | 1609 | 4 | .0 | .0 | 95.8 |
|  | 1610 | 2 | .0 | .0 | 95.8 |
|  | 1611 | 4 | .0 | .0 | 95.8 |
|  | 1612 | 4 | .0 | .0 | 95.8 |
|  | 1613 | 2 | .0 | .0 | 95.8 |
|  | 1614 | 1 | .0 | .0 | 95.8 |
|  | 1615 | 2 | .0 | .0 | 95.8 |
|  | 1616 | 3 | .0 | .0 | 95.9 |
|  | 1618 | 2 | .0 | .0 | 95.9 |
|  | 1619 | 1 | .0 | .0 | 95.9 |
|  | 1620 | 1 | .0 | .0 | 95.9 |
|  | 1621 | 3 | .0 | .0 | 95.9 |
|  | 1622 | 2 | .0 | .0 | 95.9 |
|  | 1624 | 2 | .0 | .0 | 95.9 |
|  | 1625 | 2 | .0 | .0 | 95.9 |
|  | 1626 | 5 | .0 | .0 | 95.9 |
|  | 1627 | 3 | .0 | .0 | 95.9 |
|  | 1629 | 1 | .0 | .0 | 95.9 |
|  | 1630 | 1 | .0 | .0 | 95.9 |
|  | 1631 | 4 | .0 | .0 | 95.9 |
|  | 1632 | 2 | .0 | .0 | 96.0 |
|  | 1633 | 2 | .0 | .0 | 96.0 |
|  | 1634 | 3 | .0 | .0 | 96.0 |
|  | 1635 | 4 | .0 | .0 | 96.0 |
|  | 1636 | 3 | .0 | .0 | 96.0 |
|  | 1637 | 2 | .0 | .0 | 96.0 |
|  | 1638 | 1 | .0 | .0 | 96.0 |
|  | 1639 | 1 | .0 | .0 | 96.0 |
|  | 1640 | 3 | .0 | .0 | 96.0 |
|  | 1642 | 2 | .0 | .0 | 96.0 |
|  | 1643 | 2 | .0 | .0 | 96.0 |
|  | 1645 | 1 | .0 | .0 | 96.0 |
|  | 1646 | 1 | .0 | .0 | 96.0 |
|  | 1647 | 1 | .0 | .0 | 96.0 |
|  | 1649 | 2 | .0 | .0 | 96.0 |
|  | 1650 | 4 | .0 | .0 | 96.1 |
|  | 1651 | 2 | .0 | .0 | 96.1 |
|  | 1652 | 1 | .0 | .0 | 96.1 |
|  | 1653 | 1 | .0 | .0 | 96.1 |
|  | 1655 | 3 | .0 | .0 | 96.1 |
|  | 1656 | 2 | .0 | .0 | 96.1 |
|  | 1657 | 2 | .0 | .0 | 96.1 |
|  | 1659 | 3 | .0 | .0 | 96.1 |
|  | 1660 | 1 | .0 | .0 | 96.1 |
|  | 1663 | 2 | .0 | .0 | 96.1 |
|  | 1664 | 1 | .0 | .0 | 96.1 |
|  | 1666 | 2 | .0 | .0 | 96.1 |
|  | 1667 | 7 | .0 | .0 | 96.1 |
|  | 1668 | 1 | .0 | .0 | 96.2 |
|  | 1669 | 3 | .0 | .0 | 96.2 |
|  | 1670 | 2 | .0 | .0 | 96.2 |
|  | 1671 | 1 | .0 | .0 | 96.2 |
|  | 1673 | 1 | .0 | .0 | 96.2 |
|  | 1674 | 2 | .0 | .0 | 96.2 |
|  | 1675 | 1 | .0 | .0 | 96.2 |
|  | 1676 | 1 | .0 | .0 | 96.2 |
|  | 1677 | 4 | .0 | .0 | 96.2 |
|  | 1679 | 1 | .0 | .0 | 96.2 |
|  | 1680 | 2 | .0 | .0 | 96.2 |
|  | 1681 | 1 | .0 | .0 | 96.2 |
|  | 1682 | 2 | .0 | .0 | 96.2 |
|  | 1683 | 1 | .0 | .0 | 96.2 |
|  | 1685 | 3 | .0 | .0 | 96.2 |
|  | 1686 | 1 | .0 | .0 | 96.2 |
|  | 1687 | 2 | .0 | .0 | 96.2 |
|  | 1688 | 2 | .0 | .0 | 96.2 |
|  | 1689 | 3 | .0 | .0 | 96.3 |
|  | 1690 | 1 | .0 | .0 | 96.3 |
|  | 1691 | 3 | .0 | .0 | 96.3 |
|  | 1692 | 4 | .0 | .0 | 96.3 |
|  | 1693 | 1 | .0 | .0 | 96.3 |
|  | 1694 | 4 | .0 | .0 | 96.3 |
|  | 1695 | 1 | .0 | .0 | 96.3 |
|  | 1696 | 1 | .0 | .0 | 96.3 |
|  | 1697 | 1 | .0 | .0 | 96.3 |
|  | 1698 | 2 | .0 | .0 | 96.3 |
|  | 1699 | 1 | .0 | .0 | 96.3 |
|  | 1700 | 2 | .0 | .0 | 96.3 |
|  | 1701 | 2 | .0 | .0 | 96.3 |
|  | 1703 | 2 | .0 | .0 | 96.3 |
|  | 1705 | 2 | .0 | .0 | 96.3 |
|  | 1707 | 1 | .0 | .0 | 96.4 |
|  | 1708 | 2 | .0 | .0 | 96.4 |
|  | 1709 | 2 | .0 | .0 | 96.4 |
|  | 1710 | 1 | .0 | .0 | 96.4 |
|  | 1711 | 1 | .0 | .0 | 96.4 |
|  | 1715 | 3 | .0 | .0 | 96.4 |
|  | 1717 | 2 | .0 | .0 | 96.4 |
|  | 1718 | 2 | .0 | .0 | 96.4 |
|  | 1720 | 1 | .0 | .0 | 96.4 |
|  | 1721 | 1 | .0 | .0 | 96.4 |
|  | 1723 | 1 | .0 | .0 | 96.4 |
|  | 1724 | 2 | .0 | .0 | 96.4 |
|  | 1727 | 1 | .0 | .0 | 96.4 |
|  | 1728 | 3 | .0 | .0 | 96.4 |
|  | 1729 | 3 | .0 | .0 | 96.4 |
|  | 1730 | 3 | .0 | .0 | 96.4 |
|  | 1731 | 2 | .0 | .0 | 96.4 |
|  | 1732 | 2 | .0 | .0 | 96.5 |
|  | 1733 | 2 | .0 | .0 | 96.5 |
|  | 1736 | 1 | .0 | .0 | 96.5 |
|  | 1737 | 3 | .0 | .0 | 96.5 |
|  | 1738 | 3 | .0 | .0 | 96.5 |
|  | 1739 | 3 | .0 | .0 | 96.5 |
|  | 1742 | 2 | .0 | .0 | 96.5 |
|  | 1743 | 3 | .0 | .0 | 96.5 |
|  | 1744 | 3 | .0 | .0 | 96.5 |
|  | 1745 | 2 | .0 | .0 | 96.5 |
|  | 1747 | 3 | .0 | .0 | 96.5 |
|  | 1748 | 3 | .0 | .0 | 96.5 |
|  | 1749 | 2 | .0 | .0 | 96.6 |
|  | 1750 | 1 | .0 | .0 | 96.6 |
|  | 1751 | 2 | .0 | .0 | 96.6 |
|  | 1752 | 1 | .0 | .0 | 96.6 |
|  | 1753 | 1 | .0 | .0 | 96.6 |
|  | 1754 | 2 | .0 | .0 | 96.6 |
|  | 1755 | 2 | .0 | .0 | 96.6 |
|  | 1756 | 2 | .0 | .0 | 96.6 |
|  | 1757 | 1 | .0 | .0 | 96.6 |
|  | 1758 | 2 | .0 | .0 | 96.6 |
|  | 1759 | 1 | .0 | .0 | 96.6 |
|  | 1762 | 3 | .0 | .0 | 96.6 |
|  | 1763 | 1 | .0 | .0 | 96.6 |
|  | 1764 | 2 | .0 | .0 | 96.6 |
|  | 1766 | 1 | .0 | .0 | 96.6 |
|  | 1767 | 1 | .0 | .0 | 96.6 |
|  | 1768 | 1 | .0 | .0 | 96.6 |
|  | 1769 | 2 | .0 | .0 | 96.6 |
|  | 1770 | 2 | .0 | .0 | 96.6 |
|  | 1771 | 3 | .0 | .0 | 96.7 |
|  | 1772 | 1 | .0 | .0 | 96.7 |
|  | 1773 | 2 | .0 | .0 | 96.7 |
|  | 1775 | 2 | .0 | .0 | 96.7 |
|  | 1776 | 2 | .0 | .0 | 96.7 |
|  | 1777 | 2 | .0 | .0 | 96.7 |
|  | 1778 | 2 | .0 | .0 | 96.7 |
|  | 1779 | 2 | .0 | .0 | 96.7 |
|  | 1780 | 1 | .0 | .0 | 96.7 |
|  | 1781 | 2 | .0 | .0 | 96.7 |
|  | 1782 | 4 | .0 | .0 | 96.7 |
|  | 1783 | 2 | .0 | .0 | 96.7 |
|  | 1785 | 2 | .0 | .0 | 96.7 |
|  | 1786 | 1 | .0 | .0 | 96.7 |
|  | 1787 | 1 | .0 | .0 | 96.7 |
|  | 1788 | 2 | .0 | .0 | 96.7 |
|  | 1790 | 3 | .0 | .0 | 96.8 |
|  | 1791 | 1 | .0 | .0 | 96.8 |
|  | 1792 | 2 | .0 | .0 | 96.8 |
|  | 1793 | 4 | .0 | .0 | 96.8 |
|  | 1794 | 1 | .0 | .0 | 96.8 |
|  | 1796 | 1 | .0 | .0 | 96.8 |
|  | 1797 | 2 | .0 | .0 | 96.8 |
|  | 1798 | 3 | .0 | .0 | 96.8 |
|  | 1800 | 2 | .0 | .0 | 96.8 |
|  | 1802 | 3 | .0 | .0 | 96.8 |
|  | 1803 | 2 | .0 | .0 | 96.8 |
|  | 1804 | 4 | .0 | .0 | 96.8 |
|  | 1805 | 1 | .0 | .0 | 96.8 |
|  | 1806 | 5 | .0 | .0 | 96.9 |
|  | 1808 | 5 | .0 | .0 | 96.9 |
|  | 1810 | 1 | .0 | .0 | 96.9 |
|  | 1811 | 2 | .0 | .0 | 96.9 |
|  | 1812 | 2 | .0 | .0 | 96.9 |
|  | 1813 | 3 | .0 | .0 | 96.9 |
|  | 1814 | 1 | .0 | .0 | 96.9 |
|  | 1815 | 3 | .0 | .0 | 96.9 |
|  | 1816 | 2 | .0 | .0 | 96.9 |
|  | 1818 | 1 | .0 | .0 | 96.9 |
|  | 1821 | 1 | .0 | .0 | 96.9 |
|  | 1822 | 2 | .0 | .0 | 96.9 |
|  | 1823 | 1 | .0 | .0 | 96.9 |
|  | 1824 | 2 | .0 | .0 | 96.9 |
|  | 1825 | 2 | .0 | .0 | 96.9 |
|  | 1827 | 1 | .0 | .0 | 97.0 |
|  | 1828 | 3 | .0 | .0 | 97.0 |
|  | 1829 | 2 | .0 | .0 | 97.0 |
|  | 1830 | 1 | .0 | .0 | 97.0 |
|  | 1831 | 2 | .0 | .0 | 97.0 |
|  | 1832 | 1 | .0 | .0 | 97.0 |
|  | 1833 | 1 | .0 | .0 | 97.0 |
|  | 1834 | 1 | .0 | .0 | 97.0 |
|  | 1835 | 1 | .0 | .0 | 97.0 |
|  | 1839 | 2 | .0 | .0 | 97.0 |
|  | 1841 | 1 | .0 | .0 | 97.0 |
|  | 1842 | 4 | .0 | .0 | 97.0 |
|  | 1844 | 2 | .0 | .0 | 97.0 |
|  | 1845 | 1 | .0 | .0 | 97.0 |
|  | 1846 | 2 | .0 | .0 | 97.0 |
|  | 1847 | 1 | .0 | .0 | 97.0 |
|  | 1848 | 1 | .0 | .0 | 97.0 |
|  | 1849 | 1 | .0 | .0 | 97.0 |
|  | 1850 | 1 | .0 | .0 | 97.0 |
|  | 1851 | 2 | .0 | .0 | 97.1 |
|  | 1853 | 2 | .0 | .0 | 97.1 |
|  | 1854 | 1 | .0 | .0 | 97.1 |
|  | 1856 | 2 | .0 | .0 | 97.1 |
|  | 1858 | 3 | .0 | .0 | 97.1 |
|  | 1859 | 4 | .0 | .0 | 97.1 |
|  | 1861 | 1 | .0 | .0 | 97.1 |
|  | 1863 | 1 | .0 | .0 | 97.1 |
|  | 1864 | 2 | .0 | .0 | 97.1 |
|  | 1867 | 1 | .0 | .0 | 97.1 |
|  | 1868 | 1 | .0 | .0 | 97.1 |
|  | 1870 | 3 | .0 | .0 | 97.1 |
|  | 1872 | 2 | .0 | .0 | 97.1 |
|  | 1873 | 1 | .0 | .0 | 97.1 |
|  | 1875 | 1 | .0 | .0 | 97.1 |
|  | 1876 | 2 | .0 | .0 | 97.1 |
|  | 1878 | 1 | .0 | .0 | 97.1 |
|  | 1882 | 3 | .0 | .0 | 97.2 |
|  | 1883 | 1 | .0 | .0 | 97.2 |
|  | 1884 | 2 | .0 | .0 | 97.2 |
|  | 1885 | 1 | .0 | .0 | 97.2 |
|  | 1886 | 1 | .0 | .0 | 97.2 |
|  | 1890 | 5 | .0 | .0 | 97.2 |
|  | 1891 | 3 | .0 | .0 | 97.2 |
|  | 1892 | 1 | .0 | .0 | 97.2 |
|  | 1893 | 1 | .0 | .0 | 97.2 |
|  | 1894 | 1 | .0 | .0 | 97.2 |
|  | 1896 | 4 | .0 | .0 | 97.2 |
|  | 1899 | 3 | .0 | .0 | 97.2 |
|  | 1900 | 2 | .0 | .0 | 97.2 |
|  | 1901 | 1 | .0 | .0 | 97.2 |
|  | 1904 | 1 | .0 | .0 | 97.2 |
|  | 1905 | 3 | .0 | .0 | 97.3 |
|  | 1908 | 4 | .0 | .0 | 97.3 |
|  | 1909 | 2 | .0 | .0 | 97.3 |
|  | 1910 | 1 | .0 | .0 | 97.3 |
|  | 1911 | 2 | .0 | .0 | 97.3 |
|  | 1912 | 1 | .0 | .0 | 97.3 |
|  | 1913 | 2 | .0 | .0 | 97.3 |
|  | 1914 | 1 | .0 | .0 | 97.3 |
|  | 1915 | 2 | .0 | .0 | 97.3 |
|  | 1918 | 1 | .0 | .0 | 97.3 |
|  | 1919 | 2 | .0 | .0 | 97.3 |
|  | 1921 | 1 | .0 | .0 | 97.3 |
|  | 1922 | 2 | .0 | .0 | 97.3 |
|  | 1923 | 1 | .0 | .0 | 97.3 |
|  | 1927 | 1 | .0 | .0 | 97.3 |
|  | 1930 | 1 | .0 | .0 | 97.3 |
|  | 1932 | 2 | .0 | .0 | 97.3 |
|  | 1933 | 1 | .0 | .0 | 97.3 |
|  | 1935 | 1 | .0 | .0 | 97.3 |
|  | 1936 | 1 | .0 | .0 | 97.3 |
|  | 1937 | 1 | .0 | .0 | 97.3 |
|  | 1938 | 1 | .0 | .0 | 97.4 |
|  | 1940 | 1 | .0 | .0 | 97.4 |
|  | 1941 | 3 | .0 | .0 | 97.4 |
|  | 1942 | 1 | .0 | .0 | 97.4 |
|  | 1943 | 3 | .0 | .0 | 97.4 |
|  | 1944 | 1 | .0 | .0 | 97.4 |
|  | 1945 | 4 | .0 | .0 | 97.4 |
|  | 1946 | 1 | .0 | .0 | 97.4 |
|  | 1950 | 1 | .0 | .0 | 97.4 |
|  | 1951 | 2 | .0 | .0 | 97.4 |
|  | 1954 | 1 | .0 | .0 | 97.4 |
|  | 1955 | 1 | .0 | .0 | 97.4 |
|  | 1956 | 3 | .0 | .0 | 97.4 |
|  | 1958 | 2 | .0 | .0 | 97.4 |
|  | 1960 | 2 | .0 | .0 | 97.4 |
|  | 1962 | 1 | .0 | .0 | 97.4 |
|  | 1965 | 1 | .0 | .0 | 97.4 |
|  | 1966 | 2 | .0 | .0 | 97.5 |
|  | 1968 | 1 | .0 | .0 | 97.5 |
|  | 1974 | 3 | .0 | .0 | 97.5 |
|  | 1975 | 1 | .0 | .0 | 97.5 |
|  | 1976 | 2 | .0 | .0 | 97.5 |
|  | 1978 | 1 | .0 | .0 | 97.5 |
|  | 1979 | 1 | .0 | .0 | 97.5 |
|  | 1980 | 1 | .0 | .0 | 97.5 |
|  | 1981 | 2 | .0 | .0 | 97.5 |
|  | 1982 | 1 | .0 | .0 | 97.5 |
|  | 1983 | 1 | .0 | .0 | 97.5 |
|  | 1984 | 1 | .0 | .0 | 97.5 |
|  | 1985 | 1 | .0 | .0 | 97.5 |
|  | 1986 | 2 | .0 | .0 | 97.5 |
|  | 1987 | 1 | .0 | .0 | 97.5 |
|  | 1988 | 3 | .0 | .0 | 97.5 |
|  | 1989 | 2 | .0 | .0 | 97.5 |
|  | 1990 | 1 | .0 | .0 | 97.5 |
|  | 1991 | 1 | .0 | .0 | 97.5 |
|  | 1992 | 2 | .0 | .0 | 97.5 |
|  | 1993 | 1 | .0 | .0 | 97.5 |
|  | 1996 | 1 | .0 | .0 | 97.5 |
|  | 1998 | 2 | .0 | .0 | 97.6 |
|  | 2000 | 1 | .0 | .0 | 97.6 |
|  | 2002 | 1 | .0 | .0 | 97.6 |
|  | 2003 | 1 | .0 | .0 | 97.6 |
|  | 2005 | 1 | .0 | .0 | 97.6 |
|  | 2008 | 1 | .0 | .0 | 97.6 |
|  | 2009 | 1 | .0 | .0 | 97.6 |
|  | 2013 | 2 | .0 | .0 | 97.6 |
|  | 2015 | 2 | .0 | .0 | 97.6 |
|  | 2019 | 1 | .0 | .0 | 97.6 |
|  | 2022 | 2 | .0 | .0 | 97.6 |
|  | 2023 | 1 | .0 | .0 | 97.6 |
|  | 2024 | 1 | .0 | .0 | 97.6 |
|  | 2027 | 1 | .0 | .0 | 97.6 |
|  | 2028 | 2 | .0 | .0 | 97.6 |
|  | 2029 | 1 | .0 | .0 | 97.6 |
|  | 2030 | 2 | .0 | .0 | 97.6 |
|  | 2033 | 2 | .0 | .0 | 97.6 |
|  | 2034 | 1 | .0 | .0 | 97.6 |
|  | 2036 | 3 | .0 | .0 | 97.6 |
|  | 2038 | 1 | .0 | .0 | 97.6 |
|  | 2046 | 1 | .0 | .0 | 97.6 |
|  | 2048 | 2 | .0 | .0 | 97.7 |
|  | 2049 | 1 | .0 | .0 | 97.7 |
|  | 2050 | 1 | .0 | .0 | 97.7 |
|  | 2051 | 1 | .0 | .0 | 97.7 |
|  | 2053 | 1 | .0 | .0 | 97.7 |
|  | 2054 | 1 | .0 | .0 | 97.7 |
|  | 2055 | 1 | .0 | .0 | 97.7 |
|  | 2057 | 1 | .0 | .0 | 97.7 |
|  | 2058 | 1 | .0 | .0 | 97.7 |
|  | 2060 | 1 | .0 | .0 | 97.7 |
|  | 2061 | 1 | .0 | .0 | 97.7 |
|  | 2064 | 2 | .0 | .0 | 97.7 |
|  | 2065 | 3 | .0 | .0 | 97.7 |
|  | 2066 | 2 | .0 | .0 | 97.7 |
|  | 2067 | 1 | .0 | .0 | 97.7 |
|  | 2069 | 2 | .0 | .0 | 97.7 |
|  | 2071 | 1 | .0 | .0 | 97.7 |
|  | 2072 | 1 | .0 | .0 | 97.7 |
|  | 2074 | 2 | .0 | .0 | 97.7 |
|  | 2076 | 2 | .0 | .0 | 97.7 |
|  | 2078 | 1 | .0 | .0 | 97.7 |
|  | 2080 | 1 | .0 | .0 | 97.7 |
|  | 2082 | 1 | .0 | .0 | 97.8 |
|  | 2083 | 1 | .0 | .0 | 97.8 |
|  | 2084 | 1 | .0 | .0 | 97.8 |
|  | 2085 | 1 | .0 | .0 | 97.8 |
|  | 2086 | 1 | .0 | .0 | 97.8 |
|  | 2087 | 1 | .0 | .0 | 97.8 |
|  | 2088 | 2 | .0 | .0 | 97.8 |
|  | 2092 | 1 | .0 | .0 | 97.8 |
|  | 2096 | 1 | .0 | .0 | 97.8 |
|  | 2099 | 1 | .0 | .0 | 97.8 |
|  | 2100 | 1 | .0 | .0 | 97.8 |
|  | 2101 | 1 | .0 | .0 | 97.8 |
|  | 2102 | 3 | .0 | .0 | 97.8 |
|  | 2103 | 1 | .0 | .0 | 97.8 |
|  | 2106 | 1 | .0 | .0 | 97.8 |
|  | 2110 | 1 | .0 | .0 | 97.8 |
|  | 2111 | 1 | .0 | .0 | 97.8 |
|  | 2112 | 1 | .0 | .0 | 97.8 |
|  | 2114 | 1 | .0 | .0 | 97.8 |
|  | 2115 | 2 | .0 | .0 | 97.8 |
|  | 2120 | 1 | .0 | .0 | 97.8 |
|  | 2121 | 1 | .0 | .0 | 97.8 |
|  | 2122 | 1 | .0 | .0 | 97.8 |
|  | 2123 | 4 | .0 | .0 | 97.8 |
|  | 2124 | 1 | .0 | .0 | 97.9 |
|  | 2125 | 1 | .0 | .0 | 97.9 |
|  | 2126 | 1 | .0 | .0 | 97.9 |
|  | 2129 | 1 | .0 | .0 | 97.9 |
|  | 2130 | 1 | .0 | .0 | 97.9 |
|  | 2131 | 1 | .0 | .0 | 97.9 |
|  | 2135 | 1 | .0 | .0 | 97.9 |
|  | 2136 | 1 | .0 | .0 | 97.9 |
|  | 2138 | 2 | .0 | .0 | 97.9 |
|  | 2139 | 2 | .0 | .0 | 97.9 |
|  | 2143 | 1 | .0 | .0 | 97.9 |
|  | 2145 | 1 | .0 | .0 | 97.9 |
|  | 2146 | 2 | .0 | .0 | 97.9 |
|  | 2148 | 1 | .0 | .0 | 97.9 |
|  | 2151 | 2 | .0 | .0 | 97.9 |
|  | 2152 | 1 | .0 | .0 | 97.9 |
|  | 2153 | 1 | .0 | .0 | 97.9 |
|  | 2154 | 1 | .0 | .0 | 97.9 |
|  | 2155 | 1 | .0 | .0 | 97.9 |
|  | 2160 | 2 | .0 | .0 | 97.9 |
|  | 2161 | 1 | .0 | .0 | 97.9 |
|  | 2165 | 2 | .0 | .0 | 97.9 |
|  | 2169 | 1 | .0 | .0 | 97.9 |
|  | 2172 | 1 | .0 | .0 | 97.9 |
|  | 2175 | 2 | .0 | .0 | 98.0 |
|  | 2176 | 2 | .0 | .0 | 98.0 |
|  | 2177 | 2 | .0 | .0 | 98.0 |
|  | 2178 | 1 | .0 | .0 | 98.0 |
|  | 2179 | 1 | .0 | .0 | 98.0 |
|  | 2180 | 1 | .0 | .0 | 98.0 |
|  | 2185 | 2 | .0 | .0 | 98.0 |
|  | 2187 | 2 | .0 | .0 | 98.0 |
|  | 2193 | 1 | .0 | .0 | 98.0 |
|  | 2194 | 1 | .0 | .0 | 98.0 |
|  | 2196 | 2 | .0 | .0 | 98.0 |
|  | 2198 | 2 | .0 | .0 | 98.0 |
|  | 2199 | 1 | .0 | .0 | 98.0 |
|  | 2201 | 2 | .0 | .0 | 98.0 |
|  | 2204 | 1 | .0 | .0 | 98.0 |
|  | 2208 | 1 | .0 | .0 | 98.0 |
|  | 2209 | 2 | .0 | .0 | 98.0 |
|  | 2210 | 1 | .0 | .0 | 98.0 |
|  | 2211 | 1 | .0 | .0 | 98.0 |
|  | 2212 | 2 | .0 | .0 | 98.0 |
|  | 2213 | 1 | .0 | .0 | 98.0 |
|  | 2214 | 1 | .0 | .0 | 98.1 |
|  | 2216 | 1 | .0 | .0 | 98.1 |
|  | 2217 | 3 | .0 | .0 | 98.1 |
|  | 2218 | 3 | .0 | .0 | 98.1 |
|  | 2219 | 1 | .0 | .0 | 98.1 |
|  | 2221 | 3 | .0 | .0 | 98.1 |
|  | 2222 | 1 | .0 | .0 | 98.1 |
|  | 2223 | 1 | .0 | .0 | 98.1 |
|  | 2224 | 1 | .0 | .0 | 98.1 |
|  | 2225 | 1 | .0 | .0 | 98.1 |
|  | 2227 | 1 | .0 | .0 | 98.1 |
|  | 2229 | 1 | .0 | .0 | 98.1 |
|  | 2235 | 1 | .0 | .0 | 98.1 |
|  | 2237 | 1 | .0 | .0 | 98.1 |
|  | 2239 | 4 | .0 | .0 | 98.1 |
|  | 2240 | 1 | .0 | .0 | 98.1 |
|  | 2241 | 1 | .0 | .0 | 98.1 |
|  | 2242 | 1 | .0 | .0 | 98.1 |
|  | 2244 | 1 | .0 | .0 | 98.1 |
|  | 2246 | 2 | .0 | .0 | 98.1 |
|  | 2247 | 1 | .0 | .0 | 98.2 |
|  | 2249 | 2 | .0 | .0 | 98.2 |
|  | 2252 | 2 | .0 | .0 | 98.2 |
|  | 2253 | 2 | .0 | .0 | 98.2 |
|  | 2257 | 2 | .0 | .0 | 98.2 |
|  | 2258 | 1 | .0 | .0 | 98.2 |
|  | 2262 | 1 | .0 | .0 | 98.2 |
|  | 2263 | 1 | .0 | .0 | 98.2 |
|  | 2265 | 1 | .0 | .0 | 98.2 |
|  | 2266 | 1 | .0 | .0 | 98.2 |
|  | 2267 | 1 | .0 | .0 | 98.2 |
|  | 2272 | 1 | .0 | .0 | 98.2 |
|  | 2275 | 1 | .0 | .0 | 98.2 |
|  | 2277 | 1 | .0 | .0 | 98.2 |
|  | 2279 | 1 | .0 | .0 | 98.2 |
|  | 2280 | 2 | .0 | .0 | 98.2 |
|  | 2282 | 1 | .0 | .0 | 98.2 |
|  | 2283 | 1 | .0 | .0 | 98.2 |
|  | 2284 | 1 | .0 | .0 | 98.2 |
|  | 2288 | 3 | .0 | .0 | 98.2 |
|  | 2289 | 1 | .0 | .0 | 98.2 |
|  | 2291 | 1 | .0 | .0 | 98.2 |
|  | 2292 | 1 | .0 | .0 | 98.2 |
|  | 2293 | 1 | .0 | .0 | 98.2 |
|  | 2294 | 1 | .0 | .0 | 98.3 |
|  | 2299 | 1 | .0 | .0 | 98.3 |
|  | 2300 | 1 | .0 | .0 | 98.3 |
|  | 2302 | 1 | .0 | .0 | 98.3 |
|  | 2303 | 1 | .0 | .0 | 98.3 |
|  | 2304 | 1 | .0 | .0 | 98.3 |
|  | 2308 | 1 | .0 | .0 | 98.3 |
|  | 2311 | 1 | .0 | .0 | 98.3 |
|  | 2313 | 1 | .0 | .0 | 98.3 |
|  | 2316 | 1 | .0 | .0 | 98.3 |
|  | 2318 | 1 | .0 | .0 | 98.3 |
|  | 2319 | 1 | .0 | .0 | 98.3 |
|  | 2320 | 1 | .0 | .0 | 98.3 |
|  | 2321 | 2 | .0 | .0 | 98.3 |
|  | 2322 | 1 | .0 | .0 | 98.3 |
|  | 2325 | 2 | .0 | .0 | 98.3 |
|  | 2326 | 1 | .0 | .0 | 98.3 |
|  | 2327 | 2 | .0 | .0 | 98.3 |
|  | 2329 | 1 | .0 | .0 | 98.3 |
|  | 2330 | 2 | .0 | .0 | 98.3 |
|  | 2331 | 1 | .0 | .0 | 98.3 |
|  | 2335 | 1 | .0 | .0 | 98.3 |
|  | 2336 | 1 | .0 | .0 | 98.3 |
|  | 2337 | 1 | .0 | .0 | 98.3 |
|  | 2341 | 1 | .0 | .0 | 98.3 |
|  | 2343 | 1 | .0 | .0 | 98.3 |
|  | 2344 | 1 | .0 | .0 | 98.4 |
|  | 2345 | 1 | .0 | .0 | 98.4 |
|  | 2346 | 1 | .0 | .0 | 98.4 |
|  | 2347 | 1 | .0 | .0 | 98.4 |
|  | 2348 | 1 | .0 | .0 | 98.4 |
|  | 2349 | 1 | .0 | .0 | 98.4 |
|  | 2350 | 2 | .0 | .0 | 98.4 |
|  | 2351 | 1 | .0 | .0 | 98.4 |
|  | 2352 | 2 | .0 | .0 | 98.4 |
|  | 2354 | 1 | .0 | .0 | 98.4 |
|  | 2355 | 1 | .0 | .0 | 98.4 |
|  | 2356 | 2 | .0 | .0 | 98.4 |
|  | 2357 | 1 | .0 | .0 | 98.4 |
|  | 2362 | 2 | .0 | .0 | 98.4 |
|  | 2363 | 1 | .0 | .0 | 98.4 |
|  | 2364 | 4 | .0 | .0 | 98.4 |
|  | 2366 | 1 | .0 | .0 | 98.4 |
|  | 2368 | 1 | .0 | .0 | 98.4 |
|  | 2369 | 1 | .0 | .0 | 98.4 |
|  | 2372 | 2 | .0 | .0 | 98.4 |
|  | 2374 | 3 | .0 | .0 | 98.4 |
|  | 2381 | 1 | .0 | .0 | 98.5 |
|  | 2383 | 1 | .0 | .0 | 98.5 |
|  | 2384 | 1 | .0 | .0 | 98.5 |
|  | 2385 | 1 | .0 | .0 | 98.5 |
|  | 2386 | 2 | .0 | .0 | 98.5 |
|  | 2388 | 2 | .0 | .0 | 98.5 |
|  | 2395 | 2 | .0 | .0 | 98.5 |
|  | 2396 | 1 | .0 | .0 | 98.5 |
|  | 2397 | 1 | .0 | .0 | 98.5 |
|  | 2400 | 1 | .0 | .0 | 98.5 |
|  | 2402 | 1 | .0 | .0 | 98.5 |
|  | 2403 | 1 | .0 | .0 | 98.5 |
|  | 2404 | 1 | .0 | .0 | 98.5 |
|  | 2408 | 2 | .0 | .0 | 98.5 |
|  | 2412 | 1 | .0 | .0 | 98.5 |
|  | 2414 | 2 | .0 | .0 | 98.5 |
|  | 2416 | 1 | .0 | .0 | 98.5 |
|  | 2417 | 2 | .0 | .0 | 98.5 |
|  | 2418 | 2 | .0 | .0 | 98.5 |
|  | 2419 | 1 | .0 | .0 | 98.5 |
|  | 2420 | 1 | .0 | .0 | 98.5 |
|  | 2421 | 1 | .0 | .0 | 98.5 |
|  | 2424 | 1 | .0 | .0 | 98.5 |
|  | 2426 | 1 | .0 | .0 | 98.6 |
|  | 2427 | 1 | .0 | .0 | 98.6 |
|  | 2428 | 1 | .0 | .0 | 98.6 |
|  | 2429 | 1 | .0 | .0 | 98.6 |
|  | 2430 | 2 | .0 | .0 | 98.6 |
|  | 2431 | 1 | .0 | .0 | 98.6 |
|  | 2432 | 1 | .0 | .0 | 98.6 |
|  | 2434 | 1 | .0 | .0 | 98.6 |
|  | 2437 | 2 | .0 | .0 | 98.6 |
|  | 2440 | 1 | .0 | .0 | 98.6 |
|  | 2441 | 1 | .0 | .0 | 98.6 |
|  | 2443 | 1 | .0 | .0 | 98.6 |
|  | 2445 | 1 | .0 | .0 | 98.6 |
|  | 2446 | 1 | .0 | .0 | 98.6 |
|  | 2450 | 1 | .0 | .0 | 98.6 |
|  | 2452 | 3 | .0 | .0 | 98.6 |
|  | 2454 | 1 | .0 | .0 | 98.6 |
|  | 2455 | 1 | .0 | .0 | 98.6 |
|  | 2456 | 1 | .0 | .0 | 98.6 |
|  | 2459 | 1 | .0 | .0 | 98.6 |
|  | 2461 | 1 | .0 | .0 | 98.6 |
|  | 2462 | 1 | .0 | .0 | 98.6 |
|  | 2463 | 1 | .0 | .0 | 98.6 |
|  | 2464 | 1 | .0 | .0 | 98.6 |
|  | 2465 | 1 | .0 | .0 | 98.6 |
|  | 2466 | 1 | .0 | .0 | 98.6 |
|  | 2469 | 1 | .0 | .0 | 98.6 |
|  | 2470 | 2 | .0 | .0 | 98.7 |
|  | 2471 | 2 | .0 | .0 | 98.7 |
|  | 2472 | 1 | .0 | .0 | 98.7 |
|  | 2473 | 1 | .0 | .0 | 98.7 |
|  | 2474 | 1 | .0 | .0 | 98.7 |
|  | 2475 | 1 | .0 | .0 | 98.7 |
|  | 2477 | 1 | .0 | .0 | 98.7 |
|  | 2479 | 4 | .0 | .0 | 98.7 |
|  | 2481 | 1 | .0 | .0 | 98.7 |
|  | 2482 | 3 | .0 | .0 | 98.7 |
|  | 2483 | 1 | .0 | .0 | 98.7 |
|  | 2485 | 1 | .0 | .0 | 98.7 |
|  | 2487 | 1 | .0 | .0 | 98.7 |
|  | 2488 | 1 | .0 | .0 | 98.7 |
|  | 2491 | 1 | .0 | .0 | 98.7 |
|  | 2495 | 1 | .0 | .0 | 98.7 |
|  | 2497 | 1 | .0 | .0 | 98.7 |
|  | 2499 | 2 | .0 | .0 | 98.7 |
|  | 2500 | 1 | .0 | .0 | 98.7 |
|  | 2507 | 2 | .0 | .0 | 98.7 |
|  | 2509 | 1 | .0 | .0 | 98.7 |
|  | 2512 | 2 | .0 | .0 | 98.8 |
|  | 2515 | 2 | .0 | .0 | 98.8 |
|  | 2516 | 2 | .0 | .0 | 98.8 |
|  | 2519 | 2 | .0 | .0 | 98.8 |
|  | 2522 | 2 | .0 | .0 | 98.8 |
|  | 2524 | 1 | .0 | .0 | 98.8 |
|  | 2525 | 1 | .0 | .0 | 98.8 |
|  | 2526 | 1 | .0 | .0 | 98.8 |
|  | 2527 | 2 | .0 | .0 | 98.8 |
|  | 2528 | 1 | .0 | .0 | 98.8 |
|  | 2529 | 1 | .0 | .0 | 98.8 |
|  | 2530 | 1 | .0 | .0 | 98.8 |
|  | 2531 | 1 | .0 | .0 | 98.8 |
|  | 2532 | 2 | .0 | .0 | 98.8 |
|  | 2535 | 1 | .0 | .0 | 98.8 |
|  | 2536 | 2 | .0 | .0 | 98.8 |
|  | 2538 | 1 | .0 | .0 | 98.8 |
|  | 2539 | 1 | .0 | .0 | 98.8 |
|  | 2540 | 1 | .0 | .0 | 98.8 |
|  | 2543 | 2 | .0 | .0 | 98.8 |
|  | 2544 | 1 | .0 | .0 | 98.8 |
|  | 2545 | 1 | .0 | .0 | 98.8 |
|  | 2546 | 1 | .0 | .0 | 98.9 |
|  | 2547 | 2 | .0 | .0 | 98.9 |
|  | 2548 | 2 | .0 | .0 | 98.9 |
|  | 2553 | 1 | .0 | .0 | 98.9 |
|  | 2554 | 1 | .0 | .0 | 98.9 |
|  | 2555 | 2 | .0 | .0 | 98.9 |
|  | 2556 | 1 | .0 | .0 | 98.9 |
|  | 2557 | 1 | .0 | .0 | 98.9 |
|  | 2558 | 1 | .0 | .0 | 98.9 |
|  | 2559 | 1 | .0 | .0 | 98.9 |
|  | 2560 | 2 | .0 | .0 | 98.9 |
|  | 2565 | 1 | .0 | .0 | 98.9 |
|  | 2566 | 2 | .0 | .0 | 98.9 |
|  | 2567 | 1 | .0 | .0 | 98.9 |
|  | 2568 | 3 | .0 | .0 | 98.9 |
|  | 2569 | 1 | .0 | .0 | 98.9 |
|  | 2571 | 1 | .0 | .0 | 98.9 |
|  | 2572 | 1 | .0 | .0 | 98.9 |
|  | 2573 | 1 | .0 | .0 | 98.9 |
|  | 2575 | 1 | .0 | .0 | 98.9 |
|  | 2576 | 1 | .0 | .0 | 98.9 |
|  | 2577 | 3 | .0 | .0 | 98.9 |
|  | 2578 | 1 | .0 | .0 | 99.0 |
|  | 2579 | 1 | .0 | .0 | 99.0 |
|  | 2580 | 1 | .0 | .0 | 99.0 |
|  | 2581 | 1 | .0 | .0 | 99.0 |
|  | 2583 | 1 | .0 | .0 | 99.0 |
|  | 2584 | 1 | .0 | .0 | 99.0 |
|  | 2587 | 1 | .0 | .0 | 99.0 |
|  | 2588 | 2 | .0 | .0 | 99.0 |
|  | 2591 | 1 | .0 | .0 | 99.0 |
|  | 2594 | 2 | .0 | .0 | 99.0 |
|  | 2604 | 1 | .0 | .0 | 99.0 |
|  | 2607 | 1 | .0 | .0 | 99.0 |
|  | 2608 | 1 | .0 | .0 | 99.0 |
|  | 2611 | 2 | .0 | .0 | 99.0 |
|  | 2623 | 1 | .0 | .0 | 99.0 |
|  | 2627 | 1 | .0 | .0 | 99.0 |
|  | 2631 | 1 | .0 | .0 | 99.0 |
|  | 2632 | 2 | .0 | .0 | 99.0 |
|  | 2636 | 1 | .0 | .0 | 99.0 |
|  | 2639 | 1 | .0 | .0 | 99.0 |
|  | 2641 | 1 | .0 | .0 | 99.0 |
|  | 2643 | 1 | .0 | .0 | 99.0 |
|  | 2644 | 1 | .0 | .0 | 99.0 |
|  | 2648 | 1 | .0 | .0 | 99.0 |
|  | 2649 | 1 | .0 | .0 | 99.0 |
|  | 2652 | 1 | .0 | .0 | 99.0 |
|  | 2658 | 1 | .0 | .0 | 99.1 |
|  | 2659 | 3 | .0 | .0 | 99.1 |
|  | 2660 | 1 | .0 | .0 | 99.1 |
|  | 2661 | 1 | .0 | .0 | 99.1 |
|  | 2666 | 1 | .0 | .0 | 99.1 |
|  | 2667 | 1 | .0 | .0 | 99.1 |
|  | 2669 | 1 | .0 | .0 | 99.1 |
|  | 2671 | 1 | .0 | .0 | 99.1 |
|  | 2673 | 1 | .0 | .0 | 99.1 |
|  | 2674 | 1 | .0 | .0 | 99.1 |
|  | 2676 | 1 | .0 | .0 | 99.1 |
|  | 2677 | 1 | .0 | .0 | 99.1 |
|  | 2678 | 1 | .0 | .0 | 99.1 |
|  | 2681 | 2 | .0 | .0 | 99.1 |
|  | 2682 | 1 | .0 | .0 | 99.1 |
|  | 2685 | 1 | .0 | .0 | 99.1 |
|  | 2690 | 1 | .0 | .0 | 99.1 |
|  | 2691 | 1 | .0 | .0 | 99.1 |
|  | 2692 | 2 | .0 | .0 | 99.1 |
|  | 2695 | 1 | .0 | .0 | 99.1 |
|  | 2697 | 1 | .0 | .0 | 99.1 |
|  | 2701 | 1 | .0 | .0 | 99.1 |
|  | 2704 | 1 | .0 | .0 | 99.1 |
|  | 2705 | 2 | .0 | .0 | 99.1 |
|  | 2706 | 1 | .0 | .0 | 99.1 |
|  | 2707 | 1 | .0 | .0 | 99.1 |
|  | 2709 | 1 | .0 | .0 | 99.2 |
|  | 2710 | 1 | .0 | .0 | 99.2 |
|  | 2712 | 1 | .0 | .0 | 99.2 |
|  | 2717 | 2 | .0 | .0 | 99.2 |
|  | 2718 | 1 | .0 | .0 | 99.2 |
|  | 2725 | 1 | .0 | .0 | 99.2 |
|  | 2730 | 1 | .0 | .0 | 99.2 |
|  | 2731 | 1 | .0 | .0 | 99.2 |
|  | 2736 | 1 | .0 | .0 | 99.2 |
|  | 2740 | 1 | .0 | .0 | 99.2 |
|  | 2741 | 1 | .0 | .0 | 99.2 |
|  | 2746 | 1 | .0 | .0 | 99.2 |
|  | 2749 | 1 | .0 | .0 | 99.2 |
|  | 2752 | 1 | .0 | .0 | 99.2 |
|  | 2755 | 1 | .0 | .0 | 99.2 |
|  | 2757 | 1 | .0 | .0 | 99.2 |
|  | 2760 | 1 | .0 | .0 | 99.2 |
|  | 2765 | 1 | .0 | .0 | 99.2 |
|  | 2767 | 1 | .0 | .0 | 99.2 |
|  | 2769 | 1 | .0 | .0 | 99.2 |
|  | 2771 | 1 | .0 | .0 | 99.2 |
|  | 2772 | 2 | .0 | .0 | 99.2 |
|  | 2777 | 1 | .0 | .0 | 99.2 |
|  | 2782 | 1 | .0 | .0 | 99.2 |
|  | 2784 | 2 | .0 | .0 | 99.2 |
|  | 2785 | 1 | .0 | .0 | 99.2 |
|  | 2786 | 1 | .0 | .0 | 99.2 |
|  | 2790 | 1 | .0 | .0 | 99.3 |
|  | 2794 | 2 | .0 | .0 | 99.3 |
|  | 2796 | 1 | .0 | .0 | 99.3 |
|  | 2797 | 1 | .0 | .0 | 99.3 |
|  | 2800 | 1 | .0 | .0 | 99.3 |
|  | 2802 | 1 | .0 | .0 | 99.3 |
|  | 2806 | 1 | .0 | .0 | 99.3 |
|  | 2809 | 1 | .0 | .0 | 99.3 |
|  | 2810 | 1 | .0 | .0 | 99.3 |
|  | 2814 | 1 | .0 | .0 | 99.3 |
|  | 2815 | 1 | .0 | .0 | 99.3 |
|  | 2817 | 1 | .0 | .0 | 99.3 |
|  | 2818 | 1 | .0 | .0 | 99.3 |
|  | 2823 | 1 | .0 | .0 | 99.3 |
|  | 2824 | 2 | .0 | .0 | 99.3 |
|  | 2825 | 3 | .0 | .0 | 99.3 |
|  | 2828 | 1 | .0 | .0 | 99.3 |
|  | 2831 | 1 | .0 | .0 | 99.3 |
|  | 2832 | 1 | .0 | .0 | 99.3 |
|  | 2836 | 2 | .0 | .0 | 99.3 |
|  | 2837 | 2 | .0 | .0 | 99.3 |
|  | 2839 | 1 | .0 | .0 | 99.3 |
|  | 2845 | 1 | .0 | .0 | 99.3 |
|  | 2846 | 1 | .0 | .0 | 99.3 |
|  | 2847 | 1 | .0 | .0 | 99.3 |
|  | 2853 | 1 | .0 | .0 | 99.4 |
|  | 2860 | 1 | .0 | .0 | 99.4 |
|  | 2861 | 1 | .0 | .0 | 99.4 |
|  | 2864 | 1 | .0 | .0 | 99.4 |
|  | 2872 | 1 | .0 | .0 | 99.4 |
|  | 2873 | 1 | .0 | .0 | 99.4 |
|  | 2878 | 1 | .0 | .0 | 99.4 |
|  | 2885 | 1 | .0 | .0 | 99.4 |
|  | 2886 | 1 | .0 | .0 | 99.4 |
|  | 2888 | 3 | .0 | .0 | 99.4 |
|  | 2889 | 1 | .0 | .0 | 99.4 |
|  | 2896 | 1 | .0 | .0 | 99.4 |
|  | 2903 | 1 | .0 | .0 | 99.4 |
|  | 2913 | 2 | .0 | .0 | 99.4 |
|  | 2915 | 1 | .0 | .0 | 99.4 |
|  | 2918 | 1 | .0 | .0 | 99.4 |
|  | 2924 | 2 | .0 | .0 | 99.4 |
|  | 2925 | 1 | .0 | .0 | 99.4 |
|  | 2940 | 1 | .0 | .0 | 99.4 |
|  | 2942 | 2 | .0 | .0 | 99.4 |
|  | 2944 | 1 | .0 | .0 | 99.4 |
|  | 2949 | 2 | .0 | .0 | 99.4 |
|  | 2951 | 1 | .0 | .0 | 99.4 |
|  | 2956 | 1 | .0 | .0 | 99.4 |
|  | 2957 | 1 | .0 | .0 | 99.5 |
|  | 2961 | 1 | .0 | .0 | 99.5 |
|  | 2962 | 1 | .0 | .0 | 99.5 |
|  | 2965 | 1 | .0 | .0 | 99.5 |
|  | 2968 | 2 | .0 | .0 | 99.5 |
|  | 2970 | 1 | .0 | .0 | 99.5 |
|  | 2971 | 1 | .0 | .0 | 99.5 |
|  | 2972 | 1 | .0 | .0 | 99.5 |
|  | 2974 | 1 | .0 | .0 | 99.5 |
|  | 2975 | 1 | .0 | .0 | 99.5 |
|  | 2979 | 1 | .0 | .0 | 99.5 |
|  | 2980 | 1 | .0 | .0 | 99.5 |
|  | 2981 | 1 | .0 | .0 | 99.5 |
|  | 2984 | 1 | .0 | .0 | 99.5 |
|  | 2989 | 1 | .0 | .0 | 99.5 |
|  | 2990 | 2 | .0 | .0 | 99.5 |
|  | 3005 | 1 | .0 | .0 | 99.5 |
|  | 3013 | 1 | .0 | .0 | 99.5 |
|  | 3018 | 1 | .0 | .0 | 99.5 |
|  | 3022 | 1 | .0 | .0 | 99.5 |
|  | 3024 | 2 | .0 | .0 | 99.5 |
|  | 3027 | 1 | .0 | .0 | 99.5 |
|  | 3030 | 1 | .0 | .0 | 99.5 |
|  | 3036 | 1 | .0 | .0 | 99.5 |
|  | 3054 | 1 | .0 | .0 | 99.5 |
|  | 3059 | 2 | .0 | .0 | 99.5 |
|  | 3063 | 1 | .0 | .0 | 99.5 |
|  | 3065 | 1 | .0 | .0 | 99.6 |
|  | 3066 | 1 | .0 | .0 | 99.6 |
|  | 3071 | 1 | .0 | .0 | 99.6 |
|  | 3073 | 1 | .0 | .0 | 99.6 |
|  | 3076 | 1 | .0 | .0 | 99.6 |
|  | 3077 | 1 | .0 | .0 | 99.6 |
|  | 3079 | 1 | .0 | .0 | 99.6 |
|  | 3083 | 1 | .0 | .0 | 99.6 |
|  | 3085 | 1 | .0 | .0 | 99.6 |
|  | 3086 | 2 | .0 | .0 | 99.6 |
|  | 3090 | 1 | .0 | .0 | 99.6 |
|  | 3092 | 1 | .0 | .0 | 99.6 |
|  | 3104 | 1 | .0 | .0 | 99.6 |
|  | 3109 | 1 | .0 | .0 | 99.6 |
|  | 3112 | 1 | .0 | .0 | 99.6 |
|  | 3116 | 1 | .0 | .0 | 99.6 |
|  | 3117 | 1 | .0 | .0 | 99.6 |
|  | 3120 | 1 | .0 | .0 | 99.6 |
|  | 3121 | 1 | .0 | .0 | 99.6 |
|  | 3123 | 1 | .0 | .0 | 99.6 |
|  | 3124 | 1 | .0 | .0 | 99.6 |
|  | 3125 | 1 | .0 | .0 | 99.6 |
|  | 3131 | 1 | .0 | .0 | 99.6 |
|  | 3134 | 1 | .0 | .0 | 99.6 |
|  | 3136 | 1 | .0 | .0 | 99.6 |
|  | 3142 | 1 | .0 | .0 | 99.6 |
|  | 3170 | 1 | .0 | .0 | 99.6 |
|  | 3261 | 1 | .0 | .0 | 99.6 |
|  | 3266 | 1 | .0 | .0 | 99.6 |
|  | 3270 | 1 | .0 | .0 | 99.6 |
|  | 3272 | 1 | .0 | .0 | 99.7 |
|  | 3274 | 1 | .0 | .0 | 99.7 |
|  | 3277 | 1 | .0 | .0 | 99.7 |
|  | 3279 | 1 | .0 | .0 | 99.7 |
|  | 3289 | 1 | .0 | .0 | 99.7 |
|  | 3293 | 1 | .0 | .0 | 99.7 |
|  | 3310 | 1 | .0 | .0 | 99.7 |
|  | 3315 | 1 | .0 | .0 | 99.7 |
|  | 3335 | 1 | .0 | .0 | 99.7 |
|  | 3344 | 1 | .0 | .0 | 99.7 |
|  | 3350 | 2 | .0 | .0 | 99.7 |
|  | 3354 | 1 | .0 | .0 | 99.7 |
|  | 3355 | 1 | .0 | .0 | 99.7 |
|  | 3358 | 1 | .0 | .0 | 99.7 |
|  | 3359 | 1 | .0 | .0 | 99.7 |
|  | 3368 | 1 | .0 | .0 | 99.7 |
|  | 3370 | 1 | .0 | .0 | 99.7 |
|  | 3371 | 1 | .0 | .0 | 99.7 |
|  | 3382 | 1 | .0 | .0 | 99.7 |
|  | 3386 | 2 | .0 | .0 | 99.7 |
|  | 3387 | 1 | .0 | .0 | 99.7 |
|  | 3390 | 2 | .0 | .0 | 99.7 |
|  | 3392 | 1 | .0 | .0 | 99.7 |
|  | 3393 | 1 | .0 | .0 | 99.7 |
|  | 3424 | 1 | .0 | .0 | 99.7 |
|  | 3430 | 1 | .0 | .0 | 99.7 |
|  | 3446 | 1 | .0 | .0 | 99.7 |
|  | 3447 | 1 | .0 | .0 | 99.8 |
|  | 3454 | 1 | .0 | .0 | 99.8 |
|  | 3457 | 1 | .0 | .0 | 99.8 |
|  | 3458 | 2 | .0 | .0 | 99.8 |
|  | 3470 | 1 | .0 | .0 | 99.8 |
|  | 3494 | 1 | .0 | .0 | 99.8 |
|  | 3495 | 1 | .0 | .0 | 99.8 |
|  | 3497 | 1 | .0 | .0 | 99.8 |
|  | 3498 | 1 | .0 | .0 | 99.8 |
|  | 3503 | 1 | .0 | .0 | 99.8 |
|  | 3509 | 1 | .0 | .0 | 99.8 |
|  | 3510 | 1 | .0 | .0 | 99.8 |
|  | 3511 | 1 | .0 | .0 | 99.8 |
|  | 3520 | 1 | .0 | .0 | 99.8 |
|  | 3525 | 1 | .0 | .0 | 99.8 |
|  | 3528 | 1 | .0 | .0 | 99.8 |
|  | 3542 | 1 | .0 | .0 | 99.8 |
|  | 3555 | 1 | .0 | .0 | 99.8 |
|  | 3557 | 1 | .0 | .0 | 99.8 |
|  | 3561 | 1 | .0 | .0 | 99.8 |
|  | 3563 | 2 | .0 | .0 | 99.8 |
|  | 3566 | 1 | .0 | .0 | 99.8 |
|  | 3567 | 2 | .0 | .0 | 99.8 |
|  | 3570 | 1 | .0 | .0 | 99.8 |
|  | 3578 | 3 | .0 | .0 | 99.8 |
|  | 3579 | 1 | .0 | .0 | 99.8 |
|  | 3587 | 1 | .0 | .0 | 99.9 |
|  | 3591 | 1 | .0 | .0 | 99.9 |
|  | 3595 | 1 | .0 | .0 | 99.9 |
|  | 3596 | 1 | .0 | .0 | 99.9 |
|  | 3598 | 1 | .0 | .0 | 99.9 |
|  | 3600 | 1 | .0 | .0 | 99.9 |
|  | 3612 | 1 | .0 | .0 | 99.9 |
|  | 3613 | 1 | .0 | .0 | 99.9 |
|  | 3626 | 1 | .0 | .0 | 99.9 |
|  | 3631 | 1 | .0 | .0 | 99.9 |
|  | 3635 | 1 | .0 | .0 | 99.9 |
|  | 3642 | 1 | .0 | .0 | 99.9 |
|  | 3644 | 1 | .0 | .0 | 99.9 |
|  | 3657 | 1 | .0 | .0 | 99.9 |
|  | 3663 | 1 | .0 | .0 | 99.9 |
|  | 3671 | 2 | .0 | .0 | 99.9 |
|  | 3673 | 1 | .0 | .0 | 99.9 |
|  | 3681 | 1 | .0 | .0 | 99.9 |
|  | 3684 | 1 | .0 | .0 | 99.9 |
|  | 3686 | 1 | .0 | .0 | 99.9 |
|  | 3690 | 1 | .0 | .0 | 99.9 |
|  | 3693 | 1 | .0 | .0 | 99.9 |
|  | 3697 | 1 | .0 | .0 | 99.9 |
|  | 3700 | 1 | .0 | .0 | 99.9 |
|  | 3701 | 1 | .0 | .0 | 99.9 |
|  | 3708 | 1 | .0 | .0 | 99.9 |
|  | 3716 | 1 | .0 | .0 | 99.9 |
|  | 3717 | 1 | .0 | .0 | 99.9 |
|  | 3718 | 1 | .0 | .0 | 99.9 |
|  | 3719 | 1 | .0 | .0 | 100.0 |
|  | 3720 | 1 | .0 | .0 | 100.0 |
|  | 3721 | 1 | .0 | .0 | 100.0 |
|  | 3725 | 1 | .0 | .0 | 100.0 |
|  | 3727 | 1 | .0 | .0 | 100.0 |
|  | 3729 | 1 | .0 | .0 | 100.0 |
|  | 3742 | 1 | .0 | .0 | 100.0 |
|  | 3753 | 1 | .0 | .0 | 100.0 |
|  | 3755 | 2 | .0 | .0 | 100.0 |
|  | 3756 | 1 | .0 | .0 | 100.0 |
|  | 3757 | 1 | .0 | .0 | 100.0 |
|  | 3760 | 1 | .0 | .0 | 100.0 |
|  | 3761 | 1 | .0 | .0 | 100.0 |
|  | 3762 | 1 | .0 | .0 | 100.0 |
|  | 3770 | 1 | .0 | .0 | 100.0 |
|  | Total | 30553 | 100.0 | 100.0 |  |
|  |  |  |  |  |  |

Interviewer codeInterviewer code, table, 1 levels of column headers and 2 levels of row headers, table with 6 columns and 110 rows

|  |  |  |  |  |  |
| --- | --- | --- | --- | --- | --- |
|  | | Frequency | Percent | Valid Percent | Cumulative Percent |
| Valid | 400 | 1 | .0 | .0 | .0 |
|  | 401 | 1070 | 3.5 | 3.5 | 3.5 |
|  | 402 | 1130 | 3.7 | 3.7 | 7.2 |
|  | 403 | 775 | 2.5 | 2.5 | 9.7 |
|  | 404 | 630 | 2.1 | 2.1 | 11.8 |
|  | 405 | 467 | 1.5 | 1.5 | 13.3 |
|  | 406 | 329 | 1.1 | 1.1 | 14.4 |
|  | 407 | 304 | 1.0 | 1.0 | 15.4 |
|  | 408 | 294 | 1.0 | 1.0 | 16.4 |
|  | 409 | 200 | .7 | .7 | 17.0 |
|  | 410 | 177 | .6 | .6 | 17.6 |
|  | 411 | 109 | .4 | .4 | 18.0 |
|  | 412 | 116 | .4 | .4 | 18.3 |
|  | 413 | 64 | .2 | .2 | 18.5 |
|  | 414 | 34 | .1 | .1 | 18.7 |
|  | 415 | 28 | .1 | .1 | 18.7 |
|  | 416 | 13 | .0 | .0 | 18.8 |
|  | 417 | 17 | .1 | .1 | 18.8 |
|  | 418 | 12 | .0 | .0 | 18.9 |
|  | 419 | 9 | .0 | .0 | 18.9 |
|  | 420 | 2 | .0 | .0 | 18.9 |
|  | 421 | 7 | .0 | .0 | 18.9 |
|  | 500 | 1 | .0 | .0 | 18.9 |
|  | 501 | 1864 | 6.1 | 6.1 | 25.0 |
|  | 502 | 1814 | 5.9 | 5.9 | 31.0 |
|  | 503 | 1583 | 5.2 | 5.2 | 36.2 |
|  | 504 | 1325 | 4.3 | 4.3 | 40.5 |
|  | 505 | 1070 | 3.5 | 3.5 | 44.0 |
|  | 506 | 919 | 3.0 | 3.0 | 47.0 |
|  | 507 | 735 | 2.4 | 2.4 | 49.4 |
|  | 508 | 664 | 2.2 | 2.2 | 51.6 |
|  | 509 | 627 | 2.1 | 2.1 | 53.6 |
|  | 510 | 552 | 1.8 | 1.8 | 55.5 |
|  | 511 | 537 | 1.8 | 1.8 | 57.2 |
|  | 512 | 439 | 1.4 | 1.4 | 58.6 |
|  | 513 | 367 | 1.2 | 1.2 | 59.8 |
|  | 514 | 221 | .7 | .7 | 60.6 |
|  | 515 | 204 | .7 | .7 | 61.2 |
|  | 516 | 139 | .5 | .5 | 61.7 |
|  | 517 | 117 | .4 | .4 | 62.1 |
|  | 518 | 100 | .3 | .3 | 62.4 |
|  | 519 | 84 | .3 | .3 | 62.7 |
|  | 520 | 80 | .3 | .3 | 62.9 |
|  | 521 | 53 | .2 | .2 | 63.1 |
|  | 522 | 37 | .1 | .1 | 63.2 |
|  | 523 | 41 | .1 | .1 | 63.4 |
|  | 524 | 33 | .1 | .1 | 63.5 |
|  | 525 | 32 | .1 | .1 | 63.6 |
|  | 526 | 45 | .1 | .1 | 63.7 |
|  | 527 | 44 | .1 | .1 | 63.9 |
|  | 528 | 12 | .0 | .0 | 63.9 |
|  | 529 | 12 | .0 | .0 | 64.0 |
|  | 530 | 19 | .1 | .1 | 64.0 |
|  | 531 | 10 | .0 | .0 | 64.0 |
|  | 532 | 18 | .1 | .1 | 64.1 |
|  | 533 | 18 | .1 | .1 | 64.2 |
|  | 534 | 14 | .0 | .0 | 64.2 |
|  | 535 | 16 | .1 | .1 | 64.3 |
|  | 536 | 15 | .0 | .0 | 64.3 |
|  | 537 | 21 | .1 | .1 | 64.4 |
|  | 538 | 12 | .0 | .0 | 64.4 |
|  | 539 | 23 | .1 | .1 | 64.5 |
|  | 540 | 30 | .1 | .1 | 64.6 |
|  | 601 | 2067 | 6.8 | 6.8 | 71.4 |
|  | 602 | 1635 | 5.4 | 5.4 | 76.7 |
|  | 603 | 1205 | 3.9 | 3.9 | 80.7 |
|  | 604 | 787 | 2.6 | 2.6 | 83.2 |
|  | 605 | 833 | 2.7 | 2.7 | 86.0 |
|  | 606 | 557 | 1.8 | 1.8 | 87.8 |
|  | 607 | 427 | 1.4 | 1.4 | 89.2 |
|  | 608 | 312 | 1.0 | 1.0 | 90.2 |
|  | 609 | 539 | 1.8 | 1.8 | 92.0 |
|  | 610 | 388 | 1.3 | 1.3 | 93.2 |
|  | 611 | 315 | 1.0 | 1.0 | 94.3 |
|  | 612 | 280 | .9 | .9 | 95.2 |
|  | 613 | 107 | .4 | .4 | 95.5 |
|  | 614 | 108 | .4 | .4 | 95.9 |
|  | 615 | 72 | .2 | .2 | 96.1 |
|  | 616 | 81 | .3 | .3 | 96.4 |
|  | 617 | 77 | .3 | .3 | 96.6 |
|  | 618 | 104 | .3 | .3 | 97.0 |
|  | 619 | 83 | .3 | .3 | 97.2 |
|  | 620 | 152 | .5 | .5 | 97.7 |
|  | 621 | 54 | .2 | .2 | 97.9 |
|  | 622 | 73 | .2 | .2 | 98.2 |
|  | 623 | 35 | .1 | .1 | 98.3 |
|  | 624 | 38 | .1 | .1 | 98.4 |
|  | 625 | 11 | .0 | .0 | 98.4 |
|  | 626 | 16 | .1 | .1 | 98.5 |
|  | 627 | 15 | .0 | .0 | 98.5 |
|  | 628 | 21 | .1 | .1 | 98.6 |
|  | 629 | 7 | .0 | .0 | 98.6 |
|  | 630 | 15 | .0 | .0 | 98.7 |
|  | 631 | 11 | .0 | .0 | 98.7 |
|  | 632 | 13 | .0 | .0 | 98.8 |
|  | 633 | 15 | .0 | .0 | 98.8 |
|  | 634 | 10 | .0 | .0 | 98.8 |
|  | 635 | 16 | .1 | .1 | 98.9 |
|  | 636 | 20 | .1 | .1 | 99.0 |
|  | 637 | 25 | .1 | .1 | 99.0 |
|  | 638 | 15 | .0 | .0 | 99.1 |
|  | 639 | 13 | .0 | .0 | 99.1 |
|  | 640 | 27 | .1 | .1 | 99.2 |
|  | 641 | 7 | .0 | .0 | 99.2 |
|  | 660 | 178 | .6 | .6 | 99.8 |
|  | 661 | 3 | .0 | .0 | 99.8 |
|  | 690 | 51 | .2 | .2 | 100.0 |
|  | Total | 30553 | 100.0 | 100.0 |  |
|  |  |  |  |  |  |

Interview result codeInterview result code, table, 1 levels of column headers and 2 levels of row headers, table with 6 columns and 3 rows

|  |  |  |  |  |  |
| --- | --- | --- | --- | --- | --- |
|  | | Frequency | Percent | Valid Percent | Cumulative Percent |
| Valid | Interview completed | 30553 | 100.0 | 100.0 | 100.0 |
|  |  |  |  |  |  |

Serial # of HH membersSerial # of HH members, table, 1 levels of column headers and 2 levels of row headers, table with 6 columns and 22 rows

|  |  |  |  |  |  |
| --- | --- | --- | --- | --- | --- |
|  | | Frequency | Percent | Valid Percent | Cumulative Percent |
| Valid | 2 | 125 | .4 | .4 | .4 |
|  | 3 | 5276 | 17.3 | 17.3 | 17.7 |
|  | 4 | 7990 | 26.2 | 26.2 | 43.8 |
|  | 5 | 6842 | 22.4 | 22.4 | 66.2 |
|  | 6 | 4580 | 15.0 | 15.0 | 81.2 |
|  | 7 | 2564 | 8.4 | 8.4 | 89.6 |
|  | 8 | 1458 | 4.8 | 4.8 | 94.4 |
|  | 9 | 736 | 2.4 | 2.4 | 96.8 |
|  | 10 | 400 | 1.3 | 1.3 | 98.1 |
|  | 11 | 263 | .9 | .9 | 99.0 |
|  | 12 | 134 | .4 | .4 | 99.4 |
|  | 13 | 89 | .3 | .3 | 99.7 |
|  | 14 | 43 | .1 | .1 | 99.8 |
|  | 15 | 23 | .1 | .1 | 99.9 |
|  | 16 | 15 | .0 | .0 | 100.0 |
|  | 17 | 5 | .0 | .0 | 100.0 |
|  | 18 | 5 | .0 | .0 | 100.0 |
|  | 19 | 3 | .0 | .0 | 100.0 |
|  | 21 | 2 | .0 | .0 | 100.0 |
|  | Total | 30553 | 100.0 | 100.0 |  |
|  |  |  |  |  |  |

Caregiver's serial numberCaregiver's serial number, table, 1 levels of column headers and 2 levels of row headers, table with 6 columns and 18 rows

|  |  |  |  |  |  |
| --- | --- | --- | --- | --- | --- |
|  | | Frequency | Percent | Valid Percent | Cumulative Percent |
| Valid | 0 | 1 | .0 | 3.3 | 3.3 |
|  | 1 | 3 | .0 | 10.0 | 13.3 |
|  | 2 | 15 | .0 | 50.0 | 63.3 |
|  | 3 | 1 | .0 | 3.3 | 66.7 |
|  | 4 | 2 | .0 | 6.7 | 73.3 |
|  | 5 | 1 | .0 | 3.3 | 76.7 |
|  | 8 | 1 | .0 | 3.3 | 80.0 |
|  | 15 | 1 | .0 | 3.3 | 83.3 |
|  | 18 | 1 | .0 | 3.3 | 86.7 |
|  | 25 | 1 | .0 | 3.3 | 90.0 |
|  | 27 | 1 | .0 | 3.3 | 93.3 |
|  | 30 | 1 | .0 | 3.3 | 96.7 |
|  | 60 | 1 | .0 | 3.3 | 100.0 |
|  | Total | 30 | .1 | 100.0 |  |
| Missing | System | 30523 | 99.9 |  |  |
| Total | | 30553 | 100.0 |  |  |
|  |  |  |  |  |  |

Visit numberVisit number, table, 1 levels of column headers and 2 levels of row headers, table with 6 columns and 3 rows

|  |  |  |  |  |  |
| --- | --- | --- | --- | --- | --- |
|  | | Frequency | Percent | Valid Percent | Cumulative Percent |
| Valid | 1 | 30553 | 100.0 | 100.0 | 100.0 |
|  |  |  |  |  |  |

Visit dateVisit date, table, 1 levels of column headers and 2 levels of row headers, table with 6 columns and 546 rows

|  |  |  |  |  |  |
| --- | --- | --- | --- | --- | --- |
|  | | Frequency | Percent | Valid Percent | Cumulative Percent |
| Valid | 30-MAY-2014 | 87 | .3 | .3 | .3 |
|  | 31-MAY-2014 | 239 | .8 | .8 | 1.1 |
|  | 01-JUN-2014 | 413 | 1.4 | 1.4 | 2.4 |
|  | 02-JUN-2014 | 345 | 1.1 | 1.1 | 3.5 |
|  | 03-JUN-2014 | 341 | 1.1 | 1.1 | 4.7 |
|  | 04-JUN-2014 | 215 | .7 | .7 | 5.4 |
|  | 05-JUN-2014 | 285 | .9 | .9 | 6.3 |
|  | 06-JUN-2014 | 52 | .2 | .2 | 6.5 |
|  | 07-JUN-2014 | 213 | .7 | .7 | 7.2 |
|  | 08-JUN-2014 | 170 | .6 | .6 | 7.7 |
|  | 09-JUN-2014 | 138 | .5 | .5 | 8.2 |
|  | 10-JUN-2014 | 106 | .3 | .3 | 8.5 |
|  | 11-JUN-2014 | 88 | .3 | .3 | 8.8 |
|  | 12-JUN-2014 | 76 | .2 | .2 | 9.1 |
|  | 13-JUN-2014 | 19 | .1 | .1 | 9.1 |
|  | 14-JUN-2014 | 18 | .1 | .1 | 9.2 |
|  | 15-JUN-2014 | 136 | .4 | .4 | 9.6 |
|  | 16-JUN-2014 | 121 | .4 | .4 | 10.0 |
|  | 17-JUN-2014 | 134 | .4 | .4 | 10.5 |
|  | 18-JUN-2014 | 87 | .3 | .3 | 10.7 |
|  | 19-JUN-2014 | 18 | .1 | .1 | 10.8 |
|  | 20-JUN-2014 | 21 | .1 | .1 | 10.9 |
|  | 21-JUN-2014 | 81 | .3 | .3 | 11.1 |
|  | 22-JUN-2014 | 119 | .4 | .4 | 11.5 |
|  | 23-JUN-2014 | 113 | .4 | .4 | 11.9 |
|  | 24-JUN-2014 | 65 | .2 | .2 | 12.1 |
|  | 25-JUN-2014 | 109 | .4 | .4 | 12.5 |
|  | 26-JUN-2014 | 171 | .6 | .6 | 13.0 |
|  | 27-JUN-2014 | 17 | .1 | .1 | 13.1 |
|  | 28-JUN-2014 | 103 | .3 | .3 | 13.4 |
|  | 29-JUN-2014 | 118 | .4 | .4 | 13.8 |
|  | 30-JUN-2014 | 94 | .3 | .3 | 14.1 |
|  | 01-JUL-2014 | 172 | .6 | .6 | 14.7 |
|  | 02-JUL-2014 | 147 | .5 | .5 | 15.2 |
|  | 03-JUL-2014 | 116 | .4 | .4 | 15.5 |
|  | 04-JUL-2014 | 26 | .1 | .1 | 15.6 |
|  | 05-JUL-2014 | 88 | .3 | .3 | 15.9 |
|  | 06-JUL-2014 | 150 | .5 | .5 | 16.4 |
|  | 07-JUL-2014 | 129 | .4 | .4 | 16.8 |
|  | 08-JUL-2014 | 108 | .4 | .4 | 17.2 |
|  | 09-JUL-2014 | 77 | .3 | .3 | 17.4 |
|  | 10-JUL-2014 | 126 | .4 | .4 | 17.8 |
|  | 11-JUL-2014 | 19 | .1 | .1 | 17.9 |
|  | 12-JUL-2014 | 139 | .5 | .5 | 18.4 |
|  | 13-JUL-2014 | 269 | .9 | .9 | 19.2 |
|  | 14-JUL-2014 | 122 | .4 | .4 | 19.6 |
|  | 15-JUL-2014 | 142 | .5 | .5 | 20.1 |
|  | 16-JUL-2014 | 164 | .5 | .5 | 20.6 |
|  | 17-JUL-2014 | 146 | .5 | .5 | 21.1 |
|  | 18-JUL-2014 | 35 | .1 | .1 | 21.2 |
|  | 19-JUL-2014 | 117 | .4 | .4 | 21.6 |
|  | 20-JUL-2014 | 159 | .5 | .5 | 22.1 |
|  | 21-JUL-2014 | 108 | .4 | .4 | 22.5 |
|  | 22-JUL-2014 | 162 | .5 | .5 | 23.0 |
|  | 23-JUL-2014 | 126 | .4 | .4 | 23.4 |
|  | 24-JUL-2014 | 81 | .3 | .3 | 23.7 |
|  | 25-JUL-2014 | 36 | .1 | .1 | 23.8 |
|  | 26-JUL-2014 | 40 | .1 | .1 | 23.9 |
|  | 27-JUL-2014 | 74 | .2 | .2 | 24.2 |
|  | 28-JUL-2014 | 48 | .2 | .2 | 24.3 |
|  | 29-JUL-2014 | 18 | .1 | .1 | 24.4 |
|  | 30-JUL-2014 | 3 | .0 | .0 | 24.4 |
|  | 31-JUL-2014 | 32 | .1 | .1 | 24.5 |
|  | 01-AUG-2014 | 21 | .1 | .1 | 24.6 |
|  | 02-AUG-2014 | 192 | .6 | .6 | 25.2 |
|  | 03-AUG-2014 | 53 | .2 | .2 | 25.4 |
|  | 04-AUG-2014 | 32 | .1 | .1 | 25.5 |
|  | 05-AUG-2014 | 118 | .4 | .4 | 25.9 |
|  | 06-AUG-2014 | 93 | .3 | .3 | 26.2 |
|  | 07-AUG-2014 | 91 | .3 | .3 | 26.5 |
|  | 08-AUG-2014 | 60 | .2 | .2 | 26.7 |
|  | 09-AUG-2014 | 144 | .5 | .5 | 27.1 |
|  | 10-AUG-2014 | 125 | .4 | .4 | 27.6 |
|  | 11-AUG-2014 | 113 | .4 | .4 | 27.9 |
|  | 12-AUG-2014 | 72 | .2 | .2 | 28.2 |
|  | 13-AUG-2014 | 154 | .5 | .5 | 28.7 |
|  | 14-AUG-2014 | 67 | .2 | .2 | 28.9 |
|  | 15-AUG-2014 | 9 | .0 | .0 | 28.9 |
|  | 16-AUG-2014 | 103 | .3 | .3 | 29.3 |
|  | 17-AUG-2014 | 23 | .1 | .1 | 29.3 |
|  | 18-AUG-2014 | 81 | .3 | .3 | 29.6 |
|  | 19-AUG-2014 | 98 | .3 | .3 | 29.9 |
|  | 20-AUG-2014 | 69 | .2 | .2 | 30.1 |
|  | 21-AUG-2014 | 154 | .5 | .5 | 30.6 |
|  | 22-AUG-2014 | 39 | .1 | .1 | 30.8 |
|  | 23-AUG-2014 | 122 | .4 | .4 | 31.2 |
|  | 24-AUG-2014 | 92 | .3 | .3 | 31.5 |
|  | 25-AUG-2014 | 152 | .5 | .5 | 32.0 |
|  | 26-AUG-2014 | 112 | .4 | .4 | 32.3 |
|  | 27-AUG-2014 | 41 | .1 | .1 | 32.5 |
|  | 28-AUG-2014 | 209 | .7 | .7 | 33.2 |
|  | 29-AUG-2014 | 43 | .1 | .1 | 33.3 |
|  | 30-AUG-2014 | 162 | .5 | .5 | 33.8 |
|  | 31-AUG-2014 | 52 | .2 | .2 | 34.0 |
|  | 01-SEP-2014 | 71 | .2 | .2 | 34.2 |
|  | 02-SEP-2014 | 81 | .3 | .3 | 34.5 |
|  | 03-SEP-2014 | 77 | .3 | .3 | 34.7 |
|  | 04-SEP-2014 | 119 | .4 | .4 | 35.1 |
|  | 05-SEP-2014 | 23 | .1 | .1 | 35.2 |
|  | 06-SEP-2014 | 157 | .5 | .5 | 35.7 |
|  | 07-SEP-2014 | 58 | .2 | .2 | 35.9 |
|  | 08-SEP-2014 | 130 | .4 | .4 | 36.3 |
|  | 09-SEP-2014 | 139 | .5 | .5 | 36.8 |
|  | 10-SEP-2014 | 105 | .3 | .3 | 37.1 |
|  | 11-SEP-2014 | 140 | .5 | .5 | 37.6 |
|  | 12-SEP-2014 | 15 | .0 | .0 | 37.6 |
|  | 13-SEP-2014 | 139 | .5 | .5 | 38.1 |
|  | 14-SEP-2014 | 111 | .4 | .4 | 38.5 |
|  | 15-SEP-2014 | 107 | .4 | .4 | 38.8 |
|  | 16-SEP-2014 | 144 | .5 | .5 | 39.3 |
|  | 17-SEP-2014 | 133 | .4 | .4 | 39.7 |
|  | 18-SEP-2014 | 34 | .1 | .1 | 39.8 |
|  | 19-SEP-2014 | 38 | .1 | .1 | 40.0 |
|  | 20-SEP-2014 | 88 | .3 | .3 | 40.2 |
|  | 21-SEP-2014 | 90 | .3 | .3 | 40.5 |
|  | 22-SEP-2014 | 108 | .4 | .4 | 40.9 |
|  | 23-SEP-2014 | 51 | .2 | .2 | 41.1 |
|  | 24-SEP-2014 | 70 | .2 | .2 | 41.3 |
|  | 25-SEP-2014 | 55 | .2 | .2 | 41.5 |
|  | 26-SEP-2014 | 30 | .1 | .1 | 41.6 |
|  | 27-SEP-2014 | 102 | .3 | .3 | 41.9 |
|  | 28-SEP-2014 | 60 | .2 | .2 | 42.1 |
|  | 29-SEP-2014 | 107 | .4 | .4 | 42.4 |
|  | 30-SEP-2014 | 117 | .4 | .4 | 42.8 |
|  | 01-OCT-2014 | 131 | .4 | .4 | 43.3 |
|  | 02-OCT-2014 | 107 | .4 | .4 | 43.6 |
|  | 03-OCT-2014 | 41 | .1 | .1 | 43.7 |
|  | 04-OCT-2014 | 19 | .1 | .1 | 43.8 |
|  | 05-OCT-2014 | 41 | .1 | .1 | 43.9 |
|  | 06-OCT-2014 | 18 | .1 | .1 | 44.0 |
|  | 07-OCT-2014 | 21 | .1 | .1 | 44.1 |
|  | 08-OCT-2014 | 267 | .9 | .9 | 44.9 |
|  | 09-OCT-2014 | 46 | .2 | .2 | 45.1 |
|  | 10-OCT-2014 | 33 | .1 | .1 | 45.2 |
|  | 11-OCT-2014 | 59 | .2 | .2 | 45.4 |
|  | 12-OCT-2014 | 57 | .2 | .2 | 45.6 |
|  | 13-OCT-2014 | 128 | .4 | .4 | 46.0 |
|  | 14-OCT-2014 | 79 | .3 | .3 | 46.3 |
|  | 15-OCT-2014 | 96 | .3 | .3 | 46.6 |
|  | 16-OCT-2014 | 103 | .3 | .3 | 46.9 |
|  | 17-OCT-2014 | 23 | .1 | .1 | 47.0 |
|  | 18-OCT-2014 | 163 | .5 | .5 | 47.5 |
|  | 19-OCT-2014 | 84 | .3 | .3 | 47.8 |
|  | 20-OCT-2014 | 279 | .9 | .9 | 48.7 |
|  | 21-OCT-2014 | 164 | .5 | .5 | 49.2 |
|  | 22-OCT-2014 | 143 | .5 | .5 | 49.7 |
|  | 23-OCT-2014 | 119 | .4 | .4 | 50.1 |
|  | 24-OCT-2014 | 21 | .1 | .1 | 50.2 |
|  | 25-OCT-2014 | 160 | .5 | .5 | 50.7 |
|  | 26-OCT-2014 | 101 | .3 | .3 | 51.0 |
|  | 27-OCT-2014 | 99 | .3 | .3 | 51.3 |
|  | 28-OCT-2014 | 173 | .6 | .6 | 51.9 |
|  | 29-OCT-2014 | 106 | .3 | .3 | 52.3 |
|  | 30-OCT-2014 | 83 | .3 | .3 | 52.5 |
|  | 31-OCT-2014 | 5 | .0 | .0 | 52.5 |
|  | 01-NOV-2014 | 71 | .2 | .2 | 52.8 |
|  | 02-NOV-2014 | 127 | .4 | .4 | 53.2 |
|  | 03-NOV-2014 | 121 | .4 | .4 | 53.6 |
|  | 04-NOV-2014 | 76 | .2 | .2 | 53.8 |
|  | 05-NOV-2014 | 93 | .3 | .3 | 54.1 |
|  | 06-NOV-2014 | 39 | .1 | .1 | 54.3 |
|  | 07-NOV-2014 | 27 | .1 | .1 | 54.4 |
|  | 08-NOV-2014 | 53 | .2 | .2 | 54.5 |
|  | 09-NOV-2014 | 148 | .5 | .5 | 55.0 |
|  | 10-NOV-2014 | 70 | .2 | .2 | 55.2 |
|  | 11-NOV-2014 | 111 | .4 | .4 | 55.6 |
|  | 12-NOV-2014 | 102 | .3 | .3 | 55.9 |
|  | 13-NOV-2014 | 70 | .2 | .2 | 56.2 |
|  | 14-NOV-2014 | 26 | .1 | .1 | 56.3 |
|  | 15-NOV-2014 | 128 | .4 | .4 | 56.7 |
|  | 16-NOV-2014 | 107 | .4 | .4 | 57.0 |
|  | 17-NOV-2014 | 71 | .2 | .2 | 57.3 |
|  | 18-NOV-2014 | 90 | .3 | .3 | 57.6 |
|  | 19-NOV-2014 | 48 | .2 | .2 | 57.7 |
|  | 20-NOV-2014 | 23 | .1 | .1 | 57.8 |
|  | 21-NOV-2014 | 34 | .1 | .1 | 57.9 |
|  | 22-NOV-2014 | 40 | .1 | .1 | 58.0 |
|  | 23-NOV-2014 | 56 | .2 | .2 | 58.2 |
|  | 24-NOV-2014 | 30 | .1 | .1 | 58.3 |
|  | 25-NOV-2014 | 83 | .3 | .3 | 58.6 |
|  | 26-NOV-2014 | 55 | .2 | .2 | 58.8 |
|  | 27-NOV-2014 | 27 | .1 | .1 | 58.9 |
|  | 28-NOV-2014 | 14 | .0 | .0 | 58.9 |
|  | 29-NOV-2014 | 80 | .3 | .3 | 59.2 |
|  | 30-NOV-2014 | 11 | .0 | .0 | 59.2 |
|  | 01-DEC-2014 | 80 | .3 | .3 | 59.5 |
|  | 02-DEC-2014 | 81 | .3 | .3 | 59.7 |
|  | 03-DEC-2014 | 56 | .2 | .2 | 59.9 |
|  | 04-DEC-2014 | 44 | .1 | .1 | 60.0 |
|  | 05-DEC-2014 | 28 | .1 | .1 | 60.1 |
|  | 06-DEC-2014 | 67 | .2 | .2 | 60.4 |
|  | 07-DEC-2014 | 87 | .3 | .3 | 60.6 |
|  | 08-DEC-2014 | 59 | .2 | .2 | 60.8 |
|  | 09-DEC-2014 | 38 | .1 | .1 | 61.0 |
|  | 10-DEC-2014 | 103 | .3 | .3 | 61.3 |
|  | 11-DEC-2014 | 14 | .0 | .0 | 61.3 |
|  | 12-DEC-2014 | 29 | .1 | .1 | 61.4 |
|  | 13-DEC-2014 | 70 | .2 | .2 | 61.7 |
|  | 14-DEC-2014 | 120 | .4 | .4 | 62.1 |
|  | 15-DEC-2014 | 107 | .4 | .4 | 62.4 |
|  | 16-DEC-2014 | 45 | .1 | .1 | 62.6 |
|  | 17-DEC-2014 | 86 | .3 | .3 | 62.8 |
|  | 18-DEC-2014 | 52 | .2 | .2 | 63.0 |
|  | 19-DEC-2014 | 35 | .1 | .1 | 63.1 |
|  | 20-DEC-2014 | 126 | .4 | .4 | 63.5 |
|  | 21-DEC-2014 | 104 | .3 | .3 | 63.9 |
|  | 22-DEC-2014 | 137 | .4 | .4 | 64.3 |
|  | 23-DEC-2014 | 75 | .2 | .2 | 64.6 |
|  | 24-DEC-2014 | 132 | .4 | .4 | 65.0 |
|  | 25-DEC-2014 | 48 | .2 | .2 | 65.2 |
|  | 26-DEC-2014 | 9 | .0 | .0 | 65.2 |
|  | 27-DEC-2014 | 129 | .4 | .4 | 65.6 |
|  | 28-DEC-2014 | 127 | .4 | .4 | 66.0 |
|  | 29-DEC-2014 | 148 | .5 | .5 | 66.5 |
|  | 30-DEC-2014 | 113 | .4 | .4 | 66.9 |
|  | 31-DEC-2014 | 145 | .5 | .5 | 67.4 |
|  | 01-JAN-2015 | 125 | .4 | .4 | 67.8 |
|  | 02-JAN-2015 | 47 | .2 | .2 | 67.9 |
|  | 03-JAN-2015 | 145 | .5 | .5 | 68.4 |
|  | 04-JAN-2015 | 31 | .1 | .1 | 68.5 |
|  | 05-JAN-2015 | 146 | .5 | .5 | 69.0 |
|  | 06-JAN-2015 | 122 | .4 | .4 | 69.4 |
|  | 07-JAN-2015 | 157 | .5 | .5 | 69.9 |
|  | 08-JAN-2015 | 122 | .4 | .4 | 70.3 |
|  | 09-JAN-2015 | 23 | .1 | .1 | 70.4 |
|  | 10-JAN-2015 | 168 | .5 | .5 | 70.9 |
|  | 11-JAN-2015 | 131 | .4 | .4 | 71.3 |
|  | 12-JAN-2015 | 162 | .5 | .5 | 71.9 |
|  | 13-JAN-2015 | 52 | .2 | .2 | 72.0 |
|  | 14-JAN-2015 | 131 | .4 | .4 | 72.5 |
|  | 15-JAN-2015 | 106 | .3 | .3 | 72.8 |
|  | 16-JAN-2015 | 60 | .2 | .2 | 73.0 |
|  | 17-JAN-2015 | 68 | .2 | .2 | 73.2 |
|  | 18-JAN-2015 | 63 | .2 | .2 | 73.4 |
|  | 19-JAN-2015 | 50 | .2 | .2 | 73.6 |
|  | 20-JAN-2015 | 39 | .1 | .1 | 73.7 |
|  | 21-JAN-2015 | 30 | .1 | .1 | 73.8 |
|  | 22-JAN-2015 | 48 | .2 | .2 | 74.0 |
|  | 23-JAN-2015 | 18 | .1 | .1 | 74.0 |
|  | 24-JAN-2015 | 77 | .3 | .3 | 74.3 |
|  | 25-JAN-2015 | 93 | .3 | .3 | 74.6 |
|  | 26-JAN-2015 | 81 | .3 | .3 | 74.9 |
|  | 27-JAN-2015 | 79 | .3 | .3 | 75.1 |
|  | 28-JAN-2015 | 41 | .1 | .1 | 75.3 |
|  | 29-JAN-2015 | 25 | .1 | .1 | 75.3 |
|  | 30-JAN-2015 | 14 | .0 | .0 | 75.4 |
|  | 31-JAN-2015 | 39 | .1 | .1 | 75.5 |
|  | 01-FEB-2015 | 72 | .2 | .2 | 75.8 |
|  | 02-FEB-2015 | 40 | .1 | .1 | 75.9 |
|  | 03-FEB-2015 | 21 | .1 | .1 | 76.0 |
|  | 04-FEB-2015 | 39 | .1 | .1 | 76.1 |
|  | 05-FEB-2015 | 33 | .1 | .1 | 76.2 |
|  | 06-FEB-2015 | 16 | .1 | .1 | 76.2 |
|  | 07-FEB-2015 | 39 | .1 | .1 | 76.4 |
|  | 08-FEB-2015 | 49 | .2 | .2 | 76.5 |
|  | 09-FEB-2015 | 37 | .1 | .1 | 76.7 |
|  | 10-FEB-2015 | 33 | .1 | .1 | 76.8 |
|  | 11-FEB-2015 | 35 | .1 | .1 | 76.9 |
|  | 12-FEB-2015 | 22 | .1 | .1 | 76.9 |
|  | 13-FEB-2015 | 20 | .1 | .1 | 77.0 |
|  | 14-FEB-2015 | 37 | .1 | .1 | 77.1 |
|  | 15-FEB-2015 | 41 | .1 | .1 | 77.3 |
|  | 16-FEB-2015 | 43 | .1 | .1 | 77.4 |
|  | 17-FEB-2015 | 32 | .1 | .1 | 77.5 |
|  | 18-FEB-2015 | 35 | .1 | .1 | 77.6 |
|  | 19-FEB-2015 | 39 | .1 | .1 | 77.8 |
|  | 20-FEB-2015 | 24 | .1 | .1 | 77.8 |
|  | 21-FEB-2015 | 25 | .1 | .1 | 77.9 |
|  | 22-FEB-2015 | 62 | .2 | .2 | 78.1 |
|  | 23-FEB-2015 | 54 | .2 | .2 | 78.3 |
|  | 24-FEB-2015 | 34 | .1 | .1 | 78.4 |
|  | 25-FEB-2015 | 36 | .1 | .1 | 78.5 |
|  | 26-FEB-2015 | 32 | .1 | .1 | 78.6 |
|  | 27-FEB-2015 | 15 | .0 | .0 | 78.7 |
|  | 28-FEB-2015 | 48 | .2 | .2 | 78.8 |
|  | 01-MAR-2015 | 43 | .1 | .1 | 79.0 |
|  | 02-MAR-2015 | 35 | .1 | .1 | 79.1 |
|  | 03-MAR-2015 | 31 | .1 | .1 | 79.2 |
|  | 04-MAR-2015 | 56 | .2 | .2 | 79.4 |
|  | 05-MAR-2015 | 19 | .1 | .1 | 79.4 |
|  | 06-MAR-2015 | 13 | .0 | .0 | 79.5 |
|  | 07-MAR-2015 | 37 | .1 | .1 | 79.6 |
|  | 08-MAR-2015 | 29 | .1 | .1 | 79.7 |
|  | 09-MAR-2015 | 24 | .1 | .1 | 79.8 |
|  | 10-MAR-2015 | 23 | .1 | .1 | 79.8 |
|  | 11-MAR-2015 | 27 | .1 | .1 | 79.9 |
|  | 12-MAR-2015 | 18 | .1 | .1 | 80.0 |
|  | 13-MAR-2015 | 15 | .0 | .0 | 80.0 |
|  | 14-MAR-2015 | 61 | .2 | .2 | 80.2 |
|  | 15-MAR-2015 | 31 | .1 | .1 | 80.3 |
|  | 16-MAR-2015 | 85 | .3 | .3 | 80.6 |
|  | 17-MAR-2015 | 11 | .0 | .0 | 80.7 |
|  | 18-MAR-2015 | 38 | .1 | .1 | 80.8 |
|  | 19-MAR-2015 | 41 | .1 | .1 | 80.9 |
|  | 20-MAR-2015 | 7 | .0 | .0 | 80.9 |
|  | 21-MAR-2015 | 41 | .1 | .1 | 81.1 |
|  | 22-MAR-2015 | 51 | .2 | .2 | 81.2 |
|  | 23-MAR-2015 | 81 | .3 | .3 | 81.5 |
|  | 24-MAR-2015 | 73 | .2 | .2 | 81.7 |
|  | 25-MAR-2015 | 90 | .3 | .3 | 82.0 |
|  | 26-MAR-2015 | 12 | .0 | .0 | 82.1 |
|  | 27-MAR-2015 | 18 | .1 | .1 | 82.1 |
|  | 28-MAR-2015 | 64 | .2 | .2 | 82.3 |
|  | 29-MAR-2015 | 51 | .2 | .2 | 82.5 |
|  | 30-MAR-2015 | 122 | .4 | .4 | 82.9 |
|  | 31-MAR-2015 | 69 | .2 | .2 | 83.1 |
|  | 01-APR-2015 | 52 | .2 | .2 | 83.3 |
|  | 02-APR-2015 | 75 | .2 | .2 | 83.6 |
|  | 03-APR-2015 | 42 | .1 | .1 | 83.7 |
|  | 04-APR-2015 | 68 | .2 | .2 | 83.9 |
|  | 05-APR-2015 | 86 | .3 | .3 | 84.2 |
|  | 06-APR-2015 | 51 | .2 | .2 | 84.4 |
|  | 07-APR-2015 | 43 | .1 | .1 | 84.5 |
|  | 08-APR-2015 | 82 | .3 | .3 | 84.8 |
|  | 09-APR-2015 | 45 | .1 | .1 | 84.9 |
|  | 10-APR-2015 | 32 | .1 | .1 | 85.0 |
|  | 11-APR-2015 | 94 | .3 | .3 | 85.3 |
|  | 12-APR-2015 | 86 | .3 | .3 | 85.6 |
|  | 13-APR-2015 | 85 | .3 | .3 | 85.9 |
|  | 14-APR-2015 | 11 | .0 | .0 | 85.9 |
|  | 15-APR-2015 | 81 | .3 | .3 | 86.2 |
|  | 16-APR-2015 | 35 | .1 | .1 | 86.3 |
|  | 17-APR-2015 | 12 | .0 | .0 | 86.3 |
|  | 18-APR-2015 | 80 | .3 | .3 | 86.6 |
|  | 19-APR-2015 | 79 | .3 | .3 | 86.9 |
|  | 20-APR-2015 | 44 | .1 | .1 | 87.0 |
|  | 21-APR-2015 | 79 | .3 | .3 | 87.3 |
|  | 22-APR-2015 | 77 | .3 | .3 | 87.5 |
|  | 23-APR-2015 | 26 | .1 | .1 | 87.6 |
|  | 24-APR-2015 | 25 | .1 | .1 | 87.7 |
|  | 25-APR-2015 | 131 | .4 | .4 | 88.1 |
|  | 26-APR-2015 | 59 | .2 | .2 | 88.3 |
|  | 27-APR-2015 | 86 | .3 | .3 | 88.6 |
|  | 28-APR-2015 | 58 | .2 | .2 | 88.8 |
|  | 29-APR-2015 | 49 | .2 | .2 | 88.9 |
|  | 30-APR-2015 | 30 | .1 | .1 | 89.0 |
|  | 01-MAY-2015 | 7 | .0 | .0 | 89.1 |
|  | 02-MAY-2015 | 39 | .1 | .1 | 89.2 |
|  | 03-MAY-2015 | 13 | .0 | .0 | 89.2 |
|  | 04-MAY-2015 | 33 | .1 | .1 | 89.3 |
|  | 05-MAY-2015 | 35 | .1 | .1 | 89.5 |
|  | 06-MAY-2015 | 31 | .1 | .1 | 89.6 |
|  | 07-MAY-2015 | 8 | .0 | .0 | 89.6 |
|  | 08-MAY-2015 | 3 | .0 | .0 | 89.6 |
|  | 09-MAY-2015 | 45 | .1 | .1 | 89.7 |
|  | 10-MAY-2015 | 48 | .2 | .2 | 89.9 |
|  | 11-MAY-2015 | 39 | .1 | .1 | 90.0 |
|  | 12-MAY-2015 | 23 | .1 | .1 | 90.1 |
|  | 13-MAY-2015 | 16 | .1 | .1 | 90.2 |
|  | 14-MAY-2015 | 25 | .1 | .1 | 90.2 |
|  | 15-MAY-2015 | 9 | .0 | .0 | 90.3 |
|  | 16-MAY-2015 | 29 | .1 | .1 | 90.4 |
|  | 17-MAY-2015 | 31 | .1 | .1 | 90.5 |
|  | 18-MAY-2015 | 25 | .1 | .1 | 90.5 |
|  | 19-MAY-2015 | 28 | .1 | .1 | 90.6 |
|  | 20-MAY-2015 | 39 | .1 | .1 | 90.8 |
|  | 21-MAY-2015 | 13 | .0 | .0 | 90.8 |
|  | 22-MAY-2015 | 18 | .1 | .1 | 90.9 |
|  | 23-MAY-2015 | 37 | .1 | .1 | 91.0 |
|  | 24-MAY-2015 | 32 | .1 | .1 | 91.1 |
|  | 25-MAY-2015 | 34 | .1 | .1 | 91.2 |
|  | 26-MAY-2015 | 41 | .1 | .1 | 91.3 |
|  | 27-MAY-2015 | 36 | .1 | .1 | 91.5 |
|  | 28-MAY-2015 | 19 | .1 | .1 | 91.5 |
|  | 29-MAY-2015 | 7 | .0 | .0 | 91.5 |
|  | 30-MAY-2015 | 25 | .1 | .1 | 91.6 |
|  | 31-MAY-2015 | 29 | .1 | .1 | 91.7 |
|  | 01-JUN-2015 | 38 | .1 | .1 | 91.8 |
|  | 02-JUN-2015 | 29 | .1 | .1 | 91.9 |
|  | 03-JUN-2015 | 25 | .1 | .1 | 92.0 |
|  | 04-JUN-2015 | 48 | .2 | .2 | 92.2 |
|  | 05-JUN-2015 | 22 | .1 | .1 | 92.2 |
|  | 06-JUN-2015 | 39 | .1 | .1 | 92.4 |
|  | 07-JUN-2015 | 61 | .2 | .2 | 92.6 |
|  | 08-JUN-2015 | 42 | .1 | .1 | 92.7 |
|  | 09-JUN-2015 | 42 | .1 | .1 | 92.8 |
|  | 10-JUN-2015 | 67 | .2 | .2 | 93.1 |
|  | 11-JUN-2015 | 35 | .1 | .1 | 93.2 |
|  | 12-JUN-2015 | 13 | .0 | .0 | 93.2 |
|  | 13-JUN-2015 | 32 | .1 | .1 | 93.3 |
|  | 14-JUN-2015 | 46 | .2 | .2 | 93.5 |
|  | 15-JUN-2015 | 62 | .2 | .2 | 93.7 |
|  | 16-JUN-2015 | 51 | .2 | .2 | 93.9 |
|  | 17-JUN-2015 | 45 | .1 | .1 | 94.0 |
|  | 18-JUN-2015 | 20 | .1 | .1 | 94.1 |
|  | 19-JUN-2015 | 11 | .0 | .0 | 94.1 |
|  | 20-JUN-2015 | 59 | .2 | .2 | 94.3 |
|  | 21-JUN-2015 | 53 | .2 | .2 | 94.5 |
|  | 22-JUN-2015 | 45 | .1 | .1 | 94.6 |
|  | 23-JUN-2015 | 22 | .1 | .1 | 94.7 |
|  | 24-JUN-2015 | 36 | .1 | .1 | 94.8 |
|  | 25-JUN-2015 | 20 | .1 | .1 | 94.9 |
|  | 26-JUN-2015 | 20 | .1 | .1 | 94.9 |
|  | 27-JUN-2015 | 29 | .1 | .1 | 95.0 |
|  | 28-JUN-2015 | 21 | .1 | .1 | 95.1 |
|  | 29-JUN-2015 | 20 | .1 | .1 | 95.2 |
|  | 30-JUN-2015 | 42 | .1 | .1 | 95.3 |
|  | 01-JUL-2015 | 28 | .1 | .1 | 95.4 |
|  | 02-JUL-2015 | 21 | .1 | .1 | 95.5 |
|  | 03-JUL-2015 | 3 | .0 | .0 | 95.5 |
|  | 04-JUL-2015 | 24 | .1 | .1 | 95.5 |
|  | 05-JUL-2015 | 12 | .0 | .0 | 95.6 |
|  | 06-JUL-2015 | 25 | .1 | .1 | 95.7 |
|  | 07-JUL-2015 | 12 | .0 | .0 | 95.7 |
|  | 08-JUL-2015 | 7 | .0 | .0 | 95.7 |
|  | 09-JUL-2015 | 2 | .0 | .0 | 95.7 |
|  | 10-JUL-2015 | 6 | .0 | .0 | 95.8 |
|  | 11-JUL-2015 | 13 | .0 | .0 | 95.8 |
|  | 12-JUL-2015 | 8 | .0 | .0 | 95.8 |
|  | 13-JUL-2015 | 11 | .0 | .0 | 95.9 |
|  | 14-JUL-2015 | 15 | .0 | .0 | 95.9 |
|  | 15-JUL-2015 | 8 | .0 | .0 | 95.9 |
|  | 16-JUL-2015 | 12 | .0 | .0 | 96.0 |
|  | 17-JUL-2015 | 7 | .0 | .0 | 96.0 |
|  | 18-JUL-2015 | 10 | .0 | .0 | 96.0 |
|  | 19-JUL-2015 | 11 | .0 | .0 | 96.1 |
|  | 20-JUL-2015 | 20 | .1 | .1 | 96.1 |
|  | 21-JUL-2015 | 9 | .0 | .0 | 96.2 |
|  | 22-JUL-2015 | 12 | .0 | .0 | 96.2 |
|  | 23-JUL-2015 | 7 | .0 | .0 | 96.2 |
|  | 24-JUL-2015 | 6 | .0 | .0 | 96.2 |
|  | 25-JUL-2015 | 13 | .0 | .0 | 96.3 |
|  | 26-JUL-2015 | 17 | .1 | .1 | 96.3 |
|  | 27-JUL-2015 | 17 | .1 | .1 | 96.4 |
|  | 28-JUL-2015 | 15 | .0 | .0 | 96.4 |
|  | 29-JUL-2015 | 12 | .0 | .0 | 96.5 |
|  | 30-JUL-2015 | 28 | .1 | .1 | 96.6 |
|  | 31-JUL-2015 | 1 | .0 | .0 | 96.6 |
|  | 01-AUG-2015 | 24 | .1 | .1 | 96.7 |
|  | 02-AUG-2015 | 8 | .0 | .0 | 96.7 |
|  | 03-AUG-2015 | 9 | .0 | .0 | 96.7 |
|  | 04-AUG-2015 | 18 | .1 | .1 | 96.8 |
|  | 05-AUG-2015 | 9 | .0 | .0 | 96.8 |
|  | 06-AUG-2015 | 11 | .0 | .0 | 96.8 |
|  | 07-AUG-2015 | 7 | .0 | .0 | 96.9 |
|  | 08-AUG-2015 | 21 | .1 | .1 | 96.9 |
|  | 09-AUG-2015 | 20 | .1 | .1 | 97.0 |
|  | 10-AUG-2015 | 12 | .0 | .0 | 97.0 |
|  | 11-AUG-2015 | 16 | .1 | .1 | 97.1 |
|  | 12-AUG-2015 | 25 | .1 | .1 | 97.2 |
|  | 13-AUG-2015 | 9 | .0 | .0 | 97.2 |
|  | 14-AUG-2015 | 4 | .0 | .0 | 97.2 |
|  | 15-AUG-2015 | 8 | .0 | .0 | 97.2 |
|  | 16-AUG-2015 | 21 | .1 | .1 | 97.3 |
|  | 17-AUG-2015 | 15 | .0 | .0 | 97.4 |
|  | 18-AUG-2015 | 12 | .0 | .0 | 97.4 |
|  | 19-AUG-2015 | 8 | .0 | .0 | 97.4 |
|  | 20-AUG-2015 | 16 | .1 | .1 | 97.5 |
|  | 21-AUG-2015 | 4 | .0 | .0 | 97.5 |
|  | 22-AUG-2015 | 24 | .1 | .1 | 97.6 |
|  | 23-AUG-2015 | 15 | .0 | .0 | 97.6 |
|  | 24-AUG-2015 | 9 | .0 | .0 | 97.6 |
|  | 25-AUG-2015 | 8 | .0 | .0 | 97.7 |
|  | 26-AUG-2015 | 7 | .0 | .0 | 97.7 |
|  | 27-AUG-2015 | 6 | .0 | .0 | 97.7 |
|  | 28-AUG-2015 | 5 | .0 | .0 | 97.7 |
|  | 29-AUG-2015 | 5 | .0 | .0 | 97.7 |
|  | 30-AUG-2015 | 7 | .0 | .0 | 97.8 |
|  | 31-AUG-2015 | 14 | .0 | .0 | 97.8 |
|  | 01-SEP-2015 | 10 | .0 | .0 | 97.8 |
|  | 02-SEP-2015 | 8 | .0 | .0 | 97.9 |
|  | 03-SEP-2015 | 7 | .0 | .0 | 97.9 |
|  | 04-SEP-2015 | 3 | .0 | .0 | 97.9 |
|  | 05-SEP-2015 | 6 | .0 | .0 | 97.9 |
|  | 06-SEP-2015 | 7 | .0 | .0 | 98.0 |
|  | 07-SEP-2015 | 9 | .0 | .0 | 98.0 |
|  | 08-SEP-2015 | 5 | .0 | .0 | 98.0 |
|  | 09-SEP-2015 | 7 | .0 | .0 | 98.0 |
|  | 10-SEP-2015 | 9 | .0 | .0 | 98.0 |
|  | 11-SEP-2015 | 3 | .0 | .0 | 98.1 |
|  | 12-SEP-2015 | 10 | .0 | .0 | 98.1 |
|  | 13-SEP-2015 | 14 | .0 | .0 | 98.1 |
|  | 14-SEP-2015 | 6 | .0 | .0 | 98.2 |
|  | 15-SEP-2015 | 9 | .0 | .0 | 98.2 |
|  | 16-SEP-2015 | 6 | .0 | .0 | 98.2 |
|  | 17-SEP-2015 | 3 | .0 | .0 | 98.2 |
|  | 18-SEP-2015 | 8 | .0 | .0 | 98.2 |
|  | 19-SEP-2015 | 5 | .0 | .0 | 98.3 |
|  | 20-SEP-2015 | 8 | .0 | .0 | 98.3 |
|  | 21-SEP-2015 | 7 | .0 | .0 | 98.3 |
|  | 22-SEP-2015 | 11 | .0 | .0 | 98.3 |
|  | 23-SEP-2015 | 6 | .0 | .0 | 98.4 |
|  | 24-SEP-2015 | 6 | .0 | .0 | 98.4 |
|  | 26-SEP-2015 | 6 | .0 | .0 | 98.4 |
|  | 27-SEP-2015 | 13 | .0 | .0 | 98.4 |
|  | 28-SEP-2015 | 8 | .0 | .0 | 98.5 |
|  | 29-SEP-2015 | 5 | .0 | .0 | 98.5 |
|  | 30-SEP-2015 | 7 | .0 | .0 | 98.5 |
|  | 01-OCT-2015 | 16 | .1 | .1 | 98.6 |
|  | 02-OCT-2015 | 4 | .0 | .0 | 98.6 |
|  | 03-OCT-2015 | 5 | .0 | .0 | 98.6 |
|  | 04-OCT-2015 | 9 | .0 | .0 | 98.6 |
|  | 05-OCT-2015 | 6 | .0 | .0 | 98.6 |
|  | 06-OCT-2015 | 8 | .0 | .0 | 98.7 |
|  | 07-OCT-2015 | 13 | .0 | .0 | 98.7 |
|  | 08-OCT-2015 | 7 | .0 | .0 | 98.7 |
|  | 09-OCT-2015 | 2 | .0 | .0 | 98.7 |
|  | 10-OCT-2015 | 13 | .0 | .0 | 98.8 |
|  | 11-OCT-2015 | 8 | .0 | .0 | 98.8 |
|  | 12-OCT-2015 | 6 | .0 | .0 | 98.8 |
|  | 13-OCT-2015 | 12 | .0 | .0 | 98.9 |
|  | 14-OCT-2015 | 9 | .0 | .0 | 98.9 |
|  | 15-OCT-2015 | 9 | .0 | .0 | 98.9 |
|  | 16-OCT-2015 | 12 | .0 | .0 | 99.0 |
|  | 17-OCT-2015 | 9 | .0 | .0 | 99.0 |
|  | 18-OCT-2015 | 6 | .0 | .0 | 99.0 |
|  | 19-OCT-2015 | 12 | .0 | .0 | 99.1 |
|  | 20-OCT-2015 | 5 | .0 | .0 | 99.1 |
|  | 21-OCT-2015 | 7 | .0 | .0 | 99.1 |
|  | 22-OCT-2015 | 7 | .0 | .0 | 99.1 |
|  | 23-OCT-2015 | 2 | .0 | .0 | 99.1 |
|  | 24-OCT-2015 | 4 | .0 | .0 | 99.1 |
|  | 25-OCT-2015 | 12 | .0 | .0 | 99.2 |
|  | 26-OCT-2015 | 1 | .0 | .0 | 99.2 |
|  | 27-OCT-2015 | 4 | .0 | .0 | 99.2 |
|  | 28-OCT-2015 | 7 | .0 | .0 | 99.2 |
|  | 29-OCT-2015 | 34 | .1 | .1 | 99.3 |
|  | 30-OCT-2015 | 29 | .1 | .1 | 99.4 |
|  | 31-OCT-2015 | 91 | .3 | .3 | 99.7 |
|  | 01-NOV-2015 | 5 | .0 | .0 | 99.7 |
|  | 02-NOV-2015 | 5 | .0 | .0 | 99.8 |
|  | 03-NOV-2015 | 2 | .0 | .0 | 99.8 |
|  | 04-NOV-2015 | 6 | .0 | .0 | 99.8 |
|  | 05-NOV-2015 | 1 | .0 | .0 | 99.8 |
|  | 09-NOV-2015 | 3 | .0 | .0 | 99.8 |
|  | 10-NOV-2015 | 1 | .0 | .0 | 99.8 |
|  | 11-NOV-2015 | 4 | .0 | .0 | 99.8 |
|  | 12-NOV-2015 | 6 | .0 | .0 | 99.8 |
|  | 13-NOV-2015 | 1 | .0 | .0 | 99.8 |
|  | 14-NOV-2015 | 5 | .0 | .0 | 99.8 |
|  | 15-NOV-2015 | 11 | .0 | .0 | 99.9 |
|  | 16-NOV-2015 | 6 | .0 | .0 | 99.9 |
|  | 17-NOV-2015 | 3 | .0 | .0 | 99.9 |
|  | 18-NOV-2015 | 2 | .0 | .0 | 99.9 |
|  | 19-NOV-2015 | 2 | .0 | .0 | 99.9 |
|  | 22-NOV-2015 | 4 | .0 | .0 | 99.9 |
|  | 23-NOV-2015 | 6 | .0 | .0 | 100.0 |
|  | 24-NOV-2015 | 3 | .0 | .0 | 100.0 |
|  | 26-NOV-2015 | 2 | .0 | .0 | 100.0 |
|  | 27-NOV-2015 | 1 | .0 | .0 | 100.0 |
|  | 28-NOV-2015 | 1 | .0 | .0 | 100.0 |
|  | 29-NOV-2015 | 1 | .0 | .0 | 100.0 |
|  | 30-NOV-2015 | 5 | .0 | .0 | 100.0 |
|  | Total | 30553 | 100.0 | 100.0 |  |
|  |  |  |  |  |  |

Date of birth of individualDate of birth of individual, table, 1 levels of column headers and 2 levels of row headers, table with 6 columns and 1944 rows

|  |  |  |  |  |  |
| --- | --- | --- | --- | --- | --- |
|  | | Frequency | Percent | Valid Percent | Cumulative Percent |
| Valid | 14-JUN-2009 | 1 | .0 | .0 | .0 |
|  | 15-JUN-2009 | 2 | .0 | .0 | .0 |
|  | 16-JUN-2009 | 1 | .0 | .0 | .0 |
|  | 28-JUN-2009 | 1 | .0 | .0 | .0 |
|  | 02-JUL-2009 | 1 | .0 | .0 | .0 |
|  | 03-JUL-2009 | 1 | .0 | .0 | .0 |
|  | 05-JUL-2009 | 2 | .0 | .0 | .0 |
|  | 09-JUL-2009 | 1 | .0 | .0 | .0 |
|  | 10-JUL-2009 | 1 | .0 | .0 | .0 |
|  | 12-JUL-2009 | 1 | .0 | .0 | .0 |
|  | 13-JUL-2009 | 1 | .0 | .0 | .0 |
|  | 14-JUL-2009 | 3 | .0 | .0 | .1 |
|  | 15-JUL-2009 | 2 | .0 | .0 | .1 |
|  | 17-JUL-2009 | 1 | .0 | .0 | .1 |
|  | 18-JUL-2009 | 1 | .0 | .0 | .1 |
|  | 23-JUL-2009 | 1 | .0 | .0 | .1 |
|  | 25-JUL-2009 | 2 | .0 | .0 | .1 |
|  | 27-JUL-2009 | 1 | .0 | .0 | .1 |
|  | 29-JUL-2009 | 1 | .0 | .0 | .1 |
|  | 01-AUG-2009 | 3 | .0 | .0 | .1 |
|  | 02-AUG-2009 | 1 | .0 | .0 | .1 |
|  | 04-AUG-2009 | 1 | .0 | .0 | .1 |
|  | 05-AUG-2009 | 3 | .0 | .0 | .1 |
|  | 07-AUG-2009 | 2 | .0 | .0 | .1 |
|  | 08-AUG-2009 | 1 | .0 | .0 | .1 |
|  | 10-AUG-2009 | 5 | .0 | .0 | .1 |
|  | 13-AUG-2009 | 2 | .0 | .0 | .1 |
|  | 15-AUG-2009 | 1 | .0 | .0 | .1 |
|  | 17-AUG-2009 | 1 | .0 | .0 | .1 |
|  | 19-AUG-2009 | 1 | .0 | .0 | .2 |
|  | 20-AUG-2009 | 1 | .0 | .0 | .2 |
|  | 21-AUG-2009 | 1 | .0 | .0 | .2 |
|  | 22-AUG-2009 | 1 | .0 | .0 | .2 |
|  | 26-AUG-2009 | 2 | .0 | .0 | .2 |
|  | 27-AUG-2009 | 1 | .0 | .0 | .2 |
|  | 28-AUG-2009 | 2 | .0 | .0 | .2 |
|  | 29-AUG-2009 | 3 | .0 | .0 | .2 |
|  | 31-AUG-2009 | 1 | .0 | .0 | .2 |
|  | 01-SEP-2009 | 3 | .0 | .0 | .2 |
|  | 02-SEP-2009 | 1 | .0 | .0 | .2 |
|  | 03-SEP-2009 | 1 | .0 | .0 | .2 |
|  | 04-SEP-2009 | 2 | .0 | .0 | .2 |
|  | 05-SEP-2009 | 1 | .0 | .0 | .2 |
|  | 06-SEP-2009 | 1 | .0 | .0 | .2 |
|  | 07-SEP-2009 | 4 | .0 | .0 | .2 |
|  | 08-SEP-2009 | 2 | .0 | .0 | .2 |
|  | 09-SEP-2009 | 1 | .0 | .0 | .2 |
|  | 10-SEP-2009 | 5 | .0 | .0 | .3 |
|  | 11-SEP-2009 | 3 | .0 | .0 | .3 |
|  | 14-SEP-2009 | 1 | .0 | .0 | .3 |
|  | 15-SEP-2009 | 2 | .0 | .0 | .3 |
|  | 16-SEP-2009 | 2 | .0 | .0 | .3 |
|  | 20-SEP-2009 | 1 | .0 | .0 | .3 |
|  | 21-SEP-2009 | 1 | .0 | .0 | .3 |
|  | 23-SEP-2009 | 1 | .0 | .0 | .3 |
|  | 24-SEP-2009 | 3 | .0 | .0 | .3 |
|  | 25-SEP-2009 | 1 | .0 | .0 | .3 |
|  | 27-SEP-2009 | 1 | .0 | .0 | .3 |
|  | 28-SEP-2009 | 1 | .0 | .0 | .3 |
|  | 30-SEP-2009 | 1 | .0 | .0 | .3 |
|  | 01-OCT-2009 | 2 | .0 | .0 | .3 |
|  | 05-OCT-2009 | 1 | .0 | .0 | .3 |
|  | 07-OCT-2009 | 1 | .0 | .0 | .3 |
|  | 08-OCT-2009 | 1 | .0 | .0 | .3 |
|  | 10-OCT-2009 | 1 | .0 | .0 | .3 |
|  | 12-OCT-2009 | 1 | .0 | .0 | .3 |
|  | 15-OCT-2009 | 1 | .0 | .0 | .3 |
|  | 16-OCT-2009 | 1 | .0 | .0 | .3 |
|  | 17-OCT-2009 | 1 | .0 | .0 | .4 |
|  | 18-OCT-2009 | 1 | .0 | .0 | .4 |
|  | 27-OCT-2009 | 1 | .0 | .0 | .4 |
|  | 28-OCT-2009 | 1 | .0 | .0 | .4 |
|  | 30-OCT-2009 | 1 | .0 | .0 | .4 |
|  | 01-NOV-2009 | 1 | .0 | .0 | .4 |
|  | 03-NOV-2009 | 1 | .0 | .0 | .4 |
|  | 09-NOV-2009 | 1 | .0 | .0 | .4 |
|  | 10-NOV-2009 | 1 | .0 | .0 | .4 |
|  | 13-NOV-2009 | 1 | .0 | .0 | .4 |
|  | 14-NOV-2009 | 1 | .0 | .0 | .4 |
|  | 15-NOV-2009 | 2 | .0 | .0 | .4 |
|  | 20-NOV-2009 | 1 | .0 | .0 | .4 |
|  | 23-NOV-2009 | 1 | .0 | .0 | .4 |
|  | 25-NOV-2009 | 1 | .0 | .0 | .4 |
|  | 27-NOV-2009 | 3 | .0 | .0 | .4 |
|  | 29-NOV-2009 | 1 | .0 | .0 | .4 |
|  | 30-NOV-2009 | 3 | .0 | .0 | .4 |
|  | 01-DEC-2009 | 4 | .0 | .0 | .4 |
|  | 02-DEC-2009 | 3 | .0 | .0 | .4 |
|  | 04-DEC-2009 | 1 | .0 | .0 | .4 |
|  | 05-DEC-2009 | 2 | .0 | .0 | .5 |
|  | 06-DEC-2009 | 1 | .0 | .0 | .5 |
|  | 08-DEC-2009 | 1 | .0 | .0 | .5 |
|  | 09-DEC-2009 | 1 | .0 | .0 | .5 |
|  | 10-DEC-2009 | 1 | .0 | .0 | .5 |
|  | 12-DEC-2009 | 1 | .0 | .0 | .5 |
|  | 14-DEC-2009 | 1 | .0 | .0 | .5 |
|  | 15-DEC-2009 | 1 | .0 | .0 | .5 |
|  | 16-DEC-2009 | 2 | .0 | .0 | .5 |
|  | 18-DEC-2009 | 2 | .0 | .0 | .5 |
|  | 19-DEC-2009 | 1 | .0 | .0 | .5 |
|  | 21-DEC-2009 | 2 | .0 | .0 | .5 |
|  | 22-DEC-2009 | 1 | .0 | .0 | .5 |
|  | 25-DEC-2009 | 2 | .0 | .0 | .5 |
|  | 28-DEC-2009 | 1 | .0 | .0 | .5 |
|  | 31-DEC-2009 | 2 | .0 | .0 | .5 |
|  | 01-JAN-2010 | 10 | .0 | .0 | .6 |
|  | 02-JAN-2010 | 13 | .0 | .0 | .6 |
|  | 03-JAN-2010 | 2 | .0 | .0 | .6 |
|  | 04-JAN-2010 | 5 | .0 | .0 | .6 |
|  | 05-JAN-2010 | 3 | .0 | .0 | .6 |
|  | 06-JAN-2010 | 1 | .0 | .0 | .6 |
|  | 07-JAN-2010 | 3 | .0 | .0 | .6 |
|  | 08-JAN-2010 | 2 | .0 | .0 | .6 |
|  | 09-JAN-2010 | 2 | .0 | .0 | .7 |
|  | 10-JAN-2010 | 2 | .0 | .0 | .7 |
|  | 11-JAN-2010 | 3 | .0 | .0 | .7 |
|  | 13-JAN-2010 | 1 | .0 | .0 | .7 |
|  | 15-JAN-2010 | 2 | .0 | .0 | .7 |
|  | 17-JAN-2010 | 1 | .0 | .0 | .7 |
|  | 18-JAN-2010 | 1 | .0 | .0 | .7 |
|  | 19-JAN-2010 | 3 | .0 | .0 | .7 |
|  | 20-JAN-2010 | 1 | .0 | .0 | .7 |
|  | 21-JAN-2010 | 1 | .0 | .0 | .7 |
|  | 24-JAN-2010 | 3 | .0 | .0 | .7 |
|  | 26-JAN-2010 | 3 | .0 | .0 | .7 |
|  | 27-JAN-2010 | 4 | .0 | .0 | .7 |
|  | 28-JAN-2010 | 1 | .0 | .0 | .7 |
|  | 30-JAN-2010 | 3 | .0 | .0 | .7 |
|  | 01-FEB-2010 | 12 | .0 | .0 | .8 |
|  | 02-FEB-2010 | 3 | .0 | .0 | .8 |
|  | 03-FEB-2010 | 6 | .0 | .0 | .8 |
|  | 04-FEB-2010 | 2 | .0 | .0 | .8 |
|  | 05-FEB-2010 | 5 | .0 | .0 | .8 |
|  | 06-FEB-2010 | 5 | .0 | .0 | .9 |
|  | 07-FEB-2010 | 1 | .0 | .0 | .9 |
|  | 08-FEB-2010 | 2 | .0 | .0 | .9 |
|  | 09-FEB-2010 | 6 | .0 | .0 | .9 |
|  | 10-FEB-2010 | 12 | .0 | .0 | .9 |
|  | 11-FEB-2010 | 2 | .0 | .0 | .9 |
|  | 12-FEB-2010 | 7 | .0 | .0 | 1.0 |
|  | 13-FEB-2010 | 3 | .0 | .0 | 1.0 |
|  | 15-FEB-2010 | 5 | .0 | .0 | 1.0 |
|  | 16-FEB-2010 | 1 | .0 | .0 | 1.0 |
|  | 17-FEB-2010 | 1 | .0 | .0 | 1.0 |
|  | 19-FEB-2010 | 2 | .0 | .0 | 1.0 |
|  | 20-FEB-2010 | 4 | .0 | .0 | 1.0 |
|  | 21-FEB-2010 | 2 | .0 | .0 | 1.0 |
|  | 22-FEB-2010 | 2 | .0 | .0 | 1.0 |
|  | 23-FEB-2010 | 3 | .0 | .0 | 1.0 |
|  | 24-FEB-2010 | 1 | .0 | .0 | 1.0 |
|  | 25-FEB-2010 | 2 | .0 | .0 | 1.0 |
|  | 26-FEB-2010 | 2 | .0 | .0 | 1.0 |
|  | 28-FEB-2010 | 5 | .0 | .0 | 1.1 |
|  | 01-MAR-2010 | 4 | .0 | .0 | 1.1 |
|  | 02-MAR-2010 | 7 | .0 | .0 | 1.1 |
|  | 03-MAR-2010 | 1 | .0 | .0 | 1.1 |
|  | 04-MAR-2010 | 1 | .0 | .0 | 1.1 |
|  | 05-MAR-2010 | 5 | .0 | .0 | 1.1 |
|  | 06-MAR-2010 | 1 | .0 | .0 | 1.1 |
|  | 08-MAR-2010 | 1 | .0 | .0 | 1.1 |
|  | 09-MAR-2010 | 4 | .0 | .0 | 1.1 |
|  | 10-MAR-2010 | 9 | .0 | .0 | 1.2 |
|  | 12-MAR-2010 | 4 | .0 | .0 | 1.2 |
|  | 13-MAR-2010 | 1 | .0 | .0 | 1.2 |
|  | 14-MAR-2010 | 1 | .0 | .0 | 1.2 |
|  | 15-MAR-2010 | 1 | .0 | .0 | 1.2 |
|  | 16-MAR-2010 | 4 | .0 | .0 | 1.2 |
|  | 17-MAR-2010 | 1 | .0 | .0 | 1.2 |
|  | 20-MAR-2010 | 3 | .0 | .0 | 1.2 |
|  | 21-MAR-2010 | 3 | .0 | .0 | 1.2 |
|  | 22-MAR-2010 | 4 | .0 | .0 | 1.2 |
|  | 23-MAR-2010 | 1 | .0 | .0 | 1.2 |
|  | 24-MAR-2010 | 2 | .0 | .0 | 1.3 |
|  | 25-MAR-2010 | 2 | .0 | .0 | 1.3 |
|  | 26-MAR-2010 | 2 | .0 | .0 | 1.3 |
|  | 27-MAR-2010 | 5 | .0 | .0 | 1.3 |
|  | 01-APR-2010 | 3 | .0 | .0 | 1.3 |
|  | 02-APR-2010 | 2 | .0 | .0 | 1.3 |
|  | 03-APR-2010 | 3 | .0 | .0 | 1.3 |
|  | 04-APR-2010 | 5 | .0 | .0 | 1.3 |
|  | 05-APR-2010 | 3 | .0 | .0 | 1.3 |
|  | 07-APR-2010 | 5 | .0 | .0 | 1.4 |
|  | 08-APR-2010 | 2 | .0 | .0 | 1.4 |
|  | 09-APR-2010 | 2 | .0 | .0 | 1.4 |
|  | 10-APR-2010 | 9 | .0 | .0 | 1.4 |
|  | 11-APR-2010 | 5 | .0 | .0 | 1.4 |
|  | 13-APR-2010 | 2 | .0 | .0 | 1.4 |
|  | 14-APR-2010 | 3 | .0 | .0 | 1.4 |
|  | 16-APR-2010 | 2 | .0 | .0 | 1.4 |
|  | 17-APR-2010 | 2 | .0 | .0 | 1.4 |
|  | 18-APR-2010 | 1 | .0 | .0 | 1.4 |
|  | 19-APR-2010 | 3 | .0 | .0 | 1.5 |
|  | 20-APR-2010 | 4 | .0 | .0 | 1.5 |
|  | 21-APR-2010 | 1 | .0 | .0 | 1.5 |
|  | 22-APR-2010 | 2 | .0 | .0 | 1.5 |
|  | 24-APR-2010 | 1 | .0 | .0 | 1.5 |
|  | 25-APR-2010 | 3 | .0 | .0 | 1.5 |
|  | 28-APR-2010 | 3 | .0 | .0 | 1.5 |
|  | 29-APR-2010 | 1 | .0 | .0 | 1.5 |
|  | 30-APR-2010 | 1 | .0 | .0 | 1.5 |
|  | 01-MAY-2010 | 7 | .0 | .0 | 1.5 |
|  | 02-MAY-2010 | 3 | .0 | .0 | 1.5 |
|  | 03-MAY-2010 | 4 | .0 | .0 | 1.6 |
|  | 04-MAY-2010 | 4 | .0 | .0 | 1.6 |
|  | 05-MAY-2010 | 3 | .0 | .0 | 1.6 |
|  | 06-MAY-2010 | 3 | .0 | .0 | 1.6 |
|  | 07-MAY-2010 | 3 | .0 | .0 | 1.6 |
|  | 08-MAY-2010 | 1 | .0 | .0 | 1.6 |
|  | 09-MAY-2010 | 4 | .0 | .0 | 1.6 |
|  | 10-MAY-2010 | 3 | .0 | .0 | 1.6 |
|  | 11-MAY-2010 | 4 | .0 | .0 | 1.6 |
|  | 12-MAY-2010 | 2 | .0 | .0 | 1.6 |
|  | 14-MAY-2010 | 4 | .0 | .0 | 1.7 |
|  | 15-MAY-2010 | 1 | .0 | .0 | 1.7 |
|  | 18-MAY-2010 | 5 | .0 | .0 | 1.7 |
|  | 19-MAY-2010 | 3 | .0 | .0 | 1.7 |
|  | 20-MAY-2010 | 4 | .0 | .0 | 1.7 |
|  | 21-MAY-2010 | 2 | .0 | .0 | 1.7 |
|  | 22-MAY-2010 | 1 | .0 | .0 | 1.7 |
|  | 23-MAY-2010 | 2 | .0 | .0 | 1.7 |
|  | 24-MAY-2010 | 4 | .0 | .0 | 1.7 |
|  | 25-MAY-2010 | 3 | .0 | .0 | 1.7 |
|  | 27-MAY-2010 | 3 | .0 | .0 | 1.7 |
|  | 28-MAY-2010 | 4 | .0 | .0 | 1.8 |
|  | 29-MAY-2010 | 8 | .0 | .0 | 1.8 |
|  | 30-MAY-2010 | 3 | .0 | .0 | 1.8 |
|  | 01-JUN-2010 | 22 | .1 | .1 | 1.9 |
|  | 02-JUN-2010 | 4 | .0 | .0 | 1.9 |
|  | 03-JUN-2010 | 11 | .0 | .0 | 1.9 |
|  | 04-JUN-2010 | 3 | .0 | .0 | 1.9 |
|  | 05-JUN-2010 | 7 | .0 | .0 | 1.9 |
|  | 06-JUN-2010 | 3 | .0 | .0 | 2.0 |
|  | 07-JUN-2010 | 14 | .0 | .0 | 2.0 |
|  | 08-JUN-2010 | 2 | .0 | .0 | 2.0 |
|  | 09-JUN-2010 | 5 | .0 | .0 | 2.0 |
|  | 10-JUN-2010 | 12 | .0 | .0 | 2.1 |
|  | 11-JUN-2010 | 5 | .0 | .0 | 2.1 |
|  | 12-JUN-2010 | 11 | .0 | .0 | 2.1 |
|  | 13-JUN-2010 | 2 | .0 | .0 | 2.1 |
|  | 14-JUN-2010 | 4 | .0 | .0 | 2.1 |
|  | 15-JUN-2010 | 3 | .0 | .0 | 2.1 |
|  | 16-JUN-2010 | 3 | .0 | .0 | 2.2 |
|  | 17-JUN-2010 | 1 | .0 | .0 | 2.2 |
|  | 18-JUN-2010 | 1 | .0 | .0 | 2.2 |
|  | 19-JUN-2010 | 3 | .0 | .0 | 2.2 |
|  | 20-JUN-2010 | 17 | .1 | .1 | 2.2 |
|  | 21-JUN-2010 | 7 | .0 | .0 | 2.3 |
|  | 22-JUN-2010 | 6 | .0 | .0 | 2.3 |
|  | 23-JUN-2010 | 3 | .0 | .0 | 2.3 |
|  | 24-JUN-2010 | 2 | .0 | .0 | 2.3 |
|  | 25-JUN-2010 | 6 | .0 | .0 | 2.3 |
|  | 26-JUN-2010 | 5 | .0 | .0 | 2.3 |
|  | 27-JUN-2010 | 3 | .0 | .0 | 2.3 |
|  | 28-JUN-2010 | 3 | .0 | .0 | 2.3 |
|  | 29-JUN-2010 | 3 | .0 | .0 | 2.4 |
|  | 30-JUN-2010 | 10 | .0 | .0 | 2.4 |
|  | 01-JUL-2010 | 33 | .1 | .1 | 2.5 |
|  | 02-JUL-2010 | 14 | .0 | .0 | 2.5 |
|  | 03-JUL-2010 | 9 | .0 | .0 | 2.6 |
|  | 04-JUL-2010 | 4 | .0 | .0 | 2.6 |
|  | 05-JUL-2010 | 18 | .1 | .1 | 2.6 |
|  | 06-JUL-2010 | 9 | .0 | .0 | 2.7 |
|  | 07-JUL-2010 | 6 | .0 | .0 | 2.7 |
|  | 08-JUL-2010 | 10 | .0 | .0 | 2.7 |
|  | 09-JUL-2010 | 19 | .1 | .1 | 2.8 |
|  | 10-JUL-2010 | 46 | .2 | .2 | 2.9 |
|  | 11-JUL-2010 | 4 | .0 | .0 | 2.9 |
|  | 12-JUL-2010 | 30 | .1 | .1 | 3.0 |
|  | 13-JUL-2010 | 5 | .0 | .0 | 3.1 |
|  | 14-JUL-2010 | 12 | .0 | .0 | 3.1 |
|  | 15-JUL-2010 | 15 | .0 | .0 | 3.2 |
|  | 16-JUL-2010 | 9 | .0 | .0 | 3.2 |
|  | 17-JUL-2010 | 4 | .0 | .0 | 3.2 |
|  | 18-JUL-2010 | 11 | .0 | .0 | 3.2 |
|  | 19-JUL-2010 | 6 | .0 | .0 | 3.3 |
|  | 20-JUL-2010 | 26 | .1 | .1 | 3.3 |
|  | 21-JUL-2010 | 4 | .0 | .0 | 3.3 |
|  | 22-JUL-2010 | 10 | .0 | .0 | 3.4 |
|  | 23-JUL-2010 | 6 | .0 | .0 | 3.4 |
|  | 24-JUL-2010 | 3 | .0 | .0 | 3.4 |
|  | 25-JUL-2010 | 13 | .0 | .0 | 3.5 |
|  | 26-JUL-2010 | 15 | .0 | .0 | 3.5 |
|  | 27-JUL-2010 | 14 | .0 | .0 | 3.5 |
|  | 28-JUL-2010 | 13 | .0 | .0 | 3.6 |
|  | 29-JUL-2010 | 13 | .0 | .0 | 3.6 |
|  | 30-JUL-2010 | 9 | .0 | .0 | 3.7 |
|  | 31-JUL-2010 | 3 | .0 | .0 | 3.7 |
|  | 01-AUG-2010 | 37 | .1 | .1 | 3.8 |
|  | 02-AUG-2010 | 39 | .1 | .1 | 3.9 |
|  | 03-AUG-2010 | 25 | .1 | .1 | 4.0 |
|  | 04-AUG-2010 | 18 | .1 | .1 | 4.1 |
|  | 05-AUG-2010 | 26 | .1 | .1 | 4.1 |
|  | 06-AUG-2010 | 15 | .0 | .0 | 4.2 |
|  | 07-AUG-2010 | 21 | .1 | .1 | 4.3 |
|  | 08-AUG-2010 | 5 | .0 | .0 | 4.3 |
|  | 09-AUG-2010 | 38 | .1 | .1 | 4.4 |
|  | 10-AUG-2010 | 55 | .2 | .2 | 4.6 |
|  | 11-AUG-2010 | 18 | .1 | .1 | 4.6 |
|  | 12-AUG-2010 | 37 | .1 | .1 | 4.8 |
|  | 13-AUG-2010 | 14 | .0 | .0 | 4.8 |
|  | 14-AUG-2010 | 15 | .0 | .0 | 4.9 |
|  | 15-AUG-2010 | 35 | .1 | .1 | 5.0 |
|  | 16-AUG-2010 | 12 | .0 | .0 | 5.0 |
|  | 17-AUG-2010 | 22 | .1 | .1 | 5.1 |
|  | 18-AUG-2010 | 24 | .1 | .1 | 5.2 |
|  | 19-AUG-2010 | 17 | .1 | .1 | 5.2 |
|  | 20-AUG-2010 | 52 | .2 | .2 | 5.4 |
|  | 21-AUG-2010 | 18 | .1 | .1 | 5.4 |
|  | 22-AUG-2010 | 26 | .1 | .1 | 5.5 |
|  | 23-AUG-2010 | 3 | .0 | .0 | 5.5 |
|  | 24-AUG-2010 | 13 | .0 | .0 | 5.6 |
|  | 25-AUG-2010 | 16 | .1 | .1 | 5.6 |
|  | 26-AUG-2010 | 19 | .1 | .1 | 5.7 |
|  | 27-AUG-2010 | 11 | .0 | .0 | 5.7 |
|  | 28-AUG-2010 | 15 | .0 | .0 | 5.8 |
|  | 29-AUG-2010 | 13 | .0 | .0 | 5.8 |
|  | 30-AUG-2010 | 15 | .0 | .0 | 5.9 |
|  | 31-AUG-2010 | 4 | .0 | .0 | 5.9 |
|  | 01-SEP-2010 | 94 | .3 | .3 | 6.2 |
|  | 02-SEP-2010 | 72 | .2 | .2 | 6.4 |
|  | 03-SEP-2010 | 32 | .1 | .1 | 6.5 |
|  | 04-SEP-2010 | 25 | .1 | .1 | 6.6 |
|  | 05-SEP-2010 | 29 | .1 | .1 | 6.7 |
|  | 06-SEP-2010 | 18 | .1 | .1 | 6.8 |
|  | 07-SEP-2010 | 35 | .1 | .1 | 6.9 |
|  | 08-SEP-2010 | 22 | .1 | .1 | 7.0 |
|  | 09-SEP-2010 | 30 | .1 | .1 | 7.1 |
|  | 10-SEP-2010 | 46 | .2 | .2 | 7.2 |
|  | 11-SEP-2010 | 14 | .0 | .0 | 7.3 |
|  | 12-SEP-2010 | 23 | .1 | .1 | 7.3 |
|  | 13-SEP-2010 | 12 | .0 | .0 | 7.4 |
|  | 14-SEP-2010 | 14 | .0 | .0 | 7.4 |
|  | 15-SEP-2010 | 23 | .1 | .1 | 7.5 |
|  | 16-SEP-2010 | 7 | .0 | .0 | 7.5 |
|  | 17-SEP-2010 | 10 | .0 | .0 | 7.5 |
|  | 18-SEP-2010 | 9 | .0 | .0 | 7.6 |
|  | 19-SEP-2010 | 13 | .0 | .0 | 7.6 |
|  | 20-SEP-2010 | 13 | .0 | .0 | 7.7 |
|  | 21-SEP-2010 | 6 | .0 | .0 | 7.7 |
|  | 22-SEP-2010 | 12 | .0 | .0 | 7.7 |
|  | 23-SEP-2010 | 5 | .0 | .0 | 7.7 |
|  | 24-SEP-2010 | 7 | .0 | .0 | 7.8 |
|  | 25-SEP-2010 | 13 | .0 | .0 | 7.8 |
|  | 26-SEP-2010 | 5 | .0 | .0 | 7.8 |
|  | 27-SEP-2010 | 7 | .0 | .0 | 7.8 |
|  | 28-SEP-2010 | 9 | .0 | .0 | 7.9 |
|  | 29-SEP-2010 | 4 | .0 | .0 | 7.9 |
|  | 30-SEP-2010 | 6 | .0 | .0 | 7.9 |
|  | 01-OCT-2010 | 10 | .0 | .0 | 7.9 |
|  | 02-OCT-2010 | 11 | .0 | .0 | 8.0 |
|  | 03-OCT-2010 | 8 | .0 | .0 | 8.0 |
|  | 04-OCT-2010 | 8 | .0 | .0 | 8.0 |
|  | 05-OCT-2010 | 7 | .0 | .0 | 8.0 |
|  | 06-OCT-2010 | 5 | .0 | .0 | 8.1 |
|  | 07-OCT-2010 | 9 | .0 | .0 | 8.1 |
|  | 08-OCT-2010 | 7 | .0 | .0 | 8.1 |
|  | 09-OCT-2010 | 9 | .0 | .0 | 8.1 |
|  | 10-OCT-2010 | 19 | .1 | .1 | 8.2 |
|  | 11-OCT-2010 | 2 | .0 | .0 | 8.2 |
|  | 12-OCT-2010 | 14 | .0 | .0 | 8.3 |
|  | 13-OCT-2010 | 1 | .0 | .0 | 8.3 |
|  | 14-OCT-2010 | 3 | .0 | .0 | 8.3 |
|  | 15-OCT-2010 | 6 | .0 | .0 | 8.3 |
|  | 16-OCT-2010 | 2 | .0 | .0 | 8.3 |
|  | 17-OCT-2010 | 3 | .0 | .0 | 8.3 |
|  | 18-OCT-2010 | 3 | .0 | .0 | 8.3 |
|  | 19-OCT-2010 | 5 | .0 | .0 | 8.3 |
|  | 20-OCT-2010 | 9 | .0 | .0 | 8.4 |
|  | 21-OCT-2010 | 3 | .0 | .0 | 8.4 |
|  | 22-OCT-2010 | 6 | .0 | .0 | 8.4 |
|  | 23-OCT-2010 | 3 | .0 | .0 | 8.4 |
|  | 24-OCT-2010 | 5 | .0 | .0 | 8.4 |
|  | 25-OCT-2010 | 5 | .0 | .0 | 8.4 |
|  | 26-OCT-2010 | 5 | .0 | .0 | 8.5 |
|  | 27-OCT-2010 | 5 | .0 | .0 | 8.5 |
|  | 28-OCT-2010 | 7 | .0 | .0 | 8.5 |
|  | 29-OCT-2010 | 3 | .0 | .0 | 8.5 |
|  | 30-OCT-2010 | 5 | .0 | .0 | 8.5 |
|  | 31-OCT-2010 | 1 | .0 | .0 | 8.5 |
|  | 01-NOV-2010 | 7 | .0 | .0 | 8.5 |
|  | 02-NOV-2010 | 6 | .0 | .0 | 8.6 |
|  | 03-NOV-2010 | 7 | .0 | .0 | 8.6 |
|  | 04-NOV-2010 | 3 | .0 | .0 | 8.6 |
|  | 05-NOV-2010 | 3 | .0 | .0 | 8.6 |
|  | 06-NOV-2010 | 9 | .0 | .0 | 8.6 |
|  | 07-NOV-2010 | 5 | .0 | .0 | 8.7 |
|  | 08-NOV-2010 | 3 | .0 | .0 | 8.7 |
|  | 09-NOV-2010 | 5 | .0 | .0 | 8.7 |
|  | 10-NOV-2010 | 5 | .0 | .0 | 8.7 |
|  | 11-NOV-2010 | 3 | .0 | .0 | 8.7 |
|  | 12-NOV-2010 | 5 | .0 | .0 | 8.7 |
|  | 13-NOV-2010 | 4 | .0 | .0 | 8.7 |
|  | 14-NOV-2010 | 1 | .0 | .0 | 8.7 |
|  | 15-NOV-2010 | 7 | .0 | .0 | 8.8 |
|  | 16-NOV-2010 | 1 | .0 | .0 | 8.8 |
|  | 17-NOV-2010 | 6 | .0 | .0 | 8.8 |
|  | 18-NOV-2010 | 3 | .0 | .0 | 8.8 |
|  | 19-NOV-2010 | 4 | .0 | .0 | 8.8 |
|  | 20-NOV-2010 | 5 | .0 | .0 | 8.8 |
|  | 21-NOV-2010 | 5 | .0 | .0 | 8.8 |
|  | 22-NOV-2010 | 1 | .0 | .0 | 8.8 |
|  | 23-NOV-2010 | 3 | .0 | .0 | 8.9 |
|  | 24-NOV-2010 | 4 | .0 | .0 | 8.9 |
|  | 25-NOV-2010 | 5 | .0 | .0 | 8.9 |
|  | 26-NOV-2010 | 3 | .0 | .0 | 8.9 |
|  | 27-NOV-2010 | 3 | .0 | .0 | 8.9 |
|  | 28-NOV-2010 | 3 | .0 | .0 | 8.9 |
|  | 29-NOV-2010 | 4 | .0 | .0 | 8.9 |
|  | 30-NOV-2010 | 4 | .0 | .0 | 8.9 |
|  | 01-DEC-2010 | 11 | .0 | .0 | 9.0 |
|  | 02-DEC-2010 | 5 | .0 | .0 | 9.0 |
|  | 03-DEC-2010 | 2 | .0 | .0 | 9.0 |
|  | 04-DEC-2010 | 1 | .0 | .0 | 9.0 |
|  | 05-DEC-2010 | 8 | .0 | .0 | 9.0 |
|  | 06-DEC-2010 | 9 | .0 | .0 | 9.1 |
|  | 07-DEC-2010 | 3 | .0 | .0 | 9.1 |
|  | 08-DEC-2010 | 5 | .0 | .0 | 9.1 |
|  | 09-DEC-2010 | 3 | .0 | .0 | 9.1 |
|  | 10-DEC-2010 | 15 | .0 | .0 | 9.1 |
|  | 11-DEC-2010 | 3 | .0 | .0 | 9.2 |
|  | 12-DEC-2010 | 6 | .0 | .0 | 9.2 |
|  | 13-DEC-2010 | 2 | .0 | .0 | 9.2 |
|  | 14-DEC-2010 | 2 | .0 | .0 | 9.2 |
|  | 15-DEC-2010 | 6 | .0 | .0 | 9.2 |
|  | 16-DEC-2010 | 10 | .0 | .0 | 9.2 |
|  | 17-DEC-2010 | 3 | .0 | .0 | 9.2 |
|  | 19-DEC-2010 | 3 | .0 | .0 | 9.3 |
|  | 20-DEC-2010 | 7 | .0 | .0 | 9.3 |
|  | 21-DEC-2010 | 3 | .0 | .0 | 9.3 |
|  | 22-DEC-2010 | 3 | .0 | .0 | 9.3 |
|  | 24-DEC-2010 | 1 | .0 | .0 | 9.3 |
|  | 25-DEC-2010 | 5 | .0 | .0 | 9.3 |
|  | 26-DEC-2010 | 6 | .0 | .0 | 9.3 |
|  | 27-DEC-2010 | 4 | .0 | .0 | 9.4 |
|  | 28-DEC-2010 | 5 | .0 | .0 | 9.4 |
|  | 29-DEC-2010 | 7 | .0 | .0 | 9.4 |
|  | 30-DEC-2010 | 8 | .0 | .0 | 9.4 |
|  | 31-DEC-2010 | 3 | .0 | .0 | 9.4 |
|  | 01-JAN-2011 | 65 | .2 | .2 | 9.6 |
|  | 02-JAN-2011 | 26 | .1 | .1 | 9.7 |
|  | 03-JAN-2011 | 20 | .1 | .1 | 9.8 |
|  | 04-JAN-2011 | 7 | .0 | .0 | 9.8 |
|  | 05-JAN-2011 | 21 | .1 | .1 | 9.9 |
|  | 06-JAN-2011 | 16 | .1 | .1 | 9.9 |
|  | 07-JAN-2011 | 14 | .0 | .0 | 10.0 |
|  | 08-JAN-2011 | 12 | .0 | .0 | 10.0 |
|  | 09-JAN-2011 | 10 | .0 | .0 | 10.1 |
|  | 10-JAN-2011 | 28 | .1 | .1 | 10.1 |
|  | 11-JAN-2011 | 10 | .0 | .0 | 10.2 |
|  | 12-JAN-2011 | 16 | .1 | .1 | 10.2 |
|  | 13-JAN-2011 | 4 | .0 | .0 | 10.2 |
|  | 14-JAN-2011 | 3 | .0 | .0 | 10.3 |
|  | 15-JAN-2011 | 9 | .0 | .0 | 10.3 |
|  | 16-JAN-2011 | 7 | .0 | .0 | 10.3 |
|  | 17-JAN-2011 | 8 | .0 | .0 | 10.3 |
|  | 18-JAN-2011 | 10 | .0 | .0 | 10.4 |
|  | 19-JAN-2011 | 5 | .0 | .0 | 10.4 |
|  | 20-JAN-2011 | 13 | .0 | .0 | 10.4 |
|  | 21-JAN-2011 | 10 | .0 | .0 | 10.5 |
|  | 22-JAN-2011 | 6 | .0 | .0 | 10.5 |
|  | 23-JAN-2011 | 6 | .0 | .0 | 10.5 |
|  | 24-JAN-2011 | 3 | .0 | .0 | 10.5 |
|  | 25-JAN-2011 | 9 | .0 | .0 | 10.5 |
|  | 26-JAN-2011 | 2 | .0 | .0 | 10.5 |
|  | 27-JAN-2011 | 7 | .0 | .0 | 10.6 |
|  | 28-JAN-2011 | 7 | .0 | .0 | 10.6 |
|  | 29-JAN-2011 | 3 | .0 | .0 | 10.6 |
|  | 30-JAN-2011 | 10 | .0 | .0 | 10.6 |
|  | 31-JAN-2011 | 7 | .0 | .0 | 10.7 |
|  | 01-FEB-2011 | 49 | .2 | .2 | 10.8 |
|  | 02-FEB-2011 | 13 | .0 | .0 | 10.9 |
|  | 03-FEB-2011 | 16 | .1 | .1 | 10.9 |
|  | 04-FEB-2011 | 19 | .1 | .1 | 11.0 |
|  | 05-FEB-2011 | 15 | .0 | .0 | 11.0 |
|  | 06-FEB-2011 | 8 | .0 | .0 | 11.0 |
|  | 07-FEB-2011 | 11 | .0 | .0 | 11.1 |
|  | 08-FEB-2011 | 10 | .0 | .0 | 11.1 |
|  | 09-FEB-2011 | 23 | .1 | .1 | 11.2 |
|  | 10-FEB-2011 | 34 | .1 | .1 | 11.3 |
|  | 11-FEB-2011 | 9 | .0 | .0 | 11.3 |
|  | 12-FEB-2011 | 17 | .1 | .1 | 11.4 |
|  | 13-FEB-2011 | 10 | .0 | .0 | 11.4 |
|  | 14-FEB-2011 | 8 | .0 | .0 | 11.4 |
|  | 15-FEB-2011 | 22 | .1 | .1 | 11.5 |
|  | 16-FEB-2011 | 6 | .0 | .0 | 11.5 |
|  | 17-FEB-2011 | 6 | .0 | .0 | 11.6 |
|  | 18-FEB-2011 | 9 | .0 | .0 | 11.6 |
|  | 19-FEB-2011 | 11 | .0 | .0 | 11.6 |
|  | 20-FEB-2011 | 20 | .1 | .1 | 11.7 |
|  | 21-FEB-2011 | 6 | .0 | .0 | 11.7 |
|  | 22-FEB-2011 | 11 | .0 | .0 | 11.7 |
|  | 23-FEB-2011 | 7 | .0 | .0 | 11.8 |
|  | 24-FEB-2011 | 10 | .0 | .0 | 11.8 |
|  | 25-FEB-2011 | 13 | .0 | .0 | 11.8 |
|  | 26-FEB-2011 | 7 | .0 | .0 | 11.9 |
|  | 27-FEB-2011 | 14 | .0 | .0 | 11.9 |
|  | 28-FEB-2011 | 18 | .1 | .1 | 12.0 |
|  | 01-MAR-2011 | 50 | .2 | .2 | 12.1 |
|  | 02-MAR-2011 | 32 | .1 | .1 | 12.2 |
|  | 03-MAR-2011 | 22 | .1 | .1 | 12.3 |
|  | 04-MAR-2011 | 20 | .1 | .1 | 12.4 |
|  | 05-MAR-2011 | 30 | .1 | .1 | 12.5 |
|  | 06-MAR-2011 | 13 | .0 | .0 | 12.5 |
|  | 07-MAR-2011 | 25 | .1 | .1 | 12.6 |
|  | 08-MAR-2011 | 13 | .0 | .0 | 12.6 |
|  | 09-MAR-2011 | 31 | .1 | .1 | 12.7 |
|  | 10-MAR-2011 | 38 | .1 | .1 | 12.9 |
|  | 11-MAR-2011 | 21 | .1 | .1 | 12.9 |
|  | 12-MAR-2011 | 16 | .1 | .1 | 13.0 |
|  | 13-MAR-2011 | 10 | .0 | .0 | 13.0 |
|  | 14-MAR-2011 | 15 | .0 | .0 | 13.1 |
|  | 15-MAR-2011 | 18 | .1 | .1 | 13.1 |
|  | 16-MAR-2011 | 11 | .0 | .0 | 13.2 |
|  | 17-MAR-2011 | 8 | .0 | .0 | 13.2 |
|  | 18-MAR-2011 | 10 | .0 | .0 | 13.2 |
|  | 19-MAR-2011 | 8 | .0 | .0 | 13.2 |
|  | 20-MAR-2011 | 9 | .0 | .0 | 13.3 |
|  | 21-MAR-2011 | 4 | .0 | .0 | 13.3 |
|  | 22-MAR-2011 | 6 | .0 | .0 | 13.3 |
|  | 23-MAR-2011 | 11 | .0 | .0 | 13.3 |
|  | 24-MAR-2011 | 8 | .0 | .0 | 13.4 |
|  | 25-MAR-2011 | 10 | .0 | .0 | 13.4 |
|  | 26-MAR-2011 | 5 | .0 | .0 | 13.4 |
|  | 27-MAR-2011 | 5 | .0 | .0 | 13.4 |
|  | 28-MAR-2011 | 3 | .0 | .0 | 13.4 |
|  | 29-MAR-2011 | 6 | .0 | .0 | 13.5 |
|  | 30-MAR-2011 | 3 | .0 | .0 | 13.5 |
|  | 31-MAR-2011 | 4 | .0 | .0 | 13.5 |
|  | 01-APR-2011 | 24 | .1 | .1 | 13.6 |
|  | 02-APR-2011 | 13 | .0 | .0 | 13.6 |
|  | 03-APR-2011 | 22 | .1 | .1 | 13.7 |
|  | 04-APR-2011 | 15 | .0 | .0 | 13.7 |
|  | 05-APR-2011 | 16 | .1 | .1 | 13.8 |
|  | 06-APR-2011 | 10 | .0 | .0 | 13.8 |
|  | 07-APR-2011 | 7 | .0 | .0 | 13.8 |
|  | 08-APR-2011 | 13 | .0 | .0 | 13.9 |
|  | 09-APR-2011 | 10 | .0 | .0 | 13.9 |
|  | 10-APR-2011 | 11 | .0 | .0 | 13.9 |
|  | 11-APR-2011 | 10 | .0 | .0 | 14.0 |
|  | 12-APR-2011 | 11 | .0 | .0 | 14.0 |
|  | 13-APR-2011 | 8 | .0 | .0 | 14.0 |
|  | 14-APR-2011 | 5 | .0 | .0 | 14.1 |
|  | 15-APR-2011 | 13 | .0 | .0 | 14.1 |
|  | 16-APR-2011 | 11 | .0 | .0 | 14.1 |
|  | 17-APR-2011 | 5 | .0 | .0 | 14.2 |
|  | 18-APR-2011 | 7 | .0 | .0 | 14.2 |
|  | 19-APR-2011 | 9 | .0 | .0 | 14.2 |
|  | 20-APR-2011 | 16 | .1 | .1 | 14.3 |
|  | 21-APR-2011 | 4 | .0 | .0 | 14.3 |
|  | 22-APR-2011 | 7 | .0 | .0 | 14.3 |
|  | 23-APR-2011 | 7 | .0 | .0 | 14.3 |
|  | 24-APR-2011 | 7 | .0 | .0 | 14.3 |
|  | 25-APR-2011 | 5 | .0 | .0 | 14.4 |
|  | 26-APR-2011 | 3 | .0 | .0 | 14.4 |
|  | 27-APR-2011 | 4 | .0 | .0 | 14.4 |
|  | 29-APR-2011 | 4 | .0 | .0 | 14.4 |
|  | 30-APR-2011 | 4 | .0 | .0 | 14.4 |
|  | 01-MAY-2011 | 24 | .1 | .1 | 14.5 |
|  | 02-MAY-2011 | 13 | .0 | .0 | 14.5 |
|  | 03-MAY-2011 | 15 | .0 | .0 | 14.6 |
|  | 04-MAY-2011 | 10 | .0 | .0 | 14.6 |
|  | 05-MAY-2011 | 14 | .0 | .0 | 14.7 |
|  | 06-MAY-2011 | 8 | .0 | .0 | 14.7 |
|  | 07-MAY-2011 | 7 | .0 | .0 | 14.7 |
|  | 08-MAY-2011 | 11 | .0 | .0 | 14.7 |
|  | 09-MAY-2011 | 8 | .0 | .0 | 14.8 |
|  | 10-MAY-2011 | 25 | .1 | .1 | 14.8 |
|  | 11-MAY-2011 | 6 | .0 | .0 | 14.9 |
|  | 12-MAY-2011 | 7 | .0 | .0 | 14.9 |
|  | 13-MAY-2011 | 8 | .0 | .0 | 14.9 |
|  | 14-MAY-2011 | 9 | .0 | .0 | 14.9 |
|  | 15-MAY-2011 | 14 | .0 | .0 | 15.0 |
|  | 16-MAY-2011 | 7 | .0 | .0 | 15.0 |
|  | 17-MAY-2011 | 5 | .0 | .0 | 15.0 |
|  | 18-MAY-2011 | 4 | .0 | .0 | 15.0 |
|  | 19-MAY-2011 | 8 | .0 | .0 | 15.1 |
|  | 20-MAY-2011 | 25 | .1 | .1 | 15.2 |
|  | 21-MAY-2011 | 2 | .0 | .0 | 15.2 |
|  | 22-MAY-2011 | 5 | .0 | .0 | 15.2 |
|  | 23-MAY-2011 | 4 | .0 | .0 | 15.2 |
|  | 24-MAY-2011 | 11 | .0 | .0 | 15.2 |
|  | 25-MAY-2011 | 14 | .0 | .0 | 15.3 |
|  | 26-MAY-2011 | 5 | .0 | .0 | 15.3 |
|  | 27-MAY-2011 | 6 | .0 | .0 | 15.3 |
|  | 28-MAY-2011 | 8 | .0 | .0 | 15.3 |
|  | 29-MAY-2011 | 7 | .0 | .0 | 15.4 |
|  | 30-MAY-2011 | 10 | .0 | .0 | 15.4 |
|  | 31-MAY-2011 | 6 | .0 | .0 | 15.4 |
|  | 01-JUN-2011 | 57 | .2 | .2 | 15.6 |
|  | 02-JUN-2011 | 34 | .1 | .1 | 15.7 |
|  | 03-JUN-2011 | 25 | .1 | .1 | 15.8 |
|  | 04-JUN-2011 | 19 | .1 | .1 | 15.9 |
|  | 05-JUN-2011 | 30 | .1 | .1 | 15.9 |
|  | 06-JUN-2011 | 21 | .1 | .1 | 16.0 |
|  | 07-JUN-2011 | 28 | .1 | .1 | 16.1 |
|  | 08-JUN-2011 | 23 | .1 | .1 | 16.2 |
|  | 09-JUN-2011 | 22 | .1 | .1 | 16.3 |
|  | 10-JUN-2011 | 52 | .2 | .2 | 16.4 |
|  | 11-JUN-2011 | 14 | .0 | .0 | 16.5 |
|  | 12-JUN-2011 | 36 | .1 | .1 | 16.6 |
|  | 13-JUN-2011 | 16 | .1 | .1 | 16.6 |
|  | 14-JUN-2011 | 10 | .0 | .0 | 16.7 |
|  | 15-JUN-2011 | 37 | .1 | .1 | 16.8 |
|  | 16-JUN-2011 | 18 | .1 | .1 | 16.9 |
|  | 17-JUN-2011 | 17 | .1 | .1 | 16.9 |
|  | 18-JUN-2011 | 12 | .0 | .0 | 17.0 |
|  | 19-JUN-2011 | 16 | .1 | .1 | 17.0 |
|  | 20-JUN-2011 | 54 | .2 | .2 | 17.2 |
|  | 21-JUN-2011 | 12 | .0 | .0 | 17.2 |
|  | 22-JUN-2011 | 12 | .0 | .0 | 17.3 |
|  | 23-JUN-2011 | 10 | .0 | .0 | 17.3 |
|  | 24-JUN-2011 | 13 | .0 | .0 | 17.3 |
|  | 25-JUN-2011 | 19 | .1 | .1 | 17.4 |
|  | 26-JUN-2011 | 14 | .0 | .0 | 17.4 |
|  | 27-JUN-2011 | 11 | .0 | .0 | 17.5 |
|  | 28-JUN-2011 | 15 | .0 | .0 | 17.5 |
|  | 29-JUN-2011 | 12 | .0 | .0 | 17.6 |
|  | 30-JUN-2011 | 15 | .0 | .0 | 17.6 |
|  | 01-JUL-2011 | 57 | .2 | .2 | 17.8 |
|  | 02-JUL-2011 | 26 | .1 | .1 | 17.9 |
|  | 03-JUL-2011 | 34 | .1 | .1 | 18.0 |
|  | 04-JUL-2011 | 9 | .0 | .0 | 18.0 |
|  | 05-JUL-2011 | 40 | .1 | .1 | 18.2 |
|  | 06-JUL-2011 | 31 | .1 | .1 | 18.3 |
|  | 07-JUL-2011 | 19 | .1 | .1 | 18.3 |
|  | 08-JUL-2011 | 33 | .1 | .1 | 18.4 |
|  | 09-JUL-2011 | 26 | .1 | .1 | 18.5 |
|  | 10-JUL-2011 | 52 | .2 | .2 | 18.7 |
|  | 11-JUL-2011 | 15 | .0 | .0 | 18.7 |
|  | 12-JUL-2011 | 24 | .1 | .1 | 18.8 |
|  | 13-JUL-2011 | 20 | .1 | .1 | 18.9 |
|  | 14-JUL-2011 | 18 | .1 | .1 | 18.9 |
|  | 15-JUL-2011 | 44 | .1 | .1 | 19.1 |
|  | 16-JUL-2011 | 15 | .0 | .0 | 19.1 |
|  | 17-JUL-2011 | 18 | .1 | .1 | 19.2 |
|  | 18-JUL-2011 | 19 | .1 | .1 | 19.3 |
|  | 19-JUL-2011 | 25 | .1 | .1 | 19.3 |
|  | 20-JUL-2011 | 50 | .2 | .2 | 19.5 |
|  | 21-JUL-2011 | 21 | .1 | .1 | 19.6 |
|  | 22-JUL-2011 | 14 | .0 | .0 | 19.6 |
|  | 23-JUL-2011 | 12 | .0 | .0 | 19.7 |
|  | 24-JUL-2011 | 18 | .1 | .1 | 19.7 |
|  | 25-JUL-2011 | 19 | .1 | .1 | 19.8 |
|  | 26-JUL-2011 | 10 | .0 | .0 | 19.8 |
|  | 27-JUL-2011 | 21 | .1 | .1 | 19.9 |
|  | 28-JUL-2011 | 34 | .1 | .1 | 20.0 |
|  | 29-JUL-2011 | 32 | .1 | .1 | 20.1 |
|  | 30-JUL-2011 | 33 | .1 | .1 | 20.2 |
|  | 31-JUL-2011 | 6 | .0 | .0 | 20.2 |
|  | 01-AUG-2011 | 93 | .3 | .3 | 20.5 |
|  | 02-AUG-2011 | 62 | .2 | .2 | 20.7 |
|  | 03-AUG-2011 | 55 | .2 | .2 | 20.9 |
|  | 04-AUG-2011 | 31 | .1 | .1 | 21.0 |
|  | 05-AUG-2011 | 53 | .2 | .2 | 21.2 |
|  | 06-AUG-2011 | 32 | .1 | .1 | 21.3 |
|  | 07-AUG-2011 | 49 | .2 | .2 | 21.4 |
|  | 08-AUG-2011 | 27 | .1 | .1 | 21.5 |
|  | 09-AUG-2011 | 55 | .2 | .2 | 21.7 |
|  | 10-AUG-2011 | 112 | .4 | .4 | 22.1 |
|  | 11-AUG-2011 | 27 | .1 | .1 | 22.2 |
|  | 12-AUG-2011 | 56 | .2 | .2 | 22.4 |
|  | 13-AUG-2011 | 29 | .1 | .1 | 22.4 |
|  | 14-AUG-2011 | 35 | .1 | .1 | 22.6 |
|  | 15-AUG-2011 | 52 | .2 | .2 | 22.7 |
|  | 16-AUG-2011 | 26 | .1 | .1 | 22.8 |
|  | 17-AUG-2011 | 34 | .1 | .1 | 22.9 |
|  | 18-AUG-2011 | 27 | .1 | .1 | 23.0 |
|  | 19-AUG-2011 | 31 | .1 | .1 | 23.1 |
|  | 20-AUG-2011 | 96 | .3 | .3 | 23.4 |
|  | 21-AUG-2011 | 15 | .0 | .0 | 23.5 |
|  | 22-AUG-2011 | 38 | .1 | .1 | 23.6 |
|  | 23-AUG-2011 | 14 | .0 | .0 | 23.7 |
|  | 24-AUG-2011 | 33 | .1 | .1 | 23.8 |
|  | 25-AUG-2011 | 44 | .1 | .1 | 23.9 |
|  | 26-AUG-2011 | 28 | .1 | .1 | 24.0 |
|  | 27-AUG-2011 | 23 | .1 | .1 | 24.1 |
|  | 28-AUG-2011 | 24 | .1 | .1 | 24.1 |
|  | 29-AUG-2011 | 26 | .1 | .1 | 24.2 |
|  | 30-AUG-2011 | 28 | .1 | .1 | 24.3 |
|  | 31-AUG-2011 | 16 | .1 | .1 | 24.4 |
|  | 01-SEP-2011 | 135 | .4 | .4 | 24.8 |
|  | 02-SEP-2011 | 95 | .3 | .3 | 25.1 |
|  | 03-SEP-2011 | 63 | .2 | .2 | 25.3 |
|  | 04-SEP-2011 | 35 | .1 | .1 | 25.5 |
|  | 05-SEP-2011 | 71 | .2 | .2 | 25.7 |
|  | 06-SEP-2011 | 41 | .1 | .1 | 25.8 |
|  | 07-SEP-2011 | 67 | .2 | .2 | 26.0 |
|  | 08-SEP-2011 | 49 | .2 | .2 | 26.2 |
|  | 09-SEP-2011 | 38 | .1 | .1 | 26.3 |
|  | 10-SEP-2011 | 100 | .3 | .3 | 26.6 |
|  | 11-SEP-2011 | 37 | .1 | .1 | 26.8 |
|  | 12-SEP-2011 | 64 | .2 | .2 | 27.0 |
|  | 13-SEP-2011 | 16 | .1 | .1 | 27.0 |
|  | 14-SEP-2011 | 43 | .1 | .1 | 27.2 |
|  | 15-SEP-2011 | 48 | .2 | .2 | 27.3 |
|  | 16-SEP-2011 | 24 | .1 | .1 | 27.4 |
|  | 17-SEP-2011 | 18 | .1 | .1 | 27.5 |
|  | 18-SEP-2011 | 25 | .1 | .1 | 27.5 |
|  | 19-SEP-2011 | 18 | .1 | .1 | 27.6 |
|  | 20-SEP-2011 | 33 | .1 | .1 | 27.7 |
|  | 21-SEP-2011 | 21 | .1 | .1 | 27.8 |
|  | 22-SEP-2011 | 20 | .1 | .1 | 27.8 |
|  | 23-SEP-2011 | 13 | .0 | .0 | 27.9 |
|  | 24-SEP-2011 | 13 | .0 | .0 | 27.9 |
|  | 25-SEP-2011 | 18 | .1 | .1 | 28.0 |
|  | 26-SEP-2011 | 13 | .0 | .0 | 28.0 |
|  | 27-SEP-2011 | 11 | .0 | .0 | 28.1 |
|  | 28-SEP-2011 | 17 | .1 | .1 | 28.1 |
|  | 29-SEP-2011 | 11 | .0 | .0 | 28.2 |
|  | 30-SEP-2011 | 17 | .1 | .1 | 28.2 |
|  | 01-OCT-2011 | 27 | .1 | .1 | 28.3 |
|  | 02-OCT-2011 | 22 | .1 | .1 | 28.4 |
|  | 03-OCT-2011 | 19 | .1 | .1 | 28.4 |
|  | 04-OCT-2011 | 13 | .0 | .0 | 28.5 |
|  | 05-OCT-2011 | 28 | .1 | .1 | 28.6 |
|  | 06-OCT-2011 | 20 | .1 | .1 | 28.6 |
|  | 07-OCT-2011 | 22 | .1 | .1 | 28.7 |
|  | 08-OCT-2011 | 20 | .1 | .1 | 28.8 |
|  | 09-OCT-2011 | 11 | .0 | .0 | 28.8 |
|  | 10-OCT-2011 | 24 | .1 | .1 | 28.9 |
|  | 11-OCT-2011 | 6 | .0 | .0 | 28.9 |
|  | 12-OCT-2011 | 25 | .1 | .1 | 29.0 |
|  | 13-OCT-2011 | 14 | .0 | .0 | 29.0 |
|  | 14-OCT-2011 | 15 | .0 | .0 | 29.1 |
|  | 15-OCT-2011 | 15 | .0 | .0 | 29.1 |
|  | 16-OCT-2011 | 10 | .0 | .0 | 29.2 |
|  | 17-OCT-2011 | 12 | .0 | .0 | 29.2 |
|  | 18-OCT-2011 | 18 | .1 | .1 | 29.3 |
|  | 19-OCT-2011 | 14 | .0 | .0 | 29.3 |
|  | 20-OCT-2011 | 36 | .1 | .1 | 29.4 |
|  | 21-OCT-2011 | 12 | .0 | .0 | 29.5 |
|  | 22-OCT-2011 | 16 | .1 | .1 | 29.5 |
|  | 23-OCT-2011 | 8 | .0 | .0 | 29.6 |
|  | 24-OCT-2011 | 9 | .0 | .0 | 29.6 |
|  | 25-OCT-2011 | 12 | .0 | .0 | 29.6 |
|  | 26-OCT-2011 | 10 | .0 | .0 | 29.7 |
|  | 27-OCT-2011 | 11 | .0 | .0 | 29.7 |
|  | 28-OCT-2011 | 15 | .0 | .0 | 29.7 |
|  | 29-OCT-2011 | 9 | .0 | .0 | 29.8 |
|  | 30-OCT-2011 | 10 | .0 | .0 | 29.8 |
|  | 31-OCT-2011 | 8 | .0 | .0 | 29.8 |
|  | 01-NOV-2011 | 22 | .1 | .1 | 29.9 |
|  | 02-NOV-2011 | 25 | .1 | .1 | 30.0 |
|  | 03-NOV-2011 | 29 | .1 | .1 | 30.1 |
|  | 04-NOV-2011 | 15 | .0 | .0 | 30.1 |
|  | 05-NOV-2011 | 22 | .1 | .1 | 30.2 |
|  | 06-NOV-2011 | 8 | .0 | .0 | 30.2 |
|  | 07-NOV-2011 | 17 | .1 | .1 | 30.3 |
|  | 08-NOV-2011 | 13 | .0 | .0 | 30.3 |
|  | 09-NOV-2011 | 9 | .0 | .0 | 30.4 |
|  | 10-NOV-2011 | 23 | .1 | .1 | 30.4 |
|  | 11-NOV-2011 | 21 | .1 | .1 | 30.5 |
|  | 12-NOV-2011 | 7 | .0 | .0 | 30.5 |
|  | 13-NOV-2011 | 9 | .0 | .0 | 30.5 |
|  | 14-NOV-2011 | 15 | .0 | .0 | 30.6 |
|  | 15-NOV-2011 | 15 | .0 | .0 | 30.6 |
|  | 16-NOV-2011 | 9 | .0 | .0 | 30.7 |
|  | 17-NOV-2011 | 8 | .0 | .0 | 30.7 |
|  | 18-NOV-2011 | 10 | .0 | .0 | 30.7 |
|  | 19-NOV-2011 | 6 | .0 | .0 | 30.8 |
|  | 20-NOV-2011 | 28 | .1 | .1 | 30.8 |
|  | 21-NOV-2011 | 9 | .0 | .0 | 30.9 |
|  | 22-NOV-2011 | 9 | .0 | .0 | 30.9 |
|  | 23-NOV-2011 | 9 | .0 | .0 | 30.9 |
|  | 24-NOV-2011 | 5 | .0 | .0 | 30.9 |
|  | 25-NOV-2011 | 15 | .0 | .0 | 31.0 |
|  | 26-NOV-2011 | 12 | .0 | .0 | 31.0 |
|  | 27-NOV-2011 | 13 | .0 | .0 | 31.1 |
|  | 28-NOV-2011 | 16 | .1 | .1 | 31.1 |
|  | 29-NOV-2011 | 10 | .0 | .0 | 31.2 |
|  | 30-NOV-2011 | 9 | .0 | .0 | 31.2 |
|  | 01-DEC-2011 | 39 | .1 | .1 | 31.3 |
|  | 02-DEC-2011 | 23 | .1 | .1 | 31.4 |
|  | 03-DEC-2011 | 16 | .1 | .1 | 31.5 |
|  | 04-DEC-2011 | 12 | .0 | .0 | 31.5 |
|  | 05-DEC-2011 | 14 | .0 | .0 | 31.5 |
|  | 06-DEC-2011 | 12 | .0 | .0 | 31.6 |
|  | 07-DEC-2011 | 14 | .0 | .0 | 31.6 |
|  | 08-DEC-2011 | 16 | .1 | .1 | 31.7 |
|  | 09-DEC-2011 | 12 | .0 | .0 | 31.7 |
|  | 10-DEC-2011 | 25 | .1 | .1 | 31.8 |
|  | 11-DEC-2011 | 12 | .0 | .0 | 31.8 |
|  | 12-DEC-2011 | 18 | .1 | .1 | 31.9 |
|  | 13-DEC-2011 | 17 | .1 | .1 | 31.9 |
|  | 14-DEC-2011 | 7 | .0 | .0 | 32.0 |
|  | 15-DEC-2011 | 15 | .0 | .0 | 32.0 |
|  | 16-DEC-2011 | 21 | .1 | .1 | 32.1 |
|  | 17-DEC-2011 | 16 | .1 | .1 | 32.1 |
|  | 18-DEC-2011 | 9 | .0 | .0 | 32.2 |
|  | 19-DEC-2011 | 14 | .0 | .0 | 32.2 |
|  | 20-DEC-2011 | 26 | .1 | .1 | 32.3 |
|  | 21-DEC-2011 | 9 | .0 | .0 | 32.3 |
|  | 22-DEC-2011 | 9 | .0 | .0 | 32.4 |
|  | 23-DEC-2011 | 16 | .1 | .1 | 32.4 |
|  | 24-DEC-2011 | 7 | .0 | .0 | 32.4 |
|  | 25-DEC-2011 | 13 | .0 | .0 | 32.5 |
|  | 26-DEC-2011 | 24 | .1 | .1 | 32.6 |
|  | 27-DEC-2011 | 11 | .0 | .0 | 32.6 |
|  | 28-DEC-2011 | 17 | .1 | .1 | 32.6 |
|  | 29-DEC-2011 | 17 | .1 | .1 | 32.7 |
|  | 30-DEC-2011 | 29 | .1 | .1 | 32.8 |
|  | 31-DEC-2011 | 3 | .0 | .0 | 32.8 |
|  | 01-JAN-2012 | 173 | .6 | .6 | 33.4 |
|  | 02-JAN-2012 | 62 | .2 | .2 | 33.6 |
|  | 03-JAN-2012 | 35 | .1 | .1 | 33.7 |
|  | 04-JAN-2012 | 25 | .1 | .1 | 33.8 |
|  | 05-JAN-2012 | 51 | .2 | .2 | 33.9 |
|  | 06-JAN-2012 | 32 | .1 | .1 | 34.0 |
|  | 07-JAN-2012 | 30 | .1 | .1 | 34.1 |
|  | 08-JAN-2012 | 25 | .1 | .1 | 34.2 |
|  | 09-JAN-2012 | 43 | .1 | .1 | 34.4 |
|  | 10-JAN-2012 | 70 | .2 | .2 | 34.6 |
|  | 11-JAN-2012 | 23 | .1 | .1 | 34.7 |
|  | 12-JAN-2012 | 44 | .1 | .1 | 34.8 |
|  | 13-JAN-2012 | 12 | .0 | .0 | 34.9 |
|  | 14-JAN-2012 | 23 | .1 | .1 | 34.9 |
|  | 15-JAN-2012 | 25 | .1 | .1 | 35.0 |
|  | 16-JAN-2012 | 13 | .0 | .0 | 35.1 |
|  | 17-JAN-2012 | 17 | .1 | .1 | 35.1 |
|  | 18-JAN-2012 | 15 | .0 | .0 | 35.2 |
|  | 19-JAN-2012 | 13 | .0 | .0 | 35.2 |
|  | 20-JAN-2012 | 41 | .1 | .1 | 35.3 |
|  | 21-JAN-2012 | 13 | .0 | .0 | 35.4 |
|  | 22-JAN-2012 | 17 | .1 | .1 | 35.4 |
|  | 23-JAN-2012 | 17 | .1 | .1 | 35.5 |
|  | 24-JAN-2012 | 12 | .0 | .0 | 35.5 |
|  | 25-JAN-2012 | 18 | .1 | .1 | 35.6 |
|  | 26-JAN-2012 | 16 | .1 | .1 | 35.6 |
|  | 27-JAN-2012 | 17 | .1 | .1 | 35.7 |
|  | 28-JAN-2012 | 13 | .0 | .0 | 35.7 |
|  | 29-JAN-2012 | 14 | .0 | .0 | 35.8 |
|  | 30-JAN-2012 | 15 | .0 | .0 | 35.8 |
|  | 31-JAN-2012 | 10 | .0 | .0 | 35.9 |
|  | 01-FEB-2012 | 95 | .3 | .3 | 36.2 |
|  | 02-FEB-2012 | 42 | .1 | .1 | 36.3 |
|  | 03-FEB-2012 | 24 | .1 | .1 | 36.4 |
|  | 04-FEB-2012 | 33 | .1 | .1 | 36.5 |
|  | 05-FEB-2012 | 34 | .1 | .1 | 36.6 |
|  | 06-FEB-2012 | 29 | .1 | .1 | 36.7 |
|  | 07-FEB-2012 | 29 | .1 | .1 | 36.8 |
|  | 08-FEB-2012 | 37 | .1 | .1 | 36.9 |
|  | 09-FEB-2012 | 37 | .1 | .1 | 37.0 |
|  | 10-FEB-2012 | 71 | .2 | .2 | 37.3 |
|  | 11-FEB-2012 | 23 | .1 | .1 | 37.4 |
|  | 12-FEB-2012 | 31 | .1 | .1 | 37.5 |
|  | 13-FEB-2012 | 23 | .1 | .1 | 37.5 |
|  | 14-FEB-2012 | 27 | .1 | .1 | 37.6 |
|  | 15-FEB-2012 | 29 | .1 | .1 | 37.7 |
|  | 16-FEB-2012 | 13 | .0 | .0 | 37.8 |
|  | 17-FEB-2012 | 17 | .1 | .1 | 37.8 |
|  | 18-FEB-2012 | 19 | .1 | .1 | 37.9 |
|  | 19-FEB-2012 | 26 | .1 | .1 | 38.0 |
|  | 20-FEB-2012 | 75 | .2 | .2 | 38.2 |
|  | 21-FEB-2012 | 20 | .1 | .1 | 38.3 |
|  | 22-FEB-2012 | 23 | .1 | .1 | 38.3 |
|  | 23-FEB-2012 | 15 | .0 | .0 | 38.4 |
|  | 24-FEB-2012 | 21 | .1 | .1 | 38.5 |
|  | 25-FEB-2012 | 26 | .1 | .1 | 38.5 |
|  | 26-FEB-2012 | 13 | .0 | .0 | 38.6 |
|  | 27-FEB-2012 | 16 | .1 | .1 | 38.6 |
|  | 28-FEB-2012 | 33 | .1 | .1 | 38.7 |
|  | 29-FEB-2012 | 10 | .0 | .0 | 38.8 |
|  | 01-MAR-2012 | 134 | .4 | .4 | 39.2 |
|  | 02-MAR-2012 | 76 | .2 | .2 | 39.5 |
|  | 03-MAR-2012 | 48 | .2 | .2 | 39.6 |
|  | 04-MAR-2012 | 47 | .2 | .2 | 39.8 |
|  | 05-MAR-2012 | 42 | .1 | .1 | 39.9 |
|  | 06-MAR-2012 | 45 | .1 | .1 | 40.1 |
|  | 07-MAR-2012 | 34 | .1 | .1 | 40.2 |
|  | 08-MAR-2012 | 40 | .1 | .1 | 40.3 |
|  | 09-MAR-2012 | 35 | .1 | .1 | 40.4 |
|  | 10-MAR-2012 | 74 | .2 | .2 | 40.7 |
|  | 11-MAR-2012 | 28 | .1 | .1 | 40.8 |
|  | 12-MAR-2012 | 38 | .1 | .1 | 40.9 |
|  | 13-MAR-2012 | 18 | .1 | .1 | 40.9 |
|  | 14-MAR-2012 | 16 | .1 | .1 | 41.0 |
|  | 15-MAR-2012 | 30 | .1 | .1 | 41.1 |
|  | 16-MAR-2012 | 23 | .1 | .1 | 41.2 |
|  | 17-MAR-2012 | 14 | .0 | .0 | 41.2 |
|  | 18-MAR-2012 | 15 | .0 | .0 | 41.3 |
|  | 19-MAR-2012 | 11 | .0 | .0 | 41.3 |
|  | 20-MAR-2012 | 32 | .1 | .1 | 41.4 |
|  | 21-MAR-2012 | 9 | .0 | .0 | 41.4 |
|  | 22-MAR-2012 | 22 | .1 | .1 | 41.5 |
|  | 23-MAR-2012 | 10 | .0 | .0 | 41.5 |
|  | 24-MAR-2012 | 6 | .0 | .0 | 41.6 |
|  | 25-MAR-2012 | 15 | .0 | .0 | 41.6 |
|  | 26-MAR-2012 | 14 | .0 | .0 | 41.6 |
|  | 27-MAR-2012 | 9 | .0 | .0 | 41.7 |
|  | 28-MAR-2012 | 15 | .0 | .0 | 41.7 |
|  | 29-MAR-2012 | 14 | .0 | .0 | 41.8 |
|  | 30-MAR-2012 | 19 | .1 | .1 | 41.8 |
|  | 31-MAR-2012 | 8 | .0 | .0 | 41.9 |
|  | 01-APR-2012 | 37 | .1 | .1 | 42.0 |
|  | 02-APR-2012 | 23 | .1 | .1 | 42.1 |
|  | 03-APR-2012 | 21 | .1 | .1 | 42.1 |
|  | 04-APR-2012 | 23 | .1 | .1 | 42.2 |
|  | 05-APR-2012 | 23 | .1 | .1 | 42.3 |
|  | 06-APR-2012 | 21 | .1 | .1 | 42.3 |
|  | 07-APR-2012 | 14 | .0 | .0 | 42.4 |
|  | 08-APR-2012 | 19 | .1 | .1 | 42.5 |
|  | 09-APR-2012 | 12 | .0 | .0 | 42.5 |
|  | 10-APR-2012 | 20 | .1 | .1 | 42.6 |
|  | 11-APR-2012 | 11 | .0 | .0 | 42.6 |
|  | 12-APR-2012 | 33 | .1 | .1 | 42.7 |
|  | 13-APR-2012 | 10 | .0 | .0 | 42.7 |
|  | 14-APR-2012 | 11 | .0 | .0 | 42.8 |
|  | 15-APR-2012 | 25 | .1 | .1 | 42.9 |
|  | 16-APR-2012 | 10 | .0 | .0 | 42.9 |
|  | 17-APR-2012 | 15 | .0 | .0 | 42.9 |
|  | 18-APR-2012 | 16 | .1 | .1 | 43.0 |
|  | 19-APR-2012 | 5 | .0 | .0 | 43.0 |
|  | 20-APR-2012 | 42 | .1 | .1 | 43.1 |
|  | 21-APR-2012 | 8 | .0 | .0 | 43.2 |
|  | 22-APR-2012 | 11 | .0 | .0 | 43.2 |
|  | 23-APR-2012 | 6 | .0 | .0 | 43.2 |
|  | 24-APR-2012 | 12 | .0 | .0 | 43.3 |
|  | 25-APR-2012 | 10 | .0 | .0 | 43.3 |
|  | 26-APR-2012 | 4 | .0 | .0 | 43.3 |
|  | 27-APR-2012 | 14 | .0 | .0 | 43.4 |
|  | 28-APR-2012 | 5 | .0 | .0 | 43.4 |
|  | 29-APR-2012 | 8 | .0 | .0 | 43.4 |
|  | 30-APR-2012 | 5 | .0 | .0 | 43.4 |
|  | 01-MAY-2012 | 63 | .2 | .2 | 43.6 |
|  | 02-MAY-2012 | 29 | .1 | .1 | 43.7 |
|  | 03-MAY-2012 | 24 | .1 | .1 | 43.8 |
|  | 04-MAY-2012 | 21 | .1 | .1 | 43.9 |
|  | 05-MAY-2012 | 22 | .1 | .1 | 43.9 |
|  | 06-MAY-2012 | 18 | .1 | .1 | 44.0 |
|  | 07-MAY-2012 | 16 | .1 | .1 | 44.0 |
|  | 08-MAY-2012 | 17 | .1 | .1 | 44.1 |
|  | 09-MAY-2012 | 19 | .1 | .1 | 44.2 |
|  | 10-MAY-2012 | 38 | .1 | .1 | 44.3 |
|  | 11-MAY-2012 | 19 | .1 | .1 | 44.3 |
|  | 12-MAY-2012 | 16 | .1 | .1 | 44.4 |
|  | 13-MAY-2012 | 12 | .0 | .0 | 44.4 |
|  | 14-MAY-2012 | 13 | .0 | .0 | 44.5 |
|  | 15-MAY-2012 | 18 | .1 | .1 | 44.5 |
|  | 16-MAY-2012 | 15 | .0 | .0 | 44.6 |
|  | 17-MAY-2012 | 13 | .0 | .0 | 44.6 |
|  | 18-MAY-2012 | 13 | .0 | .0 | 44.7 |
|  | 19-MAY-2012 | 13 | .0 | .0 | 44.7 |
|  | 20-MAY-2012 | 28 | .1 | .1 | 44.8 |
|  | 21-MAY-2012 | 11 | .0 | .0 | 44.8 |
|  | 22-MAY-2012 | 17 | .1 | .1 | 44.9 |
|  | 23-MAY-2012 | 11 | .0 | .0 | 44.9 |
|  | 24-MAY-2012 | 7 | .0 | .0 | 45.0 |
|  | 25-MAY-2012 | 15 | .0 | .0 | 45.0 |
|  | 26-MAY-2012 | 12 | .0 | .0 | 45.0 |
|  | 27-MAY-2012 | 11 | .0 | .0 | 45.1 |
|  | 28-MAY-2012 | 10 | .0 | .0 | 45.1 |
|  | 29-MAY-2012 | 7 | .0 | .0 | 45.1 |
|  | 30-MAY-2012 | 13 | .0 | .0 | 45.2 |
|  | 31-MAY-2012 | 7 | .0 | .0 | 45.2 |
|  | 01-JUN-2012 | 63 | .2 | .2 | 45.4 |
|  | 02-JUN-2012 | 43 | .1 | .1 | 45.6 |
|  | 03-JUN-2012 | 25 | .1 | .1 | 45.6 |
|  | 04-JUN-2012 | 14 | .0 | .0 | 45.7 |
|  | 05-JUN-2012 | 21 | .1 | .1 | 45.8 |
|  | 06-JUN-2012 | 21 | .1 | .1 | 45.8 |
|  | 07-JUN-2012 | 17 | .1 | .1 | 45.9 |
|  | 08-JUN-2012 | 19 | .1 | .1 | 45.9 |
|  | 09-JUN-2012 | 14 | .0 | .0 | 46.0 |
|  | 10-JUN-2012 | 51 | .2 | .2 | 46.1 |
|  | 11-JUN-2012 | 18 | .1 | .1 | 46.2 |
|  | 12-JUN-2012 | 27 | .1 | .1 | 46.3 |
|  | 13-JUN-2012 | 16 | .1 | .1 | 46.3 |
|  | 14-JUN-2012 | 15 | .0 | .0 | 46.4 |
|  | 15-JUN-2012 | 45 | .1 | .1 | 46.5 |
|  | 16-JUN-2012 | 9 | .0 | .0 | 46.6 |
|  | 17-JUN-2012 | 12 | .0 | .0 | 46.6 |
|  | 18-JUN-2012 | 10 | .0 | .0 | 46.6 |
|  | 19-JUN-2012 | 12 | .0 | .0 | 46.7 |
|  | 20-JUN-2012 | 49 | .2 | .2 | 46.8 |
|  | 21-JUN-2012 | 16 | .1 | .1 | 46.9 |
|  | 22-JUN-2012 | 9 | .0 | .0 | 46.9 |
|  | 23-JUN-2012 | 13 | .0 | .0 | 47.0 |
|  | 24-JUN-2012 | 13 | .0 | .0 | 47.0 |
|  | 25-JUN-2012 | 19 | .1 | .1 | 47.1 |
|  | 26-JUN-2012 | 11 | .0 | .0 | 47.1 |
|  | 27-JUN-2012 | 10 | .0 | .0 | 47.1 |
|  | 28-JUN-2012 | 17 | .1 | .1 | 47.2 |
|  | 29-JUN-2012 | 8 | .0 | .0 | 47.2 |
|  | 30-JUN-2012 | 22 | .1 | .1 | 47.3 |
|  | 01-JUL-2012 | 60 | .2 | .2 | 47.5 |
|  | 02-JUL-2012 | 38 | .1 | .1 | 47.6 |
|  | 03-JUL-2012 | 27 | .1 | .1 | 47.7 |
|  | 04-JUL-2012 | 18 | .1 | .1 | 47.8 |
|  | 05-JUL-2012 | 38 | .1 | .1 | 47.9 |
|  | 06-JUL-2012 | 18 | .1 | .1 | 47.9 |
|  | 07-JUL-2012 | 31 | .1 | .1 | 48.1 |
|  | 08-JUL-2012 | 35 | .1 | .1 | 48.2 |
|  | 09-JUL-2012 | 29 | .1 | .1 | 48.3 |
|  | 10-JUL-2012 | 53 | .2 | .2 | 48.4 |
|  | 11-JUL-2012 | 26 | .1 | .1 | 48.5 |
|  | 12-JUL-2012 | 25 | .1 | .1 | 48.6 |
|  | 13-JUL-2012 | 23 | .1 | .1 | 48.7 |
|  | 14-JUL-2012 | 21 | .1 | .1 | 48.7 |
|  | 15-JUL-2012 | 28 | .1 | .1 | 48.8 |
|  | 16-JUL-2012 | 19 | .1 | .1 | 48.9 |
|  | 17-JUL-2012 | 12 | .0 | .0 | 48.9 |
|  | 18-JUL-2012 | 7 | .0 | .0 | 49.0 |
|  | 19-JUL-2012 | 8 | .0 | .0 | 49.0 |
|  | 20-JUL-2012 | 32 | .1 | .1 | 49.1 |
|  | 21-JUL-2012 | 12 | .0 | .0 | 49.1 |
|  | 22-JUL-2012 | 16 | .1 | .1 | 49.2 |
|  | 23-JUL-2012 | 20 | .1 | .1 | 49.2 |
|  | 24-JUL-2012 | 6 | .0 | .0 | 49.3 |
|  | 25-JUL-2012 | 29 | .1 | .1 | 49.4 |
|  | 26-JUL-2012 | 12 | .0 | .0 | 49.4 |
|  | 27-JUL-2012 | 12 | .0 | .0 | 49.4 |
|  | 28-JUL-2012 | 25 | .1 | .1 | 49.5 |
|  | 29-JUL-2012 | 14 | .0 | .0 | 49.6 |
|  | 30-JUL-2012 | 28 | .1 | .1 | 49.7 |
|  | 31-JUL-2012 | 10 | .0 | .0 | 49.7 |
|  | 01-AUG-2012 | 85 | .3 | .3 | 50.0 |
|  | 02-AUG-2012 | 53 | .2 | .2 | 50.1 |
|  | 03-AUG-2012 | 36 | .1 | .1 | 50.3 |
|  | 04-AUG-2012 | 19 | .1 | .1 | 50.3 |
|  | 05-AUG-2012 | 40 | .1 | .1 | 50.5 |
|  | 06-AUG-2012 | 24 | .1 | .1 | 50.5 |
|  | 07-AUG-2012 | 26 | .1 | .1 | 50.6 |
|  | 08-AUG-2012 | 21 | .1 | .1 | 50.7 |
|  | 09-AUG-2012 | 50 | .2 | .2 | 50.9 |
|  | 10-AUG-2012 | 81 | .3 | .3 | 51.1 |
|  | 11-AUG-2012 | 20 | .1 | .1 | 51.2 |
|  | 12-AUG-2012 | 39 | .1 | .1 | 51.3 |
|  | 13-AUG-2012 | 15 | .0 | .0 | 51.4 |
|  | 14-AUG-2012 | 21 | .1 | .1 | 51.4 |
|  | 15-AUG-2012 | 35 | .1 | .1 | 51.5 |
|  | 16-AUG-2012 | 11 | .0 | .0 | 51.6 |
|  | 17-AUG-2012 | 31 | .1 | .1 | 51.7 |
|  | 18-AUG-2012 | 30 | .1 | .1 | 51.8 |
|  | 19-AUG-2012 | 15 | .0 | .0 | 51.8 |
|  | 20-AUG-2012 | 47 | .2 | .2 | 52.0 |
|  | 21-AUG-2012 | 19 | .1 | .1 | 52.0 |
|  | 22-AUG-2012 | 22 | .1 | .1 | 52.1 |
|  | 23-AUG-2012 | 12 | .0 | .0 | 52.2 |
|  | 24-AUG-2012 | 13 | .0 | .0 | 52.2 |
|  | 25-AUG-2012 | 45 | .1 | .1 | 52.3 |
|  | 26-AUG-2012 | 20 | .1 | .1 | 52.4 |
|  | 27-AUG-2012 | 18 | .1 | .1 | 52.5 |
|  | 28-AUG-2012 | 27 | .1 | .1 | 52.6 |
|  | 29-AUG-2012 | 26 | .1 | .1 | 52.6 |
|  | 30-AUG-2012 | 20 | .1 | .1 | 52.7 |
|  | 31-AUG-2012 | 11 | .0 | .0 | 52.7 |
|  | 01-SEP-2012 | 119 | .4 | .4 | 53.1 |
|  | 02-SEP-2012 | 52 | .2 | .2 | 53.3 |
|  | 03-SEP-2012 | 30 | .1 | .1 | 53.4 |
|  | 04-SEP-2012 | 36 | .1 | .1 | 53.5 |
|  | 05-SEP-2012 | 39 | .1 | .1 | 53.6 |
|  | 06-SEP-2012 | 30 | .1 | .1 | 53.7 |
|  | 07-SEP-2012 | 41 | .1 | .1 | 53.9 |
|  | 08-SEP-2012 | 35 | .1 | .1 | 54.0 |
|  | 09-SEP-2012 | 36 | .1 | .1 | 54.1 |
|  | 10-SEP-2012 | 76 | .2 | .2 | 54.4 |
|  | 11-SEP-2012 | 25 | .1 | .1 | 54.4 |
|  | 12-SEP-2012 | 33 | .1 | .1 | 54.6 |
|  | 13-SEP-2012 | 20 | .1 | .1 | 54.6 |
|  | 14-SEP-2012 | 31 | .1 | .1 | 54.7 |
|  | 15-SEP-2012 | 35 | .1 | .1 | 54.8 |
|  | 16-SEP-2012 | 27 | .1 | .1 | 54.9 |
|  | 17-SEP-2012 | 20 | .1 | .1 | 55.0 |
|  | 18-SEP-2012 | 24 | .1 | .1 | 55.1 |
|  | 19-SEP-2012 | 17 | .1 | .1 | 55.1 |
|  | 20-SEP-2012 | 42 | .1 | .1 | 55.3 |
|  | 21-SEP-2012 | 13 | .0 | .0 | 55.3 |
|  | 22-SEP-2012 | 11 | .0 | .0 | 55.3 |
|  | 23-SEP-2012 | 19 | .1 | .1 | 55.4 |
|  | 24-SEP-2012 | 8 | .0 | .0 | 55.4 |
|  | 25-SEP-2012 | 20 | .1 | .1 | 55.5 |
|  | 26-SEP-2012 | 17 | .1 | .1 | 55.5 |
|  | 27-SEP-2012 | 11 | .0 | .0 | 55.6 |
|  | 28-SEP-2012 | 20 | .1 | .1 | 55.6 |
|  | 29-SEP-2012 | 10 | .0 | .0 | 55.7 |
|  | 30-SEP-2012 | 19 | .1 | .1 | 55.7 |
|  | 01-OCT-2012 | 54 | .2 | .2 | 55.9 |
|  | 02-OCT-2012 | 45 | .1 | .1 | 56.1 |
|  | 03-OCT-2012 | 23 | .1 | .1 | 56.1 |
|  | 04-OCT-2012 | 24 | .1 | .1 | 56.2 |
|  | 05-OCT-2012 | 36 | .1 | .1 | 56.3 |
|  | 06-OCT-2012 | 22 | .1 | .1 | 56.4 |
|  | 07-OCT-2012 | 25 | .1 | .1 | 56.5 |
|  | 08-OCT-2012 | 21 | .1 | .1 | 56.6 |
|  | 09-OCT-2012 | 33 | .1 | .1 | 56.7 |
|  | 10-OCT-2012 | 45 | .1 | .1 | 56.8 |
|  | 11-OCT-2012 | 21 | .1 | .1 | 56.9 |
|  | 12-OCT-2012 | 32 | .1 | .1 | 57.0 |
|  | 13-OCT-2012 | 10 | .0 | .0 | 57.0 |
|  | 14-OCT-2012 | 12 | .0 | .0 | 57.1 |
|  | 15-OCT-2012 | 30 | .1 | .1 | 57.2 |
|  | 16-OCT-2012 | 22 | .1 | .1 | 57.2 |
|  | 17-OCT-2012 | 24 | .1 | .1 | 57.3 |
|  | 18-OCT-2012 | 15 | .0 | .0 | 57.4 |
|  | 19-OCT-2012 | 19 | .1 | .1 | 57.4 |
|  | 20-OCT-2012 | 47 | .2 | .2 | 57.6 |
|  | 21-OCT-2012 | 15 | .0 | .0 | 57.6 |
|  | 22-OCT-2012 | 18 | .1 | .1 | 57.7 |
|  | 23-OCT-2012 | 11 | .0 | .0 | 57.7 |
|  | 24-OCT-2012 | 20 | .1 | .1 | 57.8 |
|  | 25-OCT-2012 | 24 | .1 | .1 | 57.9 |
|  | 26-OCT-2012 | 12 | .0 | .0 | 57.9 |
|  | 27-OCT-2012 | 14 | .0 | .0 | 57.9 |
|  | 28-OCT-2012 | 19 | .1 | .1 | 58.0 |
|  | 29-OCT-2012 | 24 | .1 | .1 | 58.1 |
|  | 30-OCT-2012 | 14 | .0 | .0 | 58.1 |
|  | 31-OCT-2012 | 6 | .0 | .0 | 58.2 |
|  | 01-NOV-2012 | 58 | .2 | .2 | 58.3 |
|  | 02-NOV-2012 | 43 | .1 | .1 | 58.5 |
|  | 03-NOV-2012 | 14 | .0 | .0 | 58.5 |
|  | 04-NOV-2012 | 16 | .1 | .1 | 58.6 |
|  | 05-NOV-2012 | 43 | .1 | .1 | 58.7 |
|  | 06-NOV-2012 | 27 | .1 | .1 | 58.8 |
|  | 07-NOV-2012 | 32 | .1 | .1 | 58.9 |
|  | 08-NOV-2012 | 22 | .1 | .1 | 59.0 |
|  | 09-NOV-2012 | 33 | .1 | .1 | 59.1 |
|  | 10-NOV-2012 | 58 | .2 | .2 | 59.3 |
|  | 11-NOV-2012 | 18 | .1 | .1 | 59.3 |
|  | 12-NOV-2012 | 24 | .1 | .1 | 59.4 |
|  | 13-NOV-2012 | 20 | .1 | .1 | 59.5 |
|  | 14-NOV-2012 | 17 | .1 | .1 | 59.5 |
|  | 15-NOV-2012 | 28 | .1 | .1 | 59.6 |
|  | 16-NOV-2012 | 18 | .1 | .1 | 59.7 |
|  | 17-NOV-2012 | 16 | .1 | .1 | 59.7 |
|  | 18-NOV-2012 | 18 | .1 | .1 | 59.8 |
|  | 19-NOV-2012 | 14 | .0 | .0 | 59.9 |
|  | 20-NOV-2012 | 39 | .1 | .1 | 60.0 |
|  | 21-NOV-2012 | 15 | .0 | .0 | 60.0 |
|  | 22-NOV-2012 | 22 | .1 | .1 | 60.1 |
|  | 23-NOV-2012 | 10 | .0 | .0 | 60.1 |
|  | 24-NOV-2012 | 18 | .1 | .1 | 60.2 |
|  | 25-NOV-2012 | 23 | .1 | .1 | 60.3 |
|  | 26-NOV-2012 | 16 | .1 | .1 | 60.3 |
|  | 27-NOV-2012 | 17 | .1 | .1 | 60.4 |
|  | 28-NOV-2012 | 25 | .1 | .1 | 60.5 |
|  | 29-NOV-2012 | 10 | .0 | .0 | 60.5 |
|  | 30-NOV-2012 | 20 | .1 | .1 | 60.6 |
|  | 01-DEC-2012 | 84 | .3 | .3 | 60.8 |
|  | 02-DEC-2012 | 40 | .1 | .1 | 61.0 |
|  | 03-DEC-2012 | 25 | .1 | .1 | 61.0 |
|  | 04-DEC-2012 | 15 | .0 | .0 | 61.1 |
|  | 05-DEC-2012 | 21 | .1 | .1 | 61.2 |
|  | 06-DEC-2012 | 23 | .1 | .1 | 61.2 |
|  | 07-DEC-2012 | 25 | .1 | .1 | 61.3 |
|  | 08-DEC-2012 | 18 | .1 | .1 | 61.4 |
|  | 09-DEC-2012 | 28 | .1 | .1 | 61.5 |
|  | 10-DEC-2012 | 44 | .1 | .1 | 61.6 |
|  | 11-DEC-2012 | 19 | .1 | .1 | 61.7 |
|  | 12-DEC-2012 | 31 | .1 | .1 | 61.8 |
|  | 13-DEC-2012 | 12 | .0 | .0 | 61.8 |
|  | 14-DEC-2012 | 20 | .1 | .1 | 61.9 |
|  | 15-DEC-2012 | 37 | .1 | .1 | 62.0 |
|  | 16-DEC-2012 | 27 | .1 | .1 | 62.1 |
|  | 17-DEC-2012 | 17 | .1 | .1 | 62.1 |
|  | 18-DEC-2012 | 11 | .0 | .0 | 62.2 |
|  | 19-DEC-2012 | 10 | .0 | .0 | 62.2 |
|  | 20-DEC-2012 | 31 | .1 | .1 | 62.3 |
|  | 21-DEC-2012 | 10 | .0 | .0 | 62.4 |
|  | 22-DEC-2012 | 18 | .1 | .1 | 62.4 |
|  | 23-DEC-2012 | 10 | .0 | .0 | 62.4 |
|  | 24-DEC-2012 | 10 | .0 | .0 | 62.5 |
|  | 25-DEC-2012 | 33 | .1 | .1 | 62.6 |
|  | 26-DEC-2012 | 17 | .1 | .1 | 62.6 |
|  | 27-DEC-2012 | 13 | .0 | .0 | 62.7 |
|  | 28-DEC-2012 | 18 | .1 | .1 | 62.7 |
|  | 29-DEC-2012 | 13 | .0 | .0 | 62.8 |
|  | 30-DEC-2012 | 16 | .1 | .1 | 62.8 |
|  | 31-DEC-2012 | 20 | .1 | .1 | 62.9 |
|  | 01-JAN-2013 | 165 | .5 | .5 | 63.4 |
|  | 02-JAN-2013 | 82 | .3 | .3 | 63.7 |
|  | 03-JAN-2013 | 48 | .2 | .2 | 63.9 |
|  | 04-JAN-2013 | 27 | .1 | .1 | 64.0 |
|  | 05-JAN-2013 | 38 | .1 | .1 | 64.1 |
|  | 06-JAN-2013 | 18 | .1 | .1 | 64.1 |
|  | 07-JAN-2013 | 34 | .1 | .1 | 64.2 |
|  | 08-JAN-2013 | 19 | .1 | .1 | 64.3 |
|  | 09-JAN-2013 | 31 | .1 | .1 | 64.4 |
|  | 10-JAN-2013 | 65 | .2 | .2 | 64.6 |
|  | 11-JAN-2013 | 20 | .1 | .1 | 64.7 |
|  | 12-JAN-2013 | 33 | .1 | .1 | 64.8 |
|  | 13-JAN-2013 | 20 | .1 | .1 | 64.9 |
|  | 14-JAN-2013 | 16 | .1 | .1 | 64.9 |
|  | 15-JAN-2013 | 32 | .1 | .1 | 65.0 |
|  | 16-JAN-2013 | 19 | .1 | .1 | 65.1 |
|  | 17-JAN-2013 | 10 | .0 | .0 | 65.1 |
|  | 18-JAN-2013 | 15 | .0 | .0 | 65.2 |
|  | 19-JAN-2013 | 16 | .1 | .1 | 65.2 |
|  | 20-JAN-2013 | 54 | .2 | .2 | 65.4 |
|  | 21-JAN-2013 | 16 | .1 | .1 | 65.4 |
|  | 22-JAN-2013 | 14 | .0 | .0 | 65.5 |
|  | 23-JAN-2013 | 16 | .1 | .1 | 65.5 |
|  | 24-JAN-2013 | 14 | .0 | .0 | 65.6 |
|  | 25-JAN-2013 | 34 | .1 | .1 | 65.7 |
|  | 26-JAN-2013 | 14 | .0 | .0 | 65.7 |
|  | 27-JAN-2013 | 13 | .0 | .0 | 65.8 |
|  | 28-JAN-2013 | 17 | .1 | .1 | 65.8 |
|  | 29-JAN-2013 | 9 | .0 | .0 | 65.9 |
|  | 30-JAN-2013 | 9 | .0 | .0 | 65.9 |
|  | 31-JAN-2013 | 6 | .0 | .0 | 65.9 |
|  | 01-FEB-2013 | 81 | .3 | .3 | 66.2 |
|  | 02-FEB-2013 | 46 | .2 | .2 | 66.3 |
|  | 03-FEB-2013 | 26 | .1 | .1 | 66.4 |
|  | 04-FEB-2013 | 15 | .0 | .0 | 66.5 |
|  | 05-FEB-2013 | 24 | .1 | .1 | 66.6 |
|  | 06-FEB-2013 | 14 | .0 | .0 | 66.6 |
|  | 07-FEB-2013 | 21 | .1 | .1 | 66.7 |
|  | 08-FEB-2013 | 14 | .0 | .0 | 66.7 |
|  | 09-FEB-2013 | 21 | .1 | .1 | 66.8 |
|  | 10-FEB-2013 | 55 | .2 | .2 | 67.0 |
|  | 11-FEB-2013 | 14 | .0 | .0 | 67.0 |
|  | 12-FEB-2013 | 21 | .1 | .1 | 67.1 |
|  | 13-FEB-2013 | 21 | .1 | .1 | 67.1 |
|  | 14-FEB-2013 | 27 | .1 | .1 | 67.2 |
|  | 15-FEB-2013 | 31 | .1 | .1 | 67.3 |
|  | 16-FEB-2013 | 18 | .1 | .1 | 67.4 |
|  | 17-FEB-2013 | 17 | .1 | .1 | 67.5 |
|  | 18-FEB-2013 | 22 | .1 | .1 | 67.5 |
|  | 19-FEB-2013 | 18 | .1 | .1 | 67.6 |
|  | 20-FEB-2013 | 41 | .1 | .1 | 67.7 |
|  | 21-FEB-2013 | 12 | .0 | .0 | 67.8 |
|  | 22-FEB-2013 | 16 | .1 | .1 | 67.8 |
|  | 23-FEB-2013 | 11 | .0 | .0 | 67.8 |
|  | 24-FEB-2013 | 14 | .0 | .0 | 67.9 |
|  | 25-FEB-2013 | 28 | .1 | .1 | 68.0 |
|  | 26-FEB-2013 | 11 | .0 | .0 | 68.0 |
|  | 27-FEB-2013 | 7 | .0 | .0 | 68.0 |
|  | 28-FEB-2013 | 35 | .1 | .1 | 68.2 |
|  | 01-MAR-2013 | 97 | .3 | .3 | 68.5 |
|  | 02-MAR-2013 | 49 | .2 | .2 | 68.6 |
|  | 03-MAR-2013 | 21 | .1 | .1 | 68.7 |
|  | 04-MAR-2013 | 23 | .1 | .1 | 68.8 |
|  | 05-MAR-2013 | 31 | .1 | .1 | 68.9 |
|  | 06-MAR-2013 | 22 | .1 | .1 | 68.9 |
|  | 07-MAR-2013 | 23 | .1 | .1 | 69.0 |
|  | 08-MAR-2013 | 23 | .1 | .1 | 69.1 |
|  | 09-MAR-2013 | 22 | .1 | .1 | 69.2 |
|  | 10-MAR-2013 | 44 | .1 | .1 | 69.3 |
|  | 11-MAR-2013 | 17 | .1 | .1 | 69.4 |
|  | 12-MAR-2013 | 23 | .1 | .1 | 69.4 |
|  | 13-MAR-2013 | 25 | .1 | .1 | 69.5 |
|  | 14-MAR-2013 | 20 | .1 | .1 | 69.6 |
|  | 15-MAR-2013 | 30 | .1 | .1 | 69.7 |
|  | 16-MAR-2013 | 21 | .1 | .1 | 69.8 |
|  | 17-MAR-2013 | 15 | .0 | .0 | 69.8 |
|  | 18-MAR-2013 | 14 | .0 | .0 | 69.9 |
|  | 19-MAR-2013 | 13 | .0 | .0 | 69.9 |
|  | 20-MAR-2013 | 38 | .1 | .1 | 70.0 |
|  | 21-MAR-2013 | 9 | .0 | .0 | 70.1 |
|  | 22-MAR-2013 | 19 | .1 | .1 | 70.1 |
|  | 23-MAR-2013 | 11 | .0 | .0 | 70.2 |
|  | 24-MAR-2013 | 19 | .1 | .1 | 70.2 |
|  | 25-MAR-2013 | 16 | .1 | .1 | 70.3 |
|  | 26-MAR-2013 | 12 | .0 | .0 | 70.3 |
|  | 27-MAR-2013 | 14 | .0 | .0 | 70.3 |
|  | 28-MAR-2013 | 20 | .1 | .1 | 70.4 |
|  | 29-MAR-2013 | 16 | .1 | .1 | 70.5 |
|  | 30-MAR-2013 | 16 | .1 | .1 | 70.5 |
|  | 31-MAR-2013 | 9 | .0 | .0 | 70.5 |
|  | 01-APR-2013 | 50 | .2 | .2 | 70.7 |
|  | 02-APR-2013 | 41 | .1 | .1 | 70.8 |
|  | 03-APR-2013 | 22 | .1 | .1 | 70.9 |
|  | 04-APR-2013 | 23 | .1 | .1 | 71.0 |
|  | 05-APR-2013 | 31 | .1 | .1 | 71.1 |
|  | 06-APR-2013 | 12 | .0 | .0 | 71.1 |
|  | 07-APR-2013 | 21 | .1 | .1 | 71.2 |
|  | 08-APR-2013 | 22 | .1 | .1 | 71.3 |
|  | 09-APR-2013 | 15 | .0 | .0 | 71.3 |
|  | 10-APR-2013 | 54 | .2 | .2 | 71.5 |
|  | 11-APR-2013 | 18 | .1 | .1 | 71.6 |
|  | 12-APR-2013 | 23 | .1 | .1 | 71.6 |
|  | 13-APR-2013 | 15 | .0 | .0 | 71.7 |
|  | 14-APR-2013 | 15 | .0 | .0 | 71.7 |
|  | 15-APR-2013 | 17 | .1 | .1 | 71.8 |
|  | 16-APR-2013 | 12 | .0 | .0 | 71.8 |
|  | 17-APR-2013 | 13 | .0 | .0 | 71.9 |
|  | 18-APR-2013 | 12 | .0 | .0 | 71.9 |
|  | 19-APR-2013 | 11 | .0 | .0 | 71.9 |
|  | 20-APR-2013 | 44 | .1 | .1 | 72.1 |
|  | 21-APR-2013 | 9 | .0 | .0 | 72.1 |
|  | 22-APR-2013 | 10 | .0 | .0 | 72.2 |
|  | 23-APR-2013 | 11 | .0 | .0 | 72.2 |
|  | 24-APR-2013 | 10 | .0 | .0 | 72.2 |
|  | 25-APR-2013 | 16 | .1 | .1 | 72.3 |
|  | 26-APR-2013 | 11 | .0 | .0 | 72.3 |
|  | 27-APR-2013 | 12 | .0 | .0 | 72.3 |
|  | 28-APR-2013 | 15 | .0 | .0 | 72.4 |
|  | 29-APR-2013 | 16 | .1 | .1 | 72.5 |
|  | 30-APR-2013 | 26 | .1 | .1 | 72.5 |
|  | 01-MAY-2013 | 76 | .2 | .2 | 72.8 |
|  | 02-MAY-2013 | 38 | .1 | .1 | 72.9 |
|  | 03-MAY-2013 | 24 | .1 | .1 | 73.0 |
|  | 04-MAY-2013 | 19 | .1 | .1 | 73.1 |
|  | 05-MAY-2013 | 28 | .1 | .1 | 73.1 |
|  | 06-MAY-2013 | 17 | .1 | .1 | 73.2 |
|  | 07-MAY-2013 | 20 | .1 | .1 | 73.3 |
|  | 08-MAY-2013 | 14 | .0 | .0 | 73.3 |
|  | 09-MAY-2013 | 22 | .1 | .1 | 73.4 |
|  | 10-MAY-2013 | 38 | .1 | .1 | 73.5 |
|  | 11-MAY-2013 | 15 | .0 | .0 | 73.6 |
|  | 12-MAY-2013 | 28 | .1 | .1 | 73.6 |
|  | 13-MAY-2013 | 12 | .0 | .0 | 73.7 |
|  | 14-MAY-2013 | 12 | .0 | .0 | 73.7 |
|  | 15-MAY-2013 | 30 | .1 | .1 | 73.8 |
|  | 16-MAY-2013 | 13 | .0 | .0 | 73.9 |
|  | 17-MAY-2013 | 14 | .0 | .0 | 73.9 |
|  | 18-MAY-2013 | 21 | .1 | .1 | 74.0 |
|  | 19-MAY-2013 | 8 | .0 | .0 | 74.0 |
|  | 20-MAY-2013 | 36 | .1 | .1 | 74.1 |
|  | 21-MAY-2013 | 9 | .0 | .0 | 74.2 |
|  | 22-MAY-2013 | 15 | .0 | .0 | 74.2 |
|  | 23-MAY-2013 | 7 | .0 | .0 | 74.2 |
|  | 24-MAY-2013 | 11 | .0 | .0 | 74.3 |
|  | 25-MAY-2013 | 23 | .1 | .1 | 74.3 |
|  | 26-MAY-2013 | 10 | .0 | .0 | 74.4 |
|  | 27-MAY-2013 | 7 | .0 | .0 | 74.4 |
|  | 28-MAY-2013 | 17 | .1 | .1 | 74.4 |
|  | 29-MAY-2013 | 10 | .0 | .0 | 74.5 |
|  | 30-MAY-2013 | 14 | .0 | .0 | 74.5 |
|  | 31-MAY-2013 | 2 | .0 | .0 | 74.5 |
|  | 01-JUN-2013 | 50 | .2 | .2 | 74.7 |
|  | 02-JUN-2013 | 33 | .1 | .1 | 74.8 |
|  | 03-JUN-2013 | 24 | .1 | .1 | 74.9 |
|  | 04-JUN-2013 | 22 | .1 | .1 | 75.0 |
|  | 05-JUN-2013 | 22 | .1 | .1 | 75.0 |
|  | 06-JUN-2013 | 27 | .1 | .1 | 75.1 |
|  | 07-JUN-2013 | 24 | .1 | .1 | 75.2 |
|  | 08-JUN-2013 | 13 | .0 | .0 | 75.2 |
|  | 09-JUN-2013 | 25 | .1 | .1 | 75.3 |
|  | 10-JUN-2013 | 30 | .1 | .1 | 75.4 |
|  | 11-JUN-2013 | 8 | .0 | .0 | 75.4 |
|  | 12-JUN-2013 | 18 | .1 | .1 | 75.5 |
|  | 13-JUN-2013 | 13 | .0 | .0 | 75.5 |
|  | 14-JUN-2013 | 11 | .0 | .0 | 75.6 |
|  | 15-JUN-2013 | 29 | .1 | .1 | 75.7 |
|  | 16-JUN-2013 | 8 | .0 | .0 | 75.7 |
|  | 17-JUN-2013 | 13 | .0 | .0 | 75.7 |
|  | 18-JUN-2013 | 17 | .1 | .1 | 75.8 |
|  | 19-JUN-2013 | 8 | .0 | .0 | 75.8 |
|  | 20-JUN-2013 | 40 | .1 | .1 | 76.0 |
|  | 21-JUN-2013 | 6 | .0 | .0 | 76.0 |
|  | 22-JUN-2013 | 11 | .0 | .0 | 76.0 |
|  | 23-JUN-2013 | 7 | .0 | .0 | 76.0 |
|  | 24-JUN-2013 | 10 | .0 | .0 | 76.1 |
|  | 25-JUN-2013 | 18 | .1 | .1 | 76.1 |
|  | 26-JUN-2013 | 11 | .0 | .0 | 76.2 |
|  | 27-JUN-2013 | 8 | .0 | .0 | 76.2 |
|  | 28-JUN-2013 | 12 | .0 | .0 | 76.2 |
|  | 29-JUN-2013 | 11 | .0 | .0 | 76.3 |
|  | 30-JUN-2013 | 12 | .0 | .0 | 76.3 |
|  | 01-JUL-2013 | 44 | .1 | .1 | 76.4 |
|  | 02-JUL-2013 | 22 | .1 | .1 | 76.5 |
|  | 03-JUL-2013 | 14 | .0 | .0 | 76.6 |
|  | 04-JUL-2013 | 14 | .0 | .0 | 76.6 |
|  | 05-JUL-2013 | 42 | .1 | .1 | 76.7 |
|  | 06-JUL-2013 | 20 | .1 | .1 | 76.8 |
|  | 07-JUL-2013 | 15 | .0 | .0 | 76.9 |
|  | 08-JUL-2013 | 12 | .0 | .0 | 76.9 |
|  | 09-JUL-2013 | 18 | .1 | .1 | 77.0 |
|  | 10-JUL-2013 | 37 | .1 | .1 | 77.1 |
|  | 11-JUL-2013 | 14 | .0 | .0 | 77.1 |
|  | 12-JUL-2013 | 22 | .1 | .1 | 77.2 |
|  | 13-JUL-2013 | 10 | .0 | .0 | 77.2 |
|  | 14-JUL-2013 | 9 | .0 | .0 | 77.3 |
|  | 15-JUL-2013 | 31 | .1 | .1 | 77.4 |
|  | 16-JUL-2013 | 14 | .0 | .0 | 77.4 |
|  | 17-JUL-2013 | 10 | .0 | .0 | 77.4 |
|  | 18-JUL-2013 | 18 | .1 | .1 | 77.5 |
|  | 19-JUL-2013 | 19 | .1 | .1 | 77.6 |
|  | 20-JUL-2013 | 30 | .1 | .1 | 77.7 |
|  | 21-JUL-2013 | 10 | .0 | .0 | 77.7 |
|  | 22-JUL-2013 | 15 | .0 | .0 | 77.7 |
|  | 23-JUL-2013 | 10 | .0 | .0 | 77.8 |
|  | 24-JUL-2013 | 12 | .0 | .0 | 77.8 |
|  | 25-JUL-2013 | 18 | .1 | .1 | 77.9 |
|  | 26-JUL-2013 | 8 | .0 | .0 | 77.9 |
|  | 27-JUL-2013 | 15 | .0 | .0 | 77.9 |
|  | 28-JUL-2013 | 21 | .1 | .1 | 78.0 |
|  | 29-JUL-2013 | 15 | .0 | .0 | 78.1 |
|  | 30-JUL-2013 | 15 | .0 | .0 | 78.1 |
|  | 31-JUL-2013 | 13 | .0 | .0 | 78.2 |
|  | 01-AUG-2013 | 41 | .1 | .1 | 78.3 |
|  | 02-AUG-2013 | 29 | .1 | .1 | 78.4 |
|  | 03-AUG-2013 | 25 | .1 | .1 | 78.5 |
|  | 04-AUG-2013 | 15 | .0 | .0 | 78.5 |
|  | 05-AUG-2013 | 23 | .1 | .1 | 78.6 |
|  | 06-AUG-2013 | 13 | .0 | .0 | 78.6 |
|  | 07-AUG-2013 | 20 | .1 | .1 | 78.7 |
|  | 08-AUG-2013 | 21 | .1 | .1 | 78.8 |
|  | 09-AUG-2013 | 17 | .1 | .1 | 78.8 |
|  | 10-AUG-2013 | 48 | .2 | .2 | 79.0 |
|  | 11-AUG-2013 | 19 | .1 | .1 | 79.0 |
|  | 12-AUG-2013 | 10 | .0 | .0 | 79.1 |
|  | 13-AUG-2013 | 12 | .0 | .0 | 79.1 |
|  | 14-AUG-2013 | 13 | .0 | .0 | 79.2 |
|  | 15-AUG-2013 | 18 | .1 | .1 | 79.2 |
|  | 16-AUG-2013 | 15 | .0 | .0 | 79.3 |
|  | 17-AUG-2013 | 13 | .0 | .0 | 79.3 |
|  | 18-AUG-2013 | 15 | .0 | .0 | 79.4 |
|  | 19-AUG-2013 | 18 | .1 | .1 | 79.4 |
|  | 20-AUG-2013 | 34 | .1 | .1 | 79.5 |
|  | 21-AUG-2013 | 10 | .0 | .0 | 79.6 |
|  | 22-AUG-2013 | 19 | .1 | .1 | 79.6 |
|  | 23-AUG-2013 | 11 | .0 | .0 | 79.7 |
|  | 24-AUG-2013 | 13 | .0 | .0 | 79.7 |
|  | 25-AUG-2013 | 20 | .1 | .1 | 79.8 |
|  | 26-AUG-2013 | 16 | .1 | .1 | 79.8 |
|  | 27-AUG-2013 | 17 | .1 | .1 | 79.9 |
|  | 28-AUG-2013 | 20 | .1 | .1 | 79.9 |
|  | 29-AUG-2013 | 10 | .0 | .0 | 80.0 |
|  | 30-AUG-2013 | 15 | .0 | .0 | 80.0 |
|  | 31-AUG-2013 | 5 | .0 | .0 | 80.0 |
|  | 01-SEP-2013 | 34 | .1 | .1 | 80.2 |
|  | 02-SEP-2013 | 23 | .1 | .1 | 80.2 |
|  | 03-SEP-2013 | 26 | .1 | .1 | 80.3 |
|  | 04-SEP-2013 | 13 | .0 | .0 | 80.4 |
|  | 05-SEP-2013 | 21 | .1 | .1 | 80.4 |
|  | 06-SEP-2013 | 9 | .0 | .0 | 80.5 |
|  | 07-SEP-2013 | 19 | .1 | .1 | 80.5 |
|  | 08-SEP-2013 | 18 | .1 | .1 | 80.6 |
|  | 09-SEP-2013 | 19 | .1 | .1 | 80.6 |
|  | 10-SEP-2013 | 39 | .1 | .1 | 80.8 |
|  | 11-SEP-2013 | 11 | .0 | .0 | 80.8 |
|  | 12-SEP-2013 | 13 | .0 | .0 | 80.8 |
|  | 13-SEP-2013 | 16 | .1 | .1 | 80.9 |
|  | 14-SEP-2013 | 14 | .0 | .0 | 80.9 |
|  | 15-SEP-2013 | 19 | .1 | .1 | 81.0 |
|  | 16-SEP-2013 | 17 | .1 | .1 | 81.1 |
|  | 17-SEP-2013 | 16 | .1 | .1 | 81.1 |
|  | 18-SEP-2013 | 18 | .1 | .1 | 81.2 |
|  | 19-SEP-2013 | 10 | .0 | .0 | 81.2 |
|  | 20-SEP-2013 | 26 | .1 | .1 | 81.3 |
|  | 21-SEP-2013 | 9 | .0 | .0 | 81.3 |
|  | 22-SEP-2013 | 7 | .0 | .0 | 81.3 |
|  | 23-SEP-2013 | 14 | .0 | .0 | 81.4 |
|  | 24-SEP-2013 | 9 | .0 | .0 | 81.4 |
|  | 25-SEP-2013 | 21 | .1 | .1 | 81.5 |
|  | 26-SEP-2013 | 10 | .0 | .0 | 81.5 |
|  | 27-SEP-2013 | 7 | .0 | .0 | 81.5 |
|  | 28-SEP-2013 | 15 | .0 | .0 | 81.6 |
|  | 29-SEP-2013 | 16 | .1 | .1 | 81.6 |
|  | 30-SEP-2013 | 14 | .0 | .0 | 81.7 |
|  | 01-OCT-2013 | 26 | .1 | .1 | 81.8 |
|  | 02-OCT-2013 | 30 | .1 | .1 | 81.9 |
|  | 03-OCT-2013 | 18 | .1 | .1 | 81.9 |
|  | 04-OCT-2013 | 13 | .0 | .0 | 82.0 |
|  | 05-OCT-2013 | 31 | .1 | .1 | 82.1 |
|  | 06-OCT-2013 | 18 | .1 | .1 | 82.1 |
|  | 07-OCT-2013 | 22 | .1 | .1 | 82.2 |
|  | 08-OCT-2013 | 24 | .1 | .1 | 82.3 |
|  | 09-OCT-2013 | 27 | .1 | .1 | 82.4 |
|  | 10-OCT-2013 | 54 | .2 | .2 | 82.5 |
|  | 11-OCT-2013 | 20 | .1 | .1 | 82.6 |
|  | 12-OCT-2013 | 13 | .0 | .0 | 82.7 |
|  | 13-OCT-2013 | 18 | .1 | .1 | 82.7 |
|  | 14-OCT-2013 | 14 | .0 | .0 | 82.8 |
|  | 15-OCT-2013 | 30 | .1 | .1 | 82.9 |
|  | 16-OCT-2013 | 20 | .1 | .1 | 82.9 |
|  | 17-OCT-2013 | 26 | .1 | .1 | 83.0 |
|  | 18-OCT-2013 | 11 | .0 | .0 | 83.0 |
|  | 19-OCT-2013 | 19 | .1 | .1 | 83.1 |
|  | 20-OCT-2013 | 31 | .1 | .1 | 83.2 |
|  | 21-OCT-2013 | 15 | .0 | .0 | 83.3 |
|  | 22-OCT-2013 | 14 | .0 | .0 | 83.3 |
|  | 23-OCT-2013 | 20 | .1 | .1 | 83.4 |
|  | 24-OCT-2013 | 11 | .0 | .0 | 83.4 |
|  | 25-OCT-2013 | 34 | .1 | .1 | 83.5 |
|  | 26-OCT-2013 | 15 | .0 | .0 | 83.6 |
|  | 27-OCT-2013 | 16 | .1 | .1 | 83.6 |
|  | 28-OCT-2013 | 26 | .1 | .1 | 83.7 |
|  | 29-OCT-2013 | 22 | .1 | .1 | 83.8 |
|  | 30-OCT-2013 | 21 | .1 | .1 | 83.8 |
|  | 31-OCT-2013 | 11 | .0 | .0 | 83.9 |
|  | 01-NOV-2013 | 25 | .1 | .1 | 84.0 |
|  | 02-NOV-2013 | 28 | .1 | .1 | 84.1 |
|  | 03-NOV-2013 | 22 | .1 | .1 | 84.1 |
|  | 04-NOV-2013 | 17 | .1 | .1 | 84.2 |
|  | 05-NOV-2013 | 31 | .1 | .1 | 84.3 |
|  | 06-NOV-2013 | 21 | .1 | .1 | 84.4 |
|  | 07-NOV-2013 | 15 | .0 | .0 | 84.4 |
|  | 08-NOV-2013 | 17 | .1 | .1 | 84.5 |
|  | 09-NOV-2013 | 19 | .1 | .1 | 84.5 |
|  | 10-NOV-2013 | 29 | .1 | .1 | 84.6 |
|  | 11-NOV-2013 | 19 | .1 | .1 | 84.7 |
|  | 12-NOV-2013 | 22 | .1 | .1 | 84.7 |
|  | 13-NOV-2013 | 20 | .1 | .1 | 84.8 |
|  | 14-NOV-2013 | 11 | .0 | .0 | 84.8 |
|  | 15-NOV-2013 | 31 | .1 | .1 | 85.0 |
|  | 16-NOV-2013 | 21 | .1 | .1 | 85.0 |
|  | 17-NOV-2013 | 12 | .0 | .0 | 85.1 |
|  | 18-NOV-2013 | 11 | .0 | .0 | 85.1 |
|  | 19-NOV-2013 | 8 | .0 | .0 | 85.1 |
|  | 20-NOV-2013 | 32 | .1 | .1 | 85.2 |
|  | 21-NOV-2013 | 16 | .1 | .1 | 85.3 |
|  | 22-NOV-2013 | 23 | .1 | .1 | 85.4 |
|  | 23-NOV-2013 | 22 | .1 | .1 | 85.4 |
|  | 24-NOV-2013 | 20 | .1 | .1 | 85.5 |
|  | 25-NOV-2013 | 30 | .1 | .1 | 85.6 |
|  | 26-NOV-2013 | 16 | .1 | .1 | 85.6 |
|  | 27-NOV-2013 | 20 | .1 | .1 | 85.7 |
|  | 28-NOV-2013 | 17 | .1 | .1 | 85.8 |
|  | 29-NOV-2013 | 25 | .1 | .1 | 85.8 |
|  | 30-NOV-2013 | 23 | .1 | .1 | 85.9 |
|  | 01-DEC-2013 | 34 | .1 | .1 | 86.0 |
|  | 02-DEC-2013 | 25 | .1 | .1 | 86.1 |
|  | 03-DEC-2013 | 21 | .1 | .1 | 86.2 |
|  | 04-DEC-2013 | 18 | .1 | .1 | 86.2 |
|  | 05-DEC-2013 | 31 | .1 | .1 | 86.3 |
|  | 06-DEC-2013 | 16 | .1 | .1 | 86.4 |
|  | 07-DEC-2013 | 19 | .1 | .1 | 86.5 |
|  | 08-DEC-2013 | 14 | .0 | .0 | 86.5 |
|  | 09-DEC-2013 | 16 | .1 | .1 | 86.6 |
|  | 10-DEC-2013 | 27 | .1 | .1 | 86.6 |
|  | 11-DEC-2013 | 12 | .0 | .0 | 86.7 |
|  | 12-DEC-2013 | 20 | .1 | .1 | 86.7 |
|  | 13-DEC-2013 | 22 | .1 | .1 | 86.8 |
|  | 14-DEC-2013 | 23 | .1 | .1 | 86.9 |
|  | 15-DEC-2013 | 24 | .1 | .1 | 87.0 |
|  | 16-DEC-2013 | 26 | .1 | .1 | 87.1 |
|  | 17-DEC-2013 | 19 | .1 | .1 | 87.1 |
|  | 18-DEC-2013 | 16 | .1 | .1 | 87.2 |
|  | 19-DEC-2013 | 10 | .0 | .0 | 87.2 |
|  | 20-DEC-2013 | 36 | .1 | .1 | 87.3 |
|  | 21-DEC-2013 | 12 | .0 | .0 | 87.4 |
|  | 22-DEC-2013 | 20 | .1 | .1 | 87.4 |
|  | 23-DEC-2013 | 13 | .0 | .0 | 87.5 |
|  | 24-DEC-2013 | 18 | .1 | .1 | 87.5 |
|  | 25-DEC-2013 | 23 | .1 | .1 | 87.6 |
|  | 26-DEC-2013 | 17 | .1 | .1 | 87.7 |
|  | 27-DEC-2013 | 14 | .0 | .0 | 87.7 |
|  | 28-DEC-2013 | 20 | .1 | .1 | 87.8 |
|  | 29-DEC-2013 | 19 | .1 | .1 | 87.8 |
|  | 30-DEC-2013 | 12 | .0 | .0 | 87.9 |
|  | 31-DEC-2013 | 5 | .0 | .0 | 87.9 |
|  | 01-JAN-2014 | 58 | .2 | .2 | 88.1 |
|  | 02-JAN-2014 | 30 | .1 | .1 | 88.2 |
|  | 03-JAN-2014 | 26 | .1 | .1 | 88.3 |
|  | 04-JAN-2014 | 26 | .1 | .1 | 88.3 |
|  | 05-JAN-2014 | 27 | .1 | .1 | 88.4 |
|  | 06-JAN-2014 | 16 | .1 | .1 | 88.5 |
|  | 07-JAN-2014 | 21 | .1 | .1 | 88.6 |
|  | 08-JAN-2014 | 22 | .1 | .1 | 88.6 |
|  | 09-JAN-2014 | 25 | .1 | .1 | 88.7 |
|  | 10-JAN-2014 | 37 | .1 | .1 | 88.8 |
|  | 11-JAN-2014 | 9 | .0 | .0 | 88.9 |
|  | 12-JAN-2014 | 21 | .1 | .1 | 88.9 |
|  | 13-JAN-2014 | 17 | .1 | .1 | 89.0 |
|  | 14-JAN-2014 | 18 | .1 | .1 | 89.0 |
|  | 15-JAN-2014 | 34 | .1 | .1 | 89.2 |
|  | 16-JAN-2014 | 13 | .0 | .0 | 89.2 |
|  | 17-JAN-2014 | 20 | .1 | .1 | 89.3 |
|  | 18-JAN-2014 | 13 | .0 | .0 | 89.3 |
|  | 19-JAN-2014 | 10 | .0 | .0 | 89.3 |
|  | 20-JAN-2014 | 42 | .1 | .1 | 89.5 |
|  | 21-JAN-2014 | 13 | .0 | .0 | 89.5 |
|  | 22-JAN-2014 | 7 | .0 | .0 | 89.5 |
|  | 23-JAN-2014 | 13 | .0 | .0 | 89.6 |
|  | 24-JAN-2014 | 7 | .0 | .0 | 89.6 |
|  | 25-JAN-2014 | 24 | .1 | .1 | 89.7 |
|  | 26-JAN-2014 | 12 | .0 | .0 | 89.7 |
|  | 27-JAN-2014 | 8 | .0 | .0 | 89.8 |
|  | 28-JAN-2014 | 21 | .1 | .1 | 89.8 |
|  | 29-JAN-2014 | 12 | .0 | .0 | 89.9 |
|  | 30-JAN-2014 | 13 | .0 | .0 | 89.9 |
|  | 31-JAN-2014 | 7 | .0 | .0 | 89.9 |
|  | 01-FEB-2014 | 30 | .1 | .1 | 90.0 |
|  | 02-FEB-2014 | 26 | .1 | .1 | 90.1 |
|  | 03-FEB-2014 | 18 | .1 | .1 | 90.2 |
|  | 04-FEB-2014 | 16 | .1 | .1 | 90.2 |
|  | 05-FEB-2014 | 30 | .1 | .1 | 90.3 |
|  | 06-FEB-2014 | 12 | .0 | .0 | 90.4 |
|  | 07-FEB-2014 | 20 | .1 | .1 | 90.4 |
|  | 08-FEB-2014 | 17 | .1 | .1 | 90.5 |
|  | 09-FEB-2014 | 10 | .0 | .0 | 90.5 |
|  | 10-FEB-2014 | 37 | .1 | .1 | 90.6 |
|  | 11-FEB-2014 | 10 | .0 | .0 | 90.7 |
|  | 12-FEB-2014 | 20 | .1 | .1 | 90.7 |
|  | 13-FEB-2014 | 16 | .1 | .1 | 90.8 |
|  | 14-FEB-2014 | 20 | .1 | .1 | 90.8 |
|  | 15-FEB-2014 | 17 | .1 | .1 | 90.9 |
|  | 16-FEB-2014 | 9 | .0 | .0 | 90.9 |
|  | 17-FEB-2014 | 12 | .0 | .0 | 91.0 |
|  | 18-FEB-2014 | 15 | .0 | .0 | 91.0 |
|  | 19-FEB-2014 | 13 | .0 | .0 | 91.1 |
|  | 20-FEB-2014 | 19 | .1 | .1 | 91.1 |
|  | 21-FEB-2014 | 7 | .0 | .0 | 91.1 |
|  | 22-FEB-2014 | 11 | .0 | .0 | 91.2 |
|  | 23-FEB-2014 | 8 | .0 | .0 | 91.2 |
|  | 24-FEB-2014 | 17 | .1 | .1 | 91.3 |
|  | 25-FEB-2014 | 20 | .1 | .1 | 91.3 |
|  | 26-FEB-2014 | 15 | .0 | .0 | 91.4 |
|  | 27-FEB-2014 | 7 | .0 | .0 | 91.4 |
|  | 28-FEB-2014 | 19 | .1 | .1 | 91.5 |
|  | 01-MAR-2014 | 25 | .1 | .1 | 91.5 |
|  | 02-MAR-2014 | 16 | .1 | .1 | 91.6 |
|  | 03-MAR-2014 | 15 | .0 | .0 | 91.7 |
|  | 04-MAR-2014 | 16 | .1 | .1 | 91.7 |
|  | 05-MAR-2014 | 21 | .1 | .1 | 91.8 |
|  | 06-MAR-2014 | 10 | .0 | .0 | 91.8 |
|  | 07-MAR-2014 | 13 | .0 | .0 | 91.8 |
|  | 08-MAR-2014 | 14 | .0 | .0 | 91.9 |
|  | 09-MAR-2014 | 12 | .0 | .0 | 91.9 |
|  | 10-MAR-2014 | 34 | .1 | .1 | 92.0 |
|  | 11-MAR-2014 | 7 | .0 | .0 | 92.1 |
|  | 12-MAR-2014 | 22 | .1 | .1 | 92.1 |
|  | 13-MAR-2014 | 12 | .0 | .0 | 92.2 |
|  | 14-MAR-2014 | 11 | .0 | .0 | 92.2 |
|  | 15-MAR-2014 | 29 | .1 | .1 | 92.3 |
|  | 16-MAR-2014 | 10 | .0 | .0 | 92.3 |
|  | 17-MAR-2014 | 16 | .1 | .1 | 92.4 |
|  | 18-MAR-2014 | 16 | .1 | .1 | 92.4 |
|  | 19-MAR-2014 | 6 | .0 | .0 | 92.5 |
|  | 20-MAR-2014 | 27 | .1 | .1 | 92.6 |
|  | 21-MAR-2014 | 6 | .0 | .0 | 92.6 |
|  | 22-MAR-2014 | 12 | .0 | .0 | 92.6 |
|  | 23-MAR-2014 | 10 | .0 | .0 | 92.6 |
|  | 24-MAR-2014 | 13 | .0 | .0 | 92.7 |
|  | 25-MAR-2014 | 29 | .1 | .1 | 92.8 |
|  | 26-MAR-2014 | 16 | .1 | .1 | 92.8 |
|  | 27-MAR-2014 | 12 | .0 | .0 | 92.9 |
|  | 28-MAR-2014 | 10 | .0 | .0 | 92.9 |
|  | 29-MAR-2014 | 11 | .0 | .0 | 92.9 |
|  | 30-MAR-2014 | 9 | .0 | .0 | 93.0 |
|  | 31-MAR-2014 | 8 | .0 | .0 | 93.0 |
|  | 01-APR-2014 | 22 | .1 | .1 | 93.1 |
|  | 02-APR-2014 | 15 | .0 | .0 | 93.1 |
|  | 03-APR-2014 | 14 | .0 | .0 | 93.2 |
|  | 04-APR-2014 | 9 | .0 | .0 | 93.2 |
|  | 05-APR-2014 | 24 | .1 | .1 | 93.3 |
|  | 06-APR-2014 | 6 | .0 | .0 | 93.3 |
|  | 07-APR-2014 | 9 | .0 | .0 | 93.3 |
|  | 08-APR-2014 | 14 | .0 | .0 | 93.4 |
|  | 09-APR-2014 | 11 | .0 | .0 | 93.4 |
|  | 10-APR-2014 | 29 | .1 | .1 | 93.5 |
|  | 11-APR-2014 | 7 | .0 | .0 | 93.5 |
|  | 12-APR-2014 | 6 | .0 | .0 | 93.5 |
|  | 13-APR-2014 | 12 | .0 | .0 | 93.6 |
|  | 14-APR-2014 | 17 | .1 | .1 | 93.6 |
|  | 15-APR-2014 | 9 | .0 | .0 | 93.7 |
|  | 16-APR-2014 | 17 | .1 | .1 | 93.7 |
|  | 17-APR-2014 | 11 | .0 | .0 | 93.8 |
|  | 18-APR-2014 | 11 | .0 | .0 | 93.8 |
|  | 19-APR-2014 | 14 | .0 | .0 | 93.8 |
|  | 20-APR-2014 | 12 | .0 | .0 | 93.9 |
|  | 21-APR-2014 | 8 | .0 | .0 | 93.9 |
|  | 22-APR-2014 | 10 | .0 | .0 | 93.9 |
|  | 23-APR-2014 | 9 | .0 | .0 | 94.0 |
|  | 24-APR-2014 | 9 | .0 | .0 | 94.0 |
|  | 25-APR-2014 | 12 | .0 | .0 | 94.0 |
|  | 26-APR-2014 | 11 | .0 | .0 | 94.1 |
|  | 27-APR-2014 | 13 | .0 | .0 | 94.1 |
|  | 28-APR-2014 | 17 | .1 | .1 | 94.2 |
|  | 29-APR-2014 | 9 | .0 | .0 | 94.2 |
|  | 30-APR-2014 | 9 | .0 | .0 | 94.2 |
|  | 01-MAY-2014 | 19 | .1 | .1 | 94.3 |
|  | 02-MAY-2014 | 14 | .0 | .0 | 94.3 |
|  | 03-MAY-2014 | 5 | .0 | .0 | 94.4 |
|  | 04-MAY-2014 | 6 | .0 | .0 | 94.4 |
|  | 05-MAY-2014 | 18 | .1 | .1 | 94.4 |
|  | 06-MAY-2014 | 7 | .0 | .0 | 94.5 |
|  | 07-MAY-2014 | 12 | .0 | .0 | 94.5 |
|  | 08-MAY-2014 | 13 | .0 | .0 | 94.5 |
|  | 09-MAY-2014 | 13 | .0 | .0 | 94.6 |
|  | 10-MAY-2014 | 17 | .1 | .1 | 94.6 |
|  | 11-MAY-2014 | 14 | .0 | .0 | 94.7 |
|  | 12-MAY-2014 | 5 | .0 | .0 | 94.7 |
|  | 13-MAY-2014 | 16 | .1 | .1 | 94.8 |
|  | 14-MAY-2014 | 6 | .0 | .0 | 94.8 |
|  | 15-MAY-2014 | 8 | .0 | .0 | 94.8 |
|  | 16-MAY-2014 | 12 | .0 | .0 | 94.8 |
|  | 17-MAY-2014 | 10 | .0 | .0 | 94.9 |
|  | 18-MAY-2014 | 15 | .0 | .0 | 94.9 |
|  | 19-MAY-2014 | 12 | .0 | .0 | 95.0 |
|  | 20-MAY-2014 | 22 | .1 | .1 | 95.0 |
|  | 21-MAY-2014 | 9 | .0 | .0 | 95.1 |
|  | 22-MAY-2014 | 10 | .0 | .0 | 95.1 |
|  | 23-MAY-2014 | 8 | .0 | .0 | 95.1 |
|  | 24-MAY-2014 | 5 | .0 | .0 | 95.1 |
|  | 25-MAY-2014 | 13 | .0 | .0 | 95.2 |
|  | 26-MAY-2014 | 10 | .0 | .0 | 95.2 |
|  | 27-MAY-2014 | 9 | .0 | .0 | 95.2 |
|  | 28-MAY-2014 | 6 | .0 | .0 | 95.3 |
|  | 29-MAY-2014 | 7 | .0 | .0 | 95.3 |
|  | 30-MAY-2014 | 5 | .0 | .0 | 95.3 |
|  | 31-MAY-2014 | 4 | .0 | .0 | 95.3 |
|  | 01-JUN-2014 | 14 | .0 | .0 | 95.4 |
|  | 02-JUN-2014 | 8 | .0 | .0 | 95.4 |
|  | 03-JUN-2014 | 12 | .0 | .0 | 95.4 |
|  | 04-JUN-2014 | 6 | .0 | .0 | 95.4 |
|  | 05-JUN-2014 | 13 | .0 | .0 | 95.5 |
|  | 06-JUN-2014 | 4 | .0 | .0 | 95.5 |
|  | 07-JUN-2014 | 9 | .0 | .0 | 95.5 |
|  | 08-JUN-2014 | 12 | .0 | .0 | 95.6 |
|  | 09-JUN-2014 | 13 | .0 | .0 | 95.6 |
|  | 10-JUN-2014 | 13 | .0 | .0 | 95.7 |
|  | 11-JUN-2014 | 8 | .0 | .0 | 95.7 |
|  | 12-JUN-2014 | 10 | .0 | .0 | 95.7 |
|  | 13-JUN-2014 | 5 | .0 | .0 | 95.7 |
|  | 14-JUN-2014 | 13 | .0 | .0 | 95.8 |
|  | 15-JUN-2014 | 21 | .1 | .1 | 95.8 |
|  | 16-JUN-2014 | 10 | .0 | .0 | 95.9 |
|  | 17-JUN-2014 | 10 | .0 | .0 | 95.9 |
|  | 18-JUN-2014 | 15 | .0 | .0 | 96.0 |
|  | 19-JUN-2014 | 9 | .0 | .0 | 96.0 |
|  | 20-JUN-2014 | 12 | .0 | .0 | 96.0 |
|  | 21-JUN-2014 | 5 | .0 | .0 | 96.0 |
|  | 22-JUN-2014 | 7 | .0 | .0 | 96.1 |
|  | 23-JUN-2014 | 10 | .0 | .0 | 96.1 |
|  | 24-JUN-2014 | 8 | .0 | .0 | 96.1 |
|  | 25-JUN-2014 | 9 | .0 | .0 | 96.1 |
|  | 26-JUN-2014 | 8 | .0 | .0 | 96.2 |
|  | 27-JUN-2014 | 11 | .0 | .0 | 96.2 |
|  | 28-JUN-2014 | 9 | .0 | .0 | 96.2 |
|  | 29-JUN-2014 | 10 | .0 | .0 | 96.3 |
|  | 30-JUN-2014 | 9 | .0 | .0 | 96.3 |
|  | 01-JUL-2014 | 18 | .1 | .1 | 96.4 |
|  | 02-JUL-2014 | 11 | .0 | .0 | 96.4 |
|  | 03-JUL-2014 | 9 | .0 | .0 | 96.4 |
|  | 04-JUL-2014 | 8 | .0 | .0 | 96.5 |
|  | 05-JUL-2014 | 13 | .0 | .0 | 96.5 |
|  | 06-JUL-2014 | 8 | .0 | .0 | 96.5 |
|  | 07-JUL-2014 | 9 | .0 | .0 | 96.6 |
|  | 08-JUL-2014 | 12 | .0 | .0 | 96.6 |
|  | 09-JUL-2014 | 8 | .0 | .0 | 96.6 |
|  | 10-JUL-2014 | 25 | .1 | .1 | 96.7 |
|  | 11-JUL-2014 | 10 | .0 | .0 | 96.7 |
|  | 12-JUL-2014 | 12 | .0 | .0 | 96.8 |
|  | 13-JUL-2014 | 7 | .0 | .0 | 96.8 |
|  | 14-JUL-2014 | 9 | .0 | .0 | 96.8 |
|  | 15-JUL-2014 | 19 | .1 | .1 | 96.9 |
|  | 16-JUL-2014 | 5 | .0 | .0 | 96.9 |
|  | 17-JUL-2014 | 6 | .0 | .0 | 96.9 |
|  | 18-JUL-2014 | 8 | .0 | .0 | 96.9 |
|  | 19-JUL-2014 | 7 | .0 | .0 | 97.0 |
|  | 20-JUL-2014 | 17 | .1 | .1 | 97.0 |
|  | 21-JUL-2014 | 10 | .0 | .0 | 97.1 |
|  | 22-JUL-2014 | 8 | .0 | .0 | 97.1 |
|  | 23-JUL-2014 | 11 | .0 | .0 | 97.1 |
|  | 24-JUL-2014 | 5 | .0 | .0 | 97.1 |
|  | 25-JUL-2014 | 14 | .0 | .0 | 97.2 |
|  | 26-JUL-2014 | 10 | .0 | .0 | 97.2 |
|  | 27-JUL-2014 | 9 | .0 | .0 | 97.2 |
|  | 28-JUL-2014 | 12 | .0 | .0 | 97.3 |
|  | 29-JUL-2014 | 13 | .0 | .0 | 97.3 |
|  | 30-JUL-2014 | 7 | .0 | .0 | 97.3 |
|  | 31-JUL-2014 | 3 | .0 | .0 | 97.4 |
|  | 01-AUG-2014 | 14 | .0 | .0 | 97.4 |
|  | 02-AUG-2014 | 13 | .0 | .0 | 97.4 |
|  | 03-AUG-2014 | 12 | .0 | .0 | 97.5 |
|  | 04-AUG-2014 | 8 | .0 | .0 | 97.5 |
|  | 05-AUG-2014 | 8 | .0 | .0 | 97.5 |
|  | 06-AUG-2014 | 5 | .0 | .0 | 97.6 |
|  | 07-AUG-2014 | 5 | .0 | .0 | 97.6 |
|  | 08-AUG-2014 | 10 | .0 | .0 | 97.6 |
|  | 09-AUG-2014 | 12 | .0 | .0 | 97.6 |
|  | 10-AUG-2014 | 16 | .1 | .1 | 97.7 |
|  | 11-AUG-2014 | 11 | .0 | .0 | 97.7 |
|  | 12-AUG-2014 | 8 | .0 | .0 | 97.8 |
|  | 13-AUG-2014 | 3 | .0 | .0 | 97.8 |
|  | 14-AUG-2014 | 10 | .0 | .0 | 97.8 |
|  | 15-AUG-2014 | 16 | .1 | .1 | 97.9 |
|  | 16-AUG-2014 | 3 | .0 | .0 | 97.9 |
|  | 17-AUG-2014 | 4 | .0 | .0 | 97.9 |
|  | 18-AUG-2014 | 9 | .0 | .0 | 97.9 |
|  | 19-AUG-2014 | 6 | .0 | .0 | 97.9 |
|  | 20-AUG-2014 | 12 | .0 | .0 | 98.0 |
|  | 21-AUG-2014 | 3 | .0 | .0 | 98.0 |
|  | 22-AUG-2014 | 18 | .1 | .1 | 98.0 |
|  | 23-AUG-2014 | 7 | .0 | .0 | 98.1 |
|  | 24-AUG-2014 | 7 | .0 | .0 | 98.1 |
|  | 25-AUG-2014 | 11 | .0 | .0 | 98.1 |
|  | 26-AUG-2014 | 6 | .0 | .0 | 98.1 |
|  | 27-AUG-2014 | 4 | .0 | .0 | 98.1 |
|  | 28-AUG-2014 | 11 | .0 | .0 | 98.2 |
|  | 29-AUG-2014 | 5 | .0 | .0 | 98.2 |
|  | 30-AUG-2014 | 6 | .0 | .0 | 98.2 |
|  | 31-AUG-2014 | 3 | .0 | .0 | 98.2 |
|  | 01-SEP-2014 | 13 | .0 | .0 | 98.3 |
|  | 02-SEP-2014 | 2 | .0 | .0 | 98.3 |
|  | 03-SEP-2014 | 7 | .0 | .0 | 98.3 |
|  | 04-SEP-2014 | 3 | .0 | .0 | 98.3 |
|  | 05-SEP-2014 | 6 | .0 | .0 | 98.3 |
|  | 06-SEP-2014 | 7 | .0 | .0 | 98.4 |
|  | 07-SEP-2014 | 6 | .0 | .0 | 98.4 |
|  | 08-SEP-2014 | 4 | .0 | .0 | 98.4 |
|  | 09-SEP-2014 | 6 | .0 | .0 | 98.4 |
|  | 10-SEP-2014 | 12 | .0 | .0 | 98.4 |
|  | 11-SEP-2014 | 3 | .0 | .0 | 98.5 |
|  | 12-SEP-2014 | 10 | .0 | .0 | 98.5 |
|  | 13-SEP-2014 | 2 | .0 | .0 | 98.5 |
|  | 14-SEP-2014 | 1 | .0 | .0 | 98.5 |
|  | 15-SEP-2014 | 9 | .0 | .0 | 98.5 |
|  | 16-SEP-2014 | 4 | .0 | .0 | 98.5 |
|  | 17-SEP-2014 | 7 | .0 | .0 | 98.6 |
|  | 18-SEP-2014 | 4 | .0 | .0 | 98.6 |
|  | 19-SEP-2014 | 5 | .0 | .0 | 98.6 |
|  | 20-SEP-2014 | 7 | .0 | .0 | 98.6 |
|  | 21-SEP-2014 | 1 | .0 | .0 | 98.6 |
|  | 22-SEP-2014 | 5 | .0 | .0 | 98.6 |
|  | 23-SEP-2014 | 2 | .0 | .0 | 98.6 |
|  | 24-SEP-2014 | 4 | .0 | .0 | 98.7 |
|  | 25-SEP-2014 | 8 | .0 | .0 | 98.7 |
|  | 26-SEP-2014 | 4 | .0 | .0 | 98.7 |
|  | 27-SEP-2014 | 4 | .0 | .0 | 98.7 |
|  | 28-SEP-2014 | 4 | .0 | .0 | 98.7 |
|  | 29-SEP-2014 | 3 | .0 | .0 | 98.7 |
|  | 30-SEP-2014 | 3 | .0 | .0 | 98.7 |
|  | 01-OCT-2014 | 9 | .0 | .0 | 98.8 |
|  | 02-OCT-2014 | 4 | .0 | .0 | 98.8 |
|  | 03-OCT-2014 | 5 | .0 | .0 | 98.8 |
|  | 04-OCT-2014 | 3 | .0 | .0 | 98.8 |
|  | 05-OCT-2014 | 4 | .0 | .0 | 98.8 |
|  | 06-OCT-2014 | 4 | .0 | .0 | 98.8 |
|  | 07-OCT-2014 | 2 | .0 | .0 | 98.8 |
|  | 08-OCT-2014 | 5 | .0 | .0 | 98.9 |
|  | 09-OCT-2014 | 6 | .0 | .0 | 98.9 |
|  | 10-OCT-2014 | 9 | .0 | .0 | 98.9 |
|  | 11-OCT-2014 | 4 | .0 | .0 | 98.9 |
|  | 12-OCT-2014 | 2 | .0 | .0 | 98.9 |
|  | 13-OCT-2014 | 6 | .0 | .0 | 98.9 |
|  | 14-OCT-2014 | 3 | .0 | .0 | 99.0 |
|  | 15-OCT-2014 | 1 | .0 | .0 | 99.0 |
|  | 16-OCT-2014 | 4 | .0 | .0 | 99.0 |
|  | 17-OCT-2014 | 5 | .0 | .0 | 99.0 |
|  | 18-OCT-2014 | 1 | .0 | .0 | 99.0 |
|  | 19-OCT-2014 | 3 | .0 | .0 | 99.0 |
|  | 20-OCT-2014 | 9 | .0 | .0 | 99.0 |
|  | 21-OCT-2014 | 3 | .0 | .0 | 99.0 |
|  | 22-OCT-2014 | 3 | .0 | .0 | 99.1 |
|  | 23-OCT-2014 | 6 | .0 | .0 | 99.1 |
|  | 24-OCT-2014 | 4 | .0 | .0 | 99.1 |
|  | 25-OCT-2014 | 5 | .0 | .0 | 99.1 |
|  | 26-OCT-2014 | 2 | .0 | .0 | 99.1 |
|  | 27-OCT-2014 | 2 | .0 | .0 | 99.1 |
|  | 28-OCT-2014 | 3 | .0 | .0 | 99.1 |
|  | 29-OCT-2014 | 3 | .0 | .0 | 99.1 |
|  | 30-OCT-2014 | 3 | .0 | .0 | 99.1 |
|  | 01-NOV-2014 | 3 | .0 | .0 | 99.2 |
|  | 02-NOV-2014 | 1 | .0 | .0 | 99.2 |
|  | 03-NOV-2014 | 4 | .0 | .0 | 99.2 |
|  | 04-NOV-2014 | 2 | .0 | .0 | 99.2 |
|  | 05-NOV-2014 | 6 | .0 | .0 | 99.2 |
|  | 06-NOV-2014 | 4 | .0 | .0 | 99.2 |
|  | 08-NOV-2014 | 3 | .0 | .0 | 99.2 |
|  | 09-NOV-2014 | 5 | .0 | .0 | 99.2 |
|  | 10-NOV-2014 | 7 | .0 | .0 | 99.3 |
|  | 11-NOV-2014 | 3 | .0 | .0 | 99.3 |
|  | 12-NOV-2014 | 2 | .0 | .0 | 99.3 |
|  | 13-NOV-2014 | 6 | .0 | .0 | 99.3 |
|  | 14-NOV-2014 | 3 | .0 | .0 | 99.3 |
|  | 15-NOV-2014 | 8 | .0 | .0 | 99.3 |
|  | 16-NOV-2014 | 3 | .0 | .0 | 99.3 |
|  | 17-NOV-2014 | 2 | .0 | .0 | 99.3 |
|  | 18-NOV-2014 | 5 | .0 | .0 | 99.4 |
|  | 19-NOV-2014 | 2 | .0 | .0 | 99.4 |
|  | 20-NOV-2014 | 10 | .0 | .0 | 99.4 |
|  | 21-NOV-2014 | 2 | .0 | .0 | 99.4 |
|  | 22-NOV-2014 | 5 | .0 | .0 | 99.4 |
|  | 23-NOV-2014 | 2 | .0 | .0 | 99.4 |
|  | 24-NOV-2014 | 3 | .0 | .0 | 99.4 |
|  | 25-NOV-2014 | 3 | .0 | .0 | 99.5 |
|  | 26-NOV-2014 | 3 | .0 | .0 | 99.5 |
|  | 27-NOV-2014 | 3 | .0 | .0 | 99.5 |
|  | 28-NOV-2014 | 2 | .0 | .0 | 99.5 |
|  | 29-NOV-2014 | 1 | .0 | .0 | 99.5 |
|  | 01-DEC-2014 | 3 | .0 | .0 | 99.5 |
|  | 02-DEC-2014 | 1 | .0 | .0 | 99.5 |
|  | 04-DEC-2014 | 1 | .0 | .0 | 99.5 |
|  | 05-DEC-2014 | 2 | .0 | .0 | 99.5 |
|  | 06-DEC-2014 | 5 | .0 | .0 | 99.5 |
|  | 07-DEC-2014 | 7 | .0 | .0 | 99.5 |
|  | 08-DEC-2014 | 3 | .0 | .0 | 99.6 |
|  | 09-DEC-2014 | 2 | .0 | .0 | 99.6 |
|  | 10-DEC-2014 | 3 | .0 | .0 | 99.6 |
|  | 11-DEC-2014 | 2 | .0 | .0 | 99.6 |
|  | 12-DEC-2014 | 4 | .0 | .0 | 99.6 |
|  | 13-DEC-2014 | 2 | .0 | .0 | 99.6 |
|  | 14-DEC-2014 | 3 | .0 | .0 | 99.6 |
|  | 15-DEC-2014 | 5 | .0 | .0 | 99.6 |
|  | 16-DEC-2014 | 2 | .0 | .0 | 99.6 |
|  | 17-DEC-2014 | 4 | .0 | .0 | 99.6 |
|  | 18-DEC-2014 | 2 | .0 | .0 | 99.6 |
|  | 19-DEC-2014 | 1 | .0 | .0 | 99.6 |
|  | 20-DEC-2014 | 6 | .0 | .0 | 99.7 |
|  | 21-DEC-2014 | 3 | .0 | .0 | 99.7 |
|  | 22-DEC-2014 | 1 | .0 | .0 | 99.7 |
|  | 24-DEC-2014 | 3 | .0 | .0 | 99.7 |
|  | 25-DEC-2014 | 2 | .0 | .0 | 99.7 |
|  | 26-DEC-2014 | 2 | .0 | .0 | 99.7 |
|  | 27-DEC-2014 | 1 | .0 | .0 | 99.7 |
|  | 30-DEC-2014 | 2 | .0 | .0 | 99.7 |
|  | 31-DEC-2014 | 1 | .0 | .0 | 99.7 |
|  | 01-JAN-2015 | 6 | .0 | .0 | 99.7 |
|  | 02-JAN-2015 | 5 | .0 | .0 | 99.8 |
|  | 04-JAN-2015 | 1 | .0 | .0 | 99.8 |
|  | 05-JAN-2015 | 1 | .0 | .0 | 99.8 |
|  | 07-JAN-2015 | 3 | .0 | .0 | 99.8 |
|  | 08-JAN-2015 | 1 | .0 | .0 | 99.8 |
|  | 09-JAN-2015 | 4 | .0 | .0 | 99.8 |
|  | 10-JAN-2015 | 1 | .0 | .0 | 99.8 |
|  | 11-JAN-2015 | 3 | .0 | .0 | 99.8 |
|  | 12-JAN-2015 | 2 | .0 | .0 | 99.8 |
|  | 14-JAN-2015 | 1 | .0 | .0 | 99.8 |
|  | 15-JAN-2015 | 3 | .0 | .0 | 99.8 |
|  | 16-JAN-2015 | 3 | .0 | .0 | 99.8 |
|  | 17-JAN-2015 | 2 | .0 | .0 | 99.8 |
|  | 18-JAN-2015 | 1 | .0 | .0 | 99.8 |
|  | 20-JAN-2015 | 1 | .0 | .0 | 99.8 |
|  | 21-JAN-2015 | 1 | .0 | .0 | 99.8 |
|  | 22-JAN-2015 | 1 | .0 | .0 | 99.8 |
|  | 23-JAN-2015 | 1 | .0 | .0 | 99.9 |
|  | 25-JAN-2015 | 1 | .0 | .0 | 99.9 |
|  | 28-JAN-2015 | 2 | .0 | .0 | 99.9 |
|  | 30-JAN-2015 | 1 | .0 | .0 | 99.9 |
|  | 31-JAN-2015 | 1 | .0 | .0 | 99.9 |
|  | 01-FEB-2015 | 2 | .0 | .0 | 99.9 |
|  | 02-FEB-2015 | 3 | .0 | .0 | 99.9 |
|  | 04-FEB-2015 | 1 | .0 | .0 | 99.9 |
|  | 05-FEB-2015 | 2 | .0 | .0 | 99.9 |
|  | 06-FEB-2015 | 1 | .0 | .0 | 99.9 |
|  | 07-FEB-2015 | 1 | .0 | .0 | 99.9 |
|  | 08-FEB-2015 | 1 | .0 | .0 | 99.9 |
|  | 09-FEB-2015 | 1 | .0 | .0 | 99.9 |
|  | 10-FEB-2015 | 1 | .0 | .0 | 99.9 |
|  | 11-FEB-2015 | 1 | .0 | .0 | 99.9 |
|  | 13-FEB-2015 | 1 | .0 | .0 | 99.9 |
|  | 14-FEB-2015 | 2 | .0 | .0 | 99.9 |
|  | 16-FEB-2015 | 1 | .0 | .0 | 99.9 |
|  | 17-FEB-2015 | 1 | .0 | .0 | 99.9 |
|  | 18-FEB-2015 | 3 | .0 | .0 | 99.9 |
|  | 19-FEB-2015 | 2 | .0 | .0 | 99.9 |
|  | 20-FEB-2015 | 3 | .0 | .0 | 100.0 |
|  | 22-FEB-2015 | 1 | .0 | .0 | 100.0 |
|  | 24-FEB-2015 | 1 | .0 | .0 | 100.0 |
|  | 25-FEB-2015 | 1 | .0 | .0 | 100.0 |
|  | 26-FEB-2015 | 2 | .0 | .0 | 100.0 |
|  | 27-FEB-2015 | 1 | .0 | .0 | 100.0 |
|  | 28-FEB-2015 | 1 | .0 | .0 | 100.0 |
|  | 01-MAR-2015 | 3 | .0 | .0 | 100.0 |
|  | 02-MAR-2015 | 1 | .0 | .0 | 100.0 |
|  | 04-MAR-2015 | 1 | .0 | .0 | 100.0 |
|  | 10-MAR-2015 | 1 | .0 | .0 | 100.0 |
|  | Total | 30553 | 100.0 | 100.0 |  |
|  |  |  |  |  |  |

Child ageChild age, table, 1 levels of column headers and 2 levels of row headers, table with 6 columns and 56 rows

|  |  |  |  |  |  |
| --- | --- | --- | --- | --- | --- |
|  | | Frequency | Percent | Valid Percent | Cumulative Percent |
| Valid | 8 | 264 | .9 | .9 | .9 |
|  | 9 | 528 | 1.7 | 1.7 | 2.6 |
|  | 10 | 848 | 2.8 | 2.8 | 5.4 |
|  | 11 | 1177 | 3.9 | 3.9 | 9.2 |
|  | 12 | 1036 | 3.4 | 3.4 | 12.6 |
|  | 13 | 906 | 3.0 | 3.0 | 15.6 |
|  | 14 | 839 | 2.7 | 2.7 | 18.3 |
|  | 15 | 769 | 2.5 | 2.5 | 20.8 |
|  | 16 | 830 | 2.7 | 2.7 | 23.6 |
|  | 17 | 811 | 2.7 | 2.7 | 26.2 |
|  | 18 | 838 | 2.7 | 2.7 | 29.0 |
|  | 19 | 758 | 2.5 | 2.5 | 31.4 |
|  | 20 | 827 | 2.7 | 2.7 | 34.1 |
|  | 21 | 820 | 2.7 | 2.7 | 36.8 |
|  | 22 | 842 | 2.8 | 2.8 | 39.6 |
|  | 23 | 830 | 2.7 | 2.7 | 42.3 |
|  | 24 | 840 | 2.7 | 2.7 | 45.0 |
|  | 25 | 747 | 2.4 | 2.4 | 47.5 |
|  | 26 | 754 | 2.5 | 2.5 | 50.0 |
|  | 27 | 756 | 2.5 | 2.5 | 52.4 |
|  | 28 | 789 | 2.6 | 2.6 | 55.0 |
|  | 29 | 760 | 2.5 | 2.5 | 57.5 |
|  | 30 | 734 | 2.4 | 2.4 | 59.9 |
|  | 31 | 718 | 2.4 | 2.4 | 62.3 |
|  | 32 | 717 | 2.3 | 2.3 | 64.6 |
|  | 33 | 777 | 2.5 | 2.5 | 67.1 |
|  | 34 | 852 | 2.8 | 2.8 | 69.9 |
|  | 35 | 805 | 2.6 | 2.6 | 72.6 |
|  | 36 | 784 | 2.6 | 2.6 | 75.1 |
|  | 37 | 705 | 2.3 | 2.3 | 77.4 |
|  | 38 | 608 | 2.0 | 2.0 | 79.4 |
|  | 39 | 550 | 1.8 | 1.8 | 81.2 |
|  | 40 | 487 | 1.6 | 1.6 | 82.8 |
|  | 41 | 483 | 1.6 | 1.6 | 84.4 |
|  | 42 | 391 | 1.3 | 1.3 | 85.7 |
|  | 43 | 348 | 1.1 | 1.1 | 86.8 |
|  | 44 | 308 | 1.0 | 1.0 | 87.8 |
|  | 45 | 322 | 1.1 | 1.1 | 88.9 |
|  | 46 | 361 | 1.2 | 1.2 | 90.1 |
|  | 47 | 346 | 1.1 | 1.1 | 91.2 |
|  | 48 | 317 | 1.0 | 1.0 | 92.2 |
|  | 49 | 265 | .9 | .9 | 93.1 |
|  | 50 | 258 | .8 | .8 | 94.0 |
|  | 51 | 236 | .8 | .8 | 94.7 |
|  | 52 | 257 | .8 | .8 | 95.6 |
|  | 53 | 244 | .8 | .8 | 96.4 |
|  | 54 | 199 | .7 | .7 | 97.0 |
|  | 55 | 208 | .7 | .7 | 97.7 |
|  | 56 | 165 | .5 | .5 | 98.2 |
|  | 57 | 160 | .5 | .5 | 98.8 |
|  | 58 | 174 | .6 | .6 | 99.3 |
|  | 59 | 137 | .4 | .4 | 99.8 |
|  | 60 | 68 | .2 | .2 | 100.0 |
|  | Total | 30553 | 100.0 | 100.0 |  |
|  |  |  |  |  |  |

Interview start timeInterview start time, table, 1 levels of column headers and 2 levels of row headers, table with 6 columns and 678 rows

|  |  |  |  |  |  |
| --- | --- | --- | --- | --- | --- |
|  | | Frequency | Percent | Valid Percent | Cumulative Percent |
| Valid | 13:00 | 423 | 1.4 | 1.4 | 1.4 |
|  | 13:02 | 3 | .0 | .0 | 1.4 |
|  | 13:03 | 8 | .0 | .0 | 1.4 |
|  | 13:04 | 7 | .0 | .0 | 1.4 |
|  | 13:05 | 190 | .6 | .6 | 2.1 |
|  | 13:06 | 9 | .0 | .0 | 2.1 |
|  | 13:07 | 8 | .0 | .0 | 2.1 |
|  | 13:08 | 8 | .0 | .0 | 2.1 |
|  | 13:09 | 12 | .0 | .0 | 2.2 |
|  | 13:10 | 1444 | 4.7 | 4.7 | 6.9 |
|  | 13:11 | 221 | .7 | .7 | 7.6 |
|  | 13:12 | 526 | 1.7 | 1.7 | 9.4 |
|  | 13:13 | 289 | .9 | .9 | 10.3 |
|  | 13:14 | 73 | .2 | .2 | 10.5 |
|  | 13:15 | 275 | .9 | .9 | 11.4 |
|  | 13:16 | 7 | .0 | .0 | 11.5 |
|  | 13:17 | 7 | .0 | .0 | 11.5 |
|  | 13:18 | 13 | .0 | .0 | 11.5 |
|  | 13:19 | 5 | .0 | .0 | 11.5 |
|  | 13:20 | 713 | 2.3 | 2.3 | 13.9 |
|  | 13:21 | 10 | .0 | .0 | 13.9 |
|  | 13:22 | 16 | .1 | .1 | 14.0 |
|  | 13:23 | 6 | .0 | .0 | 14.0 |
|  | 13:24 | 5 | .0 | .0 | 14.0 |
|  | 13:25 | 288 | .9 | .9 | 14.9 |
|  | 13:26 | 13 | .0 | .0 | 15.0 |
|  | 13:27 | 10 | .0 | .0 | 15.0 |
|  | 13:28 | 9 | .0 | .0 | 15.0 |
|  | 13:29 | 3 | .0 | .0 | 15.1 |
|  | 13:3 | 2 | .0 | .0 | 15.1 |
|  | 13:30 | 6251 | 20.5 | 20.5 | 35.5 |
|  | 13:31 | 5 | .0 | .0 | 35.5 |
|  | 13:32 | 14 | .0 | .0 | 35.6 |
|  | 13:33 | 15 | .0 | .0 | 35.6 |
|  | 13:34 | 10 | .0 | .0 | 35.7 |
|  | 13:35 | 184 | .6 | .6 | 36.3 |
|  | 13:36 | 17 | .1 | .1 | 36.3 |
|  | 13:37 | 5 | .0 | .0 | 36.3 |
|  | 13:38 | 111 | .4 | .4 | 36.7 |
|  | 13:39 | 9 | .0 | .0 | 36.7 |
|  | 13:40 | 573 | 1.9 | 1.9 | 38.6 |
|  | 13:41 | 5 | .0 | .0 | 38.6 |
|  | 13:42 | 11 | .0 | .0 | 38.7 |
|  | 13:43 | 7 | .0 | .0 | 38.7 |
|  | 13:44 | 6 | .0 | .0 | 38.7 |
|  | 13:45 | 214 | .7 | .7 | 39.4 |
|  | 13:46 | 8 | .0 | .0 | 39.4 |
|  | 13:47 | 5 | .0 | .0 | 39.4 |
|  | 13:48 | 5 | .0 | .0 | 39.5 |
|  | 13:49 | 4 | .0 | .0 | 39.5 |
|  | 13:50 | 509 | 1.7 | 1.7 | 41.1 |
|  | 13:51 | 6 | .0 | .0 | 41.2 |
|  | 13:52 | 6 | .0 | .0 | 41.2 |
|  | 13:53 | 3 | .0 | .0 | 41.2 |
|  | 13:54 | 7 | .0 | .0 | 41.2 |
|  | 13:55 | 138 | .5 | .5 | 41.7 |
|  | 13:56 | 7 | .0 | .0 | 41.7 |
|  | 13:57 | 5 | .0 | .0 | 41.7 |
|  | 13:58 | 16 | .1 | .1 | 41.8 |
|  | 13:59 | 20 | .1 | .1 | 41.8 |
|  | 13:60 | 54 | .2 | .2 | 42.0 |
|  | 13:61 | 1 | .0 | .0 | 42.0 |
|  | 13:63 | 1 | .0 | .0 | 42.0 |
|  | 13:64 | 1 | .0 | .0 | 42.0 |
|  | 13:65 | 1 | .0 | .0 | 42.0 |
|  | 13:68 | 1 | .0 | .0 | 42.0 |
|  | 13:70 | 3 | .0 | .0 | 42.0 |
|  | 13:80 | 2 | .0 | .0 | 42.0 |
|  | 13:95 | 1 | .0 | .0 | 42.0 |
|  | 13:L1 | 1 | .0 | .0 | 42.0 |
|  | 13.00 | 7 | .0 | .0 | 42.1 |
|  | 13.05 | 1 | .0 | .0 | 42.1 |
|  | 13.08 | 1 | .0 | .0 | 42.1 |
|  | 13.10 | 8 | .0 | .0 | 42.1 |
|  | 13.13 | 1 | .0 | .0 | 42.1 |
|  | 13.15 | 5 | .0 | .0 | 42.1 |
|  | 13.19 | 1 | .0 | .0 | 42.1 |
|  | 13.20 | 11 | .0 | .0 | 42.2 |
|  | 13.25 | 2 | .0 | .0 | 42.2 |
|  | 13.26 | 1 | .0 | .0 | 42.2 |
|  | 13.30 | 22 | .1 | .1 | 42.2 |
|  | 13.35 | 4 | .0 | .0 | 42.3 |
|  | 13.40 | 9 | .0 | .0 | 42.3 |
|  | 13.45 | 3 | .0 | .0 | 42.3 |
|  | 13.50 | 9 | .0 | .0 | 42.3 |
|  | 13.55 | 4 | .0 | .0 | 42.3 |
|  | 13':2 | 1 | .0 | .0 | 42.3 |
|  | 130 | 4 | .0 | .0 | 42.3 |
|  | 1300 | 52 | .2 | .2 | 42.5 |
|  | 1302 | 1 | .0 | .0 | 42.5 |
|  | 1303 | 1 | .0 | .0 | 42.5 |
|  | 1305 | 23 | .1 | .1 | 42.6 |
|  | 1306 | 2 | .0 | .0 | 42.6 |
|  | 1307 | 1 | .0 | .0 | 42.6 |
|  | 1309 | 1 | .0 | .0 | 42.6 |
|  | 1310 | 89 | .3 | .3 | 42.9 |
|  | 1311 | 2 | .0 | .0 | 42.9 |
|  | 1312 | 4 | .0 | .0 | 42.9 |
|  | 1313 | 4 | .0 | .0 | 42.9 |
|  | 1314 | 3 | .0 | .0 | 42.9 |
|  | 1315 | 27 | .1 | .1 | 43.0 |
|  | 1316 | 1 | .0 | .0 | 43.0 |
|  | 1317 | 1 | .0 | .0 | 43.0 |
|  | 1318 | 4 | .0 | .0 | 43.1 |
|  | 1320 | 101 | .3 | .3 | 43.4 |
|  | 1322 | 2 | .0 | .0 | 43.4 |
|  | 13220 | 1 | .0 | .0 | 43.4 |
|  | 1323 | 1 | .0 | .0 | 43.4 |
|  | 1325 | 30 | .1 | .1 | 43.5 |
|  | 1326 | 2 | .0 | .0 | 43.5 |
|  | 1327 | 2 | .0 | .0 | 43.5 |
|  | 1329 | 3 | .0 | .0 | 43.5 |
|  | 1330 | 118 | .4 | .4 | 43.9 |
|  | 1331 | 1 | .0 | .0 | 43.9 |
|  | 1332 | 5 | .0 | .0 | 43.9 |
|  | 1333 | 1 | .0 | .0 | 43.9 |
|  | 1334 | 2 | .0 | .0 | 43.9 |
|  | 1335 | 21 | .1 | .1 | 44.0 |
|  | 1336 | 3 | .0 | .0 | 44.0 |
|  | 1338 | 1 | .0 | .0 | 44.0 |
|  | 1339 | 2 | .0 | .0 | 44.0 |
|  | 1340 | 108 | .4 | .4 | 44.4 |
|  | 1342 | 5 | .0 | .0 | 44.4 |
|  | 1345 | 27 | .1 | .1 | 44.5 |
|  | 1346 | 2 | .0 | .0 | 44.5 |
|  | 1347 | 2 | .0 | .0 | 44.5 |
|  | 1348 | 1 | .0 | .0 | 44.5 |
|  | 135 | 1 | .0 | .0 | 44.5 |
|  | 1350 | 66 | .2 | .2 | 44.7 |
|  | 1352 | 2 | .0 | .0 | 44.7 |
|  | 1353 | 1 | .0 | .0 | 44.7 |
|  | 1355 | 18 | .1 | .1 | 44.8 |
|  | 1356 | 3 | .0 | .0 | 44.8 |
|  | 1357 | 4 | .0 | .0 | 44.8 |
|  | 1358 | 1 | .0 | .0 | 44.8 |
|  | 1360 | 8 | .0 | .0 | 44.8 |
|  | 1369 | 1 | .0 | .0 | 44.8 |
|  | 138 | 2 | .0 | .0 | 44.8 |
|  | 14 | 4 | .0 | .0 | 44.9 |
|  | 14;'1 | 1 | .0 | .0 | 44.9 |
|  | 14;00 | 6 | .0 | .0 | 44.9 |
|  | 14;03 | 1 | .0 | .0 | 44.9 |
|  | 14;05 | 3 | .0 | .0 | 44.9 |
|  | 14;10 | 6 | .0 | .0 | 44.9 |
|  | 14;15 | 4 | .0 | .0 | 44.9 |
|  | 14;20 | 7 | .0 | .0 | 45.0 |
|  | 14;25 | 2 | .0 | .0 | 45.0 |
|  | 14;30 | 7 | .0 | .0 | 45.0 |
|  | 14;35 | 2 | .0 | .0 | 45.0 |
|  | 14;40 | 5 | .0 | .0 | 45.0 |
|  | 14;45 | 3 | .0 | .0 | 45.0 |
|  | 14;50 | 2 | .0 | .0 | 45.0 |
|  | 14;70 | 1 | .0 | .0 | 45.0 |
|  | 14:.1 | 1 | .0 | .0 | 45.0 |
|  | 14:'0 | 1 | .0 | .0 | 45.0 |
|  | 14:'3 | 1 | .0 | .0 | 45.0 |
|  | 14:0 | 1 | .0 | .0 | 45.0 |
|  | 14:00 | 862 | 2.8 | 2.8 | 47.9 |
|  | 14:02 | 14 | .0 | .0 | 47.9 |
|  | 14:03 | 10 | .0 | .0 | 47.9 |
|  | 14:04 | 14 | .0 | .0 | 48.0 |
|  | 14:05 | 168 | .5 | .5 | 48.5 |
|  | 14:06 | 5 | .0 | .0 | 48.6 |
|  | 14:07 | 5 | .0 | .0 | 48.6 |
|  | 14:08 | 13 | .0 | .0 | 48.6 |
|  | 14:09 | 6 | .0 | .0 | 48.6 |
|  | 14:10 | 785 | 2.6 | 2.6 | 51.2 |
|  | 14:11 | 17 | .1 | .1 | 51.3 |
|  | 14:12 | 31 | .1 | .1 | 51.4 |
|  | 14:13 | 40 | .1 | .1 | 51.5 |
|  | 14:14 | 16 | .1 | .1 | 51.5 |
|  | 14:15 | 313 | 1.0 | 1.0 | 52.6 |
|  | 14:16 | 23 | .1 | .1 | 52.6 |
|  | 14:17 | 19 | .1 | .1 | 52.7 |
|  | 14:18 | 13 | .0 | .0 | 52.7 |
|  | 14:19 | 8 | .0 | .0 | 52.8 |
|  | 14:20 | 872 | 2.9 | 2.9 | 55.6 |
|  | 14:21 | 14 | .0 | .0 | 55.7 |
|  | 14:22 | 22 | .1 | .1 | 55.7 |
|  | 14:23 | 12 | .0 | .0 | 55.8 |
|  | 14:24 | 5 | .0 | .0 | 55.8 |
|  | 14:25 | 298 | 1.0 | 1.0 | 56.8 |
|  | 14:26 | 14 | .0 | .0 | 56.8 |
|  | 14:27 | 7 | .0 | .0 | 56.8 |
|  | 14:28 | 24 | .1 | .1 | 56.9 |
|  | 14:29 | 14 | .0 | .0 | 57.0 |
|  | 14:30 | 940 | 3.1 | 3.1 | 60.0 |
|  | 14:31 | 12 | .0 | .0 | 60.1 |
|  | 14:32 | 17 | .1 | .1 | 60.1 |
|  | 14:33 | 12 | .0 | .0 | 60.2 |
|  | 14:34 | 9 | .0 | .0 | 60.2 |
|  | 14:35 | 199 | .7 | .7 | 60.9 |
|  | 14:36 | 7 | .0 | .0 | 60.9 |
|  | 14:37 | 8 | .0 | .0 | 60.9 |
|  | 14:38 | 23 | .1 | .1 | 61.0 |
|  | 14:39 | 5 | .0 | .0 | 61.0 |
|  | 14:40 | 659 | 2.2 | 2.2 | 63.2 |
|  | 14:41 | 10 | .0 | .0 | 63.2 |
|  | 14:42 | 19 | .1 | .1 | 63.3 |
|  | 14:43 | 7 | .0 | .0 | 63.3 |
|  | 14:44 | 6 | .0 | .0 | 63.3 |
|  | 14:45 | 217 | .7 | .7 | 64.0 |
|  | 14:46 | 4 | .0 | .0 | 64.0 |
|  | 14:47 | 7 | .0 | .0 | 64.0 |
|  | 14:48 | 9 | .0 | .0 | 64.1 |
|  | 14:49 | 7 | .0 | .0 | 64.1 |
|  | 14:5 | 1 | .0 | .0 | 64.1 |
|  | 14:50 | 583 | 1.9 | 1.9 | 66.0 |
|  | 14:51 | 3 | .0 | .0 | 66.0 |
|  | 14:52 | 13 | .0 | .0 | 66.1 |
|  | 14:53 | 4 | .0 | .0 | 66.1 |
|  | 14:54 | 6 | .0 | .0 | 66.1 |
|  | 14:55 | 151 | .5 | .5 | 66.6 |
|  | 14:56 | 9 | .0 | .0 | 66.6 |
|  | 14:57 | 12 | .0 | .0 | 66.7 |
|  | 14:58 | 18 | .1 | .1 | 66.7 |
|  | 14:59 | 17 | .1 | .1 | 66.8 |
|  | 14:60 | 66 | .2 | .2 | 67.0 |
|  | 14:71 | 1 | .0 | .0 | 67.0 |
|  | 14:72 | 2 | .0 | .0 | 67.0 |
|  | 14:73 | 1 | .0 | .0 | 67.0 |
|  | 14:75 | 1 | .0 | .0 | 67.0 |
|  | 14:80 | 1 | .0 | .0 | 67.0 |
|  | 14:90 | 2 | .0 | .0 | 67.0 |
|  | 14?:1 | 1 | .0 | .0 | 67.0 |
|  | 14?:3 | 1 | .0 | .0 | 67.0 |
|  | 14.00 | 13 | .0 | .0 | 67.1 |
|  | 14.05 | 3 | .0 | .0 | 67.1 |
|  | 14.06 | 1 | .0 | .0 | 67.1 |
|  | 14.08 | 1 | .0 | .0 | 67.1 |
|  | 14.10 | 11 | .0 | .0 | 67.1 |
|  | 14.11 | 1 | .0 | .0 | 67.1 |
|  | 14.13 | 2 | .0 | .0 | 67.1 |
|  | 14.15 | 7 | .0 | .0 | 67.1 |
|  | 14.17 | 1 | .0 | .0 | 67.1 |
|  | 14.20 | 15 | .0 | .0 | 67.2 |
|  | 14.23 | 1 | .0 | .0 | 67.2 |
|  | 14.25 | 5 | .0 | .0 | 67.2 |
|  | 14.30 | 16 | .1 | .1 | 67.3 |
|  | 14.34 | 1 | .0 | .0 | 67.3 |
|  | 14.35 | 6 | .0 | .0 | 67.3 |
|  | 14.40 | 12 | .0 | .0 | 67.3 |
|  | 14.45 | 5 | .0 | .0 | 67.3 |
|  | 14.47 | 1 | .0 | .0 | 67.3 |
|  | 14.50 | 10 | .0 | .0 | 67.4 |
|  | 14.52 | 1 | .0 | .0 | 67.4 |
|  | 14.55 | 6 | .0 | .0 | 67.4 |
|  | 140 | 2 | .0 | .0 | 67.4 |
|  | 1400 | 94 | .3 | .3 | 67.7 |
|  | 1402 | 3 | .0 | .0 | 67.7 |
|  | 1405 | 20 | .1 | .1 | 67.8 |
|  | 1406 | 1 | .0 | .0 | 67.8 |
|  | 1407 | 1 | .0 | .0 | 67.8 |
|  | 1408 | 1 | .0 | .0 | 67.8 |
|  | 1409 | 1 | .0 | .0 | 67.8 |
|  | 141:1 | 1 | .0 | .0 | 67.8 |
|  | 1410 | 92 | .3 | .3 | 68.1 |
|  | 1411 | 2 | .0 | .0 | 68.1 |
|  | 14113 | 1 | .0 | .0 | 68.1 |
|  | 1412 | 1 | .0 | .0 | 68.1 |
|  | 1413 | 2 | .0 | .0 | 68.1 |
|  | 1415 | 39 | .1 | .1 | 68.3 |
|  | 1418 | 2 | .0 | .0 | 68.3 |
|  | 1420 | 103 | .3 | .3 | 68.6 |
|  | 1421 | 1 | .0 | .0 | 68.6 |
|  | 1422 | 2 | .0 | .0 | 68.6 |
|  | 1425 | 18 | .1 | .1 | 68.7 |
|  | 1426 | 4 | .0 | .0 | 68.7 |
|  | 1428 | 1 | .0 | .0 | 68.7 |
|  | 1429 | 1 | .0 | .0 | 68.7 |
|  | 1430 | 82 | .3 | .3 | 69.0 |
|  | 1431 | 1 | .0 | .0 | 69.0 |
|  | 1432 | 3 | .0 | .0 | 69.0 |
|  | 1434 | 1 | .0 | .0 | 69.0 |
|  | 1435 | 17 | .1 | .1 | 69.0 |
|  | 1436 | 1 | .0 | .0 | 69.0 |
|  | 1438 | 1 | .0 | .0 | 69.0 |
|  | 1440 | 52 | .2 | .2 | 69.2 |
|  | 1442 | 3 | .0 | .0 | 69.2 |
|  | 1445 | 20 | .1 | .1 | 69.3 |
|  | 1446 | 2 | .0 | .0 | 69.3 |
|  | 1447 | 1 | .0 | .0 | 69.3 |
|  | 145 | 1 | .0 | .0 | 69.3 |
|  | 145:2 | 1 | .0 | .0 | 69.3 |
|  | 1450 | 53 | .2 | .2 | 69.5 |
|  | 1451 | 1 | .0 | .0 | 69.5 |
|  | 1452 | 1 | .0 | .0 | 69.5 |
|  | 1454 | 1 | .0 | .0 | 69.5 |
|  | 1455 | 4 | .0 | .0 | 69.5 |
|  | 1456 | 1 | .0 | .0 | 69.5 |
|  | 1458 | 2 | .0 | .0 | 69.5 |
|  | 1460 | 9 | .0 | .0 | 69.5 |
|  | 147:0 | 1 | .0 | .0 | 69.5 |
|  | 1475 | 1 | .0 | .0 | 69.5 |
|  | 15 | 3 | .0 | .0 | 69.6 |
|  | 15;'0 | 2 | .0 | .0 | 69.6 |
|  | 15;'1 | 1 | .0 | .0 | 69.6 |
|  | 15;00 | 1 | .0 | .0 | 69.6 |
|  | 15;05 | 5 | .0 | .0 | 69.6 |
|  | 15;10 | 1 | .0 | .0 | 69.6 |
|  | 15;16 | 1 | .0 | .0 | 69.6 |
|  | 15;20 | 4 | .0 | .0 | 69.6 |
|  | 15;25 | 1 | .0 | .0 | 69.6 |
|  | 15;30 | 2 | .0 | .0 | 69.6 |
|  | 15;31 | 1 | .0 | .0 | 69.6 |
|  | 15;40 | 1 | .0 | .0 | 69.6 |
|  | 15;50 | 7 | .0 | .0 | 69.6 |
|  | 15;55 | 1 | .0 | .0 | 69.6 |
|  | 15;56 | 1 | .0 | .0 | 69.6 |
|  | 15:00 | 674 | 2.2 | 2.2 | 71.9 |
|  | 15:02 | 14 | .0 | .0 | 71.9 |
|  | 15:03 | 8 | .0 | .0 | 71.9 |
|  | 15:04 | 4 | .0 | .0 | 71.9 |
|  | 15:05 | 160 | .5 | .5 | 72.5 |
|  | 15:06 | 7 | .0 | .0 | 72.5 |
|  | 15:07 | 10 | .0 | .0 | 72.5 |
|  | 15:08 | 11 | .0 | .0 | 72.6 |
|  | 15:09 | 7 | .0 | .0 | 72.6 |
|  | 15:10 | 560 | 1.8 | 1.8 | 74.4 |
|  | 15:11 | 5 | .0 | .0 | 74.4 |
|  | 15:12 | 17 | .1 | .1 | 74.5 |
|  | 15:13 | 23 | .1 | .1 | 74.6 |
|  | 15:14 | 12 | .0 | .0 | 74.6 |
|  | 15:15 | 171 | .6 | .6 | 75.2 |
|  | 15:16 | 7 | .0 | .0 | 75.2 |
|  | 15:17 | 12 | .0 | .0 | 75.2 |
|  | 15:18 | 45 | .1 | .1 | 75.4 |
|  | 15:19 | 3 | .0 | .0 | 75.4 |
|  | 15:20 | 606 | 2.0 | 2.0 | 77.4 |
|  | 15:21 | 10 | .0 | .0 | 77.4 |
|  | 15:22 | 9 | .0 | .0 | 77.4 |
|  | 15:23 | 6 | .0 | .0 | 77.4 |
|  | 15:24 | 5 | .0 | .0 | 77.5 |
|  | 15:26 | 14 | .0 | .0 | 77.5 |
|  | 15:27 | 6 | .0 | .0 | 77.5 |
|  | 15:28 | 13 | .0 | .0 | 77.6 |
|  | 15:29 | 4 | .0 | .0 | 77.6 |
|  | 15:30 | 923 | 3.0 | 3.0 | 80.6 |
|  | 15:31 | 4 | .0 | .0 | 80.6 |
|  | 15:32 | 11 | .0 | .0 | 80.6 |
|  | 15:33 | 7 | .0 | .0 | 80.7 |
|  | 15:34 | 7 | .0 | .0 | 80.7 |
|  | 15:35 | 148 | .5 | .5 | 81.2 |
|  | 15:36 | 13 | .0 | .0 | 81.2 |
|  | 15:37 | 9 | .0 | .0 | 81.2 |
|  | 15:38 | 15 | .0 | .0 | 81.3 |
|  | 15:39 | 1 | .0 | .0 | 81.3 |
|  | 15:40 | 442 | 1.4 | 1.4 | 82.7 |
|  | 15:41 | 4 | .0 | .0 | 82.8 |
|  | 15:42 | 14 | .0 | .0 | 82.8 |
|  | 15:43 | 4 | .0 | .0 | 82.8 |
|  | 15:44 | 3 | .0 | .0 | 82.8 |
|  | 15:45 | 170 | .6 | .6 | 83.4 |
|  | 15:46 | 7 | .0 | .0 | 83.4 |
|  | 15:47 | 8 | .0 | .0 | 83.4 |
|  | 15:48 | 8 | .0 | .0 | 83.5 |
|  | 15:49 | 3 | .0 | .0 | 83.5 |
|  | 15:50 | 406 | 1.3 | 1.3 | 84.8 |
|  | 15:51 | 7 | .0 | .0 | 84.8 |
|  | 15:52 | 4 | .0 | .0 | 84.8 |
|  | 15:53 | 7 | .0 | .0 | 84.9 |
|  | 15:54 | 3 | .0 | .0 | 84.9 |
|  | 15:55 | 78 | .3 | .3 | 85.1 |
|  | 15:56 | 9 | .0 | .0 | 85.2 |
|  | 15:57 | 6 | .0 | .0 | 85.2 |
|  | 15:58 | 7 | .0 | .0 | 85.2 |
|  | 15:59 | 14 | .0 | .0 | 85.2 |
|  | 15:60 | 39 | .1 | .1 | 85.4 |
|  | 15:62 | 3 | .0 | .0 | 85.4 |
|  | 15:66 | 1 | .0 | .0 | 85.4 |
|  | 15:70 | 2 | .0 | .0 | 85.4 |
|  | 15:75 | 1 | .0 | .0 | 85.4 |
|  | 15:8 | 1 | .0 | .0 | 85.4 |
|  | 15:82 | 1 | .0 | .0 | 85.4 |
|  | 15:90 | 2 | .0 | .0 | 85.4 |
|  | 15:L0 | 1 | .0 | .0 | 85.4 |
|  | 15:L2 | 1 | .0 | .0 | 85.4 |
|  | 15.00 | 10 | .0 | .0 | 85.4 |
|  | 15.04 | 1 | .0 | .0 | 85.4 |
|  | 15.05 | 8 | .0 | .0 | 85.5 |
|  | 15.10 | 7 | .0 | .0 | 85.5 |
|  | 15.12 | 2 | .0 | .0 | 85.5 |
|  | 15.13 | 1 | .0 | .0 | 85.5 |
|  | 15.15 | 2 | .0 | .0 | 85.5 |
|  | 15.16 | 1 | .0 | .0 | 85.5 |
|  | 15.20 | 10 | .0 | .0 | 85.5 |
|  | 15.22 | 1 | .0 | .0 | 85.6 |
|  | 15.24 | 1 | .0 | .0 | 85.6 |
|  | 15.25 | 2 | .0 | .0 | 85.6 |
|  | 15.27 | 1 | .0 | .0 | 85.6 |
|  | 15.30 | 9 | .0 | .0 | 85.6 |
|  | 15.32 | 1 | .0 | .0 | 85.6 |
|  | 15.33 | 2 | .0 | .0 | 85.6 |
|  | 15.34 | 1 | .0 | .0 | 85.6 |
|  | 15.35 | 1 | .0 | .0 | 85.6 |
|  | 15.40 | 5 | .0 | .0 | 85.6 |
|  | 15.43 | 1 | .0 | .0 | 85.6 |
|  | 15.45 | 1 | .0 | .0 | 85.6 |
|  | 15.49 | 1 | .0 | .0 | 85.6 |
|  | 15.50 | 4 | .0 | .0 | 85.7 |
|  | 15.53 | 1 | .0 | .0 | 85.7 |
|  | 15.55 | 6 | .0 | .0 | 85.7 |
|  | 15"18 | 1 | .0 | .0 | 85.7 |
|  | 15"50 | 2 | .0 | .0 | 85.7 |
|  | 15"56 | 1 | .0 | .0 | 85.7 |
|  | 150 | 2 | .0 | .0 | 85.7 |
|  | 1500 | 55 | .2 | .2 | 85.9 |
|  | 1502 | 2 | .0 | .0 | 85.9 |
|  | 1503 | 1 | .0 | .0 | 85.9 |
|  | 1504 | 1 | .0 | .0 | 85.9 |
|  | 1505 | 9 | .0 | .0 | 85.9 |
|  | 1506 | 2 | .0 | .0 | 85.9 |
|  | 1507 | 1 | .0 | .0 | 85.9 |
|  | 1508 | 2 | .0 | .0 | 85.9 |
|  | 1510 | 35 | .1 | .1 | 86.0 |
|  | 1513 | 1 | .0 | .0 | 86.1 |
|  | 1515 | 9 | .0 | .0 | 86.1 |
|  | 1517 | 1 | .0 | .0 | 86.1 |
|  | 1518 | 1 | .0 | .0 | 86.1 |
|  | 1519 | 1 | .0 | .0 | 86.1 |
|  | 1520 | 55 | .2 | .2 | 86.3 |
|  | 1523 | 1 | .0 | .0 | 86.3 |
|  | 1524 | 2 | .0 | .0 | 86.3 |
|  | 1525 | 12 | .0 | .0 | 86.3 |
|  | 1527 | 2 | .0 | .0 | 86.3 |
|  | 1528 | 1 | .0 | .0 | 86.3 |
|  | 1530 | 56 | .2 | .2 | 86.5 |
|  | 1533 | 2 | .0 | .0 | 86.5 |
|  | 1534 | 1 | .0 | .0 | 86.5 |
|  | 1535 | 13 | .0 | .0 | 86.6 |
|  | 1538 | 1 | .0 | .0 | 86.6 |
|  | 154 | 1 | .0 | .0 | 86.6 |
|  | 1540 | 36 | .1 | .1 | 86.7 |
|  | 1542 | 1 | .0 | .0 | 86.7 |
|  | 1543 | 1 | .0 | .0 | 86.7 |
|  | 1545 | 16 | .1 | .1 | 86.7 |
|  | 1547 | 1 | .0 | .0 | 86.8 |
|  | 1548 | 1 | .0 | .0 | 86.8 |
|  | 1550 | 29 | .1 | .1 | 86.8 |
|  | 1555 | 7 | .0 | .0 | 86.9 |
|  | 1560 | 1 | .0 | .0 | 86.9 |
|  | 16;05 | 1 | .0 | .0 | 86.9 |
|  | 16;15 | 1 | .0 | .0 | 86.9 |
|  | 16;20 | 2 | .0 | .0 | 86.9 |
|  | 16;35 | 1 | .0 | .0 | 86.9 |
|  | 16;40 | 2 | .0 | .0 | 86.9 |
|  | 16:0 | 1 | .0 | .0 | 86.9 |
|  | 16:00 | 382 | 1.3 | 1.3 | 88.2 |
|  | 16:02 | 6 | .0 | .0 | 88.2 |
|  | 16:03 | 7 | .0 | .0 | 88.2 |
|  | 16:04 | 1 | .0 | .0 | 88.2 |
|  | 16:05 | 73 | .2 | .2 | 88.4 |
|  | 16:06 | 3 | .0 | .0 | 88.4 |
|  | 16:07 | 2 | .0 | .0 | 88.5 |
|  | 16:08 | 7 | .0 | .0 | 88.5 |
|  | 16:09 | 7 | .0 | .0 | 88.5 |
|  | 16:10 | 292 | 1.0 | 1.0 | 89.5 |
|  | 16:11 | 4 | .0 | .0 | 89.5 |
|  | 16:12 | 7 | .0 | .0 | 89.5 |
|  | 16:13 | 20 | .1 | .1 | 89.6 |
|  | 16:14 | 9 | .0 | .0 | 89.6 |
|  | 16:15 | 101 | .3 | .3 | 89.9 |
|  | 16:16 | 6 | .0 | .0 | 89.9 |
|  | 16:17 | 9 | .0 | .0 | 90.0 |
|  | 16:18 | 6 | .0 | .0 | 90.0 |
|  | 16:19 | 3 | .0 | .0 | 90.0 |
|  | 16:20 | 298 | 1.0 | 1.0 | 91.0 |
|  | 16:21 | 4 | .0 | .0 | 91.0 |
|  | 16:22 | 13 | .0 | .0 | 91.0 |
|  | 16:23 | 3 | .0 | .0 | 91.0 |
|  | 16:24 | 3 | .0 | .0 | 91.0 |
|  | 16:25 | 87 | .3 | .3 | 91.3 |
|  | 16:26 | 4 | .0 | .0 | 91.3 |
|  | 16:27 | 7 | .0 | .0 | 91.4 |
|  | 16:28 | 7 | .0 | .0 | 91.4 |
|  | 16:29 | 5 | .0 | .0 | 91.4 |
|  | 16:30 | 299 | 1.0 | 1.0 | 92.4 |
|  | 16:31 | 7 | .0 | .0 | 92.4 |
|  | 16:32 | 11 | .0 | .0 | 92.4 |
|  | 16:33 | 1 | .0 | .0 | 92.4 |
|  | 16:34 | 3 | .0 | .0 | 92.5 |
|  | 16:35 | 58 | .2 | .2 | 92.6 |
|  | 16:36 | 5 | .0 | .0 | 92.7 |
|  | 16:37 | 6 | .0 | .0 | 92.7 |
|  | 16:38 | 7 | .0 | .0 | 92.7 |
|  | 16:39 | 5 | .0 | .0 | 92.7 |
|  | 16:40 | 214 | .7 | .7 | 93.4 |
|  | 16:41 | 4 | .0 | .0 | 93.4 |
|  | 16:42 | 4 | .0 | .0 | 93.4 |
|  | 16:43 | 2 | .0 | .0 | 93.5 |
|  | 16:44 | 5 | .0 | .0 | 93.5 |
|  | 16:45 | 77 | .3 | .3 | 93.7 |
|  | 16:46 | 5 | .0 | .0 | 93.7 |
|  | 16:47 | 5 | .0 | .0 | 93.8 |
|  | 16:48 | 6 | .0 | .0 | 93.8 |
|  | 16:49 | 6 | .0 | .0 | 93.8 |
|  | 16:50 | 167 | .5 | .5 | 94.3 |
|  | 16:51 | 2 | .0 | .0 | 94.3 |
|  | 16:52 | 8 | .0 | .0 | 94.4 |
|  | 16:53 | 5 | .0 | .0 | 94.4 |
|  | 16:55 | 49 | .2 | .2 | 94.6 |
|  | 16:56 | 2 | .0 | .0 | 94.6 |
|  | 16:57 | 2 | .0 | .0 | 94.6 |
|  | 16:58 | 7 | .0 | .0 | 94.6 |
|  | 16:59 | 13 | .0 | .0 | 94.6 |
|  | 16:60 | 14 | .0 | .0 | 94.7 |
|  | 16?:2 | 1 | .0 | .0 | 94.7 |
|  | 16.00 | 2 | .0 | .0 | 94.7 |
|  | 16.05 | 1 | .0 | .0 | 94.7 |
|  | 16.06 | 1 | .0 | .0 | 94.7 |
|  | 16.09 | 1 | .0 | .0 | 94.7 |
|  | 16.10 | 2 | .0 | .0 | 94.7 |
|  | 16.12 | 1 | .0 | .0 | 94.7 |
|  | 16.15 | 2 | .0 | .0 | 94.7 |
|  | 16.17 | 1 | .0 | .0 | 94.7 |
|  | 16.20 | 6 | .0 | .0 | 94.7 |
|  | 16.25 | 4 | .0 | .0 | 94.7 |
|  | 16.30 | 4 | .0 | .0 | 94.8 |
|  | 16.34 | 1 | .0 | .0 | 94.8 |
|  | 16.35 | 2 | .0 | .0 | 94.8 |
|  | 16.40 | 1 | .0 | .0 | 94.8 |
|  | 16.50 | 1 | .0 | .0 | 94.8 |
|  | 16.55 | 1 | .0 | .0 | 94.8 |
|  | 16.58 | 1 | .0 | .0 | 94.8 |
|  | 1600 | 22 | .1 | .1 | 94.9 |
|  | 1605 | 5 | .0 | .0 | 94.9 |
|  | 1606 | 1 | .0 | .0 | 94.9 |
|  | 1610 | 14 | .0 | .0 | 94.9 |
|  | 1612 | 1 | .0 | .0 | 94.9 |
|  | 1613 | 1 | .0 | .0 | 94.9 |
|  | 1615 | 4 | .0 | .0 | 94.9 |
|  | 1618 | 1 | .0 | .0 | 94.9 |
|  | 1620 | 13 | .0 | .0 | 95.0 |
|  | 1621 | 1 | .0 | .0 | 95.0 |
|  | 1625 | 2 | .0 | .0 | 95.0 |
|  | 1629 | 1 | .0 | .0 | 95.0 |
|  | 1630 | 13 | .0 | .0 | 95.0 |
|  | 1631 | 1 | .0 | .0 | 95.0 |
|  | 1634 | 1 | .0 | .0 | 95.0 |
|  | 1636 | 1 | .0 | .0 | 95.1 |
|  | 1640 | 6 | .0 | .0 | 95.1 |
|  | 1642 | 1 | .0 | .0 | 95.1 |
|  | 1645 | 1 | .0 | .0 | 95.1 |
|  | 1646 | 1 | .0 | .0 | 95.1 |
|  | 1650 | 3 | .0 | .0 | 95.1 |
|  | 1651 | 1 | .0 | .0 | 95.1 |
|  | 1652 | 1 | .0 | .0 | 95.1 |
|  | 1655 | 2 | .0 | .0 | 95.1 |
|  | 1656 | 1 | .0 | .0 | 95.1 |
|  | 17;20 | 3 | .0 | .0 | 95.1 |
|  | 17:00 | 205 | .7 | .7 | 95.8 |
|  | 17:02 | 7 | .0 | .0 | 95.8 |
|  | 17:03 | 2 | .0 | .0 | 95.8 |
|  | 17:04 | 3 | .0 | .0 | 95.8 |
|  | 17:05 | 25 | .1 | .1 | 95.9 |
|  | 17:06 | 2 | .0 | .0 | 95.9 |
|  | 17:08 | 1 | .0 | .0 | 95.9 |
|  | 17:09 | 1 | .0 | .0 | 95.9 |
|  | 17:10 | 201 | .7 | .7 | 96.6 |
|  | 17:11 | 5 | .0 | .0 | 96.6 |
|  | 17:12 | 7 | .0 | .0 | 96.6 |
|  | 17:13 | 5 | .0 | .0 | 96.6 |
|  | 17:15 | 35 | .1 | .1 | 96.7 |
|  | 17:16 | 2 | .0 | .0 | 96.8 |
|  | 17:17 | 2 | .0 | .0 | 96.8 |
|  | 17:18 | 5 | .0 | .0 | 96.8 |
|  | 17:19 | 1 | .0 | .0 | 96.8 |
|  | 17:20 | 115 | .4 | .4 | 97.2 |
|  | 17:21 | 2 | .0 | .0 | 97.2 |
|  | 17:22 | 3 | .0 | .0 | 97.2 |
|  | 17:23 | 2 | .0 | .0 | 97.2 |
|  | 17:24 | 3 | .0 | .0 | 97.2 |
|  | 17:25 | 18 | .1 | .1 | 97.3 |
|  | 17:26 | 3 | .0 | .0 | 97.3 |
|  | 17:27 | 2 | .0 | .0 | 97.3 |
|  | 17:28 | 6 | .0 | .0 | 97.3 |
|  | 17:29 | 1 | .0 | .0 | 97.3 |
|  | 17:30 | 86 | .3 | .3 | 97.6 |
|  | 17:31 | 3 | .0 | .0 | 97.6 |
|  | 17:32 | 1 | .0 | .0 | 97.6 |
|  | 17:33 | 2 | .0 | .0 | 97.6 |
|  | 17:34 | 1 | .0 | .0 | 97.6 |
|  | 17:35 | 15 | .0 | .0 | 97.6 |
|  | 17:36 | 3 | .0 | .0 | 97.7 |
|  | 17:37 | 1 | .0 | .0 | 97.7 |
|  | 17:38 | 2 | .0 | .0 | 97.7 |
|  | 17:39 | 2 | .0 | .0 | 97.7 |
|  | 17:40 | 109 | .4 | .4 | 98.0 |
|  | 17:42 | 4 | .0 | .0 | 98.0 |
|  | 17:44 | 1 | .0 | .0 | 98.0 |
|  | 17:45 | 24 | .1 | .1 | 98.1 |
|  | 17:47 | 1 | .0 | .0 | 98.1 |
|  | 17:48 | 2 | .0 | .0 | 98.1 |
|  | 17:49 | 4 | .0 | .0 | 98.1 |
|  | 17:50 | 36 | .1 | .1 | 98.3 |
|  | 17:54 | 5 | .0 | .0 | 98.3 |
|  | 17:55 | 8 | .0 | .0 | 98.3 |
|  | 17:56 | 2 | .0 | .0 | 98.3 |
|  | 17:57 | 5 | .0 | .0 | 98.3 |
|  | 17:59 | 1 | .0 | .0 | 98.3 |
|  | 17:60 | 4 | .0 | .0 | 98.3 |
|  | 17.0 | 1 | .0 | .0 | 98.3 |
|  | 17.00 | 1 | .0 | .0 | 98.4 |
|  | 17.05 | 1 | .0 | .0 | 98.4 |
|  | 17.07 | 1 | .0 | .0 | 98.4 |
|  | 17.11 | 1 | .0 | .0 | 98.4 |
|  | 17.19 | 1 | .0 | .0 | 98.4 |
|  | 17.20 | 3 | .0 | .0 | 98.4 |
|  | 17.25 | 2 | .0 | .0 | 98.4 |
|  | 17.28 | 1 | .0 | .0 | 98.4 |
|  | 17.40 | 2 | .0 | .0 | 98.4 |
|  | 17.41 | 1 | .0 | .0 | 98.4 |
|  | 17.45 | 1 | .0 | .0 | 98.4 |
|  | 17.50 | 1 | .0 | .0 | 98.4 |
|  | 1700 | 4 | .0 | .0 | 98.4 |
|  | 1705 | 1 | .0 | .0 | 98.4 |
|  | 1707 | 1 | .0 | .0 | 98.4 |
|  | 1709 | 1 | .0 | .0 | 98.4 |
|  | 1710 | 2 | .0 | .0 | 98.4 |
|  | 1720 | 2 | .0 | .0 | 98.4 |
|  | 1730 | 1 | .0 | .0 | 98.4 |
|  | 1735 | 1 | .0 | .0 | 98.4 |
|  | 1740 | 1 | .0 | .0 | 98.4 |
|  | 1741 | 1 | .0 | .0 | 98.4 |
|  | 1745 | 1 | .0 | .0 | 98.5 |
|  | 1748 | 1 | .0 | .0 | 98.5 |
|  | 1750 | 1 | .0 | .0 | 98.5 |
|  | 1755 | 1 | .0 | .0 | 98.5 |
|  | 18:00 | 85 | .3 | .3 | 98.7 |
|  | 18:03 | 2 | .0 | .0 | 98.7 |
|  | 18:04 | 1 | .0 | .0 | 98.7 |
|  | 18:05 | 19 | .1 | .1 | 98.8 |
|  | 18:07 | 2 | .0 | .0 | 98.8 |
|  | 18:08 | 1 | .0 | .0 | 98.8 |
|  | 18:10 | 22 | .1 | .1 | 98.9 |
|  | 18:11 | 1 | .0 | .0 | 98.9 |
|  | 18:15 | 2 | .0 | .0 | 98.9 |
|  | 18:19 | 1 | .0 | .0 | 98.9 |
|  | 18:20 | 6 | .0 | .0 | 98.9 |
|  | 18:23 | 1 | .0 | .0 | 98.9 |
|  | 18:24 | 1 | .0 | .0 | 98.9 |
|  | 18:25 | 1 | .0 | .0 | 98.9 |
|  | 18:26 | 1 | .0 | .0 | 98.9 |
|  | 18:27 | 1 | .0 | .0 | 98.9 |
|  | 18:29 | 1 | .0 | .0 | 98.9 |
|  | 18:30 | 8 | .0 | .0 | 99.0 |
|  | 18:34 | 1 | .0 | .0 | 99.0 |
|  | 18:36 | 1 | .0 | .0 | 99.0 |
|  | 18:40 | 4 | .0 | .0 | 99.0 |
|  | 18:41 | 1 | .0 | .0 | 99.0 |
|  | 18:45 | 2 | .0 | .0 | 99.0 |
|  | 18:50 | 15 | .0 | .0 | 99.1 |
|  | 18:60 | 2 | .0 | .0 | 99.1 |
|  | 18.00 | 1 | .0 | .0 | 99.1 |
|  | 18.13 | 1 | .0 | .0 | 99.1 |
|  | 18.20 | 1 | .0 | .0 | 99.1 |
|  | 18.26 | 1 | .0 | .0 | 99.1 |
|  | 18.40 | 1 | .0 | .0 | 99.1 |
|  | 1800 | 2 | .0 | .0 | 99.1 |
|  | 1810 | 1 | .0 | .0 | 99.1 |
|  | 19:00 | 41 | .1 | .1 | 99.2 |
|  | 19:03 | 1 | .0 | .0 | 99.2 |
|  | 19:04 | 1 | .0 | .0 | 99.2 |
|  | 19:05 | 13 | .0 | .0 | 99.3 |
|  | 19:10 | 27 | .1 | .1 | 99.4 |
|  | 19:15 | 14 | .0 | .0 | 99.4 |
|  | 19:20 | 43 | .1 | .1 | 99.5 |
|  | 19:25 | 18 | .1 | .1 | 99.6 |
|  | 19:26 | 1 | .0 | .0 | 99.6 |
|  | 19:30 | 33 | .1 | .1 | 99.7 |
|  | 19:35 | 11 | .0 | .0 | 99.7 |
|  | 19:40 | 24 | .1 | .1 | 99.8 |
|  | 19:45 | 13 | .0 | .0 | 99.9 |
|  | 19:50 | 24 | .1 | .1 | 99.9 |
|  | 19:55 | 16 | .1 | .1 | 100.0 |
|  | Total | 30553 | 100.0 | 100.0 |  |
|  |  |  |  |  |  |

Interview end timeInterview end time, table, 1 levels of column headers and 2 levels of row headers, table with 6 columns and 3 rows

|  |  |  |  |  |  |
| --- | --- | --- | --- | --- | --- |
|  | | Frequency | Percent | Valid Percent | Cumulative Percent |
| Valid |  | 30553 | 100.0 | 100.0 | 100.0 |
|  |  |  |  |  |  |

Sex of the individualSex of the individual, table, 1 levels of column headers and 2 levels of row headers, table with 6 columns and 5 rows

|  |  |  |  |  |  |
| --- | --- | --- | --- | --- | --- |
|  | | Frequency | Percent | Valid Percent | Cumulative Percent |
| Valid | Male | 15302 | 50.1 | 50.1 | 50.1 |
|  | Female | 15251 | 49.9 | 49.9 | 100.0 |
|  | Total | 30553 | 100.0 | 100.0 |  |
|  |  |  |  |  |  |

mother's serial numbermother's serial number, table, 1 levels of column headers and 2 levels of row headers, table with 6 columns and 13 rows

|  |  |  |  |  |  |
| --- | --- | --- | --- | --- | --- |
|  | | Frequency | Percent | Valid Percent | Cumulative Percent |
| Valid | 1 | 1076 | 3.5 | 3.5 | 3.5 |
|  | 2 | 20362 | 66.6 | 66.6 | 70.2 |
|  | 3 | 3509 | 11.5 | 11.5 | 81.7 |
|  | 4 | 3825 | 12.5 | 12.5 | 94.2 |
|  | 5 | 890 | 2.9 | 2.9 | 97.1 |
|  | 6 | 531 | 1.7 | 1.7 | 98.8 |
|  | 7 | 198 | .6 | .6 | 99.5 |
|  | 8 | 99 | .3 | .3 | 99.8 |
|  | 9 | 44 | .1 | .1 | 99.9 |
|  | 10 | 19 | .1 | .1 | 100.0 |
|  | Total | 30553 | 100.0 | 100.0 |  |
|  |  |  |  |  |  |

relationship of primary caregiver with childrelationship of primary caregiver with child, table, 1 levels of column headers and 2 levels of row headers, table with 6 columns and 14 rows

|  |  |  |  |  |  |
| --- | --- | --- | --- | --- | --- |
|  | | Frequency | Percent | Valid Percent | Cumulative Percent |
| Valid | Paternal grandmother | 756 | 2.5 | 71.5 | 71.5 |
|  | Paternal grandfather | 44 | .1 | 4.2 | 75.7 |
|  | Father | 68 | .2 | 6.4 | 82.1 |
|  | Maternal grandfather | 8 | .0 | .8 | 82.9 |
|  | Maternal grandmother | 84 | .3 | 7.9 | 90.8 |
|  | Sibling | 53 | .2 | 5.0 | 95.8 |
|  | Cousin | 4 | .0 | .4 | 96.2 |
|  | Aunt/uncle | 22 | .1 | 2.1 | 98.3 |
|  | Others(not coded) code start from 09 | 18 | .1 | 1.7 | 100.0 |
|  | Total | 1057 | 3.5 | 100.0 |  |
| Missing | System | 29496 | 96.5 |  |  |
| Total | | 30553 | 100.0 |  |  |
|  |  |  |  |  |  |

Age of the primary caregiverAge of the primary caregiver, table, 1 levels of column headers and 2 levels of row headers, table with 6 columns and 69 rows

|  |  |  |  |  |  |
| --- | --- | --- | --- | --- | --- |
|  | | Frequency | Percent | Valid Percent | Cumulative Percent |
| Valid | 7 | 1 | .0 | .1 | .1 |
|  | 8 | 4 | .0 | .4 | .5 |
|  | 10 | 23 | .1 | 2.2 | 2.6 |
|  | 11 | 11 | .0 | 1.0 | 3.7 |
|  | 12 | 10 | .0 | .9 | 4.6 |
|  | 13 | 8 | .0 | .8 | 5.4 |
|  | 15 | 7 | .0 | .7 | 6.1 |
|  | 16 | 1 | .0 | .1 | 6.1 |
|  | 17 | 1 | .0 | .1 | 6.2 |
|  | 18 | 4 | .0 | .4 | 6.6 |
|  | 19 | 4 | .0 | .4 | 7.0 |
|  | 20 | 2 | .0 | .2 | 7.2 |
|  | 21 | 1 | .0 | .1 | 7.3 |
|  | 22 | 2 | .0 | .2 | 7.5 |
|  | 23 | 1 | .0 | .1 | 7.6 |
|  | 24 | 4 | .0 | .4 | 7.9 |
|  | 25 | 16 | .1 | 1.5 | 9.5 |
|  | 26 | 1 | .0 | .1 | 9.6 |
|  | 28 | 1 | .0 | .1 | 9.6 |
|  | 30 | 19 | .1 | 1.8 | 11.4 |
|  | 32 | 1 | .0 | .1 | 11.5 |
|  | 33 | 1 | .0 | .1 | 11.6 |
|  | 34 | 1 | .0 | .1 | 11.7 |
|  | 35 | 40 | .1 | 3.8 | 15.5 |
|  | 36 | 18 | .1 | 1.7 | 17.2 |
|  | 37 | 2 | .0 | .2 | 17.4 |
|  | 38 | 16 | .1 | 1.5 | 18.9 |
|  | 39 | 14 | .0 | 1.3 | 20.2 |
|  | 40 | 127 | .4 | 12.0 | 32.3 |
|  | 41 | 15 | .0 | 1.4 | 33.7 |
|  | 42 | 31 | .1 | 2.9 | 36.6 |
|  | 43 | 31 | .1 | 2.9 | 39.5 |
|  | 44 | 7 | .0 | .7 | 40.2 |
|  | 45 | 73 | .2 | 6.9 | 47.1 |
|  | 46 | 7 | .0 | .7 | 47.8 |
|  | 47 | 10 | .0 | .9 | 48.7 |
|  | 48 | 26 | .1 | 2.5 | 51.2 |
|  | 49 | 3 | .0 | .3 | 51.5 |
|  | 50 | 131 | .4 | 12.4 | 63.9 |
|  | 51 | 9 | .0 | .9 | 64.7 |
|  | 52 | 24 | .1 | 2.3 | 67.0 |
|  | 53 | 2 | .0 | .2 | 67.2 |
|  | 54 | 1 | .0 | .1 | 67.3 |
|  | 55 | 25 | .1 | 2.4 | 69.6 |
|  | 56 | 25 | .1 | 2.4 | 72.0 |
|  | 57 | 22 | .1 | 2.1 | 74.1 |
|  | 58 | 4 | .0 | .4 | 74.5 |
|  | 59 | 17 | .1 | 1.6 | 76.1 |
|  | 60 | 72 | .2 | 6.8 | 82.9 |
|  | 61 | 5 | .0 | .5 | 83.3 |
|  | 62 | 23 | .1 | 2.2 | 85.5 |
|  | 63 | 20 | .1 | 1.9 | 87.4 |
|  | 64 | 5 | .0 | .5 | 87.9 |
|  | 65 | 9 | .0 | .9 | 88.7 |
|  | 66 | 3 | .0 | .3 | 89.0 |
|  | 67 | 13 | .0 | 1.2 | 90.3 |
|  | 69 | 2 | .0 | .2 | 90.4 |
|  | 70 | 37 | .1 | 3.5 | 93.9 |
|  | 75 | 8 | .0 | .8 | 94.7 |
|  | 77 | 26 | .1 | 2.5 | 97.2 |
|  | 78 | 4 | .0 | .4 | 97.5 |
|  | 80 | 18 | .1 | 1.7 | 99.2 |
|  | 82 | 2 | .0 | .2 | 99.4 |
|  | 83 | 6 | .0 | .6 | 100.0 |
|  | Total | 1057 | 3.5 | 100.0 |  |
| Missing | System | 29496 | 96.5 |  |  |
| Total | | 30553 | 100.0 |  |  |
|  |  |  |  |  |  |

Where the baby stay at the time of visitWhere the baby stay at the time of visit, table, 1 levels of column headers and 2 levels of row headers, table with 6 columns and 8 rows

|  |  |  |  |  |  |
| --- | --- | --- | --- | --- | --- |
|  | | Frequency | Percent | Valid Percent | Cumulative Percent |
| Valid | Inside the house | 12383 | 40.5 | 40.5 | 40.5 |
|  | Yard | 11287 | 36.9 | 36.9 | 77.5 |
|  | Outside the bari | 6191 | 20.3 | 20.3 | 97.7 |
|  | Anchal | 391 | 1.3 | 1.3 | 99.0 |
|  | Others (not coded) code start from 04 | 301 | 1.0 | 1.0 | 100.0 |
|  | Total | 30553 | 100.0 | 100.0 |  |
|  |  |  |  |  |  |

Whether the child was in the playpenWhether the child was in the playpen, table, 1 levels of column headers and 2 levels of row headers, table with 6 columns and 7 rows

|  |  |  |  |  |  |
| --- | --- | --- | --- | --- | --- |
|  | | Frequency | Percent | Valid Percent | Cumulative Percent |
| Valid | Yes | 15053 | 49.3 | 63.6 | 63.6 |
|  | No | 8617 | 28.2 | 36.4 | 100.0 |
|  | Total | 23670 | 77.5 | 100.0 |  |
| Missing | System | 6883 | 22.5 |  |  |
| Total | | 30553 | 100.0 |  |  |
|  |  |  |  |  |  |

What the child doing inside the playpenWhat the child doing inside the playpen, table, 1 levels of column headers and 2 levels of row headers, table with 6 columns and 9 rows

|  |  |  |  |  |  |
| --- | --- | --- | --- | --- | --- |
|  | | Frequency | Percent | Valid Percent | Cumulative Percent |
| Valid | Sleeping | 1075 | 3.5 | 7.1 | 7.1 |
|  | Playing | 13136 | 43.0 | 87.3 | 94.4 |
|  | Crying | 795 | 2.6 | 5.3 | 99.7 |
|  | Others (not coded) Others code will be start from 04 | 47 | .2 | .3 | 100.0 |
|  | Total | 15053 | 49.3 | 100.0 |  |
| Missing | System | 15500 | 50.7 |  |  |
| Total | | 30553 | 100.0 |  |  |
|  |  |  |  |  |  |

Location of playpenLocation of playpen, table, 1 levels of column headers and 2 levels of row headers, table with 6 columns and 11 rows

|  |  |  |  |  |  |
| --- | --- | --- | --- | --- | --- |
|  | | Frequency | Percent | Valid Percent | Cumulative Percent |
| Valid | Inside the house | 12685 | 41.5 | 41.5 | 41.5 |
|  | In the cowshed | 392 | 1.3 | 1.3 | 42.8 |
|  | Yard | 12000 | 39.3 | 39.3 | 82.1 |
|  | Kitchen | 682 | 2.2 | 2.2 | 84.3 |
|  | Rooftop | 631 | 2.1 | 2.1 | 86.4 |
|  | Not in the house | 80 | .3 | .3 | 86.6 |
|  | Porch | 4062 | 13.3 | 13.3 | 99.9 |
|  | Others (not coded) code start from 07 | 21 | .1 | .1 | 100.0 |
|  | Total | 30553 | 100.0 | 100.0 |  |
|  |  |  |  |  |  |

The physical condition of the playpenThe physical condition of the playpen, table, 1 levels of column headers and 2 levels of row headers, table with 6 columns and 9 rows

|  |  |  |  |  |  |
| --- | --- | --- | --- | --- | --- |
|  | | Frequency | Percent | Valid Percent | Cumulative Percent |
| Valid | Completely out of order/ Broken | 740 | 2.4 | 2.4 | 2.4 |
|  | Cannot be assembeled | 78 | .3 | .3 | 2.7 |
|  | Some parts are broken | 283 | .9 | .9 | 3.6 |
|  | In good / usable condition but not stable | 6637 | 21.7 | 21.7 | 25.3 |
|  | In good / usable condition and stable | 22762 | 74.5 | 74.5 | 99.8 |
|  | Others (not coded) code start from 06 | 53 | .2 | .2 | 100.0 |
|  | Total | 30553 | 100.0 | 100.0 |  |
|  |  |  |  |  |  |

What is the playpen being usedWhat is the playpen being used, table, 1 levels of column headers and 2 levels of row headers, table with 6 columns and 7 rows

|  |  |  |  |  |  |
| --- | --- | --- | --- | --- | --- |
|  | | Frequency | Percent | Valid Percent | Cumulative Percent |
| Valid | For safe keeping of the child | 29704 | 97.2 | 97.2 | 97.2 |
|  | For storing household goods | 565 | 1.8 | 1.8 | 99.1 |
|  | not in use | 277 | .9 | .9 | 100.0 |
|  | Others (not coded) code start from 03 | 7 | .0 | .0 | 100.0 |
|  | Total | 30553 | 100.0 | 100.0 |  |
|  |  |  |  |  |  |

No oneNo one, table, 1 levels of column headers and 2 levels of row headers, table with 6 columns and 5 rows

|  |  |  |  |  |  |
| --- | --- | --- | --- | --- | --- |
|  | | Frequency | Percent | Valid Percent | Cumulative Percent |
| Valid |  | 24471 | 80.1 | 80.1 | 80.1 |
|  | 1 | 6082 | 19.9 | 19.9 | 100.0 |
|  | Total | 30553 | 100.0 | 100.0 |  |
|  |  |  |  |  |  |

Mother/primary caregiverMother/primary caregiver, table, 1 levels of column headers and 2 levels of row headers, table with 6 columns and 5 rows

|  |  |  |  |  |  |
| --- | --- | --- | --- | --- | --- |
|  | | Frequency | Percent | Valid Percent | Cumulative Percent |
| Valid |  | 8844 | 28.9 | 28.9 | 28.9 |
|  | 1 | 21709 | 71.1 | 71.1 | 100.0 |
|  | Total | 30553 | 100.0 | 100.0 |  |
|  |  |  |  |  |  |

Paternal grandmotherPaternal grandmother, table, 1 levels of column headers and 2 levels of row headers, table with 6 columns and 5 rows

|  |  |  |  |  |  |
| --- | --- | --- | --- | --- | --- |
|  | | Frequency | Percent | Valid Percent | Cumulative Percent |
| Valid |  | 29794 | 97.5 | 97.5 | 97.5 |
|  | 1 | 759 | 2.5 | 2.5 | 100.0 |
|  | Total | 30553 | 100.0 | 100.0 |  |
|  |  |  |  |  |  |

Age of grandmotherAge of grandmother, table, 1 levels of column headers and 2 levels of row headers, table with 6 columns and 39 rows

|  |  |  |  |  |  |
| --- | --- | --- | --- | --- | --- |
|  | | Frequency | Percent | Valid Percent | Cumulative Percent |
| Valid |  | 29794 | 97.5 | 97.5 | 97.5 |
|  | 34 | 1 | .0 | .0 | 97.5 |
|  | 35 | 5 | .0 | .0 | 97.5 |
|  | 37 | 1 | .0 | .0 | 97.5 |
|  | 38 | 11 | .0 | .0 | 97.6 |
|  | 40 | 126 | .4 | .4 | 98.0 |
|  | 41 | 3 | .0 | .0 | 98.0 |
|  | 42 | 16 | .1 | .1 | 98.0 |
|  | 43 | 7 | .0 | .0 | 98.1 |
|  | 44 | 1 | .0 | .0 | 98.1 |
|  | 45 | 121 | .4 | .4 | 98.5 |
|  | 46 | 9 | .0 | .0 | 98.5 |
|  | 47 | 3 | .0 | .0 | 98.5 |
|  | 48 | 17 | .1 | .1 | 98.6 |
|  | 49 | 7 | .0 | .0 | 98.6 |
|  | 50 | 213 | .7 | .7 | 99.3 |
|  | 51 | 8 | .0 | .0 | 99.3 |
|  | 52 | 9 | .0 | .0 | 99.3 |
|  | 53 | 4 | .0 | .0 | 99.4 |
|  | 54 | 2 | .0 | .0 | 99.4 |
|  | 55 | 50 | .2 | .2 | 99.5 |
|  | 56 | 12 | .0 | .0 | 99.6 |
|  | 57 | 4 | .0 | .0 | 99.6 |
|  | 58 | 2 | .0 | .0 | 99.6 |
|  | 59 | 1 | .0 | .0 | 99.6 |
|  | 60 | 81 | .3 | .3 | 99.9 |
|  | 61 | 2 | .0 | .0 | 99.9 |
|  | 62 | 4 | .0 | .0 | 99.9 |
|  | 63 | 2 | .0 | .0 | 99.9 |
|  | 65 | 15 | .0 | .0 | 99.9 |
|  | 66 | 1 | .0 | .0 | 99.9 |
|  | 70 | 16 | .1 | .1 | 100.0 |
|  | 75 | 2 | .0 | .0 | 100.0 |
|  | 77 | 1 | .0 | .0 | 100.0 |
|  | 80 | 1 | .0 | .0 | 100.0 |
|  | 90 | 1 | .0 | .0 | 100.0 |
|  | Total | 30553 | 100.0 | 100.0 |  |
|  |  |  |  |  |  |

paternal grandfatherpaternal grandfather, table, 1 levels of column headers and 2 levels of row headers, table with 6 columns and 5 rows

|  |  |  |  |  |  |
| --- | --- | --- | --- | --- | --- |
|  | | Frequency | Percent | Valid Percent | Cumulative Percent |
| Valid |  | 30493 | 99.8 | 99.8 | 99.8 |
|  | 1 | 60 | .2 | .2 | 100.0 |
|  | Total | 30553 | 100.0 | 100.0 |  |
|  |  |  |  |  |  |

Age of paternal grandfatherAge of paternal grandfather, table, 1 levels of column headers and 2 levels of row headers, table with 6 columns and 19 rows

|  |  |  |  |  |  |
| --- | --- | --- | --- | --- | --- |
|  | | Frequency | Percent | Valid Percent | Cumulative Percent |
| Valid |  | 30493 | 99.8 | 99.8 | 99.8 |
|  | 40 | 8 | .0 | .0 | 99.8 |
|  | 45 | 4 | .0 | .0 | 99.8 |
|  | 48 | 1 | .0 | .0 | 99.8 |
|  | 50 | 7 | .0 | .0 | 99.9 |
|  | 52 | 1 | .0 | .0 | 99.9 |
|  | 55 | 6 | .0 | .0 | 99.9 |
|  | 56 | 2 | .0 | .0 | 99.9 |
|  | 59 | 2 | .0 | .0 | 99.9 |
|  | 60 | 17 | .1 | .1 | 100.0 |
|  | 62 | 1 | .0 | .0 | 100.0 |
|  | 65 | 4 | .0 | .0 | 100.0 |
|  | 67 | 1 | .0 | .0 | 100.0 |
|  | 70 | 3 | .0 | .0 | 100.0 |
|  | 76 | 1 | .0 | .0 | 100.0 |
|  | 80 | 2 | .0 | .0 | 100.0 |
|  | Total | 30553 | 100.0 | 100.0 |  |
|  |  |  |  |  |  |

FatherFather, table, 1 levels of column headers and 2 levels of row headers, table with 6 columns and 5 rows

|  |  |  |  |  |  |
| --- | --- | --- | --- | --- | --- |
|  | | Frequency | Percent | Valid Percent | Cumulative Percent |
| Valid |  | 30387 | 99.5 | 99.5 | 99.5 |
|  | 1 | 166 | .5 | .5 | 100.0 |
|  | Total | 30553 | 100.0 | 100.0 |  |
|  |  |  |  |  |  |

Age of fatherAge of father, table, 1 levels of column headers and 2 levels of row headers, table with 6 columns and 25 rows

|  |  |  |  |  |  |
| --- | --- | --- | --- | --- | --- |
|  | | Frequency | Percent | Valid Percent | Cumulative Percent |
| Valid |  | 30387 | 99.5 | 99.5 | 99.5 |
|  | 23 | 1 | .0 | .0 | 99.5 |
|  | 24 | 3 | .0 | .0 | 99.5 |
|  | 25 | 19 | .1 | .1 | 99.5 |
|  | 26 | 4 | .0 | .0 | 99.5 |
|  | 27 | 3 | .0 | .0 | 99.6 |
|  | 28 | 5 | .0 | .0 | 99.6 |
|  | 29 | 2 | .0 | .0 | 99.6 |
|  | 30 | 31 | .1 | .1 | 99.7 |
|  | 32 | 12 | .0 | .0 | 99.7 |
|  | 34 | 3 | .0 | .0 | 99.7 |
|  | 35 | 30 | .1 | .1 | 99.8 |
|  | 36 | 2 | .0 | .0 | 99.8 |
|  | 37 | 2 | .0 | .0 | 99.8 |
|  | 38 | 7 | .0 | .0 | 99.9 |
|  | 39 | 1 | .0 | .0 | 99.9 |
|  | 40 | 32 | .1 | .1 | 100.0 |
|  | 45 | 2 | .0 | .0 | 100.0 |
|  | 47 | 1 | .0 | .0 | 100.0 |
|  | 50 | 1 | .0 | .0 | 100.0 |
|  | 55 | 3 | .0 | .0 | 100.0 |
|  | 60 | 2 | .0 | .0 | 100.0 |
|  | Total | 30553 | 100.0 | 100.0 |  |
|  |  |  |  |  |  |

Maternal grandfatherMaternal grandfather, table, 1 levels of column headers and 2 levels of row headers, table with 6 columns and 5 rows

|  |  |  |  |  |  |
| --- | --- | --- | --- | --- | --- |
|  | | Frequency | Percent | Valid Percent | Cumulative Percent |
| Valid |  | 30541 | 100.0 | 100.0 | 100.0 |
|  | 1 | 12 | .0 | .0 | 100.0 |
|  | Total | 30553 | 100.0 | 100.0 |  |
|  |  |  |  |  |  |

Age of Maternal grandfatherAge of Maternal grandfather, table, 1 levels of column headers and 2 levels of row headers, table with 6 columns and 9 rows

|  |  |  |  |  |  |
| --- | --- | --- | --- | --- | --- |
|  | | Frequency | Percent | Valid Percent | Cumulative Percent |
| Valid |  | 30541 | 100.0 | 100.0 | 100.0 |
|  | 40 | 2 | .0 | .0 | 100.0 |
|  | 42 | 2 | .0 | .0 | 100.0 |
|  | 50 | 2 | .0 | .0 | 100.0 |
|  | 55 | 1 | .0 | .0 | 100.0 |
|  | 60 | 5 | .0 | .0 | 100.0 |
|  | Total | 30553 | 100.0 | 100.0 |  |
|  |  |  |  |  |  |

Maternal grandmotherMaternal grandmother, table, 1 levels of column headers and 2 levels of row headers, table with 6 columns and 5 rows

|  |  |  |  |  |  |
| --- | --- | --- | --- | --- | --- |
|  | | Frequency | Percent | Valid Percent | Cumulative Percent |
| Valid |  | 30408 | 99.5 | 99.5 | 99.5 |
|  | 1 | 145 | .5 | .5 | 100.0 |
|  | Total | 30553 | 100.0 | 100.0 |  |
|  |  |  |  |  |  |

Age of Maternal grandmotherAge of Maternal grandmother, table, 1 levels of column headers and 2 levels of row headers, table with 6 columns and 24 rows

|  |  |  |  |  |  |
| --- | --- | --- | --- | --- | --- |
|  | | Frequency | Percent | Valid Percent | Cumulative Percent |
| Valid |  | 30408 | 99.5 | 99.5 | 99.5 |
|  | 35 | 2 | .0 | .0 | 99.5 |
|  | 37 | 1 | .0 | .0 | 99.5 |
|  | 38 | 2 | .0 | .0 | 99.5 |
|  | 40 | 28 | .1 | .1 | 99.6 |
|  | 42 | 2 | .0 | .0 | 99.6 |
|  | 45 | 19 | .1 | .1 | 99.7 |
|  | 46 | 1 | .0 | .0 | 99.7 |
|  | 47 | 1 | .0 | .0 | 99.7 |
|  | 48 | 5 | .0 | .0 | 99.7 |
|  | 49 | 3 | .0 | .0 | 99.7 |
|  | 50 | 44 | .1 | .1 | 99.9 |
|  | 55 | 9 | .0 | .0 | 99.9 |
|  | 56 | 4 | .0 | .0 | 99.9 |
|  | 57 | 1 | .0 | .0 | 99.9 |
|  | 58 | 1 | .0 | .0 | 99.9 |
|  | 60 | 14 | .0 | .0 | 100.0 |
|  | 63 | 1 | .0 | .0 | 100.0 |
|  | 65 | 4 | .0 | .0 | 100.0 |
|  | 66 | 1 | .0 | .0 | 100.0 |
|  | 70 | 2 | .0 | .0 | 100.0 |
|  | Total | 30553 | 100.0 | 100.0 |  |
|  |  |  |  |  |  |

SiblingSibling, table, 1 levels of column headers and 2 levels of row headers, table with 6 columns and 5 rows

|  |  |  |  |  |  |
| --- | --- | --- | --- | --- | --- |
|  | | Frequency | Percent | Valid Percent | Cumulative Percent |
| Valid |  | 30292 | 99.1 | 99.1 | 99.1 |
|  | 1 | 261 | .9 | .9 | 100.0 |
|  | Total | 30553 | 100.0 | 100.0 |  |
|  |  |  |  |  |  |

Age of siblingAge of sibling, table, 1 levels of column headers and 2 levels of row headers, table with 6 columns and 22 rows

|  |  |  |  |  |  |
| --- | --- | --- | --- | --- | --- |
|  | | Frequency | Percent | Valid Percent | Cumulative Percent |
| Valid | 3 | 2 | .0 | .8 | .8 |
|  | 4 | 1 | .0 | .4 | 1.1 |
|  | 5 | 21 | .1 | 8.0 | 9.2 |
|  | 6 | 21 | .1 | 8.0 | 17.2 |
|  | 7 | 24 | .1 | 9.2 | 26.4 |
|  | 8 | 36 | .1 | 13.8 | 40.2 |
|  | 9 | 19 | .1 | 7.3 | 47.5 |
|  | 10 | 42 | .1 | 16.1 | 63.6 |
|  | 11 | 13 | .0 | 5.0 | 68.6 |
|  | 12 | 24 | .1 | 9.2 | 77.8 |
|  | 13 | 12 | .0 | 4.6 | 82.4 |
|  | 14 | 9 | .0 | 3.4 | 85.8 |
|  | 15 | 20 | .1 | 7.7 | 93.5 |
|  | 16 | 9 | .0 | 3.4 | 96.9 |
|  | 17 | 1 | .0 | .4 | 97.3 |
|  | 18 | 6 | .0 | 2.3 | 99.6 |
|  | 20 | 1 | .0 | .4 | 100.0 |
|  | Total | 261 | .9 | 100.0 |  |
| Missing | System | 30292 | 99.1 |  |  |
| Total | | 30553 | 100.0 |  |  |
|  |  |  |  |  |  |

CousinCousin, table, 1 levels of column headers and 2 levels of row headers, table with 6 columns and 5 rows

|  |  |  |  |  |  |
| --- | --- | --- | --- | --- | --- |
|  | | Frequency | Percent | Valid Percent | Cumulative Percent |
| Valid |  | 30531 | 99.9 | 99.9 | 99.9 |
|  | 1 | 22 | .1 | .1 | 100.0 |
|  | Total | 30553 | 100.0 | 100.0 |  |
|  |  |  |  |  |  |

Age of CousinAge of Cousin, table, 1 levels of column headers and 2 levels of row headers, table with 6 columns and 19 rows

|  |  |  |  |  |  |
| --- | --- | --- | --- | --- | --- |
|  | | Frequency | Percent | Valid Percent | Cumulative Percent |
| Valid | 5 | 1 | .0 | 4.5 | 4.5 |
|  | 6 | 1 | .0 | 4.5 | 9.1 |
|  | 8 | 3 | .0 | 13.6 | 22.7 |
|  | 9 | 1 | .0 | 4.5 | 27.3 |
|  | 10 | 1 | .0 | 4.5 | 31.8 |
|  | 12 | 2 | .0 | 9.1 | 40.9 |
|  | 13 | 1 | .0 | 4.5 | 45.5 |
|  | 14 | 1 | .0 | 4.5 | 50.0 |
|  | 15 | 3 | .0 | 13.6 | 63.6 |
|  | 16 | 2 | .0 | 9.1 | 72.7 |
|  | 17 | 1 | .0 | 4.5 | 77.3 |
|  | 20 | 3 | .0 | 13.6 | 90.9 |
|  | 21 | 1 | .0 | 4.5 | 95.5 |
|  | 30 | 1 | .0 | 4.5 | 100.0 |
|  | Total | 22 | .1 | 100.0 |  |
| Missing | System | 30531 | 99.9 |  |  |
| Total | | 30553 | 100.0 |  |  |
|  |  |  |  |  |  |

Aunt/uncleAunt/uncle, table, 1 levels of column headers and 2 levels of row headers, table with 6 columns and 5 rows

|  |  |  |  |  |  |
| --- | --- | --- | --- | --- | --- |
|  | | Frequency | Percent | Valid Percent | Cumulative Percent |
| Valid | 1 | 78 | .3 | 100.0 | 100.0 |
| Missing | System | 30475 | 99.7 |  |  |
| Total | | 30553 | 100.0 |  |  |
|  |  |  |  |  |  |

Age of Aunt/uncleAge of Aunt/uncle, table, 1 levels of column headers and 2 levels of row headers, table with 6 columns and 28 rows

|  |  |  |  |  |  |
| --- | --- | --- | --- | --- | --- |
|  | | Frequency | Percent | Valid Percent | Cumulative Percent |
| Valid | 9 | 1 | .0 | 1.3 | 1.3 |
|  | 10 | 3 | .0 | 3.8 | 5.1 |
|  | 11 | 1 | .0 | 1.3 | 6.4 |
|  | 12 | 1 | .0 | 1.3 | 7.7 |
|  | 13 | 2 | .0 | 2.6 | 10.3 |
|  | 14 | 4 | .0 | 5.1 | 15.4 |
|  | 15 | 2 | .0 | 2.6 | 17.9 |
|  | 16 | 2 | .0 | 2.6 | 20.5 |
|  | 17 | 3 | .0 | 3.8 | 24.4 |
|  | 18 | 1 | .0 | 1.3 | 25.6 |
|  | 20 | 11 | .0 | 14.1 | 39.7 |
|  | 21 | 1 | .0 | 1.3 | 41.0 |
|  | 22 | 4 | .0 | 5.1 | 46.2 |
|  | 24 | 1 | .0 | 1.3 | 47.4 |
|  | 25 | 14 | .0 | 17.9 | 65.4 |
|  | 26 | 1 | .0 | 1.3 | 66.7 |
|  | 28 | 1 | .0 | 1.3 | 67.9 |
|  | 30 | 9 | .0 | 11.5 | 79.5 |
|  | 35 | 5 | .0 | 6.4 | 85.9 |
|  | 38 | 1 | .0 | 1.3 | 87.2 |
|  | 40 | 6 | .0 | 7.7 | 94.9 |
|  | 45 | 2 | .0 | 2.6 | 97.4 |
|  | 50 | 2 | .0 | 2.6 | 100.0 |
|  | Total | 78 | .3 | 100.0 |  |
| Missing | System | 30475 | 99.7 |  |  |
| Total | | 30553 | 100.0 |  |  |
|  |  |  |  |  |  |

NeighbourNeighbour, table, 1 levels of column headers and 2 levels of row headers, table with 6 columns and 5 rows

|  |  |  |  |  |  |
| --- | --- | --- | --- | --- | --- |
|  | | Frequency | Percent | Valid Percent | Cumulative Percent |
| Valid | 1 | 309 | 1.0 | 100.0 | 100.0 |
| Missing | System | 30244 | 99.0 |  |  |
| Total | | 30553 | 100.0 |  |  |
|  |  |  |  |  |  |

Age of NeighbourAge of Neighbour, table, 1 levels of column headers and 2 levels of row headers, table with 6 columns and 34 rows

|  |  |  |  |  |  |
| --- | --- | --- | --- | --- | --- |
|  | | Frequency | Percent | Valid Percent | Cumulative Percent |
| Valid | 8 | 6 | .0 | 1.9 | 1.9 |
|  | 10 | 16 | .1 | 5.2 | 7.1 |
|  | 12 | 8 | .0 | 2.6 | 9.7 |
|  | 13 | 2 | .0 | .6 | 10.4 |
|  | 15 | 5 | .0 | 1.6 | 12.0 |
|  | 16 | 1 | .0 | .3 | 12.3 |
|  | 17 | 13 | .0 | 4.2 | 16.5 |
|  | 18 | 7 | .0 | 2.3 | 18.8 |
|  | 19 | 1 | .0 | .3 | 19.1 |
|  | 20 | 13 | .0 | 4.2 | 23.3 |
|  | 21 | 1 | .0 | .3 | 23.6 |
|  | 22 | 14 | .0 | 4.5 | 28.2 |
|  | 23 | 2 | .0 | .6 | 28.8 |
|  | 24 | 6 | .0 | 1.9 | 30.7 |
|  | 25 | 42 | .1 | 13.6 | 44.3 |
|  | 26 | 18 | .1 | 5.8 | 50.2 |
|  | 27 | 8 | .0 | 2.6 | 52.8 |
|  | 28 | 3 | .0 | 1.0 | 53.7 |
|  | 29 | 14 | .0 | 4.5 | 58.3 |
|  | 30 | 58 | .2 | 18.8 | 77.0 |
|  | 32 | 11 | .0 | 3.6 | 80.6 |
|  | 34 | 13 | .0 | 4.2 | 84.8 |
|  | 35 | 18 | .1 | 5.8 | 90.6 |
|  | 36 | 5 | .0 | 1.6 | 92.2 |
|  | 39 | 2 | .0 | .6 | 92.9 |
|  | 40 | 18 | .1 | 5.8 | 98.7 |
|  | 48 | 1 | .0 | .3 | 99.0 |
|  | 51 | 1 | .0 | .3 | 99.4 |
|  | 55 | 2 | .0 | .6 | 100.0 |
|  | Total | 309 | 1.0 | 100.0 |  |
| Missing | System | 30244 | 99.0 |  |  |
| Total | | 30553 | 100.0 |  |  |
|  |  |  |  |  |  |

OthersOthers, table, 1 levels of column headers and 2 levels of row headers, table with 6 columns and 5 rows

|  |  |  |  |  |  |
| --- | --- | --- | --- | --- | --- |
|  | | Frequency | Percent | Valid Percent | Cumulative Percent |
| Valid | 1 | 951 | 3.1 | 100.0 | 100.0 |
| Missing | System | 29602 | 96.9 |  |  |
| Total | | 30553 | 100.0 |  |  |
|  |  |  |  |  |  |

Other specifyOther specify, table, 1 levels of column headers and 2 levels of row headers, table with 4 columns and 3 rows

|  |  |  |  |
| --- | --- | --- | --- |
|  | | Frequency | Percent |
| Missing | System | 30553 | 100.0 |
|  |  |  |  |

Age of other personAge of other person, table, 1 levels of column headers and 2 levels of row headers, table with 6 columns and 39 rows

|  |  |  |  |  |  |
| --- | --- | --- | --- | --- | --- |
|  | | Frequency | Percent | Valid Percent | Cumulative Percent |
| Valid | 12 | 1 | .0 | .1 | .1 |
|  | 13 | 2 | .0 | .2 | .3 |
|  | 15 | 3 | .0 | .3 | .6 |
|  | 16 | 12 | .0 | 1.3 | 1.9 |
|  | 17 | 9 | .0 | .9 | 2.8 |
|  | 18 | 23 | .1 | 2.4 | 5.3 |
|  | 19 | 38 | .1 | 4.0 | 9.3 |
|  | 20 | 92 | .3 | 9.7 | 18.9 |
|  | 21 | 16 | .1 | 1.7 | 20.6 |
|  | 22 | 56 | .2 | 5.9 | 26.5 |
|  | 23 | 58 | .2 | 6.1 | 32.6 |
|  | 24 | 23 | .1 | 2.4 | 35.0 |
|  | 25 | 118 | .4 | 12.4 | 47.4 |
|  | 26 | 23 | .1 | 2.4 | 49.8 |
|  | 27 | 72 | .2 | 7.6 | 57.4 |
|  | 28 | 18 | .1 | 1.9 | 59.3 |
|  | 29 | 6 | .0 | .6 | 59.9 |
|  | 30 | 100 | .3 | 10.5 | 70.5 |
|  | 31 | 1 | .0 | .1 | 70.6 |
|  | 32 | 27 | .1 | 2.8 | 73.4 |
|  | 33 | 4 | .0 | .4 | 73.8 |
|  | 34 | 24 | .1 | 2.5 | 76.3 |
|  | 35 | 75 | .2 | 7.9 | 84.2 |
|  | 36 | 18 | .1 | 1.9 | 86.1 |
|  | 37 | 1 | .0 | .1 | 86.2 |
|  | 40 | 48 | .2 | 5.0 | 91.3 |
|  | 43 | 2 | .0 | .2 | 91.5 |
|  | 45 | 27 | .1 | 2.8 | 94.3 |
|  | 50 | 44 | .1 | 4.6 | 98.9 |
|  | 55 | 1 | .0 | .1 | 99.1 |
|  | 57 | 1 | .0 | .1 | 99.2 |
|  | 60 | 4 | .0 | .4 | 99.6 |
|  | 80 | 2 | .0 | .2 | 99.8 |
|  | 90 | 2 | .0 | .2 | 100.0 |
|  | Total | 951 | 3.1 | 100.0 |  |
| Missing | System | 29602 | 96.9 |  |  |
| Total | | 30553 | 100.0 |  |  |
|  |  |  |  |  |  |

Activites of mother/primary caregiver during that timeActivites of mother/primary caregiver during that time, table, 1 levels of column headers and 2 levels of row headers, table with 6 columns and 9 rows

|  |  |  |  |  |  |
| --- | --- | --- | --- | --- | --- |
|  | | Frequency | Percent | Valid Percent | Cumulative Percent |
| Valid | Not at home | 789 | 2.6 | 8.6 | 8.6 |
|  | Doing household chores | 7364 | 24.1 | 80.1 | 88.7 |
|  | Taking rest | 888 | 2.9 | 9.7 | 98.3 |
|  | others (not coded) code start from 04 | 152 | .5 | 1.7 | 100.0 |
|  | Total | 9193 | 30.1 | 100.0 |  |
| Missing | System | 21360 | 69.9 |  |  |
| Total | | 30553 | 100.0 |  |  |
|  |  |  |  |  |  |

Whether the mother used the plapen since the intervention worker's last visitWhether the mother used the plapen since the intervention worker's last visit, table, 1 levels of column headers and 2 levels of row headers, table with 6 columns and 5 rows

|  |  |  |  |  |  |
| --- | --- | --- | --- | --- | --- |
|  | | Frequency | Percent | Valid Percent | Cumulative Percent |
| Valid | Yes | 29209 | 95.6 | 95.6 | 95.6 |
|  | No | 1344 | 4.4 | 4.4 | 100.0 |
|  | Total | 30553 | 100.0 | 100.0 |  |
|  |  |  |  |  |  |

Number of days that the plapen used in last weekNumber of days that the plapen used in last week, table, 1 levels of column headers and 2 levels of row headers, table with 6 columns and 12 rows

|  |  |  |  |  |  |
| --- | --- | --- | --- | --- | --- |
|  | | Frequency | Percent | Valid Percent | Cumulative Percent |
| Valid | 1 | 7894 | 25.8 | 27.0 | 27.0 |
|  | 2 | 2174 | 7.1 | 7.4 | 34.5 |
|  | 3 | 2108 | 6.9 | 7.2 | 41.7 |
|  | 4 | 2772 | 9.1 | 9.5 | 51.2 |
|  | 5 | 3848 | 12.6 | 13.2 | 64.4 |
|  | 6 | 4145 | 13.6 | 14.2 | 78.5 |
|  | 7 | 6268 | 20.5 | 21.5 | 100.0 |
|  | Total | 29209 | 95.6 | 100.0 |  |
| Missing | System | 1344 | 4.4 |  |  |
| Total | | 30553 | 100.0 |  |  |
|  |  |  |  |  |  |

Average number of times the baby kept in the palpenAverage number of times the baby kept in the palpen, table, 1 levels of column headers and 2 levels of row headers, table with 6 columns and 16 rows

|  |  |  |  |  |  |
| --- | --- | --- | --- | --- | --- |
|  | | Frequency | Percent | Valid Percent | Cumulative Percent |
| Valid | 1 | 1111 | 3.6 | 3.8 | 3.8 |
|  | 2 | 4859 | 15.9 | 16.6 | 20.4 |
|  | 3 | 7661 | 25.1 | 26.2 | 46.7 |
|  | 4 | 5908 | 19.3 | 20.2 | 66.9 |
|  | 5 | 4180 | 13.7 | 14.3 | 81.2 |
|  | 6 | 2153 | 7.0 | 7.4 | 88.6 |
|  | 7 | 1032 | 3.4 | 3.5 | 92.1 |
|  | 8 | 1180 | 3.9 | 4.0 | 96.1 |
|  | 9 | 394 | 1.3 | 1.3 | 97.5 |
|  | 10 | 589 | 1.9 | 2.0 | 99.5 |
|  | 11 | 142 | .5 | .5 | 100.0 |
|  | Total | 29209 | 95.6 | 100.0 |  |
| Missing | System | 1344 | 4.4 |  |  |
| Total | | 30553 | 100.0 |  |  |
|  |  |  |  |  |  |

Wash dishes/clothWash dishes/cloth, table, 1 levels of column headers and 2 levels of row headers, table with 6 columns and 6 rows

|  |  |  |  |  |  |
| --- | --- | --- | --- | --- | --- |
|  | | Frequency | Percent | Valid Percent | Cumulative Percent |
| Valid |  | 1344 | 4.4 | 4.4 | 4.4 |
|  | 1 | 19744 | 64.6 | 64.6 | 69.0 |
|  | 2 | 9465 | 31.0 | 31.0 | 100.0 |
|  | Total | 30553 | 100.0 | 100.0 |  |
|  |  |  |  |  |  |

Taking care of the poultry/domestic animalsTaking care of the poultry/domestic animals, table, 1 levels of column headers and 2 levels of row headers, table with 6 columns and 6 rows

|  |  |  |  |  |  |
| --- | --- | --- | --- | --- | --- |
|  | | Frequency | Percent | Valid Percent | Cumulative Percent |
| Valid |  | 1344 | 4.4 | 4.4 | 4.4 |
|  | 1 | 12239 | 40.1 | 40.1 | 44.5 |
|  | 2 | 16970 | 55.5 | 55.5 | 100.0 |
|  | Total | 30553 | 100.0 | 100.0 |  |
|  |  |  |  |  |  |

Collect fuel/water/irrigation workCollect fuel/water/irrigation work, table, 1 levels of column headers and 2 levels of row headers, table with 6 columns and 6 rows

|  |  |  |  |  |  |
| --- | --- | --- | --- | --- | --- |
|  | | Frequency | Percent | Valid Percent | Cumulative Percent |
| Valid |  | 1344 | 4.4 | 4.4 | 4.4 |
|  | 1 | 5808 | 19.0 | 19.0 | 23.4 |
|  | 2 | 23401 | 76.6 | 76.6 | 100.0 |
|  | Total | 30553 | 100.0 | 100.0 |  |
|  |  |  |  |  |  |

Child Care-Bathing,teaching,feeding,taking to schoolChild Care-Bathing,teaching,feeding,taking to school, table, 1 levels of column headers and 2 levels of row headers, table with 6 columns and 6 rows

|  |  |  |  |  |  |
| --- | --- | --- | --- | --- | --- |
|  | | Frequency | Percent | Valid Percent | Cumulative Percent |
| Valid |  | 1344 | 4.4 | 4.4 | 4.4 |
|  | 1 | 4816 | 15.8 | 15.8 | 20.2 |
|  | 2 | 24393 | 79.8 | 79.8 | 100.0 |
|  | Total | 30553 | 100.0 | 100.0 |  |
|  |  |  |  |  |  |

Care of other HH membersCare of other HH members, table, 1 levels of column headers and 2 levels of row headers, table with 6 columns and 6 rows

|  |  |  |  |  |  |
| --- | --- | --- | --- | --- | --- |
|  | | Frequency | Percent | Valid Percent | Cumulative Percent |
| Valid |  | 1344 | 4.4 | 4.4 | 4.4 |
|  | 1 | 5486 | 18.0 | 18.0 | 22.4 |
|  | 2 | 23723 | 77.6 | 77.6 | 100.0 |
|  | Total | 30553 | 100.0 | 100.0 |  |
|  |  |  |  |  |  |

Other HH work e.g.Cleaning the HHOther HH work e.g.Cleaning the HH, table, 1 levels of column headers and 2 levels of row headers, table with 6 columns and 6 rows

|  |  |  |  |  |  |
| --- | --- | --- | --- | --- | --- |
|  | | Frequency | Percent | Valid Percent | Cumulative Percent |
| Valid |  | 1344 | 4.4 | 4.4 | 4.4 |
|  | 1 | 6606 | 21.6 | 21.6 | 26.0 |
|  | 2 | 22603 | 74.0 | 74.0 | 100.0 |
|  | Total | 30553 | 100.0 | 100.0 |  |
|  |  |  |  |  |  |

Working in others houseWorking in others house, table, 1 levels of column headers and 2 levels of row headers, table with 6 columns and 6 rows

|  |  |  |  |  |  |
| --- | --- | --- | --- | --- | --- |
|  | | Frequency | Percent | Valid Percent | Cumulative Percent |
| Valid |  | 1344 | 4.4 | 4.4 | 4.4 |
|  | 1 | 1184 | 3.9 | 3.9 | 8.3 |
|  | 2 | 28025 | 91.7 | 91.7 | 100.0 |
|  | Total | 30553 | 100.0 | 100.0 |  |
|  |  |  |  |  |  |

Leisure activities-chatting,sleeping,taking rest etc.Leisure activities-chatting,sleeping,taking rest etc., table, 1 levels of column headers and 2 levels of row headers, table with 6 columns and 6 rows

|  |  |  |  |  |  |
| --- | --- | --- | --- | --- | --- |
|  | | Frequency | Percent | Valid Percent | Cumulative Percent |
| Valid |  | 1344 | 4.4 | 4.4 | 4.4 |
|  | 1 | 2314 | 7.6 | 7.6 | 12.0 |
|  | 2 | 26895 | 88.0 | 88.0 | 100.0 |
|  | Total | 30553 | 100.0 | 100.0 |  |
|  |  |  |  |  |  |

CookingCooking, table, 1 levels of column headers and 2 levels of row headers, table with 6 columns and 6 rows

|  |  |  |  |  |  |
| --- | --- | --- | --- | --- | --- |
|  | | Frequency | Percent | Valid Percent | Cumulative Percent |
| Valid |  | 1344 | 4.4 | 4.4 | 4.4 |
|  | 1 | 17854 | 58.4 | 58.4 | 62.8 |
|  | 2 | 11355 | 37.2 | 37.2 | 100.0 |
|  | Total | 30553 | 100.0 | 100.0 |  |
|  |  |  |  |  |  |

OthersOthers, table, 1 levels of column headers and 2 levels of row headers, table with 6 columns and 6 rows

|  |  |  |  |  |  |
| --- | --- | --- | --- | --- | --- |
|  | | Frequency | Percent | Valid Percent | Cumulative Percent |
| Valid |  | 1344 | 4.4 | 4.4 | 4.4 |
|  | 1 | 565 | 1.8 | 1.8 | 6.2 |
|  | 2 | 28644 | 93.8 | 93.8 | 100.0 |
|  | Total | 30553 | 100.0 | 100.0 |  |
|  |  |  |  |  |  |

Other(Specify)Other(Specify), table, 1 levels of column headers and 2 levels of row headers, table with 6 columns and 3 rows

|  |  |  |  |  |  |
| --- | --- | --- | --- | --- | --- |
|  | | Frequency | Percent | Valid Percent | Cumulative Percent |
| Valid |  | 30553 | 100.0 | 100.0 | 100.0 |
|  |  |  |  |  |  |

How long the child kept in the playpen during last use of the playpenHow long the child kept in the playpen during last use of the playpen, table, 1 levels of column headers and 2 levels of row headers, table with 6 columns and 120 rows

|  |  |  |  |  |  |
| --- | --- | --- | --- | --- | --- |
|  | | Frequency | Percent | Valid Percent | Cumulative Percent |
| Valid | 10 | 4539 | 14.9 | 15.5 | 15.5 |
|  | 11 | 203 | .7 | .7 | 16.2 |
|  | 12 | 573 | 1.9 | 2.0 | 18.2 |
|  | 13 | 149 | .5 | .5 | 18.7 |
|  | 14 | 105 | .3 | .4 | 19.1 |
|  | 15 | 2788 | 9.1 | 9.5 | 28.6 |
|  | 16 | 143 | .5 | .5 | 29.1 |
|  | 17 | 85 | .3 | .3 | 29.4 |
|  | 18 | 142 | .5 | .5 | 29.9 |
|  | 19 | 61 | .2 | .2 | 30.1 |
|  | 20 | 6103 | 20.0 | 20.9 | 51.0 |
|  | 21 | 71 | .2 | .2 | 51.2 |
|  | 22 | 107 | .4 | .4 | 51.6 |
|  | 23 | 47 | .2 | .2 | 51.8 |
|  | 24 | 62 | .2 | .2 | 52.0 |
|  | 25 | 1278 | 4.2 | 4.4 | 56.3 |
|  | 26 | 58 | .2 | .2 | 56.5 |
|  | 27 | 22 | .1 | .1 | 56.6 |
|  | 28 | 31 | .1 | .1 | 56.7 |
|  | 29 | 15 | .0 | .1 | 56.8 |
|  | 30 | 4840 | 15.8 | 16.6 | 73.3 |
|  | 31 | 16 | .1 | .1 | 73.4 |
|  | 32 | 79 | .3 | .3 | 73.7 |
|  | 33 | 24 | .1 | .1 | 73.7 |
|  | 34 | 20 | .1 | .1 | 73.8 |
|  | 35 | 426 | 1.4 | 1.5 | 75.3 |
|  | 36 | 29 | .1 | .1 | 75.4 |
|  | 37 | 9 | .0 | .0 | 75.4 |
|  | 38 | 5 | .0 | .0 | 75.4 |
|  | 39 | 2 | .0 | .0 | 75.4 |
|  | 40 | 1572 | 5.1 | 5.4 | 80.8 |
|  | 41 | 7 | .0 | .0 | 80.8 |
|  | 42 | 16 | .1 | .1 | 80.9 |
|  | 43 | 1 | .0 | .0 | 80.9 |
|  | 44 | 8 | .0 | .0 | 80.9 |
|  | 45 | 1459 | 4.8 | 5.0 | 85.9 |
|  | 46 | 6 | .0 | .0 | 85.9 |
|  | 47 | 5 | .0 | .0 | 86.0 |
|  | 48 | 4 | .0 | .0 | 86.0 |
|  | 49 | 3 | .0 | .0 | 86.0 |
|  | 50 | 636 | 2.1 | 2.2 | 88.2 |
|  | 51 | 1 | .0 | .0 | 88.2 |
|  | 52 | 11 | .0 | .0 | 88.2 |
|  | 54 | 3 | .0 | .0 | 88.2 |
|  | 55 | 243 | .8 | .8 | 89.0 |
|  | 56 | 6 | .0 | .0 | 89.1 |
|  | 57 | 1 | .0 | .0 | 89.1 |
|  | 58 | 18 | .1 | .1 | 89.1 |
|  | 59 | 2 | .0 | .0 | 89.1 |
|  | 60 | 1186 | 3.9 | 4.1 | 93.2 |
|  | 62 | 22 | .1 | .1 | 93.3 |
|  | 63 | 1 | .0 | .0 | 93.3 |
|  | 65 | 12 | .0 | .0 | 93.3 |
|  | 66 | 2 | .0 | .0 | 93.3 |
|  | 67 | 2 | .0 | .0 | 93.3 |
|  | 68 | 1 | .0 | .0 | 93.3 |
|  | 69 | 2 | .0 | .0 | 93.3 |
|  | 70 | 202 | .7 | .7 | 94.0 |
|  | 72 | 2 | .0 | .0 | 94.0 |
|  | 73 | 1 | .0 | .0 | 94.0 |
|  | 75 | 26 | .1 | .1 | 94.1 |
|  | 76 | 1 | .0 | .0 | 94.1 |
|  | 78 | 1 | .0 | .0 | 94.1 |
|  | 80 | 174 | .6 | .6 | 94.7 |
|  | 82 | 3 | .0 | .0 | 94.7 |
|  | 85 | 3 | .0 | .0 | 94.7 |
|  | 90 | 589 | 1.9 | 2.0 | 96.8 |
|  | 93 | 1 | .0 | .0 | 96.8 |
|  | 95 | 4 | .0 | .0 | 96.8 |
|  | 100 | 55 | .2 | .2 | 97.0 |
|  | 101 | 6 | .0 | .0 | 97.0 |
|  | 102 | 5 | .0 | .0 | 97.0 |
|  | 103 | 1 | .0 | .0 | 97.0 |
|  | 105 | 18 | .1 | .1 | 97.1 |
|  | 106 | 1 | .0 | .0 | 97.1 |
|  | 110 | 53 | .2 | .2 | 97.3 |
|  | 111 | 1 | .0 | .0 | 97.3 |
|  | 112 | 3 | .0 | .0 | 97.3 |
|  | 113 | 6 | .0 | .0 | 97.3 |
|  | 115 | 14 | .0 | .0 | 97.3 |
|  | 119 | 1 | .0 | .0 | 97.3 |
|  | 120 | 361 | 1.2 | 1.2 | 98.6 |
|  | 121 | 1 | .0 | .0 | 98.6 |
|  | 122 | 1 | .0 | .0 | 98.6 |
|  | 124 | 1 | .0 | .0 | 98.6 |
|  | 125 | 4 | .0 | .0 | 98.6 |
|  | 128 | 1 | .0 | .0 | 98.6 |
|  | 130 | 125 | .4 | .4 | 99.0 |
|  | 131 | 3 | .0 | .0 | 99.0 |
|  | 135 | 3 | .0 | .0 | 99.1 |
|  | 140 | 23 | .1 | .1 | 99.1 |
|  | 145 | 8 | .0 | .0 | 99.2 |
|  | 150 | 24 | .1 | .1 | 99.2 |
|  | 160 | 18 | .1 | .1 | 99.3 |
|  | 162 | 1 | .0 | .0 | 99.3 |
|  | 170 | 5 | .0 | .0 | 99.3 |
|  | 175 | 2 | .0 | .0 | 99.3 |
|  | 180 | 84 | .3 | .3 | 99.6 |
|  | 190 | 2 | .0 | .0 | 99.6 |
|  | 195 | 2 | .0 | .0 | 99.6 |
|  | 200 | 22 | .1 | .1 | 99.7 |
|  | 201 | 2 | .0 | .0 | 99.7 |
|  | 202 | 7 | .0 | .0 | 99.7 |
|  | 203 | 2 | .0 | .0 | 99.7 |
|  | 205 | 1 | .0 | .0 | 99.8 |
|  | 210 | 20 | .1 | .1 | 99.8 |
|  | 215 | 4 | .0 | .0 | 99.8 |
|  | 220 | 14 | .0 | .0 | 99.9 |
|  | 230 | 13 | .0 | .0 | 99.9 |
|  | 240 | 11 | .0 | .0 | 100.0 |
|  | 250 | 2 | .0 | .0 | 100.0 |
|  | 252 | 2 | .0 | .0 | 100.0 |
|  | 260 | 3 | .0 | .0 | 100.0 |
|  | 270 | 1 | .0 | .0 | 100.0 |
|  | 280 | 3 | .0 | .0 | 100.0 |
|  | Total | 29209 | 95.6 | 100.0 |  |
| Missing | System | 1344 | 4.4 |  |  |
| Total | | 30553 | 100.0 |  |  |
|  |  |  |  |  |  |

Whether the child got any injuries(fell out.cut,bruise)while the playpen being uWhether the child got any injuries(fell out.cut,bruise)while the playpen being u, table, 1 levels of column headers and 2 levels of row headers, table with 6 columns and 7 rows

|  |  |  |  |  |  |
| --- | --- | --- | --- | --- | --- |
|  | | Frequency | Percent | Valid Percent | Cumulative Percent |
| Valid | Yes | 477 | 1.6 | 1.6 | 1.6 |
|  | No | 28732 | 94.0 | 98.4 | 100.0 |
|  | Total | 29209 | 95.6 | 100.0 |  |
| Missing | System | 1344 | 4.4 |  |  |
| Total | | 30553 | 100.0 |  |  |
|  |  |  |  |  |  |

Describe in detail how the child got injuredDescribe in detail how the child got injured, table, 1 levels of column headers and 2 levels of row headers, table with 6 columns and 3 rows

|  |  |  |  |  |  |
| --- | --- | --- | --- | --- | --- |
|  | | Frequency | Percent | Valid Percent | Cumulative Percent |
| Valid |  | 30553 | 100.0 | 100.0 | 100.0 |
|  |  |  |  |  |  |

Any difficulties faces during using the playpenAny difficulties faces during using the playpen, table, 1 levels of column headers and 2 levels of row headers, table with 6 columns and 8 rows

|  |  |  |  |  |  |
| --- | --- | --- | --- | --- | --- |
|  | | Frequency | Percent | Valid Percent | Cumulative Percent |
| Valid |  | 1506 | 4.9 | 4.9 | 4.9 |
|  | None | 28456 | 93.1 | 93.1 | 98.1 |
|  | Difficult to set up | 159 | .5 | .5 | 98.6 |
|  | Don't have enough space | 373 | 1.2 | 1.2 | 99.8 |
|  | Others (Specify) | 59 | .2 | .2 | 100.0 |
|  | Total | 30553 | 100.0 | 100.0 |  |
|  |  |  |  |  |  |

InconvenientInconvenient, table, 1 levels of column headers and 2 levels of row headers, table with 6 columns and 5 rows

|  |  |  |  |  |  |
| --- | --- | --- | --- | --- | --- |
|  | | Frequency | Percent | Valid Percent | Cumulative Percent |
| Valid | 1 | 36 | .1 | .1 | .1 |
|  | 2 | 30517 | 99.9 | 99.9 | 100.0 |
|  | Total | 30553 | 100.0 | 100.0 |  |
|  |  |  |  |  |  |

Child does not want to stay in the playpenChild does not want to stay in the playpen, table, 1 levels of column headers and 2 levels of row headers, table with 6 columns and 5 rows

|  |  |  |  |  |  |
| --- | --- | --- | --- | --- | --- |
|  | | Frequency | Percent | Valid Percent | Cumulative Percent |
| Valid | 1 | 456 | 1.5 | 1.5 | 1.5 |
|  | 2 | 30097 | 98.5 | 98.5 | 100.0 |
|  | Total | 30553 | 100.0 | 100.0 |  |
|  |  |  |  |  |  |

Child goes to crecheChild goes to creche, table, 1 levels of column headers and 2 levels of row headers, table with 6 columns and 5 rows

|  |  |  |  |  |  |
| --- | --- | --- | --- | --- | --- |
|  | | Frequency | Percent | Valid Percent | Cumulative Percent |
| Valid | 1 | 703 | 2.3 | 2.3 | 2.3 |
|  | 2 | 29850 | 97.7 | 97.7 | 100.0 |
|  | Total | 30553 | 100.0 | 100.0 |  |
|  |  |  |  |  |  |

Have a caregiver to look after the childHave a caregiver to look after the child, table, 1 levels of column headers and 2 levels of row headers, table with 6 columns and 5 rows

|  |  |  |  |  |  |
| --- | --- | --- | --- | --- | --- |
|  | | Frequency | Percent | Valid Percent | Cumulative Percent |
| Valid | 1 | 33 | .1 | .1 | .1 |
|  | 2 | 30520 | 99.9 | 99.9 | 100.0 |
|  | Total | 30553 | 100.0 | 100.0 |  |
|  |  |  |  |  |  |

Don't think it is necessaryDon't think it is necessary, table, 1 levels of column headers and 2 levels of row headers, table with 6 columns and 5 rows

|  |  |  |  |  |  |
| --- | --- | --- | --- | --- | --- |
|  | | Frequency | Percent | Valid Percent | Cumulative Percent |
| Valid | 1 | 56 | .2 | .2 | .2 |
|  | 2 | 30497 | 99.8 | 99.8 | 100.0 |
|  | Total | 30553 | 100.0 | 100.0 |  |
|  |  |  |  |  |  |

Have other methods of supervision(specify)Have other methods of supervision(specify), table, 1 levels of column headers and 2 levels of row headers, table with 6 columns and 5 rows

|  |  |  |  |  |  |
| --- | --- | --- | --- | --- | --- |
|  | | Frequency | Percent | Valid Percent | Cumulative Percent |
| Valid | 1 | 2 | .0 | .0 | .0 |
|  | 2 | 30551 | 100.0 | 100.0 | 100.0 |
|  | Total | 30553 | 100.0 | 100.0 |  |
|  |  |  |  |  |  |

OthersOthers, table, 1 levels of column headers and 2 levels of row headers, table with 6 columns and 5 rows

|  |  |  |  |  |  |
| --- | --- | --- | --- | --- | --- |
|  | | Frequency | Percent | Valid Percent | Cumulative Percent |
| Valid | 1 | 210 | .7 | .7 | .7 |
|  | 2 | 30343 | 99.3 | 99.3 | 100.0 |
|  | Total | 30553 | 100.0 | 100.0 |  |
|  |  |  |  |  |  |

Other specifyOther specify, table, 1 levels of column headers and 2 levels of row headers, table with 6 columns and 3 rows

|  |  |  |  |  |  |
| --- | --- | --- | --- | --- | --- |
|  | | Frequency | Percent | Valid Percent | Cumulative Percent |
| Valid |  | 30553 | 100.0 | 100.0 | 100.0 |
|  |  |  |  |  |  |

Satisfaction level with the playpen interventionSatisfaction level with the playpen intervention, table, 1 levels of column headers and 2 levels of row headers, table with 6 columns and 8 rows

|  |  |  |  |  |  |
| --- | --- | --- | --- | --- | --- |
|  | | Frequency | Percent | Valid Percent | Cumulative Percent |
| Valid | Very dissatisfied | 1068 | 3.5 | 3.5 | 3.5 |
|  | Moderately dissatisfiwd | 174 | .6 | .6 | 4.1 |
|  | Neigher satisfied or dissatisfied | 414 | 1.4 | 1.4 | 5.4 |
|  | Moderately satisfied | 1383 | 4.5 | 4.5 | 9.9 |
|  | Very satisfied | 27514 | 90.1 | 90.1 | 100.0 |
|  | Total | 30553 | 100.0 | 100.0 |  |
|  |  |  |  |  |  |

Any suggestions to improve the playpen interventionAny suggestions to improve the playpen intervention, table, 1 levels of column headers and 2 levels of row headers, table with 6 columns and 6 rows

|  |  |  |  |  |  |
| --- | --- | --- | --- | --- | --- |
|  | | Frequency | Percent | Valid Percent | Cumulative Percent |
| Valid |  | 476 | 1.6 | 1.6 | 1.6 |
|  | Yes (Specify) | 2441 | 8.0 | 8.0 | 9.5 |
|  | No | 27636 | 90.5 | 90.5 | 100.0 |
|  | Total | 30553 | 100.0 | 100.0 |  |
|  |  |  |  |  |  |

Code of suggestionCode of suggestion, table, 1 levels of column headers and 2 levels of row headers, table with 6 columns and 7 rows

|  |  |  |  |  |  |
| --- | --- | --- | --- | --- | --- |
|  | | Frequency | Percent | Valid Percent | Cumulative Percent |
| Valid |  | 30541 | 100.0 | 100.0 | 100.0 |
|  | 1 | 2 | .0 | .0 | 100.0 |
|  | 2 | 8 | .0 | .0 | 100.0 |
|  | 5 | 2 | .0 | .0 | 100.0 |
|  | Total | 30553 | 100.0 | 100.0 |  |
|  |  |  |  |  |  |

Currently participate in any income generating activitiesCurrently participate in any income generating activities, table, 1 levels of column headers and 2 levels of row headers, table with 6 columns and 5 rows

|  |  |  |  |  |  |
| --- | --- | --- | --- | --- | --- |
|  | | Frequency | Percent | Valid Percent | Cumulative Percent |
| Valid | Yes | 2928 | 9.6 | 9.6 | 9.6 |
|  | No | 27625 | 90.4 | 90.4 | 100.0 |
|  | Total | 30553 | 100.0 | 100.0 |  |
|  |  |  |  |  |  |

Number of hours per day devote to income generating activitiesNumber of hours per day devote to income generating activities, table, 1 levels of column headers and 2 levels of row headers, table with 6 columns and 12 rows

|  |  |  |  |  |  |
| --- | --- | --- | --- | --- | --- |
|  | | Frequency | Percent | Valid Percent | Cumulative Percent |
| Valid | None | 27625 | 90.4 | 90.4 | 90.4 |
|  | 1 | 93 | .3 | .3 | 90.7 |
|  | 2 | 393 | 1.3 | 1.3 | 92.0 |
|  | 3 | 433 | 1.4 | 1.4 | 93.4 |
|  | 4 | 862 | 2.8 | 2.8 | 96.2 |
|  | 5 | 413 | 1.4 | 1.4 | 97.6 |
|  | 6 | 408 | 1.3 | 1.3 | 98.9 |
|  | 7 | 126 | .4 | .4 | 99.3 |
|  | 8 | 200 | .7 | .7 | 100.0 |
|  | Total | 30553 | 100.0 | 100.0 |  |
|  |  |  |  |  |  |

Numder of hours per day devote to household choresNumder of hours per day devote to household chores, table, 1 levels of column headers and 2 levels of row headers, table with 6 columns and 14 rows

|  |  |  |  |  |  |
| --- | --- | --- | --- | --- | --- |
|  | | Frequency | Percent | Valid Percent | Cumulative Percent |
| Valid | None | 324 | 1.1 | 1.1 | 1.1 |
|  | 1.00 | 157 | .5 | .5 | 1.6 |
|  | 2.00 | 1063 | 3.5 | 3.5 | 5.1 |
|  | 3.00 | 2515 | 8.2 | 8.3 | 13.3 |
|  | 4.00 | 5294 | 17.3 | 17.4 | 30.7 |
|  | 5.00 | 5936 | 19.4 | 19.5 | 50.2 |
|  | 6.00 | 8328 | 27.3 | 27.3 | 77.5 |
|  | 7.00 | 3317 | 10.9 | 10.9 | 88.4 |
|  | 8.00 | 3542 | 11.6 | 11.6 | 100.0 |
|  | Total | 30476 | 99.7 | 100.0 |  |
| Missing | System | 77 | .3 |  |  |
| Total | | 30553 | 100.0 |  |  |
|  |  |  |  |  |  |

Marital statusMarital status, table, 1 levels of column headers and 2 levels of row headers, table with 6 columns and 9 rows

|  |  |  |  |  |  |
| --- | --- | --- | --- | --- | --- |
|  | | Frequency | Percent | Valid Percent | Cumulative Percent |
| Valid | Married | 16639 | 54.5 | 54.5 | 54.5 |
|  | Never married | 4886 | 16.0 | 16.0 | 70.5 |
|  | Divorced | 67 | .2 | .2 | 70.7 |
|  | Widowed | 1234 | 4.0 | 4.0 | 74.7 |
|  | Separated | 63 | .2 | .2 | 74.9 |
|  | Not applicable | 7664 | 25.1 | 25.1 | 100.0 |
|  | Total | 30553 | 100.0 | 100.0 |  |
|  |  |  |  |  |  |

Education of the individualEducation of the individual, table, 1 levels of column headers and 2 levels of row headers, table with 6 columns and 22 rows

|  |  |  |  |  |  |
| --- | --- | --- | --- | --- | --- |
|  | | Frequency | Percent | Valid Percent | Cumulative Percent |
| Valid | 1 | 2115 | 6.9 | 6.9 | 6.9 |
|  | 2 | 1337 | 4.4 | 4.4 | 11.3 |
|  | 3 | 1337 | 4.4 | 4.4 | 15.7 |
|  | 4 | 1121 | 3.7 | 3.7 | 19.3 |
|  | 5 | 3781 | 12.4 | 12.4 | 31.7 |
|  | 6 | 867 | 2.8 | 2.8 | 34.6 |
|  | 7 | 740 | 2.4 | 2.4 | 37.0 |
|  | 8 | 1289 | 4.2 | 4.2 | 41.2 |
|  | 9 | 1011 | 3.3 | 3.3 | 44.5 |
|  | Completed S.S.C | 1030 | 3.4 | 3.4 | 47.9 |
|  | Intermediate 1st year complete | 274 | .9 | .9 | 48.8 |
|  | Completed H.S.C | 709 | 2.3 | 2.3 | 51.1 |
|  | Graduation/ Hon's 1st year complete | 36 | .1 | .1 | 51.2 |
|  | Completed BA/BCOM/BSC/Graduation(2 years) complete/Hons' 2nd year complete | 183 | .6 | .6 | 51.8 |
|  | Graduation complete/ Hon's 3rd year complete | 20 | .1 | .1 | 51.9 |
|  | Hon's complete/ Preliminary Masters complete | 52 | .2 | .2 | 52.0 |
|  | No academic education | 12106 | 39.6 | 39.6 | 91.7 |
|  | Not applicable(if age < 5 years) | 2498 | 8.2 | 8.2 | 99.8 |
|  | Don’t know | 47 | .2 | .2 | 100.0 |
|  | Total | 30553 | 100.0 | 100.0 |  |
|  |  |  |  |  |  |

Primary occupationPrimary occupation, table, 1 levels of column headers and 2 levels of row headers, table with 6 columns and 20 rows

|  |  |  |  |  |  |
| --- | --- | --- | --- | --- | --- |
|  | | Frequency | Percent | Valid Percent | Cumulative Percent |
| Valid | 1 | 5138 | 16.8 | 16.8 | 16.8 |
|  | 2 | 1256 | 4.1 | 4.1 | 20.9 |
|  | 3 | 7461 | 24.4 | 24.4 | 45.3 |
|  | 4 | 8942 | 29.3 | 29.3 | 74.6 |
|  | 5 | 722 | 2.4 | 2.4 | 77.0 |
|  | 6 | 523 | 1.7 | 1.7 | 78.7 |
|  | 7 | 1382 | 4.5 | 4.5 | 83.2 |
|  | 8 | 249 | .8 | .8 | 84.0 |
|  | 9 | 35 | .1 | .1 | 84.1 |
|  | Retired | 926 | 3.0 | 3.0 | 87.2 |
|  | Van/rickshaw driver | 291 | 1.0 | 1.0 | 88.1 |
|  | CNG/bus/truck driver | 115 | .4 | .4 | 88.5 |
|  | 6. | 1 | .0 | .0 | 88.5 |
|  | Under age 6 years | 3193 | 10.5 | 10.5 | 99.0 |
|  | Not applicable | 279 | .9 | .9 | 99.9 |
|  | Others | 29 | .1 | .1 | 100.0 |
|  | Don't know | 11 | .0 | .0 | 100.0 |
|  | Total | 30553 | 100.0 | 100.0 |  |
|  |  |  |  |  |  |

OrganizationOrganization, table, 1 levels of column headers and 2 levels of row headers, table with 6 columns and 5 rows

|  |  |  |  |  |  |
| --- | --- | --- | --- | --- | --- |
|  | | Frequency | Percent | Valid Percent | Cumulative Percent |
| Valid | CIPRB | 1121 | 3.7 | 100.0 | 100.0 |
| Missing | System | 29432 | 96.3 |  |  |
| Total | | 30553 | 100.0 |  |  |
|  |  |  |  |  |  |

Unique Person IdentifierUnique Person Identifier, table, 1 levels of column headers and 2 levels of row headers, table with 6 columns and 1123 rows

|  |  |  |  |  |  |
| --- | --- | --- | --- | --- | --- |
|  | | Frequency | Percent | Valid Percent | Cumulative Percent |
| Valid |  | 29432 | 96.3 | 96.3 | 96.3 |
|  | 4404001028404 | 1 | .0 | .0 | 96.3 |
|  | 4404001033002 | 1 | .0 | .0 | 96.3 |
|  | 4404001046103 | 1 | .0 | .0 | 96.3 |
|  | 4404001049303 | 1 | .0 | .0 | 96.3 |
|  | 4404001052101 | 1 | .0 | .0 | 96.3 |
|  | 4404001054102 | 1 | .0 | .0 | 96.4 |
|  | 4404001061304 | 1 | .0 | .0 | 96.4 |
|  | 4404001064002 | 1 | .0 | .0 | 96.4 |
|  | 4404001067802 | 1 | .0 | .0 | 96.4 |
|  | 4404001083504 | 1 | .0 | .0 | 96.4 |
|  | 4404002004402 | 1 | .0 | .0 | 96.4 |
|  | 4404002011304 | 1 | .0 | .0 | 96.4 |
|  | 4404002013302 | 1 | .0 | .0 | 96.4 |
|  | 4404002013502 | 1 | .0 | .0 | 96.4 |
|  | 4404002016204 | 1 | .0 | .0 | 96.4 |
|  | 4404002021805 | 1 | .0 | .0 | 96.4 |
|  | 4404002025002 | 1 | .0 | .0 | 96.4 |
|  | 4404002034003 | 1 | .0 | .0 | 96.4 |
|  | 4404002040202 | 1 | .0 | .0 | 96.4 |
|  | 4404002040904 | 1 | .0 | .0 | 96.4 |
|  | 4404002046504 | 1 | .0 | .0 | 96.4 |
|  | 4404002048504 | 1 | .0 | .0 | 96.4 |
|  | 4404002053801 | 1 | .0 | .0 | 96.4 |
|  | 4404002054601 | 1 | .0 | .0 | 96.4 |
|  | 4404002062601 | 1 | .0 | .0 | 96.4 |
|  | 4404002065802 | 1 | .0 | .0 | 96.4 |
|  | 4404002067912 | 1 | .0 | .0 | 96.4 |
|  | 4404002068701 | 1 | .0 | .0 | 96.4 |
|  | 4404002071401 | 1 | .0 | .0 | 96.4 |
|  | 4404002074501 | 1 | .0 | .0 | 96.4 |
|  | 4404002075004 | 1 | .0 | .0 | 96.4 |
|  | 4404002083801 | 1 | .0 | .0 | 96.4 |
|  | 4404002084301 | 1 | .0 | .0 | 96.4 |
|  | 4404003003605 | 1 | .0 | .0 | 96.4 |
|  | 4404004003301 | 1 | .0 | .0 | 96.4 |
|  | 4404004006302 | 1 | .0 | .0 | 96.4 |
|  | 4404005004003 | 1 | .0 | .0 | 96.5 |
|  | 4404005004801 | 1 | .0 | .0 | 96.5 |
|  | 4404005011402 | 1 | .0 | .0 | 96.5 |
|  | 4404005016501 | 1 | .0 | .0 | 96.5 |
|  | 4404005016702 | 1 | .0 | .0 | 96.5 |
|  | 4404005020603 | 1 | .0 | .0 | 96.5 |
|  | 4404005031101 | 1 | .0 | .0 | 96.5 |
|  | 4404005045103 | 1 | .0 | .0 | 96.5 |
|  | 4404006001203 | 1 | .0 | .0 | 96.5 |
|  | 4404006018404 | 1 | .0 | .0 | 96.5 |
|  | 4404006021408 | 1 | .0 | .0 | 96.5 |
|  | 4404007019703 | 1 | .0 | .0 | 96.5 |
|  | 4404007027501 | 1 | .0 | .0 | 96.5 |
|  | 4404007029502 | 1 | .0 | .0 | 96.5 |
|  | 4404007030802 | 1 | .0 | .0 | 96.5 |
|  | 4404007033901 | 1 | .0 | .0 | 96.5 |
|  | 4404007035405 | 1 | .0 | .0 | 96.5 |
|  | 4404007043503 | 1 | .0 | .0 | 96.5 |
|  | 4404007045701 | 1 | .0 | .0 | 96.5 |
|  | 4404007046004 | 1 | .0 | .0 | 96.5 |
|  | 4404007048602 | 1 | .0 | .0 | 96.5 |
|  | 4404007052003 | 1 | .0 | .0 | 96.5 |
|  | 4404007052103 | 1 | .0 | .0 | 96.5 |
|  | 4404007053104 | 1 | .0 | .0 | 96.5 |
|  | 4404007053501 | 1 | .0 | .0 | 96.5 |
|  | 4404007056806 | 1 | .0 | .0 | 96.5 |
|  | 4404007057801 | 1 | .0 | .0 | 96.5 |
|  | 4404008038801 | 1 | .0 | .0 | 96.5 |
|  | 4404008046005 | 1 | .0 | .0 | 96.5 |
|  | 4404008050603 | 1 | .0 | .0 | 96.5 |
|  | 4404009006201 | 1 | .0 | .0 | 96.6 |
|  | 4404009016401 | 1 | .0 | .0 | 96.6 |
|  | 4404010004905 | 1 | .0 | .0 | 96.6 |
|  | 4404010006302 | 1 | .0 | .0 | 96.6 |
|  | 4404010008704 | 1 | .0 | .0 | 96.6 |
|  | 4404010012101 | 1 | .0 | .0 | 96.6 |
|  | 4404010015307 | 1 | .0 | .0 | 96.6 |
|  | 4404010015903 | 1 | .0 | .0 | 96.6 |
|  | 4404011001104 | 1 | .0 | .0 | 96.6 |
|  | 4404011001202 | 1 | .0 | .0 | 96.6 |
|  | 4404011012502 | 1 | .0 | .0 | 96.6 |
|  | 4404011039001 | 1 | .0 | .0 | 96.6 |
|  | 4404011041304 | 1 | .0 | .0 | 96.6 |
|  | 4404011043204 | 1 | .0 | .0 | 96.6 |
|  | 4404011045402 | 1 | .0 | .0 | 96.6 |
|  | 4404011045503 | 1 | .0 | .0 | 96.6 |
|  | 4404011049602 | 1 | .0 | .0 | 96.6 |
|  | 4404011055201 | 1 | .0 | .0 | 96.6 |
|  | 4404012000301 | 1 | .0 | .0 | 96.6 |
|  | 4404012001502 | 1 | .0 | .0 | 96.6 |
|  | 4404012008803 | 1 | .0 | .0 | 96.6 |
|  | 4404012009005 | 1 | .0 | .0 | 96.6 |
|  | 4404012010201 | 1 | .0 | .0 | 96.6 |
|  | 4404012011102 | 1 | .0 | .0 | 96.6 |
|  | 4404012012402 | 1 | .0 | .0 | 96.6 |
|  | 4404012013905 | 1 | .0 | .0 | 96.6 |
|  | 4404012016503 | 1 | .0 | .0 | 96.6 |
|  | 4404013016901 | 1 | .0 | .0 | 96.6 |
|  | 4404013018005 | 1 | .0 | .0 | 96.6 |
|  | 4404013028105 | 1 | .0 | .0 | 96.6 |
|  | 4404013028903 | 1 | .0 | .0 | 96.6 |
|  | 4404013029204 | 1 | .0 | .0 | 96.7 |
|  | 4404013031702 | 1 | .0 | .0 | 96.7 |
|  | 4404013032503 | 1 | .0 | .0 | 96.7 |
|  | 4404013032601 | 1 | .0 | .0 | 96.7 |
|  | 4404013034502 | 1 | .0 | .0 | 96.7 |
|  | 4404013035104 | 1 | .0 | .0 | 96.7 |
|  | 4404013035201 | 1 | .0 | .0 | 96.7 |
|  | 4404013037404 | 1 | .0 | .0 | 96.7 |
|  | 4404013041603 | 1 | .0 | .0 | 96.7 |
|  | 4404013043201 | 1 | .0 | .0 | 96.7 |
|  | 4404013045103 | 1 | .0 | .0 | 96.7 |
|  | 4404013046903 | 1 | .0 | .0 | 96.7 |
|  | 4404013047003 | 1 | .0 | .0 | 96.7 |
|  | 4404013051002 | 1 | .0 | .0 | 96.7 |
|  | 4404013066101 | 1 | .0 | .0 | 96.7 |
|  | 4404013066401 | 1 | .0 | .0 | 96.7 |
|  | 4404013069102 | 1 | .0 | .0 | 96.7 |
|  | 4404013069901 | 1 | .0 | .0 | 96.7 |
|  | 4404013073203 | 1 | .0 | .0 | 96.7 |
|  | 4404013073905 | 1 | .0 | .0 | 96.7 |
|  | 4404013081601 | 1 | .0 | .0 | 96.7 |
|  | 4404013087801 | 1 | .0 | .0 | 96.7 |
|  | 4404013087905 | 1 | .0 | .0 | 96.7 |
|  | 4404013095602 | 1 | .0 | .0 | 96.7 |
|  | 4404014000202 | 1 | .0 | .0 | 96.7 |
|  | 4404014000305 | 1 | .0 | .0 | 96.7 |
|  | 4404014005401 | 1 | .0 | .0 | 96.7 |
|  | 4404014005604 | 1 | .0 | .0 | 96.7 |
|  | 4404014008602 | 1 | .0 | .0 | 96.7 |
|  | 4404014010602 | 1 | .0 | .0 | 96.7 |
|  | 4404014012403 | 1 | .0 | .0 | 96.7 |
|  | 4404014013302 | 1 | .0 | .0 | 96.8 |
|  | 4404014015803 | 1 | .0 | .0 | 96.8 |
|  | 4404014016201 | 1 | .0 | .0 | 96.8 |
|  | 4404014016805 | 1 | .0 | .0 | 96.8 |
|  | 4404014017305 | 1 | .0 | .0 | 96.8 |
|  | 4404014017801 | 1 | .0 | .0 | 96.8 |
|  | 4404014018201 | 1 | .0 | .0 | 96.8 |
|  | 4404014018701 | 1 | .0 | .0 | 96.8 |
|  | 4404014020203 | 1 | .0 | .0 | 96.8 |
|  | 4404015007608 | 1 | .0 | .0 | 96.8 |
|  | 4404015009605 | 1 | .0 | .0 | 96.8 |
|  | 4404015021103 | 1 | .0 | .0 | 96.8 |
|  | 4404015021701 | 1 | .0 | .0 | 96.8 |
|  | 4404016008501 | 1 | .0 | .0 | 96.8 |
|  | 4404017007102 | 1 | .0 | .0 | 96.8 |
|  | 4404017014102 | 1 | .0 | .0 | 96.8 |
|  | 4404018005304 | 1 | .0 | .0 | 96.8 |
|  | 4404018007203 | 1 | .0 | .0 | 96.8 |
|  | 4404018007601 | 1 | .0 | .0 | 96.8 |
|  | 4404018010705 | 1 | .0 | .0 | 96.8 |
|  | 4404018013307 | 1 | .0 | .0 | 96.8 |
|  | 4404018013805 | 1 | .0 | .0 | 96.8 |
|  | 4404018017903 | 1 | .0 | .0 | 96.8 |
|  | 4405001002601 | 1 | .0 | .0 | 96.8 |
|  | 4405001012301 | 1 | .0 | .0 | 96.8 |
|  | 4405001014402 | 1 | .0 | .0 | 96.8 |
|  | 4405001020703 | 1 | .0 | .0 | 96.8 |
|  | 4405002017601 | 1 | .0 | .0 | 96.8 |
|  | 4405002019704 | 1 | .0 | .0 | 96.8 |
|  | 4405002024504 | 1 | .0 | .0 | 96.8 |
|  | 4405002028208 | 1 | .0 | .0 | 96.9 |
|  | 4405002028401 | 1 | .0 | .0 | 96.9 |
|  | 4405002030301 | 1 | .0 | .0 | 96.9 |
|  | 4405002033301 | 1 | .0 | .0 | 96.9 |
|  | 4405002041803 | 1 | .0 | .0 | 96.9 |
|  | 4405002042502 | 1 | .0 | .0 | 96.9 |
|  | 4405003007101 | 1 | .0 | .0 | 96.9 |
|  | 4405008007201 | 1 | .0 | .0 | 96.9 |
|  | 4405008018203 | 1 | .0 | .0 | 96.9 |
|  | 4405008031201 | 1 | .0 | .0 | 96.9 |
|  | 4405008040001 | 1 | .0 | .0 | 96.9 |
|  | 4405008054301 | 1 | .0 | .0 | 96.9 |
|  | 4405010001401 | 1 | .0 | .0 | 96.9 |
|  | 4405010014104 | 1 | .0 | .0 | 96.9 |
|  | 4405010024106 | 1 | .0 | .0 | 96.9 |
|  | 4405010029601 | 1 | .0 | .0 | 96.9 |
|  | 4405010047701 | 1 | .0 | .0 | 96.9 |
|  | 4405010078503 | 1 | .0 | .0 | 96.9 |
|  | 4405010082601 | 1 | .0 | .0 | 96.9 |
|  | 4405010085207 | 1 | .0 | .0 | 96.9 |
|  | 4405010085602 | 1 | .0 | .0 | 96.9 |
|  | 4405010094901 | 1 | .0 | .0 | 96.9 |
|  | 4405010098603 | 1 | .0 | .0 | 96.9 |
|  | 4405010117001 | 1 | .0 | .0 | 96.9 |
|  | 4405010118601 | 1 | .0 | .0 | 96.9 |
|  | 4405010119901 | 1 | .0 | .0 | 96.9 |
|  | 4405010121305 | 1 | .0 | .0 | 96.9 |
|  | 4405010125201 | 1 | .0 | .0 | 96.9 |
|  | 4405010127614 | 1 | .0 | .0 | 96.9 |
|  | 4405010131201 | 1 | .0 | .0 | 96.9 |
|  | 4405010132407 | 1 | .0 | .0 | 96.9 |
|  | 4405010134002 | 1 | .0 | .0 | 97.0 |
|  | 4405010147304 | 1 | .0 | .0 | 97.0 |
|  | 4405012012001 | 1 | .0 | .0 | 97.0 |
|  | 4405013031704 | 1 | .0 | .0 | 97.0 |
|  | 4405015006305 | 1 | .0 | .0 | 97.0 |
|  | 4405016010103 | 1 | .0 | .0 | 97.0 |
|  | 4405017001101 | 1 | .0 | .0 | 97.0 |
|  | 4405017030802 | 1 | .0 | .0 | 97.0 |
|  | 4405017069102 | 1 | .0 | .0 | 97.0 |
|  | 4405018000602 | 1 | .0 | .0 | 97.0 |
|  | 4405018020102 | 1 | .0 | .0 | 97.0 |
|  | 4405018029006 | 1 | .0 | .0 | 97.0 |
|  | 4405018043803 | 1 | .0 | .0 | 97.0 |
|  | 4405018047902 | 1 | .0 | .0 | 97.0 |
|  | 4405018049102 | 1 | .0 | .0 | 97.0 |
|  | 4405018058406 | 1 | .0 | .0 | 97.0 |
|  | 4405018078101 | 1 | .0 | .0 | 97.0 |
|  | 4405018078105 | 1 | .0 | .0 | 97.0 |
|  | 4405018080805 | 1 | .0 | .0 | 97.0 |
|  | 4405018088002 | 1 | .0 | .0 | 97.0 |
|  | 4405018095402 | 1 | .0 | .0 | 97.0 |
|  | 4405018098001 | 1 | .0 | .0 | 97.0 |
|  | 4405019013202 | 1 | .0 | .0 | 97.0 |
|  | 4405020006201 | 1 | .0 | .0 | 97.0 |
|  | 4405020011901 | 1 | .0 | .0 | 97.0 |
|  | 4405020012101 | 1 | .0 | .0 | 97.0 |
|  | 4405020015001 | 1 | .0 | .0 | 97.0 |
|  | 4405020022903 | 1 | .0 | .0 | 97.0 |
|  | 4405020034103 | 1 | .0 | .0 | 97.0 |
|  | 4405020034202 | 1 | .0 | .0 | 97.0 |
|  | 4405020039001 | 1 | .0 | .0 | 97.1 |
|  | 4405020040602 | 1 | .0 | .0 | 97.1 |
|  | 4405020046402 | 1 | .0 | .0 | 97.1 |
|  | 4405020054301 | 1 | .0 | .0 | 97.1 |
|  | 4405020059205 | 1 | .0 | .0 | 97.1 |
|  | 4405020059901 | 2 | .0 | .0 | 97.1 |
|  | 4405020060701 | 1 | .0 | .0 | 97.1 |
|  | 4405020060903 | 1 | .0 | .0 | 97.1 |
|  | 4405020061702 | 1 | .0 | .0 | 97.1 |
|  | 4405020061902 | 1 | .0 | .0 | 97.1 |
|  | 4405020065413 | 1 | .0 | .0 | 97.1 |
|  | 4405020071301 | 1 | .0 | .0 | 97.1 |
|  | 4405020078803 | 1 | .0 | .0 | 97.1 |
|  | 4405026000704 | 1 | .0 | .0 | 97.1 |
|  | 4406001001501 | 1 | .0 | .0 | 97.1 |
|  | 4406001001601 | 1 | .0 | .0 | 97.1 |
|  | 4406001001603 | 1 | .0 | .0 | 97.1 |
|  | 4406001001902 | 1 | .0 | .0 | 97.1 |
|  | 4406001004401 | 1 | .0 | .0 | 97.1 |
|  | 4406001004602 | 1 | .0 | .0 | 97.1 |
|  | 4406001006502 | 1 | .0 | .0 | 97.1 |
|  | 4406001009703 | 1 | .0 | .0 | 97.1 |
|  | 4406001010301 | 1 | .0 | .0 | 97.1 |
|  | 4406001010904 | 1 | .0 | .0 | 97.1 |
|  | 4406001011103 | 1 | .0 | .0 | 97.1 |
|  | 4406001012503 | 1 | .0 | .0 | 97.1 |
|  | 4406001014504 | 1 | .0 | .0 | 97.1 |
|  | 4406001023901 | 1 | .0 | .0 | 97.1 |
|  | 4406001026906 | 1 | .0 | .0 | 97.1 |
|  | 4406001027801 | 1 | .0 | .0 | 97.1 |
|  | 4406001032201 | 1 | .0 | .0 | 97.2 |
|  | 4406001033204 | 1 | .0 | .0 | 97.2 |
|  | 4406001033601 | 1 | .0 | .0 | 97.2 |
|  | 4406002001301 | 1 | .0 | .0 | 97.2 |
|  | 4406002001705 | 1 | .0 | .0 | 97.2 |
|  | 4406002003002 | 1 | .0 | .0 | 97.2 |
|  | 4406002003801 | 1 | .0 | .0 | 97.2 |
|  | 4406002003804 | 1 | .0 | .0 | 97.2 |
|  | 4406002006802 | 1 | .0 | .0 | 97.2 |
|  | 4406002007202 | 1 | .0 | .0 | 97.2 |
|  | 4406002008001 | 1 | .0 | .0 | 97.2 |
|  | 4406002010103 | 1 | .0 | .0 | 97.2 |
|  | 4406002015203 | 1 | .0 | .0 | 97.2 |
|  | 4406002016506 | 1 | .0 | .0 | 97.2 |
|  | 4406002017401 | 1 | .0 | .0 | 97.2 |
|  | 4406002017502 | 1 | .0 | .0 | 97.2 |
|  | 4406002018904 | 1 | .0 | .0 | 97.2 |
|  | 4406002022301 | 1 | .0 | .0 | 97.2 |
|  | 4406002022402 | 1 | .0 | .0 | 97.2 |
|  | 4406003011505 | 1 | .0 | .0 | 97.2 |
|  | 4406003011803 | 1 | .0 | .0 | 97.2 |
|  | 4406003011902 | 1 | .0 | .0 | 97.2 |
|  | 4406004000609 | 1 | .0 | .0 | 97.2 |
|  | 4406004006101 | 1 | .0 | .0 | 97.2 |
|  | 4406004007603 | 1 | .0 | .0 | 97.2 |
|  | 4406004007803 | 1 | .0 | .0 | 97.2 |
|  | 4406004008001 | 1 | .0 | .0 | 97.2 |
|  | 4406004009106 | 1 | .0 | .0 | 97.2 |
|  | 4406004010103 | 1 | .0 | .0 | 97.2 |
|  | 4406004015202 | 1 | .0 | .0 | 97.2 |
|  | 4406004017901 | 1 | .0 | .0 | 97.3 |
|  | 4406004019507 | 1 | .0 | .0 | 97.3 |
|  | 4406005009103 | 1 | .0 | .0 | 97.3 |
|  | 4406005011302 | 1 | .0 | .0 | 97.3 |
|  | 4406005013201 | 1 | .0 | .0 | 97.3 |
|  | 4406005013604 | 1 | .0 | .0 | 97.3 |
|  | 4406005014301 | 1 | .0 | .0 | 97.3 |
|  | 4406005014306 | 1 | .0 | .0 | 97.3 |
|  | 4406005016901 | 1 | .0 | .0 | 97.3 |
|  | 4406005018209 | 1 | .0 | .0 | 97.3 |
|  | 4406005030402 | 1 | .0 | .0 | 97.3 |
|  | 4406005030901 | 1 | .0 | .0 | 97.3 |
|  | 4406005032003 | 1 | .0 | .0 | 97.3 |
|  | 4406005032602 | 1 | .0 | .0 | 97.3 |
|  | 4406005036002 | 1 | .0 | .0 | 97.3 |
|  | 4406005036808 | 1 | .0 | .0 | 97.3 |
|  | 4406005037504 | 1 | .0 | .0 | 97.3 |
|  | 4406005039304 | 1 | .0 | .0 | 97.3 |
|  | 4406005041808 | 1 | .0 | .0 | 97.3 |
|  | 4406006001506 | 1 | .0 | .0 | 97.3 |
|  | 4406006004102 | 1 | .0 | .0 | 97.3 |
|  | 4406007009903 | 1 | .0 | .0 | 97.3 |
|  | 4406007010701 | 1 | .0 | .0 | 97.3 |
|  | 4406007010704 | 1 | .0 | .0 | 97.3 |
|  | 4406007011402 | 1 | .0 | .0 | 97.3 |
|  | 4406007012505 | 1 | .0 | .0 | 97.3 |
|  | 4406007012803 | 1 | .0 | .0 | 97.3 |
|  | 4406007012906 | 1 | .0 | .0 | 97.3 |
|  | 4406007013703 | 1 | .0 | .0 | 97.3 |
|  | 4406007014501 | 1 | .0 | .0 | 97.3 |
|  | 4406007016203 | 1 | .0 | .0 | 97.3 |
|  | 4406007017202 | 1 | .0 | .0 | 97.4 |
|  | 4406007019402 | 1 | .0 | .0 | 97.4 |
|  | 4406007023001 | 1 | .0 | .0 | 97.4 |
|  | 4406007028101 | 1 | .0 | .0 | 97.4 |
|  | 4406007029304 | 1 | .0 | .0 | 97.4 |
|  | 4406007030103 | 1 | .0 | .0 | 97.4 |
|  | 4406007030701 | 1 | .0 | .0 | 97.4 |
|  | 4406008015603 | 1 | .0 | .0 | 97.4 |
|  | 4406008017804 | 1 | .0 | .0 | 97.4 |
|  | 4406008027608 | 1 | .0 | .0 | 97.4 |
|  | 4406008034703 | 1 | .0 | .0 | 97.4 |
|  | 4406008038104 | 1 | .0 | .0 | 97.4 |
|  | 4406008046407 | 1 | .0 | .0 | 97.4 |
|  | 4406008047402 | 1 | .0 | .0 | 97.4 |
|  | 4406009015601 | 1 | .0 | .0 | 97.4 |
|  | 4406010000102 | 1 | .0 | .0 | 97.4 |
|  | 4406010002004 | 1 | .0 | .0 | 97.4 |
|  | 4406010003604 | 1 | .0 | .0 | 97.4 |
|  | 4406010004105 | 1 | .0 | .0 | 97.4 |
|  | 4406010008202 | 1 | .0 | .0 | 97.4 |
|  | 4406010013601 | 1 | .0 | .0 | 97.4 |
|  | 4406011004506 | 1 | .0 | .0 | 97.4 |
|  | 4406011009703 | 1 | .0 | .0 | 97.4 |
|  | 4406011010202 | 1 | .0 | .0 | 97.4 |
|  | 4406011010305 | 1 | .0 | .0 | 97.4 |
|  | 4406011010903 | 1 | .0 | .0 | 97.4 |
|  | 4406011012802 | 1 | .0 | .0 | 97.4 |
|  | 4406011012902 | 1 | .0 | .0 | 97.4 |
|  | 4406011014202 | 1 | .0 | .0 | 97.4 |
|  | 4406011017401 | 1 | .0 | .0 | 97.4 |
|  | 4406011020502 | 1 | .0 | .0 | 97.5 |
|  | 4406011020603 | 1 | .0 | .0 | 97.5 |
|  | 4406011021204 | 1 | .0 | .0 | 97.5 |
|  | 4406011021302 | 1 | .0 | .0 | 97.5 |
|  | 4406011021402 | 1 | .0 | .0 | 97.5 |
|  | 4406011021510 | 1 | .0 | .0 | 97.5 |
|  | 4406011023103 | 1 | .0 | .0 | 97.5 |
|  | 4406011025104 | 1 | .0 | .0 | 97.5 |
|  | 4406011026402 | 1 | .0 | .0 | 97.5 |
|  | 4406011033103 | 1 | .0 | .0 | 97.5 |
|  | 4406011040101 | 1 | .0 | .0 | 97.5 |
|  | 4406011058703 | 1 | .0 | .0 | 97.5 |
|  | 4406011078503 | 1 | .0 | .0 | 97.5 |
|  | 4406012001803 | 1 | .0 | .0 | 97.5 |
|  | 4406012004305 | 1 | .0 | .0 | 97.5 |
|  | 4406012006401 | 1 | .0 | .0 | 97.5 |
|  | 4406012006804 | 1 | .0 | .0 | 97.5 |
|  | 4406012010702 | 1 | .0 | .0 | 97.5 |
|  | 4406012011201 | 1 | .0 | .0 | 97.5 |
|  | 4406012016301 | 1 | .0 | .0 | 97.5 |
|  | 4406012017001 | 1 | .0 | .0 | 97.5 |
|  | 4406012017402 | 1 | .0 | .0 | 97.5 |
|  | 4406012019804 | 1 | .0 | .0 | 97.5 |
|  | 4406012022102 | 1 | .0 | .0 | 97.5 |
|  | 4406012027404 | 1 | .0 | .0 | 97.5 |
|  | 4406012027804 | 1 | .0 | .0 | 97.5 |
|  | 4406013004701 | 1 | .0 | .0 | 97.5 |
|  | 4406013005006 | 1 | .0 | .0 | 97.5 |
|  | 4406013006407 | 1 | .0 | .0 | 97.5 |
|  | 4406013011708 | 1 | .0 | .0 | 97.5 |
|  | 4406013013602 | 1 | .0 | .0 | 97.5 |
|  | 4406013015401 | 1 | .0 | .0 | 97.6 |
|  | 4406013018303 | 1 | .0 | .0 | 97.6 |
|  | 4406013020401 | 1 | .0 | .0 | 97.6 |
|  | 4406013021107 | 1 | .0 | .0 | 97.6 |
|  | 4406013025905 | 1 | .0 | .0 | 97.6 |
|  | 4406013027502 | 1 | .0 | .0 | 97.6 |
|  | 4406013027802 | 1 | .0 | .0 | 97.6 |
|  | 4406013029205 | 1 | .0 | .0 | 97.6 |
|  | 4406013029901 | 1 | .0 | .0 | 97.6 |
|  | 4406013031304 | 1 | .0 | .0 | 97.6 |
|  | 4406013032101 | 1 | .0 | .0 | 97.6 |
|  | 4406013034305 | 1 | .0 | .0 | 97.6 |
|  | 4406013040302 | 1 | .0 | .0 | 97.6 |
|  | 4406013047701 | 1 | .0 | .0 | 97.6 |
|  | 4406013049901 | 1 | .0 | .0 | 97.6 |
|  | 4406013058402 | 1 | .0 | .0 | 97.6 |
|  | 4406013058502 | 1 | .0 | .0 | 97.6 |
|  | 4406013059403 | 1 | .0 | .0 | 97.6 |
|  | 4406013059801 | 1 | .0 | .0 | 97.6 |
|  | 4406013060602 | 1 | .0 | .0 | 97.6 |
|  | 4406013068501 | 1 | .0 | .0 | 97.6 |
|  | 4406014001801 | 1 | .0 | .0 | 97.6 |
|  | 4406014002503 | 1 | .0 | .0 | 97.6 |
|  | 4406014002505 | 1 | .0 | .0 | 97.6 |
|  | 4406014002802 | 1 | .0 | .0 | 97.6 |
|  | 4406014005604 | 1 | .0 | .0 | 97.6 |
|  | 4406014005704 | 1 | .0 | .0 | 97.6 |
|  | 4406014007501 | 1 | .0 | .0 | 97.6 |
|  | 4406014010602 | 1 | .0 | .0 | 97.6 |
|  | 4406014011602 | 1 | .0 | .0 | 97.6 |
|  | 4406014012108 | 1 | .0 | .0 | 97.6 |
|  | 4406014012701 | 1 | .0 | .0 | 97.7 |
|  | 4406014013203 | 1 | .0 | .0 | 97.7 |
|  | 4406015001601 | 1 | .0 | .0 | 97.7 |
|  | 4406015002602 | 1 | .0 | .0 | 97.7 |
|  | 4406015002604 | 1 | .0 | .0 | 97.7 |
|  | 4406015003002 | 1 | .0 | .0 | 97.7 |
|  | 4406015003307 | 1 | .0 | .0 | 97.7 |
|  | 4406015003803 | 1 | .0 | .0 | 97.7 |
|  | 4406015004202 | 1 | .0 | .0 | 97.7 |
|  | 4406015005401 | 1 | .0 | .0 | 97.7 |
|  | 4406015006202 | 1 | .0 | .0 | 97.7 |
|  | 4406015006506 | 1 | .0 | .0 | 97.7 |
|  | 4406015013203 | 1 | .0 | .0 | 97.7 |
|  | 4406015014002 | 1 | .0 | .0 | 97.7 |
|  | 4406015030003 | 1 | .0 | .0 | 97.7 |
|  | 4406015034901 | 1 | .0 | .0 | 97.7 |
|  | 4406015039202 | 1 | .0 | .0 | 97.7 |
|  | 4406015044703 | 1 | .0 | .0 | 97.7 |
|  | 4406015049303 | 1 | .0 | .0 | 97.7 |
|  | 4406015066301 | 1 | .0 | .0 | 97.7 |
|  | 4406015069501 | 1 | .0 | .0 | 97.7 |
|  | 4406015071005 | 1 | .0 | .0 | 97.7 |
|  | 4406015071006 | 1 | .0 | .0 | 97.7 |
|  | 4406016004501 | 1 | .0 | .0 | 97.7 |
|  | 4406017005302 | 1 | .0 | .0 | 97.7 |
|  | 4406017009004 | 1 | .0 | .0 | 97.7 |
|  | 4406017009202 | 1 | .0 | .0 | 97.7 |
|  | 4406018002801 | 1 | .0 | .0 | 97.7 |
|  | 4406019005901 | 1 | .0 | .0 | 97.7 |
|  | 4406019007302 | 1 | .0 | .0 | 97.7 |
|  | 4406019009203 | 1 | .0 | .0 | 97.8 |
|  | 4406020000302 | 1 | .0 | .0 | 97.8 |
|  | 4406020003203 | 1 | .0 | .0 | 97.8 |
|  | 4406020006101 | 1 | .0 | .0 | 97.8 |
|  | 4406020006305 | 1 | .0 | .0 | 97.8 |
|  | 4406020014201 | 1 | .0 | .0 | 97.8 |
|  | 4406020019007 | 1 | .0 | .0 | 97.8 |
|  | 4406020023004 | 1 | .0 | .0 | 97.8 |
|  | 4406020023402 | 1 | .0 | .0 | 97.8 |
|  | 4406020025106 | 1 | .0 | .0 | 97.8 |
|  | 4406020027503 | 1 | .0 | .0 | 97.8 |
|  | 4406020028202 | 1 | .0 | .0 | 97.8 |
|  | 4406020031305 | 1 | .0 | .0 | 97.8 |
|  | 4406021002901 | 1 | .0 | .0 | 97.8 |
|  | 4406021003302 | 1 | .0 | .0 | 97.8 |
|  | 4406021005204 | 1 | .0 | .0 | 97.8 |
|  | 4406021005805 | 1 | .0 | .0 | 97.8 |
|  | 4406021007606 | 1 | .0 | .0 | 97.8 |
|  | 4406021008604 | 1 | .0 | .0 | 97.8 |
|  | 4406021009104 | 1 | .0 | .0 | 97.8 |
|  | 4406021009202 | 1 | .0 | .0 | 97.8 |
|  | 4406021010102 | 1 | .0 | .0 | 97.8 |
|  | 4406021011002 | 1 | .0 | .0 | 97.8 |
|  | 4406021011102 | 1 | .0 | .0 | 97.8 |
|  | 4406021011602 | 1 | .0 | .0 | 97.8 |
|  | 4406021012202 | 1 | .0 | .0 | 97.8 |
|  | 4406021013801 | 1 | .0 | .0 | 97.8 |
|  | 4406021013901 | 1 | .0 | .0 | 97.8 |
|  | 4406021014502 | 1 | .0 | .0 | 97.8 |
|  | 4406021016203 | 1 | .0 | .0 | 97.8 |
|  | 4406021025304 | 1 | .0 | .0 | 97.8 |
|  | 4406021029708 | 1 | .0 | .0 | 97.9 |
|  | 4406021030306 | 1 | .0 | .0 | 97.9 |
|  | 4406021032403 | 1 | .0 | .0 | 97.9 |
|  | 4406021033805 | 1 | .0 | .0 | 97.9 |
|  | 4406021036701 | 1 | .0 | .0 | 97.9 |
|  | 4406021039806 | 1 | .0 | .0 | 97.9 |
|  | 4406021042704 | 1 | .0 | .0 | 97.9 |
|  | 4406023000103 | 1 | .0 | .0 | 97.9 |
|  | 4406023000403 | 1 | .0 | .0 | 97.9 |
|  | 4406023017605 | 1 | .0 | .0 | 97.9 |
|  | 4406023020001 | 1 | .0 | .0 | 97.9 |
|  | 4406024000204 | 1 | .0 | .0 | 97.9 |
|  | 4406024002003 | 1 | .0 | .0 | 97.9 |
|  | 4406024004602 | 1 | .0 | .0 | 97.9 |
|  | 4406024004907 | 1 | .0 | .0 | 97.9 |
|  | 4406025001002 | 1 | .0 | .0 | 97.9 |
|  | 4406027004401 | 1 | .0 | .0 | 97.9 |
|  | 4406027008303 | 1 | .0 | .0 | 97.9 |
|  | 4406027009001 | 1 | .0 | .0 | 97.9 |
|  | 4406027009901 | 1 | .0 | .0 | 97.9 |
|  | 4406028004204 | 1 | .0 | .0 | 97.9 |
|  | 4406028008201 | 1 | .0 | .0 | 97.9 |
|  | 4406028008801 | 1 | .0 | .0 | 97.9 |
|  | 4406029000903 | 1 | .0 | .0 | 97.9 |
|  | 4406029001402 | 1 | .0 | .0 | 97.9 |
|  | 4406029001702 | 1 | .0 | .0 | 97.9 |
|  | 4406029005503 | 1 | .0 | .0 | 97.9 |
|  | 4406029007502 | 1 | .0 | .0 | 97.9 |
|  | 4406029007602 | 1 | .0 | .0 | 97.9 |
|  | 4406029013703 | 1 | .0 | .0 | 97.9 |
|  | 4406029017301 | 1 | .0 | .0 | 98.0 |
|  | 4406029021002 | 1 | .0 | .0 | 98.0 |
|  | 4406029022606 | 1 | .0 | .0 | 98.0 |
|  | 4406030000901 | 1 | .0 | .0 | 98.0 |
|  | 4406030008201 | 1 | .0 | .0 | 98.0 |
|  | 4406030013701 | 1 | .0 | .0 | 98.0 |
|  | 4406030020402 | 1 | .0 | .0 | 98.0 |
|  | 4406030023901 | 1 | .0 | .0 | 98.0 |
|  | 4406030025402 | 1 | .0 | .0 | 98.0 |
|  | 4406030033508 | 1 | .0 | .0 | 98.0 |
|  | 4406030038501 | 1 | .0 | .0 | 98.0 |
|  | 4406031001904 | 1 | .0 | .0 | 98.0 |
|  | 4406031008602 | 1 | .0 | .0 | 98.0 |
|  | 4406031013304 | 1 | .0 | .0 | 98.0 |
|  | 4406031014603 | 1 | .0 | .0 | 98.0 |
|  | 4406031017101 | 1 | .0 | .0 | 98.0 |
|  | 4406032002905 | 1 | .0 | .0 | 98.0 |
|  | 4406032003703 | 1 | .0 | .0 | 98.0 |
|  | 4406032003807 | 1 | .0 | .0 | 98.0 |
|  | 5501001016004 | 1 | .0 | .0 | 98.0 |
|  | 5501001024901 | 1 | .0 | .0 | 98.0 |
|  | 5501002010802 | 1 | .0 | .0 | 98.0 |
|  | 5501002011901 | 1 | .0 | .0 | 98.0 |
|  | 5501002028601 | 1 | .0 | .0 | 98.0 |
|  | 5501002031102 | 1 | .0 | .0 | 98.0 |
|  | 5501002036404 | 1 | .0 | .0 | 98.0 |
|  | 5501002044801 | 1 | .0 | .0 | 98.0 |
|  | 5501004009204 | 1 | .0 | .0 | 98.0 |
|  | 5501004016401 | 1 | .0 | .0 | 98.0 |
|  | 5501004017206 | 1 | .0 | .0 | 98.0 |
|  | 5501007011302 | 1 | .0 | .0 | 98.0 |
|  | 5501007013203 | 1 | .0 | .0 | 98.1 |
|  | 5501007013604 | 1 | .0 | .0 | 98.1 |
|  | 5501008014802 | 1 | .0 | .0 | 98.1 |
|  | 5501008021804 | 1 | .0 | .0 | 98.1 |
|  | 5501008030404 | 1 | .0 | .0 | 98.1 |
|  | 5501008032604 | 1 | .0 | .0 | 98.1 |
|  | 5501008033106 | 1 | .0 | .0 | 98.1 |
|  | 5501008033502 | 1 | .0 | .0 | 98.1 |
|  | 5501008036002 | 1 | .0 | .0 | 98.1 |
|  | 5501008038707 | 1 | .0 | .0 | 98.1 |
|  | 5501009007303 | 1 | .0 | .0 | 98.1 |
|  | 5501009009201 | 1 | .0 | .0 | 98.1 |
|  | 5501009015903 | 1 | .0 | .0 | 98.1 |
|  | 5501009016901 | 1 | .0 | .0 | 98.1 |
|  | 5501011031901 | 1 | .0 | .0 | 98.1 |
|  | 5501011032301 | 1 | .0 | .0 | 98.1 |
|  | 5501011036701 | 1 | .0 | .0 | 98.1 |
|  | 5501011037003 | 1 | .0 | .0 | 98.1 |
|  | 5501011039004 | 1 | .0 | .0 | 98.1 |
|  | 5501012004502 | 1 | .0 | .0 | 98.1 |
|  | 5501012029502 | 1 | .0 | .0 | 98.1 |
|  | 5501013002601 | 1 | .0 | .0 | 98.1 |
|  | 5501013005606 | 1 | .0 | .0 | 98.1 |
|  | 5501014003705 | 1 | .0 | .0 | 98.1 |
|  | 5501014005701 | 1 | .0 | .0 | 98.1 |
|  | 5501014005801 | 1 | .0 | .0 | 98.1 |
|  | 5501014005802 | 1 | .0 | .0 | 98.1 |
|  | 5501014011201 | 1 | .0 | .0 | 98.1 |
|  | 5501014023503 | 1 | .0 | .0 | 98.1 |
|  | 5501014032901 | 1 | .0 | .0 | 98.1 |
|  | 5501014043402 | 1 | .0 | .0 | 98.2 |
|  | 5501014044402 | 1 | .0 | .0 | 98.2 |
|  | 5501015006404 | 1 | .0 | .0 | 98.2 |
|  | 5501015015106 | 1 | .0 | .0 | 98.2 |
|  | 5501016002504 | 1 | .0 | .0 | 98.2 |
|  | 5501016005702 | 1 | .0 | .0 | 98.2 |
|  | 5501016005903 | 1 | .0 | .0 | 98.2 |
|  | 5501017009002 | 1 | .0 | .0 | 98.2 |
|  | 5501017016901 | 1 | .0 | .0 | 98.2 |
|  | 5501017024605 | 1 | .0 | .0 | 98.2 |
|  | 5501017026503 | 1 | .0 | .0 | 98.2 |
|  | 5501018005204 | 1 | .0 | .0 | 98.2 |
|  | 5501018012603 | 1 | .0 | .0 | 98.2 |
|  | 5501018013202 | 1 | .0 | .0 | 98.2 |
|  | 5501018018702 | 1 | .0 | .0 | 98.2 |
|  | 5501018022502 | 1 | .0 | .0 | 98.2 |
|  | 5501018026101 | 1 | .0 | .0 | 98.2 |
|  | 5501018033102 | 1 | .0 | .0 | 98.2 |
|  | 5501018035005 | 1 | .0 | .0 | 98.2 |
|  | 5501018046603 | 1 | .0 | .0 | 98.2 |
|  | 5501018069303 | 1 | .0 | .0 | 98.2 |
|  | 5502004062802 | 1 | .0 | .0 | 98.2 |
|  | 5502004063201 | 1 | .0 | .0 | 98.2 |
|  | 5502005003803 | 1 | .0 | .0 | 98.2 |
|  | 5502006035303 | 1 | .0 | .0 | 98.2 |
|  | 5502007001103 | 1 | .0 | .0 | 98.2 |
|  | 5502007035702 | 1 | .0 | .0 | 98.2 |
|  | 5502007051801 | 1 | .0 | .0 | 98.2 |
|  | 5502007053002 | 1 | .0 | .0 | 98.2 |
|  | 5502007069002 | 1 | .0 | .0 | 98.2 |
|  | 5502007091303 | 1 | .0 | .0 | 98.2 |
|  | 5502007096302 | 1 | .0 | .0 | 98.3 |
|  | 5502007104901 | 1 | .0 | .0 | 98.3 |
|  | 5502007128401 | 1 | .0 | .0 | 98.3 |
|  | 5502007130701 | 1 | .0 | .0 | 98.3 |
|  | 5502008026403 | 1 | .0 | .0 | 98.3 |
|  | 5502009038606 | 1 | .0 | .0 | 98.3 |
|  | 5502011036302 | 1 | .0 | .0 | 98.3 |
|  | 5502011056906 | 1 | .0 | .0 | 98.3 |
|  | 5502011079702 | 1 | .0 | .0 | 98.3 |
|  | 5502011137405 | 1 | .0 | .0 | 98.3 |
|  | 5502011147803 | 1 | .0 | .0 | 98.3 |
|  | 5502011148902 | 1 | .0 | .0 | 98.3 |
|  | 5502011153402 | 1 | .0 | .0 | 98.3 |
|  | 5502012010404 | 1 | .0 | .0 | 98.3 |
|  | 5502012039802 | 1 | .0 | .0 | 98.3 |
|  | 5502012042201 | 1 | .0 | .0 | 98.3 |
|  | 5502012042601 | 1 | .0 | .0 | 98.3 |
|  | 5503001086404 | 1 | .0 | .0 | 98.3 |
|  | 5503002000404 | 1 | .0 | .0 | 98.3 |
|  | 5503002072308 | 1 | .0 | .0 | 98.3 |
|  | 5503003004003 | 1 | .0 | .0 | 98.3 |
|  | 5503003041502 | 1 | .0 | .0 | 98.3 |
|  | 5503003044004 | 1 | .0 | .0 | 98.3 |
|  | 5503003047205 | 1 | .0 | .0 | 98.3 |
|  | 5503003073601 | 1 | .0 | .0 | 98.3 |
|  | 5503003082201 | 1 | .0 | .0 | 98.3 |
|  | 5503003100503 | 1 | .0 | .0 | 98.3 |
|  | 5503003120801 | 1 | .0 | .0 | 98.3 |
|  | 5503003121004 | 1 | .0 | .0 | 98.3 |
|  | 5503003125302 | 1 | .0 | .0 | 98.3 |
|  | 5503003132504 | 1 | .0 | .0 | 98.4 |
|  | 5503003154103 | 1 | .0 | .0 | 98.4 |
|  | 5503005005301 | 1 | .0 | .0 | 98.4 |
|  | 5503005016904 | 1 | .0 | .0 | 98.4 |
|  | 5503005026304 | 1 | .0 | .0 | 98.4 |
|  | 5503005035004 | 1 | .0 | .0 | 98.4 |
|  | 5503005035104 | 1 | .0 | .0 | 98.4 |
|  | 5503005043102 | 1 | .0 | .0 | 98.4 |
|  | 5503005050401 | 1 | .0 | .0 | 98.4 |
|  | 5503005054204 | 1 | .0 | .0 | 98.4 |
|  | 5503005073402 | 1 | .0 | .0 | 98.4 |
|  | 5503005104201 | 1 | .0 | .0 | 98.4 |
|  | 5503006007906 | 1 | .0 | .0 | 98.4 |
|  | 5503006017706 | 1 | .0 | .0 | 98.4 |
|  | 5503006062303 | 1 | .0 | .0 | 98.4 |
|  | 5503009015501 | 1 | .0 | .0 | 98.4 |
|  | 5503009032101 | 1 | .0 | .0 | 98.4 |
|  | 5503009045401 | 1 | .0 | .0 | 98.4 |
|  | 5504001004203 | 1 | .0 | .0 | 98.4 |
|  | 5504001005002 | 1 | .0 | .0 | 98.4 |
|  | 5504002007602 | 1 | .0 | .0 | 98.4 |
|  | 5504002009301 | 1 | .0 | .0 | 98.4 |
|  | 5504002018303 | 1 | .0 | .0 | 98.4 |
|  | 5504002018702 | 1 | .0 | .0 | 98.4 |
|  | 5504002020004 | 1 | .0 | .0 | 98.4 |
|  | 5504002067701 | 1 | .0 | .0 | 98.4 |
|  | 5504003010601 | 1 | .0 | .0 | 98.4 |
|  | 5504003012204 | 1 | .0 | .0 | 98.4 |
|  | 5504004001402 | 1 | .0 | .0 | 98.4 |
|  | 5504005000403 | 1 | .0 | .0 | 98.4 |
|  | 5504005002604 | 1 | .0 | .0 | 98.4 |
|  | 5504005006502 | 1 | .0 | .0 | 98.5 |
|  | 5504005008502 | 1 | .0 | .0 | 98.5 |
|  | 5504005010402 | 1 | .0 | .0 | 98.5 |
|  | 5504005030501 | 1 | .0 | .0 | 98.5 |
|  | 5504009016202 | 1 | .0 | .0 | 98.5 |
|  | 5504009016602 | 1 | .0 | .0 | 98.5 |
|  | 5504009025301 | 1 | .0 | .0 | 98.5 |
|  | 5504009029202 | 1 | .0 | .0 | 98.5 |
|  | 5504009030905 | 1 | .0 | .0 | 98.5 |
|  | 5504009053304 | 1 | .0 | .0 | 98.5 |
|  | 5504009058501 | 1 | .0 | .0 | 98.5 |
|  | 5504010000106 | 1 | .0 | .0 | 98.5 |
|  | 5504010004503 | 1 | .0 | .0 | 98.5 |
|  | 5504010033401 | 1 | .0 | .0 | 98.5 |
|  | 5504011019301 | 1 | .0 | .0 | 98.5 |
|  | 5504011063503 | 1 | .0 | .0 | 98.5 |
|  | 5504011070202 | 1 | .0 | .0 | 98.5 |
|  | 5504011071003 | 1 | .0 | .0 | 98.5 |
|  | 5504011071502 | 1 | .0 | .0 | 98.5 |
|  | 5504012020901 | 1 | .0 | .0 | 98.5 |
|  | 5505001064603 | 1 | .0 | .0 | 98.5 |
|  | 5505004008902 | 1 | .0 | .0 | 98.5 |
|  | 5505004042103 | 1 | .0 | .0 | 98.5 |
|  | 5505006043502 | 1 | .0 | .0 | 98.5 |
|  | 5505006139205 | 1 | .0 | .0 | 98.5 |
|  | 5505006142904 | 1 | .0 | .0 | 98.5 |
|  | 5505006179301 | 1 | .0 | .0 | 98.5 |
|  | 5505006238402 | 1 | .0 | .0 | 98.5 |
|  | 5505008008602 | 1 | .0 | .0 | 98.5 |
|  | 5505008045102 | 1 | .0 | .0 | 98.5 |
|  | 5505009073901 | 1 | .0 | .0 | 98.6 |
|  | 5505010065301 | 1 | .0 | .0 | 98.6 |
|  | 5505010072202 | 1 | .0 | .0 | 98.6 |
|  | 5506001013603 | 1 | .0 | .0 | 98.6 |
|  | 5506001018202 | 1 | .0 | .0 | 98.6 |
|  | 5506001043702 | 1 | .0 | .0 | 98.6 |
|  | 5506002009605 | 1 | .0 | .0 | 98.6 |
|  | 5506002010402 | 1 | .0 | .0 | 98.6 |
|  | 5506002025905 | 1 | .0 | .0 | 98.6 |
|  | 5506002035804 | 1 | .0 | .0 | 98.6 |
|  | 5506002036302 | 1 | .0 | .0 | 98.6 |
|  | 5506002047901 | 1 | .0 | .0 | 98.6 |
|  | 5506003005201 | 1 | .0 | .0 | 98.6 |
|  | 5506003005303 | 1 | .0 | .0 | 98.6 |
|  | 5506003005402 | 1 | .0 | .0 | 98.6 |
|  | 5506003006604 | 1 | .0 | .0 | 98.6 |
|  | 5506003011603 | 1 | .0 | .0 | 98.6 |
|  | 5506003012001 | 1 | .0 | .0 | 98.6 |
|  | 5506003012202 | 1 | .0 | .0 | 98.6 |
|  | 5506003018201 | 1 | .0 | .0 | 98.6 |
|  | 5506005007002 | 1 | .0 | .0 | 98.6 |
|  | 5506005016905 | 1 | .0 | .0 | 98.6 |
|  | 5506005019601 | 1 | .0 | .0 | 98.6 |
|  | 5506005034002 | 1 | .0 | .0 | 98.6 |
|  | 5506006006701 | 1 | .0 | .0 | 98.6 |
|  | 5506006021401 | 1 | .0 | .0 | 98.6 |
|  | 5506007016401 | 1 | .0 | .0 | 98.6 |
|  | 5506007016703 | 1 | .0 | .0 | 98.6 |
|  | 5506007017105 | 1 | .0 | .0 | 98.6 |
|  | 5506007017208 | 1 | .0 | .0 | 98.6 |
|  | 5506007017702 | 1 | .0 | .0 | 98.6 |
|  | 5506008018203 | 1 | .0 | .0 | 98.7 |
|  | 5506008022901 | 1 | .0 | .0 | 98.7 |
|  | 5506008037701 | 1 | .0 | .0 | 98.7 |
|  | 5506008041701 | 1 | .0 | .0 | 98.7 |
|  | 5506008051505 | 1 | .0 | .0 | 98.7 |
|  | 5506008077902 | 1 | .0 | .0 | 98.7 |
|  | 5506009032807 | 1 | .0 | .0 | 98.7 |
|  | 5506009057003 | 1 | .0 | .0 | 98.7 |
|  | 5506009067002 | 1 | .0 | .0 | 98.7 |
|  | 5506009079102 | 1 | .0 | .0 | 98.7 |
|  | 5506009082203 | 1 | .0 | .0 | 98.7 |
|  | 5506009082905 | 1 | .0 | .0 | 98.7 |
|  | 5506010026102 | 1 | .0 | .0 | 98.7 |
|  | 5506010049403 | 1 | .0 | .0 | 98.7 |
|  | 5506010074202 | 1 | .0 | .0 | 98.7 |
|  | 5506010078904 | 1 | .0 | .0 | 98.7 |
|  | 5506011006203 | 1 | .0 | .0 | 98.7 |
|  | 5506011009202 | 1 | .0 | .0 | 98.7 |
|  | 5506011028603 | 1 | .0 | .0 | 98.7 |
|  | 5506011032805 | 1 | .0 | .0 | 98.7 |
|  | 5506012002602 | 1 | .0 | .0 | 98.7 |
|  | 5506012013401 | 1 | .0 | .0 | 98.7 |
|  | 5506012014102 | 1 | .0 | .0 | 98.7 |
|  | 5506012020501 | 1 | .0 | .0 | 98.7 |
|  | 5506012022102 | 1 | .0 | .0 | 98.7 |
|  | 5506014000602 | 1 | .0 | .0 | 98.7 |
|  | 5506014002002 | 1 | .0 | .0 | 98.7 |
|  | 5506014002208 | 1 | .0 | .0 | 98.7 |
|  | 5506014003004 | 1 | .0 | .0 | 98.7 |
|  | 5506014004002 | 1 | .0 | .0 | 98.7 |
|  | 5506014007602 | 1 | .0 | .0 | 98.7 |
|  | 5506014010404 | 1 | .0 | .0 | 98.8 |
|  | 5506014013101 | 1 | .0 | .0 | 98.8 |
|  | 5506014017403 | 1 | .0 | .0 | 98.8 |
|  | 5506014030404 | 1 | .0 | .0 | 98.8 |
|  | 5506014030601 | 1 | .0 | .0 | 98.8 |
|  | 5506014035803 | 1 | .0 | .0 | 98.8 |
|  | 5506014038701 | 1 | .0 | .0 | 98.8 |
|  | 5506014039001 | 1 | .0 | .0 | 98.8 |
|  | 5506014039902 | 1 | .0 | .0 | 98.8 |
|  | 5506014057403 | 1 | .0 | .0 | 98.8 |
|  | 5506014057501 | 1 | .0 | .0 | 98.8 |
|  | 5506015006701 | 1 | .0 | .0 | 98.8 |
|  | 5506015023403 | 1 | .0 | .0 | 98.8 |
|  | 5506016002601 | 1 | .0 | .0 | 98.8 |
|  | 5506017002502 | 1 | .0 | .0 | 98.8 |
|  | 5506017007301 | 1 | .0 | .0 | 98.8 |
|  | 5506017008001 | 1 | .0 | .0 | 98.8 |
|  | 5506017013302 | 1 | .0 | .0 | 98.8 |
|  | 5506017019901 | 1 | .0 | .0 | 98.8 |
|  | 5506017021903 | 1 | .0 | .0 | 98.8 |
|  | 5506018012101 | 1 | .0 | .0 | 98.8 |
|  | 5507003004202 | 1 | .0 | .0 | 98.8 |
|  | 5507003022203 | 1 | .0 | .0 | 98.8 |
|  | 5507003023004 | 1 | .0 | .0 | 98.8 |
|  | 5507004007501 | 1 | .0 | .0 | 98.8 |
|  | 5507006021103 | 1 | .0 | .0 | 98.8 |
|  | 5507007001601 | 1 | .0 | .0 | 98.8 |
|  | 5507007050102 | 1 | .0 | .0 | 98.8 |
|  | 5507007055701 | 1 | .0 | .0 | 98.8 |
|  | 5507008057701 | 1 | .0 | .0 | 98.8 |
|  | 5507008064202 | 1 | .0 | .0 | 98.9 |
|  | 5507009008701 | 1 | .0 | .0 | 98.9 |
|  | 5507010009703 | 1 | .0 | .0 | 98.9 |
|  | 5507010011401 | 1 | .0 | .0 | 98.9 |
|  | 5507010017903 | 1 | .0 | .0 | 98.9 |
|  | 5507010031801 | 1 | .0 | .0 | 98.9 |
|  | 5507010039403 | 1 | .0 | .0 | 98.9 |
|  | 5507010045201 | 1 | .0 | .0 | 98.9 |
|  | 5507010060102 | 1 | .0 | .0 | 98.9 |
|  | 5507010072204 | 1 | .0 | .0 | 98.9 |
|  | 5507010088202 | 1 | .0 | .0 | 98.9 |
|  | 5507010100501 | 1 | .0 | .0 | 98.9 |
|  | 5507010111104 | 1 | .0 | .0 | 98.9 |
|  | 5507010123402 | 1 | .0 | .0 | 98.9 |
|  | 5507010149103 | 1 | .0 | .0 | 98.9 |
|  | 5507010152202 | 1 | .0 | .0 | 98.9 |
|  | 5507010156202 | 1 | .0 | .0 | 98.9 |
|  | 5507010160504 | 1 | .0 | .0 | 98.9 |
|  | 5507010165103 | 1 | .0 | .0 | 98.9 |
|  | 5507010177604 | 1 | .0 | .0 | 98.9 |
|  | 5507010189905 | 1 | .0 | .0 | 98.9 |
|  | 5507010196604 | 1 | .0 | .0 | 98.9 |
|  | 5507010197601 | 1 | .0 | .0 | 98.9 |
|  | 5507010225801 | 1 | .0 | .0 | 98.9 |
|  | 5507010228303 | 1 | .0 | .0 | 98.9 |
|  | 5507010239602 | 1 | .0 | .0 | 98.9 |
|  | 5507010241602 | 1 | .0 | .0 | 98.9 |
|  | 5507010245602 | 1 | .0 | .0 | 98.9 |
|  | 5507010261102 | 1 | .0 | .0 | 98.9 |
|  | 5507010263102 | 1 | .0 | .0 | 98.9 |
|  | 5507010263202 | 1 | .0 | .0 | 98.9 |
|  | 5507010270102 | 1 | .0 | .0 | 99.0 |
|  | 5507010283603 | 1 | .0 | .0 | 99.0 |
|  | 5507010302401 | 1 | .0 | .0 | 99.0 |
|  | 5507010306604 | 1 | .0 | .0 | 99.0 |
|  | 5507010307601 | 1 | .0 | .0 | 99.0 |
|  | 5507010309204 | 1 | .0 | .0 | 99.0 |
|  | 5507010310405 | 1 | .0 | .0 | 99.0 |
|  | 5507010312002 | 1 | .0 | .0 | 99.0 |
|  | 5507010338201 | 1 | .0 | .0 | 99.0 |
|  | 5507010355702 | 1 | .0 | .0 | 99.0 |
|  | 5507010356102 | 1 | .0 | .0 | 99.0 |
|  | 5507010357001 | 1 | .0 | .0 | 99.0 |
|  | 5507010357801 | 1 | .0 | .0 | 99.0 |
|  | 5507012003203 | 1 | .0 | .0 | 99.0 |
|  | 5507012014002 | 1 | .0 | .0 | 99.0 |
|  | 5507012027405 | 1 | .0 | .0 | 99.0 |
|  | 5507012077006 | 1 | .0 | .0 | 99.0 |
|  | 5507012129706 | 1 | .0 | .0 | 99.0 |
|  | 5507013005203 | 1 | .0 | .0 | 99.0 |
|  | 5507013021302 | 1 | .0 | .0 | 99.0 |
|  | 5507013023202 | 1 | .0 | .0 | 99.0 |
|  | 6601001001803 | 1 | .0 | .0 | 99.0 |
|  | 6601001008002 | 1 | .0 | .0 | 99.0 |
|  | 6601001009904 | 1 | .0 | .0 | 99.0 |
|  | 6601001050203 | 1 | .0 | .0 | 99.0 |
|  | 6601001050601 | 1 | .0 | .0 | 99.0 |
|  | 6601001092304 | 1 | .0 | .0 | 99.0 |
|  | 6601002004403 | 1 | .0 | .0 | 99.0 |
|  | 6601002035904 | 1 | .0 | .0 | 99.0 |
|  | 6601002043702 | 1 | .0 | .0 | 99.0 |
|  | 6601002047104 | 1 | .0 | .0 | 99.1 |
|  | 6601003012601 | 1 | .0 | .0 | 99.1 |
|  | 6601003030001 | 1 | .0 | .0 | 99.1 |
|  | 6601003033503 | 1 | .0 | .0 | 99.1 |
|  | 6601005026401 | 1 | .0 | .0 | 99.1 |
|  | 6601005042301 | 1 | .0 | .0 | 99.1 |
|  | 6601005081403 | 1 | .0 | .0 | 99.1 |
|  | 6601009018602 | 1 | .0 | .0 | 99.1 |
|  | 6601016002302 | 1 | .0 | .0 | 99.1 |
|  | 6601017000205 | 1 | .0 | .0 | 99.1 |
|  | 6601017004302 | 1 | .0 | .0 | 99.1 |
|  | 6601017006701 | 1 | .0 | .0 | 99.1 |
|  | 6601018000604 | 1 | .0 | .0 | 99.1 |
|  | 6601018015404 | 1 | .0 | .0 | 99.1 |
|  | 6601019000602 | 1 | .0 | .0 | 99.1 |
|  | 6601019001402 | 1 | .0 | .0 | 99.1 |
|  | 6601019006502 | 2 | .0 | .0 | 99.1 |
|  | 6601019015402 | 1 | .0 | .0 | 99.1 |
|  | 6601019025102 | 1 | .0 | .0 | 99.1 |
|  | 6601019026301 | 1 | .0 | .0 | 99.1 |
|  | 6601019026601 | 1 | .0 | .0 | 99.1 |
|  | 6601019028202 | 1 | .0 | .0 | 99.1 |
|  | 6601019031102 | 1 | .0 | .0 | 99.1 |
|  | 6601019031304 | 1 | .0 | .0 | 99.1 |
|  | 6601020002801 | 1 | .0 | .0 | 99.1 |
|  | 6602001013401 | 1 | .0 | .0 | 99.1 |
|  | 6602001014701 | 1 | .0 | .0 | 99.1 |
|  | 6602001035201 | 1 | .0 | .0 | 99.1 |
|  | 6602001040001 | 1 | .0 | .0 | 99.1 |
|  | 6602001045701 | 1 | .0 | .0 | 99.1 |
|  | 6602001049101 | 1 | .0 | .0 | 99.2 |
|  | 6602001049308 | 1 | .0 | .0 | 99.2 |
|  | 6602003003403 | 1 | .0 | .0 | 99.2 |
|  | 6602004010503 | 1 | .0 | .0 | 99.2 |
|  | 6602004013805 | 1 | .0 | .0 | 99.2 |
|  | 6602004017403 | 1 | .0 | .0 | 99.2 |
|  | 6602004017502 | 1 | .0 | .0 | 99.2 |
|  | 6602004023901 | 1 | .0 | .0 | 99.2 |
|  | 6602004030702 | 1 | .0 | .0 | 99.2 |
|  | 6602004040602 | 1 | .0 | .0 | 99.2 |
|  | 6602005019907 | 1 | .0 | .0 | 99.2 |
|  | 6602005029502 | 1 | .0 | .0 | 99.2 |
|  | 6602005044007 | 1 | .0 | .0 | 99.2 |
|  | 6602006029003 | 1 | .0 | .0 | 99.2 |
|  | 6602007000303 | 1 | .0 | .0 | 99.2 |
|  | 6602007048402 | 1 | .0 | .0 | 99.2 |
|  | 6602007050706 | 1 | .0 | .0 | 99.2 |
|  | 6602008010201 | 1 | .0 | .0 | 99.2 |
|  | 6602009004705 | 1 | .0 | .0 | 99.2 |
|  | 6602009006702 | 1 | .0 | .0 | 99.2 |
|  | 6602009006802 | 1 | .0 | .0 | 99.2 |
|  | 6602009008001 | 1 | .0 | .0 | 99.2 |
|  | 6602009019103 | 1 | .0 | .0 | 99.2 |
|  | 6602009025402 | 1 | .0 | .0 | 99.2 |
|  | 6602009025607 | 1 | .0 | .0 | 99.2 |
|  | 6602009116204 | 1 | .0 | .0 | 99.2 |
|  | 6602009144201 | 1 | .0 | .0 | 99.2 |
|  | 6602009150205 | 1 | .0 | .0 | 99.2 |
|  | 6602009150304 | 1 | .0 | .0 | 99.2 |
|  | 6602009154202 | 1 | .0 | .0 | 99.2 |
|  | 6602009155502 | 1 | .0 | .0 | 99.3 |
|  | 6602010019102 | 1 | .0 | .0 | 99.3 |
|  | 6602010021205 | 1 | .0 | .0 | 99.3 |
|  | 6602011000805 | 1 | .0 | .0 | 99.3 |
|  | 6602011010205 | 1 | .0 | .0 | 99.3 |
|  | 6603001003001 | 1 | .0 | .0 | 99.3 |
|  | 6603001005403 | 1 | .0 | .0 | 99.3 |
|  | 6603002010501 | 1 | .0 | .0 | 99.3 |
|  | 6603003018005 | 1 | .0 | .0 | 99.3 |
|  | 6603003019202 | 1 | .0 | .0 | 99.3 |
|  | 6603003026006 | 1 | .0 | .0 | 99.3 |
|  | 6603004009401 | 1 | .0 | .0 | 99.3 |
|  | 6603005010201 | 1 | .0 | .0 | 99.3 |
|  | 6603005012201 | 1 | .0 | .0 | 99.3 |
|  | 6603006007501 | 1 | .0 | .0 | 99.3 |
|  | 6603006007601 | 1 | .0 | .0 | 99.3 |
|  | 6603006010302 | 1 | .0 | .0 | 99.3 |
|  | 6603006010801 | 1 | .0 | .0 | 99.3 |
|  | 6603006016804 | 1 | .0 | .0 | 99.3 |
|  | 6603006021802 | 1 | .0 | .0 | 99.3 |
|  | 6603006023604 | 1 | .0 | .0 | 99.3 |
|  | 6603006026401 | 1 | .0 | .0 | 99.3 |
|  | 6603006029801 | 1 | .0 | .0 | 99.3 |
|  | 6603006030201 | 1 | .0 | .0 | 99.3 |
|  | 6603006032302 | 1 | .0 | .0 | 99.3 |
|  | 6603006042705 | 1 | .0 | .0 | 99.3 |
|  | 6603006044201 | 1 | .0 | .0 | 99.3 |
|  | 6603006045005 | 1 | .0 | .0 | 99.3 |
|  | 6603006045902 | 1 | .0 | .0 | 99.3 |
|  | 6603006052702 | 1 | .0 | .0 | 99.3 |
|  | 6603006059701 | 1 | .0 | .0 | 99.3 |
|  | 6603006070503 | 1 | .0 | .0 | 99.4 |
|  | 6603007005501 | 1 | .0 | .0 | 99.4 |
|  | 6603008030704 | 1 | .0 | .0 | 99.4 |
|  | 6603008050903 | 1 | .0 | .0 | 99.4 |
|  | 6603008056206 | 1 | .0 | .0 | 99.4 |
|  | 6603009003301 | 1 | .0 | .0 | 99.4 |
|  | 6603009010702 | 1 | .0 | .0 | 99.4 |
|  | 6603009019802 | 1 | .0 | .0 | 99.4 |
|  | 6603010011102 | 1 | .0 | .0 | 99.4 |
|  | 6603010012703 | 1 | .0 | .0 | 99.4 |
|  | 6603010028601 | 1 | .0 | .0 | 99.4 |
|  | 6603010039801 | 1 | .0 | .0 | 99.4 |
|  | 6603010059801 | 1 | .0 | .0 | 99.4 |
|  | 6603010061404 | 1 | .0 | .0 | 99.4 |
|  | 6603010061704 | 1 | .0 | .0 | 99.4 |
|  | 6603011000503 | 1 | .0 | .0 | 99.4 |
|  | 6603011013901 | 1 | .0 | .0 | 99.4 |
|  | 6603011019902 | 1 | .0 | .0 | 99.4 |
|  | 6603011026003 | 1 | .0 | .0 | 99.4 |
|  | 6603012013902 | 1 | .0 | .0 | 99.4 |
|  | 6603012021401 | 1 | .0 | .0 | 99.4 |
|  | 6603013016004 | 1 | .0 | .0 | 99.4 |
|  | 6603014002002 | 1 | .0 | .0 | 99.4 |
|  | 6603017000803 | 1 | .0 | .0 | 99.4 |
|  | 6603017031001 | 1 | .0 | .0 | 99.4 |
|  | 6603017033302 | 1 | .0 | .0 | 99.4 |
|  | 6604001012701 | 1 | .0 | .0 | 99.4 |
|  | 6604001026201 | 1 | .0 | .0 | 99.4 |
|  | 6604001048901 | 1 | .0 | .0 | 99.4 |
|  | 6604001062501 | 1 | .0 | .0 | 99.4 |
|  | 6604001071101 | 1 | .0 | .0 | 99.5 |
|  | 6604001073901 | 1 | .0 | .0 | 99.5 |
|  | 6604001077803 | 1 | .0 | .0 | 99.5 |
|  | 6604001079701 | 1 | .0 | .0 | 99.5 |
|  | 6604001086101 | 1 | .0 | .0 | 99.5 |
|  | 6604001106304 | 1 | .0 | .0 | 99.5 |
|  | 6604001152303 | 1 | .0 | .0 | 99.5 |
|  | 6604001156201 | 1 | .0 | .0 | 99.5 |
|  | 6604002000801 | 1 | .0 | .0 | 99.5 |
|  | 6604002014502 | 1 | .0 | .0 | 99.5 |
|  | 6604002035701 | 1 | .0 | .0 | 99.5 |
|  | 6604003011901 | 1 | .0 | .0 | 99.5 |
|  | 6604004009305 | 1 | .0 | .0 | 99.5 |
|  | 6604004012001 | 1 | .0 | .0 | 99.5 |
|  | 6604004035605 | 1 | .0 | .0 | 99.5 |
|  | 6604005014101 | 1 | .0 | .0 | 99.5 |
|  | 6604005015305 | 1 | .0 | .0 | 99.5 |
|  | 6604005071904 | 1 | .0 | .0 | 99.5 |
|  | 6604005074810 | 1 | .0 | .0 | 99.5 |
|  | 6604005077003 | 1 | .0 | .0 | 99.5 |
|  | 6604005083302 | 1 | .0 | .0 | 99.5 |
|  | 6604005085105 | 1 | .0 | .0 | 99.5 |
|  | 6604005094404 | 1 | .0 | .0 | 99.5 |
|  | 6604005169802 | 1 | .0 | .0 | 99.5 |
|  | 6605002002404 | 1 | .0 | .0 | 99.5 |
|  | 6605002051804 | 1 | .0 | .0 | 99.5 |
|  | 6605002053204 | 1 | .0 | .0 | 99.5 |
|  | 6605002061501 | 1 | .0 | .0 | 99.5 |
|  | 6605002063201 | 1 | .0 | .0 | 99.5 |
|  | 6605002084303 | 1 | .0 | .0 | 99.5 |
|  | 6605006038401 | 1 | .0 | .0 | 99.5 |
|  | 6605007025806 | 1 | .0 | .0 | 99.6 |
|  | 6605007040201 | 1 | .0 | .0 | 99.6 |
|  | 6605007061201 | 1 | .0 | .0 | 99.6 |
|  | 6605007065404 | 1 | .0 | .0 | 99.6 |
|  | 6605007072403 | 1 | .0 | .0 | 99.6 |
|  | 6605007078801 | 1 | .0 | .0 | 99.6 |
|  | 6605007085601 | 1 | .0 | .0 | 99.6 |
|  | 6605007085801 | 1 | .0 | .0 | 99.6 |
|  | 6605007101402 | 1 | .0 | .0 | 99.6 |
|  | 6605007105702 | 1 | .0 | .0 | 99.6 |
|  | 6605007113302 | 1 | .0 | .0 | 99.6 |
|  | 6605007113803 | 1 | .0 | .0 | 99.6 |
|  | 6605007116205 | 1 | .0 | .0 | 99.6 |
|  | 6605008006906 | 1 | .0 | .0 | 99.6 |
|  | 6605009003907 | 1 | .0 | .0 | 99.6 |
|  | 6605009005601 | 1 | .0 | .0 | 99.6 |
|  | 6605009037603 | 1 | .0 | .0 | 99.6 |
|  | 6605009049803 | 1 | .0 | .0 | 99.6 |
|  | 6605009062705 | 1 | .0 | .0 | 99.6 |
|  | 6605010005404 | 1 | .0 | .0 | 99.6 |
|  | 6605011015101 | 1 | .0 | .0 | 99.6 |
|  | 6605011015501 | 1 | .0 | .0 | 99.6 |
|  | 6605013006404 | 1 | .0 | .0 | 99.6 |
|  | 6605013009603 | 1 | .0 | .0 | 99.6 |
|  | 6605013019302 | 1 | .0 | .0 | 99.6 |
|  | 6605013020005 | 1 | .0 | .0 | 99.6 |
|  | 6605013030801 | 1 | .0 | .0 | 99.6 |
|  | 6605013031301 | 1 | .0 | .0 | 99.6 |
|  | 6605013042401 | 1 | .0 | .0 | 99.6 |
|  | 6605013042504 | 1 | .0 | .0 | 99.6 |
|  | 6605013053905 | 1 | .0 | .0 | 99.6 |
|  | 6605013057301 | 1 | .0 | .0 | 99.7 |
|  | 6605013066402 | 1 | .0 | .0 | 99.7 |
|  | 6605013067903 | 1 | .0 | .0 | 99.7 |
|  | 6605013075001 | 1 | .0 | .0 | 99.7 |
|  | 6605014006605 | 1 | .0 | .0 | 99.7 |
|  | 6605014030603 | 1 | .0 | .0 | 99.7 |
|  | 6605014113903 | 1 | .0 | .0 | 99.7 |
|  | 6606001001404 | 1 | .0 | .0 | 99.7 |
|  | 6606001037404 | 1 | .0 | .0 | 99.7 |
|  | 6606001048301 | 1 | .0 | .0 | 99.7 |
|  | 6606001051701 | 1 | .0 | .0 | 99.7 |
|  | 6606001068101 | 1 | .0 | .0 | 99.7 |
|  | 6606001090902 | 1 | .0 | .0 | 99.7 |
|  | 6606001104101 | 1 | .0 | .0 | 99.7 |
|  | 6606001109601 | 1 | .0 | .0 | 99.7 |
|  | 6606001122802 | 1 | .0 | .0 | 99.7 |
|  | 6606001158203 | 1 | .0 | .0 | 99.7 |
|  | 6606001176402 | 1 | .0 | .0 | 99.7 |
|  | 6606001190005 | 1 | .0 | .0 | 99.7 |
|  | 6606001216001 | 1 | .0 | .0 | 99.7 |
|  | 6606001216502 | 1 | .0 | .0 | 99.7 |
|  | 6606001222301 | 1 | .0 | .0 | 99.7 |
|  | 6606001225203 | 1 | .0 | .0 | 99.7 |
|  | 6606001236803 | 1 | .0 | .0 | 99.7 |
|  | 6606001253901 | 1 | .0 | .0 | 99.7 |
|  | 6606001259106 | 1 | .0 | .0 | 99.7 |
|  | 6607001003015 | 1 | .0 | .0 | 99.7 |
|  | 6607003018005 | 1 | .0 | .0 | 99.7 |
|  | 6607003030001 | 1 | .0 | .0 | 99.7 |
|  | 6607003060604 | 1 | .0 | .0 | 99.7 |
|  | 6607004016702 | 1 | .0 | .0 | 99.8 |
|  | 6607008016501 | 1 | .0 | .0 | 99.8 |
|  | 6607008024602 | 1 | .0 | .0 | 99.8 |
|  | 6607013003202 | 1 | .0 | .0 | 99.8 |
|  | 6607013024004 | 1 | .0 | .0 | 99.8 |
|  | 6607014008202 | 1 | .0 | .0 | 99.8 |
|  | 6607014011903 | 1 | .0 | .0 | 99.8 |
|  | 6607019019602 | 1 | .0 | .0 | 99.8 |
|  | 6607019026003 | 1 | .0 | .0 | 99.8 |
|  | 6607019026402 | 1 | .0 | .0 | 99.8 |
|  | 6607021023403 | 1 | .0 | .0 | 99.8 |
|  | 6607021047801 | 1 | .0 | .0 | 99.8 |
|  | 6607022025403 | 1 | .0 | .0 | 99.8 |
|  | 6607023000601 | 1 | .0 | .0 | 99.8 |
|  | 6607023003304 | 1 | .0 | .0 | 99.8 |
|  | 6607023007804 | 1 | .0 | .0 | 99.8 |
|  | 6608002025501 | 1 | .0 | .0 | 99.8 |
|  | 6608002047401 | 1 | .0 | .0 | 99.8 |
|  | 6608002062402 | 1 | .0 | .0 | 99.8 |
|  | 6608004001702 | 1 | .0 | .0 | 99.8 |
|  | 6608005010102 | 1 | .0 | .0 | 99.8 |
|  | 6608005013001 | 1 | .0 | .0 | 99.8 |
|  | 6608005016201 | 1 | .0 | .0 | 99.8 |
|  | 6608005021802 | 1 | .0 | .0 | 99.8 |
|  | 6608006009903 | 1 | .0 | .0 | 99.8 |
|  | 6608008038201 | 1 | .0 | .0 | 99.8 |
|  | 6608008045102 | 1 | .0 | .0 | 99.8 |
|  | 6608009011807 | 1 | .0 | .0 | 99.8 |
|  | 6608009013403 | 1 | .0 | .0 | 99.8 |
|  | 6608009016202 | 1 | .0 | .0 | 99.8 |
|  | 6608011007303 | 1 | .0 | .0 | 99.8 |
|  | 6608011021603 | 1 | .0 | .0 | 99.9 |
|  | 6608011021701 | 1 | .0 | .0 | 99.9 |
|  | 6608011027204 | 1 | .0 | .0 | 99.9 |
|  | 6608011035103 | 1 | .0 | .0 | 99.9 |
|  | 6608011057206 | 1 | .0 | .0 | 99.9 |
|  | 6608013005403 | 1 | .0 | .0 | 99.9 |
|  | 6608013010305 | 1 | .0 | .0 | 99.9 |
|  | 6608013011003 | 1 | .0 | .0 | 99.9 |
|  | 6608014003902 | 1 | .0 | .0 | 99.9 |
|  | 6608014006905 | 1 | .0 | .0 | 99.9 |
|  | 6608015007302 | 1 | .0 | .0 | 99.9 |
|  | 6608015010504 | 1 | .0 | .0 | 99.9 |
|  | 6608015010602 | 1 | .0 | .0 | 99.9 |
|  | 6608016003601 | 1 | .0 | .0 | 99.9 |
|  | 6608017044404 | 1 | .0 | .0 | 99.9 |
|  | 6608017046101 | 1 | .0 | .0 | 99.9 |
|  | 6608017046201 | 1 | .0 | .0 | 99.9 |
|  | 6608017050904 | 1 | .0 | .0 | 99.9 |
|  | 6608017051402 | 1 | .0 | .0 | 99.9 |
|  | 6608017054905 | 1 | .0 | .0 | 99.9 |
|  | 6608017058601 | 1 | .0 | .0 | 99.9 |
|  | 6608018014304 | 1 | .0 | .0 | 99.9 |
|  | 6608018026602 | 1 | .0 | .0 | 99.9 |
|  | 6608018027002 | 1 | .0 | .0 | 99.9 |
|  | 6608019012904 | 1 | .0 | .0 | 99.9 |
|  | 6608022001402 | 1 | .0 | .0 | 99.9 |
|  | 6608023016402 | 1 | .0 | .0 | 99.9 |
|  | 6609001017804 | 1 | .0 | .0 | 99.9 |
|  | 6609001073503 | 1 | .0 | .0 | 99.9 |
|  | 6609001074701 | 1 | .0 | .0 | 99.9 |
|  | 6609002006302 | 1 | .0 | .0 | 100.0 |
|  | 6609003018304 | 1 | .0 | .0 | 100.0 |
|  | 6609003025202 | 1 | .0 | .0 | 100.0 |
|  | 6609003026701 | 1 | .0 | .0 | 100.0 |
|  | 6609003033004 | 1 | .0 | .0 | 100.0 |
|  | 6609003049801 | 1 | .0 | .0 | 100.0 |
|  | 6609003059001 | 1 | .0 | .0 | 100.0 |
|  | 6609003071901 | 1 | .0 | .0 | 100.0 |
|  | 6609005002301 | 1 | .0 | .0 | 100.0 |
|  | 6609005002601 | 1 | .0 | .0 | 100.0 |
|  | 6609005020906 | 1 | .0 | .0 | 100.0 |
|  | 6609005031002 | 1 | .0 | .0 | 100.0 |
|  | 6609005034804 | 1 | .0 | .0 | 100.0 |
|  | 6609005064902 | 1 | .0 | .0 | 100.0 |
|  | 6609005090201 | 1 | .0 | .0 | 100.0 |
|  | 6609005097703 | 1 | .0 | .0 | 100.0 |
|  | Total | 30553 | 100.0 | 100.0 |  |
|  |  |  |  |  |  |

Serial # of HH memberSerial # of HH member, table, 1 levels of column headers and 2 levels of row headers, table with 6 columns and 5 rows

|  |  |  |  |  |  |
| --- | --- | --- | --- | --- | --- |
|  | | Frequency | Percent | Valid Percent | Cumulative Percent |
| Valid |  | 29432 | 96.3 | 96.3 | 96.3 |
|  | InjID | 1121 | 3.7 | 3.7 | 100.0 |
|  | Total | 30553 | 100.0 | 100.0 |  |
|  |  |  |  |  |  |

Injury serial numberInjury serial number, table, 1 levels of column headers and 2 levels of row headers, table with 6 columns and 9 rows

|  |  |  |  |  |  |
| --- | --- | --- | --- | --- | --- |
|  | | Frequency | Percent | Valid Percent | Cumulative Percent |
| Valid |  | 29432 | 96.3 | 96.3 | 96.3 |
|  | 01 | 957 | 3.1 | 3.1 | 99.5 |
|  | 02 | 151 | .5 | .5 | 100.0 |
|  | 03 | 9 | .0 | .0 | 100.0 |
|  | 04 | 3 | .0 | .0 | 100.0 |
|  | 05 | 1 | .0 | .0 | 100.0 |
|  | Total | 30553 | 100.0 | 100.0 |  |
|  |  |  |  |  |  |

Injury eventInjury event, table, 1 levels of column headers and 2 levels of row headers, table with 6 columns and 5 rows

|  |  |  |  |  |  |
| --- | --- | --- | --- | --- | --- |
|  | | Frequency | Percent | Valid Percent | Cumulative Percent |
| Valid | 1 | 1121 | 3.7 | 100.0 | 100.0 |
| Missing | System | 29432 | 96.3 |  |  |
| Total | | 30553 | 100.0 |  |  |
|  |  |  |  |  |  |

District codeDistrict code, table, 1 levels of column headers and 2 levels of row headers, table with 6 columns and 8 rows

|  |  |  |  |  |  |
| --- | --- | --- | --- | --- | --- |
|  | | Frequency | Percent | Valid Percent | Cumulative Percent |
| Valid | Sirajganj | 513 | 1.7 | 45.8 | 45.8 |
|  | Sherpur | 308 | 1.0 | 27.5 | 73.2 |
|  | Narshindi | 300 | 1.0 | 26.8 | 100.0 |
|  | Total | 1121 | 3.7 | 100.0 |  |
| Missing | System | 29432 | 96.3 |  |  |
| Total | | 30553 | 100.0 |  |  |
|  |  |  |  |  |  |

Upazila codeUpazila code, table, 1 levels of column headers and 2 levels of row headers, table with 6 columns and 8 rows

|  |  |  |  |  |  |
| --- | --- | --- | --- | --- | --- |
|  | | Frequency | Percent | Valid Percent | Cumulative Percent |
| Valid | Raiganj | 513 | 1.7 | 45.8 | 45.8 |
|  | Sherpur Sadar | 308 | 1.0 | 27.5 | 73.2 |
|  | Manohardi | 300 | 1.0 | 26.8 | 100.0 |
|  | Total | 1121 | 3.7 | 100.0 |  |
| Missing | System | 29432 | 96.3 |  |  |
| Total | | 30553 | 100.0 |  |  |
|  |  |  |  |  |  |

Unions codeUnions code, table, 1 levels of column headers and 2 levels of row headers, table with 6 columns and 14 rows

|  |  |  |  |  |  |
| --- | --- | --- | --- | --- | --- |
|  | | Frequency | Percent | Valid Percent | Cumulative Percent |
| Valid | 1 | 98 | .3 | 8.7 | 8.7 |
|  | 2 | 67 | .2 | 6.0 | 14.7 |
|  | 3 | 83 | .3 | 7.4 | 22.1 |
|  | 4 | 212 | .7 | 18.9 | 41.0 |
|  | 5 | 141 | .5 | 12.6 | 53.6 |
|  | 6 | 378 | 1.2 | 33.7 | 87.3 |
|  | 7 | 81 | .3 | 7.2 | 94.6 |
|  | 8 | 42 | .1 | 3.7 | 98.3 |
|  | 9 | 19 | .1 | 1.7 | 100.0 |
|  | Total | 1121 | 3.7 | 100.0 |  |
| Missing | System | 29432 | 96.3 |  |  |
| Total | | 30553 | 100.0 |  |  |
|  |  |  |  |  |  |

Village codeVillage code, table, 1 levels of column headers and 2 levels of row headers, table with 6 columns and 36 rows

|  |  |  |  |  |  |
| --- | --- | --- | --- | --- | --- |
|  | | Frequency | Percent | Valid Percent | Cumulative Percent |
| Valid |  | 29432 | 96.3 | 96.3 | 96.3 |
|  | 001 | 92 | .3 | .3 | 96.6 |
|  | 002 | 86 | .3 | .3 | 96.9 |
|  | 003 | 48 | .2 | .2 | 97.1 |
|  | 004 | 34 | .1 | .1 | 97.2 |
|  | 005 | 75 | .2 | .2 | 97.4 |
|  | 006 | 38 | .1 | .1 | 97.6 |
|  | 007 | 71 | .2 | .2 | 97.8 |
|  | 008 | 43 | .1 | .1 | 97.9 |
|  | 009 | 51 | .2 | .2 | 98.1 |
|  | 010 | 94 | .3 | .3 | 98.4 |
|  | 011 | 67 | .2 | .2 | 98.6 |
|  | 012 | 42 | .1 | .1 | 98.8 |
|  | 013 | 79 | .3 | .3 | 99.0 |
|  | 014 | 62 | .2 | .2 | 99.2 |
|  | 015 | 33 | .1 | .1 | 99.3 |
|  | 016 | 9 | .0 | .0 | 99.4 |
|  | 017 | 31 | .1 | .1 | 99.5 |
|  | 018 | 37 | .1 | .1 | 99.6 |
|  | 019 | 19 | .1 | .1 | 99.6 |
|  | 020 | 34 | .1 | .1 | 99.8 |
|  | 021 | 27 | .1 | .1 | 99.8 |
|  | 022 | 2 | .0 | .0 | 99.8 |
|  | 023 | 8 | .0 | .0 | 99.9 |
|  | 024 | 4 | .0 | .0 | 99.9 |
|  | 025 | 1 | .0 | .0 | 99.9 |
|  | 026 | 1 | .0 | .0 | 99.9 |
|  | 027 | 4 | .0 | .0 | 99.9 |
|  | 028 | 3 | .0 | .0 | 99.9 |
|  | 029 | 10 | .0 | .0 | 99.9 |
|  | 030 | 8 | .0 | .0 | 100.0 |
|  | 031 | 5 | .0 | .0 | 100.0 |
|  | 032 | 3 | .0 | .0 | 100.0 |
|  | Total | 30553 | 100.0 | 100.0 |  |
|  |  |  |  |  |  |

Respondent id numberRespondent id number, table, 1 levels of column headers and 2 levels of row headers, table with 6 columns and 14 rows

|  |  |  |  |  |  |
| --- | --- | --- | --- | --- | --- |
|  | | Frequency | Percent | Valid Percent | Cumulative Percent |
| Valid |  | 29432 | 96.3 | 96.3 | 96.3 |
|  | 01 | 134 | .4 | .4 | 96.8 |
|  | 02 | 740 | 2.4 | 2.4 | 99.2 |
|  | 03 | 121 | .4 | .4 | 99.6 |
|  | 04 | 83 | .3 | .3 | 99.9 |
|  | 05 | 20 | .1 | .1 | 99.9 |
|  | 06 | 14 | .0 | .0 | 100.0 |
|  | 07 | 6 | .0 | .0 | 100.0 |
|  | 08 | 1 | .0 | .0 | 100.0 |
|  | 09 | 1 | .0 | .0 | 100.0 |
|  | 10 | 1 | .0 | .0 | 100.0 |
|  | Total | 30553 | 100.0 | 100.0 |  |
|  |  |  |  |  |  |

Household headâ€™s serial numberHousehold headâ€™s serial number, table, 1 levels of column headers and 2 levels of row headers, table with 6 columns and 5 rows

|  |  |  |  |  |  |
| --- | --- | --- | --- | --- | --- |
|  | | Frequency | Percent | Valid Percent | Cumulative Percent |
| Valid |  | 29432 | 96.3 | 96.3 | 96.3 |
|  | 01 | 1121 | 3.7 | 3.7 | 100.0 |
|  | Total | 30553 | 100.0 | 100.0 |  |
|  |  |  |  |  |  |

Visit dateVisit date, table, 1 levels of column headers and 2 levels of row headers, table with 6 columns and 110 rows

|  |  |  |  |  |  |
| --- | --- | --- | --- | --- | --- |
|  | | Frequency | Percent | Valid Percent | Cumulative Percent |
| Valid | 04-JUN-2013 | 1 | .0 | .1 | .1 |
|  | 07-JUN-2013 | 1 | .0 | .1 | .2 |
|  | 08-JUN-2013 | 1 | .0 | .1 | .3 |
|  | 09-JUN-2013 | 3 | .0 | .3 | .5 |
|  | 12-JUN-2013 | 1 | .0 | .1 | .6 |
|  | 14-JUN-2013 | 1 | .0 | .1 | .7 |
|  | 15-JUN-2013 | 1 | .0 | .1 | .8 |
|  | 16-JUN-2013 | 2 | .0 | .2 | 1.0 |
|  | 17-JUN-2013 | 2 | .0 | .2 | 1.2 |
|  | 18-JUN-2013 | 1 | .0 | .1 | 1.2 |
|  | 19-JUN-2013 | 1 | .0 | .1 | 1.3 |
|  | 20-JUN-2013 | 3 | .0 | .3 | 1.6 |
|  | 21-JUN-2013 | 1 | .0 | .1 | 1.7 |
|  | 22-JUN-2013 | 8 | .0 | .7 | 2.4 |
|  | 23-JUN-2013 | 1 | .0 | .1 | 2.5 |
|  | 24-JUN-2013 | 2 | .0 | .2 | 2.7 |
|  | 26-JUN-2013 | 6 | .0 | .5 | 3.2 |
|  | 27-JUN-2013 | 4 | .0 | .4 | 3.6 |
|  | 28-JUN-2013 | 1 | .0 | .1 | 3.7 |
|  | 29-JUN-2013 | 1 | .0 | .1 | 3.7 |
|  | 30-JUN-2013 | 1 | .0 | .1 | 3.8 |
|  | 01-JUL-2013 | 3 | .0 | .3 | 4.1 |
|  | 02-JUL-2013 | 5 | .0 | .4 | 4.5 |
|  | 03-JUL-2013 | 7 | .0 | .6 | 5.2 |
|  | 04-JUL-2013 | 5 | .0 | .4 | 5.6 |
|  | 06-JUL-2013 | 6 | .0 | .5 | 6.2 |
|  | 07-JUL-2013 | 7 | .0 | .6 | 6.8 |
|  | 08-JUL-2013 | 10 | .0 | .9 | 7.7 |
|  | 09-JUL-2013 | 5 | .0 | .4 | 8.1 |
|  | 10-JUL-2013 | 5 | .0 | .4 | 8.6 |
|  | 11-JUL-2013 | 9 | .0 | .8 | 9.4 |
|  | 13-JUL-2013 | 9 | .0 | .8 | 10.2 |
|  | 14-JUL-2013 | 7 | .0 | .6 | 10.8 |
|  | 15-JUL-2013 | 8 | .0 | .7 | 11.5 |
|  | 16-JUL-2013 | 8 | .0 | .7 | 12.2 |
|  | 17-JUL-2013 | 3 | .0 | .3 | 12.5 |
|  | 18-JUL-2013 | 7 | .0 | .6 | 13.1 |
|  | 20-JUL-2013 | 12 | .0 | 1.1 | 14.2 |
|  | 21-JUL-2013 | 9 | .0 | .8 | 15.0 |
|  | 22-JUL-2013 | 11 | .0 | 1.0 | 16.0 |
|  | 23-JUL-2013 | 12 | .0 | 1.1 | 17.0 |
|  | 24-JUL-2013 | 17 | .1 | 1.5 | 18.6 |
|  | 25-JUL-2013 | 11 | .0 | 1.0 | 19.5 |
|  | 26-JUL-2013 | 4 | .0 | .4 | 19.9 |
|  | 27-JUL-2013 | 10 | .0 | .9 | 20.8 |
|  | 28-JUL-2013 | 17 | .1 | 1.5 | 22.3 |
|  | 29-JUL-2013 | 9 | .0 | .8 | 23.1 |
|  | 30-JUL-2013 | 12 | .0 | 1.1 | 24.2 |
|  | 31-JUL-2013 | 8 | .0 | .7 | 24.9 |
|  | 01-AUG-2013 | 18 | .1 | 1.6 | 26.5 |
|  | 02-AUG-2013 | 5 | .0 | .4 | 26.9 |
|  | 03-AUG-2013 | 17 | .1 | 1.5 | 28.5 |
|  | 04-AUG-2013 | 17 | .1 | 1.5 | 30.0 |
|  | 05-AUG-2013 | 15 | .0 | 1.3 | 31.3 |
|  | 07-AUG-2013 | 8 | .0 | .7 | 32.0 |
|  | 09-AUG-2013 | 1 | .0 | .1 | 32.1 |
|  | 11-AUG-2013 | 10 | .0 | .9 | 33.0 |
|  | 12-AUG-2013 | 19 | .1 | 1.7 | 34.7 |
|  | 13-AUG-2013 | 14 | .0 | 1.2 | 36.0 |
|  | 14-AUG-2013 | 9 | .0 | .8 | 36.8 |
|  | 16-AUG-2013 | 1 | .0 | .1 | 36.8 |
|  | 17-AUG-2013 | 17 | .1 | 1.5 | 38.4 |
|  | 18-AUG-2013 | 12 | .0 | 1.1 | 39.4 |
|  | 19-AUG-2013 | 22 | .1 | 2.0 | 41.4 |
|  | 20-AUG-2013 | 16 | .1 | 1.4 | 42.8 |
|  | 21-AUG-2013 | 22 | .1 | 2.0 | 44.8 |
|  | 22-AUG-2013 | 20 | .1 | 1.8 | 46.6 |
|  | 23-AUG-2013 | 3 | .0 | .3 | 46.8 |
|  | 24-AUG-2013 | 19 | .1 | 1.7 | 48.5 |
|  | 25-AUG-2013 | 12 | .0 | 1.1 | 49.6 |
|  | 26-AUG-2013 | 17 | .1 | 1.5 | 51.1 |
|  | 27-AUG-2013 | 12 | .0 | 1.1 | 52.2 |
|  | 28-AUG-2013 | 8 | .0 | .7 | 52.9 |
|  | 29-AUG-2013 | 10 | .0 | .9 | 53.8 |
|  | 30-AUG-2013 | 1 | .0 | .1 | 53.9 |
|  | 31-AUG-2013 | 13 | .0 | 1.2 | 55.0 |
|  | 01-SEP-2013 | 18 | .1 | 1.6 | 56.6 |
|  | 02-SEP-2013 | 15 | .0 | 1.3 | 58.0 |
|  | 03-SEP-2013 | 23 | .1 | 2.1 | 60.0 |
|  | 04-SEP-2013 | 24 | .1 | 2.1 | 62.2 |
|  | 05-SEP-2013 | 18 | .1 | 1.6 | 63.8 |
|  | 07-SEP-2013 | 18 | .1 | 1.6 | 65.4 |
|  | 08-SEP-2013 | 11 | .0 | 1.0 | 66.4 |
|  | 09-SEP-2013 | 29 | .1 | 2.6 | 69.0 |
|  | 10-SEP-2013 | 21 | .1 | 1.9 | 70.8 |
|  | 11-SEP-2013 | 22 | .1 | 2.0 | 72.8 |
|  | 12-SEP-2013 | 31 | .1 | 2.8 | 75.6 |
|  | 13-SEP-2013 | 5 | .0 | .4 | 76.0 |
|  | 14-SEP-2013 | 28 | .1 | 2.5 | 78.5 |
|  | 15-SEP-2013 | 25 | .1 | 2.2 | 80.7 |
|  | 16-SEP-2013 | 34 | .1 | 3.0 | 83.8 |
|  | 17-SEP-2013 | 24 | .1 | 2.1 | 85.9 |
|  | 18-SEP-2013 | 23 | .1 | 2.1 | 88.0 |
|  | 19-SEP-2013 | 25 | .1 | 2.2 | 90.2 |
|  | 20-SEP-2013 | 1 | .0 | .1 | 90.3 |
|  | 21-SEP-2013 | 26 | .1 | 2.3 | 92.6 |
|  | 22-SEP-2013 | 26 | .1 | 2.3 | 94.9 |
|  | 23-SEP-2013 | 19 | .1 | 1.7 | 96.6 |
|  | 24-SEP-2013 | 13 | .0 | 1.2 | 97.8 |
|  | 25-SEP-2013 | 7 | .0 | .6 | 98.4 |
|  | 26-SEP-2013 | 5 | .0 | .4 | 98.8 |
|  | 27-SEP-2013 | 1 | .0 | .1 | 98.9 |
|  | 28-SEP-2013 | 6 | .0 | .5 | 99.5 |
|  | 29-SEP-2013 | 4 | .0 | .4 | 99.8 |
|  | 30-SEP-2013 | 2 | .0 | .2 | 100.0 |
|  | Total | 1121 | 3.7 | 100.0 |  |
| Missing | System | 29432 | 96.3 |  |  |
| Total | | 30553 | 100.0 |  |  |
|  |  |  |  |  |  |

Mother's serial number of the individualMother's serial number of the individual, table, 1 levels of column headers and 2 levels of row headers, table with 6 columns and 14 rows

|  |  |  |  |  |  |
| --- | --- | --- | --- | --- | --- |
|  | | Frequency | Percent | Valid Percent | Cumulative Percent |
| Valid | Personâ€™s mother is not available in that HH | 658 | 2.2 | 58.7 | 58.7 |
|  | 1 | 10 | .0 | .9 | 59.6 |
|  | 2 | 367 | 1.2 | 32.7 | 92.3 |
|  | 3 | 50 | .2 | 4.5 | 96.8 |
|  | 4 | 22 | .1 | 2.0 | 98.8 |
|  | 5 | 7 | .0 | .6 | 99.4 |
|  | 6 | 4 | .0 | .4 | 99.7 |
|  | 8 | 2 | .0 | .2 | 99.9 |
|  | 11 | 1 | .0 | .1 | 100.0 |
|  | Total | 1121 | 3.7 | 100.0 |  |
| Missing | System | 29432 | 96.3 |  |  |
| Total | | 30553 | 100.0 |  |  |
|  |  |  |  |  |  |

Age groupAge group, table, 1 levels of column headers and 2 levels of row headers, table with 6 columns and 13 rows

|  |  |  |  |  |  |
| --- | --- | --- | --- | --- | --- |
|  | | Frequency | Percent | Valid Percent | Cumulative Percent |
| Valid | <1yr | 11 | .0 | 1.0 | 1.0 |
|  | 1-4yr | 118 | .4 | 10.5 | 11.5 |
|  | 5-9yr | 140 | .5 | 12.5 | 24.0 |
|  | 10-14yr | 83 | .3 | 7.4 | 31.4 |
|  | 15-17yr | 15 | .0 | 1.3 | 32.7 |
|  | 18-24yr | 100 | .3 | 8.9 | 41.7 |
|  | 25-64yr | 596 | 2.0 | 53.2 | 94.8 |
|  | 65+ | 58 | .2 | 5.2 | 100.0 |
|  | Total | 1121 | 3.7 | 100.0 |  |
| Missing | System | 29432 | 96.3 |  |  |
| Total | | 30553 | 100.0 |  |  |
|  |  |  |  |  |  |

Date of birthDate of birth, table, 1 levels of column headers and 2 levels of row headers, table with 6 columns and 1053 rows

|  |  |  |  |  |  |
| --- | --- | --- | --- | --- | --- |
|  | | Frequency | Percent | Valid Percent | Cumulative Percent |
| Valid | 21-MAR-1924 | 2 | .0 | .2 | .2 |
|  | 19-SEP-1925 | 1 | .0 | .1 | .3 |
|  | 15-SEP-1928 | 1 | .0 | .1 | .4 |
|  | 16-MAR-1930 | 1 | .0 | .1 | .4 |
|  | 17-JAN-1933 | 1 | .0 | .1 | .5 |
|  | 22-JAN-1933 | 1 | .0 | .1 | .6 |
|  | 20-MAR-1933 | 1 | .0 | .1 | .7 |
|  | 11-MAY-1933 | 1 | .0 | .1 | .8 |
|  | 19-JUL-1933 | 1 | .0 | .1 | .9 |
|  | 01-AUG-1933 | 1 | .0 | .1 | 1.0 |
|  | 15-SEP-1937 | 1 | .0 | .1 | 1.1 |
|  | 02-MAY-1938 | 1 | .0 | .1 | 1.2 |
|  | 06-AUG-1938 | 1 | .0 | .1 | 1.2 |
|  | 12-FEB-1940 | 1 | .0 | .1 | 1.3 |
|  | 15-APR-1940 | 1 | .0 | .1 | 1.4 |
|  | 07-MAY-1940 | 1 | .0 | .1 | 1.5 |
|  | 12-SEP-1940 | 1 | .0 | .1 | 1.6 |
|  | 18-DEC-1940 | 1 | .0 | .1 | 1.7 |
|  | 13-FEB-1941 | 1 | .0 | .1 | 1.8 |
|  | 10-AUG-1942 | 1 | .0 | .1 | 1.9 |
|  | 04-JAN-1943 | 2 | .0 | .2 | 2.1 |
|  | 12-JAN-1943 | 1 | .0 | .1 | 2.1 |
|  | 19-JAN-1943 | 1 | .0 | .1 | 2.2 |
|  | 01-FEB-1943 | 1 | .0 | .1 | 2.3 |
|  | 03-FEB-1943 | 1 | .0 | .1 | 2.4 |
|  | 09-FEB-1943 | 1 | .0 | .1 | 2.5 |
|  | 10-FEB-1943 | 1 | .0 | .1 | 2.6 |
|  | 01-MAR-1943 | 1 | .0 | .1 | 2.7 |
|  | 02-MAR-1943 | 1 | .0 | .1 | 2.8 |
|  | 14-MAR-1943 | 1 | .0 | .1 | 2.9 |
|  | 19-MAR-1943 | 1 | .0 | .1 | 2.9 |
|  | 20-MAR-1943 | 1 | .0 | .1 | 3.0 |
|  | 21-APR-1943 | 1 | .0 | .1 | 3.1 |
|  | 22-MAY-1943 | 1 | .0 | .1 | 3.2 |
|  | 27-MAY-1943 | 1 | .0 | .1 | 3.3 |
|  | 01-JUL-1943 | 1 | .0 | .1 | 3.4 |
|  | 10-AUG-1943 | 1 | .0 | .1 | 3.5 |
|  | 20-SEP-1943 | 1 | .0 | .1 | 3.6 |
|  | 07-OCT-1943 | 1 | .0 | .1 | 3.7 |
|  | 04-MAR-1946 | 1 | .0 | .1 | 3.7 |
|  | 01-JUN-1946 | 1 | .0 | .1 | 3.8 |
|  | 13-JAN-1947 | 1 | .0 | .1 | 3.9 |
|  | 19-NOV-1947 | 1 | .0 | .1 | 4.0 |
|  | 20-NOV-1947 | 1 | .0 | .1 | 4.1 |
|  | 01-JAN-1948 | 1 | .0 | .1 | 4.2 |
|  | 10-JAN-1948 | 1 | .0 | .1 | 4.3 |
|  | 19-JAN-1948 | 1 | .0 | .1 | 4.4 |
|  | 02-FEB-1948 | 1 | .0 | .1 | 4.5 |
|  | 05-FEB-1948 | 1 | .0 | .1 | 4.5 |
|  | 01-MAR-1948 | 1 | .0 | .1 | 4.6 |
|  | 04-MAR-1948 | 1 | .0 | .1 | 4.7 |
|  | 10-MAR-1948 | 1 | .0 | .1 | 4.8 |
|  | 18-MAR-1948 | 1 | .0 | .1 | 4.9 |
|  | 03-MAY-1948 | 1 | .0 | .1 | 5.0 |
|  | 08-MAY-1948 | 1 | .0 | .1 | 5.1 |
|  | 15-JUN-1948 | 1 | .0 | .1 | 5.2 |
|  | 26-JUL-1948 | 1 | .0 | .1 | 5.3 |
|  | 09-JAN-1950 | 1 | .0 | .1 | 5.4 |
|  | 09-FEB-1950 | 1 | .0 | .1 | 5.4 |
|  | 11-FEB-1950 | 1 | .0 | .1 | 5.5 |
|  | 03-MAR-1950 | 1 | .0 | .1 | 5.6 |
|  | 05-APR-1950 | 1 | .0 | .1 | 5.7 |
|  | 11-APR-1950 | 1 | .0 | .1 | 5.8 |
|  | 17-APR-1950 | 2 | .0 | .2 | 6.0 |
|  | 18-APR-1950 | 1 | .0 | .1 | 6.1 |
|  | 10-MAY-1950 | 1 | .0 | .1 | 6.2 |
|  | 10-JUN-1950 | 1 | .0 | .1 | 6.2 |
|  | 05-JUL-1950 | 1 | .0 | .1 | 6.3 |
|  | 15-DEC-1950 | 1 | .0 | .1 | 6.4 |
|  | 16-MAY-1951 | 1 | .0 | .1 | 6.5 |
|  | 12-JUL-1951 | 1 | .0 | .1 | 6.6 |
|  | 12-JUN-1952 | 1 | .0 | .1 | 6.7 |
|  | 09-OCT-1952 | 1 | .0 | .1 | 6.8 |
|  | 08-NOV-1952 | 1 | .0 | .1 | 6.9 |
|  | 04-JAN-1953 | 1 | .0 | .1 | 7.0 |
|  | 19-JAN-1953 | 1 | .0 | .1 | 7.0 |
|  | 22-JAN-1953 | 1 | .0 | .1 | 7.1 |
|  | 03-FEB-1953 | 1 | .0 | .1 | 7.2 |
|  | 04-FEB-1953 | 1 | .0 | .1 | 7.3 |
|  | 09-FEB-1953 | 1 | .0 | .1 | 7.4 |
|  | 10-FEB-1953 | 2 | .0 | .2 | 7.6 |
|  | 12-FEB-1953 | 1 | .0 | .1 | 7.7 |
|  | 01-MAR-1953 | 1 | .0 | .1 | 7.8 |
|  | 11-MAR-1953 | 1 | .0 | .1 | 7.9 |
|  | 12-MAR-1953 | 1 | .0 | .1 | 7.9 |
|  | 19-MAR-1953 | 1 | .0 | .1 | 8.0 |
|  | 04-MAY-1953 | 2 | .0 | .2 | 8.2 |
|  | 08-MAY-1953 | 1 | .0 | .1 | 8.3 |
|  | 02-JUN-1953 | 1 | .0 | .1 | 8.4 |
|  | 11-JUN-1953 | 1 | .0 | .1 | 8.5 |
|  | 26-JUN-1953 | 1 | .0 | .1 | 8.6 |
|  | 01-JUL-1953 | 1 | .0 | .1 | 8.7 |
|  | 04-JUL-1953 | 1 | .0 | .1 | 8.7 |
|  | 10-JUL-1953 | 1 | .0 | .1 | 8.8 |
|  | 10-AUG-1953 | 1 | .0 | .1 | 8.9 |
|  | 15-AUG-1953 | 1 | .0 | .1 | 9.0 |
|  | 07-SEP-1953 | 1 | .0 | .1 | 9.1 |
|  | 10-SEP-1953 | 1 | .0 | .1 | 9.2 |
|  | 25-SEP-1953 | 1 | .0 | .1 | 9.3 |
|  | 02-OCT-1953 | 1 | .0 | .1 | 9.4 |
|  | 17-NOV-1953 | 1 | .0 | .1 | 9.5 |
|  | 01-DEC-1953 | 1 | .0 | .1 | 9.5 |
|  | 11-DEC-1953 | 1 | .0 | .1 | 9.6 |
|  | 01-JAN-1955 | 1 | .0 | .1 | 9.7 |
|  | 20-APR-1955 | 1 | .0 | .1 | 9.8 |
|  | 27-JUN-1955 | 1 | .0 | .1 | 9.9 |
|  | 11-JUL-1955 | 1 | .0 | .1 | 10.0 |
|  | 25-AUG-1955 | 1 | .0 | .1 | 10.1 |
|  | 10-OCT-1955 | 1 | .0 | .1 | 10.2 |
|  | 06-NOV-1955 | 1 | .0 | .1 | 10.3 |
|  | 27-NOV-1955 | 1 | .0 | .1 | 10.3 |
|  | 20-MAR-1956 | 1 | .0 | .1 | 10.4 |
|  | 17-MAY-1956 | 1 | .0 | .1 | 10.5 |
|  | 25-JUL-1956 | 1 | .0 | .1 | 10.6 |
|  | 08-FEB-1957 | 1 | .0 | .1 | 10.7 |
|  | 13-FEB-1957 | 1 | .0 | .1 | 10.8 |
|  | 28-JUN-1957 | 1 | .0 | .1 | 10.9 |
|  | 15-JUL-1957 | 1 | .0 | .1 | 11.0 |
|  | 06-SEP-1957 | 1 | .0 | .1 | 11.1 |
|  | 11-OCT-1957 | 1 | .0 | .1 | 11.2 |
|  | 20-OCT-1957 | 1 | .0 | .1 | 11.2 |
|  | 01-JAN-1958 | 1 | .0 | .1 | 11.3 |
|  | 02-JAN-1958 | 1 | .0 | .1 | 11.4 |
|  | 01-FEB-1958 | 1 | .0 | .1 | 11.5 |
|  | 03-FEB-1958 | 1 | .0 | .1 | 11.6 |
|  | 09-FEB-1958 | 1 | .0 | .1 | 11.7 |
|  | 11-FEB-1958 | 2 | .0 | .2 | 11.9 |
|  | 14-FEB-1958 | 2 | .0 | .2 | 12.0 |
|  | 15-FEB-1958 | 1 | .0 | .1 | 12.1 |
|  | 19-FEB-1958 | 1 | .0 | .1 | 12.2 |
|  | 20-FEB-1958 | 1 | .0 | .1 | 12.3 |
|  | 11-MAR-1958 | 1 | .0 | .1 | 12.4 |
|  | 12-MAR-1958 | 1 | .0 | .1 | 12.5 |
|  | 20-MAR-1958 | 2 | .0 | .2 | 12.7 |
|  | 25-MAR-1958 | 1 | .0 | .1 | 12.8 |
|  | 10-APR-1958 | 1 | .0 | .1 | 12.8 |
|  | 15-APR-1958 | 1 | .0 | .1 | 12.9 |
|  | 01-MAY-1958 | 1 | .0 | .1 | 13.0 |
|  | 06-MAY-1958 | 1 | .0 | .1 | 13.1 |
|  | 20-MAY-1958 | 1 | .0 | .1 | 13.2 |
|  | 21-MAY-1958 | 1 | .0 | .1 | 13.3 |
|  | 03-JUN-1958 | 1 | .0 | .1 | 13.4 |
|  | 06-JUN-1958 | 1 | .0 | .1 | 13.5 |
|  | 09-JUN-1958 | 1 | .0 | .1 | 13.6 |
|  | 22-JUN-1958 | 1 | .0 | .1 | 13.6 |
|  | 03-JUL-1958 | 1 | .0 | .1 | 13.7 |
|  | 05-JUL-1958 | 1 | .0 | .1 | 13.8 |
|  | 12-JUL-1958 | 1 | .0 | .1 | 13.9 |
|  | 02-AUG-1958 | 1 | .0 | .1 | 14.0 |
|  | 06-AUG-1958 | 1 | .0 | .1 | 14.1 |
|  | 10-SEP-1958 | 1 | .0 | .1 | 14.2 |
|  | 11-SEP-1958 | 1 | .0 | .1 | 14.3 |
|  | 17-SEP-1958 | 1 | .0 | .1 | 14.4 |
|  | 08-DEC-1958 | 1 | .0 | .1 | 14.5 |
|  | 11-FEB-1959 | 1 | .0 | .1 | 14.5 |
|  | 15-JUN-1959 | 1 | .0 | .1 | 14.6 |
|  | 17-AUG-1959 | 1 | .0 | .1 | 14.7 |
|  | 26-SEP-1959 | 1 | .0 | .1 | 14.8 |
|  | 14-OCT-1959 | 1 | .0 | .1 | 14.9 |
|  | 20-JAN-1960 | 1 | .0 | .1 | 15.0 |
|  | 01-FEB-1960 | 2 | .0 | .2 | 15.2 |
|  | 18-FEB-1960 | 2 | .0 | .2 | 15.3 |
|  | 14-MAR-1960 | 1 | .0 | .1 | 15.4 |
|  | 28-MAR-1960 | 1 | .0 | .1 | 15.5 |
|  | 12-APR-1960 | 1 | .0 | .1 | 15.6 |
|  | 10-MAY-1960 | 1 | .0 | .1 | 15.7 |
|  | 04-JUN-1960 | 1 | .0 | .1 | 15.8 |
|  | 05-JUL-1960 | 2 | .0 | .2 | 16.0 |
|  | 13-JUL-1960 | 1 | .0 | .1 | 16.1 |
|  | 11-SEP-1960 | 1 | .0 | .1 | 16.1 |
|  | 16-NOV-1960 | 1 | .0 | .1 | 16.2 |
|  | 29-DEC-1960 | 1 | .0 | .1 | 16.3 |
|  | 17-FEB-1961 | 2 | .0 | .2 | 16.5 |
|  | 20-MAR-1961 | 1 | .0 | .1 | 16.6 |
|  | 03-APR-1961 | 1 | .0 | .1 | 16.7 |
|  | 17-APR-1961 | 1 | .0 | .1 | 16.8 |
|  | 04-MAY-1961 | 1 | .0 | .1 | 16.9 |
|  | 06-JUN-1961 | 1 | .0 | .1 | 16.9 |
|  | 14-JUN-1961 | 1 | .0 | .1 | 17.0 |
|  | 21-OCT-1961 | 1 | .0 | .1 | 17.1 |
|  | 01-JAN-1962 | 1 | .0 | .1 | 17.2 |
|  | 10-JAN-1962 | 1 | .0 | .1 | 17.3 |
|  | 21-JAN-1962 | 1 | .0 | .1 | 17.4 |
|  | 05-MAR-1962 | 2 | .0 | .2 | 17.6 |
|  | 09-MAR-1962 | 1 | .0 | .1 | 17.7 |
|  | 10-MAR-1962 | 1 | .0 | .1 | 17.8 |
|  | 21-MAY-1962 | 1 | .0 | .1 | 17.8 |
|  | 02-JUN-1962 | 1 | .0 | .1 | 17.9 |
|  | 10-SEP-1962 | 1 | .0 | .1 | 18.0 |
|  | 22-SEP-1962 | 1 | .0 | .1 | 18.1 |
|  | 20-OCT-1962 | 1 | .0 | .1 | 18.2 |
|  | 01-NOV-1962 | 1 | .0 | .1 | 18.3 |
|  | 07-DEC-1962 | 1 | .0 | .1 | 18.4 |
|  | 01-JAN-1963 | 2 | .0 | .2 | 18.6 |
|  | 03-JAN-1963 | 1 | .0 | .1 | 18.6 |
|  | 05-JAN-1963 | 1 | .0 | .1 | 18.7 |
|  | 08-JAN-1963 | 1 | .0 | .1 | 18.8 |
|  | 10-FEB-1963 | 2 | .0 | .2 | 19.0 |
|  | 11-FEB-1963 | 1 | .0 | .1 | 19.1 |
|  | 12-FEB-1963 | 2 | .0 | .2 | 19.3 |
|  | 15-FEB-1963 | 1 | .0 | .1 | 19.4 |
|  | 19-FEB-1963 | 1 | .0 | .1 | 19.4 |
|  | 02-MAR-1963 | 2 | .0 | .2 | 19.6 |
|  | 04-MAR-1963 | 1 | .0 | .1 | 19.7 |
|  | 10-MAR-1963 | 1 | .0 | .1 | 19.8 |
|  | 13-MAR-1963 | 1 | .0 | .1 | 19.9 |
|  | 14-MAR-1963 | 1 | .0 | .1 | 20.0 |
|  | 15-MAR-1963 | 1 | .0 | .1 | 20.1 |
|  | 03-APR-1963 | 1 | .0 | .1 | 20.2 |
|  | 05-APR-1963 | 1 | .0 | .1 | 20.2 |
|  | 07-APR-1963 | 1 | .0 | .1 | 20.3 |
|  | 08-APR-1963 | 1 | .0 | .1 | 20.4 |
|  | 10-APR-1963 | 1 | .0 | .1 | 20.5 |
|  | 18-APR-1963 | 1 | .0 | .1 | 20.6 |
|  | 20-APR-1963 | 1 | .0 | .1 | 20.7 |
|  | 27-APR-1963 | 1 | .0 | .1 | 20.8 |
|  | 09-MAY-1963 | 1 | .0 | .1 | 20.9 |
|  | 11-MAY-1963 | 1 | .0 | .1 | 21.0 |
|  | 12-MAY-1963 | 1 | .0 | .1 | 21.1 |
|  | 16-MAY-1963 | 1 | .0 | .1 | 21.1 |
|  | 06-JUN-1963 | 1 | .0 | .1 | 21.2 |
|  | 07-JUN-1963 | 1 | .0 | .1 | 21.3 |
|  | 12-JUN-1963 | 2 | .0 | .2 | 21.5 |
|  | 18-JUN-1963 | 1 | .0 | .1 | 21.6 |
|  | 20-JUN-1963 | 1 | .0 | .1 | 21.7 |
|  | 08-JUL-1963 | 1 | .0 | .1 | 21.8 |
|  | 13-JUL-1963 | 1 | .0 | .1 | 21.9 |
|  | 23-JUL-1963 | 1 | .0 | .1 | 21.9 |
|  | 19-AUG-1963 | 1 | .0 | .1 | 22.0 |
|  | 14-SEP-1963 | 1 | .0 | .1 | 22.1 |
|  | 17-SEP-1963 | 1 | .0 | .1 | 22.2 |
|  | 05-OCT-1963 | 1 | .0 | .1 | 22.3 |
|  | 07-OCT-1963 | 1 | .0 | .1 | 22.4 |
|  | 02-FEB-1965 | 1 | .0 | .1 | 22.5 |
|  | 15-APR-1965 | 1 | .0 | .1 | 22.6 |
|  | 07-MAY-1965 | 1 | .0 | .1 | 22.7 |
|  | 10-MAY-1965 | 1 | .0 | .1 | 22.7 |
|  | 13-JUL-1965 | 1 | .0 | .1 | 22.8 |
|  | 29-SEP-1965 | 1 | .0 | .1 | 22.9 |
|  | 21-MAR-1966 | 1 | .0 | .1 | 23.0 |
|  | 01-JAN-1967 | 1 | .0 | .1 | 23.1 |
|  | 07-MAY-1967 | 1 | .0 | .1 | 23.2 |
|  | 12-MAY-1967 | 1 | .0 | .1 | 23.3 |
|  | 29-MAY-1967 | 1 | .0 | .1 | 23.4 |
|  | 04-JUN-1967 | 1 | .0 | .1 | 23.5 |
|  | 11-JUN-1967 | 1 | .0 | .1 | 23.6 |
|  | 06-DEC-1967 | 1 | .0 | .1 | 23.6 |
|  | 01-JAN-1968 | 1 | .0 | .1 | 23.7 |
|  | 02-JAN-1968 | 1 | .0 | .1 | 23.8 |
|  | 10-JAN-1968 | 1 | .0 | .1 | 23.9 |
|  | 01-FEB-1968 | 1 | .0 | .1 | 24.0 |
|  | 03-FEB-1968 | 1 | .0 | .1 | 24.1 |
|  | 01-APR-1968 | 1 | .0 | .1 | 24.2 |
|  | 20-MAY-1968 | 1 | .0 | .1 | 24.3 |
|  | 01-JUN-1968 | 1 | .0 | .1 | 24.4 |
|  | 12-JUN-1968 | 1 | .0 | .1 | 24.4 |
|  | 20-JUN-1968 | 1 | .0 | .1 | 24.5 |
|  | 05-JUL-1968 | 1 | .0 | .1 | 24.6 |
|  | 13-JUL-1968 | 1 | .0 | .1 | 24.7 |
|  | 01-SEP-1968 | 1 | .0 | .1 | 24.8 |
|  | 03-MAR-1969 | 1 | .0 | .1 | 24.9 |
|  | 10-MAR-1969 | 1 | .0 | .1 | 25.0 |
|  | 10-JUL-1969 | 1 | .0 | .1 | 25.1 |
|  | 01-JAN-1970 | 2 | .0 | .2 | 25.2 |
|  | 24-JAN-1970 | 1 | .0 | .1 | 25.3 |
|  | 10-FEB-1970 | 1 | .0 | .1 | 25.4 |
|  | 10-MAR-1970 | 1 | .0 | .1 | 25.5 |
|  | 15-MAR-1970 | 1 | .0 | .1 | 25.6 |
|  | 01-APR-1970 | 1 | .0 | .1 | 25.7 |
|  | 10-APR-1970 | 1 | .0 | .1 | 25.8 |
|  | 12-APR-1970 | 1 | .0 | .1 | 25.9 |
|  | 04-MAY-1970 | 1 | .0 | .1 | 26.0 |
|  | 09-MAY-1970 | 1 | .0 | .1 | 26.0 |
|  | 11-MAY-1970 | 1 | .0 | .1 | 26.1 |
|  | 10-JUN-1970 | 1 | .0 | .1 | 26.2 |
|  | 07-AUG-1970 | 1 | .0 | .1 | 26.3 |
|  | 09-SEP-1970 | 1 | .0 | .1 | 26.4 |
|  | 17-NOV-1970 | 1 | .0 | .1 | 26.5 |
|  | 03-DEC-1970 | 1 | .0 | .1 | 26.6 |
|  | 01-FEB-1971 | 1 | .0 | .1 | 26.7 |
|  | 20-MAR-1971 | 1 | .0 | .1 | 26.8 |
|  | 17-JUN-1971 | 1 | .0 | .1 | 26.9 |
|  | 03-DEC-1971 | 1 | .0 | .1 | 26.9 |
|  | 03-JAN-1972 | 1 | .0 | .1 | 27.0 |
|  | 08-FEB-1972 | 1 | .0 | .1 | 27.1 |
|  | 23-MAR-1972 | 1 | .0 | .1 | 27.2 |
|  | 10-MAY-1972 | 1 | .0 | .1 | 27.3 |
|  | 20-MAY-1972 | 1 | .0 | .1 | 27.4 |
|  | 20-SEP-1972 | 1 | .0 | .1 | 27.5 |
|  | 04-OCT-1972 | 1 | .0 | .1 | 27.6 |
|  | 23-DEC-1972 | 1 | .0 | .1 | 27.7 |
|  | 01-JAN-1973 | 1 | .0 | .1 | 27.7 |
|  | 01-FEB-1973 | 1 | .0 | .1 | 27.8 |
|  | 22-FEB-1973 | 1 | .0 | .1 | 27.9 |
|  | 25-FEB-1973 | 1 | .0 | .1 | 28.0 |
|  | 13-MAR-1973 | 1 | .0 | .1 | 28.1 |
|  | 20-MAR-1973 | 1 | .0 | .1 | 28.2 |
|  | 06-APR-1973 | 1 | .0 | .1 | 28.3 |
|  | 07-APR-1973 | 1 | .0 | .1 | 28.4 |
|  | 08-APR-1973 | 1 | .0 | .1 | 28.5 |
|  | 10-APR-1973 | 1 | .0 | .1 | 28.5 |
|  | 11-APR-1973 | 1 | .0 | .1 | 28.6 |
|  | 20-APR-1973 | 1 | .0 | .1 | 28.7 |
|  | 01-MAY-1973 | 1 | .0 | .1 | 28.8 |
|  | 06-MAY-1973 | 1 | .0 | .1 | 28.9 |
|  | 02-JUN-1973 | 1 | .0 | .1 | 29.0 |
|  | 10-JUN-1973 | 1 | .0 | .1 | 29.1 |
|  | 18-JUN-1973 | 1 | .0 | .1 | 29.2 |
|  | 10-JUL-1973 | 1 | .0 | .1 | 29.3 |
|  | 17-JUL-1973 | 1 | .0 | .1 | 29.3 |
|  | 20-JUL-1973 | 1 | .0 | .1 | 29.4 |
|  | 15-SEP-1973 | 1 | .0 | .1 | 29.5 |
|  | 10-MAR-1974 | 1 | .0 | .1 | 29.6 |
|  | 01-JAN-1975 | 1 | .0 | .1 | 29.7 |
|  | 03-JAN-1975 | 1 | .0 | .1 | 29.8 |
|  | 04-JAN-1975 | 1 | .0 | .1 | 29.9 |
|  | 19-JAN-1975 | 1 | .0 | .1 | 30.0 |
|  | 01-MAR-1975 | 1 | .0 | .1 | 30.1 |
|  | 12-MAR-1975 | 2 | .0 | .2 | 30.2 |
|  | 17-MAR-1975 | 1 | .0 | .1 | 30.3 |
|  | 18-APR-1975 | 1 | .0 | .1 | 30.4 |
|  | 04-MAY-1975 | 1 | .0 | .1 | 30.5 |
|  | 10-MAY-1975 | 1 | .0 | .1 | 30.6 |
|  | 05-JUN-1975 | 1 | .0 | .1 | 30.7 |
|  | 11-JUN-1975 | 1 | .0 | .1 | 30.8 |
|  | 12-JUN-1975 | 1 | .0 | .1 | 30.9 |
|  | 25-AUG-1975 | 1 | .0 | .1 | 31.0 |
|  | 11-SEP-1975 | 1 | .0 | .1 | 31.0 |
|  | 20-OCT-1975 | 1 | .0 | .1 | 31.1 |
|  | 04-NOV-1975 | 1 | .0 | .1 | 31.2 |
|  | 02-JAN-1976 | 1 | .0 | .1 | 31.3 |
|  | 15-JAN-1976 | 1 | .0 | .1 | 31.4 |
|  | 10-FEB-1976 | 1 | .0 | .1 | 31.5 |
|  | 02-APR-1976 | 1 | .0 | .1 | 31.6 |
|  | 16-APR-1976 | 2 | .0 | .2 | 31.8 |
|  | 03-MAY-1976 | 1 | .0 | .1 | 31.8 |
|  | 12-JUN-1976 | 1 | .0 | .1 | 31.9 |
|  | 14-AUG-1976 | 1 | .0 | .1 | 32.0 |
|  | 02-MAR-1977 | 1 | .0 | .1 | 32.1 |
|  | 24-MAY-1977 | 1 | .0 | .1 | 32.2 |
|  | 06-JUL-1977 | 1 | .0 | .1 | 32.3 |
|  | 09-AUG-1977 | 1 | .0 | .1 | 32.4 |
|  | 11-AUG-1977 | 1 | .0 | .1 | 32.5 |
|  | 15-AUG-1977 | 2 | .0 | .2 | 32.6 |
|  | 06-SEP-1977 | 1 | .0 | .1 | 32.7 |
|  | 18-SEP-1977 | 1 | .0 | .1 | 32.8 |
|  | 01-NOV-1977 | 1 | .0 | .1 | 32.9 |
|  | 08-NOV-1977 | 1 | .0 | .1 | 33.0 |
|  | 01-JAN-1978 | 1 | .0 | .1 | 33.1 |
|  | 03-JAN-1978 | 1 | .0 | .1 | 33.2 |
|  | 22-JAN-1978 | 1 | .0 | .1 | 33.3 |
|  | 03-FEB-1978 | 1 | .0 | .1 | 33.4 |
|  | 12-FEB-1978 | 1 | .0 | .1 | 33.5 |
|  | 17-FEB-1978 | 1 | .0 | .1 | 33.5 |
|  | 18-FEB-1978 | 1 | .0 | .1 | 33.6 |
|  | 20-FEB-1978 | 1 | .0 | .1 | 33.7 |
|  | 02-MAR-1978 | 1 | .0 | .1 | 33.8 |
|  | 09-MAR-1978 | 1 | .0 | .1 | 33.9 |
|  | 12-MAR-1978 | 2 | .0 | .2 | 34.1 |
|  | 15-MAR-1978 | 1 | .0 | .1 | 34.2 |
|  | 28-MAR-1978 | 1 | .0 | .1 | 34.3 |
|  | 02-APR-1978 | 1 | .0 | .1 | 34.3 |
|  | 05-APR-1978 | 1 | .0 | .1 | 34.4 |
|  | 12-APR-1978 | 2 | .0 | .2 | 34.6 |
|  | 21-APR-1978 | 1 | .0 | .1 | 34.7 |
|  | 26-APR-1978 | 1 | .0 | .1 | 34.8 |
|  | 07-MAY-1978 | 1 | .0 | .1 | 34.9 |
|  | 12-MAY-1978 | 1 | .0 | .1 | 35.0 |
|  | 15-MAY-1978 | 1 | .0 | .1 | 35.1 |
|  | 17-MAY-1978 | 1 | .0 | .1 | 35.1 |
|  | 18-MAY-1978 | 1 | .0 | .1 | 35.2 |
|  | 03-JUN-1978 | 1 | .0 | .1 | 35.3 |
|  | 05-JUN-1978 | 1 | .0 | .1 | 35.4 |
|  | 09-JUN-1978 | 2 | .0 | .2 | 35.6 |
|  | 10-JUN-1978 | 2 | .0 | .2 | 35.8 |
|  | 22-JUN-1978 | 1 | .0 | .1 | 35.9 |
|  | 05-JUL-1978 | 1 | .0 | .1 | 36.0 |
|  | 08-JUL-1978 | 1 | .0 | .1 | 36.0 |
|  | 10-JUL-1978 | 1 | .0 | .1 | 36.1 |
|  | 12-JUL-1978 | 2 | .0 | .2 | 36.3 |
|  | 13-AUG-1978 | 1 | .0 | .1 | 36.4 |
|  | 20-AUG-1978 | 1 | .0 | .1 | 36.5 |
|  | 01-SEP-1978 | 1 | .0 | .1 | 36.6 |
|  | 10-SEP-1978 | 1 | .0 | .1 | 36.7 |
|  | 04-OCT-1978 | 1 | .0 | .1 | 36.8 |
|  | 10-OCT-1978 | 1 | .0 | .1 | 36.8 |
|  | 01-NOV-1978 | 1 | .0 | .1 | 36.9 |
|  | 10-MAR-1979 | 1 | .0 | .1 | 37.0 |
|  | 13-MAR-1979 | 1 | .0 | .1 | 37.1 |
|  | 18-APR-1979 | 1 | .0 | .1 | 37.2 |
|  | 03-MAY-1979 | 1 | .0 | .1 | 37.3 |
|  | 28-JUN-1979 | 1 | .0 | .1 | 37.4 |
|  | 09-JUL-1979 | 1 | .0 | .1 | 37.5 |
|  | 08-DEC-1979 | 1 | .0 | .1 | 37.6 |
|  | 02-JAN-1980 | 1 | .0 | .1 | 37.6 |
|  | 04-JAN-1980 | 1 | .0 | .1 | 37.7 |
|  | 21-JAN-1980 | 1 | .0 | .1 | 37.8 |
|  | 02-APR-1980 | 1 | .0 | .1 | 37.9 |
|  | 10-APR-1980 | 1 | .0 | .1 | 38.0 |
|  | 12-APR-1980 | 1 | .0 | .1 | 38.1 |
|  | 14-APR-1980 | 1 | .0 | .1 | 38.2 |
|  | 20-APR-1980 | 1 | .0 | .1 | 38.3 |
|  | 14-MAY-1980 | 1 | .0 | .1 | 38.4 |
|  | 18-AUG-1980 | 1 | .0 | .1 | 38.4 |
|  | 04-OCT-1980 | 1 | .0 | .1 | 38.5 |
|  | 20-OCT-1980 | 1 | .0 | .1 | 38.6 |
|  | 17-NOV-1980 | 1 | .0 | .1 | 38.7 |
|  | 23-NOV-1980 | 1 | .0 | .1 | 38.8 |
|  | 10-DEC-1980 | 1 | .0 | .1 | 38.9 |
|  | 25-DEC-1980 | 1 | .0 | .1 | 39.0 |
|  | 30-DEC-1980 | 1 | .0 | .1 | 39.1 |
|  | 31-DEC-1980 | 1 | .0 | .1 | 39.2 |
|  | 13-JAN-1981 | 1 | .0 | .1 | 39.3 |
|  | 25-JAN-1981 | 2 | .0 | .2 | 39.4 |
|  | 10-FEB-1981 | 1 | .0 | .1 | 39.5 |
|  | 23-FEB-1981 | 1 | .0 | .1 | 39.6 |
|  | 24-FEB-1981 | 1 | .0 | .1 | 39.7 |
|  | 27-FEB-1981 | 1 | .0 | .1 | 39.8 |
|  | 18-APR-1981 | 1 | .0 | .1 | 39.9 |
|  | 09-MAY-1981 | 1 | .0 | .1 | 40.0 |
|  | 29-MAY-1981 | 1 | .0 | .1 | 40.1 |
|  | 05-JUN-1981 | 1 | .0 | .1 | 40.1 |
|  | 10-JUN-1981 | 1 | .0 | .1 | 40.2 |
|  | 13-JUN-1981 | 1 | .0 | .1 | 40.3 |
|  | 21-JUN-1981 | 1 | .0 | .1 | 40.4 |
|  | 06-AUG-1981 | 1 | .0 | .1 | 40.5 |
|  | 25-NOV-1981 | 1 | .0 | .1 | 40.6 |
|  | 20-FEB-1982 | 1 | .0 | .1 | 40.7 |
|  | 01-MAR-1982 | 1 | .0 | .1 | 40.8 |
|  | 03-MAR-1982 | 1 | .0 | .1 | 40.9 |
|  | 11-MAR-1982 | 1 | .0 | .1 | 40.9 |
|  | 14-MAR-1982 | 1 | .0 | .1 | 41.0 |
|  | 01-APR-1982 | 1 | .0 | .1 | 41.1 |
|  | 02-APR-1982 | 1 | .0 | .1 | 41.2 |
|  | 03-APR-1982 | 1 | .0 | .1 | 41.3 |
|  | 07-APR-1982 | 1 | .0 | .1 | 41.4 |
|  | 11-MAY-1982 | 1 | .0 | .1 | 41.5 |
|  | 08-JUL-1982 | 1 | .0 | .1 | 41.6 |
|  | 12-JUL-1982 | 1 | .0 | .1 | 41.7 |
|  | 13-JUL-1982 | 1 | .0 | .1 | 41.7 |
|  | 06-AUG-1982 | 1 | .0 | .1 | 41.8 |
|  | 15-AUG-1982 | 1 | .0 | .1 | 41.9 |
|  | 20-AUG-1982 | 1 | .0 | .1 | 42.0 |
|  | 03-SEP-1982 | 1 | .0 | .1 | 42.1 |
|  | 02-OCT-1982 | 1 | .0 | .1 | 42.2 |
|  | 16-OCT-1982 | 1 | .0 | .1 | 42.3 |
|  | 25-OCT-1982 | 1 | .0 | .1 | 42.4 |
|  | 28-OCT-1982 | 1 | .0 | .1 | 42.5 |
|  | 09-NOV-1982 | 1 | .0 | .1 | 42.6 |
|  | 01-JAN-1983 | 2 | .0 | .2 | 42.7 |
|  | 02-JAN-1983 | 2 | .0 | .2 | 42.9 |
|  | 03-JAN-1983 | 1 | .0 | .1 | 43.0 |
|  | 15-JAN-1983 | 1 | .0 | .1 | 43.1 |
|  | 01-FEB-1983 | 2 | .0 | .2 | 43.3 |
|  | 02-FEB-1983 | 1 | .0 | .1 | 43.4 |
|  | 10-FEB-1983 | 2 | .0 | .2 | 43.5 |
|  | 13-FEB-1983 | 1 | .0 | .1 | 43.6 |
|  | 14-FEB-1983 | 1 | .0 | .1 | 43.7 |
|  | 17-FEB-1983 | 2 | .0 | .2 | 43.9 |
|  | 23-FEB-1983 | 1 | .0 | .1 | 44.0 |
|  | 02-MAR-1983 | 2 | .0 | .2 | 44.2 |
|  | 06-MAR-1983 | 2 | .0 | .2 | 44.3 |
|  | 08-MAR-1983 | 1 | .0 | .1 | 44.4 |
|  | 10-MAR-1983 | 1 | .0 | .1 | 44.5 |
|  | 12-MAR-1983 | 3 | .0 | .3 | 44.8 |
|  | 15-MAR-1983 | 1 | .0 | .1 | 44.9 |
|  | 03-APR-1983 | 1 | .0 | .1 | 45.0 |
|  | 05-APR-1983 | 1 | .0 | .1 | 45.0 |
|  | 06-APR-1983 | 1 | .0 | .1 | 45.1 |
|  | 12-APR-1983 | 1 | .0 | .1 | 45.2 |
|  | 18-APR-1983 | 1 | .0 | .1 | 45.3 |
|  | 19-APR-1983 | 1 | .0 | .1 | 45.4 |
|  | 22-APR-1983 | 1 | .0 | .1 | 45.5 |
|  | 28-APR-1983 | 1 | .0 | .1 | 45.6 |
|  | 08-MAY-1983 | 1 | .0 | .1 | 45.7 |
|  | 10-MAY-1983 | 1 | .0 | .1 | 45.8 |
|  | 12-MAY-1983 | 1 | .0 | .1 | 45.9 |
|  | 13-MAY-1983 | 1 | .0 | .1 | 45.9 |
|  | 15-MAY-1983 | 1 | .0 | .1 | 46.0 |
|  | 28-MAY-1983 | 1 | .0 | .1 | 46.1 |
|  | 30-MAY-1983 | 1 | .0 | .1 | 46.2 |
|  | 02-JUN-1983 | 1 | .0 | .1 | 46.3 |
|  | 05-JUN-1983 | 1 | .0 | .1 | 46.4 |
|  | 15-JUN-1983 | 1 | .0 | .1 | 46.5 |
|  | 18-JUN-1983 | 1 | .0 | .1 | 46.6 |
|  | 29-JUN-1983 | 1 | .0 | .1 | 46.7 |
|  | 19-JUL-1983 | 1 | .0 | .1 | 46.7 |
|  | 28-JUL-1983 | 1 | .0 | .1 | 46.8 |
|  | 30-JUL-1983 | 1 | .0 | .1 | 46.9 |
|  | 07-AUG-1983 | 1 | .0 | .1 | 47.0 |
|  | 10-SEP-1983 | 1 | .0 | .1 | 47.1 |
|  | 02-OCT-1983 | 1 | .0 | .1 | 47.2 |
|  | 06-OCT-1983 | 1 | .0 | .1 | 47.3 |
|  | 15-DEC-1983 | 1 | .0 | .1 | 47.4 |
|  | 21-DEC-1983 | 1 | .0 | .1 | 47.5 |
|  | 01-JAN-1984 | 1 | .0 | .1 | 47.5 |
|  | 01-FEB-1984 | 1 | .0 | .1 | 47.6 |
|  | 11-FEB-1984 | 1 | .0 | .1 | 47.7 |
|  | 18-FEB-1984 | 1 | .0 | .1 | 47.8 |
|  | 19-FEB-1984 | 1 | .0 | .1 | 47.9 |
|  | 01-MAR-1984 | 1 | .0 | .1 | 48.0 |
|  | 18-MAR-1984 | 1 | .0 | .1 | 48.1 |
|  | 20-MAR-1984 | 1 | .0 | .1 | 48.2 |
|  | 10-MAY-1984 | 1 | .0 | .1 | 48.3 |
|  | 03-JUN-1984 | 1 | .0 | .1 | 48.3 |
|  | 01-NOV-1984 | 1 | .0 | .1 | 48.4 |
|  | 02-JAN-1985 | 1 | .0 | .1 | 48.5 |
|  | 14-JAN-1985 | 1 | .0 | .1 | 48.6 |
|  | 15-JAN-1985 | 1 | .0 | .1 | 48.7 |
|  | 01-FEB-1985 | 2 | .0 | .2 | 48.9 |
|  | 06-FEB-1985 | 1 | .0 | .1 | 49.0 |
|  | 12-FEB-1985 | 1 | .0 | .1 | 49.1 |
|  | 27-FEB-1985 | 1 | .0 | .1 | 49.2 |
|  | 08-MAR-1985 | 1 | .0 | .1 | 49.2 |
|  | 14-MAR-1985 | 1 | .0 | .1 | 49.3 |
|  | 20-MAR-1985 | 1 | .0 | .1 | 49.4 |
|  | 01-APR-1985 | 1 | .0 | .1 | 49.5 |
|  | 10-APR-1985 | 1 | .0 | .1 | 49.6 |
|  | 19-APR-1985 | 1 | .0 | .1 | 49.7 |
|  | 01-MAY-1985 | 1 | .0 | .1 | 49.8 |
|  | 03-MAY-1985 | 1 | .0 | .1 | 49.9 |
|  | 05-MAY-1985 | 1 | .0 | .1 | 50.0 |
|  | 14-MAY-1985 | 1 | .0 | .1 | 50.0 |
|  | 22-MAY-1985 | 1 | .0 | .1 | 50.1 |
|  | 05-JUN-1985 | 1 | .0 | .1 | 50.2 |
|  | 10-JUN-1985 | 3 | .0 | .3 | 50.5 |
|  | 11-JUN-1985 | 1 | .0 | .1 | 50.6 |
|  | 12-JUN-1985 | 1 | .0 | .1 | 50.7 |
|  | 13-JUN-1985 | 1 | .0 | .1 | 50.8 |
|  | 15-JUN-1985 | 1 | .0 | .1 | 50.8 |
|  | 17-JUN-1985 | 1 | .0 | .1 | 50.9 |
|  | 10-JUL-1985 | 1 | .0 | .1 | 51.0 |
|  | 13-JUL-1985 | 1 | .0 | .1 | 51.1 |
|  | 20-JUL-1985 | 1 | .0 | .1 | 51.2 |
|  | 12-AUG-1985 | 1 | .0 | .1 | 51.3 |
|  | 13-SEP-1985 | 1 | .0 | .1 | 51.4 |
|  | 12-OCT-1985 | 1 | .0 | .1 | 51.5 |
|  | 16-OCT-1985 | 1 | .0 | .1 | 51.6 |
|  | 10-NOV-1985 | 1 | .0 | .1 | 51.7 |
|  | 09-MAR-1986 | 1 | .0 | .1 | 51.7 |
|  | 10-MAR-1986 | 1 | .0 | .1 | 51.8 |
|  | 13-APR-1986 | 1 | .0 | .1 | 51.9 |
|  | 25-APR-1986 | 1 | .0 | .1 | 52.0 |
|  | 08-JUN-1986 | 1 | .0 | .1 | 52.1 |
|  | 10-JUN-1986 | 1 | .0 | .1 | 52.2 |
|  | 29-JUN-1986 | 1 | .0 | .1 | 52.3 |
|  | 18-JUL-1986 | 1 | .0 | .1 | 52.4 |
|  | 21-SEP-1986 | 1 | .0 | .1 | 52.5 |
|  | 28-SEP-1986 | 1 | .0 | .1 | 52.5 |
|  | 01-JAN-1987 | 1 | .0 | .1 | 52.6 |
|  | 10-JAN-1987 | 1 | .0 | .1 | 52.7 |
|  | 22-JAN-1987 | 1 | .0 | .1 | 52.8 |
|  | 07-FEB-1987 | 1 | .0 | .1 | 52.9 |
|  | 10-FEB-1987 | 1 | .0 | .1 | 53.0 |
|  | 11-FEB-1987 | 1 | .0 | .1 | 53.1 |
|  | 05-MAR-1987 | 1 | .0 | .1 | 53.2 |
|  | 06-APR-1987 | 1 | .0 | .1 | 53.3 |
|  | 08-APR-1987 | 1 | .0 | .1 | 53.3 |
|  | 10-APR-1987 | 1 | .0 | .1 | 53.4 |
|  | 27-APR-1987 | 1 | .0 | .1 | 53.5 |
|  | 10-MAY-1987 | 1 | .0 | .1 | 53.6 |
|  | 13-MAY-1987 | 1 | .0 | .1 | 53.7 |
|  | 14-MAY-1987 | 1 | .0 | .1 | 53.8 |
|  | 15-MAY-1987 | 1 | .0 | .1 | 53.9 |
|  | 20-MAY-1987 | 1 | .0 | .1 | 54.0 |
|  | 03-JUN-1987 | 1 | .0 | .1 | 54.1 |
|  | 19-JUN-1987 | 1 | .0 | .1 | 54.1 |
|  | 27-JUN-1987 | 1 | .0 | .1 | 54.2 |
|  | 10-JUL-1987 | 1 | .0 | .1 | 54.3 |
|  | 17-JUL-1987 | 1 | .0 | .1 | 54.4 |
|  | 20-JUL-1987 | 1 | .0 | .1 | 54.5 |
|  | 11-AUG-1987 | 1 | .0 | .1 | 54.6 |
|  | 18-AUG-1987 | 1 | .0 | .1 | 54.7 |
|  | 10-SEP-1987 | 1 | .0 | .1 | 54.8 |
|  | 15-SEP-1987 | 2 | .0 | .2 | 55.0 |
|  | 17-SEP-1987 | 1 | .0 | .1 | 55.0 |
|  | 08-OCT-1987 | 1 | .0 | .1 | 55.1 |
|  | 17-NOV-1987 | 1 | .0 | .1 | 55.2 |
|  | 28-DEC-1987 | 1 | .0 | .1 | 55.3 |
|  | 01-JAN-1988 | 3 | .0 | .3 | 55.6 |
|  | 07-JAN-1988 | 1 | .0 | .1 | 55.7 |
|  | 19-JAN-1988 | 1 | .0 | .1 | 55.8 |
|  | 01-FEB-1988 | 1 | .0 | .1 | 55.8 |
|  | 20-FEB-1988 | 1 | .0 | .1 | 55.9 |
|  | 01-MAR-1988 | 1 | .0 | .1 | 56.0 |
|  | 02-MAR-1988 | 1 | .0 | .1 | 56.1 |
|  | 03-MAR-1988 | 1 | .0 | .1 | 56.2 |
|  | 10-MAR-1988 | 2 | .0 | .2 | 56.4 |
|  | 14-MAR-1988 | 1 | .0 | .1 | 56.5 |
|  | 25-MAR-1988 | 1 | .0 | .1 | 56.6 |
|  | 04-APR-1988 | 1 | .0 | .1 | 56.6 |
|  | 22-APR-1988 | 2 | .0 | .2 | 56.8 |
|  | 06-MAY-1988 | 1 | .0 | .1 | 56.9 |
|  | 11-MAY-1988 | 1 | .0 | .1 | 57.0 |
|  | 17-MAY-1988 | 1 | .0 | .1 | 57.1 |
|  | 19-MAY-1988 | 1 | .0 | .1 | 57.2 |
|  | 25-MAY-1988 | 1 | .0 | .1 | 57.3 |
|  | 02-JUN-1988 | 1 | .0 | .1 | 57.4 |
|  | 08-JUN-1988 | 1 | .0 | .1 | 57.4 |
|  | 12-JUN-1988 | 1 | .0 | .1 | 57.5 |
|  | 15-JUN-1988 | 1 | .0 | .1 | 57.6 |
|  | 07-JUL-1988 | 1 | .0 | .1 | 57.7 |
|  | 09-JUL-1988 | 1 | .0 | .1 | 57.8 |
|  | 13-JUL-1988 | 1 | .0 | .1 | 57.9 |
|  | 04-AUG-1988 | 1 | .0 | .1 | 58.0 |
|  | 18-AUG-1988 | 1 | .0 | .1 | 58.1 |
|  | 03-OCT-1988 | 1 | .0 | .1 | 58.2 |
|  | 08-OCT-1988 | 1 | .0 | .1 | 58.3 |
|  | 16-DEC-1988 | 1 | .0 | .1 | 58.3 |
|  | 05-JAN-1989 | 1 | .0 | .1 | 58.4 |
|  | 10-FEB-1989 | 1 | .0 | .1 | 58.5 |
|  | 11-FEB-1989 | 1 | .0 | .1 | 58.6 |
|  | 07-MAR-1989 | 1 | .0 | .1 | 58.7 |
|  | 10-MAR-1989 | 1 | .0 | .1 | 58.8 |
|  | 10-MAY-1989 | 1 | .0 | .1 | 58.9 |
|  | 22-MAY-1989 | 1 | .0 | .1 | 59.0 |
|  | 04-JUN-1989 | 1 | .0 | .1 | 59.1 |
|  | 10-JUN-1989 | 1 | .0 | .1 | 59.1 |
|  | 14-JUN-1989 | 1 | .0 | .1 | 59.2 |
|  | 12-JUL-1989 | 1 | .0 | .1 | 59.3 |
|  | 19-JUL-1989 | 1 | .0 | .1 | 59.4 |
|  | 20-JUL-1989 | 1 | .0 | .1 | 59.5 |
|  | 03-AUG-1989 | 1 | .0 | .1 | 59.6 |
|  | 26-DEC-1989 | 1 | .0 | .1 | 59.7 |
|  | 01-JAN-1990 | 3 | .0 | .3 | 59.9 |
|  | 05-JAN-1990 | 1 | .0 | .1 | 60.0 |
|  | 12-JAN-1990 | 1 | .0 | .1 | 60.1 |
|  | 31-JAN-1990 | 1 | .0 | .1 | 60.2 |
|  | 01-FEB-1990 | 1 | .0 | .1 | 60.3 |
|  | 12-FEB-1990 | 1 | .0 | .1 | 60.4 |
|  | 13-FEB-1990 | 1 | .0 | .1 | 60.5 |
|  | 16-FEB-1990 | 1 | .0 | .1 | 60.6 |
|  | 01-MAR-1990 | 1 | .0 | .1 | 60.7 |
|  | 10-MAR-1990 | 1 | .0 | .1 | 60.7 |
|  | 14-MAR-1990 | 1 | .0 | .1 | 60.8 |
|  | 10-APR-1990 | 1 | .0 | .1 | 60.9 |
|  | 12-APR-1990 | 1 | .0 | .1 | 61.0 |
|  | 12-MAY-1990 | 1 | .0 | .1 | 61.1 |
|  | 21-MAY-1990 | 1 | .0 | .1 | 61.2 |
|  | 12-JUN-1990 | 1 | .0 | .1 | 61.3 |
|  | 21-JUN-1990 | 1 | .0 | .1 | 61.4 |
|  | 01-JUL-1990 | 1 | .0 | .1 | 61.5 |
|  | 07-JUL-1990 | 1 | .0 | .1 | 61.6 |
|  | 13-SEP-1990 | 1 | .0 | .1 | 61.6 |
|  | 15-SEP-1990 | 1 | .0 | .1 | 61.7 |
|  | 26-SEP-1990 | 1 | .0 | .1 | 61.8 |
|  | 10-NOV-1990 | 1 | .0 | .1 | 61.9 |
|  | 05-DEC-1990 | 1 | .0 | .1 | 62.0 |
|  | 07-JAN-1991 | 1 | .0 | .1 | 62.1 |
|  | 10-FEB-1991 | 2 | .0 | .2 | 62.3 |
|  | 17-MAR-1991 | 1 | .0 | .1 | 62.4 |
|  | 18-MAR-1991 | 1 | .0 | .1 | 62.4 |
|  | 03-APR-1991 | 1 | .0 | .1 | 62.5 |
|  | 07-APR-1991 | 1 | .0 | .1 | 62.6 |
|  | 02-MAY-1991 | 1 | .0 | .1 | 62.7 |
|  | 08-MAY-1991 | 1 | .0 | .1 | 62.8 |
|  | 20-JUN-1991 | 1 | .0 | .1 | 62.9 |
|  | 03-JUL-1991 | 1 | .0 | .1 | 63.0 |
|  | 10-AUG-1991 | 1 | .0 | .1 | 63.1 |
|  | 08-OCT-1991 | 1 | .0 | .1 | 63.2 |
|  | 28-NOV-1991 | 1 | .0 | .1 | 63.2 |
|  | 30-DEC-1991 | 1 | .0 | .1 | 63.3 |
|  | 09-JAN-1992 | 1 | .0 | .1 | 63.4 |
|  | 15-MAR-1992 | 1 | .0 | .1 | 63.5 |
|  | 11-APR-1992 | 1 | .0 | .1 | 63.6 |
|  | 05-JUL-1992 | 1 | .0 | .1 | 63.7 |
|  | 18-SEP-1992 | 1 | .0 | .1 | 63.8 |
|  | 17-NOV-1992 | 1 | .0 | .1 | 63.9 |
|  | 15-DEC-1992 | 1 | .0 | .1 | 64.0 |
|  | 24-DEC-1992 | 1 | .0 | .1 | 64.0 |
|  | 13-JAN-1993 | 1 | .0 | .1 | 64.1 |
|  | 08-FEB-1993 | 1 | .0 | .1 | 64.2 |
|  | 13-FEB-1993 | 1 | .0 | .1 | 64.3 |
|  | 15-FEB-1993 | 1 | .0 | .1 | 64.4 |
|  | 08-MAR-1993 | 1 | .0 | .1 | 64.5 |
|  | 11-MAR-1993 | 1 | .0 | .1 | 64.6 |
|  | 16-MAR-1993 | 1 | .0 | .1 | 64.7 |
|  | 10-MAY-1993 | 1 | .0 | .1 | 64.8 |
|  | 12-JUN-1993 | 1 | .0 | .1 | 64.9 |
|  | 18-JUN-1993 | 1 | .0 | .1 | 64.9 |
|  | 08-JUL-1993 | 1 | .0 | .1 | 65.0 |
|  | 16-JUL-1993 | 1 | .0 | .1 | 65.1 |
|  | 09-DEC-1993 | 1 | .0 | .1 | 65.2 |
|  | 10-JAN-1994 | 1 | .0 | .1 | 65.3 |
|  | 17-JAN-1994 | 1 | .0 | .1 | 65.4 |
|  | 01-FEB-1994 | 1 | .0 | .1 | 65.5 |
|  | 28-FEB-1994 | 1 | .0 | .1 | 65.6 |
|  | 16-MAR-1994 | 1 | .0 | .1 | 65.7 |
|  | 02-APR-1994 | 1 | .0 | .1 | 65.7 |
|  | 18-MAY-1994 | 1 | .0 | .1 | 65.8 |
|  | 20-MAY-1994 | 1 | .0 | .1 | 65.9 |
|  | 01-JAN-1995 | 1 | .0 | .1 | 66.0 |
|  | 01-FEB-1995 | 1 | .0 | .1 | 66.1 |
|  | 10-FEB-1995 | 1 | .0 | .1 | 66.2 |
|  | 23-FEB-1995 | 1 | .0 | .1 | 66.3 |
|  | 25-FEB-1995 | 1 | .0 | .1 | 66.4 |
|  | 01-MAR-1995 | 1 | .0 | .1 | 66.5 |
|  | 13-MAR-1995 | 1 | .0 | .1 | 66.5 |
|  | 01-JUN-1995 | 1 | .0 | .1 | 66.6 |
|  | 02-JUN-1995 | 1 | .0 | .1 | 66.7 |
|  | 10-JUN-1995 | 1 | .0 | .1 | 66.8 |
|  | 20-JUN-1995 | 1 | .0 | .1 | 66.9 |
|  | 25-JUN-1995 | 1 | .0 | .1 | 67.0 |
|  | 18-JUL-1995 | 1 | .0 | .1 | 67.1 |
|  | 05-AUG-1995 | 1 | .0 | .1 | 67.2 |
|  | 10-DEC-1995 | 1 | .0 | .1 | 67.3 |
|  | 09-JAN-1996 | 1 | .0 | .1 | 67.4 |
|  | 10-FEB-1996 | 1 | .0 | .1 | 67.4 |
|  | 21-APR-1996 | 1 | .0 | .1 | 67.5 |
|  | 07-MAY-1996 | 1 | .0 | .1 | 67.6 |
|  | 03-JUL-1996 | 1 | .0 | .1 | 67.7 |
|  | 17-MAR-1997 | 1 | .0 | .1 | 67.8 |
|  | 04-JUN-1997 | 1 | .0 | .1 | 67.9 |
|  | 01-SEP-1997 | 1 | .0 | .1 | 68.0 |
|  | 18-JAN-1998 | 1 | .0 | .1 | 68.1 |
|  | 12-MAR-1998 | 1 | .0 | .1 | 68.2 |
|  | 27-APR-1998 | 1 | .0 | .1 | 68.2 |
|  | 01-JUN-1998 | 1 | .0 | .1 | 68.3 |
|  | 03-JUN-1998 | 1 | .0 | .1 | 68.4 |
|  | 17-JUL-1998 | 1 | .0 | .1 | 68.5 |
|  | 09-DEC-1998 | 1 | .0 | .1 | 68.6 |
|  | 21-MAR-1999 | 1 | .0 | .1 | 68.7 |
|  | 13-MAY-1999 | 1 | .0 | .1 | 68.8 |
|  | 30-JUN-1999 | 1 | .0 | .1 | 68.9 |
|  | 19-AUG-1999 | 1 | .0 | .1 | 69.0 |
|  | 20-AUG-1999 | 1 | .0 | .1 | 69.0 |
|  | 01-JAN-2000 | 1 | .0 | .1 | 69.1 |
|  | 05-JAN-2000 | 1 | .0 | .1 | 69.2 |
|  | 10-JAN-2000 | 1 | .0 | .1 | 69.3 |
|  | 01-FEB-2000 | 1 | .0 | .1 | 69.4 |
|  | 07-MAR-2000 | 1 | .0 | .1 | 69.5 |
|  | 11-MAR-2000 | 1 | .0 | .1 | 69.6 |
|  | 18-APR-2000 | 1 | .0 | .1 | 69.7 |
|  | 01-MAY-2000 | 1 | .0 | .1 | 69.8 |
|  | 13-JUN-2000 | 1 | .0 | .1 | 69.8 |
|  | 15-JUN-2000 | 1 | .0 | .1 | 69.9 |
|  | 22-JUN-2000 | 1 | .0 | .1 | 70.0 |
|  | 02-JUL-2000 | 1 | .0 | .1 | 70.1 |
|  | 05-JUL-2000 | 1 | .0 | .1 | 70.2 |
|  | 06-JUL-2000 | 1 | .0 | .1 | 70.3 |
|  | 13-JUL-2000 | 1 | .0 | .1 | 70.4 |
|  | 28-JUL-2000 | 1 | .0 | .1 | 70.5 |
|  | 20-AUG-2000 | 1 | .0 | .1 | 70.6 |
|  | 25-AUG-2000 | 1 | .0 | .1 | 70.7 |
|  | 13-NOV-2000 | 1 | .0 | .1 | 70.7 |
|  | 30-DEC-2000 | 1 | .0 | .1 | 70.8 |
|  | 02-JAN-2001 | 1 | .0 | .1 | 70.9 |
|  | 01-FEB-2001 | 1 | .0 | .1 | 71.0 |
|  | 10-FEB-2001 | 2 | .0 | .2 | 71.2 |
|  | 13-FEB-2001 | 1 | .0 | .1 | 71.3 |
|  | 18-MAR-2001 | 1 | .0 | .1 | 71.4 |
|  | 28-MAR-2001 | 1 | .0 | .1 | 71.5 |
|  | 02-APR-2001 | 1 | .0 | .1 | 71.5 |
|  | 05-APR-2001 | 1 | .0 | .1 | 71.6 |
|  | 09-APR-2001 | 1 | .0 | .1 | 71.7 |
|  | 14-APR-2001 | 1 | .0 | .1 | 71.8 |
|  | 06-MAY-2001 | 1 | .0 | .1 | 71.9 |
|  | 10-MAY-2001 | 1 | .0 | .1 | 72.0 |
|  | 11-MAY-2001 | 1 | .0 | .1 | 72.1 |
|  | 15-MAY-2001 | 1 | .0 | .1 | 72.2 |
|  | 18-MAY-2001 | 1 | .0 | .1 | 72.3 |
|  | 09-JUN-2001 | 1 | .0 | .1 | 72.3 |
|  | 10-JUN-2001 | 1 | .0 | .1 | 72.4 |
|  | 12-JUN-2001 | 1 | .0 | .1 | 72.5 |
|  | 16-JUN-2001 | 2 | .0 | .2 | 72.7 |
|  | 04-JUL-2001 | 1 | .0 | .1 | 72.8 |
|  | 04-AUG-2001 | 1 | .0 | .1 | 72.9 |
|  | 21-DEC-2001 | 1 | .0 | .1 | 73.0 |
|  | 17-JAN-2002 | 1 | .0 | .1 | 73.1 |
|  | 12-MAR-2002 | 1 | .0 | .1 | 73.1 |
|  | 16-MAR-2002 | 1 | .0 | .1 | 73.2 |
|  | 11-APR-2002 | 1 | .0 | .1 | 73.3 |
|  | 30-APR-2002 | 1 | .0 | .1 | 73.4 |
|  | 17-MAY-2002 | 1 | .0 | .1 | 73.5 |
|  | 20-JUN-2002 | 2 | .0 | .2 | 73.7 |
|  | 01-AUG-2002 | 1 | .0 | .1 | 73.8 |
|  | 23-OCT-2002 | 1 | .0 | .1 | 73.9 |
|  | 24-OCT-2002 | 1 | .0 | .1 | 74.0 |
|  | 10-NOV-2002 | 1 | .0 | .1 | 74.0 |
|  | 15-NOV-2002 | 1 | .0 | .1 | 74.1 |
|  | 18-NOV-2002 | 1 | .0 | .1 | 74.2 |
|  | 01-JAN-2003 | 2 | .0 | .2 | 74.4 |
|  | 02-JAN-2003 | 2 | .0 | .2 | 74.6 |
|  | 15-JAN-2003 | 1 | .0 | .1 | 74.7 |
|  | 19-JAN-2003 | 1 | .0 | .1 | 74.8 |
|  | 25-FEB-2003 | 1 | .0 | .1 | 74.8 |
|  | 10-MAR-2003 | 1 | .0 | .1 | 74.9 |
|  | 10-APR-2003 | 1 | .0 | .1 | 75.0 |
|  | 15-APR-2003 | 1 | .0 | .1 | 75.1 |
|  | 13-MAY-2003 | 2 | .0 | .2 | 75.3 |
|  | 25-MAY-2003 | 1 | .0 | .1 | 75.4 |
|  | 02-JUN-2003 | 1 | .0 | .1 | 75.5 |
|  | 04-JUN-2003 | 1 | .0 | .1 | 75.6 |
|  | 06-JUN-2003 | 1 | .0 | .1 | 75.6 |
|  | 19-JUN-2003 | 1 | .0 | .1 | 75.7 |
|  | 11-JUL-2003 | 1 | .0 | .1 | 75.8 |
|  | 03-AUG-2003 | 1 | .0 | .1 | 75.9 |
|  | 02-SEP-2003 | 1 | .0 | .1 | 76.0 |
|  | 01-DEC-2003 | 1 | .0 | .1 | 76.1 |
|  | 20-DEC-2003 | 1 | .0 | .1 | 76.2 |
|  | 01-JAN-2004 | 1 | .0 | .1 | 76.3 |
|  | 09-JAN-2004 | 1 | .0 | .1 | 76.4 |
|  | 04-FEB-2004 | 1 | .0 | .1 | 76.4 |
|  | 05-MAR-2004 | 1 | .0 | .1 | 76.5 |
|  | 12-MAR-2004 | 1 | .0 | .1 | 76.6 |
|  | 19-MAR-2004 | 1 | .0 | .1 | 76.7 |
|  | 26-MAR-2004 | 1 | .0 | .1 | 76.8 |
|  | 04-APR-2004 | 1 | .0 | .1 | 76.9 |
|  | 12-APR-2004 | 1 | .0 | .1 | 77.0 |
|  | 13-APR-2004 | 1 | .0 | .1 | 77.1 |
|  | 02-MAY-2004 | 1 | .0 | .1 | 77.2 |
|  | 10-MAY-2004 | 1 | .0 | .1 | 77.3 |
|  | 11-MAY-2004 | 1 | .0 | .1 | 77.3 |
|  | 13-MAY-2004 | 1 | .0 | .1 | 77.4 |
|  | 14-MAY-2004 | 1 | .0 | .1 | 77.5 |
|  | 15-MAY-2004 | 1 | .0 | .1 | 77.6 |
|  | 22-MAY-2004 | 1 | .0 | .1 | 77.7 |
|  | 05-JUN-2004 | 1 | .0 | .1 | 77.8 |
|  | 12-JUL-2004 | 1 | .0 | .1 | 77.9 |
|  | 10-AUG-2004 | 1 | .0 | .1 | 78.0 |
|  | 01-SEP-2004 | 1 | .0 | .1 | 78.1 |
|  | 02-OCT-2004 | 1 | .0 | .1 | 78.1 |
|  | 15-NOV-2004 | 1 | .0 | .1 | 78.2 |
|  | 01-DEC-2004 | 1 | .0 | .1 | 78.3 |
|  | 01-JAN-2005 | 2 | .0 | .2 | 78.5 |
|  | 04-JAN-2005 | 1 | .0 | .1 | 78.6 |
|  | 08-JAN-2005 | 1 | .0 | .1 | 78.7 |
|  | 10-JAN-2005 | 1 | .0 | .1 | 78.8 |
|  | 14-JAN-2005 | 1 | .0 | .1 | 78.9 |
|  | 20-JAN-2005 | 1 | .0 | .1 | 78.9 |
|  | 03-FEB-2005 | 1 | .0 | .1 | 79.0 |
|  | 01-MAR-2005 | 1 | .0 | .1 | 79.1 |
|  | 02-MAR-2005 | 1 | .0 | .1 | 79.2 |
|  | 04-MAR-2005 | 1 | .0 | .1 | 79.3 |
|  | 10-MAR-2005 | 1 | .0 | .1 | 79.4 |
|  | 18-MAR-2005 | 1 | .0 | .1 | 79.5 |
|  | 19-MAR-2005 | 1 | .0 | .1 | 79.6 |
|  | 01-APR-2005 | 1 | .0 | .1 | 79.7 |
|  | 02-APR-2005 | 2 | .0 | .2 | 79.8 |
|  | 03-APR-2005 | 1 | .0 | .1 | 79.9 |
|  | 10-APR-2005 | 1 | .0 | .1 | 80.0 |
|  | 12-APR-2005 | 1 | .0 | .1 | 80.1 |
|  | 01-MAY-2005 | 2 | .0 | .2 | 80.3 |
|  | 10-JUN-2005 | 1 | .0 | .1 | 80.4 |
|  | 18-JUN-2005 | 1 | .0 | .1 | 80.5 |
|  | 15-JUL-2005 | 1 | .0 | .1 | 80.6 |
|  | 06-AUG-2005 | 1 | .0 | .1 | 80.6 |
|  | 11-AUG-2005 | 1 | .0 | .1 | 80.7 |
|  | 31-AUG-2005 | 1 | .0 | .1 | 80.8 |
|  | 06-SEP-2005 | 1 | .0 | .1 | 80.9 |
|  | 08-SEP-2005 | 1 | .0 | .1 | 81.0 |
|  | 19-SEP-2005 | 1 | .0 | .1 | 81.1 |
|  | 28-SEP-2005 | 1 | .0 | .1 | 81.2 |
|  | 15-OCT-2005 | 2 | .0 | .2 | 81.4 |
|  | 18-OCT-2005 | 1 | .0 | .1 | 81.4 |
|  | 07-NOV-2005 | 1 | .0 | .1 | 81.5 |
|  | 07-DEC-2005 | 1 | .0 | .1 | 81.6 |
|  | 08-DEC-2005 | 1 | .0 | .1 | 81.7 |
|  | 12-DEC-2005 | 1 | .0 | .1 | 81.8 |
|  | 01-JAN-2006 | 1 | .0 | .1 | 81.9 |
|  | 03-JAN-2006 | 1 | .0 | .1 | 82.0 |
|  | 07-JAN-2006 | 1 | .0 | .1 | 82.1 |
|  | 16-JAN-2006 | 1 | .0 | .1 | 82.2 |
|  | 01-FEB-2006 | 1 | .0 | .1 | 82.2 |
|  | 02-FEB-2006 | 2 | .0 | .2 | 82.4 |
|  | 18-FEB-2006 | 1 | .0 | .1 | 82.5 |
|  | 01-MAR-2006 | 1 | .0 | .1 | 82.6 |
|  | 10-MAR-2006 | 1 | .0 | .1 | 82.7 |
|  | 16-MAR-2006 | 1 | .0 | .1 | 82.8 |
|  | 04-APR-2006 | 1 | .0 | .1 | 82.9 |
|  | 07-APR-2006 | 1 | .0 | .1 | 83.0 |
|  | 11-APR-2006 | 1 | .0 | .1 | 83.1 |
|  | 16-APR-2006 | 1 | .0 | .1 | 83.1 |
|  | 03-MAY-2006 | 1 | .0 | .1 | 83.2 |
|  | 08-MAY-2006 | 1 | .0 | .1 | 83.3 |
|  | 11-MAY-2006 | 1 | .0 | .1 | 83.4 |
|  | 18-MAY-2006 | 1 | .0 | .1 | 83.5 |
|  | 14-JUN-2006 | 1 | .0 | .1 | 83.6 |
|  | 29-JUN-2006 | 1 | .0 | .1 | 83.7 |
|  | 01-JUL-2006 | 1 | .0 | .1 | 83.8 |
|  | 10-JUL-2006 | 1 | .0 | .1 | 83.9 |
|  | 12-JUL-2006 | 1 | .0 | .1 | 83.9 |
|  | 13-JUL-2006 | 1 | .0 | .1 | 84.0 |
|  | 13-AUG-2006 | 1 | .0 | .1 | 84.1 |
|  | 19-AUG-2006 | 2 | .0 | .2 | 84.3 |
|  | 30-SEP-2006 | 1 | .0 | .1 | 84.4 |
|  | 02-JAN-2007 | 1 | .0 | .1 | 84.5 |
|  | 05-JAN-2007 | 1 | .0 | .1 | 84.6 |
|  | 20-JAN-2007 | 1 | .0 | .1 | 84.7 |
|  | 10-FEB-2007 | 1 | .0 | .1 | 84.7 |
|  | 15-FEB-2007 | 1 | .0 | .1 | 84.8 |
|  | 07-MAR-2007 | 1 | .0 | .1 | 84.9 |
|  | 08-MAR-2007 | 1 | .0 | .1 | 85.0 |
|  | 13-MAR-2007 | 1 | .0 | .1 | 85.1 |
|  | 15-MAR-2007 | 1 | .0 | .1 | 85.2 |
|  | 11-APR-2007 | 1 | .0 | .1 | 85.3 |
|  | 12-APR-2007 | 1 | .0 | .1 | 85.4 |
|  | 19-APR-2007 | 1 | .0 | .1 | 85.5 |
|  | 06-MAY-2007 | 1 | .0 | .1 | 85.5 |
|  | 10-MAY-2007 | 1 | .0 | .1 | 85.6 |
|  | 01-JUN-2007 | 1 | .0 | .1 | 85.7 |
|  | 09-JUN-2007 | 1 | .0 | .1 | 85.8 |
|  | 13-JUN-2007 | 1 | .0 | .1 | 85.9 |
|  | 09-JUL-2007 | 1 | .0 | .1 | 86.0 |
|  | 12-JUL-2007 | 1 | .0 | .1 | 86.1 |
|  | 13-JUL-2007 | 1 | .0 | .1 | 86.2 |
|  | 15-JUL-2007 | 1 | .0 | .1 | 86.3 |
|  | 20-JUL-2007 | 2 | .0 | .2 | 86.4 |
|  | 23-SEP-2007 | 1 | .0 | .1 | 86.5 |
|  | 02-OCT-2007 | 1 | .0 | .1 | 86.6 |
|  | 10-DEC-2007 | 1 | .0 | .1 | 86.7 |
|  | 30-DEC-2007 | 1 | .0 | .1 | 86.8 |
|  | 04-JAN-2008 | 1 | .0 | .1 | 86.9 |
|  | 13-JAN-2008 | 1 | .0 | .1 | 87.0 |
|  | 20-JAN-2008 | 1 | .0 | .1 | 87.1 |
|  | 05-FEB-2008 | 1 | .0 | .1 | 87.2 |
|  | 01-MAR-2008 | 1 | .0 | .1 | 87.2 |
|  | 02-MAR-2008 | 1 | .0 | .1 | 87.3 |
|  | 10-MAR-2008 | 1 | .0 | .1 | 87.4 |
|  | 05-APR-2008 | 1 | .0 | .1 | 87.5 |
|  | 09-APR-2008 | 1 | .0 | .1 | 87.6 |
|  | 14-APR-2008 | 1 | .0 | .1 | 87.7 |
|  | 09-MAY-2008 | 1 | .0 | .1 | 87.8 |
|  | 14-MAY-2008 | 1 | .0 | .1 | 87.9 |
|  | 18-MAY-2008 | 1 | .0 | .1 | 88.0 |
|  | 01-JUN-2008 | 1 | .0 | .1 | 88.0 |
|  | 05-JUN-2008 | 2 | .0 | .2 | 88.2 |
|  | 13-JUN-2008 | 1 | .0 | .1 | 88.3 |
|  | 21-AUG-2008 | 1 | .0 | .1 | 88.4 |
|  | 06-SEP-2008 | 1 | .0 | .1 | 88.5 |
|  | 02-OCT-2008 | 1 | .0 | .1 | 88.6 |
|  | 28-JAN-2009 | 1 | .0 | .1 | 88.7 |
|  | 01-FEB-2009 | 1 | .0 | .1 | 88.8 |
|  | 04-FEB-2009 | 1 | .0 | .1 | 88.8 |
|  | 08-FEB-2009 | 1 | .0 | .1 | 88.9 |
|  | 04-MAR-2009 | 1 | .0 | .1 | 89.0 |
|  | 03-JUN-2009 | 1 | .0 | .1 | 89.1 |
|  | 12-JUL-2009 | 1 | .0 | .1 | 89.2 |
|  | 01-AUG-2009 | 2 | .0 | .2 | 89.4 |
|  | 07-AUG-2009 | 1 | .0 | .1 | 89.5 |
|  | 24-AUG-2009 | 1 | .0 | .1 | 89.6 |
|  | 04-SEP-2009 | 1 | .0 | .1 | 89.7 |
|  | 15-DEC-2009 | 1 | .0 | .1 | 89.7 |
|  | 01-JAN-2010 | 1 | .0 | .1 | 89.8 |
|  | 02-JAN-2010 | 1 | .0 | .1 | 89.9 |
|  | 10-FEB-2010 | 1 | .0 | .1 | 90.0 |
|  | 27-MAR-2010 | 1 | .0 | .1 | 90.1 |
|  | 01-APR-2010 | 1 | .0 | .1 | 90.2 |
|  | 29-APR-2010 | 1 | .0 | .1 | 90.3 |
|  | 03-JUL-2010 | 1 | .0 | .1 | 90.4 |
|  | 10-JUL-2010 | 2 | .0 | .2 | 90.5 |
|  | 12-JUL-2010 | 1 | .0 | .1 | 90.6 |
|  | 25-JUL-2010 | 1 | .0 | .1 | 90.7 |
|  | 01-AUG-2010 | 1 | .0 | .1 | 90.8 |
|  | 10-AUG-2010 | 1 | .0 | .1 | 90.9 |
|  | 11-AUG-2010 | 1 | .0 | .1 | 91.0 |
|  | 12-AUG-2010 | 1 | .0 | .1 | 91.1 |
|  | 18-AUG-2010 | 1 | .0 | .1 | 91.2 |
|  | 20-AUG-2010 | 1 | .0 | .1 | 91.3 |
|  | 25-AUG-2010 | 1 | .0 | .1 | 91.3 |
|  | 28-AUG-2010 | 2 | .0 | .2 | 91.5 |
|  | 30-AUG-2010 | 1 | .0 | .1 | 91.6 |
|  | 03-SEP-2010 | 1 | .0 | .1 | 91.7 |
|  | 05-SEP-2010 | 1 | .0 | .1 | 91.8 |
|  | 07-SEP-2010 | 1 | .0 | .1 | 91.9 |
|  | 09-SEP-2010 | 2 | .0 | .2 | 92.1 |
|  | 15-SEP-2010 | 2 | .0 | .2 | 92.2 |
|  | 18-SEP-2010 | 1 | .0 | .1 | 92.3 |
|  | 21-SEP-2010 | 1 | .0 | .1 | 92.4 |
|  | 25-SEP-2010 | 1 | .0 | .1 | 92.5 |
|  | 04-OCT-2010 | 1 | .0 | .1 | 92.6 |
|  | 19-OCT-2010 | 1 | .0 | .1 | 92.7 |
|  | 25-OCT-2010 | 1 | .0 | .1 | 92.8 |
|  | 15-NOV-2010 | 1 | .0 | .1 | 92.9 |
|  | 06-DEC-2010 | 1 | .0 | .1 | 93.0 |
|  | 01-JAN-2011 | 1 | .0 | .1 | 93.0 |
|  | 12-JAN-2011 | 1 | .0 | .1 | 93.1 |
|  | 28-JAN-2011 | 1 | .0 | .1 | 93.2 |
|  | 20-FEB-2011 | 1 | .0 | .1 | 93.3 |
|  | 02-MAR-2011 | 1 | .0 | .1 | 93.4 |
|  | 03-MAR-2011 | 1 | .0 | .1 | 93.5 |
|  | 01-APR-2011 | 1 | .0 | .1 | 93.6 |
|  | 06-APR-2011 | 1 | .0 | .1 | 93.7 |
|  | 08-APR-2011 | 1 | .0 | .1 | 93.8 |
|  | 15-APR-2011 | 1 | .0 | .1 | 93.8 |
|  | 01-MAY-2011 | 1 | .0 | .1 | 93.9 |
|  | 25-MAY-2011 | 1 | .0 | .1 | 94.0 |
|  | 10-JUN-2011 | 1 | .0 | .1 | 94.1 |
|  | 14-JUN-2011 | 1 | .0 | .1 | 94.2 |
|  | 26-JUN-2011 | 1 | .0 | .1 | 94.3 |
|  | 30-JUN-2011 | 1 | .0 | .1 | 94.4 |
|  | 01-JUL-2011 | 1 | .0 | .1 | 94.5 |
|  | 05-JUL-2011 | 2 | .0 | .2 | 94.6 |
|  | 10-JUL-2011 | 1 | .0 | .1 | 94.7 |
|  | 07-AUG-2011 | 1 | .0 | .1 | 94.8 |
|  | 08-AUG-2011 | 1 | .0 | .1 | 94.9 |
|  | 18-AUG-2011 | 1 | .0 | .1 | 95.0 |
|  | 20-AUG-2011 | 1 | .0 | .1 | 95.1 |
|  | 21-AUG-2011 | 1 | .0 | .1 | 95.2 |
|  | 23-AUG-2011 | 1 | .0 | .1 | 95.3 |
|  | 27-AUG-2011 | 1 | .0 | .1 | 95.4 |
|  | 02-SEP-2011 | 1 | .0 | .1 | 95.5 |
|  | 07-SEP-2011 | 1 | .0 | .1 | 95.5 |
|  | 08-SEP-2011 | 1 | .0 | .1 | 95.6 |
|  | 12-SEP-2011 | 3 | .0 | .3 | 95.9 |
|  | 04-OCT-2011 | 1 | .0 | .1 | 96.0 |
|  | 07-OCT-2011 | 1 | .0 | .1 | 96.1 |
|  | 14-OCT-2011 | 1 | .0 | .1 | 96.2 |
|  | 20-OCT-2011 | 1 | .0 | .1 | 96.3 |
|  | 22-OCT-2011 | 1 | .0 | .1 | 96.3 |
|  | 10-NOV-2011 | 1 | .0 | .1 | 96.4 |
|  | 25-NOV-2011 | 1 | .0 | .1 | 96.5 |
|  | 13-DEC-2011 | 1 | .0 | .1 | 96.6 |
|  | 20-DEC-2011 | 1 | .0 | .1 | 96.7 |
|  | 29-DEC-2011 | 2 | .0 | .2 | 96.9 |
|  | 01-JAN-2012 | 2 | .0 | .2 | 97.1 |
|  | 02-JAN-2012 | 1 | .0 | .1 | 97.1 |
|  | 09-FEB-2012 | 1 | .0 | .1 | 97.2 |
|  | 01-MAR-2012 | 1 | .0 | .1 | 97.3 |
|  | 02-MAR-2012 | 1 | .0 | .1 | 97.4 |
|  | 03-MAR-2012 | 2 | .0 | .2 | 97.6 |
|  | 10-MAR-2012 | 1 | .0 | .1 | 97.7 |
|  | 12-MAR-2012 | 1 | .0 | .1 | 97.8 |
|  | 20-MAR-2012 | 1 | .0 | .1 | 97.9 |
|  | 26-MAR-2012 | 1 | .0 | .1 | 97.9 |
|  | 01-MAY-2012 | 1 | .0 | .1 | 98.0 |
|  | 05-MAY-2012 | 1 | .0 | .1 | 98.1 |
|  | 04-JUN-2012 | 1 | .0 | .1 | 98.2 |
|  | 10-JUN-2012 | 1 | .0 | .1 | 98.3 |
|  | 29-JUN-2012 | 1 | .0 | .1 | 98.4 |
|  | 10-JUL-2012 | 1 | .0 | .1 | 98.5 |
|  | 17-AUG-2012 | 1 | .0 | .1 | 98.6 |
|  | 06-SEP-2012 | 1 | .0 | .1 | 98.7 |
|  | 09-SEP-2012 | 1 | .0 | .1 | 98.8 |
|  | 10-SEP-2012 | 1 | .0 | .1 | 98.8 |
|  | 11-SEP-2012 | 1 | .0 | .1 | 98.9 |
|  | 15-SEP-2012 | 1 | .0 | .1 | 99.0 |
|  | 08-OCT-2012 | 1 | .0 | .1 | 99.1 |
|  | 14-OCT-2012 | 1 | .0 | .1 | 99.2 |
|  | 21-OCT-2012 | 1 | .0 | .1 | 99.3 |
|  | 29-OCT-2012 | 1 | .0 | .1 | 99.4 |
|  | 22-NOV-2012 | 1 | .0 | .1 | 99.5 |
|  | 25-NOV-2012 | 1 | .0 | .1 | 99.6 |
|  | 19-DEC-2012 | 1 | .0 | .1 | 99.6 |
|  | 10-JAN-2013 | 1 | .0 | .1 | 99.7 |
|  | 10-FEB-2013 | 1 | .0 | .1 | 99.8 |
|  | 02-MAR-2013 | 1 | .0 | .1 | 99.9 |
|  | 02-MAY-2013 | 1 | .0 | .1 | 100.0 |
|  | Total | 1121 | 3.7 | 100.0 |  |
| Missing | System | 29432 | 96.3 |  |  |
| Total | | 30553 | 100.0 |  |  |
|  |  |  |  |  |  |

Sex of the individualSex of the individual, table, 1 levels of column headers and 2 levels of row headers, table with 6 columns and 7 rows

|  |  |  |  |  |  |
| --- | --- | --- | --- | --- | --- |
|  | | Frequency | Percent | Valid Percent | Cumulative Percent |
| Valid | Male | 623 | 2.0 | 55.6 | 55.6 |
|  | Female | 498 | 1.6 | 44.4 | 100.0 |
|  | Total | 1121 | 3.7 | 100.0 |  |
| Missing | System | 29432 | 96.3 |  |  |
| Total | | 30553 | 100.0 |  |  |
|  |  |  |  |  |  |

5 quantiles of econ5 quantiles of econ, table, 1 levels of column headers and 2 levels of row headers, table with 6 columns and 10 rows

|  |  |  |  |  |  |
| --- | --- | --- | --- | --- | --- |
|  | | Frequency | Percent | Valid Percent | Cumulative Percent |
| Valid | Lowest | 240 | .8 | 21.4 | 21.4 |
|  | Low | 266 | .9 | 23.7 | 45.1 |
|  | Middle | 198 | .6 | 17.7 | 62.8 |
|  | High | 228 | .7 | 20.3 | 83.1 |
|  | Highest | 189 | .6 | 16.9 | 100.0 |
|  | Total | 1121 | 3.7 | 100.0 |  |
| Missing | System | 29432 | 96.3 |  |  |
| Total | | 30553 | 100.0 |  |  |
|  |  |  |  |  |  |

Age categoryAge category, table, 1 levels of column headers and 2 levels of row headers, table with 6 columns and 6 rows

|  |  |  |  |  |  |
| --- | --- | --- | --- | --- | --- |
|  | | Frequency | Percent | Valid Percent | Cumulative Percent |
| Valid | 1.00 | 8846 | 29.0 | 29.0 | 29.0 |
|  | 2.00 | 4917 | 16.1 | 16.1 | 45.0 |
|  | 3.00 | 16790 | 55.0 | 55.0 | 100.0 |
|  | Total | 30553 | 100.0 | 100.0 |  |
|  |  |  |  |  |  |
