# Supplementary material for: Compliance to playpen usages to enhance parental supervision of under-five children in rural community of Bangladesh
Source: PLoS One. 2022 May 9;17(5):e0264902. doi: 10.1371/journal.pone.0264902 (PMC9084520; doi:10.1371/journal.pone.0264902)
Supplement: S2 File — (HTM) [file pone.0264902.s002.htm]

DrPlayPenCompliance\_All Visits\_10March2022.htm


GET  
  FILE='D:\CreCHeS\JHU\_SoLiD 2 Paper\Playpen\Manuscript\Journal\PlosOne\_Playpen\Review-7\DrPlayPenCompliance\_All Visits\_10March2022.sav'.  
DATASET NAME DataSet1 WINDOW=FRONT.  
DESCRIPTIVES VARIABLES=Upazila W25 Age\_Cat Age\_category ChSex  
  /STATISTICS=MEAN STDDEV MIN MAX.

  

# **Descriptives**

  

[DataSet1] D:\CreCHeS\JHU\_SoLiD 2 Paper\Playpen\Manuscript\Journal\PlosOne\_Playpen\Review-7\DrPlayPenCompliance\_All Visits\_10March2022.sav

  

Descriptive StatisticsDescriptive Statistics, table, 1 levels of column headers and 1 levels of row headers, table with 6 columns and 8 rows

|  |  |  |  |  |  |
| --- | --- | --- | --- | --- | --- |
|  | N | Minimum | Maximum | Mean | Std. Deviation |
| Upazila code | 190290 | 4 | 6 | 5.14 | .710 |
| Currently participate in any income generating activities | 190290 | 1 | 2 | 1.91 | .280 |
| Age category | 190290 | 1 | 3 | 2.16 | .541 |
| Age\_category | 190290 | 1.00 | 4.00 | 1.7998 | .86846 |
| Sex of the individual | 190290 | 1 | 2 | 1.50 | .500 |
| Valid N (listwise) | 190290 |  |  |  |  |
|  |  |  |  |  |  |

FREQUENCIES VARIABLES=Dist Upazila Unions Village Bari HHNo Intcode Result MSlNo CareSl VisitNo  
    VDate BDate ChAge StTime EnTime ChSex MoSl W05a W05b W06 W07 W08 W09 W10 W11 W12a W12b W12c W12cAge  
    W12d W12dAge W12e W12eAge W12f W12fAge W12g W12gAge W12h W12hAge W12i W12iAge W12j W12jAge W12k  
    W12kAge W12X W12x1 W12xAge W13 W14 W15 W16 W17a W17b W17c W17d W17e W17f W17g W17h W17i W17x W17x1  
    W18 W19 W20 W21 W22a W22b W22c W22d W22e W22f W22x W22x1 W23 W24 W24a W25 W26 W27 Age\_Cat  
    Age\_category  
  /ORDER=ANALYSIS.

  

# **Frequencies**

  

StatisticsStatistics, table, 1 levels of column headers and 2 levels of row headers, table with 86 columns and 4 rows

|  |  |  |  |  |  |  |  |  |  |  |  |  |  |  |  |  |  |  |  |  |  |  |  |  |  |  |  |  |  |  |  |  |  |  |  |  |  |  |  |  |  |  |  |  |  |  |  |  |  |  |  |  |  |  |  |  |  |  |  |  |  |  |  |  |  |  |  |  |  |  |  |  |  |  |  |  |  |  |  |  |  |  |  |  |  |
| --- | --- | --- | --- | --- | --- | --- | --- | --- | --- | --- | --- | --- | --- | --- | --- | --- | --- | --- | --- | --- | --- | --- | --- | --- | --- | --- | --- | --- | --- | --- | --- | --- | --- | --- | --- | --- | --- | --- | --- | --- | --- | --- | --- | --- | --- | --- | --- | --- | --- | --- | --- | --- | --- | --- | --- | --- | --- | --- | --- | --- | --- | --- | --- | --- | --- | --- | --- | --- | --- | --- | --- | --- | --- | --- | --- | --- | --- | --- | --- | --- | --- | --- | --- | --- | --- |
|  | | District code | Upazila code | Unions code | Village code | Bari number | Identification number | Interviewer code | Interview result code | Serial # of HH members | Caregiver's serial number | Visit number | Visit date | Date of birth of individual | Child age | Interview start time | Interview end time | Sex of the individual | mother's serial number | relationship of primary caregiver with child | Age of the primary caregiver | Where the baby stay at the time of visit | Whether the child was in the playpen | What the child doing inside the playpen | Location of playpen | The physical condition of the playpen | What is the playpen being used | No one | Mother/primary caregiver | Paternal grandmother | Age of grandmother | paternal grandfather | Age of paternal grandfather | Father | Age of father | Maternal grandfather | Age of Maternal grandfather | Maternal grandmother | Age of Maternal grandmother | Sibling | Age of sibling | Cousin | Age of Cousin | Aunt/uncle | Age of Aunt/uncle | Neighbour | Age of Neighbour | Others | Other specify | Age of other person | Activites of mother/primary caregiver during that time | Whether the mother used the playpen since the intervention worker's last visit | Number of days that the playpen used in last week | Average number of times the baby kept in the playpen | Wash dishes/cloth | Taking care of the poultry/domestic animals | Collect fuel/water/irrigation work | Child Care-Bathing,teaching,feeding,taking to school | Care of other HH members | Other HH work e.g.Cleaning the HH | Working in others house | Leisure activities-chatting,sleeping,taking rest etc. | Cooking | Others | Other(Specify) | How long the child kept in the playpen during last use of the playpen | Whether the child got any injuries(fell out.cut,bruise)while the playpen being u | Describe in detail how the child got injured | Any difficulties faces during using the playpen | Inconvenient | Child does not want to stay in the playpen | Child goes to creche | Have a caregiver to look after the child | Don't think it is necessary | Have other methods of supervision(specify) | Others | Other specify | Satisfaction level with the playpen intervention | Any suggestions to improve the playpen intervention | Code of suggestion | Currently participate in any income generating activities | Number of hours per day devote to income generating activities | Numder of hours per day devote to household chores | Age category | Age\_category |
| N | Valid | 190290 | 190290 | 190290 | 190290 | 190290 | 190290 | 190290 | 190290 | 190290 | 152 | 190290 | 190290 | 190290 | 190290 | 190290 | 190290 | 190290 | 190290 | 4587 | 4587 | 190290 | 151199 | 91368 | 190290 | 190290 | 190290 | 190290 | 190290 | 190290 | 190290 | 190290 | 190290 | 190290 | 190290 | 190290 | 190290 | 190290 | 190290 | 190290 | 1543 | 190290 | 172 | 414 | 414 | 1607 | 1607 | 4190 | 0 | 4190 | 61502 | 190290 | 178868 | 178868 | 190290 | 190290 | 190290 | 190290 | 190290 | 190290 | 190290 | 190290 | 190290 | 190290 | 190290 | 178868 | 178870 | 190290 | 190290 | 190290 | 190290 | 190290 | 190290 | 190290 | 190290 | 190290 | 190290 | 190290 | 190290 | 190290 | 190290 | 190290 | 189615 | 190290 | 190290 |
|  | Missing | 0 | 0 | 0 | 0 | 0 | 0 | 0 | 0 | 0 | 190138 | 0 | 0 | 0 | 0 | 0 | 0 | 0 | 0 | 185703 | 185703 | 0 | 39091 | 98922 | 0 | 0 | 0 | 0 | 0 | 0 | 0 | 0 | 0 | 0 | 0 | 0 | 0 | 0 | 0 | 0 | 188747 | 0 | 190118 | 189876 | 189876 | 188683 | 188683 | 186100 | 190290 | 186100 | 128788 | 0 | 11422 | 11422 | 0 | 0 | 0 | 0 | 0 | 0 | 0 | 0 | 0 | 0 | 0 | 11422 | 11420 | 0 | 0 | 0 | 0 | 0 | 0 | 0 | 0 | 0 | 0 | 0 | 0 | 0 | 0 | 0 | 675 | 0 | 0 |
|  |  |  |  |  |  |  |  |  |  |  |  |  |  |  |  |  |  |  |  |  |  |  |  |  |  |  |  |  |  |  |  |  |  |  |  |  |  |  |  |  |  |  |  |  |  |  |  |  |  |  |  |  |  |  |  |  |  |  |  |  |  |  |  |  |  |  |  |  |  |  |  |  |  |  |  |  |  |  |  |  |  |  |  |  |  |

# **Frequency Table**

  

District codeDistrict code, table, 1 levels of column headers and 2 levels of row headers, table with 6 columns and 6 rows

|  |  |  |  |  |  |
| --- | --- | --- | --- | --- | --- |
|  | | Frequency | Percent | Valid Percent | Cumulative Percent |
| Valid | Sirajganj | 36203 | 19.0 | 19.0 | 19.0 |
|  | Sherpur | 90389 | 47.5 | 47.5 | 66.5 |
|  | Narshindi | 63698 | 33.5 | 33.5 | 100.0 |
|  | Total | 190290 | 100.0 | 100.0 |  |
|  |  |  |  |  |  |

Upazila codeUpazila code, table, 1 levels of column headers and 2 levels of row headers, table with 6 columns and 6 rows

|  |  |  |  |  |  |
| --- | --- | --- | --- | --- | --- |
|  | | Frequency | Percent | Valid Percent | Cumulative Percent |
| Valid | Raiganj | 36203 | 19.0 | 19.0 | 19.0 |
|  | Sherpur Sadar | 90389 | 47.5 | 47.5 | 66.5 |
|  | Manohardi | 63698 | 33.5 | 33.5 | 100.0 |
|  | Total | 190290 | 100.0 | 100.0 |  |
|  |  |  |  |  |  |

Unions codeUnions code, table, 1 levels of column headers and 2 levels of row headers, table with 6 columns and 12 rows

|  |  |  |  |  |  |
| --- | --- | --- | --- | --- | --- |
|  | | Frequency | Percent | Valid Percent | Cumulative Percent |
| Valid | 1 | 17052 | 9.0 | 9.0 | 9.0 |
|  | 2 | 20365 | 10.7 | 10.7 | 19.7 |
|  | 3 | 21051 | 11.1 | 11.1 | 30.7 |
|  | 4 | 28467 | 15.0 | 15.0 | 45.7 |
|  | 5 | 31666 | 16.6 | 16.6 | 62.3 |
|  | 6 | 36117 | 19.0 | 19.0 | 81.3 |
|  | 7 | 21898 | 11.5 | 11.5 | 92.8 |
|  | 8 | 7590 | 4.0 | 4.0 | 96.8 |
|  | 9 | 6084 | 3.2 | 3.2 | 100.0 |
|  | Total | 190290 | 100.0 | 100.0 |  |
|  |  |  |  |  |  |

Village codeVillage code, table, 1 levels of column headers and 2 levels of row headers, table with 6 columns and 35 rows

|  |  |  |  |  |  |
| --- | --- | --- | --- | --- | --- |
|  | | Frequency | Percent | Valid Percent | Cumulative Percent |
| Valid | 001 | 18308 | 9.6 | 9.6 | 9.6 |
|  | 002 | 14201 | 7.5 | 7.5 | 17.1 |
|  | 003 | 10435 | 5.5 | 5.5 | 22.6 |
|  | 004 | 8144 | 4.3 | 4.3 | 26.8 |
|  | 005 | 13926 | 7.3 | 7.3 | 34.2 |
|  | 006 | 12694 | 6.7 | 6.7 | 40.8 |
|  | 007 | 11898 | 6.3 | 6.3 | 47.1 |
|  | 008 | 12348 | 6.5 | 6.5 | 53.6 |
|  | 009 | 10566 | 5.6 | 5.6 | 59.1 |
|  | 010 | 14540 | 7.6 | 7.6 | 66.8 |
|  | 011 | 11145 | 5.9 | 5.9 | 72.6 |
|  | 012 | 9159 | 4.8 | 4.8 | 77.4 |
|  | 013 | 11657 | 6.1 | 6.1 | 83.6 |
|  | 014 | 5640 | 3.0 | 3.0 | 86.5 |
|  | 015 | 3555 | 1.9 | 1.9 | 88.4 |
|  | 016 | 2473 | 1.3 | 1.3 | 89.7 |
|  | 017 | 3486 | 1.8 | 1.8 | 91.5 |
|  | 018 | 3724 | 2.0 | 2.0 | 93.5 |
|  | 019 | 1554 | .8 | .8 | 94.3 |
|  | 020 | 1786 | .9 | .9 | 95.2 |
|  | 021 | 2005 | 1.1 | 1.1 | 96.3 |
|  | 022 | 1109 | .6 | .6 | 96.9 |
|  | 023 | 1117 | .6 | .6 | 97.5 |
|  | 024 | 1419 | .7 | .7 | 98.2 |
|  | 025 | 611 | .3 | .3 | 98.5 |
|  | 026 | 642 | .3 | .3 | 98.9 |
|  | 027 | 268 | .1 | .1 | 99.0 |
|  | 028 | 180 | .1 | .1 | 99.1 |
|  | 029 | 511 | .3 | .3 | 99.4 |
|  | 030 | 610 | .3 | .3 | 99.7 |
|  | 031 | 382 | .2 | .2 | 99.9 |
|  | 032 | 197 | .1 | .1 | 100.0 |
|  | Total | 190290 | 100.0 | 100.0 |  |
|  |  |  |  |  |  |

Bari numberBari number, table, 1 levels of column headers and 2 levels of row headers, table with 6 columns and 3 rows

|  |  |  |  |  |  |
| --- | --- | --- | --- | --- | --- |
|  | | Frequency | Percent | Valid Percent | Cumulative Percent |
| Valid |  | 190290 | 100.0 | 100.0 | 100.0 |
|  |  |  |  |  |  |

Identification numberIdentification number, table, 1 levels of column headers and 2 levels of row headers, table with 6 columns and 2478 rows

|  |  |  |  |  |  |
| --- | --- | --- | --- | --- | --- |
|  | | Frequency | Percent | Valid Percent | Cumulative Percent |
| Valid | 1 | 414 | .2 | .2 | .2 |
|  | 2 | 372 | .2 | .2 | .4 |
|  | 3 | 401 | .2 | .2 | .6 |
|  | 4 | 435 | .2 | .2 | .9 |
|  | 5 | 413 | .2 | .2 | 1.1 |
|  | 6 | 476 | .3 | .3 | 1.3 |
|  | 7 | 484 | .3 | .3 | 1.6 |
|  | 8 | 411 | .2 | .2 | 1.8 |
|  | 9 | 421 | .2 | .2 | 2.0 |
|  | 10 | 414 | .2 | .2 | 2.2 |
|  | 11 | 398 | .2 | .2 | 2.4 |
|  | 12 | 441 | .2 | .2 | 2.7 |
|  | 13 | 308 | .2 | .2 | 2.8 |
|  | 14 | 440 | .2 | .2 | 3.1 |
|  | 15 | 501 | .3 | .3 | 3.3 |
|  | 16 | 367 | .2 | .2 | 3.5 |
|  | 17 | 452 | .2 | .2 | 3.8 |
|  | 18 | 398 | .2 | .2 | 4.0 |
|  | 19 | 517 | .3 | .3 | 4.2 |
|  | 20 | 527 | .3 | .3 | 4.5 |
|  | 21 | 407 | .2 | .2 | 4.7 |
|  | 22 | 445 | .2 | .2 | 5.0 |
|  | 23 | 436 | .2 | .2 | 5.2 |
|  | 24 | 382 | .2 | .2 | 5.4 |
|  | 25 | 355 | .2 | .2 | 5.6 |
|  | 26 | 513 | .3 | .3 | 5.8 |
|  | 27 | 376 | .2 | .2 | 6.0 |
|  | 28 | 524 | .3 | .3 | 6.3 |
|  | 29 | 447 | .2 | .2 | 6.6 |
|  | 30 | 436 | .2 | .2 | 6.8 |
|  | 31 | 360 | .2 | .2 | 7.0 |
|  | 32 | 457 | .2 | .2 | 7.2 |
|  | 33 | 434 | .2 | .2 | 7.4 |
|  | 34 | 424 | .2 | .2 | 7.7 |
|  | 35 | 422 | .2 | .2 | 7.9 |
|  | 36 | 429 | .2 | .2 | 8.1 |
|  | 37 | 437 | .2 | .2 | 8.3 |
|  | 38 | 402 | .2 | .2 | 8.6 |
|  | 39 | 432 | .2 | .2 | 8.8 |
|  | 40 | 499 | .3 | .3 | 9.0 |
|  | 41 | 380 | .2 | .2 | 9.2 |
|  | 42 | 456 | .2 | .2 | 9.5 |
|  | 43 | 357 | .2 | .2 | 9.7 |
|  | 44 | 411 | .2 | .2 | 9.9 |
|  | 45 | 325 | .2 | .2 | 10.1 |
|  | 46 | 399 | .2 | .2 | 10.3 |
|  | 47 | 460 | .2 | .2 | 10.5 |
|  | 48 | 467 | .2 | .2 | 10.8 |
|  | 49 | 392 | .2 | .2 | 11.0 |
|  | 50 | 432 | .2 | .2 | 11.2 |
|  | 51 | 472 | .2 | .2 | 11.4 |
|  | 52 | 448 | .2 | .2 | 11.7 |
|  | 53 | 470 | .2 | .2 | 11.9 |
|  | 54 | 449 | .2 | .2 | 12.2 |
|  | 55 | 391 | .2 | .2 | 12.4 |
|  | 56 | 414 | .2 | .2 | 12.6 |
|  | 57 | 497 | .3 | .3 | 12.8 |
|  | 58 | 408 | .2 | .2 | 13.1 |
|  | 59 | 358 | .2 | .2 | 13.2 |
|  | 60 | 436 | .2 | .2 | 13.5 |
|  | 61 | 328 | .2 | .2 | 13.6 |
|  | 62 | 436 | .2 | .2 | 13.9 |
|  | 63 | 424 | .2 | .2 | 14.1 |
|  | 64 | 398 | .2 | .2 | 14.3 |
|  | 65 | 364 | .2 | .2 | 14.5 |
|  | 66 | 439 | .2 | .2 | 14.7 |
|  | 67 | 414 | .2 | .2 | 14.9 |
|  | 68 | 360 | .2 | .2 | 15.1 |
|  | 69 | 415 | .2 | .2 | 15.3 |
|  | 70 | 398 | .2 | .2 | 15.6 |
|  | 71 | 373 | .2 | .2 | 15.8 |
|  | 72 | 372 | .2 | .2 | 15.9 |
|  | 73 | 431 | .2 | .2 | 16.2 |
|  | 74 | 397 | .2 | .2 | 16.4 |
|  | 75 | 354 | .2 | .2 | 16.6 |
|  | 76 | 450 | .2 | .2 | 16.8 |
|  | 77 | 324 | .2 | .2 | 17.0 |
|  | 78 | 403 | .2 | .2 | 17.2 |
|  | 79 | 421 | .2 | .2 | 17.4 |
|  | 80 | 479 | .3 | .3 | 17.7 |
|  | 81 | 360 | .2 | .2 | 17.9 |
|  | 82 | 392 | .2 | .2 | 18.1 |
|  | 83 | 342 | .2 | .2 | 18.2 |
|  | 84 | 378 | .2 | .2 | 18.4 |
|  | 85 | 385 | .2 | .2 | 18.6 |
|  | 86 | 386 | .2 | .2 | 18.8 |
|  | 87 | 323 | .2 | .2 | 19.0 |
|  | 88 | 401 | .2 | .2 | 19.2 |
|  | 89 | 365 | .2 | .2 | 19.4 |
|  | 90 | 397 | .2 | .2 | 19.6 |
|  | 91 | 409 | .2 | .2 | 19.8 |
|  | 92 | 316 | .2 | .2 | 20.0 |
|  | 93 | 475 | .2 | .2 | 20.3 |
|  | 94 | 293 | .2 | .2 | 20.4 |
|  | 95 | 370 | .2 | .2 | 20.6 |
|  | 96 | 309 | .2 | .2 | 20.8 |
|  | 97 | 401 | .2 | .2 | 21.0 |
|  | 98 | 359 | .2 | .2 | 21.2 |
|  | 99 | 270 | .1 | .1 | 21.3 |
|  | 100 | 363 | .2 | .2 | 21.5 |
|  | 101 | 393 | .2 | .2 | 21.7 |
|  | 102 | 474 | .2 | .2 | 22.0 |
|  | 103 | 354 | .2 | .2 | 22.1 |
|  | 104 | 420 | .2 | .2 | 22.4 |
|  | 105 | 421 | .2 | .2 | 22.6 |
|  | 106 | 421 | .2 | .2 | 22.8 |
|  | 107 | 315 | .2 | .2 | 23.0 |
|  | 108 | 371 | .2 | .2 | 23.2 |
|  | 109 | 427 | .2 | .2 | 23.4 |
|  | 110 | 299 | .2 | .2 | 23.5 |
|  | 111 | 392 | .2 | .2 | 23.7 |
|  | 112 | 216 | .1 | .1 | 23.9 |
|  | 113 | 369 | .2 | .2 | 24.1 |
|  | 114 | 433 | .2 | .2 | 24.3 |
|  | 115 | 366 | .2 | .2 | 24.5 |
|  | 116 | 411 | .2 | .2 | 24.7 |
|  | 117 | 327 | .2 | .2 | 24.9 |
|  | 118 | 236 | .1 | .1 | 25.0 |
|  | 119 | 447 | .2 | .2 | 25.2 |
|  | 120 | 289 | .2 | .2 | 25.4 |
|  | 121 | 347 | .2 | .2 | 25.6 |
|  | 122 | 329 | .2 | .2 | 25.7 |
|  | 123 | 368 | .2 | .2 | 25.9 |
|  | 124 | 329 | .2 | .2 | 26.1 |
|  | 125 | 295 | .2 | .2 | 26.3 |
|  | 126 | 305 | .2 | .2 | 26.4 |
|  | 127 | 266 | .1 | .1 | 26.6 |
|  | 128 | 326 | .2 | .2 | 26.7 |
|  | 129 | 339 | .2 | .2 | 26.9 |
|  | 130 | 342 | .2 | .2 | 27.1 |
|  | 131 | 273 | .1 | .1 | 27.2 |
|  | 132 | 376 | .2 | .2 | 27.4 |
|  | 133 | 367 | .2 | .2 | 27.6 |
|  | 134 | 364 | .2 | .2 | 27.8 |
|  | 135 | 337 | .2 | .2 | 28.0 |
|  | 136 | 289 | .2 | .2 | 28.1 |
|  | 137 | 340 | .2 | .2 | 28.3 |
|  | 138 | 315 | .2 | .2 | 28.5 |
|  | 139 | 408 | .2 | .2 | 28.7 |
|  | 140 | 248 | .1 | .1 | 28.8 |
|  | 141 | 356 | .2 | .2 | 29.0 |
|  | 142 | 365 | .2 | .2 | 29.2 |
|  | 143 | 325 | .2 | .2 | 29.4 |
|  | 144 | 317 | .2 | .2 | 29.5 |
|  | 145 | 331 | .2 | .2 | 29.7 |
|  | 146 | 235 | .1 | .1 | 29.8 |
|  | 147 | 275 | .1 | .1 | 30.0 |
|  | 148 | 324 | .2 | .2 | 30.2 |
|  | 149 | 375 | .2 | .2 | 30.3 |
|  | 150 | 214 | .1 | .1 | 30.5 |
|  | 151 | 184 | .1 | .1 | 30.6 |
|  | 152 | 247 | .1 | .1 | 30.7 |
|  | 153 | 233 | .1 | .1 | 30.8 |
|  | 154 | 218 | .1 | .1 | 30.9 |
|  | 155 | 280 | .1 | .1 | 31.1 |
|  | 156 | 347 | .2 | .2 | 31.3 |
|  | 157 | 272 | .1 | .1 | 31.4 |
|  | 158 | 278 | .1 | .1 | 31.5 |
|  | 159 | 378 | .2 | .2 | 31.7 |
|  | 160 | 287 | .2 | .2 | 31.9 |
|  | 161 | 289 | .2 | .2 | 32.0 |
|  | 162 | 281 | .1 | .1 | 32.2 |
|  | 163 | 307 | .2 | .2 | 32.4 |
|  | 164 | 317 | .2 | .2 | 32.5 |
|  | 165 | 246 | .1 | .1 | 32.6 |
|  | 166 | 317 | .2 | .2 | 32.8 |
|  | 167 | 334 | .2 | .2 | 33.0 |
|  | 168 | 387 | .2 | .2 | 33.2 |
|  | 169 | 293 | .2 | .2 | 33.3 |
|  | 170 | 272 | .1 | .1 | 33.5 |
|  | 171 | 290 | .2 | .2 | 33.6 |
|  | 172 | 311 | .2 | .2 | 33.8 |
|  | 173 | 255 | .1 | .1 | 33.9 |
|  | 174 | 318 | .2 | .2 | 34.1 |
|  | 175 | 311 | .2 | .2 | 34.3 |
|  | 176 | 245 | .1 | .1 | 34.4 |
|  | 177 | 276 | .1 | .1 | 34.5 |
|  | 178 | 272 | .1 | .1 | 34.7 |
|  | 179 | 263 | .1 | .1 | 34.8 |
|  | 180 | 268 | .1 | .1 | 35.0 |
|  | 181 | 286 | .2 | .2 | 35.1 |
|  | 182 | 351 | .2 | .2 | 35.3 |
|  | 183 | 296 | .2 | .2 | 35.5 |
|  | 184 | 231 | .1 | .1 | 35.6 |
|  | 185 | 261 | .1 | .1 | 35.7 |
|  | 186 | 250 | .1 | .1 | 35.8 |
|  | 187 | 249 | .1 | .1 | 36.0 |
|  | 188 | 284 | .1 | .1 | 36.1 |
|  | 189 | 179 | .1 | .1 | 36.2 |
|  | 190 | 272 | .1 | .1 | 36.4 |
|  | 191 | 307 | .2 | .2 | 36.5 |
|  | 192 | 269 | .1 | .1 | 36.7 |
|  | 193 | 317 | .2 | .2 | 36.8 |
|  | 194 | 253 | .1 | .1 | 37.0 |
|  | 195 | 292 | .2 | .2 | 37.1 |
|  | 196 | 284 | .1 | .1 | 37.3 |
|  | 197 | 240 | .1 | .1 | 37.4 |
|  | 198 | 248 | .1 | .1 | 37.5 |
|  | 199 | 328 | .2 | .2 | 37.7 |
|  | 200 | 255 | .1 | .1 | 37.8 |
|  | 201 | 261 | .1 | .1 | 38.0 |
|  | 202 | 276 | .1 | .1 | 38.1 |
|  | 203 | 260 | .1 | .1 | 38.3 |
|  | 204 | 322 | .2 | .2 | 38.4 |
|  | 205 | 318 | .2 | .2 | 38.6 |
|  | 206 | 254 | .1 | .1 | 38.7 |
|  | 207 | 220 | .1 | .1 | 38.8 |
|  | 208 | 328 | .2 | .2 | 39.0 |
|  | 209 | 251 | .1 | .1 | 39.1 |
|  | 210 | 266 | .1 | .1 | 39.3 |
|  | 211 | 334 | .2 | .2 | 39.5 |
|  | 212 | 260 | .1 | .1 | 39.6 |
|  | 213 | 305 | .2 | .2 | 39.8 |
|  | 214 | 304 | .2 | .2 | 39.9 |
|  | 215 | 235 | .1 | .1 | 40.0 |
|  | 216 | 238 | .1 | .1 | 40.2 |
|  | 217 | 245 | .1 | .1 | 40.3 |
|  | 218 | 258 | .1 | .1 | 40.4 |
|  | 219 | 241 | .1 | .1 | 40.6 |
|  | 220 | 199 | .1 | .1 | 40.7 |
|  | 221 | 255 | .1 | .1 | 40.8 |
|  | 222 | 204 | .1 | .1 | 40.9 |
|  | 223 | 258 | .1 | .1 | 41.0 |
|  | 224 | 289 | .2 | .2 | 41.2 |
|  | 225 | 247 | .1 | .1 | 41.3 |
|  | 226 | 177 | .1 | .1 | 41.4 |
|  | 227 | 242 | .1 | .1 | 41.5 |
|  | 228 | 206 | .1 | .1 | 41.6 |
|  | 229 | 264 | .1 | .1 | 41.8 |
|  | 230 | 272 | .1 | .1 | 41.9 |
|  | 231 | 307 | .2 | .2 | 42.1 |
|  | 232 | 222 | .1 | .1 | 42.2 |
|  | 233 | 224 | .1 | .1 | 42.3 |
|  | 234 | 268 | .1 | .1 | 42.5 |
|  | 235 | 319 | .2 | .2 | 42.6 |
|  | 236 | 203 | .1 | .1 | 42.7 |
|  | 237 | 256 | .1 | .1 | 42.9 |
|  | 238 | 301 | .2 | .2 | 43.0 |
|  | 239 | 277 | .1 | .1 | 43.2 |
|  | 240 | 297 | .2 | .2 | 43.3 |
|  | 241 | 293 | .2 | .2 | 43.5 |
|  | 242 | 311 | .2 | .2 | 43.6 |
|  | 243 | 190 | .1 | .1 | 43.7 |
|  | 244 | 288 | .2 | .2 | 43.9 |
|  | 245 | 201 | .1 | .1 | 44.0 |
|  | 246 | 246 | .1 | .1 | 44.1 |
|  | 247 | 259 | .1 | .1 | 44.3 |
|  | 248 | 261 | .1 | .1 | 44.4 |
|  | 249 | 257 | .1 | .1 | 44.5 |
|  | 250 | 203 | .1 | .1 | 44.6 |
|  | 251 | 330 | .2 | .2 | 44.8 |
|  | 252 | 250 | .1 | .1 | 45.0 |
|  | 253 | 230 | .1 | .1 | 45.1 |
|  | 254 | 253 | .1 | .1 | 45.2 |
|  | 255 | 251 | .1 | .1 | 45.3 |
|  | 256 | 235 | .1 | .1 | 45.5 |
|  | 257 | 218 | .1 | .1 | 45.6 |
|  | 258 | 229 | .1 | .1 | 45.7 |
|  | 259 | 199 | .1 | .1 | 45.8 |
|  | 260 | 252 | .1 | .1 | 45.9 |
|  | 261 | 124 | .1 | .1 | 46.0 |
|  | 262 | 175 | .1 | .1 | 46.1 |
|  | 263 | 236 | .1 | .1 | 46.2 |
|  | 264 | 230 | .1 | .1 | 46.3 |
|  | 265 | 321 | .2 | .2 | 46.5 |
|  | 266 | 222 | .1 | .1 | 46.6 |
|  | 267 | 218 | .1 | .1 | 46.7 |
|  | 268 | 193 | .1 | .1 | 46.8 |
|  | 269 | 234 | .1 | .1 | 47.0 |
|  | 270 | 252 | .1 | .1 | 47.1 |
|  | 271 | 199 | .1 | .1 | 47.2 |
|  | 272 | 236 | .1 | .1 | 47.3 |
|  | 273 | 317 | .2 | .2 | 47.5 |
|  | 274 | 202 | .1 | .1 | 47.6 |
|  | 275 | 255 | .1 | .1 | 47.7 |
|  | 276 | 236 | .1 | .1 | 47.9 |
|  | 277 | 239 | .1 | .1 | 48.0 |
|  | 278 | 292 | .2 | .2 | 48.1 |
|  | 279 | 217 | .1 | .1 | 48.2 |
|  | 280 | 231 | .1 | .1 | 48.4 |
|  | 281 | 191 | .1 | .1 | 48.5 |
|  | 282 | 249 | .1 | .1 | 48.6 |
|  | 283 | 209 | .1 | .1 | 48.7 |
|  | 284 | 287 | .2 | .2 | 48.9 |
|  | 285 | 249 | .1 | .1 | 49.0 |
|  | 286 | 217 | .1 | .1 | 49.1 |
|  | 287 | 206 | .1 | .1 | 49.2 |
|  | 288 | 236 | .1 | .1 | 49.3 |
|  | 289 | 247 | .1 | .1 | 49.5 |
|  | 290 | 260 | .1 | .1 | 49.6 |
|  | 291 | 192 | .1 | .1 | 49.7 |
|  | 292 | 222 | .1 | .1 | 49.8 |
|  | 293 | 197 | .1 | .1 | 49.9 |
|  | 294 | 151 | .1 | .1 | 50.0 |
|  | 295 | 236 | .1 | .1 | 50.1 |
|  | 296 | 168 | .1 | .1 | 50.2 |
|  | 297 | 240 | .1 | .1 | 50.3 |
|  | 298 | 184 | .1 | .1 | 50.4 |
|  | 299 | 204 | .1 | .1 | 50.5 |
|  | 300 | 210 | .1 | .1 | 50.7 |
|  | 301 | 124 | .1 | .1 | 50.7 |
|  | 302 | 161 | .1 | .1 | 50.8 |
|  | 303 | 158 | .1 | .1 | 50.9 |
|  | 304 | 251 | .1 | .1 | 51.0 |
|  | 305 | 191 | .1 | .1 | 51.1 |
|  | 306 | 191 | .1 | .1 | 51.2 |
|  | 307 | 213 | .1 | .1 | 51.3 |
|  | 308 | 224 | .1 | .1 | 51.5 |
|  | 309 | 233 | .1 | .1 | 51.6 |
|  | 310 | 183 | .1 | .1 | 51.7 |
|  | 311 | 290 | .2 | .2 | 51.8 |
|  | 312 | 202 | .1 | .1 | 51.9 |
|  | 313 | 156 | .1 | .1 | 52.0 |
|  | 314 | 202 | .1 | .1 | 52.1 |
|  | 315 | 119 | .1 | .1 | 52.2 |
|  | 316 | 161 | .1 | .1 | 52.3 |
|  | 317 | 141 | .1 | .1 | 52.3 |
|  | 318 | 189 | .1 | .1 | 52.4 |
|  | 319 | 208 | .1 | .1 | 52.5 |
|  | 320 | 135 | .1 | .1 | 52.6 |
|  | 321 | 195 | .1 | .1 | 52.7 |
|  | 322 | 151 | .1 | .1 | 52.8 |
|  | 323 | 209 | .1 | .1 | 52.9 |
|  | 324 | 217 | .1 | .1 | 53.0 |
|  | 325 | 184 | .1 | .1 | 53.1 |
|  | 326 | 222 | .1 | .1 | 53.2 |
|  | 327 | 209 | .1 | .1 | 53.3 |
|  | 328 | 187 | .1 | .1 | 53.4 |
|  | 329 | 190 | .1 | .1 | 53.5 |
|  | 330 | 187 | .1 | .1 | 53.6 |
|  | 331 | 154 | .1 | .1 | 53.7 |
|  | 332 | 143 | .1 | .1 | 53.8 |
|  | 333 | 191 | .1 | .1 | 53.9 |
|  | 334 | 203 | .1 | .1 | 54.0 |
|  | 335 | 243 | .1 | .1 | 54.1 |
|  | 336 | 192 | .1 | .1 | 54.2 |
|  | 337 | 188 | .1 | .1 | 54.3 |
|  | 338 | 130 | .1 | .1 | 54.4 |
|  | 339 | 241 | .1 | .1 | 54.5 |
|  | 340 | 111 | .1 | .1 | 54.6 |
|  | 341 | 219 | .1 | .1 | 54.7 |
|  | 342 | 217 | .1 | .1 | 54.8 |
|  | 343 | 152 | .1 | .1 | 54.9 |
|  | 344 | 222 | .1 | .1 | 55.0 |
|  | 345 | 127 | .1 | .1 | 55.1 |
|  | 346 | 202 | .1 | .1 | 55.2 |
|  | 347 | 249 | .1 | .1 | 55.3 |
|  | 348 | 237 | .1 | .1 | 55.4 |
|  | 349 | 207 | .1 | .1 | 55.5 |
|  | 350 | 276 | .1 | .1 | 55.7 |
|  | 351 | 183 | .1 | .1 | 55.8 |
|  | 352 | 242 | .1 | .1 | 55.9 |
|  | 353 | 231 | .1 | .1 | 56.0 |
|  | 354 | 218 | .1 | .1 | 56.2 |
|  | 355 | 140 | .1 | .1 | 56.2 |
|  | 356 | 147 | .1 | .1 | 56.3 |
|  | 357 | 244 | .1 | .1 | 56.4 |
|  | 358 | 219 | .1 | .1 | 56.5 |
|  | 359 | 125 | .1 | .1 | 56.6 |
|  | 360 | 188 | .1 | .1 | 56.7 |
|  | 361 | 188 | .1 | .1 | 56.8 |
|  | 362 | 241 | .1 | .1 | 56.9 |
|  | 363 | 170 | .1 | .1 | 57.0 |
|  | 364 | 123 | .1 | .1 | 57.1 |
|  | 365 | 168 | .1 | .1 | 57.2 |
|  | 366 | 170 | .1 | .1 | 57.3 |
|  | 367 | 182 | .1 | .1 | 57.4 |
|  | 368 | 186 | .1 | .1 | 57.5 |
|  | 369 | 190 | .1 | .1 | 57.6 |
|  | 370 | 176 | .1 | .1 | 57.7 |
|  | 371 | 162 | .1 | .1 | 57.7 |
|  | 372 | 139 | .1 | .1 | 57.8 |
|  | 373 | 159 | .1 | .1 | 57.9 |
|  | 374 | 115 | .1 | .1 | 58.0 |
|  | 375 | 169 | .1 | .1 | 58.0 |
|  | 376 | 238 | .1 | .1 | 58.2 |
|  | 377 | 228 | .1 | .1 | 58.3 |
|  | 378 | 200 | .1 | .1 | 58.4 |
|  | 379 | 177 | .1 | .1 | 58.5 |
|  | 380 | 189 | .1 | .1 | 58.6 |
|  | 381 | 163 | .1 | .1 | 58.7 |
|  | 382 | 145 | .1 | .1 | 58.8 |
|  | 383 | 199 | .1 | .1 | 58.9 |
|  | 384 | 159 | .1 | .1 | 58.9 |
|  | 385 | 155 | .1 | .1 | 59.0 |
|  | 386 | 154 | .1 | .1 | 59.1 |
|  | 387 | 184 | .1 | .1 | 59.2 |
|  | 388 | 129 | .1 | .1 | 59.3 |
|  | 389 | 215 | .1 | .1 | 59.4 |
|  | 390 | 179 | .1 | .1 | 59.5 |
|  | 391 | 173 | .1 | .1 | 59.6 |
|  | 392 | 190 | .1 | .1 | 59.7 |
|  | 393 | 137 | .1 | .1 | 59.7 |
|  | 394 | 157 | .1 | .1 | 59.8 |
|  | 395 | 182 | .1 | .1 | 59.9 |
|  | 396 | 203 | .1 | .1 | 60.0 |
|  | 397 | 208 | .1 | .1 | 60.1 |
|  | 398 | 203 | .1 | .1 | 60.2 |
|  | 399 | 192 | .1 | .1 | 60.3 |
|  | 400 | 223 | .1 | .1 | 60.5 |
|  | 401 | 160 | .1 | .1 | 60.5 |
|  | 402 | 139 | .1 | .1 | 60.6 |
|  | 403 | 225 | .1 | .1 | 60.7 |
|  | 404 | 159 | .1 | .1 | 60.8 |
|  | 405 | 141 | .1 | .1 | 60.9 |
|  | 406 | 130 | .1 | .1 | 61.0 |
|  | 407 | 170 | .1 | .1 | 61.0 |
|  | 408 | 141 | .1 | .1 | 61.1 |
|  | 409 | 109 | .1 | .1 | 61.2 |
|  | 410 | 161 | .1 | .1 | 61.3 |
|  | 411 | 151 | .1 | .1 | 61.3 |
|  | 412 | 173 | .1 | .1 | 61.4 |
|  | 413 | 173 | .1 | .1 | 61.5 |
|  | 414 | 161 | .1 | .1 | 61.6 |
|  | 415 | 152 | .1 | .1 | 61.7 |
|  | 416 | 137 | .1 | .1 | 61.8 |
|  | 417 | 190 | .1 | .1 | 61.9 |
|  | 418 | 150 | .1 | .1 | 61.9 |
|  | 419 | 182 | .1 | .1 | 62.0 |
|  | 420 | 197 | .1 | .1 | 62.1 |
|  | 421 | 177 | .1 | .1 | 62.2 |
|  | 422 | 101 | .1 | .1 | 62.3 |
|  | 423 | 157 | .1 | .1 | 62.4 |
|  | 424 | 175 | .1 | .1 | 62.5 |
|  | 425 | 168 | .1 | .1 | 62.5 |
|  | 426 | 153 | .1 | .1 | 62.6 |
|  | 427 | 186 | .1 | .1 | 62.7 |
|  | 428 | 224 | .1 | .1 | 62.8 |
|  | 429 | 152 | .1 | .1 | 62.9 |
|  | 430 | 142 | .1 | .1 | 63.0 |
|  | 431 | 172 | .1 | .1 | 63.1 |
|  | 432 | 161 | .1 | .1 | 63.2 |
|  | 433 | 132 | .1 | .1 | 63.2 |
|  | 434 | 133 | .1 | .1 | 63.3 |
|  | 435 | 193 | .1 | .1 | 63.4 |
|  | 436 | 155 | .1 | .1 | 63.5 |
|  | 437 | 184 | .1 | .1 | 63.6 |
|  | 438 | 193 | .1 | .1 | 63.7 |
|  | 439 | 129 | .1 | .1 | 63.8 |
|  | 440 | 179 | .1 | .1 | 63.9 |
|  | 441 | 231 | .1 | .1 | 64.0 |
|  | 442 | 195 | .1 | .1 | 64.1 |
|  | 443 | 196 | .1 | .1 | 64.2 |
|  | 444 | 159 | .1 | .1 | 64.3 |
|  | 445 | 207 | .1 | .1 | 64.4 |
|  | 446 | 159 | .1 | .1 | 64.5 |
|  | 447 | 191 | .1 | .1 | 64.6 |
|  | 448 | 131 | .1 | .1 | 64.6 |
|  | 449 | 136 | .1 | .1 | 64.7 |
|  | 450 | 158 | .1 | .1 | 64.8 |
|  | 451 | 153 | .1 | .1 | 64.9 |
|  | 452 | 187 | .1 | .1 | 65.0 |
|  | 453 | 92 | .0 | .0 | 65.0 |
|  | 454 | 154 | .1 | .1 | 65.1 |
|  | 455 | 146 | .1 | .1 | 65.2 |
|  | 456 | 123 | .1 | .1 | 65.2 |
|  | 457 | 85 | .0 | .0 | 65.3 |
|  | 458 | 134 | .1 | .1 | 65.3 |
|  | 459 | 174 | .1 | .1 | 65.4 |
|  | 460 | 120 | .1 | .1 | 65.5 |
|  | 461 | 147 | .1 | .1 | 65.6 |
|  | 462 | 108 | .1 | .1 | 65.6 |
|  | 463 | 108 | .1 | .1 | 65.7 |
|  | 464 | 168 | .1 | .1 | 65.8 |
|  | 465 | 147 | .1 | .1 | 65.9 |
|  | 466 | 162 | .1 | .1 | 65.9 |
|  | 467 | 142 | .1 | .1 | 66.0 |
|  | 468 | 174 | .1 | .1 | 66.1 |
|  | 469 | 180 | .1 | .1 | 66.2 |
|  | 470 | 157 | .1 | .1 | 66.3 |
|  | 471 | 142 | .1 | .1 | 66.4 |
|  | 472 | 129 | .1 | .1 | 66.4 |
|  | 473 | 141 | .1 | .1 | 66.5 |
|  | 474 | 142 | .1 | .1 | 66.6 |
|  | 475 | 193 | .1 | .1 | 66.7 |
|  | 476 | 97 | .1 | .1 | 66.7 |
|  | 477 | 144 | .1 | .1 | 66.8 |
|  | 478 | 118 | .1 | .1 | 66.9 |
|  | 479 | 126 | .1 | .1 | 66.9 |
|  | 480 | 205 | .1 | .1 | 67.0 |
|  | 481 | 82 | .0 | .0 | 67.1 |
|  | 482 | 177 | .1 | .1 | 67.2 |
|  | 483 | 153 | .1 | .1 | 67.3 |
|  | 484 | 118 | .1 | .1 | 67.3 |
|  | 485 | 129 | .1 | .1 | 67.4 |
|  | 486 | 140 | .1 | .1 | 67.5 |
|  | 487 | 161 | .1 | .1 | 67.5 |
|  | 488 | 99 | .1 | .1 | 67.6 |
|  | 489 | 140 | .1 | .1 | 67.7 |
|  | 490 | 150 | .1 | .1 | 67.7 |
|  | 491 | 149 | .1 | .1 | 67.8 |
|  | 492 | 107 | .1 | .1 | 67.9 |
|  | 493 | 177 | .1 | .1 | 68.0 |
|  | 494 | 181 | .1 | .1 | 68.1 |
|  | 495 | 137 | .1 | .1 | 68.1 |
|  | 496 | 155 | .1 | .1 | 68.2 |
|  | 497 | 113 | .1 | .1 | 68.3 |
|  | 498 | 133 | .1 | .1 | 68.4 |
|  | 499 | 139 | .1 | .1 | 68.4 |
|  | 500 | 124 | .1 | .1 | 68.5 |
|  | 501 | 105 | .1 | .1 | 68.5 |
|  | 502 | 96 | .1 | .1 | 68.6 |
|  | 503 | 153 | .1 | .1 | 68.7 |
|  | 504 | 126 | .1 | .1 | 68.7 |
|  | 505 | 103 | .1 | .1 | 68.8 |
|  | 506 | 106 | .1 | .1 | 68.9 |
|  | 507 | 173 | .1 | .1 | 68.9 |
|  | 508 | 76 | .0 | .0 | 69.0 |
|  | 509 | 125 | .1 | .1 | 69.0 |
|  | 510 | 92 | .0 | .0 | 69.1 |
|  | 511 | 113 | .1 | .1 | 69.2 |
|  | 512 | 115 | .1 | .1 | 69.2 |
|  | 513 | 116 | .1 | .1 | 69.3 |
|  | 514 | 51 | .0 | .0 | 69.3 |
|  | 515 | 118 | .1 | .1 | 69.4 |
|  | 516 | 81 | .0 | .0 | 69.4 |
|  | 517 | 186 | .1 | .1 | 69.5 |
|  | 518 | 125 | .1 | .1 | 69.6 |
|  | 519 | 145 | .1 | .1 | 69.6 |
|  | 520 | 87 | .0 | .0 | 69.7 |
|  | 521 | 152 | .1 | .1 | 69.8 |
|  | 522 | 161 | .1 | .1 | 69.9 |
|  | 523 | 126 | .1 | .1 | 69.9 |
|  | 524 | 92 | .0 | .0 | 70.0 |
|  | 525 | 102 | .1 | .1 | 70.0 |
|  | 526 | 93 | .0 | .0 | 70.1 |
|  | 527 | 107 | .1 | .1 | 70.1 |
|  | 528 | 153 | .1 | .1 | 70.2 |
|  | 529 | 88 | .0 | .0 | 70.3 |
|  | 530 | 83 | .0 | .0 | 70.3 |
|  | 531 | 91 | .0 | .0 | 70.4 |
|  | 532 | 83 | .0 | .0 | 70.4 |
|  | 533 | 146 | .1 | .1 | 70.5 |
|  | 534 | 95 | .0 | .0 | 70.5 |
|  | 535 | 132 | .1 | .1 | 70.6 |
|  | 536 | 82 | .0 | .0 | 70.6 |
|  | 537 | 112 | .1 | .1 | 70.7 |
|  | 538 | 98 | .1 | .1 | 70.7 |
|  | 539 | 71 | .0 | .0 | 70.8 |
|  | 540 | 120 | .1 | .1 | 70.8 |
|  | 541 | 118 | .1 | .1 | 70.9 |
|  | 542 | 129 | .1 | .1 | 71.0 |
|  | 543 | 81 | .0 | .0 | 71.0 |
|  | 544 | 130 | .1 | .1 | 71.1 |
|  | 545 | 118 | .1 | .1 | 71.1 |
|  | 546 | 152 | .1 | .1 | 71.2 |
|  | 547 | 115 | .1 | .1 | 71.3 |
|  | 548 | 92 | .0 | .0 | 71.3 |
|  | 549 | 98 | .1 | .1 | 71.4 |
|  | 550 | 150 | .1 | .1 | 71.5 |
|  | 551 | 82 | .0 | .0 | 71.5 |
|  | 552 | 88 | .0 | .0 | 71.6 |
|  | 553 | 85 | .0 | .0 | 71.6 |
|  | 554 | 118 | .1 | .1 | 71.7 |
|  | 555 | 120 | .1 | .1 | 71.7 |
|  | 556 | 125 | .1 | .1 | 71.8 |
|  | 557 | 104 | .1 | .1 | 71.8 |
|  | 558 | 79 | .0 | .0 | 71.9 |
|  | 559 | 87 | .0 | .0 | 71.9 |
|  | 560 | 99 | .1 | .1 | 72.0 |
|  | 561 | 66 | .0 | .0 | 72.0 |
|  | 562 | 88 | .0 | .0 | 72.1 |
|  | 563 | 105 | .1 | .1 | 72.1 |
|  | 564 | 89 | .0 | .0 | 72.2 |
|  | 565 | 118 | .1 | .1 | 72.2 |
|  | 566 | 86 | .0 | .0 | 72.3 |
|  | 567 | 59 | .0 | .0 | 72.3 |
|  | 568 | 107 | .1 | .1 | 72.4 |
|  | 569 | 106 | .1 | .1 | 72.4 |
|  | 570 | 39 | .0 | .0 | 72.4 |
|  | 571 | 95 | .0 | .0 | 72.5 |
|  | 572 | 124 | .1 | .1 | 72.6 |
|  | 573 | 118 | .1 | .1 | 72.6 |
|  | 574 | 103 | .1 | .1 | 72.7 |
|  | 575 | 76 | .0 | .0 | 72.7 |
|  | 576 | 91 | .0 | .0 | 72.8 |
|  | 577 | 107 | .1 | .1 | 72.8 |
|  | 578 | 114 | .1 | .1 | 72.9 |
|  | 579 | 102 | .1 | .1 | 72.9 |
|  | 580 | 106 | .1 | .1 | 73.0 |
|  | 581 | 107 | .1 | .1 | 73.0 |
|  | 582 | 41 | .0 | .0 | 73.1 |
|  | 583 | 97 | .1 | .1 | 73.1 |
|  | 584 | 146 | .1 | .1 | 73.2 |
|  | 585 | 98 | .1 | .1 | 73.2 |
|  | 586 | 113 | .1 | .1 | 73.3 |
|  | 587 | 76 | .0 | .0 | 73.3 |
|  | 588 | 92 | .0 | .0 | 73.4 |
|  | 589 | 88 | .0 | .0 | 73.4 |
|  | 590 | 99 | .1 | .1 | 73.5 |
|  | 591 | 130 | .1 | .1 | 73.6 |
|  | 592 | 68 | .0 | .0 | 73.6 |
|  | 593 | 101 | .1 | .1 | 73.6 |
|  | 594 | 93 | .0 | .0 | 73.7 |
|  | 595 | 99 | .1 | .1 | 73.7 |
|  | 596 | 102 | .1 | .1 | 73.8 |
|  | 597 | 71 | .0 | .0 | 73.8 |
|  | 598 | 87 | .0 | .0 | 73.9 |
|  | 599 | 126 | .1 | .1 | 73.9 |
|  | 600 | 101 | .1 | .1 | 74.0 |
|  | 601 | 131 | .1 | .1 | 74.1 |
|  | 602 | 129 | .1 | .1 | 74.1 |
|  | 603 | 135 | .1 | .1 | 74.2 |
|  | 604 | 124 | .1 | .1 | 74.3 |
|  | 605 | 74 | .0 | .0 | 74.3 |
|  | 606 | 136 | .1 | .1 | 74.4 |
|  | 607 | 169 | .1 | .1 | 74.5 |
|  | 608 | 91 | .0 | .0 | 74.5 |
|  | 609 | 95 | .0 | .0 | 74.6 |
|  | 610 | 127 | .1 | .1 | 74.6 |
|  | 611 | 82 | .0 | .0 | 74.7 |
|  | 612 | 116 | .1 | .1 | 74.7 |
|  | 613 | 129 | .1 | .1 | 74.8 |
|  | 614 | 82 | .0 | .0 | 74.9 |
|  | 615 | 108 | .1 | .1 | 74.9 |
|  | 616 | 98 | .1 | .1 | 75.0 |
|  | 617 | 140 | .1 | .1 | 75.0 |
|  | 618 | 111 | .1 | .1 | 75.1 |
|  | 619 | 137 | .1 | .1 | 75.2 |
|  | 620 | 74 | .0 | .0 | 75.2 |
|  | 621 | 85 | .0 | .0 | 75.2 |
|  | 622 | 57 | .0 | .0 | 75.3 |
|  | 623 | 99 | .1 | .1 | 75.3 |
|  | 624 | 122 | .1 | .1 | 75.4 |
|  | 625 | 92 | .0 | .0 | 75.4 |
|  | 626 | 99 | .1 | .1 | 75.5 |
|  | 627 | 99 | .1 | .1 | 75.5 |
|  | 628 | 74 | .0 | .0 | 75.6 |
|  | 629 | 96 | .1 | .1 | 75.6 |
|  | 630 | 126 | .1 | .1 | 75.7 |
|  | 631 | 55 | .0 | .0 | 75.7 |
|  | 632 | 127 | .1 | .1 | 75.8 |
|  | 633 | 68 | .0 | .0 | 75.8 |
|  | 634 | 82 | .0 | .0 | 75.9 |
|  | 635 | 122 | .1 | .1 | 75.9 |
|  | 636 | 90 | .0 | .0 | 76.0 |
|  | 637 | 124 | .1 | .1 | 76.1 |
|  | 638 | 115 | .1 | .1 | 76.1 |
|  | 639 | 89 | .0 | .0 | 76.2 |
|  | 640 | 150 | .1 | .1 | 76.2 |
|  | 641 | 98 | .1 | .1 | 76.3 |
|  | 642 | 60 | .0 | .0 | 76.3 |
|  | 643 | 87 | .0 | .0 | 76.4 |
|  | 644 | 116 | .1 | .1 | 76.4 |
|  | 645 | 100 | .1 | .1 | 76.5 |
|  | 646 | 130 | .1 | .1 | 76.5 |
|  | 647 | 87 | .0 | .0 | 76.6 |
|  | 648 | 137 | .1 | .1 | 76.7 |
|  | 649 | 55 | .0 | .0 | 76.7 |
|  | 650 | 108 | .1 | .1 | 76.8 |
|  | 651 | 84 | .0 | .0 | 76.8 |
|  | 652 | 84 | .0 | .0 | 76.8 |
|  | 653 | 121 | .1 | .1 | 76.9 |
|  | 654 | 120 | .1 | .1 | 77.0 |
|  | 655 | 108 | .1 | .1 | 77.0 |
|  | 656 | 71 | .0 | .0 | 77.1 |
|  | 657 | 63 | .0 | .0 | 77.1 |
|  | 658 | 79 | .0 | .0 | 77.1 |
|  | 659 | 110 | .1 | .1 | 77.2 |
|  | 660 | 151 | .1 | .1 | 77.3 |
|  | 661 | 105 | .1 | .1 | 77.3 |
|  | 662 | 94 | .0 | .0 | 77.4 |
|  | 663 | 127 | .1 | .1 | 77.4 |
|  | 664 | 143 | .1 | .1 | 77.5 |
|  | 665 | 125 | .1 | .1 | 77.6 |
|  | 666 | 112 | .1 | .1 | 77.6 |
|  | 667 | 96 | .1 | .1 | 77.7 |
|  | 668 | 100 | .1 | .1 | 77.7 |
|  | 669 | 98 | .1 | .1 | 77.8 |
|  | 670 | 86 | .0 | .0 | 77.8 |
|  | 671 | 106 | .1 | .1 | 77.9 |
|  | 672 | 103 | .1 | .1 | 78.0 |
|  | 673 | 66 | .0 | .0 | 78.0 |
|  | 674 | 47 | .0 | .0 | 78.0 |
|  | 675 | 64 | .0 | .0 | 78.0 |
|  | 676 | 64 | .0 | .0 | 78.1 |
|  | 677 | 97 | .1 | .1 | 78.1 |
|  | 678 | 66 | .0 | .0 | 78.2 |
|  | 679 | 92 | .0 | .0 | 78.2 |
|  | 680 | 89 | .0 | .0 | 78.3 |
|  | 681 | 158 | .1 | .1 | 78.3 |
|  | 682 | 101 | .1 | .1 | 78.4 |
|  | 683 | 71 | .0 | .0 | 78.4 |
|  | 684 | 79 | .0 | .0 | 78.5 |
|  | 685 | 102 | .1 | .1 | 78.5 |
|  | 686 | 90 | .0 | .0 | 78.6 |
|  | 687 | 45 | .0 | .0 | 78.6 |
|  | 688 | 76 | .0 | .0 | 78.6 |
|  | 689 | 85 | .0 | .0 | 78.7 |
|  | 690 | 60 | .0 | .0 | 78.7 |
|  | 691 | 100 | .1 | .1 | 78.8 |
|  | 692 | 87 | .0 | .0 | 78.8 |
|  | 693 | 159 | .1 | .1 | 78.9 |
|  | 694 | 74 | .0 | .0 | 78.9 |
|  | 695 | 58 | .0 | .0 | 79.0 |
|  | 696 | 49 | .0 | .0 | 79.0 |
|  | 697 | 67 | .0 | .0 | 79.0 |
|  | 698 | 73 | .0 | .0 | 79.1 |
|  | 699 | 83 | .0 | .0 | 79.1 |
|  | 700 | 70 | .0 | .0 | 79.1 |
|  | 701 | 98 | .1 | .1 | 79.2 |
|  | 702 | 70 | .0 | .0 | 79.2 |
|  | 703 | 37 | .0 | .0 | 79.3 |
|  | 704 | 76 | .0 | .0 | 79.3 |
|  | 705 | 45 | .0 | .0 | 79.3 |
|  | 706 | 57 | .0 | .0 | 79.3 |
|  | 707 | 53 | .0 | .0 | 79.4 |
|  | 708 | 70 | .0 | .0 | 79.4 |
|  | 709 | 53 | .0 | .0 | 79.4 |
|  | 710 | 75 | .0 | .0 | 79.5 |
|  | 711 | 93 | .0 | .0 | 79.5 |
|  | 712 | 82 | .0 | .0 | 79.6 |
|  | 713 | 105 | .1 | .1 | 79.6 |
|  | 714 | 50 | .0 | .0 | 79.7 |
|  | 715 | 125 | .1 | .1 | 79.7 |
|  | 716 | 114 | .1 | .1 | 79.8 |
|  | 717 | 76 | .0 | .0 | 79.8 |
|  | 718 | 107 | .1 | .1 | 79.9 |
|  | 719 | 88 | .0 | .0 | 79.9 |
|  | 720 | 80 | .0 | .0 | 80.0 |
|  | 721 | 125 | .1 | .1 | 80.0 |
|  | 722 | 97 | .1 | .1 | 80.1 |
|  | 723 | 80 | .0 | .0 | 80.1 |
|  | 724 | 46 | .0 | .0 | 80.1 |
|  | 725 | 68 | .0 | .0 | 80.2 |
|  | 726 | 135 | .1 | .1 | 80.3 |
|  | 727 | 92 | .0 | .0 | 80.3 |
|  | 728 | 87 | .0 | .0 | 80.3 |
|  | 729 | 34 | .0 | .0 | 80.4 |
|  | 730 | 54 | .0 | .0 | 80.4 |
|  | 731 | 43 | .0 | .0 | 80.4 |
|  | 732 | 68 | .0 | .0 | 80.5 |
|  | 733 | 92 | .0 | .0 | 80.5 |
|  | 734 | 65 | .0 | .0 | 80.5 |
|  | 735 | 70 | .0 | .0 | 80.6 |
|  | 736 | 39 | .0 | .0 | 80.6 |
|  | 737 | 65 | .0 | .0 | 80.6 |
|  | 738 | 62 | .0 | .0 | 80.7 |
|  | 739 | 64 | .0 | .0 | 80.7 |
|  | 740 | 71 | .0 | .0 | 80.7 |
|  | 741 | 109 | .1 | .1 | 80.8 |
|  | 742 | 84 | .0 | .0 | 80.8 |
|  | 743 | 55 | .0 | .0 | 80.9 |
|  | 744 | 85 | .0 | .0 | 80.9 |
|  | 745 | 101 | .1 | .1 | 81.0 |
|  | 746 | 81 | .0 | .0 | 81.0 |
|  | 747 | 116 | .1 | .1 | 81.1 |
|  | 748 | 78 | .0 | .0 | 81.1 |
|  | 749 | 29 | .0 | .0 | 81.1 |
|  | 750 | 100 | .1 | .1 | 81.2 |
|  | 751 | 47 | .0 | .0 | 81.2 |
|  | 752 | 38 | .0 | .0 | 81.2 |
|  | 753 | 115 | .1 | .1 | 81.3 |
|  | 754 | 92 | .0 | .0 | 81.3 |
|  | 755 | 120 | .1 | .1 | 81.4 |
|  | 756 | 101 | .1 | .1 | 81.4 |
|  | 757 | 40 | .0 | .0 | 81.5 |
|  | 758 | 20 | .0 | .0 | 81.5 |
|  | 759 | 71 | .0 | .0 | 81.5 |
|  | 760 | 71 | .0 | .0 | 81.5 |
|  | 761 | 71 | .0 | .0 | 81.6 |
|  | 762 | 55 | .0 | .0 | 81.6 |
|  | 763 | 100 | .1 | .1 | 81.7 |
|  | 764 | 69 | .0 | .0 | 81.7 |
|  | 765 | 40 | .0 | .0 | 81.7 |
|  | 766 | 58 | .0 | .0 | 81.8 |
|  | 767 | 52 | .0 | .0 | 81.8 |
|  | 768 | 43 | .0 | .0 | 81.8 |
|  | 769 | 19 | .0 | .0 | 81.8 |
|  | 770 | 115 | .1 | .1 | 81.9 |
|  | 771 | 51 | .0 | .0 | 81.9 |
|  | 772 | 55 | .0 | .0 | 81.9 |
|  | 773 | 24 | .0 | .0 | 81.9 |
|  | 774 | 54 | .0 | .0 | 82.0 |
|  | 775 | 77 | .0 | .0 | 82.0 |
|  | 776 | 51 | .0 | .0 | 82.0 |
|  | 777 | 43 | .0 | .0 | 82.1 |
|  | 778 | 112 | .1 | .1 | 82.1 |
|  | 779 | 65 | .0 | .0 | 82.2 |
|  | 780 | 55 | .0 | .0 | 82.2 |
|  | 781 | 97 | .1 | .1 | 82.2 |
|  | 782 | 98 | .1 | .1 | 82.3 |
|  | 783 | 64 | .0 | .0 | 82.3 |
|  | 784 | 54 | .0 | .0 | 82.3 |
|  | 785 | 127 | .1 | .1 | 82.4 |
|  | 786 | 57 | .0 | .0 | 82.4 |
|  | 787 | 73 | .0 | .0 | 82.5 |
|  | 788 | 93 | .0 | .0 | 82.5 |
|  | 789 | 69 | .0 | .0 | 82.6 |
|  | 790 | 71 | .0 | .0 | 82.6 |
|  | 791 | 73 | .0 | .0 | 82.6 |
|  | 792 | 59 | .0 | .0 | 82.7 |
|  | 793 | 78 | .0 | .0 | 82.7 |
|  | 794 | 38 | .0 | .0 | 82.7 |
|  | 795 | 68 | .0 | .0 | 82.8 |
|  | 796 | 48 | .0 | .0 | 82.8 |
|  | 797 | 63 | .0 | .0 | 82.8 |
|  | 798 | 53 | .0 | .0 | 82.9 |
|  | 799 | 22 | .0 | .0 | 82.9 |
|  | 800 | 43 | .0 | .0 | 82.9 |
|  | 801 | 63 | .0 | .0 | 82.9 |
|  | 802 | 54 | .0 | .0 | 83.0 |
|  | 803 | 37 | .0 | .0 | 83.0 |
|  | 804 | 43 | .0 | .0 | 83.0 |
|  | 805 | 95 | .0 | .0 | 83.0 |
|  | 806 | 94 | .0 | .0 | 83.1 |
|  | 807 | 66 | .0 | .0 | 83.1 |
|  | 808 | 50 | .0 | .0 | 83.2 |
|  | 809 | 49 | .0 | .0 | 83.2 |
|  | 810 | 34 | .0 | .0 | 83.2 |
|  | 811 | 64 | .0 | .0 | 83.2 |
|  | 812 | 52 | .0 | .0 | 83.3 |
|  | 813 | 59 | .0 | .0 | 83.3 |
|  | 814 | 91 | .0 | .0 | 83.3 |
|  | 815 | 86 | .0 | .0 | 83.4 |
|  | 816 | 109 | .1 | .1 | 83.4 |
|  | 817 | 37 | .0 | .0 | 83.5 |
|  | 818 | 58 | .0 | .0 | 83.5 |
|  | 819 | 56 | .0 | .0 | 83.5 |
|  | 820 | 93 | .0 | .0 | 83.6 |
|  | 821 | 57 | .0 | .0 | 83.6 |
|  | 822 | 55 | .0 | .0 | 83.6 |
|  | 823 | 34 | .0 | .0 | 83.6 |
|  | 824 | 65 | .0 | .0 | 83.7 |
|  | 825 | 67 | .0 | .0 | 83.7 |
|  | 826 | 68 | .0 | .0 | 83.7 |
|  | 827 | 37 | .0 | .0 | 83.8 |
|  | 828 | 66 | .0 | .0 | 83.8 |
|  | 829 | 52 | .0 | .0 | 83.8 |
|  | 830 | 81 | .0 | .0 | 83.9 |
|  | 831 | 46 | .0 | .0 | 83.9 |
|  | 832 | 74 | .0 | .0 | 83.9 |
|  | 833 | 45 | .0 | .0 | 84.0 |
|  | 834 | 72 | .0 | .0 | 84.0 |
|  | 835 | 58 | .0 | .0 | 84.0 |
|  | 836 | 63 | .0 | .0 | 84.1 |
|  | 837 | 48 | .0 | .0 | 84.1 |
|  | 838 | 63 | .0 | .0 | 84.1 |
|  | 839 | 27 | .0 | .0 | 84.1 |
|  | 840 | 51 | .0 | .0 | 84.2 |
|  | 841 | 88 | .0 | .0 | 84.2 |
|  | 842 | 91 | .0 | .0 | 84.3 |
|  | 843 | 76 | .0 | .0 | 84.3 |
|  | 844 | 68 | .0 | .0 | 84.3 |
|  | 845 | 81 | .0 | .0 | 84.4 |
|  | 846 | 56 | .0 | .0 | 84.4 |
|  | 847 | 67 | .0 | .0 | 84.4 |
|  | 848 | 72 | .0 | .0 | 84.5 |
|  | 849 | 45 | .0 | .0 | 84.5 |
|  | 850 | 56 | .0 | .0 | 84.5 |
|  | 851 | 78 | .0 | .0 | 84.6 |
|  | 852 | 91 | .0 | .0 | 84.6 |
|  | 853 | 56 | .0 | .0 | 84.6 |
|  | 854 | 59 | .0 | .0 | 84.7 |
|  | 855 | 61 | .0 | .0 | 84.7 |
|  | 856 | 46 | .0 | .0 | 84.7 |
|  | 857 | 51 | .0 | .0 | 84.8 |
|  | 858 | 52 | .0 | .0 | 84.8 |
|  | 859 | 47 | .0 | .0 | 84.8 |
|  | 860 | 51 | .0 | .0 | 84.8 |
|  | 861 | 37 | .0 | .0 | 84.9 |
|  | 862 | 44 | .0 | .0 | 84.9 |
|  | 863 | 46 | .0 | .0 | 84.9 |
|  | 864 | 69 | .0 | .0 | 84.9 |
|  | 865 | 59 | .0 | .0 | 85.0 |
|  | 866 | 37 | .0 | .0 | 85.0 |
|  | 867 | 65 | .0 | .0 | 85.0 |
|  | 868 | 51 | .0 | .0 | 85.1 |
|  | 869 | 57 | .0 | .0 | 85.1 |
|  | 870 | 74 | .0 | .0 | 85.1 |
|  | 871 | 62 | .0 | .0 | 85.2 |
|  | 872 | 55 | .0 | .0 | 85.2 |
|  | 873 | 57 | .0 | .0 | 85.2 |
|  | 874 | 67 | .0 | .0 | 85.2 |
|  | 875 | 81 | .0 | .0 | 85.3 |
|  | 876 | 40 | .0 | .0 | 85.3 |
|  | 877 | 55 | .0 | .0 | 85.3 |
|  | 878 | 57 | .0 | .0 | 85.4 |
|  | 879 | 59 | .0 | .0 | 85.4 |
|  | 880 | 58 | .0 | .0 | 85.4 |
|  | 881 | 31 | .0 | .0 | 85.4 |
|  | 882 | 61 | .0 | .0 | 85.5 |
|  | 883 | 27 | .0 | .0 | 85.5 |
|  | 884 | 48 | .0 | .0 | 85.5 |
|  | 885 | 41 | .0 | .0 | 85.5 |
|  | 886 | 83 | .0 | .0 | 85.6 |
|  | 887 | 52 | .0 | .0 | 85.6 |
|  | 888 | 85 | .0 | .0 | 85.7 |
|  | 889 | 71 | .0 | .0 | 85.7 |
|  | 890 | 53 | .0 | .0 | 85.7 |
|  | 891 | 55 | .0 | .0 | 85.8 |
|  | 892 | 71 | .0 | .0 | 85.8 |
|  | 893 | 89 | .0 | .0 | 85.8 |
|  | 894 | 33 | .0 | .0 | 85.9 |
|  | 895 | 37 | .0 | .0 | 85.9 |
|  | 896 | 47 | .0 | .0 | 85.9 |
|  | 897 | 48 | .0 | .0 | 85.9 |
|  | 898 | 46 | .0 | .0 | 85.9 |
|  | 899 | 61 | .0 | .0 | 86.0 |
|  | 900 | 41 | .0 | .0 | 86.0 |
|  | 901 | 54 | .0 | .0 | 86.0 |
|  | 902 | 35 | .0 | .0 | 86.0 |
|  | 903 | 46 | .0 | .0 | 86.1 |
|  | 904 | 76 | .0 | .0 | 86.1 |
|  | 905 | 30 | .0 | .0 | 86.1 |
|  | 906 | 99 | .1 | .1 | 86.2 |
|  | 907 | 80 | .0 | .0 | 86.2 |
|  | 908 | 29 | .0 | .0 | 86.2 |
|  | 909 | 88 | .0 | .0 | 86.3 |
|  | 910 | 77 | .0 | .0 | 86.3 |
|  | 911 | 51 | .0 | .0 | 86.4 |
|  | 912 | 26 | .0 | .0 | 86.4 |
|  | 913 | 47 | .0 | .0 | 86.4 |
|  | 914 | 25 | .0 | .0 | 86.4 |
|  | 915 | 54 | .0 | .0 | 86.4 |
|  | 916 | 51 | .0 | .0 | 86.5 |
|  | 917 | 36 | .0 | .0 | 86.5 |
|  | 918 | 21 | .0 | .0 | 86.5 |
|  | 919 | 56 | .0 | .0 | 86.5 |
|  | 920 | 44 | .0 | .0 | 86.5 |
|  | 921 | 67 | .0 | .0 | 86.6 |
|  | 922 | 60 | .0 | .0 | 86.6 |
|  | 923 | 18 | .0 | .0 | 86.6 |
|  | 924 | 61 | .0 | .0 | 86.6 |
|  | 925 | 37 | .0 | .0 | 86.7 |
|  | 926 | 53 | .0 | .0 | 86.7 |
|  | 927 | 99 | .1 | .1 | 86.7 |
|  | 928 | 62 | .0 | .0 | 86.8 |
|  | 929 | 72 | .0 | .0 | 86.8 |
|  | 930 | 55 | .0 | .0 | 86.8 |
|  | 931 | 51 | .0 | .0 | 86.9 |
|  | 932 | 73 | .0 | .0 | 86.9 |
|  | 933 | 67 | .0 | .0 | 86.9 |
|  | 934 | 33 | .0 | .0 | 87.0 |
|  | 935 | 28 | .0 | .0 | 87.0 |
|  | 936 | 75 | .0 | .0 | 87.0 |
|  | 937 | 47 | .0 | .0 | 87.0 |
|  | 938 | 57 | .0 | .0 | 87.1 |
|  | 939 | 57 | .0 | .0 | 87.1 |
|  | 940 | 66 | .0 | .0 | 87.1 |
|  | 941 | 44 | .0 | .0 | 87.2 |
|  | 942 | 55 | .0 | .0 | 87.2 |
|  | 943 | 50 | .0 | .0 | 87.2 |
|  | 944 | 69 | .0 | .0 | 87.3 |
|  | 945 | 69 | .0 | .0 | 87.3 |
|  | 946 | 22 | .0 | .0 | 87.3 |
|  | 947 | 50 | .0 | .0 | 87.3 |
|  | 948 | 44 | .0 | .0 | 87.3 |
|  | 949 | 47 | .0 | .0 | 87.4 |
|  | 950 | 21 | .0 | .0 | 87.4 |
|  | 951 | 35 | .0 | .0 | 87.4 |
|  | 952 | 28 | .0 | .0 | 87.4 |
|  | 953 | 34 | .0 | .0 | 87.4 |
|  | 954 | 39 | .0 | .0 | 87.5 |
|  | 955 | 17 | .0 | .0 | 87.5 |
|  | 956 | 25 | .0 | .0 | 87.5 |
|  | 957 | 35 | .0 | .0 | 87.5 |
|  | 958 | 29 | .0 | .0 | 87.5 |
|  | 959 | 12 | .0 | .0 | 87.5 |
|  | 960 | 28 | .0 | .0 | 87.5 |
|  | 961 | 51 | .0 | .0 | 87.6 |
|  | 962 | 24 | .0 | .0 | 87.6 |
|  | 963 | 49 | .0 | .0 | 87.6 |
|  | 964 | 39 | .0 | .0 | 87.6 |
|  | 965 | 59 | .0 | .0 | 87.6 |
|  | 966 | 60 | .0 | .0 | 87.7 |
|  | 967 | 37 | .0 | .0 | 87.7 |
|  | 968 | 20 | .0 | .0 | 87.7 |
|  | 969 | 32 | .0 | .0 | 87.7 |
|  | 970 | 50 | .0 | .0 | 87.8 |
|  | 971 | 56 | .0 | .0 | 87.8 |
|  | 972 | 40 | .0 | .0 | 87.8 |
|  | 973 | 36 | .0 | .0 | 87.8 |
|  | 974 | 33 | .0 | .0 | 87.8 |
|  | 975 | 47 | .0 | .0 | 87.9 |
|  | 976 | 24 | .0 | .0 | 87.9 |
|  | 977 | 62 | .0 | .0 | 87.9 |
|  | 978 | 31 | .0 | .0 | 87.9 |
|  | 979 | 27 | .0 | .0 | 87.9 |
|  | 980 | 68 | .0 | .0 | 88.0 |
|  | 981 | 32 | .0 | .0 | 88.0 |
|  | 982 | 48 | .0 | .0 | 88.0 |
|  | 983 | 39 | .0 | .0 | 88.0 |
|  | 984 | 51 | .0 | .0 | 88.1 |
|  | 985 | 32 | .0 | .0 | 88.1 |
|  | 986 | 18 | .0 | .0 | 88.1 |
|  | 987 | 64 | .0 | .0 | 88.1 |
|  | 988 | 29 | .0 | .0 | 88.1 |
|  | 989 | 22 | .0 | .0 | 88.2 |
|  | 990 | 28 | .0 | .0 | 88.2 |
|  | 991 | 55 | .0 | .0 | 88.2 |
|  | 992 | 25 | .0 | .0 | 88.2 |
|  | 993 | 33 | .0 | .0 | 88.2 |
|  | 994 | 34 | .0 | .0 | 88.2 |
|  | 995 | 75 | .0 | .0 | 88.3 |
|  | 996 | 29 | .0 | .0 | 88.3 |
|  | 997 | 24 | .0 | .0 | 88.3 |
|  | 998 | 34 | .0 | .0 | 88.3 |
|  | 999 | 13 | .0 | .0 | 88.3 |
|  | 1000 | 31 | .0 | .0 | 88.4 |
|  | 1001 | 7 | .0 | .0 | 88.4 |
|  | 1002 | 33 | .0 | .0 | 88.4 |
|  | 1003 | 23 | .0 | .0 | 88.4 |
|  | 1004 | 54 | .0 | .0 | 88.4 |
|  | 1005 | 48 | .0 | .0 | 88.4 |
|  | 1006 | 35 | .0 | .0 | 88.5 |
|  | 1007 | 30 | .0 | .0 | 88.5 |
|  | 1008 | 25 | .0 | .0 | 88.5 |
|  | 1009 | 32 | .0 | .0 | 88.5 |
|  | 1010 | 7 | .0 | .0 | 88.5 |
|  | 1011 | 29 | .0 | .0 | 88.5 |
|  | 1012 | 58 | .0 | .0 | 88.6 |
|  | 1013 | 37 | .0 | .0 | 88.6 |
|  | 1014 | 26 | .0 | .0 | 88.6 |
|  | 1015 | 68 | .0 | .0 | 88.6 |
|  | 1016 | 38 | .0 | .0 | 88.6 |
|  | 1017 | 21 | .0 | .0 | 88.7 |
|  | 1018 | 15 | .0 | .0 | 88.7 |
|  | 1019 | 32 | .0 | .0 | 88.7 |
|  | 1020 | 12 | .0 | .0 | 88.7 |
|  | 1021 | 8 | .0 | .0 | 88.7 |
|  | 1022 | 25 | .0 | .0 | 88.7 |
|  | 1023 | 68 | .0 | .0 | 88.7 |
|  | 1024 | 31 | .0 | .0 | 88.8 |
|  | 1025 | 21 | .0 | .0 | 88.8 |
|  | 1026 | 28 | .0 | .0 | 88.8 |
|  | 1027 | 55 | .0 | .0 | 88.8 |
|  | 1028 | 44 | .0 | .0 | 88.8 |
|  | 1029 | 22 | .0 | .0 | 88.8 |
|  | 1030 | 17 | .0 | .0 | 88.9 |
|  | 1031 | 3 | .0 | .0 | 88.9 |
|  | 1032 | 27 | .0 | .0 | 88.9 |
|  | 1033 | 38 | .0 | .0 | 88.9 |
|  | 1034 | 40 | .0 | .0 | 88.9 |
|  | 1035 | 25 | .0 | .0 | 88.9 |
|  | 1036 | 44 | .0 | .0 | 88.9 |
|  | 1037 | 26 | .0 | .0 | 89.0 |
|  | 1038 | 51 | .0 | .0 | 89.0 |
|  | 1039 | 44 | .0 | .0 | 89.0 |
|  | 1040 | 49 | .0 | .0 | 89.0 |
|  | 1041 | 36 | .0 | .0 | 89.1 |
|  | 1042 | 55 | .0 | .0 | 89.1 |
|  | 1043 | 49 | .0 | .0 | 89.1 |
|  | 1044 | 51 | .0 | .0 | 89.1 |
|  | 1045 | 45 | .0 | .0 | 89.2 |
|  | 1046 | 44 | .0 | .0 | 89.2 |
|  | 1047 | 25 | .0 | .0 | 89.2 |
|  | 1048 | 28 | .0 | .0 | 89.2 |
|  | 1049 | 30 | .0 | .0 | 89.2 |
|  | 1050 | 15 | .0 | .0 | 89.2 |
|  | 1051 | 14 | .0 | .0 | 89.2 |
|  | 1052 | 41 | .0 | .0 | 89.3 |
|  | 1053 | 15 | .0 | .0 | 89.3 |
|  | 1054 | 37 | .0 | .0 | 89.3 |
|  | 1055 | 29 | .0 | .0 | 89.3 |
|  | 1056 | 50 | .0 | .0 | 89.3 |
|  | 1057 | 53 | .0 | .0 | 89.4 |
|  | 1058 | 24 | .0 | .0 | 89.4 |
|  | 1059 | 35 | .0 | .0 | 89.4 |
|  | 1060 | 14 | .0 | .0 | 89.4 |
|  | 1061 | 74 | .0 | .0 | 89.4 |
|  | 1062 | 50 | .0 | .0 | 89.5 |
|  | 1063 | 16 | .0 | .0 | 89.5 |
|  | 1064 | 26 | .0 | .0 | 89.5 |
|  | 1065 | 72 | .0 | .0 | 89.5 |
|  | 1066 | 43 | .0 | .0 | 89.5 |
|  | 1067 | 27 | .0 | .0 | 89.6 |
|  | 1068 | 33 | .0 | .0 | 89.6 |
|  | 1069 | 24 | .0 | .0 | 89.6 |
|  | 1070 | 3 | .0 | .0 | 89.6 |
|  | 1071 | 11 | .0 | .0 | 89.6 |
|  | 1073 | 19 | .0 | .0 | 89.6 |
|  | 1074 | 21 | .0 | .0 | 89.6 |
|  | 1075 | 33 | .0 | .0 | 89.6 |
|  | 1076 | 21 | .0 | .0 | 89.6 |
|  | 1077 | 64 | .0 | .0 | 89.7 |
|  | 1078 | 41 | .0 | .0 | 89.7 |
|  | 1079 | 36 | .0 | .0 | 89.7 |
|  | 1080 | 30 | .0 | .0 | 89.7 |
|  | 1081 | 31 | .0 | .0 | 89.8 |
|  | 1082 | 17 | .0 | .0 | 89.8 |
|  | 1083 | 27 | .0 | .0 | 89.8 |
|  | 1084 | 18 | .0 | .0 | 89.8 |
|  | 1085 | 23 | .0 | .0 | 89.8 |
|  | 1086 | 10 | .0 | .0 | 89.8 |
|  | 1087 | 36 | .0 | .0 | 89.8 |
|  | 1088 | 36 | .0 | .0 | 89.8 |
|  | 1089 | 22 | .0 | .0 | 89.9 |
|  | 1090 | 24 | .0 | .0 | 89.9 |
|  | 1091 | 17 | .0 | .0 | 89.9 |
|  | 1092 | 3 | .0 | .0 | 89.9 |
|  | 1093 | 17 | .0 | .0 | 89.9 |
|  | 1094 | 21 | .0 | .0 | 89.9 |
|  | 1095 | 35 | .0 | .0 | 89.9 |
|  | 1096 | 37 | .0 | .0 | 89.9 |
|  | 1097 | 44 | .0 | .0 | 90.0 |
|  | 1098 | 1 | .0 | .0 | 90.0 |
|  | 1099 | 28 | .0 | .0 | 90.0 |
|  | 1100 | 23 | .0 | .0 | 90.0 |
|  | 1101 | 26 | .0 | .0 | 90.0 |
|  | 1102 | 25 | .0 | .0 | 90.0 |
|  | 1103 | 23 | .0 | .0 | 90.0 |
|  | 1104 | 15 | .0 | .0 | 90.0 |
|  | 1105 | 32 | .0 | .0 | 90.0 |
|  | 1106 | 18 | .0 | .0 | 90.1 |
|  | 1107 | 33 | .0 | .0 | 90.1 |
|  | 1108 | 32 | .0 | .0 | 90.1 |
|  | 1109 | 34 | .0 | .0 | 90.1 |
|  | 1110 | 29 | .0 | .0 | 90.1 |
|  | 1111 | 24 | .0 | .0 | 90.1 |
|  | 1112 | 27 | .0 | .0 | 90.1 |
|  | 1113 | 25 | .0 | .0 | 90.2 |
|  | 1114 | 27 | .0 | .0 | 90.2 |
|  | 1115 | 28 | .0 | .0 | 90.2 |
|  | 1116 | 44 | .0 | .0 | 90.2 |
|  | 1117 | 23 | .0 | .0 | 90.2 |
|  | 1118 | 35 | .0 | .0 | 90.2 |
|  | 1119 | 16 | .0 | .0 | 90.3 |
|  | 1120 | 12 | .0 | .0 | 90.3 |
|  | 1121 | 41 | .0 | .0 | 90.3 |
|  | 1122 | 41 | .0 | .0 | 90.3 |
|  | 1123 | 31 | .0 | .0 | 90.3 |
|  | 1124 | 36 | .0 | .0 | 90.3 |
|  | 1125 | 26 | .0 | .0 | 90.4 |
|  | 1126 | 30 | .0 | .0 | 90.4 |
|  | 1127 | 27 | .0 | .0 | 90.4 |
|  | 1128 | 30 | .0 | .0 | 90.4 |
|  | 1129 | 29 | .0 | .0 | 90.4 |
|  | 1130 | 19 | .0 | .0 | 90.4 |
|  | 1131 | 23 | .0 | .0 | 90.4 |
|  | 1132 | 5 | .0 | .0 | 90.4 |
|  | 1133 | 35 | .0 | .0 | 90.5 |
|  | 1134 | 12 | .0 | .0 | 90.5 |
|  | 1135 | 25 | .0 | .0 | 90.5 |
|  | 1136 | 21 | .0 | .0 | 90.5 |
|  | 1137 | 50 | .0 | .0 | 90.5 |
|  | 1138 | 57 | .0 | .0 | 90.5 |
|  | 1139 | 45 | .0 | .0 | 90.6 |
|  | 1140 | 20 | .0 | .0 | 90.6 |
|  | 1141 | 50 | .0 | .0 | 90.6 |
|  | 1142 | 57 | .0 | .0 | 90.6 |
|  | 1143 | 41 | .0 | .0 | 90.7 |
|  | 1144 | 50 | .0 | .0 | 90.7 |
|  | 1145 | 6 | .0 | .0 | 90.7 |
|  | 1146 | 21 | .0 | .0 | 90.7 |
|  | 1147 | 9 | .0 | .0 | 90.7 |
|  | 1148 | 14 | .0 | .0 | 90.7 |
|  | 1150 | 13 | .0 | .0 | 90.7 |
|  | 1151 | 19 | .0 | .0 | 90.7 |
|  | 1152 | 24 | .0 | .0 | 90.7 |
|  | 1153 | 30 | .0 | .0 | 90.8 |
|  | 1154 | 8 | .0 | .0 | 90.8 |
|  | 1155 | 36 | .0 | .0 | 90.8 |
|  | 1156 | 20 | .0 | .0 | 90.8 |
|  | 1157 | 34 | .0 | .0 | 90.8 |
|  | 1158 | 25 | .0 | .0 | 90.8 |
|  | 1159 | 48 | .0 | .0 | 90.8 |
|  | 1160 | 39 | .0 | .0 | 90.9 |
|  | 1161 | 52 | .0 | .0 | 90.9 |
|  | 1162 | 65 | .0 | .0 | 90.9 |
|  | 1163 | 34 | .0 | .0 | 90.9 |
|  | 1164 | 55 | .0 | .0 | 91.0 |
|  | 1165 | 61 | .0 | .0 | 91.0 |
|  | 1166 | 17 | .0 | .0 | 91.0 |
|  | 1167 | 15 | .0 | .0 | 91.0 |
|  | 1168 | 33 | .0 | .0 | 91.0 |
|  | 1169 | 43 | .0 | .0 | 91.1 |
|  | 1170 | 38 | .0 | .0 | 91.1 |
|  | 1171 | 46 | .0 | .0 | 91.1 |
|  | 1172 | 31 | .0 | .0 | 91.1 |
|  | 1173 | 17 | .0 | .0 | 91.1 |
|  | 1174 | 26 | .0 | .0 | 91.1 |
|  | 1175 | 1 | .0 | .0 | 91.1 |
|  | 1176 | 37 | .0 | .0 | 91.2 |
|  | 1177 | 51 | .0 | .0 | 91.2 |
|  | 1178 | 26 | .0 | .0 | 91.2 |
|  | 1179 | 42 | .0 | .0 | 91.2 |
|  | 1180 | 38 | .0 | .0 | 91.2 |
|  | 1181 | 47 | .0 | .0 | 91.3 |
|  | 1182 | 38 | .0 | .0 | 91.3 |
|  | 1183 | 18 | .0 | .0 | 91.3 |
|  | 1184 | 22 | .0 | .0 | 91.3 |
|  | 1185 | 45 | .0 | .0 | 91.3 |
|  | 1186 | 46 | .0 | .0 | 91.4 |
|  | 1187 | 17 | .0 | .0 | 91.4 |
|  | 1188 | 35 | .0 | .0 | 91.4 |
|  | 1189 | 15 | .0 | .0 | 91.4 |
|  | 1190 | 54 | .0 | .0 | 91.4 |
|  | 1191 | 24 | .0 | .0 | 91.4 |
|  | 1192 | 60 | .0 | .0 | 91.5 |
|  | 1193 | 35 | .0 | .0 | 91.5 |
|  | 1194 | 16 | .0 | .0 | 91.5 |
|  | 1195 | 20 | .0 | .0 | 91.5 |
|  | 1196 | 34 | .0 | .0 | 91.5 |
|  | 1197 | 54 | .0 | .0 | 91.6 |
|  | 1198 | 42 | .0 | .0 | 91.6 |
|  | 1199 | 38 | .0 | .0 | 91.6 |
|  | 1200 | 30 | .0 | .0 | 91.6 |
|  | 1201 | 25 | .0 | .0 | 91.6 |
|  | 1202 | 55 | .0 | .0 | 91.7 |
|  | 1203 | 56 | .0 | .0 | 91.7 |
|  | 1204 | 15 | .0 | .0 | 91.7 |
|  | 1205 | 16 | .0 | .0 | 91.7 |
|  | 1206 | 21 | .0 | .0 | 91.7 |
|  | 1207 | 25 | .0 | .0 | 91.7 |
|  | 1208 | 60 | .0 | .0 | 91.8 |
|  | 1209 | 34 | .0 | .0 | 91.8 |
|  | 1210 | 28 | .0 | .0 | 91.8 |
|  | 1211 | 20 | .0 | .0 | 91.8 |
|  | 1212 | 17 | .0 | .0 | 91.8 |
|  | 1213 | 24 | .0 | .0 | 91.8 |
|  | 1214 | 4 | .0 | .0 | 91.8 |
|  | 1215 | 14 | .0 | .0 | 91.8 |
|  | 1216 | 7 | .0 | .0 | 91.8 |
|  | 1217 | 19 | .0 | .0 | 91.8 |
|  | 1218 | 37 | .0 | .0 | 91.9 |
|  | 1219 | 12 | .0 | .0 | 91.9 |
|  | 1220 | 38 | .0 | .0 | 91.9 |
|  | 1221 | 18 | .0 | .0 | 91.9 |
|  | 1222 | 16 | .0 | .0 | 91.9 |
|  | 1223 | 58 | .0 | .0 | 91.9 |
|  | 1224 | 19 | .0 | .0 | 91.9 |
|  | 1225 | 7 | .0 | .0 | 91.9 |
|  | 1226 | 29 | .0 | .0 | 92.0 |
|  | 1227 | 32 | .0 | .0 | 92.0 |
|  | 1228 | 31 | .0 | .0 | 92.0 |
|  | 1229 | 25 | .0 | .0 | 92.0 |
|  | 1230 | 33 | .0 | .0 | 92.0 |
|  | 1231 | 20 | .0 | .0 | 92.0 |
|  | 1232 | 12 | .0 | .0 | 92.0 |
|  | 1233 | 1 | .0 | .0 | 92.0 |
|  | 1234 | 18 | .0 | .0 | 92.1 |
|  | 1235 | 24 | .0 | .0 | 92.1 |
|  | 1236 | 42 | .0 | .0 | 92.1 |
|  | 1237 | 20 | .0 | .0 | 92.1 |
|  | 1238 | 12 | .0 | .0 | 92.1 |
|  | 1239 | 5 | .0 | .0 | 92.1 |
|  | 1240 | 20 | .0 | .0 | 92.1 |
|  | 1241 | 8 | .0 | .0 | 92.1 |
|  | 1242 | 23 | .0 | .0 | 92.1 |
|  | 1243 | 25 | .0 | .0 | 92.1 |
|  | 1244 | 10 | .0 | .0 | 92.2 |
|  | 1245 | 19 | .0 | .0 | 92.2 |
|  | 1246 | 39 | .0 | .0 | 92.2 |
|  | 1247 | 5 | .0 | .0 | 92.2 |
|  | 1248 | 13 | .0 | .0 | 92.2 |
|  | 1249 | 28 | .0 | .0 | 92.2 |
|  | 1250 | 17 | .0 | .0 | 92.2 |
|  | 1251 | 15 | .0 | .0 | 92.2 |
|  | 1252 | 22 | .0 | .0 | 92.2 |
|  | 1253 | 33 | .0 | .0 | 92.3 |
|  | 1254 | 29 | .0 | .0 | 92.3 |
|  | 1255 | 1 | .0 | .0 | 92.3 |
|  | 1256 | 24 | .0 | .0 | 92.3 |
|  | 1257 | 19 | .0 | .0 | 92.3 |
|  | 1258 | 17 | .0 | .0 | 92.3 |
|  | 1259 | 44 | .0 | .0 | 92.3 |
|  | 1260 | 8 | .0 | .0 | 92.3 |
|  | 1261 | 43 | .0 | .0 | 92.3 |
|  | 1262 | 15 | .0 | .0 | 92.4 |
|  | 1263 | 18 | .0 | .0 | 92.4 |
|  | 1264 | 36 | .0 | .0 | 92.4 |
|  | 1265 | 42 | .0 | .0 | 92.4 |
|  | 1266 | 19 | .0 | .0 | 92.4 |
|  | 1267 | 22 | .0 | .0 | 92.4 |
|  | 1268 | 2 | .0 | .0 | 92.4 |
|  | 1269 | 13 | .0 | .0 | 92.4 |
|  | 1270 | 41 | .0 | .0 | 92.5 |
|  | 1271 | 22 | .0 | .0 | 92.5 |
|  | 1272 | 24 | .0 | .0 | 92.5 |
|  | 1273 | 24 | .0 | .0 | 92.5 |
|  | 1274 | 50 | .0 | .0 | 92.5 |
|  | 1275 | 9 | .0 | .0 | 92.5 |
|  | 1276 | 30 | .0 | .0 | 92.5 |
|  | 1277 | 16 | .0 | .0 | 92.6 |
|  | 1278 | 26 | .0 | .0 | 92.6 |
|  | 1279 | 48 | .0 | .0 | 92.6 |
|  | 1280 | 50 | .0 | .0 | 92.6 |
|  | 1281 | 19 | .0 | .0 | 92.6 |
|  | 1282 | 20 | .0 | .0 | 92.6 |
|  | 1283 | 51 | .0 | .0 | 92.7 |
|  | 1284 | 25 | .0 | .0 | 92.7 |
|  | 1285 | 13 | .0 | .0 | 92.7 |
|  | 1286 | 31 | .0 | .0 | 92.7 |
|  | 1287 | 33 | .0 | .0 | 92.7 |
|  | 1288 | 18 | .0 | .0 | 92.7 |
|  | 1289 | 8 | .0 | .0 | 92.7 |
|  | 1291 | 37 | .0 | .0 | 92.8 |
|  | 1292 | 20 | .0 | .0 | 92.8 |
|  | 1293 | 13 | .0 | .0 | 92.8 |
|  | 1294 | 43 | .0 | .0 | 92.8 |
|  | 1295 | 28 | .0 | .0 | 92.8 |
|  | 1296 | 37 | .0 | .0 | 92.8 |
|  | 1297 | 47 | .0 | .0 | 92.8 |
|  | 1298 | 6 | .0 | .0 | 92.9 |
|  | 1299 | 8 | .0 | .0 | 92.9 |
|  | 1300 | 17 | .0 | .0 | 92.9 |
|  | 1301 | 29 | .0 | .0 | 92.9 |
|  | 1302 | 27 | .0 | .0 | 92.9 |
|  | 1303 | 8 | .0 | .0 | 92.9 |
|  | 1304 | 46 | .0 | .0 | 92.9 |
|  | 1305 | 35 | .0 | .0 | 92.9 |
|  | 1306 | 38 | .0 | .0 | 93.0 |
|  | 1307 | 44 | .0 | .0 | 93.0 |
|  | 1308 | 10 | .0 | .0 | 93.0 |
|  | 1309 | 7 | .0 | .0 | 93.0 |
|  | 1310 | 16 | .0 | .0 | 93.0 |
|  | 1311 | 19 | .0 | .0 | 93.0 |
|  | 1312 | 9 | .0 | .0 | 93.0 |
|  | 1313 | 5 | .0 | .0 | 93.0 |
|  | 1314 | 42 | .0 | .0 | 93.0 |
|  | 1315 | 10 | .0 | .0 | 93.0 |
|  | 1316 | 25 | .0 | .0 | 93.1 |
|  | 1317 | 5 | .0 | .0 | 93.1 |
|  | 1318 | 28 | .0 | .0 | 93.1 |
|  | 1319 | 36 | .0 | .0 | 93.1 |
|  | 1320 | 14 | .0 | .0 | 93.1 |
|  | 1321 | 32 | .0 | .0 | 93.1 |
|  | 1322 | 30 | .0 | .0 | 93.1 |
|  | 1323 | 18 | .0 | .0 | 93.1 |
|  | 1324 | 24 | .0 | .0 | 93.2 |
|  | 1325 | 33 | .0 | .0 | 93.2 |
|  | 1326 | 23 | .0 | .0 | 93.2 |
|  | 1327 | 16 | .0 | .0 | 93.2 |
|  | 1328 | 30 | .0 | .0 | 93.2 |
|  | 1329 | 4 | .0 | .0 | 93.2 |
|  | 1330 | 28 | .0 | .0 | 93.2 |
|  | 1331 | 23 | .0 | .0 | 93.2 |
|  | 1332 | 56 | .0 | .0 | 93.3 |
|  | 1333 | 10 | .0 | .0 | 93.3 |
|  | 1334 | 23 | .0 | .0 | 93.3 |
|  | 1335 | 13 | .0 | .0 | 93.3 |
|  | 1336 | 28 | .0 | .0 | 93.3 |
|  | 1337 | 13 | .0 | .0 | 93.3 |
|  | 1338 | 26 | .0 | .0 | 93.3 |
|  | 1339 | 8 | .0 | .0 | 93.3 |
|  | 1340 | 52 | .0 | .0 | 93.4 |
|  | 1341 | 1 | .0 | .0 | 93.4 |
|  | 1342 | 7 | .0 | .0 | 93.4 |
|  | 1343 | 18 | .0 | .0 | 93.4 |
|  | 1345 | 28 | .0 | .0 | 93.4 |
|  | 1346 | 8 | .0 | .0 | 93.4 |
|  | 1347 | 10 | .0 | .0 | 93.4 |
|  | 1348 | 7 | .0 | .0 | 93.4 |
|  | 1350 | 30 | .0 | .0 | 93.4 |
|  | 1351 | 22 | .0 | .0 | 93.4 |
|  | 1352 | 27 | .0 | .0 | 93.4 |
|  | 1353 | 5 | .0 | .0 | 93.4 |
|  | 1354 | 26 | .0 | .0 | 93.5 |
|  | 1355 | 11 | .0 | .0 | 93.5 |
|  | 1356 | 41 | .0 | .0 | 93.5 |
|  | 1358 | 40 | .0 | .0 | 93.5 |
|  | 1359 | 5 | .0 | .0 | 93.5 |
|  | 1360 | 32 | .0 | .0 | 93.5 |
|  | 1361 | 16 | .0 | .0 | 93.5 |
|  | 1362 | 36 | .0 | .0 | 93.6 |
|  | 1363 | 18 | .0 | .0 | 93.6 |
|  | 1364 | 12 | .0 | .0 | 93.6 |
|  | 1365 | 54 | .0 | .0 | 93.6 |
|  | 1366 | 36 | .0 | .0 | 93.6 |
|  | 1367 | 42 | .0 | .0 | 93.6 |
|  | 1368 | 37 | .0 | .0 | 93.7 |
|  | 1369 | 19 | .0 | .0 | 93.7 |
|  | 1370 | 10 | .0 | .0 | 93.7 |
|  | 1371 | 32 | .0 | .0 | 93.7 |
|  | 1372 | 8 | .0 | .0 | 93.7 |
|  | 1373 | 35 | .0 | .0 | 93.7 |
|  | 1374 | 18 | .0 | .0 | 93.7 |
|  | 1375 | 41 | .0 | .0 | 93.7 |
|  | 1376 | 23 | .0 | .0 | 93.8 |
|  | 1377 | 11 | .0 | .0 | 93.8 |
|  | 1378 | 6 | .0 | .0 | 93.8 |
|  | 1379 | 14 | .0 | .0 | 93.8 |
|  | 1380 | 18 | .0 | .0 | 93.8 |
|  | 1381 | 15 | .0 | .0 | 93.8 |
|  | 1382 | 30 | .0 | .0 | 93.8 |
|  | 1383 | 3 | .0 | .0 | 93.8 |
|  | 1384 | 8 | .0 | .0 | 93.8 |
|  | 1385 | 25 | .0 | .0 | 93.8 |
|  | 1386 | 25 | .0 | .0 | 93.8 |
|  | 1387 | 16 | .0 | .0 | 93.8 |
|  | 1388 | 28 | .0 | .0 | 93.9 |
|  | 1389 | 13 | .0 | .0 | 93.9 |
|  | 1390 | 1 | .0 | .0 | 93.9 |
|  | 1391 | 40 | .0 | .0 | 93.9 |
|  | 1392 | 22 | .0 | .0 | 93.9 |
|  | 1393 | 21 | .0 | .0 | 93.9 |
|  | 1394 | 30 | .0 | .0 | 93.9 |
|  | 1395 | 15 | .0 | .0 | 93.9 |
|  | 1396 | 30 | .0 | .0 | 94.0 |
|  | 1397 | 30 | .0 | .0 | 94.0 |
|  | 1398 | 34 | .0 | .0 | 94.0 |
|  | 1399 | 37 | .0 | .0 | 94.0 |
|  | 1400 | 6 | .0 | .0 | 94.0 |
|  | 1401 | 32 | .0 | .0 | 94.0 |
|  | 1402 | 11 | .0 | .0 | 94.0 |
|  | 1403 | 9 | .0 | .0 | 94.0 |
|  | 1404 | 31 | .0 | .0 | 94.1 |
|  | 1405 | 30 | .0 | .0 | 94.1 |
|  | 1406 | 40 | .0 | .0 | 94.1 |
|  | 1407 | 28 | .0 | .0 | 94.1 |
|  | 1408 | 38 | .0 | .0 | 94.1 |
|  | 1409 | 18 | .0 | .0 | 94.1 |
|  | 1410 | 9 | .0 | .0 | 94.1 |
|  | 1411 | 13 | .0 | .0 | 94.1 |
|  | 1412 | 38 | .0 | .0 | 94.2 |
|  | 1413 | 16 | .0 | .0 | 94.2 |
|  | 1414 | 35 | .0 | .0 | 94.2 |
|  | 1415 | 45 | .0 | .0 | 94.2 |
|  | 1416 | 7 | .0 | .0 | 94.2 |
|  | 1417 | 31 | .0 | .0 | 94.2 |
|  | 1418 | 30 | .0 | .0 | 94.3 |
|  | 1419 | 18 | .0 | .0 | 94.3 |
|  | 1420 | 23 | .0 | .0 | 94.3 |
|  | 1421 | 24 | .0 | .0 | 94.3 |
|  | 1422 | 9 | .0 | .0 | 94.3 |
|  | 1423 | 29 | .0 | .0 | 94.3 |
|  | 1424 | 29 | .0 | .0 | 94.3 |
|  | 1425 | 9 | .0 | .0 | 94.3 |
|  | 1426 | 8 | .0 | .0 | 94.3 |
|  | 1427 | 24 | .0 | .0 | 94.3 |
|  | 1428 | 40 | .0 | .0 | 94.4 |
|  | 1429 | 13 | .0 | .0 | 94.4 |
|  | 1430 | 27 | .0 | .0 | 94.4 |
|  | 1431 | 21 | .0 | .0 | 94.4 |
|  | 1432 | 20 | .0 | .0 | 94.4 |
|  | 1433 | 6 | .0 | .0 | 94.4 |
|  | 1434 | 43 | .0 | .0 | 94.4 |
|  | 1435 | 27 | .0 | .0 | 94.4 |
|  | 1436 | 10 | .0 | .0 | 94.5 |
|  | 1437 | 20 | .0 | .0 | 94.5 |
|  | 1438 | 19 | .0 | .0 | 94.5 |
|  | 1439 | 26 | .0 | .0 | 94.5 |
|  | 1440 | 25 | .0 | .0 | 94.5 |
|  | 1441 | 23 | .0 | .0 | 94.5 |
|  | 1442 | 24 | .0 | .0 | 94.5 |
|  | 1443 | 13 | .0 | .0 | 94.5 |
|  | 1444 | 48 | .0 | .0 | 94.6 |
|  | 1445 | 29 | .0 | .0 | 94.6 |
|  | 1446 | 5 | .0 | .0 | 94.6 |
|  | 1447 | 11 | .0 | .0 | 94.6 |
|  | 1448 | 10 | .0 | .0 | 94.6 |
|  | 1449 | 16 | .0 | .0 | 94.6 |
|  | 1450 | 5 | .0 | .0 | 94.6 |
|  | 1451 | 16 | .0 | .0 | 94.6 |
|  | 1452 | 8 | .0 | .0 | 94.6 |
|  | 1453 | 7 | .0 | .0 | 94.6 |
|  | 1454 | 21 | .0 | .0 | 94.6 |
|  | 1455 | 5 | .0 | .0 | 94.6 |
|  | 1456 | 10 | .0 | .0 | 94.6 |
|  | 1457 | 15 | .0 | .0 | 94.6 |
|  | 1458 | 12 | .0 | .0 | 94.6 |
|  | 1459 | 23 | .0 | .0 | 94.7 |
|  | 1460 | 27 | .0 | .0 | 94.7 |
|  | 1461 | 31 | .0 | .0 | 94.7 |
|  | 1462 | 27 | .0 | .0 | 94.7 |
|  | 1463 | 14 | .0 | .0 | 94.7 |
|  | 1464 | 8 | .0 | .0 | 94.7 |
|  | 1465 | 15 | .0 | .0 | 94.7 |
|  | 1466 | 5 | .0 | .0 | 94.7 |
|  | 1467 | 16 | .0 | .0 | 94.7 |
|  | 1468 | 8 | .0 | .0 | 94.7 |
|  | 1469 | 18 | .0 | .0 | 94.7 |
|  | 1471 | 12 | .0 | .0 | 94.8 |
|  | 1472 | 11 | .0 | .0 | 94.8 |
|  | 1473 | 17 | .0 | .0 | 94.8 |
|  | 1475 | 6 | .0 | .0 | 94.8 |
|  | 1476 | 27 | .0 | .0 | 94.8 |
|  | 1477 | 9 | .0 | .0 | 94.8 |
|  | 1478 | 14 | .0 | .0 | 94.8 |
|  | 1479 | 5 | .0 | .0 | 94.8 |
|  | 1480 | 12 | .0 | .0 | 94.8 |
|  | 1481 | 1 | .0 | .0 | 94.8 |
|  | 1482 | 20 | .0 | .0 | 94.8 |
|  | 1483 | 7 | .0 | .0 | 94.8 |
|  | 1484 | 13 | .0 | .0 | 94.8 |
|  | 1485 | 6 | .0 | .0 | 94.8 |
|  | 1486 | 19 | .0 | .0 | 94.8 |
|  | 1487 | 16 | .0 | .0 | 94.8 |
|  | 1488 | 21 | .0 | .0 | 94.9 |
|  | 1489 | 46 | .0 | .0 | 94.9 |
|  | 1490 | 26 | .0 | .0 | 94.9 |
|  | 1491 | 16 | .0 | .0 | 94.9 |
|  | 1492 | 18 | .0 | .0 | 94.9 |
|  | 1493 | 2 | .0 | .0 | 94.9 |
|  | 1494 | 13 | .0 | .0 | 94.9 |
|  | 1495 | 17 | .0 | .0 | 94.9 |
|  | 1496 | 23 | .0 | .0 | 94.9 |
|  | 1497 | 41 | .0 | .0 | 95.0 |
|  | 1498 | 13 | .0 | .0 | 95.0 |
|  | 1499 | 30 | .0 | .0 | 95.0 |
|  | 1500 | 17 | .0 | .0 | 95.0 |
|  | 1501 | 15 | .0 | .0 | 95.0 |
|  | 1502 | 29 | .0 | .0 | 95.0 |
|  | 1503 | 16 | .0 | .0 | 95.0 |
|  | 1504 | 17 | .0 | .0 | 95.0 |
|  | 1505 | 12 | .0 | .0 | 95.0 |
|  | 1506 | 26 | .0 | .0 | 95.1 |
|  | 1507 | 1 | .0 | .0 | 95.1 |
|  | 1508 | 18 | .0 | .0 | 95.1 |
|  | 1509 | 25 | .0 | .0 | 95.1 |
|  | 1513 | 23 | .0 | .0 | 95.1 |
|  | 1514 | 8 | .0 | .0 | 95.1 |
|  | 1515 | 16 | .0 | .0 | 95.1 |
|  | 1516 | 24 | .0 | .0 | 95.1 |
|  | 1517 | 21 | .0 | .0 | 95.1 |
|  | 1518 | 13 | .0 | .0 | 95.1 |
|  | 1519 | 28 | .0 | .0 | 95.1 |
|  | 1520 | 5 | .0 | .0 | 95.2 |
|  | 1521 | 8 | .0 | .0 | 95.2 |
|  | 1522 | 19 | .0 | .0 | 95.2 |
|  | 1523 | 10 | .0 | .0 | 95.2 |
|  | 1524 | 1 | .0 | .0 | 95.2 |
|  | 1525 | 25 | .0 | .0 | 95.2 |
|  | 1526 | 9 | .0 | .0 | 95.2 |
|  | 1527 | 26 | .0 | .0 | 95.2 |
|  | 1528 | 9 | .0 | .0 | 95.2 |
|  | 1529 | 14 | .0 | .0 | 95.2 |
|  | 1530 | 14 | .0 | .0 | 95.2 |
|  | 1531 | 18 | .0 | .0 | 95.2 |
|  | 1532 | 19 | .0 | .0 | 95.2 |
|  | 1533 | 10 | .0 | .0 | 95.2 |
|  | 1534 | 8 | .0 | .0 | 95.3 |
|  | 1536 | 20 | .0 | .0 | 95.3 |
|  | 1538 | 9 | .0 | .0 | 95.3 |
|  | 1539 | 11 | .0 | .0 | 95.3 |
|  | 1541 | 8 | .0 | .0 | 95.3 |
|  | 1542 | 10 | .0 | .0 | 95.3 |
|  | 1543 | 20 | .0 | .0 | 95.3 |
|  | 1544 | 17 | .0 | .0 | 95.3 |
|  | 1545 | 21 | .0 | .0 | 95.3 |
|  | 1546 | 8 | .0 | .0 | 95.3 |
|  | 1547 | 17 | .0 | .0 | 95.3 |
|  | 1548 | 4 | .0 | .0 | 95.3 |
|  | 1549 | 9 | .0 | .0 | 95.3 |
|  | 1550 | 60 | .0 | .0 | 95.4 |
|  | 1551 | 18 | .0 | .0 | 95.4 |
|  | 1552 | 18 | .0 | .0 | 95.4 |
|  | 1553 | 5 | .0 | .0 | 95.4 |
|  | 1554 | 8 | .0 | .0 | 95.4 |
|  | 1555 | 23 | .0 | .0 | 95.4 |
|  | 1556 | 26 | .0 | .0 | 95.4 |
|  | 1557 | 5 | .0 | .0 | 95.4 |
|  | 1558 | 45 | .0 | .0 | 95.4 |
|  | 1559 | 17 | .0 | .0 | 95.5 |
|  | 1560 | 28 | .0 | .0 | 95.5 |
|  | 1561 | 10 | .0 | .0 | 95.5 |
|  | 1562 | 14 | .0 | .0 | 95.5 |
|  | 1563 | 22 | .0 | .0 | 95.5 |
|  | 1564 | 12 | .0 | .0 | 95.5 |
|  | 1565 | 16 | .0 | .0 | 95.5 |
|  | 1566 | 9 | .0 | .0 | 95.5 |
|  | 1567 | 9 | .0 | .0 | 95.5 |
|  | 1569 | 23 | .0 | .0 | 95.5 |
|  | 1570 | 26 | .0 | .0 | 95.5 |
|  | 1572 | 15 | .0 | .0 | 95.5 |
|  | 1573 | 34 | .0 | .0 | 95.6 |
|  | 1574 | 30 | .0 | .0 | 95.6 |
|  | 1575 | 13 | .0 | .0 | 95.6 |
|  | 1576 | 16 | .0 | .0 | 95.6 |
|  | 1577 | 8 | .0 | .0 | 95.6 |
|  | 1578 | 23 | .0 | .0 | 95.6 |
|  | 1580 | 15 | .0 | .0 | 95.6 |
|  | 1581 | 8 | .0 | .0 | 95.6 |
|  | 1582 | 18 | .0 | .0 | 95.6 |
|  | 1583 | 19 | .0 | .0 | 95.6 |
|  | 1584 | 36 | .0 | .0 | 95.7 |
|  | 1585 | 12 | .0 | .0 | 95.7 |
|  | 1586 | 42 | .0 | .0 | 95.7 |
|  | 1587 | 40 | .0 | .0 | 95.7 |
|  | 1588 | 30 | .0 | .0 | 95.7 |
|  | 1589 | 16 | .0 | .0 | 95.7 |
|  | 1590 | 8 | .0 | .0 | 95.7 |
|  | 1591 | 18 | .0 | .0 | 95.8 |
|  | 1592 | 29 | .0 | .0 | 95.8 |
|  | 1593 | 43 | .0 | .0 | 95.8 |
|  | 1594 | 19 | .0 | .0 | 95.8 |
|  | 1595 | 31 | .0 | .0 | 95.8 |
|  | 1596 | 4 | .0 | .0 | 95.8 |
|  | 1597 | 16 | .0 | .0 | 95.8 |
|  | 1598 | 33 | .0 | .0 | 95.8 |
|  | 1599 | 1 | .0 | .0 | 95.8 |
|  | 1600 | 12 | .0 | .0 | 95.8 |
|  | 1601 | 24 | .0 | .0 | 95.9 |
|  | 1602 | 33 | .0 | .0 | 95.9 |
|  | 1603 | 9 | .0 | .0 | 95.9 |
|  | 1605 | 4 | .0 | .0 | 95.9 |
|  | 1606 | 3 | .0 | .0 | 95.9 |
|  | 1607 | 19 | .0 | .0 | 95.9 |
|  | 1608 | 11 | .0 | .0 | 95.9 |
|  | 1609 | 15 | .0 | .0 | 95.9 |
|  | 1610 | 12 | .0 | .0 | 95.9 |
|  | 1611 | 28 | .0 | .0 | 95.9 |
|  | 1612 | 27 | .0 | .0 | 95.9 |
|  | 1613 | 16 | .0 | .0 | 96.0 |
|  | 1614 | 1 | .0 | .0 | 96.0 |
|  | 1615 | 14 | .0 | .0 | 96.0 |
|  | 1616 | 29 | .0 | .0 | 96.0 |
|  | 1618 | 17 | .0 | .0 | 96.0 |
|  | 1619 | 3 | .0 | .0 | 96.0 |
|  | 1620 | 9 | .0 | .0 | 96.0 |
|  | 1621 | 25 | .0 | .0 | 96.0 |
|  | 1622 | 15 | .0 | .0 | 96.0 |
|  | 1624 | 13 | .0 | .0 | 96.0 |
|  | 1625 | 15 | .0 | .0 | 96.0 |
|  | 1626 | 26 | .0 | .0 | 96.0 |
|  | 1627 | 25 | .0 | .0 | 96.1 |
|  | 1629 | 9 | .0 | .0 | 96.1 |
|  | 1630 | 7 | .0 | .0 | 96.1 |
|  | 1631 | 24 | .0 | .0 | 96.1 |
|  | 1632 | 5 | .0 | .0 | 96.1 |
|  | 1633 | 7 | .0 | .0 | 96.1 |
|  | 1634 | 20 | .0 | .0 | 96.1 |
|  | 1635 | 25 | .0 | .0 | 96.1 |
|  | 1636 | 15 | .0 | .0 | 96.1 |
|  | 1637 | 9 | .0 | .0 | 96.1 |
|  | 1638 | 1 | .0 | .0 | 96.1 |
|  | 1639 | 5 | .0 | .0 | 96.1 |
|  | 1640 | 16 | .0 | .0 | 96.1 |
|  | 1642 | 5 | .0 | .0 | 96.1 |
|  | 1643 | 15 | .0 | .0 | 96.1 |
|  | 1645 | 11 | .0 | .0 | 96.1 |
|  | 1646 | 7 | .0 | .0 | 96.2 |
|  | 1647 | 9 | .0 | .0 | 96.2 |
|  | 1649 | 9 | .0 | .0 | 96.2 |
|  | 1650 | 32 | .0 | .0 | 96.2 |
|  | 1651 | 13 | .0 | .0 | 96.2 |
|  | 1652 | 9 | .0 | .0 | 96.2 |
|  | 1653 | 7 | .0 | .0 | 96.2 |
|  | 1655 | 15 | .0 | .0 | 96.2 |
|  | 1656 | 17 | .0 | .0 | 96.2 |
|  | 1657 | 12 | .0 | .0 | 96.2 |
|  | 1659 | 19 | .0 | .0 | 96.2 |
|  | 1660 | 8 | .0 | .0 | 96.2 |
|  | 1663 | 2 | .0 | .0 | 96.2 |
|  | 1664 | 8 | .0 | .0 | 96.2 |
|  | 1666 | 16 | .0 | .0 | 96.2 |
|  | 1667 | 46 | .0 | .0 | 96.3 |
|  | 1668 | 10 | .0 | .0 | 96.3 |
|  | 1669 | 19 | .0 | .0 | 96.3 |
|  | 1670 | 13 | .0 | .0 | 96.3 |
|  | 1671 | 10 | .0 | .0 | 96.3 |
|  | 1673 | 2 | .0 | .0 | 96.3 |
|  | 1674 | 10 | .0 | .0 | 96.3 |
|  | 1675 | 9 | .0 | .0 | 96.3 |
|  | 1676 | 7 | .0 | .0 | 96.3 |
|  | 1677 | 21 | .0 | .0 | 96.3 |
|  | 1679 | 7 | .0 | .0 | 96.3 |
|  | 1680 | 17 | .0 | .0 | 96.3 |
|  | 1681 | 7 | .0 | .0 | 96.3 |
|  | 1682 | 11 | .0 | .0 | 96.3 |
|  | 1683 | 1 | .0 | .0 | 96.3 |
|  | 1685 | 20 | .0 | .0 | 96.4 |
|  | 1686 | 8 | .0 | .0 | 96.4 |
|  | 1687 | 9 | .0 | .0 | 96.4 |
|  | 1688 | 9 | .0 | .0 | 96.4 |
|  | 1689 | 20 | .0 | .0 | 96.4 |
|  | 1690 | 9 | .0 | .0 | 96.4 |
|  | 1691 | 19 | .0 | .0 | 96.4 |
|  | 1692 | 14 | .0 | .0 | 96.4 |
|  | 1693 | 8 | .0 | .0 | 96.4 |
|  | 1694 | 23 | .0 | .0 | 96.4 |
|  | 1695 | 2 | .0 | .0 | 96.4 |
|  | 1696 | 4 | .0 | .0 | 96.4 |
|  | 1697 | 8 | .0 | .0 | 96.4 |
|  | 1698 | 15 | .0 | .0 | 96.4 |
|  | 1699 | 7 | .0 | .0 | 96.4 |
|  | 1700 | 14 | .0 | .0 | 96.4 |
|  | 1701 | 13 | .0 | .0 | 96.4 |
|  | 1703 | 17 | .0 | .0 | 96.5 |
|  | 1705 | 13 | .0 | .0 | 96.5 |
|  | 1707 | 6 | .0 | .0 | 96.5 |
|  | 1708 | 14 | .0 | .0 | 96.5 |
|  | 1709 | 6 | .0 | .0 | 96.5 |
|  | 1710 | 4 | .0 | .0 | 96.5 |
|  | 1711 | 9 | .0 | .0 | 96.5 |
|  | 1715 | 21 | .0 | .0 | 96.5 |
|  | 1717 | 10 | .0 | .0 | 96.5 |
|  | 1718 | 11 | .0 | .0 | 96.5 |
|  | 1720 | 10 | .0 | .0 | 96.5 |
|  | 1721 | 7 | .0 | .0 | 96.5 |
|  | 1723 | 9 | .0 | .0 | 96.5 |
|  | 1724 | 12 | .0 | .0 | 96.5 |
|  | 1727 | 2 | .0 | .0 | 96.5 |
|  | 1728 | 15 | .0 | .0 | 96.5 |
|  | 1729 | 17 | .0 | .0 | 96.5 |
|  | 1730 | 27 | .0 | .0 | 96.6 |
|  | 1731 | 13 | .0 | .0 | 96.6 |
|  | 1732 | 10 | .0 | .0 | 96.6 |
|  | 1733 | 14 | .0 | .0 | 96.6 |
|  | 1736 | 10 | .0 | .0 | 96.6 |
|  | 1737 | 13 | .0 | .0 | 96.6 |
|  | 1738 | 23 | .0 | .0 | 96.6 |
|  | 1739 | 16 | .0 | .0 | 96.6 |
|  | 1742 | 13 | .0 | .0 | 96.6 |
|  | 1743 | 24 | .0 | .0 | 96.6 |
|  | 1744 | 9 | .0 | .0 | 96.6 |
|  | 1745 | 9 | .0 | .0 | 96.6 |
|  | 1747 | 11 | .0 | .0 | 96.6 |
|  | 1748 | 7 | .0 | .0 | 96.7 |
|  | 1749 | 9 | .0 | .0 | 96.7 |
|  | 1750 | 6 | .0 | .0 | 96.7 |
|  | 1751 | 12 | .0 | .0 | 96.7 |
|  | 1752 | 5 | .0 | .0 | 96.7 |
|  | 1753 | 7 | .0 | .0 | 96.7 |
|  | 1754 | 6 | .0 | .0 | 96.7 |
|  | 1755 | 7 | .0 | .0 | 96.7 |
|  | 1756 | 13 | .0 | .0 | 96.7 |
|  | 1757 | 7 | .0 | .0 | 96.7 |
|  | 1758 | 10 | .0 | .0 | 96.7 |
|  | 1759 | 6 | .0 | .0 | 96.7 |
|  | 1762 | 20 | .0 | .0 | 96.7 |
|  | 1763 | 9 | .0 | .0 | 96.7 |
|  | 1764 | 11 | .0 | .0 | 96.7 |
|  | 1766 | 1 | .0 | .0 | 96.7 |
|  | 1767 | 6 | .0 | .0 | 96.7 |
|  | 1768 | 6 | .0 | .0 | 96.7 |
|  | 1769 | 15 | .0 | .0 | 96.7 |
|  | 1770 | 15 | .0 | .0 | 96.7 |
|  | 1771 | 21 | .0 | .0 | 96.8 |
|  | 1772 | 4 | .0 | .0 | 96.8 |
|  | 1773 | 11 | .0 | .0 | 96.8 |
|  | 1775 | 10 | .0 | .0 | 96.8 |
|  | 1776 | 12 | .0 | .0 | 96.8 |
|  | 1777 | 11 | .0 | .0 | 96.8 |
|  | 1778 | 13 | .0 | .0 | 96.8 |
|  | 1779 | 5 | .0 | .0 | 96.8 |
|  | 1780 | 9 | .0 | .0 | 96.8 |
|  | 1781 | 16 | .0 | .0 | 96.8 |
|  | 1782 | 28 | .0 | .0 | 96.8 |
|  | 1783 | 14 | .0 | .0 | 96.8 |
|  | 1785 | 17 | .0 | .0 | 96.8 |
|  | 1786 | 4 | .0 | .0 | 96.8 |
|  | 1787 | 6 | .0 | .0 | 96.8 |
|  | 1788 | 5 | .0 | .0 | 96.8 |
|  | 1790 | 19 | .0 | .0 | 96.8 |
|  | 1791 | 7 | .0 | .0 | 96.9 |
|  | 1792 | 11 | .0 | .0 | 96.9 |
|  | 1793 | 27 | .0 | .0 | 96.9 |
|  | 1794 | 6 | .0 | .0 | 96.9 |
|  | 1796 | 2 | .0 | .0 | 96.9 |
|  | 1797 | 8 | .0 | .0 | 96.9 |
|  | 1798 | 22 | .0 | .0 | 96.9 |
|  | 1800 | 17 | .0 | .0 | 96.9 |
|  | 1802 | 23 | .0 | .0 | 96.9 |
|  | 1803 | 16 | .0 | .0 | 96.9 |
|  | 1804 | 29 | .0 | .0 | 96.9 |
|  | 1805 | 9 | .0 | .0 | 96.9 |
|  | 1806 | 42 | .0 | .0 | 97.0 |
|  | 1808 | 36 | .0 | .0 | 97.0 |
|  | 1810 | 8 | .0 | .0 | 97.0 |
|  | 1811 | 14 | .0 | .0 | 97.0 |
|  | 1812 | 9 | .0 | .0 | 97.0 |
|  | 1813 | 14 | .0 | .0 | 97.0 |
|  | 1814 | 4 | .0 | .0 | 97.0 |
|  | 1815 | 9 | .0 | .0 | 97.0 |
|  | 1816 | 17 | .0 | .0 | 97.0 |
|  | 1818 | 5 | .0 | .0 | 97.0 |
|  | 1821 | 7 | .0 | .0 | 97.0 |
|  | 1822 | 15 | .0 | .0 | 97.0 |
|  | 1823 | 6 | .0 | .0 | 97.0 |
|  | 1824 | 9 | .0 | .0 | 97.0 |
|  | 1825 | 9 | .0 | .0 | 97.0 |
|  | 1827 | 9 | .0 | .0 | 97.1 |
|  | 1828 | 15 | .0 | .0 | 97.1 |
|  | 1829 | 14 | .0 | .0 | 97.1 |
|  | 1830 | 8 | .0 | .0 | 97.1 |
|  | 1831 | 16 | .0 | .0 | 97.1 |
|  | 1832 | 10 | .0 | .0 | 97.1 |
|  | 1833 | 4 | .0 | .0 | 97.1 |
|  | 1834 | 2 | .0 | .0 | 97.1 |
|  | 1835 | 8 | .0 | .0 | 97.1 |
|  | 1839 | 7 | .0 | .0 | 97.1 |
|  | 1841 | 5 | .0 | .0 | 97.1 |
|  | 1842 | 18 | .0 | .0 | 97.1 |
|  | 1844 | 14 | .0 | .0 | 97.1 |
|  | 1845 | 5 | .0 | .0 | 97.1 |
|  | 1846 | 6 | .0 | .0 | 97.1 |
|  | 1847 | 6 | .0 | .0 | 97.1 |
|  | 1848 | 6 | .0 | .0 | 97.1 |
|  | 1849 | 9 | .0 | .0 | 97.1 |
|  | 1850 | 5 | .0 | .0 | 97.1 |
|  | 1851 | 10 | .0 | .0 | 97.1 |
|  | 1853 | 15 | .0 | .0 | 97.1 |
|  | 1854 | 1 | .0 | .0 | 97.1 |
|  | 1856 | 15 | .0 | .0 | 97.2 |
|  | 1858 | 23 | .0 | .0 | 97.2 |
|  | 1859 | 23 | .0 | .0 | 97.2 |
|  | 1861 | 8 | .0 | .0 | 97.2 |
|  | 1863 | 3 | .0 | .0 | 97.2 |
|  | 1864 | 13 | .0 | .0 | 97.2 |
|  | 1867 | 9 | .0 | .0 | 97.2 |
|  | 1868 | 8 | .0 | .0 | 97.2 |
|  | 1870 | 20 | .0 | .0 | 97.2 |
|  | 1872 | 5 | .0 | .0 | 97.2 |
|  | 1873 | 4 | .0 | .0 | 97.2 |
|  | 1875 | 2 | .0 | .0 | 97.2 |
|  | 1876 | 8 | .0 | .0 | 97.2 |
|  | 1878 | 2 | .0 | .0 | 97.2 |
|  | 1882 | 7 | .0 | .0 | 97.2 |
|  | 1883 | 4 | .0 | .0 | 97.2 |
|  | 1884 | 8 | .0 | .0 | 97.2 |
|  | 1885 | 4 | .0 | .0 | 97.2 |
|  | 1886 | 1 | .0 | .0 | 97.2 |
|  | 1890 | 26 | .0 | .0 | 97.3 |
|  | 1891 | 10 | .0 | .0 | 97.3 |
|  | 1892 | 11 | .0 | .0 | 97.3 |
|  | 1893 | 1 | .0 | .0 | 97.3 |
|  | 1894 | 6 | .0 | .0 | 97.3 |
|  | 1896 | 15 | .0 | .0 | 97.3 |
|  | 1899 | 15 | .0 | .0 | 97.3 |
|  | 1900 | 8 | .0 | .0 | 97.3 |
|  | 1901 | 5 | .0 | .0 | 97.3 |
|  | 1904 | 6 | .0 | .0 | 97.3 |
|  | 1905 | 22 | .0 | .0 | 97.3 |
|  | 1908 | 20 | .0 | .0 | 97.3 |
|  | 1909 | 12 | .0 | .0 | 97.3 |
|  | 1910 | 7 | .0 | .0 | 97.3 |
|  | 1911 | 8 | .0 | .0 | 97.3 |
|  | 1912 | 6 | .0 | .0 | 97.3 |
|  | 1913 | 6 | .0 | .0 | 97.3 |
|  | 1914 | 5 | .0 | .0 | 97.3 |
|  | 1915 | 5 | .0 | .0 | 97.3 |
|  | 1918 | 6 | .0 | .0 | 97.3 |
|  | 1919 | 12 | .0 | .0 | 97.3 |
|  | 1921 | 7 | .0 | .0 | 97.4 |
|  | 1922 | 2 | .0 | .0 | 97.4 |
|  | 1923 | 3 | .0 | .0 | 97.4 |
|  | 1927 | 7 | .0 | .0 | 97.4 |
|  | 1930 | 5 | .0 | .0 | 97.4 |
|  | 1932 | 10 | .0 | .0 | 97.4 |
|  | 1933 | 5 | .0 | .0 | 97.4 |
|  | 1935 | 4 | .0 | .0 | 97.4 |
|  | 1936 | 7 | .0 | .0 | 97.4 |
|  | 1937 | 5 | .0 | .0 | 97.4 |
|  | 1938 | 6 | .0 | .0 | 97.4 |
|  | 1940 | 5 | .0 | .0 | 97.4 |
|  | 1941 | 21 | .0 | .0 | 97.4 |
|  | 1942 | 1 | .0 | .0 | 97.4 |
|  | 1943 | 14 | .0 | .0 | 97.4 |
|  | 1944 | 5 | .0 | .0 | 97.4 |
|  | 1945 | 27 | .0 | .0 | 97.4 |
|  | 1946 | 6 | .0 | .0 | 97.4 |
|  | 1950 | 5 | .0 | .0 | 97.4 |
|  | 1951 | 11 | .0 | .0 | 97.4 |
|  | 1954 | 6 | .0 | .0 | 97.4 |
|  | 1955 | 5 | .0 | .0 | 97.4 |
|  | 1956 | 7 | .0 | .0 | 97.4 |
|  | 1958 | 13 | .0 | .0 | 97.4 |
|  | 1960 | 12 | .0 | .0 | 97.5 |
|  | 1962 | 7 | .0 | .0 | 97.5 |
|  | 1965 | 5 | .0 | .0 | 97.5 |
|  | 1966 | 12 | .0 | .0 | 97.5 |
|  | 1968 | 6 | .0 | .0 | 97.5 |
|  | 1974 | 13 | .0 | .0 | 97.5 |
|  | 1975 | 7 | .0 | .0 | 97.5 |
|  | 1976 | 12 | .0 | .0 | 97.5 |
|  | 1978 | 5 | .0 | .0 | 97.5 |
|  | 1979 | 4 | .0 | .0 | 97.5 |
|  | 1980 | 2 | .0 | .0 | 97.5 |
|  | 1981 | 8 | .0 | .0 | 97.5 |
|  | 1982 | 6 | .0 | .0 | 97.5 |
|  | 1983 | 5 | .0 | .0 | 97.5 |
|  | 1984 | 7 | .0 | .0 | 97.5 |
|  | 1985 | 2 | .0 | .0 | 97.5 |
|  | 1986 | 10 | .0 | .0 | 97.5 |
|  | 1987 | 4 | .0 | .0 | 97.5 |
|  | 1988 | 10 | .0 | .0 | 97.5 |
|  | 1989 | 5 | .0 | .0 | 97.5 |
|  | 1990 | 4 | .0 | .0 | 97.5 |
|  | 1991 | 4 | .0 | .0 | 97.5 |
|  | 1992 | 14 | .0 | .0 | 97.5 |
|  | 1993 | 4 | .0 | .0 | 97.5 |
|  | 1996 | 7 | .0 | .0 | 97.5 |
|  | 1998 | 11 | .0 | .0 | 97.5 |
|  | 2000 | 5 | .0 | .0 | 97.5 |
|  | 2002 | 5 | .0 | .0 | 97.6 |
|  | 2003 | 6 | .0 | .0 | 97.6 |
|  | 2005 | 7 | .0 | .0 | 97.6 |
|  | 2008 | 5 | .0 | .0 | 97.6 |
|  | 2009 | 7 | .0 | .0 | 97.6 |
|  | 2013 | 14 | .0 | .0 | 97.6 |
|  | 2015 | 13 | .0 | .0 | 97.6 |
|  | 2019 | 9 | .0 | .0 | 97.6 |
|  | 2022 | 12 | .0 | .0 | 97.6 |
|  | 2023 | 8 | .0 | .0 | 97.6 |
|  | 2024 | 9 | .0 | .0 | 97.6 |
|  | 2027 | 8 | .0 | .0 | 97.6 |
|  | 2028 | 11 | .0 | .0 | 97.6 |
|  | 2029 | 7 | .0 | .0 | 97.6 |
|  | 2030 | 12 | .0 | .0 | 97.6 |
|  | 2033 | 15 | .0 | .0 | 97.6 |
|  | 2034 | 1 | .0 | .0 | 97.6 |
|  | 2036 | 16 | .0 | .0 | 97.6 |
|  | 2038 | 8 | .0 | .0 | 97.6 |
|  | 2046 | 5 | .0 | .0 | 97.6 |
|  | 2048 | 16 | .0 | .0 | 97.6 |
|  | 2049 | 4 | .0 | .0 | 97.7 |
|  | 2050 | 1 | .0 | .0 | 97.7 |
|  | 2051 | 3 | .0 | .0 | 97.7 |
|  | 2053 | 5 | .0 | .0 | 97.7 |
|  | 2054 | 9 | .0 | .0 | 97.7 |
|  | 2055 | 7 | .0 | .0 | 97.7 |
|  | 2057 | 8 | .0 | .0 | 97.7 |
|  | 2058 | 7 | .0 | .0 | 97.7 |
|  | 2060 | 6 | .0 | .0 | 97.7 |
|  | 2061 | 9 | .0 | .0 | 97.7 |
|  | 2064 | 10 | .0 | .0 | 97.7 |
|  | 2065 | 15 | .0 | .0 | 97.7 |
|  | 2066 | 14 | .0 | .0 | 97.7 |
|  | 2067 | 8 | .0 | .0 | 97.7 |
|  | 2069 | 13 | .0 | .0 | 97.7 |
|  | 2071 | 8 | .0 | .0 | 97.7 |
|  | 2072 | 6 | .0 | .0 | 97.7 |
|  | 2074 | 19 | .0 | .0 | 97.7 |
|  | 2076 | 15 | .0 | .0 | 97.7 |
|  | 2078 | 3 | .0 | .0 | 97.7 |
|  | 2080 | 1 | .0 | .0 | 97.7 |
|  | 2082 | 8 | .0 | .0 | 97.7 |
|  | 2083 | 6 | .0 | .0 | 97.7 |
|  | 2084 | 5 | .0 | .0 | 97.7 |
|  | 2085 | 9 | .0 | .0 | 97.8 |
|  | 2086 | 5 | .0 | .0 | 97.8 |
|  | 2087 | 7 | .0 | .0 | 97.8 |
|  | 2088 | 16 | .0 | .0 | 97.8 |
|  | 2092 | 7 | .0 | .0 | 97.8 |
|  | 2096 | 10 | .0 | .0 | 97.8 |
|  | 2099 | 5 | .0 | .0 | 97.8 |
|  | 2100 | 7 | .0 | .0 | 97.8 |
|  | 2101 | 7 | .0 | .0 | 97.8 |
|  | 2102 | 9 | .0 | .0 | 97.8 |
|  | 2103 | 7 | .0 | .0 | 97.8 |
|  | 2106 | 1 | .0 | .0 | 97.8 |
|  | 2110 | 1 | .0 | .0 | 97.8 |
|  | 2111 | 11 | .0 | .0 | 97.8 |
|  | 2112 | 10 | .0 | .0 | 97.8 |
|  | 2114 | 5 | .0 | .0 | 97.8 |
|  | 2115 | 10 | .0 | .0 | 97.8 |
|  | 2120 | 5 | .0 | .0 | 97.8 |
|  | 2121 | 7 | .0 | .0 | 97.8 |
|  | 2122 | 10 | .0 | .0 | 97.8 |
|  | 2123 | 21 | .0 | .0 | 97.8 |
|  | 2124 | 10 | .0 | .0 | 97.8 |
|  | 2125 | 7 | .0 | .0 | 97.8 |
|  | 2126 | 5 | .0 | .0 | 97.9 |
|  | 2129 | 7 | .0 | .0 | 97.9 |
|  | 2130 | 6 | .0 | .0 | 97.9 |
|  | 2131 | 8 | .0 | .0 | 97.9 |
|  | 2135 | 6 | .0 | .0 | 97.9 |
|  | 2136 | 8 | .0 | .0 | 97.9 |
|  | 2138 | 20 | .0 | .0 | 97.9 |
|  | 2139 | 12 | .0 | .0 | 97.9 |
|  | 2143 | 4 | .0 | .0 | 97.9 |
|  | 2145 | 7 | .0 | .0 | 97.9 |
|  | 2146 | 10 | .0 | .0 | 97.9 |
|  | 2148 | 7 | .0 | .0 | 97.9 |
|  | 2151 | 11 | .0 | .0 | 97.9 |
|  | 2152 | 7 | .0 | .0 | 97.9 |
|  | 2153 | 7 | .0 | .0 | 97.9 |
|  | 2154 | 7 | .0 | .0 | 97.9 |
|  | 2155 | 7 | .0 | .0 | 97.9 |
|  | 2160 | 15 | .0 | .0 | 97.9 |
|  | 2161 | 8 | .0 | .0 | 97.9 |
|  | 2165 | 15 | .0 | .0 | 97.9 |
|  | 2169 | 8 | .0 | .0 | 97.9 |
|  | 2172 | 1 | .0 | .0 | 97.9 |
|  | 2175 | 12 | .0 | .0 | 98.0 |
|  | 2176 | 13 | .0 | .0 | 98.0 |
|  | 2177 | 15 | .0 | .0 | 98.0 |
|  | 2178 | 7 | .0 | .0 | 98.0 |
|  | 2179 | 8 | .0 | .0 | 98.0 |
|  | 2180 | 8 | .0 | .0 | 98.0 |
|  | 2185 | 14 | .0 | .0 | 98.0 |
|  | 2187 | 12 | .0 | .0 | 98.0 |
|  | 2193 | 8 | .0 | .0 | 98.0 |
|  | 2194 | 4 | .0 | .0 | 98.0 |
|  | 2196 | 7 | .0 | .0 | 98.0 |
|  | 2198 | 8 | .0 | .0 | 98.0 |
|  | 2199 | 8 | .0 | .0 | 98.0 |
|  | 2201 | 12 | .0 | .0 | 98.0 |
|  | 2204 | 7 | .0 | .0 | 98.0 |
|  | 2208 | 7 | .0 | .0 | 98.0 |
|  | 2209 | 10 | .0 | .0 | 98.0 |
|  | 2210 | 5 | .0 | .0 | 98.0 |
|  | 2211 | 10 | .0 | .0 | 98.0 |
|  | 2212 | 11 | .0 | .0 | 98.0 |
|  | 2213 | 9 | .0 | .0 | 98.0 |
|  | 2214 | 2 | .0 | .0 | 98.0 |
|  | 2216 | 6 | .0 | .0 | 98.1 |
|  | 2217 | 15 | .0 | .0 | 98.1 |
|  | 2218 | 18 | .0 | .0 | 98.1 |
|  | 2219 | 8 | .0 | .0 | 98.1 |
|  | 2221 | 20 | .0 | .0 | 98.1 |
|  | 2222 | 10 | .0 | .0 | 98.1 |
|  | 2223 | 5 | .0 | .0 | 98.1 |
|  | 2224 | 10 | .0 | .0 | 98.1 |
|  | 2225 | 8 | .0 | .0 | 98.1 |
|  | 2227 | 11 | .0 | .0 | 98.1 |
|  | 2229 | 5 | .0 | .0 | 98.1 |
|  | 2235 | 7 | .0 | .0 | 98.1 |
|  | 2237 | 7 | .0 | .0 | 98.1 |
|  | 2239 | 26 | .0 | .0 | 98.1 |
|  | 2240 | 5 | .0 | .0 | 98.1 |
|  | 2241 | 4 | .0 | .0 | 98.1 |
|  | 2242 | 7 | .0 | .0 | 98.1 |
|  | 2244 | 6 | .0 | .0 | 98.1 |
|  | 2246 | 11 | .0 | .0 | 98.1 |
|  | 2247 | 5 | .0 | .0 | 98.2 |
|  | 2249 | 11 | .0 | .0 | 98.2 |
|  | 2252 | 13 | .0 | .0 | 98.2 |
|  | 2253 | 12 | .0 | .0 | 98.2 |
|  | 2257 | 13 | .0 | .0 | 98.2 |
|  | 2258 | 6 | .0 | .0 | 98.2 |
|  | 2262 | 10 | .0 | .0 | 98.2 |
|  | 2263 | 4 | .0 | .0 | 98.2 |
|  | 2265 | 9 | .0 | .0 | 98.2 |
|  | 2266 | 8 | .0 | .0 | 98.2 |
|  | 2267 | 9 | .0 | .0 | 98.2 |
|  | 2272 | 6 | .0 | .0 | 98.2 |
|  | 2275 | 11 | .0 | .0 | 98.2 |
|  | 2277 | 2 | .0 | .0 | 98.2 |
|  | 2279 | 7 | .0 | .0 | 98.2 |
|  | 2280 | 13 | .0 | .0 | 98.2 |
|  | 2282 | 6 | .0 | .0 | 98.2 |
|  | 2283 | 10 | .0 | .0 | 98.2 |
|  | 2284 | 9 | .0 | .0 | 98.2 |
|  | 2288 | 14 | .0 | .0 | 98.2 |
|  | 2289 | 10 | .0 | .0 | 98.2 |
|  | 2291 | 6 | .0 | .0 | 98.3 |
|  | 2292 | 10 | .0 | .0 | 98.3 |
|  | 2293 | 10 | .0 | .0 | 98.3 |
|  | 2294 | 9 | .0 | .0 | 98.3 |
|  | 2299 | 9 | .0 | .0 | 98.3 |
|  | 2300 | 8 | .0 | .0 | 98.3 |
|  | 2302 | 11 | .0 | .0 | 98.3 |
|  | 2303 | 10 | .0 | .0 | 98.3 |
|  | 2304 | 3 | .0 | .0 | 98.3 |
|  | 2308 | 3 | .0 | .0 | 98.3 |
|  | 2311 | 11 | .0 | .0 | 98.3 |
|  | 2313 | 11 | .0 | .0 | 98.3 |
|  | 2316 | 7 | .0 | .0 | 98.3 |
|  | 2318 | 8 | .0 | .0 | 98.3 |
|  | 2319 | 4 | .0 | .0 | 98.3 |
|  | 2320 | 7 | .0 | .0 | 98.3 |
|  | 2321 | 16 | .0 | .0 | 98.3 |
|  | 2322 | 3 | .0 | .0 | 98.3 |
|  | 2325 | 7 | .0 | .0 | 98.3 |
|  | 2326 | 7 | .0 | .0 | 98.3 |
|  | 2327 | 15 | .0 | .0 | 98.3 |
|  | 2329 | 3 | .0 | .0 | 98.3 |
|  | 2330 | 3 | .0 | .0 | 98.3 |
|  | 2331 | 4 | .0 | .0 | 98.3 |
|  | 2335 | 6 | .0 | .0 | 98.3 |
|  | 2336 | 7 | .0 | .0 | 98.4 |
|  | 2337 | 5 | .0 | .0 | 98.4 |
|  | 2341 | 5 | .0 | .0 | 98.4 |
|  | 2343 | 1 | .0 | .0 | 98.4 |
|  | 2344 | 7 | .0 | .0 | 98.4 |
|  | 2345 | 3 | .0 | .0 | 98.4 |
|  | 2346 | 8 | .0 | .0 | 98.4 |
|  | 2347 | 4 | .0 | .0 | 98.4 |
|  | 2348 | 7 | .0 | .0 | 98.4 |
|  | 2349 | 6 | .0 | .0 | 98.4 |
|  | 2350 | 14 | .0 | .0 | 98.4 |
|  | 2351 | 5 | .0 | .0 | 98.4 |
|  | 2352 | 8 | .0 | .0 | 98.4 |
|  | 2354 | 7 | .0 | .0 | 98.4 |
|  | 2355 | 8 | .0 | .0 | 98.4 |
|  | 2356 | 17 | .0 | .0 | 98.4 |
|  | 2357 | 5 | .0 | .0 | 98.4 |
|  | 2362 | 15 | .0 | .0 | 98.4 |
|  | 2363 | 4 | .0 | .0 | 98.4 |
|  | 2364 | 18 | .0 | .0 | 98.4 |
|  | 2366 | 7 | .0 | .0 | 98.4 |
|  | 2368 | 6 | .0 | .0 | 98.4 |
|  | 2369 | 1 | .0 | .0 | 98.4 |
|  | 2372 | 9 | .0 | .0 | 98.4 |
|  | 2374 | 18 | .0 | .0 | 98.4 |
|  | 2381 | 8 | .0 | .0 | 98.5 |
|  | 2383 | 4 | .0 | .0 | 98.5 |
|  | 2384 | 8 | .0 | .0 | 98.5 |
|  | 2385 | 9 | .0 | .0 | 98.5 |
|  | 2386 | 4 | .0 | .0 | 98.5 |
|  | 2388 | 14 | .0 | .0 | 98.5 |
|  | 2395 | 13 | .0 | .0 | 98.5 |
|  | 2396 | 5 | .0 | .0 | 98.5 |
|  | 2397 | 5 | .0 | .0 | 98.5 |
|  | 2400 | 7 | .0 | .0 | 98.5 |
|  | 2402 | 9 | .0 | .0 | 98.5 |
|  | 2403 | 9 | .0 | .0 | 98.5 |
|  | 2404 | 3 | .0 | .0 | 98.5 |
|  | 2408 | 7 | .0 | .0 | 98.5 |
|  | 2412 | 6 | .0 | .0 | 98.5 |
|  | 2414 | 2 | .0 | .0 | 98.5 |
|  | 2416 | 1 | .0 | .0 | 98.5 |
|  | 2417 | 9 | .0 | .0 | 98.5 |
|  | 2418 | 9 | .0 | .0 | 98.5 |
|  | 2419 | 12 | .0 | .0 | 98.5 |
|  | 2420 | 8 | .0 | .0 | 98.5 |
|  | 2421 | 3 | .0 | .0 | 98.5 |
|  | 2424 | 10 | .0 | .0 | 98.5 |
|  | 2426 | 1 | .0 | .0 | 98.5 |
|  | 2427 | 10 | .0 | .0 | 98.5 |
|  | 2428 | 9 | .0 | .0 | 98.5 |
|  | 2429 | 9 | .0 | .0 | 98.6 |
|  | 2430 | 15 | .0 | .0 | 98.6 |
|  | 2431 | 6 | .0 | .0 | 98.6 |
|  | 2432 | 12 | .0 | .0 | 98.6 |
|  | 2434 | 10 | .0 | .0 | 98.6 |
|  | 2437 | 19 | .0 | .0 | 98.6 |
|  | 2440 | 1 | .0 | .0 | 98.6 |
|  | 2441 | 9 | .0 | .0 | 98.6 |
|  | 2443 | 10 | .0 | .0 | 98.6 |
|  | 2445 | 9 | .0 | .0 | 98.6 |
|  | 2446 | 5 | .0 | .0 | 98.6 |
|  | 2450 | 11 | .0 | .0 | 98.6 |
|  | 2452 | 21 | .0 | .0 | 98.6 |
|  | 2454 | 10 | .0 | .0 | 98.6 |
|  | 2455 | 6 | .0 | .0 | 98.6 |
|  | 2456 | 12 | .0 | .0 | 98.6 |
|  | 2459 | 7 | .0 | .0 | 98.6 |
|  | 2461 | 3 | .0 | .0 | 98.6 |
|  | 2462 | 10 | .0 | .0 | 98.6 |
|  | 2463 | 3 | .0 | .0 | 98.6 |
|  | 2464 | 10 | .0 | .0 | 98.7 |
|  | 2465 | 10 | .0 | .0 | 98.7 |
|  | 2466 | 7 | .0 | .0 | 98.7 |
|  | 2469 | 4 | .0 | .0 | 98.7 |
|  | 2470 | 8 | .0 | .0 | 98.7 |
|  | 2471 | 17 | .0 | .0 | 98.7 |
|  | 2472 | 10 | .0 | .0 | 98.7 |
|  | 2473 | 2 | .0 | .0 | 98.7 |
|  | 2474 | 4 | .0 | .0 | 98.7 |
|  | 2475 | 10 | .0 | .0 | 98.7 |
|  | 2477 | 10 | .0 | .0 | 98.7 |
|  | 2479 | 4 | .0 | .0 | 98.7 |
|  | 2481 | 8 | .0 | .0 | 98.7 |
|  | 2482 | 15 | .0 | .0 | 98.7 |
|  | 2483 | 7 | .0 | .0 | 98.7 |
|  | 2485 | 10 | .0 | .0 | 98.7 |
|  | 2487 | 3 | .0 | .0 | 98.7 |
|  | 2488 | 7 | .0 | .0 | 98.7 |
|  | 2491 | 8 | .0 | .0 | 98.7 |
|  | 2495 | 8 | .0 | .0 | 98.7 |
|  | 2497 | 10 | .0 | .0 | 98.7 |
|  | 2499 | 9 | .0 | .0 | 98.7 |
|  | 2500 | 1 | .0 | .0 | 98.7 |
|  | 2507 | 18 | .0 | .0 | 98.8 |
|  | 2509 | 1 | .0 | .0 | 98.8 |
|  | 2512 | 12 | .0 | .0 | 98.8 |
|  | 2515 | 17 | .0 | .0 | 98.8 |
|  | 2516 | 16 | .0 | .0 | 98.8 |
|  | 2519 | 15 | .0 | .0 | 98.8 |
|  | 2522 | 13 | .0 | .0 | 98.8 |
|  | 2524 | 1 | .0 | .0 | 98.8 |
|  | 2525 | 5 | .0 | .0 | 98.8 |
|  | 2526 | 10 | .0 | .0 | 98.8 |
|  | 2527 | 16 | .0 | .0 | 98.8 |
|  | 2528 | 7 | .0 | .0 | 98.8 |
|  | 2529 | 7 | .0 | .0 | 98.8 |
|  | 2530 | 7 | .0 | .0 | 98.8 |
|  | 2531 | 10 | .0 | .0 | 98.8 |
|  | 2532 | 17 | .0 | .0 | 98.8 |
|  | 2535 | 5 | .0 | .0 | 98.8 |
|  | 2536 | 17 | .0 | .0 | 98.8 |
|  | 2538 | 7 | .0 | .0 | 98.8 |
|  | 2539 | 4 | .0 | .0 | 98.8 |
|  | 2540 | 1 | .0 | .0 | 98.8 |
|  | 2543 | 3 | .0 | .0 | 98.9 |
|  | 2544 | 8 | .0 | .0 | 98.9 |
|  | 2545 | 7 | .0 | .0 | 98.9 |
|  | 2546 | 8 | .0 | .0 | 98.9 |
|  | 2547 | 10 | .0 | .0 | 98.9 |
|  | 2548 | 13 | .0 | .0 | 98.9 |
|  | 2553 | 10 | .0 | .0 | 98.9 |
|  | 2554 | 7 | .0 | .0 | 98.9 |
|  | 2555 | 17 | .0 | .0 | 98.9 |
|  | 2556 | 9 | .0 | .0 | 98.9 |
|  | 2557 | 7 | .0 | .0 | 98.9 |
|  | 2558 | 10 | .0 | .0 | 98.9 |
|  | 2559 | 6 | .0 | .0 | 98.9 |
|  | 2560 | 9 | .0 | .0 | 98.9 |
|  | 2565 | 10 | .0 | .0 | 98.9 |
|  | 2566 | 8 | .0 | .0 | 98.9 |
|  | 2567 | 10 | .0 | .0 | 98.9 |
|  | 2568 | 16 | .0 | .0 | 98.9 |
|  | 2569 | 6 | .0 | .0 | 98.9 |
|  | 2571 | 6 | .0 | .0 | 98.9 |
|  | 2572 | 5 | .0 | .0 | 98.9 |
|  | 2573 | 1 | .0 | .0 | 98.9 |
|  | 2575 | 7 | .0 | .0 | 99.0 |
|  | 2576 | 7 | .0 | .0 | 99.0 |
|  | 2577 | 16 | .0 | .0 | 99.0 |
|  | 2578 | 3 | .0 | .0 | 99.0 |
|  | 2579 | 5 | .0 | .0 | 99.0 |
|  | 2580 | 7 | .0 | .0 | 99.0 |
|  | 2581 | 7 | .0 | .0 | 99.0 |
|  | 2583 | 9 | .0 | .0 | 99.0 |
|  | 2584 | 10 | .0 | .0 | 99.0 |
|  | 2587 | 1 | .0 | .0 | 99.0 |
|  | 2588 | 2 | .0 | .0 | 99.0 |
|  | 2591 | 4 | .0 | .0 | 99.0 |
|  | 2594 | 10 | .0 | .0 | 99.0 |
|  | 2604 | 12 | .0 | .0 | 99.0 |
|  | 2607 | 8 | .0 | .0 | 99.0 |
|  | 2608 | 6 | .0 | .0 | 99.0 |
|  | 2611 | 12 | .0 | .0 | 99.0 |
|  | 2623 | 7 | .0 | .0 | 99.0 |
|  | 2627 | 9 | .0 | .0 | 99.0 |
|  | 2631 | 7 | .0 | .0 | 99.0 |
|  | 2632 | 9 | .0 | .0 | 99.0 |
|  | 2636 | 1 | .0 | .0 | 99.0 |
|  | 2639 | 8 | .0 | .0 | 99.0 |
|  | 2641 | 9 | .0 | .0 | 99.0 |
|  | 2643 | 5 | .0 | .0 | 99.0 |
|  | 2644 | 1 | .0 | .0 | 99.0 |
|  | 2648 | 1 | .0 | .0 | 99.0 |
|  | 2649 | 10 | .0 | .0 | 99.0 |
|  | 2652 | 9 | .0 | .0 | 99.1 |
|  | 2658 | 4 | .0 | .0 | 99.1 |
|  | 2659 | 16 | .0 | .0 | 99.1 |
|  | 2660 | 9 | .0 | .0 | 99.1 |
|  | 2661 | 5 | .0 | .0 | 99.1 |
|  | 2666 | 9 | .0 | .0 | 99.1 |
|  | 2667 | 9 | .0 | .0 | 99.1 |
|  | 2669 | 9 | .0 | .0 | 99.1 |
|  | 2671 | 4 | .0 | .0 | 99.1 |
|  | 2673 | 1 | .0 | .0 | 99.1 |
|  | 2674 | 7 | .0 | .0 | 99.1 |
|  | 2676 | 9 | .0 | .0 | 99.1 |
|  | 2677 | 7 | .0 | .0 | 99.1 |
|  | 2678 | 8 | .0 | .0 | 99.1 |
|  | 2681 | 17 | .0 | .0 | 99.1 |
|  | 2682 | 7 | .0 | .0 | 99.1 |
|  | 2685 | 9 | .0 | .0 | 99.1 |
|  | 2690 | 6 | .0 | .0 | 99.1 |
|  | 2691 | 9 | .0 | .0 | 99.1 |
|  | 2692 | 10 | .0 | .0 | 99.1 |
|  | 2695 | 2 | .0 | .0 | 99.1 |
|  | 2697 | 1 | .0 | .0 | 99.1 |
|  | 2701 | 6 | .0 | .0 | 99.1 |
|  | 2704 | 8 | .0 | .0 | 99.1 |
|  | 2705 | 16 | .0 | .0 | 99.2 |
|  | 2706 | 7 | .0 | .0 | 99.2 |
|  | 2707 | 12 | .0 | .0 | 99.2 |
|  | 2709 | 9 | .0 | .0 | 99.2 |
|  | 2710 | 10 | .0 | .0 | 99.2 |
|  | 2712 | 8 | .0 | .0 | 99.2 |
|  | 2717 | 9 | .0 | .0 | 99.2 |
|  | 2718 | 11 | .0 | .0 | 99.2 |
|  | 2725 | 1 | .0 | .0 | 99.2 |
|  | 2730 | 12 | .0 | .0 | 99.2 |
|  | 2731 | 6 | .0 | .0 | 99.2 |
|  | 2736 | 11 | .0 | .0 | 99.2 |
|  | 2740 | 8 | .0 | .0 | 99.2 |
|  | 2741 | 2 | .0 | .0 | 99.2 |
|  | 2746 | 7 | .0 | .0 | 99.2 |
|  | 2749 | 4 | .0 | .0 | 99.2 |
|  | 2752 | 6 | .0 | .0 | 99.2 |
|  | 2755 | 11 | .0 | .0 | 99.2 |
|  | 2757 | 10 | .0 | .0 | 99.2 |
|  | 2760 | 9 | .0 | .0 | 99.2 |
|  | 2765 | 7 | .0 | .0 | 99.2 |
|  | 2767 | 11 | .0 | .0 | 99.2 |
|  | 2769 | 3 | .0 | .0 | 99.2 |
|  | 2771 | 9 | .0 | .0 | 99.2 |
|  | 2772 | 19 | .0 | .0 | 99.3 |
|  | 2777 | 8 | .0 | .0 | 99.3 |
|  | 2782 | 1 | .0 | .0 | 99.3 |
|  | 2784 | 13 | .0 | .0 | 99.3 |
|  | 2785 | 5 | .0 | .0 | 99.3 |
|  | 2786 | 7 | .0 | .0 | 99.3 |
|  | 2790 | 8 | .0 | .0 | 99.3 |
|  | 2794 | 14 | .0 | .0 | 99.3 |
|  | 2796 | 7 | .0 | .0 | 99.3 |
|  | 2797 | 7 | .0 | .0 | 99.3 |
|  | 2800 | 3 | .0 | .0 | 99.3 |
|  | 2802 | 12 | .0 | .0 | 99.3 |
|  | 2806 | 1 | .0 | .0 | 99.3 |
|  | 2809 | 7 | .0 | .0 | 99.3 |
|  | 2810 | 6 | .0 | .0 | 99.3 |
|  | 2814 | 7 | .0 | .0 | 99.3 |
|  | 2815 | 1 | .0 | .0 | 99.3 |
|  | 2817 | 7 | .0 | .0 | 99.3 |
|  | 2818 | 8 | .0 | .0 | 99.3 |
|  | 2823 | 7 | .0 | .0 | 99.3 |
|  | 2824 | 5 | .0 | .0 | 99.3 |
|  | 2825 | 15 | .0 | .0 | 99.3 |
|  | 2828 | 8 | .0 | .0 | 99.3 |
|  | 2831 | 7 | .0 | .0 | 99.3 |
|  | 2832 | 6 | .0 | .0 | 99.3 |
|  | 2836 | 12 | .0 | .0 | 99.4 |
|  | 2837 | 14 | .0 | .0 | 99.4 |
|  | 2839 | 4 | .0 | .0 | 99.4 |
|  | 2845 | 7 | .0 | .0 | 99.4 |
|  | 2846 | 8 | .0 | .0 | 99.4 |
|  | 2847 | 7 | .0 | .0 | 99.4 |
|  | 2853 | 7 | .0 | .0 | 99.4 |
|  | 2860 | 7 | .0 | .0 | 99.4 |
|  | 2861 | 9 | .0 | .0 | 99.4 |
|  | 2864 | 8 | .0 | .0 | 99.4 |
|  | 2872 | 7 | .0 | .0 | 99.4 |
|  | 2873 | 2 | .0 | .0 | 99.4 |
|  | 2878 | 4 | .0 | .0 | 99.4 |
|  | 2885 | 6 | .0 | .0 | 99.4 |
|  | 2886 | 1 | .0 | .0 | 99.4 |
|  | 2888 | 3 | .0 | .0 | 99.4 |
|  | 2889 | 6 | .0 | .0 | 99.4 |
|  | 2896 | 11 | .0 | .0 | 99.4 |
|  | 2903 | 7 | .0 | .0 | 99.4 |
|  | 2913 | 6 | .0 | .0 | 99.4 |
|  | 2915 | 7 | .0 | .0 | 99.4 |
|  | 2918 | 1 | .0 | .0 | 99.4 |
|  | 2924 | 15 | .0 | .0 | 99.4 |
|  | 2925 | 1 | .0 | .0 | 99.4 |
|  | 2940 | 1 | .0 | .0 | 99.4 |
|  | 2942 | 11 | .0 | .0 | 99.4 |
|  | 2944 | 5 | .0 | .0 | 99.4 |
|  | 2949 | 11 | .0 | .0 | 99.4 |
|  | 2951 | 5 | .0 | .0 | 99.4 |
|  | 2956 | 5 | .0 | .0 | 99.5 |
|  | 2957 | 9 | .0 | .0 | 99.5 |
|  | 2961 | 11 | .0 | .0 | 99.5 |
|  | 2962 | 6 | .0 | .0 | 99.5 |
|  | 2965 | 5 | .0 | .0 | 99.5 |
|  | 2968 | 13 | .0 | .0 | 99.5 |
|  | 2970 | 6 | .0 | .0 | 99.5 |
|  | 2971 | 9 | .0 | .0 | 99.5 |
|  | 2972 | 7 | .0 | .0 | 99.5 |
|  | 2974 | 6 | .0 | .0 | 99.5 |
|  | 2975 | 8 | .0 | .0 | 99.5 |
|  | 2979 | 7 | .0 | .0 | 99.5 |
|  | 2980 | 4 | .0 | .0 | 99.5 |
|  | 2981 | 9 | .0 | .0 | 99.5 |
|  | 2984 | 10 | .0 | .0 | 99.5 |
|  | 2989 | 1 | .0 | .0 | 99.5 |
|  | 2990 | 2 | .0 | .0 | 99.5 |
|  | 3005 | 6 | .0 | .0 | 99.5 |
|  | 3013 | 5 | .0 | .0 | 99.5 |
|  | 3018 | 6 | .0 | .0 | 99.5 |
|  | 3022 | 5 | .0 | .0 | 99.5 |
|  | 3024 | 16 | .0 | .0 | 99.5 |
|  | 3027 | 7 | .0 | .0 | 99.5 |
|  | 3030 | 8 | .0 | .0 | 99.5 |
|  | 3036 | 6 | .0 | .0 | 99.5 |
|  | 3054 | 8 | .0 | .0 | 99.5 |
|  | 3059 | 10 | .0 | .0 | 99.6 |
|  | 3063 | 4 | .0 | .0 | 99.6 |
|  | 3065 | 3 | .0 | .0 | 99.6 |
|  | 3066 | 4 | .0 | .0 | 99.6 |
|  | 3071 | 4 | .0 | .0 | 99.6 |
|  | 3073 | 1 | .0 | .0 | 99.6 |
|  | 3076 | 4 | .0 | .0 | 99.6 |
|  | 3077 | 4 | .0 | .0 | 99.6 |
|  | 3079 | 8 | .0 | .0 | 99.6 |
|  | 3083 | 8 | .0 | .0 | 99.6 |
|  | 3085 | 7 | .0 | .0 | 99.6 |
|  | 3086 | 13 | .0 | .0 | 99.6 |
|  | 3090 | 8 | .0 | .0 | 99.6 |
|  | 3092 | 8 | .0 | .0 | 99.6 |
|  | 3104 | 8 | .0 | .0 | 99.6 |
|  | 3109 | 6 | .0 | .0 | 99.6 |
|  | 3112 | 6 | .0 | .0 | 99.6 |
|  | 3116 | 7 | .0 | .0 | 99.6 |
|  | 3117 | 4 | .0 | .0 | 99.6 |
|  | 3120 | 7 | .0 | .0 | 99.6 |
|  | 3121 | 6 | .0 | .0 | 99.6 |
|  | 3123 | 1 | .0 | .0 | 99.6 |
|  | 3124 | 5 | .0 | .0 | 99.6 |
|  | 3125 | 4 | .0 | .0 | 99.6 |
|  | 3131 | 7 | .0 | .0 | 99.6 |
|  | 3134 | 1 | .0 | .0 | 99.6 |
|  | 3136 | 8 | .0 | .0 | 99.6 |
|  | 3142 | 7 | .0 | .0 | 99.6 |
|  | 3170 | 9 | .0 | .0 | 99.6 |
|  | 3261 | 5 | .0 | .0 | 99.6 |
|  | 3266 | 7 | .0 | .0 | 99.6 |
|  | 3270 | 8 | .0 | .0 | 99.6 |
|  | 3272 | 4 | .0 | .0 | 99.6 |
|  | 3274 | 5 | .0 | .0 | 99.7 |
|  | 3277 | 1 | .0 | .0 | 99.7 |
|  | 3279 | 5 | .0 | .0 | 99.7 |
|  | 3289 | 4 | .0 | .0 | 99.7 |
|  | 3293 | 5 | .0 | .0 | 99.7 |
|  | 3310 | 3 | .0 | .0 | 99.7 |
|  | 3315 | 7 | .0 | .0 | 99.7 |
|  | 3335 | 8 | .0 | .0 | 99.7 |
|  | 3344 | 8 | .0 | .0 | 99.7 |
|  | 3350 | 9 | .0 | .0 | 99.7 |
|  | 3354 | 9 | .0 | .0 | 99.7 |
|  | 3355 | 6 | .0 | .0 | 99.7 |
|  | 3358 | 8 | .0 | .0 | 99.7 |
|  | 3359 | 7 | .0 | .0 | 99.7 |
|  | 3368 | 9 | .0 | .0 | 99.7 |
|  | 3370 | 8 | .0 | .0 | 99.7 |
|  | 3371 | 7 | .0 | .0 | 99.7 |
|  | 3382 | 5 | .0 | .0 | 99.7 |
|  | 3386 | 14 | .0 | .0 | 99.7 |
|  | 3387 | 7 | .0 | .0 | 99.7 |
|  | 3390 | 16 | .0 | .0 | 99.7 |
|  | 3392 | 8 | .0 | .0 | 99.7 |
|  | 3393 | 7 | .0 | .0 | 99.7 |
|  | 3424 | 8 | .0 | .0 | 99.7 |
|  | 3430 | 8 | .0 | .0 | 99.7 |
|  | 3446 | 8 | .0 | .0 | 99.7 |
|  | 3447 | 6 | .0 | .0 | 99.8 |
|  | 3454 | 7 | .0 | .0 | 99.8 |
|  | 3457 | 8 | .0 | .0 | 99.8 |
|  | 3458 | 7 | .0 | .0 | 99.8 |
|  | 3470 | 7 | .0 | .0 | 99.8 |
|  | 3494 | 4 | .0 | .0 | 99.8 |
|  | 3495 | 6 | .0 | .0 | 99.8 |
|  | 3497 | 7 | .0 | .0 | 99.8 |
|  | 3498 | 8 | .0 | .0 | 99.8 |
|  | 3503 | 6 | .0 | .0 | 99.8 |
|  | 3509 | 6 | .0 | .0 | 99.8 |
|  | 3510 | 7 | .0 | .0 | 99.8 |
|  | 3511 | 7 | .0 | .0 | 99.8 |
|  | 3520 | 8 | .0 | .0 | 99.8 |
|  | 3525 | 7 | .0 | .0 | 99.8 |
|  | 3528 | 8 | .0 | .0 | 99.8 |
|  | 3542 | 7 | .0 | .0 | 99.8 |
|  | 3555 | 8 | .0 | .0 | 99.8 |
|  | 3557 | 1 | .0 | .0 | 99.8 |
|  | 3561 | 6 | .0 | .0 | 99.8 |
|  | 3563 | 14 | .0 | .0 | 99.8 |
|  | 3566 | 8 | .0 | .0 | 99.8 |
|  | 3567 | 9 | .0 | .0 | 99.8 |
|  | 3570 | 8 | .0 | .0 | 99.8 |
|  | 3578 | 23 | .0 | .0 | 99.9 |
|  | 3579 | 7 | .0 | .0 | 99.9 |
|  | 3587 | 11 | .0 | .0 | 99.9 |
|  | 3591 | 9 | .0 | .0 | 99.9 |
|  | 3595 | 6 | .0 | .0 | 99.9 |
|  | 3596 | 5 | .0 | .0 | 99.9 |
|  | 3598 | 7 | .0 | .0 | 99.9 |
|  | 3600 | 8 | .0 | .0 | 99.9 |
|  | 3612 | 8 | .0 | .0 | 99.9 |
|  | 3613 | 1 | .0 | .0 | 99.9 |
|  | 3626 | 7 | .0 | .0 | 99.9 |
|  | 3631 | 5 | .0 | .0 | 99.9 |
|  | 3635 | 8 | .0 | .0 | 99.9 |
|  | 3642 | 6 | .0 | .0 | 99.9 |
|  | 3644 | 3 | .0 | .0 | 99.9 |
|  | 3657 | 8 | .0 | .0 | 99.9 |
|  | 3663 | 2 | .0 | .0 | 99.9 |
|  | 3671 | 10 | .0 | .0 | 99.9 |
|  | 3673 | 9 | .0 | .0 | 99.9 |
|  | 3681 | 9 | .0 | .0 | 99.9 |
|  | 3684 | 4 | .0 | .0 | 99.9 |
|  | 3686 | 5 | .0 | .0 | 99.9 |
|  | 3690 | 7 | .0 | .0 | 99.9 |
|  | 3693 | 7 | .0 | .0 | 99.9 |
|  | 3697 | 7 | .0 | .0 | 99.9 |
|  | 3700 | 5 | .0 | .0 | 99.9 |
|  | 3701 | 10 | .0 | .0 | 99.9 |
|  | 3708 | 5 | .0 | .0 | 99.9 |
|  | 3716 | 1 | .0 | .0 | 99.9 |
|  | 3717 | 9 | .0 | .0 | 100.0 |
|  | 3718 | 7 | .0 | .0 | 100.0 |
|  | 3719 | 5 | .0 | .0 | 100.0 |
|  | 3720 | 4 | .0 | .0 | 100.0 |
|  | 3721 | 6 | .0 | .0 | 100.0 |
|  | 3725 | 10 | .0 | .0 | 100.0 |
|  | 3727 | 9 | .0 | .0 | 100.0 |
|  | 3729 | 8 | .0 | .0 | 100.0 |
|  | 3742 | 9 | .0 | .0 | 100.0 |
|  | 3753 | 6 | .0 | .0 | 100.0 |
|  | 3755 | 9 | .0 | .0 | 100.0 |
|  | 3756 | 1 | .0 | .0 | 100.0 |
|  | 3757 | 6 | .0 | .0 | 100.0 |
|  | 3760 | 1 | .0 | .0 | 100.0 |
|  | 3761 | 4 | .0 | .0 | 100.0 |
|  | 3762 | 6 | .0 | .0 | 100.0 |
|  | 3770 | 4 | .0 | .0 | 100.0 |
|  | Total | 190290 | 100.0 | 100.0 |  |
|  |  |  |  |  |  |

Interviewer codeInterviewer code, table, 1 levels of column headers and 2 levels of row headers, table with 6 columns and 110 rows

|  |  |  |  |  |  |
| --- | --- | --- | --- | --- | --- |
|  | | Frequency | Percent | Valid Percent | Cumulative Percent |
| Valid | 400 | 13 | .0 | .0 | .0 |
|  | 401 | 6892 | 3.6 | 3.6 | 3.6 |
|  | 402 | 7234 | 3.8 | 3.8 | 7.4 |
|  | 403 | 4940 | 2.6 | 2.6 | 10.0 |
|  | 404 | 4122 | 2.2 | 2.2 | 12.2 |
|  | 405 | 2815 | 1.5 | 1.5 | 13.7 |
|  | 406 | 1862 | 1.0 | 1.0 | 14.7 |
|  | 407 | 1900 | 1.0 | 1.0 | 15.6 |
|  | 408 | 1968 | 1.0 | 1.0 | 16.7 |
|  | 409 | 1267 | .7 | .7 | 17.3 |
|  | 410 | 978 | .5 | .5 | 17.9 |
|  | 411 | 548 | .3 | .3 | 18.2 |
|  | 412 | 668 | .4 | .4 | 18.5 |
|  | 413 | 395 | .2 | .2 | 18.7 |
|  | 414 | 236 | .1 | .1 | 18.8 |
|  | 415 | 52 | .0 | .0 | 18.9 |
|  | 416 | 28 | .0 | .0 | 18.9 |
|  | 417 | 88 | .0 | .0 | 18.9 |
|  | 418 | 75 | .0 | .0 | 19.0 |
|  | 419 | 75 | .0 | .0 | 19.0 |
|  | 420 | 13 | .0 | .0 | 19.0 |
|  | 421 | 101 | .1 | .1 | 19.1 |
|  | 500 | 16 | .0 | .0 | 19.1 |
|  | 501 | 12363 | 6.5 | 6.5 | 25.6 |
|  | 502 | 12105 | 6.4 | 6.4 | 31.9 |
|  | 503 | 10325 | 5.4 | 5.4 | 37.4 |
|  | 504 | 8082 | 4.2 | 4.2 | 41.6 |
|  | 505 | 6991 | 3.7 | 3.7 | 45.3 |
|  | 506 | 5688 | 3.0 | 3.0 | 48.3 |
|  | 507 | 5029 | 2.6 | 2.6 | 50.9 |
|  | 508 | 4243 | 2.2 | 2.2 | 53.1 |
|  | 509 | 3818 | 2.0 | 2.0 | 55.1 |
|  | 510 | 3574 | 1.9 | 1.9 | 57.0 |
|  | 511 | 3270 | 1.7 | 1.7 | 58.7 |
|  | 512 | 2648 | 1.4 | 1.4 | 60.1 |
|  | 513 | 2207 | 1.2 | 1.2 | 61.3 |
|  | 514 | 1566 | .8 | .8 | 62.1 |
|  | 515 | 1508 | .8 | .8 | 62.9 |
|  | 516 | 906 | .5 | .5 | 63.4 |
|  | 517 | 864 | .5 | .5 | 63.8 |
|  | 518 | 654 | .3 | .3 | 64.2 |
|  | 519 | 571 | .3 | .3 | 64.5 |
|  | 520 | 470 | .2 | .2 | 64.7 |
|  | 521 | 314 | .2 | .2 | 64.9 |
|  | 522 | 264 | .1 | .1 | 65.0 |
|  | 523 | 247 | .1 | .1 | 65.2 |
|  | 524 | 268 | .1 | .1 | 65.3 |
|  | 525 | 219 | .1 | .1 | 65.4 |
|  | 526 | 354 | .2 | .2 | 65.6 |
|  | 527 | 271 | .1 | .1 | 65.7 |
|  | 528 | 106 | .1 | .1 | 65.8 |
|  | 529 | 80 | .0 | .0 | 65.8 |
|  | 530 | 106 | .1 | .1 | 65.9 |
|  | 531 | 74 | .0 | .0 | 65.9 |
|  | 532 | 99 | .1 | .1 | 66.0 |
|  | 533 | 154 | .1 | .1 | 66.1 |
|  | 534 | 99 | .1 | .1 | 66.1 |
|  | 535 | 147 | .1 | .1 | 66.2 |
|  | 536 | 117 | .1 | .1 | 66.3 |
|  | 537 | 154 | .1 | .1 | 66.3 |
|  | 538 | 82 | .0 | .0 | 66.4 |
|  | 539 | 144 | .1 | .1 | 66.5 |
|  | 540 | 195 | .1 | .1 | 66.6 |
|  | 601 | 12920 | 6.8 | 6.8 | 73.4 |
|  | 602 | 10379 | 5.5 | 5.5 | 78.8 |
|  | 603 | 7340 | 3.9 | 3.9 | 82.7 |
|  | 604 | 4904 | 2.6 | 2.6 | 85.2 |
|  | 605 | 4993 | 2.6 | 2.6 | 87.9 |
|  | 606 | 4006 | 2.1 | 2.1 | 90.0 |
|  | 607 | 2778 | 1.5 | 1.5 | 91.4 |
|  | 608 | 2040 | 1.1 | 1.1 | 92.5 |
|  | 609 | 2292 | 1.2 | 1.2 | 93.7 |
|  | 610 | 1797 | .9 | .9 | 94.7 |
|  | 611 | 1368 | .7 | .7 | 95.4 |
|  | 612 | 1124 | .6 | .6 | 96.0 |
|  | 613 | 717 | .4 | .4 | 96.3 |
|  | 614 | 618 | .3 | .3 | 96.7 |
|  | 615 | 545 | .3 | .3 | 96.9 |
|  | 616 | 489 | .3 | .3 | 97.2 |
|  | 617 | 440 | .2 | .2 | 97.4 |
|  | 618 | 502 | .3 | .3 | 97.7 |
|  | 619 | 471 | .2 | .2 | 97.9 |
|  | 620 | 604 | .3 | .3 | 98.3 |
|  | 621 | 322 | .2 | .2 | 98.4 |
|  | 622 | 395 | .2 | .2 | 98.6 |
|  | 623 | 254 | .1 | .1 | 98.8 |
|  | 624 | 246 | .1 | .1 | 98.9 |
|  | 625 | 81 | .0 | .0 | 98.9 |
|  | 626 | 138 | .1 | .1 | 99.0 |
|  | 627 | 83 | .0 | .0 | 99.1 |
|  | 628 | 119 | .1 | .1 | 99.1 |
|  | 629 | 43 | .0 | .0 | 99.1 |
|  | 630 | 116 | .1 | .1 | 99.2 |
|  | 631 | 65 | .0 | .0 | 99.2 |
|  | 632 | 66 | .0 | .0 | 99.3 |
|  | 633 | 99 | .1 | .1 | 99.3 |
|  | 634 | 62 | .0 | .0 | 99.4 |
|  | 635 | 80 | .0 | .0 | 99.4 |
|  | 636 | 113 | .1 | .1 | 99.5 |
|  | 637 | 138 | .1 | .1 | 99.5 |
|  | 638 | 79 | .0 | .0 | 99.6 |
|  | 639 | 70 | .0 | .0 | 99.6 |
|  | 640 | 193 | .1 | .1 | 99.7 |
|  | 641 | 49 | .0 | .0 | 99.7 |
|  | 660 | 422 | .2 | .2 | 100.0 |
|  | 661 | 12 | .0 | .0 | 100.0 |
|  | 690 | 56 | .0 | .0 | 100.0 |
|  | Total | 190290 | 100.0 | 100.0 |  |
|  |  |  |  |  |  |

Interview result codeInterview result code, table, 1 levels of column headers and 2 levels of row headers, table with 6 columns and 3 rows

|  |  |  |  |  |  |
| --- | --- | --- | --- | --- | --- |
|  | | Frequency | Percent | Valid Percent | Cumulative Percent |
| Valid | Interview completed | 190290 | 100.0 | 100.0 | 100.0 |
|  |  |  |  |  |  |

Serial # of HH membersSerial # of HH members, table, 1 levels of column headers and 2 levels of row headers, table with 6 columns and 22 rows

|  |  |  |  |  |  |
| --- | --- | --- | --- | --- | --- |
|  | | Frequency | Percent | Valid Percent | Cumulative Percent |
| Valid | 2 | 589 | .3 | .3 | .3 |
|  | 3 | 32838 | 17.3 | 17.3 | 17.6 |
|  | 4 | 50852 | 26.7 | 26.7 | 44.3 |
|  | 5 | 42998 | 22.6 | 22.6 | 66.9 |
|  | 6 | 28871 | 15.2 | 15.2 | 82.1 |
|  | 7 | 15676 | 8.2 | 8.2 | 90.3 |
|  | 8 | 8542 | 4.5 | 4.5 | 94.8 |
|  | 9 | 4369 | 2.3 | 2.3 | 97.1 |
|  | 10 | 2327 | 1.2 | 1.2 | 98.3 |
|  | 11 | 1440 | .8 | .8 | 99.1 |
|  | 12 | 834 | .4 | .4 | 99.5 |
|  | 13 | 503 | .3 | .3 | 99.8 |
|  | 14 | 239 | .1 | .1 | 99.9 |
|  | 15 | 104 | .1 | .1 | 99.9 |
|  | 16 | 57 | .0 | .0 | 100.0 |
|  | 17 | 18 | .0 | .0 | 100.0 |
|  | 18 | 16 | .0 | .0 | 100.0 |
|  | 19 | 6 | .0 | .0 | 100.0 |
|  | 21 | 11 | .0 | .0 | 100.0 |
|  | Total | 190290 | 100.0 | 100.0 |  |
|  |  |  |  |  |  |

Caregiver's serial numberCaregiver's serial number, table, 1 levels of column headers and 2 levels of row headers, table with 6 columns and 41 rows

|  |  |  |  |  |  |
| --- | --- | --- | --- | --- | --- |
|  | | Frequency | Percent | Valid Percent | Cumulative Percent |
| Valid | 0 | 2 | .0 | 1.3 | 1.3 |
|  | 1 | 10 | .0 | 6.6 | 7.9 |
|  | 2 | 50 | .0 | 32.9 | 40.8 |
|  | 3 | 16 | .0 | 10.5 | 51.3 |
|  | 4 | 6 | .0 | 3.9 | 55.3 |
|  | 5 | 6 | .0 | 3.9 | 59.2 |
|  | 6 | 4 | .0 | 2.6 | 61.8 |
|  | 7 | 1 | .0 | .7 | 62.5 |
|  | 8 | 2 | .0 | 1.3 | 63.8 |
|  | 9 | 2 | .0 | 1.3 | 65.1 |
|  | 10 | 3 | .0 | 2.0 | 67.1 |
|  | 11 | 1 | .0 | .7 | 67.8 |
|  | 12 | 2 | .0 | 1.3 | 69.1 |
|  | 13 | 3 | .0 | 2.0 | 71.1 |
|  | 14 | 1 | .0 | .7 | 71.7 |
|  | 15 | 2 | .0 | 1.3 | 73.0 |
|  | 16 | 2 | .0 | 1.3 | 74.3 |
|  | 17 | 2 | .0 | 1.3 | 75.7 |
|  | 18 | 6 | .0 | 3.9 | 79.6 |
|  | 19 | 2 | .0 | 1.3 | 80.9 |
|  | 20 | 1 | .0 | .7 | 81.6 |
|  | 21 | 2 | .0 | 1.3 | 82.9 |
|  | 22 | 1 | .0 | .7 | 83.6 |
|  | 23 | 2 | .0 | 1.3 | 84.9 |
|  | 24 | 1 | .0 | .7 | 85.5 |
|  | 25 | 3 | .0 | 2.0 | 87.5 |
|  | 27 | 4 | .0 | 2.6 | 90.1 |
|  | 28 | 2 | .0 | 1.3 | 91.4 |
|  | 29 | 3 | .0 | 2.0 | 93.4 |
|  | 30 | 3 | .0 | 2.0 | 95.4 |
|  | 44 | 1 | .0 | .7 | 96.1 |
|  | 45 | 2 | .0 | 1.3 | 97.4 |
|  | 52 | 1 | .0 | .7 | 98.0 |
|  | 55 | 1 | .0 | .7 | 98.7 |
|  | 60 | 1 | .0 | .7 | 99.3 |
|  | 66 | 1 | .0 | .7 | 100.0 |
|  | Total | 152 | .1 | 100.0 |  |
| Missing | System | 190138 | 99.9 |  |  |
| Total | | 190290 | 100.0 |  |  |
|  |  |  |  |  |  |

Visit numberVisit number, table, 1 levels of column headers and 2 levels of row headers, table with 6 columns and 15 rows

|  |  |  |  |  |  |
| --- | --- | --- | --- | --- | --- |
|  | | Frequency | Percent | Valid Percent | Cumulative Percent |
| Valid | 1 | 30553 | 16.1 | 16.1 | 16.1 |
|  | 2 | 27353 | 14.4 | 14.4 | 30.4 |
|  | 3 | 26059 | 13.7 | 13.7 | 44.1 |
|  | 4 | 24596 | 12.9 | 12.9 | 57.1 |
|  | 5 | 22127 | 11.6 | 11.6 | 68.7 |
|  | 6 | 18769 | 9.9 | 9.9 | 78.5 |
|  | 7 | 15504 | 8.1 | 8.1 | 86.7 |
|  | 8 | 11246 | 5.9 | 5.9 | 92.6 |
|  | 9 | 7197 | 3.8 | 3.8 | 96.4 |
|  | 10 | 3877 | 2.0 | 2.0 | 98.4 |
|  | 11 | 1892 | 1.0 | 1.0 | 99.4 |
|  | 12 | 1117 | .6 | .6 | 100.0 |
|  | Total | 190290 | 100.0 | 100.0 |  |
|  |  |  |  |  |  |

Visit dateVisit date, table, 1 levels of column headers and 2 levels of row headers, table with 6 columns and 551 rows

|  |  |  |  |  |  |
| --- | --- | --- | --- | --- | --- |
|  | | Frequency | Percent | Valid Percent | Cumulative Percent |
| Valid | 30-MAY-2014 | 109 | .1 | .1 | .1 |
|  | 31-MAY-2014 | 267 | .1 | .1 | .2 |
|  | 01-JUN-2014 | 461 | .2 | .2 | .4 |
|  | 02-JUN-2014 | 405 | .2 | .2 | .7 |
|  | 03-JUN-2014 | 489 | .3 | .3 | .9 |
|  | 04-JUN-2014 | 331 | .2 | .2 | 1.1 |
|  | 05-JUN-2014 | 528 | .3 | .3 | 1.4 |
|  | 06-JUN-2014 | 184 | .1 | .1 | 1.5 |
|  | 07-JUN-2014 | 424 | .2 | .2 | 1.7 |
|  | 08-JUN-2014 | 345 | .2 | .2 | 1.9 |
|  | 09-JUN-2014 | 190 | .1 | .1 | 2.0 |
|  | 10-JUN-2014 | 184 | .1 | .1 | 2.1 |
|  | 11-JUN-2014 | 235 | .1 | .1 | 2.2 |
|  | 12-JUN-2014 | 143 | .1 | .1 | 2.3 |
|  | 13-JUN-2014 | 46 | .0 | .0 | 2.3 |
|  | 14-JUN-2014 | 27 | .0 | .0 | 2.3 |
|  | 15-JUN-2014 | 275 | .1 | .1 | 2.4 |
|  | 16-JUN-2014 | 186 | .1 | .1 | 2.5 |
|  | 17-JUN-2014 | 231 | .1 | .1 | 2.7 |
|  | 18-JUN-2014 | 218 | .1 | .1 | 2.8 |
|  | 19-JUN-2014 | 43 | .0 | .0 | 2.8 |
|  | 20-JUN-2014 | 24 | .0 | .0 | 2.8 |
|  | 21-JUN-2014 | 144 | .1 | .1 | 2.9 |
|  | 22-JUN-2014 | 147 | .1 | .1 | 3.0 |
|  | 23-JUN-2014 | 259 | .1 | .1 | 3.1 |
|  | 24-JUN-2014 | 96 | .1 | .1 | 3.1 |
|  | 25-JUN-2014 | 147 | .1 | .1 | 3.2 |
|  | 26-JUN-2014 | 225 | .1 | .1 | 3.3 |
|  | 27-JUN-2014 | 30 | .0 | .0 | 3.4 |
|  | 28-JUN-2014 | 171 | .1 | .1 | 3.4 |
|  | 29-JUN-2014 | 180 | .1 | .1 | 3.5 |
|  | 30-JUN-2014 | 119 | .1 | .1 | 3.6 |
|  | 01-JUL-2014 | 308 | .2 | .2 | 3.8 |
|  | 02-JUL-2014 | 273 | .1 | .1 | 3.9 |
|  | 03-JUL-2014 | 148 | .1 | .1 | 4.0 |
|  | 04-JUL-2014 | 40 | .0 | .0 | 4.0 |
|  | 05-JUL-2014 | 176 | .1 | .1 | 4.1 |
|  | 06-JUL-2014 | 239 | .1 | .1 | 4.2 |
|  | 07-JUL-2014 | 236 | .1 | .1 | 4.4 |
|  | 08-JUL-2014 | 181 | .1 | .1 | 4.4 |
|  | 09-JUL-2014 | 140 | .1 | .1 | 4.5 |
|  | 10-JUL-2014 | 167 | .1 | .1 | 4.6 |
|  | 11-JUL-2014 | 47 | .0 | .0 | 4.6 |
|  | 12-JUL-2014 | 238 | .1 | .1 | 4.8 |
|  | 13-JUL-2014 | 364 | .2 | .2 | 5.0 |
|  | 14-JUL-2014 | 243 | .1 | .1 | 5.1 |
|  | 15-JUL-2014 | 208 | .1 | .1 | 5.2 |
|  | 16-JUL-2014 | 230 | .1 | .1 | 5.3 |
|  | 17-JUL-2014 | 213 | .1 | .1 | 5.4 |
|  | 18-JUL-2014 | 113 | .1 | .1 | 5.5 |
|  | 19-JUL-2014 | 307 | .2 | .2 | 5.6 |
|  | 20-JUL-2014 | 388 | .2 | .2 | 5.8 |
|  | 21-JUL-2014 | 272 | .1 | .1 | 6.0 |
|  | 22-JUL-2014 | 294 | .2 | .2 | 6.1 |
|  | 23-JUL-2014 | 236 | .1 | .1 | 6.3 |
|  | 24-JUL-2014 | 239 | .1 | .1 | 6.4 |
|  | 25-JUL-2014 | 105 | .1 | .1 | 6.4 |
|  | 26-JUL-2014 | 197 | .1 | .1 | 6.6 |
|  | 27-JUL-2014 | 176 | .1 | .1 | 6.6 |
|  | 28-JUL-2014 | 150 | .1 | .1 | 6.7 |
|  | 29-JUL-2014 | 94 | .0 | .0 | 6.8 |
|  | 30-JUL-2014 | 71 | .0 | .0 | 6.8 |
|  | 31-JUL-2014 | 90 | .0 | .0 | 6.9 |
|  | 01-AUG-2014 | 61 | .0 | .0 | 6.9 |
|  | 02-AUG-2014 | 529 | .3 | .3 | 7.2 |
|  | 03-AUG-2014 | 229 | .1 | .1 | 7.3 |
|  | 04-AUG-2014 | 148 | .1 | .1 | 7.4 |
|  | 05-AUG-2014 | 207 | .1 | .1 | 7.5 |
|  | 06-AUG-2014 | 294 | .2 | .2 | 7.6 |
|  | 07-AUG-2014 | 238 | .1 | .1 | 7.8 |
|  | 08-AUG-2014 | 134 | .1 | .1 | 7.8 |
|  | 09-AUG-2014 | 262 | .1 | .1 | 8.0 |
|  | 10-AUG-2014 | 300 | .2 | .2 | 8.1 |
|  | 11-AUG-2014 | 242 | .1 | .1 | 8.2 |
|  | 12-AUG-2014 | 171 | .1 | .1 | 8.3 |
|  | 13-AUG-2014 | 297 | .2 | .2 | 8.5 |
|  | 14-AUG-2014 | 124 | .1 | .1 | 8.6 |
|  | 15-AUG-2014 | 57 | .0 | .0 | 8.6 |
|  | 16-AUG-2014 | 278 | .1 | .1 | 8.7 |
|  | 17-AUG-2014 | 152 | .1 | .1 | 8.8 |
|  | 18-AUG-2014 | 248 | .1 | .1 | 8.9 |
|  | 19-AUG-2014 | 309 | .2 | .2 | 9.1 |
|  | 20-AUG-2014 | 213 | .1 | .1 | 9.2 |
|  | 21-AUG-2014 | 218 | .1 | .1 | 9.3 |
|  | 22-AUG-2014 | 67 | .0 | .0 | 9.4 |
|  | 23-AUG-2014 | 314 | .2 | .2 | 9.5 |
|  | 24-AUG-2014 | 171 | .1 | .1 | 9.6 |
|  | 25-AUG-2014 | 348 | .2 | .2 | 9.8 |
|  | 26-AUG-2014 | 314 | .2 | .2 | 10.0 |
|  | 27-AUG-2014 | 189 | .1 | .1 | 10.1 |
|  | 28-AUG-2014 | 397 | .2 | .2 | 10.3 |
|  | 29-AUG-2014 | 103 | .1 | .1 | 10.3 |
|  | 30-AUG-2014 | 350 | .2 | .2 | 10.5 |
|  | 31-AUG-2014 | 153 | .1 | .1 | 10.6 |
|  | 01-SEP-2014 | 241 | .1 | .1 | 10.7 |
|  | 02-SEP-2014 | 194 | .1 | .1 | 10.8 |
|  | 03-SEP-2014 | 272 | .1 | .1 | 11.0 |
|  | 04-SEP-2014 | 183 | .1 | .1 | 11.1 |
|  | 05-SEP-2014 | 69 | .0 | .0 | 11.1 |
|  | 06-SEP-2014 | 365 | .2 | .2 | 11.3 |
|  | 07-SEP-2014 | 211 | .1 | .1 | 11.4 |
|  | 08-SEP-2014 | 280 | .1 | .1 | 11.5 |
|  | 09-SEP-2014 | 291 | .2 | .2 | 11.7 |
|  | 10-SEP-2014 | 342 | .2 | .2 | 11.9 |
|  | 11-SEP-2014 | 268 | .1 | .1 | 12.0 |
|  | 12-SEP-2014 | 138 | .1 | .1 | 12.1 |
|  | 13-SEP-2014 | 440 | .2 | .2 | 12.3 |
|  | 14-SEP-2014 | 440 | .2 | .2 | 12.6 |
|  | 15-SEP-2014 | 312 | .2 | .2 | 12.7 |
|  | 16-SEP-2014 | 412 | .2 | .2 | 12.9 |
|  | 17-SEP-2014 | 401 | .2 | .2 | 13.1 |
|  | 18-SEP-2014 | 184 | .1 | .1 | 13.2 |
|  | 19-SEP-2014 | 145 | .1 | .1 | 13.3 |
|  | 20-SEP-2014 | 386 | .2 | .2 | 13.5 |
|  | 21-SEP-2014 | 243 | .1 | .1 | 13.7 |
|  | 22-SEP-2014 | 268 | .1 | .1 | 13.8 |
|  | 23-SEP-2014 | 153 | .1 | .1 | 13.9 |
|  | 24-SEP-2014 | 149 | .1 | .1 | 14.0 |
|  | 25-SEP-2014 | 199 | .1 | .1 | 14.1 |
|  | 26-SEP-2014 | 81 | .0 | .0 | 14.1 |
|  | 27-SEP-2014 | 415 | .2 | .2 | 14.3 |
|  | 28-SEP-2014 | 197 | .1 | .1 | 14.4 |
|  | 29-SEP-2014 | 252 | .1 | .1 | 14.6 |
|  | 30-SEP-2014 | 262 | .1 | .1 | 14.7 |
|  | 01-OCT-2014 | 345 | .2 | .2 | 14.9 |
|  | 02-OCT-2014 | 386 | .2 | .2 | 15.1 |
|  | 03-OCT-2014 | 110 | .1 | .1 | 15.1 |
|  | 04-OCT-2014 | 167 | .1 | .1 | 15.2 |
|  | 05-OCT-2014 | 185 | .1 | .1 | 15.3 |
|  | 06-OCT-2014 | 110 | .1 | .1 | 15.4 |
|  | 07-OCT-2014 | 189 | .1 | .1 | 15.5 |
|  | 08-OCT-2014 | 966 | .5 | .5 | 16.0 |
|  | 09-OCT-2014 | 164 | .1 | .1 | 16.1 |
|  | 10-OCT-2014 | 145 | .1 | .1 | 16.1 |
|  | 11-OCT-2014 | 219 | .1 | .1 | 16.3 |
|  | 12-OCT-2014 | 271 | .1 | .1 | 16.4 |
|  | 13-OCT-2014 | 298 | .2 | .2 | 16.6 |
|  | 14-OCT-2014 | 253 | .1 | .1 | 16.7 |
|  | 15-OCT-2014 | 217 | .1 | .1 | 16.8 |
|  | 16-OCT-2014 | 269 | .1 | .1 | 16.9 |
|  | 17-OCT-2014 | 102 | .1 | .1 | 17.0 |
|  | 18-OCT-2014 | 427 | .2 | .2 | 17.2 |
|  | 19-OCT-2014 | 377 | .2 | .2 | 17.4 |
|  | 20-OCT-2014 | 589 | .3 | .3 | 17.7 |
|  | 21-OCT-2014 | 401 | .2 | .2 | 17.9 |
|  | 22-OCT-2014 | 481 | .3 | .3 | 18.2 |
|  | 23-OCT-2014 | 277 | .1 | .1 | 18.3 |
|  | 24-OCT-2014 | 87 | .0 | .0 | 18.4 |
|  | 25-OCT-2014 | 558 | .3 | .3 | 18.7 |
|  | 26-OCT-2014 | 443 | .2 | .2 | 18.9 |
|  | 27-OCT-2014 | 335 | .2 | .2 | 19.1 |
|  | 28-OCT-2014 | 401 | .2 | .2 | 19.3 |
|  | 29-OCT-2014 | 312 | .2 | .2 | 19.5 |
|  | 30-OCT-2014 | 257 | .1 | .1 | 19.6 |
|  | 31-OCT-2014 | 17 | .0 | .0 | 19.6 |
|  | 01-NOV-2014 | 462 | .2 | .2 | 19.9 |
|  | 02-NOV-2014 | 397 | .2 | .2 | 20.1 |
|  | 03-NOV-2014 | 400 | .2 | .2 | 20.3 |
|  | 04-NOV-2014 | 175 | .1 | .1 | 20.4 |
|  | 05-NOV-2014 | 484 | .3 | .3 | 20.6 |
|  | 06-NOV-2014 | 298 | .2 | .2 | 20.8 |
|  | 07-NOV-2014 | 127 | .1 | .1 | 20.8 |
|  | 08-NOV-2014 | 446 | .2 | .2 | 21.1 |
|  | 09-NOV-2014 | 555 | .3 | .3 | 21.4 |
|  | 10-NOV-2014 | 375 | .2 | .2 | 21.6 |
|  | 11-NOV-2014 | 390 | .2 | .2 | 21.8 |
|  | 12-NOV-2014 | 368 | .2 | .2 | 22.0 |
|  | 13-NOV-2014 | 266 | .1 | .1 | 22.1 |
|  | 14-NOV-2014 | 155 | .1 | .1 | 22.2 |
|  | 15-NOV-2014 | 542 | .3 | .3 | 22.5 |
|  | 16-NOV-2014 | 481 | .3 | .3 | 22.7 |
|  | 17-NOV-2014 | 303 | .2 | .2 | 22.9 |
|  | 18-NOV-2014 | 330 | .2 | .2 | 23.1 |
|  | 19-NOV-2014 | 239 | .1 | .1 | 23.2 |
|  | 20-NOV-2014 | 224 | .1 | .1 | 23.3 |
|  | 21-NOV-2014 | 143 | .1 | .1 | 23.4 |
|  | 22-NOV-2014 | 497 | .3 | .3 | 23.6 |
|  | 23-NOV-2014 | 303 | .2 | .2 | 23.8 |
|  | 24-NOV-2014 | 183 | .1 | .1 | 23.9 |
|  | 25-NOV-2014 | 302 | .2 | .2 | 24.0 |
|  | 26-NOV-2014 | 320 | .2 | .2 | 24.2 |
|  | 27-NOV-2014 | 301 | .2 | .2 | 24.4 |
|  | 28-NOV-2014 | 121 | .1 | .1 | 24.4 |
|  | 29-NOV-2014 | 419 | .2 | .2 | 24.7 |
|  | 30-NOV-2014 | 198 | .1 | .1 | 24.8 |
|  | 01-DEC-2014 | 265 | .1 | .1 | 24.9 |
|  | 02-DEC-2014 | 362 | .2 | .2 | 25.1 |
|  | 03-DEC-2014 | 494 | .3 | .3 | 25.4 |
|  | 04-DEC-2014 | 407 | .2 | .2 | 25.6 |
|  | 05-DEC-2014 | 255 | .1 | .1 | 25.7 |
|  | 06-DEC-2014 | 436 | .2 | .2 | 25.9 |
|  | 07-DEC-2014 | 340 | .2 | .2 | 26.1 |
|  | 08-DEC-2014 | 405 | .2 | .2 | 26.3 |
|  | 09-DEC-2014 | 170 | .1 | .1 | 26.4 |
|  | 10-DEC-2014 | 410 | .2 | .2 | 26.6 |
|  | 11-DEC-2014 | 92 | .0 | .0 | 26.7 |
|  | 12-DEC-2014 | 138 | .1 | .1 | 26.7 |
|  | 13-DEC-2014 | 403 | .2 | .2 | 27.0 |
|  | 14-DEC-2014 | 534 | .3 | .3 | 27.2 |
|  | 15-DEC-2014 | 512 | .3 | .3 | 27.5 |
|  | 16-DEC-2014 | 122 | .1 | .1 | 27.6 |
|  | 17-DEC-2014 | 547 | .3 | .3 | 27.9 |
|  | 18-DEC-2014 | 305 | .2 | .2 | 28.0 |
|  | 19-DEC-2014 | 136 | .1 | .1 | 28.1 |
|  | 20-DEC-2014 | 680 | .4 | .4 | 28.4 |
|  | 21-DEC-2014 | 485 | .3 | .3 | 28.7 |
|  | 22-DEC-2014 | 384 | .2 | .2 | 28.9 |
|  | 23-DEC-2014 | 430 | .2 | .2 | 29.1 |
|  | 24-DEC-2014 | 416 | .2 | .2 | 29.3 |
|  | 25-DEC-2014 | 211 | .1 | .1 | 29.5 |
|  | 26-DEC-2014 | 145 | .1 | .1 | 29.5 |
|  | 27-DEC-2014 | 806 | .4 | .4 | 30.0 |
|  | 28-DEC-2014 | 576 | .3 | .3 | 30.3 |
|  | 29-DEC-2014 | 503 | .3 | .3 | 30.5 |
|  | 30-DEC-2014 | 362 | .2 | .2 | 30.7 |
|  | 31-DEC-2014 | 453 | .2 | .2 | 31.0 |
|  | 01-JAN-2015 | 584 | .3 | .3 | 31.3 |
|  | 02-JAN-2015 | 293 | .2 | .2 | 31.4 |
|  | 03-JAN-2015 | 717 | .4 | .4 | 31.8 |
|  | 04-JAN-2015 | 339 | .2 | .2 | 32.0 |
|  | 05-JAN-2015 | 940 | .5 | .5 | 32.5 |
|  | 06-JAN-2015 | 497 | .3 | .3 | 32.7 |
|  | 07-JAN-2015 | 608 | .3 | .3 | 33.0 |
|  | 08-JAN-2015 | 436 | .2 | .2 | 33.3 |
|  | 09-JAN-2015 | 155 | .1 | .1 | 33.4 |
|  | 10-JAN-2015 | 650 | .3 | .3 | 33.7 |
|  | 11-JAN-2015 | 533 | .3 | .3 | 34.0 |
|  | 12-JAN-2015 | 626 | .3 | .3 | 34.3 |
|  | 13-JAN-2015 | 315 | .2 | .2 | 34.5 |
|  | 14-JAN-2015 | 582 | .3 | .3 | 34.8 |
|  | 15-JAN-2015 | 471 | .2 | .2 | 35.0 |
|  | 16-JAN-2015 | 181 | .1 | .1 | 35.1 |
|  | 17-JAN-2015 | 618 | .3 | .3 | 35.4 |
|  | 18-JAN-2015 | 489 | .3 | .3 | 35.7 |
|  | 19-JAN-2015 | 543 | .3 | .3 | 36.0 |
|  | 20-JAN-2015 | 293 | .2 | .2 | 36.1 |
|  | 21-JAN-2015 | 374 | .2 | .2 | 36.3 |
|  | 22-JAN-2015 | 317 | .2 | .2 | 36.5 |
|  | 23-JAN-2015 | 121 | .1 | .1 | 36.6 |
|  | 24-JAN-2015 | 481 | .3 | .3 | 36.8 |
|  | 25-JAN-2015 | 528 | .3 | .3 | 37.1 |
|  | 26-JAN-2015 | 304 | .2 | .2 | 37.3 |
|  | 27-JAN-2015 | 350 | .2 | .2 | 37.4 |
|  | 28-JAN-2015 | 505 | .3 | .3 | 37.7 |
|  | 29-JAN-2015 | 235 | .1 | .1 | 37.8 |
|  | 30-JAN-2015 | 206 | .1 | .1 | 37.9 |
|  | 31-JAN-2015 | 558 | .3 | .3 | 38.2 |
|  | 01-FEB-2015 | 437 | .2 | .2 | 38.5 |
|  | 02-FEB-2015 | 301 | .2 | .2 | 38.6 |
|  | 03-FEB-2015 | 184 | .1 | .1 | 38.7 |
|  | 04-FEB-2015 | 254 | .1 | .1 | 38.8 |
|  | 05-FEB-2015 | 194 | .1 | .1 | 39.0 |
|  | 06-FEB-2015 | 172 | .1 | .1 | 39.0 |
|  | 07-FEB-2015 | 414 | .2 | .2 | 39.3 |
|  | 08-FEB-2015 | 509 | .3 | .3 | 39.5 |
|  | 09-FEB-2015 | 415 | .2 | .2 | 39.7 |
|  | 10-FEB-2015 | 407 | .2 | .2 | 40.0 |
|  | 11-FEB-2015 | 526 | .3 | .3 | 40.2 |
|  | 12-FEB-2015 | 364 | .2 | .2 | 40.4 |
|  | 13-FEB-2015 | 124 | .1 | .1 | 40.5 |
|  | 14-FEB-2015 | 757 | .4 | .4 | 40.9 |
|  | 15-FEB-2015 | 575 | .3 | .3 | 41.2 |
|  | 16-FEB-2015 | 474 | .2 | .2 | 41.4 |
|  | 17-FEB-2015 | 379 | .2 | .2 | 41.6 |
|  | 18-FEB-2015 | 309 | .2 | .2 | 41.8 |
|  | 19-FEB-2015 | 227 | .1 | .1 | 41.9 |
|  | 20-FEB-2015 | 232 | .1 | .1 | 42.0 |
|  | 21-FEB-2015 | 215 | .1 | .1 | 42.2 |
|  | 22-FEB-2015 | 929 | .5 | .5 | 42.6 |
|  | 23-FEB-2015 | 500 | .3 | .3 | 42.9 |
|  | 24-FEB-2015 | 357 | .2 | .2 | 43.1 |
|  | 25-FEB-2015 | 575 | .3 | .3 | 43.4 |
|  | 26-FEB-2015 | 318 | .2 | .2 | 43.6 |
|  | 27-FEB-2015 | 195 | .1 | .1 | 43.7 |
|  | 28-FEB-2015 | 854 | .4 | .4 | 44.1 |
|  | 01-MAR-2015 | 623 | .3 | .3 | 44.4 |
|  | 02-MAR-2015 | 387 | .2 | .2 | 44.6 |
|  | 03-MAR-2015 | 529 | .3 | .3 | 44.9 |
|  | 04-MAR-2015 | 553 | .3 | .3 | 45.2 |
|  | 05-MAR-2015 | 339 | .2 | .2 | 45.4 |
|  | 06-MAR-2015 | 152 | .1 | .1 | 45.5 |
|  | 07-MAR-2015 | 632 | .3 | .3 | 45.8 |
|  | 08-MAR-2015 | 514 | .3 | .3 | 46.1 |
|  | 09-MAR-2015 | 460 | .2 | .2 | 46.3 |
|  | 10-MAR-2015 | 356 | .2 | .2 | 46.5 |
|  | 11-MAR-2015 | 439 | .2 | .2 | 46.7 |
|  | 12-MAR-2015 | 263 | .1 | .1 | 46.9 |
|  | 13-MAR-2015 | 183 | .1 | .1 | 47.0 |
|  | 14-MAR-2015 | 651 | .3 | .3 | 47.3 |
|  | 15-MAR-2015 | 356 | .2 | .2 | 47.5 |
|  | 16-MAR-2015 | 680 | .4 | .4 | 47.9 |
|  | 17-MAR-2015 | 170 | .1 | .1 | 47.9 |
|  | 18-MAR-2015 | 546 | .3 | .3 | 48.2 |
|  | 19-MAR-2015 | 365 | .2 | .2 | 48.4 |
|  | 20-MAR-2015 | 109 | .1 | .1 | 48.5 |
|  | 21-MAR-2015 | 330 | .2 | .2 | 48.7 |
|  | 22-MAR-2015 | 404 | .2 | .2 | 48.9 |
|  | 23-MAR-2015 | 404 | .2 | .2 | 49.1 |
|  | 24-MAR-2015 | 446 | .2 | .2 | 49.3 |
|  | 25-MAR-2015 | 619 | .3 | .3 | 49.6 |
|  | 26-MAR-2015 | 188 | .1 | .1 | 49.7 |
|  | 27-MAR-2015 | 273 | .1 | .1 | 49.9 |
|  | 28-MAR-2015 | 689 | .4 | .4 | 50.2 |
|  | 29-MAR-2015 | 423 | .2 | .2 | 50.5 |
|  | 30-MAR-2015 | 492 | .3 | .3 | 50.7 |
|  | 31-MAR-2015 | 322 | .2 | .2 | 50.9 |
|  | 01-APR-2015 | 292 | .2 | .2 | 51.0 |
|  | 02-APR-2015 | 417 | .2 | .2 | 51.3 |
|  | 03-APR-2015 | 191 | .1 | .1 | 51.4 |
|  | 04-APR-2015 | 692 | .4 | .4 | 51.7 |
|  | 05-APR-2015 | 504 | .3 | .3 | 52.0 |
|  | 06-APR-2015 | 443 | .2 | .2 | 52.2 |
|  | 07-APR-2015 | 368 | .2 | .2 | 52.4 |
|  | 08-APR-2015 | 529 | .3 | .3 | 52.7 |
|  | 09-APR-2015 | 349 | .2 | .2 | 52.9 |
|  | 10-APR-2015 | 244 | .1 | .1 | 53.0 |
|  | 11-APR-2015 | 895 | .5 | .5 | 53.5 |
|  | 12-APR-2015 | 554 | .3 | .3 | 53.8 |
|  | 13-APR-2015 | 495 | .3 | .3 | 54.0 |
|  | 14-APR-2015 | 183 | .1 | .1 | 54.1 |
|  | 15-APR-2015 | 690 | .4 | .4 | 54.5 |
|  | 16-APR-2015 | 268 | .1 | .1 | 54.6 |
|  | 17-APR-2015 | 200 | .1 | .1 | 54.7 |
|  | 18-APR-2015 | 541 | .3 | .3 | 55.0 |
|  | 19-APR-2015 | 704 | .4 | .4 | 55.4 |
|  | 20-APR-2015 | 515 | .3 | .3 | 55.7 |
|  | 21-APR-2015 | 468 | .2 | .2 | 55.9 |
|  | 22-APR-2015 | 553 | .3 | .3 | 56.2 |
|  | 23-APR-2015 | 284 | .1 | .1 | 56.3 |
|  | 24-APR-2015 | 191 | .1 | .1 | 56.4 |
|  | 25-APR-2015 | 1058 | .6 | .6 | 57.0 |
|  | 26-APR-2015 | 581 | .3 | .3 | 57.3 |
|  | 27-APR-2015 | 481 | .3 | .3 | 57.6 |
|  | 28-APR-2015 | 688 | .4 | .4 | 57.9 |
|  | 29-APR-2015 | 455 | .2 | .2 | 58.2 |
|  | 30-APR-2015 | 521 | .3 | .3 | 58.4 |
|  | 01-MAY-2015 | 170 | .1 | .1 | 58.5 |
|  | 02-MAY-2015 | 691 | .4 | .4 | 58.9 |
|  | 03-MAY-2015 | 279 | .1 | .1 | 59.0 |
|  | 04-MAY-2015 | 616 | .3 | .3 | 59.4 |
|  | 05-MAY-2015 | 454 | .2 | .2 | 59.6 |
|  | 06-MAY-2015 | 421 | .2 | .2 | 59.8 |
|  | 07-MAY-2015 | 263 | .1 | .1 | 60.0 |
|  | 08-MAY-2015 | 145 | .1 | .1 | 60.0 |
|  | 09-MAY-2015 | 678 | .4 | .4 | 60.4 |
|  | 10-MAY-2015 | 416 | .2 | .2 | 60.6 |
|  | 11-MAY-2015 | 525 | .3 | .3 | 60.9 |
|  | 12-MAY-2015 | 538 | .3 | .3 | 61.2 |
|  | 13-MAY-2015 | 380 | .2 | .2 | 61.4 |
|  | 14-MAY-2015 | 379 | .2 | .2 | 61.6 |
|  | 15-MAY-2015 | 136 | .1 | .1 | 61.6 |
|  | 16-MAY-2015 | 457 | .2 | .2 | 61.9 |
|  | 17-MAY-2015 | 262 | .1 | .1 | 62.0 |
|  | 18-MAY-2015 | 271 | .1 | .1 | 62.2 |
|  | 19-MAY-2015 | 396 | .2 | .2 | 62.4 |
|  | 20-MAY-2015 | 588 | .3 | .3 | 62.7 |
|  | 21-MAY-2015 | 312 | .2 | .2 | 62.8 |
|  | 22-MAY-2015 | 151 | .1 | .1 | 62.9 |
|  | 23-MAY-2015 | 684 | .4 | .4 | 63.3 |
|  | 24-MAY-2015 | 397 | .2 | .2 | 63.5 |
|  | 25-MAY-2015 | 529 | .3 | .3 | 63.8 |
|  | 26-MAY-2015 | 431 | .2 | .2 | 64.0 |
|  | 27-MAY-2015 | 299 | .2 | .2 | 64.1 |
|  | 28-MAY-2015 | 207 | .1 | .1 | 64.3 |
|  | 29-MAY-2015 | 179 | .1 | .1 | 64.4 |
|  | 30-MAY-2015 | 615 | .3 | .3 | 64.7 |
|  | 31-MAY-2015 | 430 | .2 | .2 | 64.9 |
|  | 01-JUN-2015 | 599 | .3 | .3 | 65.2 |
|  | 02-JUN-2015 | 354 | .2 | .2 | 65.4 |
|  | 03-JUN-2015 | 410 | .2 | .2 | 65.6 |
|  | 04-JUN-2015 | 604 | .3 | .3 | 65.9 |
|  | 05-JUN-2015 | 216 | .1 | .1 | 66.0 |
|  | 06-JUN-2015 | 806 | .4 | .4 | 66.5 |
|  | 07-JUN-2015 | 560 | .3 | .3 | 66.8 |
|  | 08-JUN-2015 | 429 | .2 | .2 | 67.0 |
|  | 09-JUN-2015 | 380 | .2 | .2 | 67.2 |
|  | 10-JUN-2015 | 653 | .3 | .3 | 67.5 |
|  | 11-JUN-2015 | 301 | .2 | .2 | 67.7 |
|  | 12-JUN-2015 | 225 | .1 | .1 | 67.8 |
|  | 13-JUN-2015 | 549 | .3 | .3 | 68.1 |
|  | 14-JUN-2015 | 740 | .4 | .4 | 68.5 |
|  | 15-JUN-2015 | 729 | .4 | .4 | 68.9 |
|  | 16-JUN-2015 | 422 | .2 | .2 | 69.1 |
|  | 17-JUN-2015 | 710 | .4 | .4 | 69.5 |
|  | 18-JUN-2015 | 397 | .2 | .2 | 69.7 |
|  | 19-JUN-2015 | 164 | .1 | .1 | 69.8 |
|  | 20-JUN-2015 | 955 | .5 | .5 | 70.3 |
|  | 21-JUN-2015 | 491 | .3 | .3 | 70.5 |
|  | 22-JUN-2015 | 639 | .3 | .3 | 70.9 |
|  | 23-JUN-2015 | 496 | .3 | .3 | 71.1 |
|  | 24-JUN-2015 | 587 | .3 | .3 | 71.4 |
|  | 25-JUN-2015 | 481 | .3 | .3 | 71.7 |
|  | 26-JUN-2015 | 283 | .1 | .1 | 71.8 |
|  | 27-JUN-2015 | 767 | .4 | .4 | 72.2 |
|  | 28-JUN-2015 | 508 | .3 | .3 | 72.5 |
|  | 29-JUN-2015 | 371 | .2 | .2 | 72.7 |
|  | 30-JUN-2015 | 495 | .3 | .3 | 73.0 |
|  | 01-JUL-2015 | 672 | .4 | .4 | 73.3 |
|  | 02-JUL-2015 | 349 | .2 | .2 | 73.5 |
|  | 03-JUL-2015 | 134 | .1 | .1 | 73.6 |
|  | 04-JUL-2015 | 542 | .3 | .3 | 73.8 |
|  | 05-JUL-2015 | 300 | .2 | .2 | 74.0 |
|  | 06-JUL-2015 | 631 | .3 | .3 | 74.3 |
|  | 07-JUL-2015 | 390 | .2 | .2 | 74.5 |
|  | 08-JUL-2015 | 364 | .2 | .2 | 74.7 |
|  | 09-JUL-2015 | 277 | .1 | .1 | 74.9 |
|  | 10-JUL-2015 | 192 | .1 | .1 | 75.0 |
|  | 11-JUL-2015 | 421 | .2 | .2 | 75.2 |
|  | 12-JUL-2015 | 249 | .1 | .1 | 75.3 |
|  | 13-JUL-2015 | 276 | .1 | .1 | 75.5 |
|  | 14-JUL-2015 | 523 | .3 | .3 | 75.7 |
|  | 15-JUL-2015 | 331 | .2 | .2 | 75.9 |
|  | 16-JUL-2015 | 499 | .3 | .3 | 76.2 |
|  | 17-JUL-2015 | 184 | .1 | .1 | 76.3 |
|  | 18-JUL-2015 | 212 | .1 | .1 | 76.4 |
|  | 19-JUL-2015 | 412 | .2 | .2 | 76.6 |
|  | 20-JUL-2015 | 680 | .4 | .4 | 77.0 |
|  | 21-JUL-2015 | 360 | .2 | .2 | 77.2 |
|  | 22-JUL-2015 | 335 | .2 | .2 | 77.3 |
|  | 23-JUL-2015 | 238 | .1 | .1 | 77.5 |
|  | 24-JUL-2015 | 174 | .1 | .1 | 77.5 |
|  | 25-JUL-2015 | 692 | .4 | .4 | 77.9 |
|  | 26-JUL-2015 | 551 | .3 | .3 | 78.2 |
|  | 27-JUL-2015 | 404 | .2 | .2 | 78.4 |
|  | 28-JUL-2015 | 350 | .2 | .2 | 78.6 |
|  | 29-JUL-2015 | 374 | .2 | .2 | 78.8 |
|  | 30-JUL-2015 | 501 | .3 | .3 | 79.1 |
|  | 31-JUL-2015 | 74 | .0 | .0 | 79.1 |
|  | 01-AUG-2015 | 806 | .4 | .4 | 79.5 |
|  | 02-AUG-2015 | 454 | .2 | .2 | 79.8 |
|  | 03-AUG-2015 | 393 | .2 | .2 | 80.0 |
|  | 04-AUG-2015 | 423 | .2 | .2 | 80.2 |
|  | 05-AUG-2015 | 458 | .2 | .2 | 80.4 |
|  | 06-AUG-2015 | 220 | .1 | .1 | 80.5 |
|  | 07-AUG-2015 | 240 | .1 | .1 | 80.7 |
|  | 08-AUG-2015 | 763 | .4 | .4 | 81.1 |
|  | 09-AUG-2015 | 532 | .3 | .3 | 81.3 |
|  | 10-AUG-2015 | 519 | .3 | .3 | 81.6 |
|  | 11-AUG-2015 | 342 | .2 | .2 | 81.8 |
|  | 12-AUG-2015 | 534 | .3 | .3 | 82.1 |
|  | 13-AUG-2015 | 468 | .2 | .2 | 82.3 |
|  | 14-AUG-2015 | 161 | .1 | .1 | 82.4 |
|  | 15-AUG-2015 | 396 | .2 | .2 | 82.6 |
|  | 16-AUG-2015 | 954 | .5 | .5 | 83.1 |
|  | 17-AUG-2015 | 469 | .2 | .2 | 83.4 |
|  | 18-AUG-2015 | 664 | .3 | .3 | 83.7 |
|  | 19-AUG-2015 | 440 | .2 | .2 | 83.9 |
|  | 20-AUG-2015 | 453 | .2 | .2 | 84.2 |
|  | 21-AUG-2015 | 180 | .1 | .1 | 84.3 |
|  | 22-AUG-2015 | 846 | .4 | .4 | 84.7 |
|  | 23-AUG-2015 | 554 | .3 | .3 | 85.0 |
|  | 24-AUG-2015 | 339 | .2 | .2 | 85.2 |
|  | 25-AUG-2015 | 435 | .2 | .2 | 85.4 |
|  | 26-AUG-2015 | 359 | .2 | .2 | 85.6 |
|  | 27-AUG-2015 | 451 | .2 | .2 | 85.8 |
|  | 28-AUG-2015 | 174 | .1 | .1 | 85.9 |
|  | 29-AUG-2015 | 425 | .2 | .2 | 86.2 |
|  | 30-AUG-2015 | 392 | .2 | .2 | 86.4 |
|  | 31-AUG-2015 | 429 | .2 | .2 | 86.6 |
|  | 01-SEP-2015 | 330 | .2 | .2 | 86.8 |
|  | 02-SEP-2015 | 559 | .3 | .3 | 87.1 |
|  | 03-SEP-2015 | 437 | .2 | .2 | 87.3 |
|  | 04-SEP-2015 | 181 | .1 | .1 | 87.4 |
|  | 05-SEP-2015 | 340 | .2 | .2 | 87.6 |
|  | 06-SEP-2015 | 393 | .2 | .2 | 87.8 |
|  | 07-SEP-2015 | 372 | .2 | .2 | 88.0 |
|  | 08-SEP-2015 | 222 | .1 | .1 | 88.1 |
|  | 09-SEP-2015 | 367 | .2 | .2 | 88.3 |
|  | 10-SEP-2015 | 328 | .2 | .2 | 88.5 |
|  | 11-SEP-2015 | 136 | .1 | .1 | 88.5 |
|  | 12-SEP-2015 | 502 | .3 | .3 | 88.8 |
|  | 13-SEP-2015 | 319 | .2 | .2 | 89.0 |
|  | 14-SEP-2015 | 469 | .2 | .2 | 89.2 |
|  | 15-SEP-2015 | 358 | .2 | .2 | 89.4 |
|  | 16-SEP-2015 | 534 | .3 | .3 | 89.7 |
|  | 17-SEP-2015 | 274 | .1 | .1 | 89.8 |
|  | 18-SEP-2015 | 231 | .1 | .1 | 89.9 |
|  | 19-SEP-2015 | 516 | .3 | .3 | 90.2 |
|  | 20-SEP-2015 | 444 | .2 | .2 | 90.4 |
|  | 21-SEP-2015 | 429 | .2 | .2 | 90.7 |
|  | 22-SEP-2015 | 497 | .3 | .3 | 90.9 |
|  | 23-SEP-2015 | 491 | .3 | .3 | 91.2 |
|  | 24-SEP-2015 | 278 | .1 | .1 | 91.3 |
|  | 25-SEP-2015 | 118 | .1 | .1 | 91.4 |
|  | 26-SEP-2015 | 506 | .3 | .3 | 91.7 |
|  | 27-SEP-2015 | 812 | .4 | .4 | 92.1 |
|  | 28-SEP-2015 | 481 | .3 | .3 | 92.3 |
|  | 29-SEP-2015 | 370 | .2 | .2 | 92.5 |
|  | 30-SEP-2015 | 534 | .3 | .3 | 92.8 |
|  | 01-OCT-2015 | 270 | .1 | .1 | 93.0 |
|  | 02-OCT-2015 | 196 | .1 | .1 | 93.1 |
|  | 03-OCT-2015 | 587 | .3 | .3 | 93.4 |
|  | 04-OCT-2015 | 485 | .3 | .3 | 93.6 |
|  | 05-OCT-2015 | 402 | .2 | .2 | 93.8 |
|  | 06-OCT-2015 | 403 | .2 | .2 | 94.0 |
|  | 07-OCT-2015 | 419 | .2 | .2 | 94.3 |
|  | 08-OCT-2015 | 480 | .3 | .3 | 94.5 |
|  | 09-OCT-2015 | 151 | .1 | .1 | 94.6 |
|  | 10-OCT-2015 | 675 | .4 | .4 | 94.9 |
|  | 11-OCT-2015 | 436 | .2 | .2 | 95.2 |
|  | 12-OCT-2015 | 550 | .3 | .3 | 95.5 |
|  | 13-OCT-2015 | 565 | .3 | .3 | 95.8 |
|  | 14-OCT-2015 | 479 | .3 | .3 | 96.0 |
|  | 15-OCT-2015 | 344 | .2 | .2 | 96.2 |
|  | 16-OCT-2015 | 243 | .1 | .1 | 96.3 |
|  | 17-OCT-2015 | 642 | .3 | .3 | 96.7 |
|  | 18-OCT-2015 | 485 | .3 | .3 | 96.9 |
|  | 19-OCT-2015 | 366 | .2 | .2 | 97.1 |
|  | 20-OCT-2015 | 332 | .2 | .2 | 97.3 |
|  | 21-OCT-2015 | 441 | .2 | .2 | 97.5 |
|  | 22-OCT-2015 | 288 | .2 | .2 | 97.7 |
|  | 23-OCT-2015 | 192 | .1 | .1 | 97.8 |
|  | 24-OCT-2015 | 309 | .2 | .2 | 97.9 |
|  | 25-OCT-2015 | 682 | .4 | .4 | 98.3 |
|  | 26-OCT-2015 | 347 | .2 | .2 | 98.5 |
|  | 27-OCT-2015 | 333 | .2 | .2 | 98.6 |
|  | 28-OCT-2015 | 322 | .2 | .2 | 98.8 |
|  | 29-OCT-2015 | 471 | .2 | .2 | 99.1 |
|  | 30-OCT-2015 | 224 | .1 | .1 | 99.2 |
|  | 31-OCT-2015 | 669 | .4 | .4 | 99.5 |
|  | 01-NOV-2015 | 73 | .0 | .0 | 99.6 |
|  | 02-NOV-2015 | 52 | .0 | .0 | 99.6 |
|  | 03-NOV-2015 | 15 | .0 | .0 | 99.6 |
|  | 04-NOV-2015 | 44 | .0 | .0 | 99.6 |
|  | 05-NOV-2015 | 12 | .0 | .0 | 99.6 |
|  | 07-NOV-2015 | 1 | .0 | .0 | 99.6 |
|  | 08-NOV-2015 | 17 | .0 | .0 | 99.6 |
|  | 09-NOV-2015 | 31 | .0 | .0 | 99.7 |
|  | 10-NOV-2015 | 34 | .0 | .0 | 99.7 |
|  | 11-NOV-2015 | 27 | .0 | .0 | 99.7 |
|  | 12-NOV-2015 | 70 | .0 | .0 | 99.7 |
|  | 13-NOV-2015 | 11 | .0 | .0 | 99.7 |
|  | 14-NOV-2015 | 49 | .0 | .0 | 99.8 |
|  | 15-NOV-2015 | 86 | .0 | .0 | 99.8 |
|  | 16-NOV-2015 | 65 | .0 | .0 | 99.8 |
|  | 17-NOV-2015 | 12 | .0 | .0 | 99.8 |
|  | 18-NOV-2015 | 12 | .0 | .0 | 99.9 |
|  | 19-NOV-2015 | 38 | .0 | .0 | 99.9 |
|  | 21-NOV-2015 | 2 | .0 | .0 | 99.9 |
|  | 22-NOV-2015 | 48 | .0 | .0 | 99.9 |
|  | 23-NOV-2015 | 58 | .0 | .0 | 99.9 |
|  | 24-NOV-2015 | 20 | .0 | .0 | 99.9 |
|  | 25-NOV-2015 | 2 | .0 | .0 | 99.9 |
|  | 26-NOV-2015 | 18 | .0 | .0 | 100.0 |
|  | 27-NOV-2015 | 15 | .0 | .0 | 100.0 |
|  | 28-NOV-2015 | 13 | .0 | .0 | 100.0 |
|  | 29-NOV-2015 | 12 | .0 | .0 | 100.0 |
|  | 30-NOV-2015 | 54 | .0 | .0 | 100.0 |
|  | Total | 190290 | 100.0 | 100.0 |  |
|  |  |  |  |  |  |

Date of birth of individualDate of birth of individual, table, 1 levels of column headers and 2 levels of row headers, table with 6 columns and 1944 rows

|  |  |  |  |  |  |
| --- | --- | --- | --- | --- | --- |
|  | | Frequency | Percent | Valid Percent | Cumulative Percent |
| Valid | 14-JUN-2009 | 1 | .0 | .0 | .0 |
|  | 15-JUN-2009 | 2 | .0 | .0 | .0 |
|  | 16-JUN-2009 | 2 | .0 | .0 | .0 |
|  | 28-JUN-2009 | 1 | .0 | .0 | .0 |
|  | 02-JUL-2009 | 8 | .0 | .0 | .0 |
|  | 03-JUL-2009 | 12 | .0 | .0 | .0 |
|  | 05-JUL-2009 | 12 | .0 | .0 | .0 |
|  | 09-JUL-2009 | 3 | .0 | .0 | .0 |
|  | 10-JUL-2009 | 1 | .0 | .0 | .0 |
|  | 12-JUL-2009 | 11 | .0 | .0 | .0 |
|  | 13-JUL-2009 | 4 | .0 | .0 | .0 |
|  | 14-JUL-2009 | 14 | .0 | .0 | .0 |
|  | 15-JUL-2009 | 11 | .0 | .0 | .0 |
|  | 17-JUL-2009 | 4 | .0 | .0 | .0 |
|  | 18-JUL-2009 | 7 | .0 | .0 | .0 |
|  | 23-JUL-2009 | 1 | .0 | .0 | .0 |
|  | 25-JUL-2009 | 13 | .0 | .0 | .1 |
|  | 27-JUL-2009 | 2 | .0 | .0 | .1 |
|  | 29-JUL-2009 | 9 | .0 | .0 | .1 |
|  | 01-AUG-2009 | 19 | .0 | .0 | .1 |
|  | 02-AUG-2009 | 5 | .0 | .0 | .1 |
|  | 04-AUG-2009 | 3 | .0 | .0 | .1 |
|  | 05-AUG-2009 | 21 | .0 | .0 | .1 |
|  | 07-AUG-2009 | 6 | .0 | .0 | .1 |
|  | 08-AUG-2009 | 4 | .0 | .0 | .1 |
|  | 10-AUG-2009 | 29 | .0 | .0 | .1 |
|  | 13-AUG-2009 | 7 | .0 | .0 | .1 |
|  | 15-AUG-2009 | 10 | .0 | .0 | .1 |
|  | 17-AUG-2009 | 9 | .0 | .0 | .1 |
|  | 19-AUG-2009 | 2 | .0 | .0 | .1 |
|  | 20-AUG-2009 | 4 | .0 | .0 | .1 |
|  | 21-AUG-2009 | 7 | .0 | .0 | .1 |
|  | 22-AUG-2009 | 3 | .0 | .0 | .1 |
|  | 26-AUG-2009 | 3 | .0 | .0 | .1 |
|  | 27-AUG-2009 | 8 | .0 | .0 | .1 |
|  | 28-AUG-2009 | 4 | .0 | .0 | .1 |
|  | 29-AUG-2009 | 3 | .0 | .0 | .1 |
|  | 31-AUG-2009 | 7 | .0 | .0 | .1 |
|  | 01-SEP-2009 | 31 | .0 | .0 | .2 |
|  | 02-SEP-2009 | 2 | .0 | .0 | .2 |
|  | 03-SEP-2009 | 1 | .0 | .0 | .2 |
|  | 04-SEP-2009 | 10 | .0 | .0 | .2 |
|  | 05-SEP-2009 | 7 | .0 | .0 | .2 |
|  | 06-SEP-2009 | 1 | .0 | .0 | .2 |
|  | 07-SEP-2009 | 20 | .0 | .0 | .2 |
|  | 08-SEP-2009 | 13 | .0 | .0 | .2 |
|  | 09-SEP-2009 | 9 | .0 | .0 | .2 |
|  | 10-SEP-2009 | 17 | .0 | .0 | .2 |
|  | 11-SEP-2009 | 3 | .0 | .0 | .2 |
|  | 14-SEP-2009 | 1 | .0 | .0 | .2 |
|  | 15-SEP-2009 | 6 | .0 | .0 | .2 |
|  | 16-SEP-2009 | 2 | .0 | .0 | .2 |
|  | 20-SEP-2009 | 4 | .0 | .0 | .2 |
|  | 21-SEP-2009 | 1 | .0 | .0 | .2 |
|  | 23-SEP-2009 | 1 | .0 | .0 | .2 |
|  | 24-SEP-2009 | 13 | .0 | .0 | .2 |
|  | 25-SEP-2009 | 5 | .0 | .0 | .2 |
|  | 27-SEP-2009 | 2 | .0 | .0 | .2 |
|  | 28-SEP-2009 | 11 | .0 | .0 | .2 |
|  | 30-SEP-2009 | 8 | .0 | .0 | .2 |
|  | 01-OCT-2009 | 3 | .0 | .0 | .2 |
|  | 05-OCT-2009 | 4 | .0 | .0 | .2 |
|  | 07-OCT-2009 | 5 | .0 | .0 | .2 |
|  | 08-OCT-2009 | 2 | .0 | .0 | .2 |
|  | 10-OCT-2009 | 9 | .0 | .0 | .2 |
|  | 12-OCT-2009 | 1 | .0 | .0 | .2 |
|  | 15-OCT-2009 | 1 | .0 | .0 | .2 |
|  | 16-OCT-2009 | 1 | .0 | .0 | .2 |
|  | 17-OCT-2009 | 9 | .0 | .0 | .2 |
|  | 18-OCT-2009 | 7 | .0 | .0 | .3 |
|  | 27-OCT-2009 | 5 | .0 | .0 | .3 |
|  | 28-OCT-2009 | 3 | .0 | .0 | .3 |
|  | 30-OCT-2009 | 10 | .0 | .0 | .3 |
|  | 01-NOV-2009 | 7 | .0 | .0 | .3 |
|  | 03-NOV-2009 | 10 | .0 | .0 | .3 |
|  | 09-NOV-2009 | 1 | .0 | .0 | .3 |
|  | 10-NOV-2009 | 1 | .0 | .0 | .3 |
|  | 13-NOV-2009 | 1 | .0 | .0 | .3 |
|  | 14-NOV-2009 | 1 | .0 | .0 | .3 |
|  | 15-NOV-2009 | 15 | .0 | .0 | .3 |
|  | 20-NOV-2009 | 7 | .0 | .0 | .3 |
|  | 23-NOV-2009 | 1 | .0 | .0 | .3 |
|  | 25-NOV-2009 | 1 | .0 | .0 | .3 |
|  | 27-NOV-2009 | 12 | .0 | .0 | .3 |
|  | 29-NOV-2009 | 6 | .0 | .0 | .3 |
|  | 30-NOV-2009 | 26 | .0 | .0 | .3 |
|  | 01-DEC-2009 | 12 | .0 | .0 | .3 |
|  | 02-DEC-2009 | 7 | .0 | .0 | .3 |
|  | 04-DEC-2009 | 2 | .0 | .0 | .3 |
|  | 05-DEC-2009 | 12 | .0 | .0 | .3 |
|  | 06-DEC-2009 | 1 | .0 | .0 | .3 |
|  | 08-DEC-2009 | 8 | .0 | .0 | .3 |
|  | 09-DEC-2009 | 1 | .0 | .0 | .3 |
|  | 10-DEC-2009 | 4 | .0 | .0 | .3 |
|  | 12-DEC-2009 | 9 | .0 | .0 | .3 |
|  | 14-DEC-2009 | 1 | .0 | .0 | .3 |
|  | 15-DEC-2009 | 1 | .0 | .0 | .3 |
|  | 16-DEC-2009 | 14 | .0 | .0 | .3 |
|  | 18-DEC-2009 | 3 | .0 | .0 | .3 |
|  | 19-DEC-2009 | 8 | .0 | .0 | .4 |
|  | 21-DEC-2009 | 2 | .0 | .0 | .4 |
|  | 22-DEC-2009 | 5 | .0 | .0 | .4 |
|  | 25-DEC-2009 | 6 | .0 | .0 | .4 |
|  | 28-DEC-2009 | 7 | .0 | .0 | .4 |
|  | 31-DEC-2009 | 3 | .0 | .0 | .4 |
|  | 01-JAN-2010 | 67 | .0 | .0 | .4 |
|  | 02-JAN-2010 | 57 | .0 | .0 | .4 |
|  | 03-JAN-2010 | 13 | .0 | .0 | .4 |
|  | 04-JAN-2010 | 13 | .0 | .0 | .4 |
|  | 05-JAN-2010 | 25 | .0 | .0 | .5 |
|  | 06-JAN-2010 | 12 | .0 | .0 | .5 |
|  | 07-JAN-2010 | 19 | .0 | .0 | .5 |
|  | 08-JAN-2010 | 7 | .0 | .0 | .5 |
|  | 09-JAN-2010 | 8 | .0 | .0 | .5 |
|  | 10-JAN-2010 | 11 | .0 | .0 | .5 |
|  | 11-JAN-2010 | 28 | .0 | .0 | .5 |
|  | 13-JAN-2010 | 1 | .0 | .0 | .5 |
|  | 15-JAN-2010 | 8 | .0 | .0 | .5 |
|  | 17-JAN-2010 | 7 | .0 | .0 | .5 |
|  | 18-JAN-2010 | 5 | .0 | .0 | .5 |
|  | 19-JAN-2010 | 13 | .0 | .0 | .5 |
|  | 20-JAN-2010 | 4 | .0 | .0 | .5 |
|  | 21-JAN-2010 | 9 | .0 | .0 | .5 |
|  | 24-JAN-2010 | 14 | .0 | .0 | .5 |
|  | 26-JAN-2010 | 18 | .0 | .0 | .5 |
|  | 27-JAN-2010 | 18 | .0 | .0 | .6 |
|  | 28-JAN-2010 | 6 | .0 | .0 | .6 |
|  | 30-JAN-2010 | 14 | .0 | .0 | .6 |
|  | 01-FEB-2010 | 63 | .0 | .0 | .6 |
|  | 02-FEB-2010 | 7 | .0 | .0 | .6 |
|  | 03-FEB-2010 | 35 | .0 | .0 | .6 |
|  | 04-FEB-2010 | 16 | .0 | .0 | .6 |
|  | 05-FEB-2010 | 32 | .0 | .0 | .6 |
|  | 06-FEB-2010 | 20 | .0 | .0 | .7 |
|  | 07-FEB-2010 | 10 | .0 | .0 | .7 |
|  | 08-FEB-2010 | 13 | .0 | .0 | .7 |
|  | 09-FEB-2010 | 47 | .0 | .0 | .7 |
|  | 10-FEB-2010 | 61 | .0 | .0 | .7 |
|  | 11-FEB-2010 | 15 | .0 | .0 | .7 |
|  | 12-FEB-2010 | 32 | .0 | .0 | .7 |
|  | 13-FEB-2010 | 24 | .0 | .0 | .8 |
|  | 15-FEB-2010 | 28 | .0 | .0 | .8 |
|  | 16-FEB-2010 | 7 | .0 | .0 | .8 |
|  | 17-FEB-2010 | 7 | .0 | .0 | .8 |
|  | 19-FEB-2010 | 8 | .0 | .0 | .8 |
|  | 20-FEB-2010 | 15 | .0 | .0 | .8 |
|  | 21-FEB-2010 | 7 | .0 | .0 | .8 |
|  | 22-FEB-2010 | 12 | .0 | .0 | .8 |
|  | 23-FEB-2010 | 8 | .0 | .0 | .8 |
|  | 24-FEB-2010 | 1 | .0 | .0 | .8 |
|  | 25-FEB-2010 | 5 | .0 | .0 | .8 |
|  | 26-FEB-2010 | 12 | .0 | .0 | .8 |
|  | 28-FEB-2010 | 17 | .0 | .0 | .8 |
|  | 01-MAR-2010 | 33 | .0 | .0 | .8 |
|  | 02-MAR-2010 | 30 | .0 | .0 | .9 |
|  | 03-MAR-2010 | 6 | .0 | .0 | .9 |
|  | 04-MAR-2010 | 10 | .0 | .0 | .9 |
|  | 05-MAR-2010 | 18 | .0 | .0 | .9 |
|  | 06-MAR-2010 | 1 | .0 | .0 | .9 |
|  | 08-MAR-2010 | 6 | .0 | .0 | .9 |
|  | 09-MAR-2010 | 18 | .0 | .0 | .9 |
|  | 10-MAR-2010 | 35 | .0 | .0 | .9 |
|  | 12-MAR-2010 | 25 | .0 | .0 | .9 |
|  | 13-MAR-2010 | 6 | .0 | .0 | .9 |
|  | 14-MAR-2010 | 8 | .0 | .0 | .9 |
|  | 15-MAR-2010 | 1 | .0 | .0 | .9 |
|  | 16-MAR-2010 | 10 | .0 | .0 | .9 |
|  | 17-MAR-2010 | 3 | .0 | .0 | .9 |
|  | 20-MAR-2010 | 11 | .0 | .0 | .9 |
|  | 21-MAR-2010 | 12 | .0 | .0 | .9 |
|  | 22-MAR-2010 | 26 | .0 | .0 | 1.0 |
|  | 23-MAR-2010 | 1 | .0 | .0 | 1.0 |
|  | 24-MAR-2010 | 13 | .0 | .0 | 1.0 |
|  | 25-MAR-2010 | 3 | .0 | .0 | 1.0 |
|  | 26-MAR-2010 | 2 | .0 | .0 | 1.0 |
|  | 27-MAR-2010 | 30 | .0 | .0 | 1.0 |
|  | 01-APR-2010 | 21 | .0 | .0 | 1.0 |
|  | 02-APR-2010 | 5 | .0 | .0 | 1.0 |
|  | 03-APR-2010 | 8 | .0 | .0 | 1.0 |
|  | 04-APR-2010 | 24 | .0 | .0 | 1.0 |
|  | 05-APR-2010 | 4 | .0 | .0 | 1.0 |
|  | 07-APR-2010 | 23 | .0 | .0 | 1.0 |
|  | 08-APR-2010 | 5 | .0 | .0 | 1.0 |
|  | 09-APR-2010 | 10 | .0 | .0 | 1.0 |
|  | 10-APR-2010 | 41 | .0 | .0 | 1.1 |
|  | 11-APR-2010 | 29 | .0 | .0 | 1.1 |
|  | 13-APR-2010 | 9 | .0 | .0 | 1.1 |
|  | 14-APR-2010 | 14 | .0 | .0 | 1.1 |
|  | 16-APR-2010 | 13 | .0 | .0 | 1.1 |
|  | 17-APR-2010 | 21 | .0 | .0 | 1.1 |
|  | 18-APR-2010 | 8 | .0 | .0 | 1.1 |
|  | 19-APR-2010 | 4 | .0 | .0 | 1.1 |
|  | 20-APR-2010 | 17 | .0 | .0 | 1.1 |
|  | 21-APR-2010 | 1 | .0 | .0 | 1.1 |
|  | 22-APR-2010 | 2 | .0 | .0 | 1.1 |
|  | 24-APR-2010 | 7 | .0 | .0 | 1.1 |
|  | 25-APR-2010 | 16 | .0 | .0 | 1.1 |
|  | 28-APR-2010 | 29 | .0 | .0 | 1.2 |
|  | 29-APR-2010 | 7 | .0 | .0 | 1.2 |
|  | 30-APR-2010 | 8 | .0 | .0 | 1.2 |
|  | 01-MAY-2010 | 30 | .0 | .0 | 1.2 |
|  | 02-MAY-2010 | 10 | .0 | .0 | 1.2 |
|  | 03-MAY-2010 | 13 | .0 | .0 | 1.2 |
|  | 04-MAY-2010 | 26 | .0 | .0 | 1.2 |
|  | 05-MAY-2010 | 7 | .0 | .0 | 1.2 |
|  | 06-MAY-2010 | 18 | .0 | .0 | 1.2 |
|  | 07-MAY-2010 | 10 | .0 | .0 | 1.2 |
|  | 08-MAY-2010 | 7 | .0 | .0 | 1.2 |
|  | 09-MAY-2010 | 22 | .0 | .0 | 1.2 |
|  | 10-MAY-2010 | 12 | .0 | .0 | 1.2 |
|  | 11-MAY-2010 | 17 | .0 | .0 | 1.3 |
|  | 12-MAY-2010 | 14 | .0 | .0 | 1.3 |
|  | 14-MAY-2010 | 10 | .0 | .0 | 1.3 |
|  | 15-MAY-2010 | 8 | .0 | .0 | 1.3 |
|  | 18-MAY-2010 | 14 | .0 | .0 | 1.3 |
|  | 19-MAY-2010 | 16 | .0 | .0 | 1.3 |
|  | 20-MAY-2010 | 21 | .0 | .0 | 1.3 |
|  | 21-MAY-2010 | 2 | .0 | .0 | 1.3 |
|  | 22-MAY-2010 | 1 | .0 | .0 | 1.3 |
|  | 23-MAY-2010 | 17 | .0 | .0 | 1.3 |
|  | 24-MAY-2010 | 11 | .0 | .0 | 1.3 |
|  | 25-MAY-2010 | 18 | .0 | .0 | 1.3 |
|  | 27-MAY-2010 | 20 | .0 | .0 | 1.3 |
|  | 28-MAY-2010 | 18 | .0 | .0 | 1.3 |
|  | 29-MAY-2010 | 32 | .0 | .0 | 1.4 |
|  | 30-MAY-2010 | 9 | .0 | .0 | 1.4 |
|  | 01-JUN-2010 | 102 | .1 | .1 | 1.4 |
|  | 02-JUN-2010 | 12 | .0 | .0 | 1.4 |
|  | 03-JUN-2010 | 45 | .0 | .0 | 1.4 |
|  | 04-JUN-2010 | 10 | .0 | .0 | 1.5 |
|  | 05-JUN-2010 | 21 | .0 | .0 | 1.5 |
|  | 06-JUN-2010 | 22 | .0 | .0 | 1.5 |
|  | 07-JUN-2010 | 69 | .0 | .0 | 1.5 |
|  | 08-JUN-2010 | 11 | .0 | .0 | 1.5 |
|  | 09-JUN-2010 | 20 | .0 | .0 | 1.5 |
|  | 10-JUN-2010 | 52 | .0 | .0 | 1.6 |
|  | 11-JUN-2010 | 18 | .0 | .0 | 1.6 |
|  | 12-JUN-2010 | 41 | .0 | .0 | 1.6 |
|  | 13-JUN-2010 | 12 | .0 | .0 | 1.6 |
|  | 14-JUN-2010 | 23 | .0 | .0 | 1.6 |
|  | 15-JUN-2010 | 3 | .0 | .0 | 1.6 |
|  | 16-JUN-2010 | 11 | .0 | .0 | 1.6 |
|  | 17-JUN-2010 | 1 | .0 | .0 | 1.6 |
|  | 18-JUN-2010 | 9 | .0 | .0 | 1.6 |
|  | 19-JUN-2010 | 18 | .0 | .0 | 1.6 |
|  | 20-JUN-2010 | 87 | .0 | .0 | 1.7 |
|  | 21-JUN-2010 | 35 | .0 | .0 | 1.7 |
|  | 22-JUN-2010 | 17 | .0 | .0 | 1.7 |
|  | 23-JUN-2010 | 24 | .0 | .0 | 1.7 |
|  | 24-JUN-2010 | 13 | .0 | .0 | 1.7 |
|  | 25-JUN-2010 | 34 | .0 | .0 | 1.7 |
|  | 26-JUN-2010 | 14 | .0 | .0 | 1.7 |
|  | 27-JUN-2010 | 21 | .0 | .0 | 1.8 |
|  | 28-JUN-2010 | 16 | .0 | .0 | 1.8 |
|  | 29-JUN-2010 | 12 | .0 | .0 | 1.8 |
|  | 30-JUN-2010 | 53 | .0 | .0 | 1.8 |
|  | 01-JUL-2010 | 131 | .1 | .1 | 1.9 |
|  | 02-JUL-2010 | 70 | .0 | .0 | 1.9 |
|  | 03-JUL-2010 | 51 | .0 | .0 | 1.9 |
|  | 04-JUL-2010 | 16 | .0 | .0 | 1.9 |
|  | 05-JUL-2010 | 81 | .0 | .0 | 2.0 |
|  | 06-JUL-2010 | 35 | .0 | .0 | 2.0 |
|  | 07-JUL-2010 | 38 | .0 | .0 | 2.0 |
|  | 08-JUL-2010 | 50 | .0 | .0 | 2.0 |
|  | 09-JUL-2010 | 89 | .0 | .0 | 2.1 |
|  | 10-JUL-2010 | 246 | .1 | .1 | 2.2 |
|  | 11-JUL-2010 | 11 | .0 | .0 | 2.2 |
|  | 12-JUL-2010 | 130 | .1 | .1 | 2.3 |
|  | 13-JUL-2010 | 28 | .0 | .0 | 2.3 |
|  | 14-JUL-2010 | 29 | .0 | .0 | 2.3 |
|  | 15-JUL-2010 | 55 | .0 | .0 | 2.4 |
|  | 16-JUL-2010 | 54 | .0 | .0 | 2.4 |
|  | 17-JUL-2010 | 18 | .0 | .0 | 2.4 |
|  | 18-JUL-2010 | 42 | .0 | .0 | 2.4 |
|  | 19-JUL-2010 | 28 | .0 | .0 | 2.4 |
|  | 20-JUL-2010 | 100 | .1 | .1 | 2.5 |
|  | 21-JUL-2010 | 24 | .0 | .0 | 2.5 |
|  | 22-JUL-2010 | 43 | .0 | .0 | 2.5 |
|  | 23-JUL-2010 | 30 | .0 | .0 | 2.5 |
|  | 24-JUL-2010 | 13 | .0 | .0 | 2.5 |
|  | 25-JUL-2010 | 62 | .0 | .0 | 2.6 |
|  | 26-JUL-2010 | 46 | .0 | .0 | 2.6 |
|  | 27-JUL-2010 | 77 | .0 | .0 | 2.6 |
|  | 28-JUL-2010 | 72 | .0 | .0 | 2.7 |
|  | 29-JUL-2010 | 59 | .0 | .0 | 2.7 |
|  | 30-JUL-2010 | 50 | .0 | .0 | 2.7 |
|  | 31-JUL-2010 | 19 | .0 | .0 | 2.7 |
|  | 01-AUG-2010 | 173 | .1 | .1 | 2.8 |
|  | 02-AUG-2010 | 208 | .1 | .1 | 2.9 |
|  | 03-AUG-2010 | 163 | .1 | .1 | 3.0 |
|  | 04-AUG-2010 | 94 | .0 | .0 | 3.1 |
|  | 05-AUG-2010 | 106 | .1 | .1 | 3.1 |
|  | 06-AUG-2010 | 98 | .1 | .1 | 3.2 |
|  | 07-AUG-2010 | 107 | .1 | .1 | 3.2 |
|  | 08-AUG-2010 | 38 | .0 | .0 | 3.3 |
|  | 09-AUG-2010 | 184 | .1 | .1 | 3.4 |
|  | 10-AUG-2010 | 277 | .1 | .1 | 3.5 |
|  | 11-AUG-2010 | 92 | .0 | .0 | 3.5 |
|  | 12-AUG-2010 | 189 | .1 | .1 | 3.6 |
|  | 13-AUG-2010 | 75 | .0 | .0 | 3.7 |
|  | 14-AUG-2010 | 62 | .0 | .0 | 3.7 |
|  | 15-AUG-2010 | 187 | .1 | .1 | 3.8 |
|  | 16-AUG-2010 | 38 | .0 | .0 | 3.8 |
|  | 17-AUG-2010 | 103 | .1 | .1 | 3.9 |
|  | 18-AUG-2010 | 133 | .1 | .1 | 4.0 |
|  | 19-AUG-2010 | 60 | .0 | .0 | 4.0 |
|  | 20-AUG-2010 | 200 | .1 | .1 | 4.1 |
|  | 21-AUG-2010 | 117 | .1 | .1 | 4.2 |
|  | 22-AUG-2010 | 108 | .1 | .1 | 4.2 |
|  | 23-AUG-2010 | 26 | .0 | .0 | 4.2 |
|  | 24-AUG-2010 | 58 | .0 | .0 | 4.3 |
|  | 25-AUG-2010 | 63 | .0 | .0 | 4.3 |
|  | 26-AUG-2010 | 97 | .1 | .1 | 4.3 |
|  | 27-AUG-2010 | 54 | .0 | .0 | 4.4 |
|  | 28-AUG-2010 | 72 | .0 | .0 | 4.4 |
|  | 29-AUG-2010 | 54 | .0 | .0 | 4.4 |
|  | 30-AUG-2010 | 74 | .0 | .0 | 4.5 |
|  | 31-AUG-2010 | 30 | .0 | .0 | 4.5 |
|  | 01-SEP-2010 | 393 | .2 | .2 | 4.7 |
|  | 02-SEP-2010 | 294 | .2 | .2 | 4.9 |
|  | 03-SEP-2010 | 144 | .1 | .1 | 4.9 |
|  | 04-SEP-2010 | 92 | .0 | .0 | 5.0 |
|  | 05-SEP-2010 | 136 | .1 | .1 | 5.1 |
|  | 06-SEP-2010 | 76 | .0 | .0 | 5.1 |
|  | 07-SEP-2010 | 175 | .1 | .1 | 5.2 |
|  | 08-SEP-2010 | 92 | .0 | .0 | 5.2 |
|  | 09-SEP-2010 | 118 | .1 | .1 | 5.3 |
|  | 10-SEP-2010 | 193 | .1 | .1 | 5.4 |
|  | 11-SEP-2010 | 77 | .0 | .0 | 5.4 |
|  | 12-SEP-2010 | 95 | .0 | .0 | 5.5 |
|  | 13-SEP-2010 | 42 | .0 | .0 | 5.5 |
|  | 14-SEP-2010 | 74 | .0 | .0 | 5.5 |
|  | 15-SEP-2010 | 100 | .1 | .1 | 5.6 |
|  | 16-SEP-2010 | 26 | .0 | .0 | 5.6 |
|  | 17-SEP-2010 | 47 | .0 | .0 | 5.6 |
|  | 18-SEP-2010 | 36 | .0 | .0 | 5.7 |
|  | 19-SEP-2010 | 28 | .0 | .0 | 5.7 |
|  | 20-SEP-2010 | 66 | .0 | .0 | 5.7 |
|  | 21-SEP-2010 | 26 | .0 | .0 | 5.7 |
|  | 22-SEP-2010 | 47 | .0 | .0 | 5.7 |
|  | 23-SEP-2010 | 31 | .0 | .0 | 5.8 |
|  | 24-SEP-2010 | 23 | .0 | .0 | 5.8 |
|  | 25-SEP-2010 | 63 | .0 | .0 | 5.8 |
|  | 26-SEP-2010 | 30 | .0 | .0 | 5.8 |
|  | 27-SEP-2010 | 43 | .0 | .0 | 5.8 |
|  | 28-SEP-2010 | 29 | .0 | .0 | 5.9 |
|  | 29-SEP-2010 | 21 | .0 | .0 | 5.9 |
|  | 30-SEP-2010 | 37 | .0 | .0 | 5.9 |
|  | 01-OCT-2010 | 42 | .0 | .0 | 5.9 |
|  | 02-OCT-2010 | 57 | .0 | .0 | 5.9 |
|  | 03-OCT-2010 | 21 | .0 | .0 | 6.0 |
|  | 04-OCT-2010 | 63 | .0 | .0 | 6.0 |
|  | 05-OCT-2010 | 33 | .0 | .0 | 6.0 |
|  | 06-OCT-2010 | 25 | .0 | .0 | 6.0 |
|  | 07-OCT-2010 | 52 | .0 | .0 | 6.0 |
|  | 08-OCT-2010 | 21 | .0 | .0 | 6.1 |
|  | 09-OCT-2010 | 39 | .0 | .0 | 6.1 |
|  | 10-OCT-2010 | 87 | .0 | .0 | 6.1 |
|  | 11-OCT-2010 | 12 | .0 | .0 | 6.1 |
|  | 12-OCT-2010 | 75 | .0 | .0 | 6.2 |
|  | 13-OCT-2010 | 2 | .0 | .0 | 6.2 |
|  | 14-OCT-2010 | 5 | .0 | .0 | 6.2 |
|  | 15-OCT-2010 | 34 | .0 | .0 | 6.2 |
|  | 16-OCT-2010 | 9 | .0 | .0 | 6.2 |
|  | 17-OCT-2010 | 18 | .0 | .0 | 6.2 |
|  | 18-OCT-2010 | 19 | .0 | .0 | 6.2 |
|  | 19-OCT-2010 | 19 | .0 | .0 | 6.2 |
|  | 20-OCT-2010 | 44 | .0 | .0 | 6.2 |
|  | 21-OCT-2010 | 7 | .0 | .0 | 6.2 |
|  | 22-OCT-2010 | 17 | .0 | .0 | 6.3 |
|  | 23-OCT-2010 | 23 | .0 | .0 | 6.3 |
|  | 24-OCT-2010 | 11 | .0 | .0 | 6.3 |
|  | 25-OCT-2010 | 36 | .0 | .0 | 6.3 |
|  | 26-OCT-2010 | 22 | .0 | .0 | 6.3 |
|  | 27-OCT-2010 | 26 | .0 | .0 | 6.3 |
|  | 28-OCT-2010 | 27 | .0 | .0 | 6.3 |
|  | 29-OCT-2010 | 12 | .0 | .0 | 6.3 |
|  | 30-OCT-2010 | 14 | .0 | .0 | 6.3 |
|  | 31-OCT-2010 | 11 | .0 | .0 | 6.4 |
|  | 01-NOV-2010 | 23 | .0 | .0 | 6.4 |
|  | 02-NOV-2010 | 29 | .0 | .0 | 6.4 |
|  | 03-NOV-2010 | 40 | .0 | .0 | 6.4 |
|  | 04-NOV-2010 | 4 | .0 | .0 | 6.4 |
|  | 05-NOV-2010 | 19 | .0 | .0 | 6.4 |
|  | 06-NOV-2010 | 46 | .0 | .0 | 6.4 |
|  | 07-NOV-2010 | 39 | .0 | .0 | 6.5 |
|  | 08-NOV-2010 | 19 | .0 | .0 | 6.5 |
|  | 09-NOV-2010 | 16 | .0 | .0 | 6.5 |
|  | 10-NOV-2010 | 30 | .0 | .0 | 6.5 |
|  | 11-NOV-2010 | 9 | .0 | .0 | 6.5 |
|  | 12-NOV-2010 | 18 | .0 | .0 | 6.5 |
|  | 13-NOV-2010 | 20 | .0 | .0 | 6.5 |
|  | 14-NOV-2010 | 5 | .0 | .0 | 6.5 |
|  | 15-NOV-2010 | 26 | .0 | .0 | 6.5 |
|  | 16-NOV-2010 | 9 | .0 | .0 | 6.5 |
|  | 17-NOV-2010 | 21 | .0 | .0 | 6.6 |
|  | 18-NOV-2010 | 10 | .0 | .0 | 6.6 |
|  | 19-NOV-2010 | 25 | .0 | .0 | 6.6 |
|  | 20-NOV-2010 | 22 | .0 | .0 | 6.6 |
|  | 21-NOV-2010 | 19 | .0 | .0 | 6.6 |
|  | 22-NOV-2010 | 1 | .0 | .0 | 6.6 |
|  | 23-NOV-2010 | 23 | .0 | .0 | 6.6 |
|  | 24-NOV-2010 | 17 | .0 | .0 | 6.6 |
|  | 25-NOV-2010 | 21 | .0 | .0 | 6.6 |
|  | 26-NOV-2010 | 15 | .0 | .0 | 6.6 |
|  | 27-NOV-2010 | 8 | .0 | .0 | 6.6 |
|  | 28-NOV-2010 | 22 | .0 | .0 | 6.6 |
|  | 29-NOV-2010 | 19 | .0 | .0 | 6.7 |
|  | 30-NOV-2010 | 16 | .0 | .0 | 6.7 |
|  | 01-DEC-2010 | 54 | .0 | .0 | 6.7 |
|  | 02-DEC-2010 | 21 | .0 | .0 | 6.7 |
|  | 03-DEC-2010 | 9 | .0 | .0 | 6.7 |
|  | 04-DEC-2010 | 7 | .0 | .0 | 6.7 |
|  | 05-DEC-2010 | 32 | .0 | .0 | 6.7 |
|  | 06-DEC-2010 | 55 | .0 | .0 | 6.8 |
|  | 07-DEC-2010 | 3 | .0 | .0 | 6.8 |
|  | 08-DEC-2010 | 16 | .0 | .0 | 6.8 |
|  | 09-DEC-2010 | 15 | .0 | .0 | 6.8 |
|  | 10-DEC-2010 | 51 | .0 | .0 | 6.8 |
|  | 11-DEC-2010 | 17 | .0 | .0 | 6.8 |
|  | 12-DEC-2010 | 40 | .0 | .0 | 6.8 |
|  | 13-DEC-2010 | 9 | .0 | .0 | 6.8 |
|  | 14-DEC-2010 | 3 | .0 | .0 | 6.8 |
|  | 15-DEC-2010 | 28 | .0 | .0 | 6.9 |
|  | 16-DEC-2010 | 52 | .0 | .0 | 6.9 |
|  | 17-DEC-2010 | 4 | .0 | .0 | 6.9 |
|  | 19-DEC-2010 | 10 | .0 | .0 | 6.9 |
|  | 20-DEC-2010 | 27 | .0 | .0 | 6.9 |
|  | 21-DEC-2010 | 25 | .0 | .0 | 6.9 |
|  | 22-DEC-2010 | 21 | .0 | .0 | 6.9 |
|  | 24-DEC-2010 | 6 | .0 | .0 | 6.9 |
|  | 25-DEC-2010 | 33 | .0 | .0 | 6.9 |
|  | 26-DEC-2010 | 38 | .0 | .0 | 7.0 |
|  | 27-DEC-2010 | 24 | .0 | .0 | 7.0 |
|  | 28-DEC-2010 | 23 | .0 | .0 | 7.0 |
|  | 29-DEC-2010 | 23 | .0 | .0 | 7.0 |
|  | 30-DEC-2010 | 25 | .0 | .0 | 7.0 |
|  | 31-DEC-2010 | 14 | .0 | .0 | 7.0 |
|  | 01-JAN-2011 | 337 | .2 | .2 | 7.2 |
|  | 02-JAN-2011 | 156 | .1 | .1 | 7.3 |
|  | 03-JAN-2011 | 104 | .1 | .1 | 7.3 |
|  | 04-JAN-2011 | 25 | .0 | .0 | 7.4 |
|  | 05-JAN-2011 | 98 | .1 | .1 | 7.4 |
|  | 06-JAN-2011 | 88 | .0 | .0 | 7.4 |
|  | 07-JAN-2011 | 89 | .0 | .0 | 7.5 |
|  | 08-JAN-2011 | 72 | .0 | .0 | 7.5 |
|  | 09-JAN-2011 | 51 | .0 | .0 | 7.6 |
|  | 10-JAN-2011 | 171 | .1 | .1 | 7.7 |
|  | 11-JAN-2011 | 48 | .0 | .0 | 7.7 |
|  | 12-JAN-2011 | 71 | .0 | .0 | 7.7 |
|  | 13-JAN-2011 | 9 | .0 | .0 | 7.7 |
|  | 14-JAN-2011 | 17 | .0 | .0 | 7.7 |
|  | 15-JAN-2011 | 59 | .0 | .0 | 7.8 |
|  | 16-JAN-2011 | 28 | .0 | .0 | 7.8 |
|  | 17-JAN-2011 | 32 | .0 | .0 | 7.8 |
|  | 18-JAN-2011 | 66 | .0 | .0 | 7.8 |
|  | 19-JAN-2011 | 19 | .0 | .0 | 7.8 |
|  | 20-JAN-2011 | 56 | .0 | .0 | 7.9 |
|  | 21-JAN-2011 | 38 | .0 | .0 | 7.9 |
|  | 22-JAN-2011 | 40 | .0 | .0 | 7.9 |
|  | 23-JAN-2011 | 38 | .0 | .0 | 7.9 |
|  | 24-JAN-2011 | 16 | .0 | .0 | 7.9 |
|  | 25-JAN-2011 | 75 | .0 | .0 | 8.0 |
|  | 26-JAN-2011 | 13 | .0 | .0 | 8.0 |
|  | 27-JAN-2011 | 31 | .0 | .0 | 8.0 |
|  | 28-JAN-2011 | 30 | .0 | .0 | 8.0 |
|  | 29-JAN-2011 | 15 | .0 | .0 | 8.0 |
|  | 30-JAN-2011 | 49 | .0 | .0 | 8.0 |
|  | 31-JAN-2011 | 39 | .0 | .0 | 8.1 |
|  | 01-FEB-2011 | 276 | .1 | .1 | 8.2 |
|  | 02-FEB-2011 | 76 | .0 | .0 | 8.3 |
|  | 03-FEB-2011 | 96 | .1 | .1 | 8.3 |
|  | 04-FEB-2011 | 93 | .0 | .0 | 8.3 |
|  | 05-FEB-2011 | 88 | .0 | .0 | 8.4 |
|  | 06-FEB-2011 | 46 | .0 | .0 | 8.4 |
|  | 07-FEB-2011 | 54 | .0 | .0 | 8.4 |
|  | 08-FEB-2011 | 59 | .0 | .0 | 8.5 |
|  | 09-FEB-2011 | 121 | .1 | .1 | 8.5 |
|  | 10-FEB-2011 | 208 | .1 | .1 | 8.7 |
|  | 11-FEB-2011 | 48 | .0 | .0 | 8.7 |
|  | 12-FEB-2011 | 116 | .1 | .1 | 8.7 |
|  | 13-FEB-2011 | 51 | .0 | .0 | 8.8 |
|  | 14-FEB-2011 | 34 | .0 | .0 | 8.8 |
|  | 15-FEB-2011 | 124 | .1 | .1 | 8.8 |
|  | 16-FEB-2011 | 33 | .0 | .0 | 8.9 |
|  | 17-FEB-2011 | 29 | .0 | .0 | 8.9 |
|  | 18-FEB-2011 | 47 | .0 | .0 | 8.9 |
|  | 19-FEB-2011 | 54 | .0 | .0 | 8.9 |
|  | 20-FEB-2011 | 100 | .1 | .1 | 9.0 |
|  | 21-FEB-2011 | 25 | .0 | .0 | 9.0 |
|  | 22-FEB-2011 | 44 | .0 | .0 | 9.0 |
|  | 23-FEB-2011 | 26 | .0 | .0 | 9.0 |
|  | 24-FEB-2011 | 63 | .0 | .0 | 9.1 |
|  | 25-FEB-2011 | 54 | .0 | .0 | 9.1 |
|  | 26-FEB-2011 | 35 | .0 | .0 | 9.1 |
|  | 27-FEB-2011 | 77 | .0 | .0 | 9.2 |
|  | 28-FEB-2011 | 96 | .1 | .1 | 9.2 |
|  | 01-MAR-2011 | 257 | .1 | .1 | 9.3 |
|  | 02-MAR-2011 | 173 | .1 | .1 | 9.4 |
|  | 03-MAR-2011 | 147 | .1 | .1 | 9.5 |
|  | 04-MAR-2011 | 107 | .1 | .1 | 9.6 |
|  | 05-MAR-2011 | 194 | .1 | .1 | 9.7 |
|  | 06-MAR-2011 | 107 | .1 | .1 | 9.7 |
|  | 07-MAR-2011 | 138 | .1 | .1 | 9.8 |
|  | 08-MAR-2011 | 61 | .0 | .0 | 9.8 |
|  | 09-MAR-2011 | 172 | .1 | .1 | 9.9 |
|  | 10-MAR-2011 | 181 | .1 | .1 | 10.0 |
|  | 11-MAR-2011 | 129 | .1 | .1 | 10.1 |
|  | 12-MAR-2011 | 96 | .1 | .1 | 10.1 |
|  | 13-MAR-2011 | 65 | .0 | .0 | 10.2 |
|  | 14-MAR-2011 | 76 | .0 | .0 | 10.2 |
|  | 15-MAR-2011 | 73 | .0 | .0 | 10.2 |
|  | 16-MAR-2011 | 46 | .0 | .0 | 10.3 |
|  | 17-MAR-2011 | 27 | .0 | .0 | 10.3 |
|  | 18-MAR-2011 | 60 | .0 | .0 | 10.3 |
|  | 19-MAR-2011 | 45 | .0 | .0 | 10.3 |
|  | 20-MAR-2011 | 30 | .0 | .0 | 10.4 |
|  | 21-MAR-2011 | 17 | .0 | .0 | 10.4 |
|  | 22-MAR-2011 | 30 | .0 | .0 | 10.4 |
|  | 23-MAR-2011 | 62 | .0 | .0 | 10.4 |
|  | 24-MAR-2011 | 44 | .0 | .0 | 10.4 |
|  | 25-MAR-2011 | 63 | .0 | .0 | 10.5 |
|  | 26-MAR-2011 | 28 | .0 | .0 | 10.5 |
|  | 27-MAR-2011 | 33 | .0 | .0 | 10.5 |
|  | 28-MAR-2011 | 11 | .0 | .0 | 10.5 |
|  | 29-MAR-2011 | 39 | .0 | .0 | 10.5 |
|  | 30-MAR-2011 | 18 | .0 | .0 | 10.5 |
|  | 31-MAR-2011 | 26 | .0 | .0 | 10.5 |
|  | 01-APR-2011 | 155 | .1 | .1 | 10.6 |
|  | 02-APR-2011 | 85 | .0 | .0 | 10.7 |
|  | 03-APR-2011 | 145 | .1 | .1 | 10.8 |
|  | 04-APR-2011 | 81 | .0 | .0 | 10.8 |
|  | 05-APR-2011 | 100 | .1 | .1 | 10.8 |
|  | 06-APR-2011 | 62 | .0 | .0 | 10.9 |
|  | 07-APR-2011 | 52 | .0 | .0 | 10.9 |
|  | 08-APR-2011 | 79 | .0 | .0 | 10.9 |
|  | 09-APR-2011 | 61 | .0 | .0 | 11.0 |
|  | 10-APR-2011 | 92 | .0 | .0 | 11.0 |
|  | 11-APR-2011 | 70 | .0 | .0 | 11.1 |
|  | 12-APR-2011 | 75 | .0 | .0 | 11.1 |
|  | 13-APR-2011 | 42 | .0 | .0 | 11.1 |
|  | 14-APR-2011 | 35 | .0 | .0 | 11.1 |
|  | 15-APR-2011 | 68 | .0 | .0 | 11.2 |
|  | 16-APR-2011 | 63 | .0 | .0 | 11.2 |
|  | 17-APR-2011 | 29 | .0 | .0 | 11.2 |
|  | 18-APR-2011 | 50 | .0 | .0 | 11.3 |
|  | 19-APR-2011 | 55 | .0 | .0 | 11.3 |
|  | 20-APR-2011 | 82 | .0 | .0 | 11.3 |
|  | 21-APR-2011 | 26 | .0 | .0 | 11.3 |
|  | 22-APR-2011 | 46 | .0 | .0 | 11.4 |
|  | 23-APR-2011 | 38 | .0 | .0 | 11.4 |
|  | 24-APR-2011 | 64 | .0 | .0 | 11.4 |
|  | 25-APR-2011 | 31 | .0 | .0 | 11.4 |
|  | 26-APR-2011 | 16 | .0 | .0 | 11.4 |
|  | 27-APR-2011 | 26 | .0 | .0 | 11.5 |
|  | 29-APR-2011 | 16 | .0 | .0 | 11.5 |
|  | 30-APR-2011 | 25 | .0 | .0 | 11.5 |
|  | 01-MAY-2011 | 156 | .1 | .1 | 11.6 |
|  | 02-MAY-2011 | 75 | .0 | .0 | 11.6 |
|  | 03-MAY-2011 | 93 | .0 | .0 | 11.6 |
|  | 04-MAY-2011 | 72 | .0 | .0 | 11.7 |
|  | 05-MAY-2011 | 69 | .0 | .0 | 11.7 |
|  | 06-MAY-2011 | 71 | .0 | .0 | 11.8 |
|  | 07-MAY-2011 | 39 | .0 | .0 | 11.8 |
|  | 08-MAY-2011 | 71 | .0 | .0 | 11.8 |
|  | 09-MAY-2011 | 45 | .0 | .0 | 11.8 |
|  | 10-MAY-2011 | 155 | .1 | .1 | 11.9 |
|  | 11-MAY-2011 | 51 | .0 | .0 | 12.0 |
|  | 12-MAY-2011 | 41 | .0 | .0 | 12.0 |
|  | 13-MAY-2011 | 41 | .0 | .0 | 12.0 |
|  | 14-MAY-2011 | 61 | .0 | .0 | 12.0 |
|  | 15-MAY-2011 | 90 | .0 | .0 | 12.1 |
|  | 16-MAY-2011 | 34 | .0 | .0 | 12.1 |
|  | 17-MAY-2011 | 32 | .0 | .0 | 12.1 |
|  | 18-MAY-2011 | 26 | .0 | .0 | 12.1 |
|  | 19-MAY-2011 | 67 | .0 | .0 | 12.2 |
|  | 20-MAY-2011 | 167 | .1 | .1 | 12.2 |
|  | 21-MAY-2011 | 21 | .0 | .0 | 12.3 |
|  | 22-MAY-2011 | 30 | .0 | .0 | 12.3 |
|  | 23-MAY-2011 | 18 | .0 | .0 | 12.3 |
|  | 24-MAY-2011 | 68 | .0 | .0 | 12.3 |
|  | 25-MAY-2011 | 102 | .1 | .1 | 12.4 |
|  | 26-MAY-2011 | 25 | .0 | .0 | 12.4 |
|  | 27-MAY-2011 | 37 | .0 | .0 | 12.4 |
|  | 28-MAY-2011 | 43 | .0 | .0 | 12.4 |
|  | 29-MAY-2011 | 44 | .0 | .0 | 12.4 |
|  | 30-MAY-2011 | 61 | .0 | .0 | 12.5 |
|  | 31-MAY-2011 | 43 | .0 | .0 | 12.5 |
|  | 01-JUN-2011 | 380 | .2 | .2 | 12.7 |
|  | 02-JUN-2011 | 220 | .1 | .1 | 12.8 |
|  | 03-JUN-2011 | 152 | .1 | .1 | 12.9 |
|  | 04-JUN-2011 | 127 | .1 | .1 | 13.0 |
|  | 05-JUN-2011 | 194 | .1 | .1 | 13.1 |
|  | 06-JUN-2011 | 129 | .1 | .1 | 13.1 |
|  | 07-JUN-2011 | 207 | .1 | .1 | 13.2 |
|  | 08-JUN-2011 | 133 | .1 | .1 | 13.3 |
|  | 09-JUN-2011 | 141 | .1 | .1 | 13.4 |
|  | 10-JUN-2011 | 357 | .2 | .2 | 13.6 |
|  | 11-JUN-2011 | 74 | .0 | .0 | 13.6 |
|  | 12-JUN-2011 | 242 | .1 | .1 | 13.7 |
|  | 13-JUN-2011 | 116 | .1 | .1 | 13.8 |
|  | 14-JUN-2011 | 53 | .0 | .0 | 13.8 |
|  | 15-JUN-2011 | 275 | .1 | .1 | 14.0 |
|  | 16-JUN-2011 | 114 | .1 | .1 | 14.0 |
|  | 17-JUN-2011 | 104 | .1 | .1 | 14.1 |
|  | 18-JUN-2011 | 87 | .0 | .0 | 14.1 |
|  | 19-JUN-2011 | 109 | .1 | .1 | 14.2 |
|  | 20-JUN-2011 | 380 | .2 | .2 | 14.4 |
|  | 21-JUN-2011 | 91 | .0 | .0 | 14.4 |
|  | 22-JUN-2011 | 55 | .0 | .0 | 14.5 |
|  | 23-JUN-2011 | 66 | .0 | .0 | 14.5 |
|  | 24-JUN-2011 | 85 | .0 | .0 | 14.5 |
|  | 25-JUN-2011 | 129 | .1 | .1 | 14.6 |
|  | 26-JUN-2011 | 107 | .1 | .1 | 14.7 |
|  | 27-JUN-2011 | 44 | .0 | .0 | 14.7 |
|  | 28-JUN-2011 | 101 | .1 | .1 | 14.7 |
|  | 29-JUN-2011 | 95 | .0 | .0 | 14.8 |
|  | 30-JUN-2011 | 89 | .0 | .0 | 14.8 |
|  | 01-JUL-2011 | 365 | .2 | .2 | 15.0 |
|  | 02-JUL-2011 | 191 | .1 | .1 | 15.1 |
|  | 03-JUL-2011 | 198 | .1 | .1 | 15.2 |
|  | 04-JUL-2011 | 74 | .0 | .0 | 15.3 |
|  | 05-JUL-2011 | 276 | .1 | .1 | 15.4 |
|  | 06-JUL-2011 | 208 | .1 | .1 | 15.5 |
|  | 07-JUL-2011 | 143 | .1 | .1 | 15.6 |
|  | 08-JUL-2011 | 245 | .1 | .1 | 15.7 |
|  | 09-JUL-2011 | 208 | .1 | .1 | 15.8 |
|  | 10-JUL-2011 | 360 | .2 | .2 | 16.0 |
|  | 11-JUL-2011 | 110 | .1 | .1 | 16.1 |
|  | 12-JUL-2011 | 179 | .1 | .1 | 16.2 |
|  | 13-JUL-2011 | 146 | .1 | .1 | 16.3 |
|  | 14-JUL-2011 | 136 | .1 | .1 | 16.3 |
|  | 15-JUL-2011 | 348 | .2 | .2 | 16.5 |
|  | 16-JUL-2011 | 106 | .1 | .1 | 16.6 |
|  | 17-JUL-2011 | 101 | .1 | .1 | 16.6 |
|  | 18-JUL-2011 | 107 | .1 | .1 | 16.7 |
|  | 19-JUL-2011 | 184 | .1 | .1 | 16.8 |
|  | 20-JUL-2011 | 357 | .2 | .2 | 17.0 |
|  | 21-JUL-2011 | 150 | .1 | .1 | 17.0 |
|  | 22-JUL-2011 | 103 | .1 | .1 | 17.1 |
|  | 23-JUL-2011 | 82 | .0 | .0 | 17.1 |
|  | 24-JUL-2011 | 106 | .1 | .1 | 17.2 |
|  | 25-JUL-2011 | 129 | .1 | .1 | 17.3 |
|  | 26-JUL-2011 | 83 | .0 | .0 | 17.3 |
|  | 27-JUL-2011 | 131 | .1 | .1 | 17.4 |
|  | 28-JUL-2011 | 221 | .1 | .1 | 17.5 |
|  | 29-JUL-2011 | 243 | .1 | .1 | 17.6 |
|  | 30-JUL-2011 | 236 | .1 | .1 | 17.7 |
|  | 31-JUL-2011 | 38 | .0 | .0 | 17.8 |
|  | 01-AUG-2011 | 667 | .4 | .4 | 18.1 |
|  | 02-AUG-2011 | 421 | .2 | .2 | 18.3 |
|  | 03-AUG-2011 | 412 | .2 | .2 | 18.6 |
|  | 04-AUG-2011 | 244 | .1 | .1 | 18.7 |
|  | 05-AUG-2011 | 377 | .2 | .2 | 18.9 |
|  | 06-AUG-2011 | 251 | .1 | .1 | 19.0 |
|  | 07-AUG-2011 | 359 | .2 | .2 | 19.2 |
|  | 08-AUG-2011 | 198 | .1 | .1 | 19.3 |
|  | 09-AUG-2011 | 398 | .2 | .2 | 19.5 |
|  | 10-AUG-2011 | 809 | .4 | .4 | 19.9 |
|  | 11-AUG-2011 | 184 | .1 | .1 | 20.0 |
|  | 12-AUG-2011 | 394 | .2 | .2 | 20.2 |
|  | 13-AUG-2011 | 228 | .1 | .1 | 20.4 |
|  | 14-AUG-2011 | 276 | .1 | .1 | 20.5 |
|  | 15-AUG-2011 | 385 | .2 | .2 | 20.7 |
|  | 16-AUG-2011 | 181 | .1 | .1 | 20.8 |
|  | 17-AUG-2011 | 243 | .1 | .1 | 20.9 |
|  | 18-AUG-2011 | 192 | .1 | .1 | 21.0 |
|  | 19-AUG-2011 | 207 | .1 | .1 | 21.1 |
|  | 20-AUG-2011 | 687 | .4 | .4 | 21.5 |
|  | 21-AUG-2011 | 119 | .1 | .1 | 21.6 |
|  | 22-AUG-2011 | 259 | .1 | .1 | 21.7 |
|  | 23-AUG-2011 | 78 | .0 | .0 | 21.7 |
|  | 24-AUG-2011 | 237 | .1 | .1 | 21.9 |
|  | 25-AUG-2011 | 300 | .2 | .2 | 22.0 |
|  | 26-AUG-2011 | 204 | .1 | .1 | 22.1 |
|  | 27-AUG-2011 | 157 | .1 | .1 | 22.2 |
|  | 28-AUG-2011 | 157 | .1 | .1 | 22.3 |
|  | 29-AUG-2011 | 206 | .1 | .1 | 22.4 |
|  | 30-AUG-2011 | 163 | .1 | .1 | 22.5 |
|  | 31-AUG-2011 | 112 | .1 | .1 | 22.6 |
|  | 01-SEP-2011 | 960 | .5 | .5 | 23.1 |
|  | 02-SEP-2011 | 664 | .3 | .3 | 23.4 |
|  | 03-SEP-2011 | 449 | .2 | .2 | 23.6 |
|  | 04-SEP-2011 | 234 | .1 | .1 | 23.8 |
|  | 05-SEP-2011 | 517 | .3 | .3 | 24.0 |
|  | 06-SEP-2011 | 295 | .2 | .2 | 24.2 |
|  | 07-SEP-2011 | 469 | .2 | .2 | 24.4 |
|  | 08-SEP-2011 | 336 | .2 | .2 | 24.6 |
|  | 09-SEP-2011 | 279 | .1 | .1 | 24.8 |
|  | 10-SEP-2011 | 701 | .4 | .4 | 25.1 |
|  | 11-SEP-2011 | 242 | .1 | .1 | 25.3 |
|  | 12-SEP-2011 | 477 | .3 | .3 | 25.5 |
|  | 13-SEP-2011 | 120 | .1 | .1 | 25.6 |
|  | 14-SEP-2011 | 297 | .2 | .2 | 25.7 |
|  | 15-SEP-2011 | 330 | .2 | .2 | 25.9 |
|  | 16-SEP-2011 | 154 | .1 | .1 | 26.0 |
|  | 17-SEP-2011 | 121 | .1 | .1 | 26.0 |
|  | 18-SEP-2011 | 165 | .1 | .1 | 26.1 |
|  | 19-SEP-2011 | 134 | .1 | .1 | 26.2 |
|  | 20-SEP-2011 | 216 | .1 | .1 | 26.3 |
|  | 21-SEP-2011 | 127 | .1 | .1 | 26.4 |
|  | 22-SEP-2011 | 128 | .1 | .1 | 26.5 |
|  | 23-SEP-2011 | 104 | .1 | .1 | 26.5 |
|  | 24-SEP-2011 | 81 | .0 | .0 | 26.5 |
|  | 25-SEP-2011 | 124 | .1 | .1 | 26.6 |
|  | 26-SEP-2011 | 94 | .0 | .0 | 26.7 |
|  | 27-SEP-2011 | 92 | .0 | .0 | 26.7 |
|  | 28-SEP-2011 | 121 | .1 | .1 | 26.8 |
|  | 29-SEP-2011 | 73 | .0 | .0 | 26.8 |
|  | 30-SEP-2011 | 126 | .1 | .1 | 26.9 |
|  | 01-OCT-2011 | 181 | .1 | .1 | 27.0 |
|  | 02-OCT-2011 | 154 | .1 | .1 | 27.1 |
|  | 03-OCT-2011 | 105 | .1 | .1 | 27.1 |
|  | 04-OCT-2011 | 95 | .0 | .0 | 27.2 |
|  | 05-OCT-2011 | 178 | .1 | .1 | 27.3 |
|  | 06-OCT-2011 | 156 | .1 | .1 | 27.3 |
|  | 07-OCT-2011 | 162 | .1 | .1 | 27.4 |
|  | 08-OCT-2011 | 136 | .1 | .1 | 27.5 |
|  | 09-OCT-2011 | 72 | .0 | .0 | 27.5 |
|  | 10-OCT-2011 | 181 | .1 | .1 | 27.6 |
|  | 11-OCT-2011 | 49 | .0 | .0 | 27.7 |
|  | 12-OCT-2011 | 192 | .1 | .1 | 27.8 |
|  | 13-OCT-2011 | 103 | .1 | .1 | 27.8 |
|  | 14-OCT-2011 | 103 | .1 | .1 | 27.9 |
|  | 15-OCT-2011 | 106 | .1 | .1 | 27.9 |
|  | 16-OCT-2011 | 69 | .0 | .0 | 28.0 |
|  | 17-OCT-2011 | 72 | .0 | .0 | 28.0 |
|  | 18-OCT-2011 | 125 | .1 | .1 | 28.1 |
|  | 19-OCT-2011 | 100 | .1 | .1 | 28.1 |
|  | 20-OCT-2011 | 270 | .1 | .1 | 28.2 |
|  | 21-OCT-2011 | 92 | .0 | .0 | 28.3 |
|  | 22-OCT-2011 | 118 | .1 | .1 | 28.4 |
|  | 23-OCT-2011 | 61 | .0 | .0 | 28.4 |
|  | 24-OCT-2011 | 71 | .0 | .0 | 28.4 |
|  | 25-OCT-2011 | 71 | .0 | .0 | 28.5 |
|  | 26-OCT-2011 | 76 | .0 | .0 | 28.5 |
|  | 27-OCT-2011 | 73 | .0 | .0 | 28.5 |
|  | 28-OCT-2011 | 89 | .0 | .0 | 28.6 |
|  | 29-OCT-2011 | 71 | .0 | .0 | 28.6 |
|  | 30-OCT-2011 | 64 | .0 | .0 | 28.7 |
|  | 31-OCT-2011 | 59 | .0 | .0 | 28.7 |
|  | 01-NOV-2011 | 167 | .1 | .1 | 28.8 |
|  | 02-NOV-2011 | 168 | .1 | .1 | 28.9 |
|  | 03-NOV-2011 | 196 | .1 | .1 | 29.0 |
|  | 04-NOV-2011 | 113 | .1 | .1 | 29.0 |
|  | 05-NOV-2011 | 147 | .1 | .1 | 29.1 |
|  | 06-NOV-2011 | 47 | .0 | .0 | 29.1 |
|  | 07-NOV-2011 | 119 | .1 | .1 | 29.2 |
|  | 08-NOV-2011 | 92 | .0 | .0 | 29.2 |
|  | 09-NOV-2011 | 63 | .0 | .0 | 29.3 |
|  | 10-NOV-2011 | 167 | .1 | .1 | 29.4 |
|  | 11-NOV-2011 | 161 | .1 | .1 | 29.5 |
|  | 12-NOV-2011 | 45 | .0 | .0 | 29.5 |
|  | 13-NOV-2011 | 64 | .0 | .0 | 29.5 |
|  | 14-NOV-2011 | 108 | .1 | .1 | 29.6 |
|  | 15-NOV-2011 | 108 | .1 | .1 | 29.6 |
|  | 16-NOV-2011 | 58 | .0 | .0 | 29.7 |
|  | 17-NOV-2011 | 46 | .0 | .0 | 29.7 |
|  | 18-NOV-2011 | 70 | .0 | .0 | 29.7 |
|  | 19-NOV-2011 | 41 | .0 | .0 | 29.7 |
|  | 20-NOV-2011 | 196 | .1 | .1 | 29.8 |
|  | 21-NOV-2011 | 66 | .0 | .0 | 29.9 |
|  | 22-NOV-2011 | 64 | .0 | .0 | 29.9 |
|  | 23-NOV-2011 | 55 | .0 | .0 | 29.9 |
|  | 24-NOV-2011 | 39 | .0 | .0 | 30.0 |
|  | 25-NOV-2011 | 93 | .0 | .0 | 30.0 |
|  | 26-NOV-2011 | 93 | .0 | .0 | 30.1 |
|  | 27-NOV-2011 | 77 | .0 | .0 | 30.1 |
|  | 28-NOV-2011 | 135 | .1 | .1 | 30.2 |
|  | 29-NOV-2011 | 90 | .0 | .0 | 30.2 |
|  | 30-NOV-2011 | 61 | .0 | .0 | 30.2 |
|  | 01-DEC-2011 | 268 | .1 | .1 | 30.4 |
|  | 02-DEC-2011 | 171 | .1 | .1 | 30.5 |
|  | 03-DEC-2011 | 112 | .1 | .1 | 30.5 |
|  | 04-DEC-2011 | 83 | .0 | .0 | 30.6 |
|  | 05-DEC-2011 | 117 | .1 | .1 | 30.6 |
|  | 06-DEC-2011 | 89 | .0 | .0 | 30.7 |
|  | 07-DEC-2011 | 98 | .1 | .1 | 30.7 |
|  | 08-DEC-2011 | 120 | .1 | .1 | 30.8 |
|  | 09-DEC-2011 | 90 | .0 | .0 | 30.8 |
|  | 10-DEC-2011 | 161 | .1 | .1 | 30.9 |
|  | 11-DEC-2011 | 93 | .0 | .0 | 31.0 |
|  | 12-DEC-2011 | 125 | .1 | .1 | 31.0 |
|  | 13-DEC-2011 | 123 | .1 | .1 | 31.1 |
|  | 14-DEC-2011 | 62 | .0 | .0 | 31.1 |
|  | 15-DEC-2011 | 105 | .1 | .1 | 31.2 |
|  | 16-DEC-2011 | 159 | .1 | .1 | 31.3 |
|  | 17-DEC-2011 | 113 | .1 | .1 | 31.3 |
|  | 18-DEC-2011 | 60 | .0 | .0 | 31.4 |
|  | 19-DEC-2011 | 117 | .1 | .1 | 31.4 |
|  | 20-DEC-2011 | 177 | .1 | .1 | 31.5 |
|  | 21-DEC-2011 | 68 | .0 | .0 | 31.6 |
|  | 22-DEC-2011 | 56 | .0 | .0 | 31.6 |
|  | 23-DEC-2011 | 114 | .1 | .1 | 31.7 |
|  | 24-DEC-2011 | 49 | .0 | .0 | 31.7 |
|  | 25-DEC-2011 | 85 | .0 | .0 | 31.7 |
|  | 26-DEC-2011 | 166 | .1 | .1 | 31.8 |
|  | 27-DEC-2011 | 86 | .0 | .0 | 31.9 |
|  | 28-DEC-2011 | 127 | .1 | .1 | 31.9 |
|  | 29-DEC-2011 | 116 | .1 | .1 | 32.0 |
|  | 30-DEC-2011 | 232 | .1 | .1 | 32.1 |
|  | 31-DEC-2011 | 17 | .0 | .0 | 32.1 |
|  | 01-JAN-2012 | 1205 | .6 | .6 | 32.7 |
|  | 02-JAN-2012 | 398 | .2 | .2 | 33.0 |
|  | 03-JAN-2012 | 243 | .1 | .1 | 33.1 |
|  | 04-JAN-2012 | 188 | .1 | .1 | 33.2 |
|  | 05-JAN-2012 | 374 | .2 | .2 | 33.4 |
|  | 06-JAN-2012 | 211 | .1 | .1 | 33.5 |
|  | 07-JAN-2012 | 226 | .1 | .1 | 33.6 |
|  | 08-JAN-2012 | 167 | .1 | .1 | 33.7 |
|  | 09-JAN-2012 | 324 | .2 | .2 | 33.9 |
|  | 10-JAN-2012 | 520 | .3 | .3 | 34.1 |
|  | 11-JAN-2012 | 170 | .1 | .1 | 34.2 |
|  | 12-JAN-2012 | 310 | .2 | .2 | 34.4 |
|  | 13-JAN-2012 | 72 | .0 | .0 | 34.4 |
|  | 14-JAN-2012 | 151 | .1 | .1 | 34.5 |
|  | 15-JAN-2012 | 181 | .1 | .1 | 34.6 |
|  | 16-JAN-2012 | 97 | .1 | .1 | 34.7 |
|  | 17-JAN-2012 | 123 | .1 | .1 | 34.7 |
|  | 18-JAN-2012 | 112 | .1 | .1 | 34.8 |
|  | 19-JAN-2012 | 95 | .0 | .0 | 34.8 |
|  | 20-JAN-2012 | 287 | .2 | .2 | 35.0 |
|  | 21-JAN-2012 | 102 | .1 | .1 | 35.0 |
|  | 22-JAN-2012 | 119 | .1 | .1 | 35.1 |
|  | 23-JAN-2012 | 107 | .1 | .1 | 35.2 |
|  | 24-JAN-2012 | 73 | .0 | .0 | 35.2 |
|  | 25-JAN-2012 | 150 | .1 | .1 | 35.3 |
|  | 26-JAN-2012 | 108 | .1 | .1 | 35.3 |
|  | 27-JAN-2012 | 128 | .1 | .1 | 35.4 |
|  | 28-JAN-2012 | 107 | .1 | .1 | 35.4 |
|  | 29-JAN-2012 | 98 | .1 | .1 | 35.5 |
|  | 30-JAN-2012 | 104 | .1 | .1 | 35.6 |
|  | 31-JAN-2012 | 75 | .0 | .0 | 35.6 |
|  | 01-FEB-2012 | 679 | .4 | .4 | 36.0 |
|  | 02-FEB-2012 | 298 | .2 | .2 | 36.1 |
|  | 03-FEB-2012 | 176 | .1 | .1 | 36.2 |
|  | 04-FEB-2012 | 257 | .1 | .1 | 36.3 |
|  | 05-FEB-2012 | 244 | .1 | .1 | 36.5 |
|  | 06-FEB-2012 | 219 | .1 | .1 | 36.6 |
|  | 07-FEB-2012 | 216 | .1 | .1 | 36.7 |
|  | 08-FEB-2012 | 275 | .1 | .1 | 36.8 |
|  | 09-FEB-2012 | 283 | .1 | .1 | 37.0 |
|  | 10-FEB-2012 | 496 | .3 | .3 | 37.2 |
|  | 11-FEB-2012 | 145 | .1 | .1 | 37.3 |
|  | 12-FEB-2012 | 231 | .1 | .1 | 37.4 |
|  | 13-FEB-2012 | 158 | .1 | .1 | 37.5 |
|  | 14-FEB-2012 | 179 | .1 | .1 | 37.6 |
|  | 15-FEB-2012 | 207 | .1 | .1 | 37.7 |
|  | 16-FEB-2012 | 80 | .0 | .0 | 37.8 |
|  | 17-FEB-2012 | 110 | .1 | .1 | 37.8 |
|  | 18-FEB-2012 | 114 | .1 | .1 | 37.9 |
|  | 19-FEB-2012 | 194 | .1 | .1 | 38.0 |
|  | 20-FEB-2012 | 490 | .3 | .3 | 38.2 |
|  | 21-FEB-2012 | 145 | .1 | .1 | 38.3 |
|  | 22-FEB-2012 | 165 | .1 | .1 | 38.4 |
|  | 23-FEB-2012 | 98 | .1 | .1 | 38.5 |
|  | 24-FEB-2012 | 143 | .1 | .1 | 38.5 |
|  | 25-FEB-2012 | 207 | .1 | .1 | 38.6 |
|  | 26-FEB-2012 | 102 | .1 | .1 | 38.7 |
|  | 27-FEB-2012 | 107 | .1 | .1 | 38.8 |
|  | 28-FEB-2012 | 215 | .1 | .1 | 38.9 |
|  | 29-FEB-2012 | 70 | .0 | .0 | 38.9 |
|  | 01-MAR-2012 | 954 | .5 | .5 | 39.4 |
|  | 02-MAR-2012 | 534 | .3 | .3 | 39.7 |
|  | 03-MAR-2012 | 373 | .2 | .2 | 39.9 |
|  | 04-MAR-2012 | 337 | .2 | .2 | 40.1 |
|  | 05-MAR-2012 | 268 | .1 | .1 | 40.2 |
|  | 06-MAR-2012 | 322 | .2 | .2 | 40.4 |
|  | 07-MAR-2012 | 240 | .1 | .1 | 40.5 |
|  | 08-MAR-2012 | 315 | .2 | .2 | 40.7 |
|  | 09-MAR-2012 | 258 | .1 | .1 | 40.8 |
|  | 10-MAR-2012 | 522 | .3 | .3 | 41.1 |
|  | 11-MAR-2012 | 176 | .1 | .1 | 41.2 |
|  | 12-MAR-2012 | 268 | .1 | .1 | 41.3 |
|  | 13-MAR-2012 | 131 | .1 | .1 | 41.4 |
|  | 14-MAR-2012 | 116 | .1 | .1 | 41.4 |
|  | 15-MAR-2012 | 195 | .1 | .1 | 41.5 |
|  | 16-MAR-2012 | 161 | .1 | .1 | 41.6 |
|  | 17-MAR-2012 | 94 | .0 | .0 | 41.7 |
|  | 18-MAR-2012 | 112 | .1 | .1 | 41.7 |
|  | 19-MAR-2012 | 78 | .0 | .0 | 41.8 |
|  | 20-MAR-2012 | 236 | .1 | .1 | 41.9 |
|  | 21-MAR-2012 | 57 | .0 | .0 | 41.9 |
|  | 22-MAR-2012 | 166 | .1 | .1 | 42.0 |
|  | 23-MAR-2012 | 66 | .0 | .0 | 42.0 |
|  | 24-MAR-2012 | 38 | .0 | .0 | 42.1 |
|  | 25-MAR-2012 | 123 | .1 | .1 | 42.1 |
|  | 26-MAR-2012 | 95 | .0 | .0 | 42.2 |
|  | 27-MAR-2012 | 61 | .0 | .0 | 42.2 |
|  | 28-MAR-2012 | 99 | .1 | .1 | 42.3 |
|  | 29-MAR-2012 | 91 | .0 | .0 | 42.3 |
|  | 30-MAR-2012 | 140 | .1 | .1 | 42.4 |
|  | 31-MAR-2012 | 50 | .0 | .0 | 42.4 |
|  | 01-APR-2012 | 270 | .1 | .1 | 42.6 |
|  | 02-APR-2012 | 180 | .1 | .1 | 42.7 |
|  | 03-APR-2012 | 149 | .1 | .1 | 42.7 |
|  | 04-APR-2012 | 159 | .1 | .1 | 42.8 |
|  | 05-APR-2012 | 149 | .1 | .1 | 42.9 |
|  | 06-APR-2012 | 103 | .1 | .1 | 42.9 |
|  | 07-APR-2012 | 115 | .1 | .1 | 43.0 |
|  | 08-APR-2012 | 132 | .1 | .1 | 43.1 |
|  | 09-APR-2012 | 88 | .0 | .0 | 43.1 |
|  | 10-APR-2012 | 155 | .1 | .1 | 43.2 |
|  | 11-APR-2012 | 87 | .0 | .0 | 43.2 |
|  | 12-APR-2012 | 232 | .1 | .1 | 43.4 |
|  | 13-APR-2012 | 72 | .0 | .0 | 43.4 |
|  | 14-APR-2012 | 66 | .0 | .0 | 43.4 |
|  | 15-APR-2012 | 181 | .1 | .1 | 43.5 |
|  | 16-APR-2012 | 78 | .0 | .0 | 43.6 |
|  | 17-APR-2012 | 109 | .1 | .1 | 43.6 |
|  | 18-APR-2012 | 106 | .1 | .1 | 43.7 |
|  | 19-APR-2012 | 41 | .0 | .0 | 43.7 |
|  | 20-APR-2012 | 305 | .2 | .2 | 43.9 |
|  | 21-APR-2012 | 55 | .0 | .0 | 43.9 |
|  | 22-APR-2012 | 84 | .0 | .0 | 43.9 |
|  | 23-APR-2012 | 51 | .0 | .0 | 44.0 |
|  | 24-APR-2012 | 99 | .1 | .1 | 44.0 |
|  | 25-APR-2012 | 68 | .0 | .0 | 44.1 |
|  | 26-APR-2012 | 28 | .0 | .0 | 44.1 |
|  | 27-APR-2012 | 105 | .1 | .1 | 44.1 |
|  | 28-APR-2012 | 38 | .0 | .0 | 44.2 |
|  | 29-APR-2012 | 49 | .0 | .0 | 44.2 |
|  | 30-APR-2012 | 40 | .0 | .0 | 44.2 |
|  | 01-MAY-2012 | 431 | .2 | .2 | 44.4 |
|  | 02-MAY-2012 | 202 | .1 | .1 | 44.5 |
|  | 03-MAY-2012 | 174 | .1 | .1 | 44.6 |
|  | 04-MAY-2012 | 133 | .1 | .1 | 44.7 |
|  | 05-MAY-2012 | 178 | .1 | .1 | 44.8 |
|  | 06-MAY-2012 | 116 | .1 | .1 | 44.8 |
|  | 07-MAY-2012 | 112 | .1 | .1 | 44.9 |
|  | 08-MAY-2012 | 105 | .1 | .1 | 45.0 |
|  | 09-MAY-2012 | 154 | .1 | .1 | 45.0 |
|  | 10-MAY-2012 | 278 | .1 | .1 | 45.2 |
|  | 11-MAY-2012 | 138 | .1 | .1 | 45.3 |
|  | 12-MAY-2012 | 126 | .1 | .1 | 45.3 |
|  | 13-MAY-2012 | 80 | .0 | .0 | 45.4 |
|  | 14-MAY-2012 | 87 | .0 | .0 | 45.4 |
|  | 15-MAY-2012 | 133 | .1 | .1 | 45.5 |
|  | 16-MAY-2012 | 117 | .1 | .1 | 45.5 |
|  | 17-MAY-2012 | 105 | .1 | .1 | 45.6 |
|  | 18-MAY-2012 | 98 | .1 | .1 | 45.7 |
|  | 19-MAY-2012 | 87 | .0 | .0 | 45.7 |
|  | 20-MAY-2012 | 196 | .1 | .1 | 45.8 |
|  | 21-MAY-2012 | 67 | .0 | .0 | 45.8 |
|  | 22-MAY-2012 | 113 | .1 | .1 | 45.9 |
|  | 23-MAY-2012 | 75 | .0 | .0 | 45.9 |
|  | 24-MAY-2012 | 49 | .0 | .0 | 46.0 |
|  | 25-MAY-2012 | 110 | .1 | .1 | 46.0 |
|  | 26-MAY-2012 | 65 | .0 | .0 | 46.1 |
|  | 27-MAY-2012 | 87 | .0 | .0 | 46.1 |
|  | 28-MAY-2012 | 70 | .0 | .0 | 46.1 |
|  | 29-MAY-2012 | 48 | .0 | .0 | 46.2 |
|  | 30-MAY-2012 | 93 | .0 | .0 | 46.2 |
|  | 31-MAY-2012 | 57 | .0 | .0 | 46.2 |
|  | 01-JUN-2012 | 440 | .2 | .2 | 46.5 |
|  | 02-JUN-2012 | 313 | .2 | .2 | 46.6 |
|  | 03-JUN-2012 | 187 | .1 | .1 | 46.7 |
|  | 04-JUN-2012 | 106 | .1 | .1 | 46.8 |
|  | 05-JUN-2012 | 153 | .1 | .1 | 46.9 |
|  | 06-JUN-2012 | 141 | .1 | .1 | 46.9 |
|  | 07-JUN-2012 | 130 | .1 | .1 | 47.0 |
|  | 08-JUN-2012 | 113 | .1 | .1 | 47.1 |
|  | 09-JUN-2012 | 89 | .0 | .0 | 47.1 |
|  | 10-JUN-2012 | 381 | .2 | .2 | 47.3 |
|  | 11-JUN-2012 | 118 | .1 | .1 | 47.4 |
|  | 12-JUN-2012 | 194 | .1 | .1 | 47.5 |
|  | 13-JUN-2012 | 119 | .1 | .1 | 47.5 |
|  | 14-JUN-2012 | 107 | .1 | .1 | 47.6 |
|  | 15-JUN-2012 | 278 | .1 | .1 | 47.7 |
|  | 16-JUN-2012 | 71 | .0 | .0 | 47.8 |
|  | 17-JUN-2012 | 90 | .0 | .0 | 47.8 |
|  | 18-JUN-2012 | 62 | .0 | .0 | 47.9 |
|  | 19-JUN-2012 | 68 | .0 | .0 | 47.9 |
|  | 20-JUN-2012 | 313 | .2 | .2 | 48.1 |
|  | 21-JUN-2012 | 116 | .1 | .1 | 48.1 |
|  | 22-JUN-2012 | 62 | .0 | .0 | 48.2 |
|  | 23-JUN-2012 | 94 | .0 | .0 | 48.2 |
|  | 24-JUN-2012 | 82 | .0 | .0 | 48.3 |
|  | 25-JUN-2012 | 152 | .1 | .1 | 48.3 |
|  | 26-JUN-2012 | 86 | .0 | .0 | 48.4 |
|  | 27-JUN-2012 | 71 | .0 | .0 | 48.4 |
|  | 28-JUN-2012 | 131 | .1 | .1 | 48.5 |
|  | 29-JUN-2012 | 52 | .0 | .0 | 48.5 |
|  | 30-JUN-2012 | 161 | .1 | .1 | 48.6 |
|  | 01-JUL-2012 | 415 | .2 | .2 | 48.8 |
|  | 02-JUL-2012 | 274 | .1 | .1 | 49.0 |
|  | 03-JUL-2012 | 190 | .1 | .1 | 49.1 |
|  | 04-JUL-2012 | 114 | .1 | .1 | 49.1 |
|  | 05-JUL-2012 | 235 | .1 | .1 | 49.2 |
|  | 06-JUL-2012 | 141 | .1 | .1 | 49.3 |
|  | 07-JUL-2012 | 226 | .1 | .1 | 49.4 |
|  | 08-JUL-2012 | 241 | .1 | .1 | 49.6 |
|  | 09-JUL-2012 | 220 | .1 | .1 | 49.7 |
|  | 10-JUL-2012 | 404 | .2 | .2 | 49.9 |
|  | 11-JUL-2012 | 196 | .1 | .1 | 50.0 |
|  | 12-JUL-2012 | 184 | .1 | .1 | 50.1 |
|  | 13-JUL-2012 | 171 | .1 | .1 | 50.2 |
|  | 14-JUL-2012 | 155 | .1 | .1 | 50.3 |
|  | 15-JUL-2012 | 196 | .1 | .1 | 50.4 |
|  | 16-JUL-2012 | 134 | .1 | .1 | 50.4 |
|  | 17-JUL-2012 | 71 | .0 | .0 | 50.5 |
|  | 18-JUL-2012 | 46 | .0 | .0 | 50.5 |
|  | 19-JUL-2012 | 58 | .0 | .0 | 50.5 |
|  | 20-JUL-2012 | 227 | .1 | .1 | 50.6 |
|  | 21-JUL-2012 | 86 | .0 | .0 | 50.7 |
|  | 22-JUL-2012 | 105 | .1 | .1 | 50.7 |
|  | 23-JUL-2012 | 138 | .1 | .1 | 50.8 |
|  | 24-JUL-2012 | 39 | .0 | .0 | 50.8 |
|  | 25-JUL-2012 | 192 | .1 | .1 | 50.9 |
|  | 26-JUL-2012 | 81 | .0 | .0 | 51.0 |
|  | 27-JUL-2012 | 83 | .0 | .0 | 51.0 |
|  | 28-JUL-2012 | 180 | .1 | .1 | 51.1 |
|  | 29-JUL-2012 | 102 | .1 | .1 | 51.2 |
|  | 30-JUL-2012 | 201 | .1 | .1 | 51.3 |
|  | 31-JUL-2012 | 59 | .0 | .0 | 51.3 |
|  | 01-AUG-2012 | 617 | .3 | .3 | 51.6 |
|  | 02-AUG-2012 | 351 | .2 | .2 | 51.8 |
|  | 03-AUG-2012 | 265 | .1 | .1 | 52.0 |
|  | 04-AUG-2012 | 146 | .1 | .1 | 52.0 |
|  | 05-AUG-2012 | 276 | .1 | .1 | 52.2 |
|  | 06-AUG-2012 | 170 | .1 | .1 | 52.3 |
|  | 07-AUG-2012 | 211 | .1 | .1 | 52.4 |
|  | 08-AUG-2012 | 154 | .1 | .1 | 52.5 |
|  | 09-AUG-2012 | 350 | .2 | .2 | 52.6 |
|  | 10-AUG-2012 | 597 | .3 | .3 | 53.0 |
|  | 11-AUG-2012 | 147 | .1 | .1 | 53.0 |
|  | 12-AUG-2012 | 271 | .1 | .1 | 53.2 |
|  | 13-AUG-2012 | 111 | .1 | .1 | 53.2 |
|  | 14-AUG-2012 | 159 | .1 | .1 | 53.3 |
|  | 15-AUG-2012 | 249 | .1 | .1 | 53.4 |
|  | 16-AUG-2012 | 94 | .0 | .0 | 53.5 |
|  | 17-AUG-2012 | 202 | .1 | .1 | 53.6 |
|  | 18-AUG-2012 | 216 | .1 | .1 | 53.7 |
|  | 19-AUG-2012 | 106 | .1 | .1 | 53.8 |
|  | 20-AUG-2012 | 331 | .2 | .2 | 53.9 |
|  | 21-AUG-2012 | 132 | .1 | .1 | 54.0 |
|  | 22-AUG-2012 | 178 | .1 | .1 | 54.1 |
|  | 23-AUG-2012 | 98 | .1 | .1 | 54.2 |
|  | 24-AUG-2012 | 104 | .1 | .1 | 54.2 |
|  | 25-AUG-2012 | 354 | .2 | .2 | 54.4 |
|  | 26-AUG-2012 | 145 | .1 | .1 | 54.5 |
|  | 27-AUG-2012 | 146 | .1 | .1 | 54.6 |
|  | 28-AUG-2012 | 169 | .1 | .1 | 54.6 |
|  | 29-AUG-2012 | 181 | .1 | .1 | 54.7 |
|  | 30-AUG-2012 | 151 | .1 | .1 | 54.8 |
|  | 31-AUG-2012 | 74 | .0 | .0 | 54.9 |
|  | 01-SEP-2012 | 874 | .5 | .5 | 55.3 |
|  | 02-SEP-2012 | 372 | .2 | .2 | 55.5 |
|  | 03-SEP-2012 | 223 | .1 | .1 | 55.6 |
|  | 04-SEP-2012 | 265 | .1 | .1 | 55.8 |
|  | 05-SEP-2012 | 273 | .1 | .1 | 55.9 |
|  | 06-SEP-2012 | 208 | .1 | .1 | 56.0 |
|  | 07-SEP-2012 | 278 | .1 | .1 | 56.2 |
|  | 08-SEP-2012 | 221 | .1 | .1 | 56.3 |
|  | 09-SEP-2012 | 256 | .1 | .1 | 56.4 |
|  | 10-SEP-2012 | 585 | .3 | .3 | 56.7 |
|  | 11-SEP-2012 | 181 | .1 | .1 | 56.8 |
|  | 12-SEP-2012 | 258 | .1 | .1 | 57.0 |
|  | 13-SEP-2012 | 125 | .1 | .1 | 57.0 |
|  | 14-SEP-2012 | 208 | .1 | .1 | 57.1 |
|  | 15-SEP-2012 | 245 | .1 | .1 | 57.3 |
|  | 16-SEP-2012 | 204 | .1 | .1 | 57.4 |
|  | 17-SEP-2012 | 157 | .1 | .1 | 57.5 |
|  | 18-SEP-2012 | 162 | .1 | .1 | 57.5 |
|  | 19-SEP-2012 | 135 | .1 | .1 | 57.6 |
|  | 20-SEP-2012 | 304 | .2 | .2 | 57.8 |
|  | 21-SEP-2012 | 91 | .0 | .0 | 57.8 |
|  | 22-SEP-2012 | 73 | .0 | .0 | 57.9 |
|  | 23-SEP-2012 | 142 | .1 | .1 | 57.9 |
|  | 24-SEP-2012 | 59 | .0 | .0 | 58.0 |
|  | 25-SEP-2012 | 139 | .1 | .1 | 58.0 |
|  | 26-SEP-2012 | 115 | .1 | .1 | 58.1 |
|  | 27-SEP-2012 | 84 | .0 | .0 | 58.1 |
|  | 28-SEP-2012 | 118 | .1 | .1 | 58.2 |
|  | 29-SEP-2012 | 81 | .0 | .0 | 58.2 |
|  | 30-SEP-2012 | 121 | .1 | .1 | 58.3 |
|  | 01-OCT-2012 | 389 | .2 | .2 | 58.5 |
|  | 02-OCT-2012 | 327 | .2 | .2 | 58.7 |
|  | 03-OCT-2012 | 156 | .1 | .1 | 58.8 |
|  | 04-OCT-2012 | 158 | .1 | .1 | 58.8 |
|  | 05-OCT-2012 | 269 | .1 | .1 | 59.0 |
|  | 06-OCT-2012 | 177 | .1 | .1 | 59.1 |
|  | 07-OCT-2012 | 206 | .1 | .1 | 59.2 |
|  | 08-OCT-2012 | 155 | .1 | .1 | 59.3 |
|  | 09-OCT-2012 | 212 | .1 | .1 | 59.4 |
|  | 10-OCT-2012 | 318 | .2 | .2 | 59.5 |
|  | 11-OCT-2012 | 127 | .1 | .1 | 59.6 |
|  | 12-OCT-2012 | 243 | .1 | .1 | 59.7 |
|  | 13-OCT-2012 | 81 | .0 | .0 | 59.8 |
|  | 14-OCT-2012 | 96 | .1 | .1 | 59.8 |
|  | 15-OCT-2012 | 239 | .1 | .1 | 60.0 |
|  | 16-OCT-2012 | 149 | .1 | .1 | 60.0 |
|  | 17-OCT-2012 | 173 | .1 | .1 | 60.1 |
|  | 18-OCT-2012 | 116 | .1 | .1 | 60.2 |
|  | 19-OCT-2012 | 137 | .1 | .1 | 60.3 |
|  | 20-OCT-2012 | 325 | .2 | .2 | 60.4 |
|  | 21-OCT-2012 | 130 | .1 | .1 | 60.5 |
|  | 22-OCT-2012 | 110 | .1 | .1 | 60.6 |
|  | 23-OCT-2012 | 74 | .0 | .0 | 60.6 |
|  | 24-OCT-2012 | 141 | .1 | .1 | 60.7 |
|  | 25-OCT-2012 | 157 | .1 | .1 | 60.8 |
|  | 26-OCT-2012 | 79 | .0 | .0 | 60.8 |
|  | 27-OCT-2012 | 104 | .1 | .1 | 60.9 |
|  | 28-OCT-2012 | 153 | .1 | .1 | 60.9 |
|  | 29-OCT-2012 | 193 | .1 | .1 | 61.0 |
|  | 30-OCT-2012 | 82 | .0 | .0 | 61.1 |
|  | 31-OCT-2012 | 33 | .0 | .0 | 61.1 |
|  | 01-NOV-2012 | 416 | .2 | .2 | 61.3 |
|  | 02-NOV-2012 | 303 | .2 | .2 | 61.5 |
|  | 03-NOV-2012 | 112 | .1 | .1 | 61.5 |
|  | 04-NOV-2012 | 87 | .0 | .0 | 61.6 |
|  | 05-NOV-2012 | 314 | .2 | .2 | 61.7 |
|  | 06-NOV-2012 | 183 | .1 | .1 | 61.8 |
|  | 07-NOV-2012 | 234 | .1 | .1 | 62.0 |
|  | 08-NOV-2012 | 158 | .1 | .1 | 62.0 |
|  | 09-NOV-2012 | 233 | .1 | .1 | 62.2 |
|  | 10-NOV-2012 | 418 | .2 | .2 | 62.4 |
|  | 11-NOV-2012 | 135 | .1 | .1 | 62.5 |
|  | 12-NOV-2012 | 165 | .1 | .1 | 62.5 |
|  | 13-NOV-2012 | 136 | .1 | .1 | 62.6 |
|  | 14-NOV-2012 | 111 | .1 | .1 | 62.7 |
|  | 15-NOV-2012 | 185 | .1 | .1 | 62.8 |
|  | 16-NOV-2012 | 121 | .1 | .1 | 62.8 |
|  | 17-NOV-2012 | 104 | .1 | .1 | 62.9 |
|  | 18-NOV-2012 | 129 | .1 | .1 | 63.0 |
|  | 19-NOV-2012 | 105 | .1 | .1 | 63.0 |
|  | 20-NOV-2012 | 301 | .2 | .2 | 63.2 |
|  | 21-NOV-2012 | 113 | .1 | .1 | 63.2 |
|  | 22-NOV-2012 | 156 | .1 | .1 | 63.3 |
|  | 23-NOV-2012 | 75 | .0 | .0 | 63.4 |
|  | 24-NOV-2012 | 122 | .1 | .1 | 63.4 |
|  | 25-NOV-2012 | 155 | .1 | .1 | 63.5 |
|  | 26-NOV-2012 | 112 | .1 | .1 | 63.6 |
|  | 27-NOV-2012 | 104 | .1 | .1 | 63.6 |
|  | 28-NOV-2012 | 175 | .1 | .1 | 63.7 |
|  | 29-NOV-2012 | 68 | .0 | .0 | 63.7 |
|  | 30-NOV-2012 | 141 | .1 | .1 | 63.8 |
|  | 01-DEC-2012 | 563 | .3 | .3 | 64.1 |
|  | 02-DEC-2012 | 268 | .1 | .1 | 64.2 |
|  | 03-DEC-2012 | 159 | .1 | .1 | 64.3 |
|  | 04-DEC-2012 | 109 | .1 | .1 | 64.4 |
|  | 05-DEC-2012 | 153 | .1 | .1 | 64.5 |
|  | 06-DEC-2012 | 150 | .1 | .1 | 64.5 |
|  | 07-DEC-2012 | 168 | .1 | .1 | 64.6 |
|  | 08-DEC-2012 | 134 | .1 | .1 | 64.7 |
|  | 09-DEC-2012 | 182 | .1 | .1 | 64.8 |
|  | 10-DEC-2012 | 329 | .2 | .2 | 65.0 |
|  | 11-DEC-2012 | 118 | .1 | .1 | 65.0 |
|  | 12-DEC-2012 | 210 | .1 | .1 | 65.1 |
|  | 13-DEC-2012 | 79 | .0 | .0 | 65.2 |
|  | 14-DEC-2012 | 132 | .1 | .1 | 65.3 |
|  | 15-DEC-2012 | 238 | .1 | .1 | 65.4 |
|  | 16-DEC-2012 | 170 | .1 | .1 | 65.5 |
|  | 17-DEC-2012 | 110 | .1 | .1 | 65.5 |
|  | 18-DEC-2012 | 73 | .0 | .0 | 65.6 |
|  | 19-DEC-2012 | 73 | .0 | .0 | 65.6 |
|  | 20-DEC-2012 | 209 | .1 | .1 | 65.7 |
|  | 21-DEC-2012 | 80 | .0 | .0 | 65.8 |
|  | 22-DEC-2012 | 105 | .1 | .1 | 65.8 |
|  | 23-DEC-2012 | 75 | .0 | .0 | 65.9 |
|  | 24-DEC-2012 | 57 | .0 | .0 | 65.9 |
|  | 25-DEC-2012 | 216 | .1 | .1 | 66.0 |
|  | 26-DEC-2012 | 124 | .1 | .1 | 66.1 |
|  | 27-DEC-2012 | 72 | .0 | .0 | 66.1 |
|  | 28-DEC-2012 | 132 | .1 | .1 | 66.2 |
|  | 29-DEC-2012 | 90 | .0 | .0 | 66.2 |
|  | 30-DEC-2012 | 101 | .1 | .1 | 66.3 |
|  | 31-DEC-2012 | 134 | .1 | .1 | 66.3 |
|  | 01-JAN-2013 | 1156 | .6 | .6 | 66.9 |
|  | 02-JAN-2013 | 531 | .3 | .3 | 67.2 |
|  | 03-JAN-2013 | 318 | .2 | .2 | 67.4 |
|  | 04-JAN-2013 | 201 | .1 | .1 | 67.5 |
|  | 05-JAN-2013 | 285 | .1 | .1 | 67.6 |
|  | 06-JAN-2013 | 112 | .1 | .1 | 67.7 |
|  | 07-JAN-2013 | 222 | .1 | .1 | 67.8 |
|  | 08-JAN-2013 | 136 | .1 | .1 | 67.9 |
|  | 09-JAN-2013 | 225 | .1 | .1 | 68.0 |
|  | 10-JAN-2013 | 471 | .2 | .2 | 68.3 |
|  | 11-JAN-2013 | 151 | .1 | .1 | 68.3 |
|  | 12-JAN-2013 | 241 | .1 | .1 | 68.5 |
|  | 13-JAN-2013 | 123 | .1 | .1 | 68.5 |
|  | 14-JAN-2013 | 114 | .1 | .1 | 68.6 |
|  | 15-JAN-2013 | 208 | .1 | .1 | 68.7 |
|  | 16-JAN-2013 | 131 | .1 | .1 | 68.8 |
|  | 17-JAN-2013 | 81 | .0 | .0 | 68.8 |
|  | 18-JAN-2013 | 99 | .1 | .1 | 68.9 |
|  | 19-JAN-2013 | 109 | .1 | .1 | 68.9 |
|  | 20-JAN-2013 | 334 | .2 | .2 | 69.1 |
|  | 21-JAN-2013 | 124 | .1 | .1 | 69.2 |
|  | 22-JAN-2013 | 93 | .0 | .0 | 69.2 |
|  | 23-JAN-2013 | 117 | .1 | .1 | 69.3 |
|  | 24-JAN-2013 | 88 | .0 | .0 | 69.3 |
|  | 25-JAN-2013 | 254 | .1 | .1 | 69.5 |
|  | 26-JAN-2013 | 76 | .0 | .0 | 69.5 |
|  | 27-JAN-2013 | 86 | .0 | .0 | 69.5 |
|  | 28-JAN-2013 | 114 | .1 | .1 | 69.6 |
|  | 29-JAN-2013 | 57 | .0 | .0 | 69.6 |
|  | 30-JAN-2013 | 75 | .0 | .0 | 69.7 |
|  | 31-JAN-2013 | 47 | .0 | .0 | 69.7 |
|  | 01-FEB-2013 | 548 | .3 | .3 | 70.0 |
|  | 02-FEB-2013 | 268 | .1 | .1 | 70.1 |
|  | 03-FEB-2013 | 184 | .1 | .1 | 70.2 |
|  | 04-FEB-2013 | 107 | .1 | .1 | 70.3 |
|  | 05-FEB-2013 | 164 | .1 | .1 | 70.4 |
|  | 06-FEB-2013 | 100 | .1 | .1 | 70.4 |
|  | 07-FEB-2013 | 147 | .1 | .1 | 70.5 |
|  | 08-FEB-2013 | 76 | .0 | .0 | 70.5 |
|  | 09-FEB-2013 | 148 | .1 | .1 | 70.6 |
|  | 10-FEB-2013 | 366 | .2 | .2 | 70.8 |
|  | 11-FEB-2013 | 99 | .1 | .1 | 70.9 |
|  | 12-FEB-2013 | 147 | .1 | .1 | 70.9 |
|  | 13-FEB-2013 | 133 | .1 | .1 | 71.0 |
|  | 14-FEB-2013 | 179 | .1 | .1 | 71.1 |
|  | 15-FEB-2013 | 203 | .1 | .1 | 71.2 |
|  | 16-FEB-2013 | 118 | .1 | .1 | 71.3 |
|  | 17-FEB-2013 | 101 | .1 | .1 | 71.3 |
|  | 18-FEB-2013 | 152 | .1 | .1 | 71.4 |
|  | 19-FEB-2013 | 111 | .1 | .1 | 71.5 |
|  | 20-FEB-2013 | 275 | .1 | .1 | 71.6 |
|  | 21-FEB-2013 | 58 | .0 | .0 | 71.6 |
|  | 22-FEB-2013 | 113 | .1 | .1 | 71.7 |
|  | 23-FEB-2013 | 69 | .0 | .0 | 71.7 |
|  | 24-FEB-2013 | 89 | .0 | .0 | 71.8 |
|  | 25-FEB-2013 | 210 | .1 | .1 | 71.9 |
|  | 26-FEB-2013 | 83 | .0 | .0 | 71.9 |
|  | 27-FEB-2013 | 44 | .0 | .0 | 71.9 |
|  | 28-FEB-2013 | 238 | .1 | .1 | 72.1 |
|  | 01-MAR-2013 | 611 | .3 | .3 | 72.4 |
|  | 02-MAR-2013 | 347 | .2 | .2 | 72.6 |
|  | 03-MAR-2013 | 139 | .1 | .1 | 72.7 |
|  | 04-MAR-2013 | 140 | .1 | .1 | 72.7 |
|  | 05-MAR-2013 | 193 | .1 | .1 | 72.8 |
|  | 06-MAR-2013 | 133 | .1 | .1 | 72.9 |
|  | 07-MAR-2013 | 133 | .1 | .1 | 73.0 |
|  | 08-MAR-2013 | 154 | .1 | .1 | 73.0 |
|  | 09-MAR-2013 | 160 | .1 | .1 | 73.1 |
|  | 10-MAR-2013 | 324 | .2 | .2 | 73.3 |
|  | 11-MAR-2013 | 107 | .1 | .1 | 73.4 |
|  | 12-MAR-2013 | 144 | .1 | .1 | 73.4 |
|  | 13-MAR-2013 | 172 | .1 | .1 | 73.5 |
|  | 14-MAR-2013 | 148 | .1 | .1 | 73.6 |
|  | 15-MAR-2013 | 169 | .1 | .1 | 73.7 |
|  | 16-MAR-2013 | 140 | .1 | .1 | 73.8 |
|  | 17-MAR-2013 | 92 | .0 | .0 | 73.8 |
|  | 18-MAR-2013 | 97 | .1 | .1 | 73.9 |
|  | 19-MAR-2013 | 83 | .0 | .0 | 73.9 |
|  | 20-MAR-2013 | 274 | .1 | .1 | 74.0 |
|  | 21-MAR-2013 | 62 | .0 | .0 | 74.1 |
|  | 22-MAR-2013 | 107 | .1 | .1 | 74.1 |
|  | 23-MAR-2013 | 66 | .0 | .0 | 74.2 |
|  | 24-MAR-2013 | 112 | .1 | .1 | 74.2 |
|  | 25-MAR-2013 | 102 | .1 | .1 | 74.3 |
|  | 26-MAR-2013 | 106 | .1 | .1 | 74.3 |
|  | 27-MAR-2013 | 69 | .0 | .0 | 74.4 |
|  | 28-MAR-2013 | 136 | .1 | .1 | 74.4 |
|  | 29-MAR-2013 | 97 | .1 | .1 | 74.5 |
|  | 30-MAR-2013 | 119 | .1 | .1 | 74.6 |
|  | 31-MAR-2013 | 63 | .0 | .0 | 74.6 |
|  | 01-APR-2013 | 372 | .2 | .2 | 74.8 |
|  | 02-APR-2013 | 248 | .1 | .1 | 74.9 |
|  | 03-APR-2013 | 117 | .1 | .1 | 75.0 |
|  | 04-APR-2013 | 159 | .1 | .1 | 75.1 |
|  | 05-APR-2013 | 200 | .1 | .1 | 75.2 |
|  | 06-APR-2013 | 89 | .0 | .0 | 75.2 |
|  | 07-APR-2013 | 139 | .1 | .1 | 75.3 |
|  | 08-APR-2013 | 131 | .1 | .1 | 75.4 |
|  | 09-APR-2013 | 88 | .0 | .0 | 75.4 |
|  | 10-APR-2013 | 351 | .2 | .2 | 75.6 |
|  | 11-APR-2013 | 120 | .1 | .1 | 75.7 |
|  | 12-APR-2013 | 151 | .1 | .1 | 75.7 |
|  | 13-APR-2013 | 96 | .1 | .1 | 75.8 |
|  | 14-APR-2013 | 108 | .1 | .1 | 75.8 |
|  | 15-APR-2013 | 104 | .1 | .1 | 75.9 |
|  | 16-APR-2013 | 66 | .0 | .0 | 75.9 |
|  | 17-APR-2013 | 102 | .1 | .1 | 76.0 |
|  | 18-APR-2013 | 85 | .0 | .0 | 76.0 |
|  | 19-APR-2013 | 82 | .0 | .0 | 76.1 |
|  | 20-APR-2013 | 303 | .2 | .2 | 76.2 |
|  | 21-APR-2013 | 52 | .0 | .0 | 76.3 |
|  | 22-APR-2013 | 67 | .0 | .0 | 76.3 |
|  | 23-APR-2013 | 62 | .0 | .0 | 76.3 |
|  | 24-APR-2013 | 52 | .0 | .0 | 76.4 |
|  | 25-APR-2013 | 133 | .1 | .1 | 76.4 |
|  | 26-APR-2013 | 86 | .0 | .0 | 76.5 |
|  | 27-APR-2013 | 83 | .0 | .0 | 76.5 |
|  | 28-APR-2013 | 105 | .1 | .1 | 76.6 |
|  | 29-APR-2013 | 98 | .1 | .1 | 76.6 |
|  | 30-APR-2013 | 168 | .1 | .1 | 76.7 |
|  | 01-MAY-2013 | 493 | .3 | .3 | 77.0 |
|  | 02-MAY-2013 | 258 | .1 | .1 | 77.1 |
|  | 03-MAY-2013 | 149 | .1 | .1 | 77.2 |
|  | 04-MAY-2013 | 112 | .1 | .1 | 77.2 |
|  | 05-MAY-2013 | 184 | .1 | .1 | 77.3 |
|  | 06-MAY-2013 | 112 | .1 | .1 | 77.4 |
|  | 07-MAY-2013 | 137 | .1 | .1 | 77.5 |
|  | 08-MAY-2013 | 94 | .0 | .0 | 77.5 |
|  | 09-MAY-2013 | 156 | .1 | .1 | 77.6 |
|  | 10-MAY-2013 | 262 | .1 | .1 | 77.7 |
|  | 11-MAY-2013 | 83 | .0 | .0 | 77.8 |
|  | 12-MAY-2013 | 159 | .1 | .1 | 77.9 |
|  | 13-MAY-2013 | 85 | .0 | .0 | 77.9 |
|  | 14-MAY-2013 | 72 | .0 | .0 | 77.9 |
|  | 15-MAY-2013 | 192 | .1 | .1 | 78.0 |
|  | 16-MAY-2013 | 94 | .0 | .0 | 78.1 |
|  | 17-MAY-2013 | 95 | .0 | .0 | 78.1 |
|  | 18-MAY-2013 | 148 | .1 | .1 | 78.2 |
|  | 19-MAY-2013 | 61 | .0 | .0 | 78.3 |
|  | 20-MAY-2013 | 239 | .1 | .1 | 78.4 |
|  | 21-MAY-2013 | 66 | .0 | .0 | 78.4 |
|  | 22-MAY-2013 | 97 | .1 | .1 | 78.5 |
|  | 23-MAY-2013 | 53 | .0 | .0 | 78.5 |
|  | 24-MAY-2013 | 82 | .0 | .0 | 78.5 |
|  | 25-MAY-2013 | 154 | .1 | .1 | 78.6 |
|  | 26-MAY-2013 | 65 | .0 | .0 | 78.7 |
|  | 27-MAY-2013 | 60 | .0 | .0 | 78.7 |
|  | 28-MAY-2013 | 111 | .1 | .1 | 78.7 |
|  | 29-MAY-2013 | 67 | .0 | .0 | 78.8 |
|  | 30-MAY-2013 | 87 | .0 | .0 | 78.8 |
|  | 31-MAY-2013 | 17 | .0 | .0 | 78.8 |
|  | 01-JUN-2013 | 333 | .2 | .2 | 79.0 |
|  | 02-JUN-2013 | 243 | .1 | .1 | 79.1 |
|  | 03-JUN-2013 | 150 | .1 | .1 | 79.2 |
|  | 04-JUN-2013 | 154 | .1 | .1 | 79.3 |
|  | 05-JUN-2013 | 139 | .1 | .1 | 79.4 |
|  | 06-JUN-2013 | 188 | .1 | .1 | 79.5 |
|  | 07-JUN-2013 | 123 | .1 | .1 | 79.5 |
|  | 08-JUN-2013 | 84 | .0 | .0 | 79.6 |
|  | 09-JUN-2013 | 152 | .1 | .1 | 79.7 |
|  | 10-JUN-2013 | 208 | .1 | .1 | 79.8 |
|  | 11-JUN-2013 | 45 | .0 | .0 | 79.8 |
|  | 12-JUN-2013 | 113 | .1 | .1 | 79.8 |
|  | 13-JUN-2013 | 71 | .0 | .0 | 79.9 |
|  | 14-JUN-2013 | 76 | .0 | .0 | 79.9 |
|  | 15-JUN-2013 | 191 | .1 | .1 | 80.0 |
|  | 16-JUN-2013 | 47 | .0 | .0 | 80.0 |
|  | 17-JUN-2013 | 75 | .0 | .0 | 80.1 |
|  | 18-JUN-2013 | 99 | .1 | .1 | 80.1 |
|  | 19-JUN-2013 | 45 | .0 | .0 | 80.2 |
|  | 20-JUN-2013 | 297 | .2 | .2 | 80.3 |
|  | 21-JUN-2013 | 31 | .0 | .0 | 80.3 |
|  | 22-JUN-2013 | 65 | .0 | .0 | 80.4 |
|  | 23-JUN-2013 | 47 | .0 | .0 | 80.4 |
|  | 24-JUN-2013 | 68 | .0 | .0 | 80.4 |
|  | 25-JUN-2013 | 114 | .1 | .1 | 80.5 |
|  | 26-JUN-2013 | 68 | .0 | .0 | 80.5 |
|  | 27-JUN-2013 | 50 | .0 | .0 | 80.6 |
|  | 28-JUN-2013 | 76 | .0 | .0 | 80.6 |
|  | 29-JUN-2013 | 74 | .0 | .0 | 80.6 |
|  | 30-JUN-2013 | 78 | .0 | .0 | 80.7 |
|  | 01-JUL-2013 | 313 | .2 | .2 | 80.8 |
|  | 02-JUL-2013 | 157 | .1 | .1 | 80.9 |
|  | 03-JUL-2013 | 95 | .0 | .0 | 81.0 |
|  | 04-JUL-2013 | 103 | .1 | .1 | 81.0 |
|  | 05-JUL-2013 | 285 | .1 | .1 | 81.2 |
|  | 06-JUL-2013 | 132 | .1 | .1 | 81.2 |
|  | 07-JUL-2013 | 84 | .0 | .0 | 81.3 |
|  | 08-JUL-2013 | 76 | .0 | .0 | 81.3 |
|  | 09-JUL-2013 | 87 | .0 | .0 | 81.4 |
|  | 10-JUL-2013 | 207 | .1 | .1 | 81.5 |
|  | 11-JUL-2013 | 83 | .0 | .0 | 81.5 |
|  | 12-JUL-2013 | 117 | .1 | .1 | 81.6 |
|  | 13-JUL-2013 | 60 | .0 | .0 | 81.6 |
|  | 14-JUL-2013 | 56 | .0 | .0 | 81.6 |
|  | 15-JUL-2013 | 178 | .1 | .1 | 81.7 |
|  | 16-JUL-2013 | 78 | .0 | .0 | 81.8 |
|  | 17-JUL-2013 | 73 | .0 | .0 | 81.8 |
|  | 18-JUL-2013 | 121 | .1 | .1 | 81.9 |
|  | 19-JUL-2013 | 125 | .1 | .1 | 82.0 |
|  | 20-JUL-2013 | 177 | .1 | .1 | 82.0 |
|  | 21-JUL-2013 | 48 | .0 | .0 | 82.1 |
|  | 22-JUL-2013 | 113 | .1 | .1 | 82.1 |
|  | 23-JUL-2013 | 57 | .0 | .0 | 82.2 |
|  | 24-JUL-2013 | 82 | .0 | .0 | 82.2 |
|  | 25-JUL-2013 | 96 | .1 | .1 | 82.3 |
|  | 26-JUL-2013 | 51 | .0 | .0 | 82.3 |
|  | 27-JUL-2013 | 96 | .1 | .1 | 82.3 |
|  | 28-JUL-2013 | 134 | .1 | .1 | 82.4 |
|  | 29-JUL-2013 | 75 | .0 | .0 | 82.4 |
|  | 30-JUL-2013 | 79 | .0 | .0 | 82.5 |
|  | 31-JUL-2013 | 83 | .0 | .0 | 82.5 |
|  | 01-AUG-2013 | 230 | .1 | .1 | 82.6 |
|  | 02-AUG-2013 | 203 | .1 | .1 | 82.8 |
|  | 03-AUG-2013 | 148 | .1 | .1 | 82.8 |
|  | 04-AUG-2013 | 98 | .1 | .1 | 82.9 |
|  | 05-AUG-2013 | 146 | .1 | .1 | 83.0 |
|  | 06-AUG-2013 | 85 | .0 | .0 | 83.0 |
|  | 07-AUG-2013 | 116 | .1 | .1 | 83.1 |
|  | 08-AUG-2013 | 141 | .1 | .1 | 83.1 |
|  | 09-AUG-2013 | 114 | .1 | .1 | 83.2 |
|  | 10-AUG-2013 | 321 | .2 | .2 | 83.4 |
|  | 11-AUG-2013 | 126 | .1 | .1 | 83.4 |
|  | 12-AUG-2013 | 69 | .0 | .0 | 83.5 |
|  | 13-AUG-2013 | 88 | .0 | .0 | 83.5 |
|  | 14-AUG-2013 | 98 | .1 | .1 | 83.6 |
|  | 15-AUG-2013 | 101 | .1 | .1 | 83.6 |
|  | 16-AUG-2013 | 99 | .1 | .1 | 83.7 |
|  | 17-AUG-2013 | 66 | .0 | .0 | 83.7 |
|  | 18-AUG-2013 | 71 | .0 | .0 | 83.7 |
|  | 19-AUG-2013 | 127 | .1 | .1 | 83.8 |
|  | 20-AUG-2013 | 213 | .1 | .1 | 83.9 |
|  | 21-AUG-2013 | 58 | .0 | .0 | 84.0 |
|  | 22-AUG-2013 | 89 | .0 | .0 | 84.0 |
|  | 23-AUG-2013 | 61 | .0 | .0 | 84.0 |
|  | 24-AUG-2013 | 91 | .0 | .0 | 84.1 |
|  | 25-AUG-2013 | 115 | .1 | .1 | 84.1 |
|  | 26-AUG-2013 | 83 | .0 | .0 | 84.2 |
|  | 27-AUG-2013 | 111 | .1 | .1 | 84.2 |
|  | 28-AUG-2013 | 138 | .1 | .1 | 84.3 |
|  | 29-AUG-2013 | 60 | .0 | .0 | 84.3 |
|  | 30-AUG-2013 | 92 | .0 | .0 | 84.4 |
|  | 31-AUG-2013 | 25 | .0 | .0 | 84.4 |
|  | 01-SEP-2013 | 186 | .1 | .1 | 84.5 |
|  | 02-SEP-2013 | 131 | .1 | .1 | 84.6 |
|  | 03-SEP-2013 | 144 | .1 | .1 | 84.6 |
|  | 04-SEP-2013 | 75 | .0 | .0 | 84.7 |
|  | 05-SEP-2013 | 104 | .1 | .1 | 84.7 |
|  | 06-SEP-2013 | 61 | .0 | .0 | 84.8 |
|  | 07-SEP-2013 | 132 | .1 | .1 | 84.8 |
|  | 08-SEP-2013 | 101 | .1 | .1 | 84.9 |
|  | 09-SEP-2013 | 103 | .1 | .1 | 85.0 |
|  | 10-SEP-2013 | 233 | .1 | .1 | 85.1 |
|  | 11-SEP-2013 | 65 | .0 | .0 | 85.1 |
|  | 12-SEP-2013 | 62 | .0 | .0 | 85.1 |
|  | 13-SEP-2013 | 114 | .1 | .1 | 85.2 |
|  | 14-SEP-2013 | 82 | .0 | .0 | 85.2 |
|  | 15-SEP-2013 | 120 | .1 | .1 | 85.3 |
|  | 16-SEP-2013 | 100 | .1 | .1 | 85.4 |
|  | 17-SEP-2013 | 100 | .1 | .1 | 85.4 |
|  | 18-SEP-2013 | 111 | .1 | .1 | 85.5 |
|  | 19-SEP-2013 | 60 | .0 | .0 | 85.5 |
|  | 20-SEP-2013 | 157 | .1 | .1 | 85.6 |
|  | 21-SEP-2013 | 46 | .0 | .0 | 85.6 |
|  | 22-SEP-2013 | 33 | .0 | .0 | 85.6 |
|  | 23-SEP-2013 | 76 | .0 | .0 | 85.7 |
|  | 24-SEP-2013 | 44 | .0 | .0 | 85.7 |
|  | 25-SEP-2013 | 128 | .1 | .1 | 85.8 |
|  | 26-SEP-2013 | 70 | .0 | .0 | 85.8 |
|  | 27-SEP-2013 | 28 | .0 | .0 | 85.8 |
|  | 28-SEP-2013 | 74 | .0 | .0 | 85.8 |
|  | 29-SEP-2013 | 64 | .0 | .0 | 85.9 |
|  | 30-SEP-2013 | 65 | .0 | .0 | 85.9 |
|  | 01-OCT-2013 | 149 | .1 | .1 | 86.0 |
|  | 02-OCT-2013 | 160 | .1 | .1 | 86.1 |
|  | 03-OCT-2013 | 108 | .1 | .1 | 86.1 |
|  | 04-OCT-2013 | 73 | .0 | .0 | 86.2 |
|  | 05-OCT-2013 | 169 | .1 | .1 | 86.3 |
|  | 06-OCT-2013 | 105 | .1 | .1 | 86.3 |
|  | 07-OCT-2013 | 137 | .1 | .1 | 86.4 |
|  | 08-OCT-2013 | 137 | .1 | .1 | 86.5 |
|  | 09-OCT-2013 | 155 | .1 | .1 | 86.5 |
|  | 10-OCT-2013 | 310 | .2 | .2 | 86.7 |
|  | 11-OCT-2013 | 127 | .1 | .1 | 86.8 |
|  | 12-OCT-2013 | 75 | .0 | .0 | 86.8 |
|  | 13-OCT-2013 | 113 | .1 | .1 | 86.9 |
|  | 14-OCT-2013 | 91 | .0 | .0 | 86.9 |
|  | 15-OCT-2013 | 168 | .1 | .1 | 87.0 |
|  | 16-OCT-2013 | 137 | .1 | .1 | 87.1 |
|  | 17-OCT-2013 | 137 | .1 | .1 | 87.1 |
|  | 18-OCT-2013 | 64 | .0 | .0 | 87.2 |
|  | 19-OCT-2013 | 111 | .1 | .1 | 87.2 |
|  | 20-OCT-2013 | 146 | .1 | .1 | 87.3 |
|  | 21-OCT-2013 | 83 | .0 | .0 | 87.4 |
|  | 22-OCT-2013 | 73 | .0 | .0 | 87.4 |
|  | 23-OCT-2013 | 113 | .1 | .1 | 87.5 |
|  | 24-OCT-2013 | 62 | .0 | .0 | 87.5 |
|  | 25-OCT-2013 | 191 | .1 | .1 | 87.6 |
|  | 26-OCT-2013 | 97 | .1 | .1 | 87.6 |
|  | 27-OCT-2013 | 96 | .1 | .1 | 87.7 |
|  | 28-OCT-2013 | 131 | .1 | .1 | 87.8 |
|  | 29-OCT-2013 | 125 | .1 | .1 | 87.8 |
|  | 30-OCT-2013 | 112 | .1 | .1 | 87.9 |
|  | 31-OCT-2013 | 56 | .0 | .0 | 87.9 |
|  | 01-NOV-2013 | 121 | .1 | .1 | 88.0 |
|  | 02-NOV-2013 | 165 | .1 | .1 | 88.1 |
|  | 03-NOV-2013 | 136 | .1 | .1 | 88.1 |
|  | 04-NOV-2013 | 94 | .0 | .0 | 88.2 |
|  | 05-NOV-2013 | 201 | .1 | .1 | 88.3 |
|  | 06-NOV-2013 | 116 | .1 | .1 | 88.4 |
|  | 07-NOV-2013 | 76 | .0 | .0 | 88.4 |
|  | 08-NOV-2013 | 80 | .0 | .0 | 88.4 |
|  | 09-NOV-2013 | 93 | .0 | .0 | 88.5 |
|  | 10-NOV-2013 | 155 | .1 | .1 | 88.6 |
|  | 11-NOV-2013 | 99 | .1 | .1 | 88.6 |
|  | 12-NOV-2013 | 146 | .1 | .1 | 88.7 |
|  | 13-NOV-2013 | 113 | .1 | .1 | 88.8 |
|  | 14-NOV-2013 | 62 | .0 | .0 | 88.8 |
|  | 15-NOV-2013 | 167 | .1 | .1 | 88.9 |
|  | 16-NOV-2013 | 106 | .1 | .1 | 88.9 |
|  | 17-NOV-2013 | 78 | .0 | .0 | 89.0 |
|  | 18-NOV-2013 | 66 | .0 | .0 | 89.0 |
|  | 19-NOV-2013 | 48 | .0 | .0 | 89.0 |
|  | 20-NOV-2013 | 157 | .1 | .1 | 89.1 |
|  | 21-NOV-2013 | 95 | .0 | .0 | 89.2 |
|  | 22-NOV-2013 | 131 | .1 | .1 | 89.2 |
|  | 23-NOV-2013 | 135 | .1 | .1 | 89.3 |
|  | 24-NOV-2013 | 139 | .1 | .1 | 89.4 |
|  | 25-NOV-2013 | 175 | .1 | .1 | 89.5 |
|  | 26-NOV-2013 | 82 | .0 | .0 | 89.5 |
|  | 27-NOV-2013 | 96 | .1 | .1 | 89.6 |
|  | 28-NOV-2013 | 114 | .1 | .1 | 89.6 |
|  | 29-NOV-2013 | 143 | .1 | .1 | 89.7 |
|  | 30-NOV-2013 | 132 | .1 | .1 | 89.8 |
|  | 01-DEC-2013 | 205 | .1 | .1 | 89.9 |
|  | 02-DEC-2013 | 121 | .1 | .1 | 89.9 |
|  | 03-DEC-2013 | 123 | .1 | .1 | 90.0 |
|  | 04-DEC-2013 | 101 | .1 | .1 | 90.1 |
|  | 05-DEC-2013 | 155 | .1 | .1 | 90.1 |
|  | 06-DEC-2013 | 82 | .0 | .0 | 90.2 |
|  | 07-DEC-2013 | 87 | .0 | .0 | 90.2 |
|  | 08-DEC-2013 | 80 | .0 | .0 | 90.3 |
|  | 09-DEC-2013 | 73 | .0 | .0 | 90.3 |
|  | 10-DEC-2013 | 142 | .1 | .1 | 90.4 |
|  | 11-DEC-2013 | 61 | .0 | .0 | 90.4 |
|  | 12-DEC-2013 | 113 | .1 | .1 | 90.5 |
|  | 13-DEC-2013 | 118 | .1 | .1 | 90.5 |
|  | 14-DEC-2013 | 138 | .1 | .1 | 90.6 |
|  | 15-DEC-2013 | 142 | .1 | .1 | 90.7 |
|  | 16-DEC-2013 | 135 | .1 | .1 | 90.8 |
|  | 17-DEC-2013 | 90 | .0 | .0 | 90.8 |
|  | 18-DEC-2013 | 102 | .1 | .1 | 90.9 |
|  | 19-DEC-2013 | 51 | .0 | .0 | 90.9 |
|  | 20-DEC-2013 | 219 | .1 | .1 | 91.0 |
|  | 21-DEC-2013 | 59 | .0 | .0 | 91.0 |
|  | 22-DEC-2013 | 103 | .1 | .1 | 91.1 |
|  | 23-DEC-2013 | 69 | .0 | .0 | 91.1 |
|  | 24-DEC-2013 | 103 | .1 | .1 | 91.2 |
|  | 25-DEC-2013 | 121 | .1 | .1 | 91.2 |
|  | 26-DEC-2013 | 96 | .1 | .1 | 91.3 |
|  | 27-DEC-2013 | 79 | .0 | .0 | 91.3 |
|  | 28-DEC-2013 | 105 | .1 | .1 | 91.4 |
|  | 29-DEC-2013 | 103 | .1 | .1 | 91.4 |
|  | 30-DEC-2013 | 58 | .0 | .0 | 91.5 |
|  | 31-DEC-2013 | 30 | .0 | .0 | 91.5 |
|  | 01-JAN-2014 | 300 | .2 | .2 | 91.6 |
|  | 02-JAN-2014 | 168 | .1 | .1 | 91.7 |
|  | 03-JAN-2014 | 134 | .1 | .1 | 91.8 |
|  | 04-JAN-2014 | 131 | .1 | .1 | 91.9 |
|  | 05-JAN-2014 | 127 | .1 | .1 | 91.9 |
|  | 06-JAN-2014 | 83 | .0 | .0 | 92.0 |
|  | 07-JAN-2014 | 112 | .1 | .1 | 92.0 |
|  | 08-JAN-2014 | 107 | .1 | .1 | 92.1 |
|  | 09-JAN-2014 | 141 | .1 | .1 | 92.2 |
|  | 10-JAN-2014 | 206 | .1 | .1 | 92.3 |
|  | 11-JAN-2014 | 48 | .0 | .0 | 92.3 |
|  | 12-JAN-2014 | 119 | .1 | .1 | 92.4 |
|  | 13-JAN-2014 | 87 | .0 | .0 | 92.4 |
|  | 14-JAN-2014 | 104 | .1 | .1 | 92.5 |
|  | 15-JAN-2014 | 166 | .1 | .1 | 92.6 |
|  | 16-JAN-2014 | 70 | .0 | .0 | 92.6 |
|  | 17-JAN-2014 | 111 | .1 | .1 | 92.6 |
|  | 18-JAN-2014 | 66 | .0 | .0 | 92.7 |
|  | 19-JAN-2014 | 45 | .0 | .0 | 92.7 |
|  | 20-JAN-2014 | 223 | .1 | .1 | 92.8 |
|  | 21-JAN-2014 | 63 | .0 | .0 | 92.9 |
|  | 22-JAN-2014 | 43 | .0 | .0 | 92.9 |
|  | 23-JAN-2014 | 66 | .0 | .0 | 92.9 |
|  | 24-JAN-2014 | 46 | .0 | .0 | 92.9 |
|  | 25-JAN-2014 | 135 | .1 | .1 | 93.0 |
|  | 26-JAN-2014 | 65 | .0 | .0 | 93.0 |
|  | 27-JAN-2014 | 44 | .0 | .0 | 93.1 |
|  | 28-JAN-2014 | 119 | .1 | .1 | 93.1 |
|  | 29-JAN-2014 | 63 | .0 | .0 | 93.2 |
|  | 30-JAN-2014 | 69 | .0 | .0 | 93.2 |
|  | 31-JAN-2014 | 40 | .0 | .0 | 93.2 |
|  | 01-FEB-2014 | 161 | .1 | .1 | 93.3 |
|  | 02-FEB-2014 | 147 | .1 | .1 | 93.4 |
|  | 03-FEB-2014 | 75 | .0 | .0 | 93.4 |
|  | 04-FEB-2014 | 95 | .0 | .0 | 93.5 |
|  | 05-FEB-2014 | 152 | .1 | .1 | 93.5 |
|  | 06-FEB-2014 | 59 | .0 | .0 | 93.6 |
|  | 07-FEB-2014 | 106 | .1 | .1 | 93.6 |
|  | 08-FEB-2014 | 86 | .0 | .0 | 93.7 |
|  | 09-FEB-2014 | 56 | .0 | .0 | 93.7 |
|  | 10-FEB-2014 | 196 | .1 | .1 | 93.8 |
|  | 11-FEB-2014 | 59 | .0 | .0 | 93.8 |
|  | 12-FEB-2014 | 90 | .0 | .0 | 93.9 |
|  | 13-FEB-2014 | 80 | .0 | .0 | 93.9 |
|  | 14-FEB-2014 | 109 | .1 | .1 | 94.0 |
|  | 15-FEB-2014 | 70 | .0 | .0 | 94.0 |
|  | 16-FEB-2014 | 64 | .0 | .0 | 94.1 |
|  | 17-FEB-2014 | 63 | .0 | .0 | 94.1 |
|  | 18-FEB-2014 | 90 | .0 | .0 | 94.1 |
|  | 19-FEB-2014 | 71 | .0 | .0 | 94.2 |
|  | 20-FEB-2014 | 96 | .1 | .1 | 94.2 |
|  | 21-FEB-2014 | 41 | .0 | .0 | 94.3 |
|  | 22-FEB-2014 | 53 | .0 | .0 | 94.3 |
|  | 23-FEB-2014 | 46 | .0 | .0 | 94.3 |
|  | 24-FEB-2014 | 91 | .0 | .0 | 94.4 |
|  | 25-FEB-2014 | 84 | .0 | .0 | 94.4 |
|  | 26-FEB-2014 | 66 | .0 | .0 | 94.4 |
|  | 27-FEB-2014 | 52 | .0 | .0 | 94.5 |
|  | 28-FEB-2014 | 107 | .1 | .1 | 94.5 |
|  | 01-MAR-2014 | 123 | .1 | .1 | 94.6 |
|  | 02-MAR-2014 | 93 | .0 | .0 | 94.6 |
|  | 03-MAR-2014 | 76 | .0 | .0 | 94.7 |
|  | 04-MAR-2014 | 90 | .0 | .0 | 94.7 |
|  | 05-MAR-2014 | 138 | .1 | .1 | 94.8 |
|  | 06-MAR-2014 | 47 | .0 | .0 | 94.8 |
|  | 07-MAR-2014 | 63 | .0 | .0 | 94.8 |
|  | 08-MAR-2014 | 55 | .0 | .0 | 94.9 |
|  | 09-MAR-2014 | 41 | .0 | .0 | 94.9 |
|  | 10-MAR-2014 | 172 | .1 | .1 | 95.0 |
|  | 11-MAR-2014 | 43 | .0 | .0 | 95.0 |
|  | 12-MAR-2014 | 131 | .1 | .1 | 95.1 |
|  | 13-MAR-2014 | 54 | .0 | .0 | 95.1 |
|  | 14-MAR-2014 | 59 | .0 | .0 | 95.1 |
|  | 15-MAR-2014 | 150 | .1 | .1 | 95.2 |
|  | 16-MAR-2014 | 55 | .0 | .0 | 95.2 |
|  | 17-MAR-2014 | 66 | .0 | .0 | 95.3 |
|  | 18-MAR-2014 | 93 | .0 | .0 | 95.3 |
|  | 19-MAR-2014 | 26 | .0 | .0 | 95.3 |
|  | 20-MAR-2014 | 140 | .1 | .1 | 95.4 |
|  | 21-MAR-2014 | 26 | .0 | .0 | 95.4 |
|  | 22-MAR-2014 | 73 | .0 | .0 | 95.5 |
|  | 23-MAR-2014 | 55 | .0 | .0 | 95.5 |
|  | 24-MAR-2014 | 72 | .0 | .0 | 95.5 |
|  | 25-MAR-2014 | 169 | .1 | .1 | 95.6 |
|  | 26-MAR-2014 | 96 | .1 | .1 | 95.7 |
|  | 27-MAR-2014 | 52 | .0 | .0 | 95.7 |
|  | 28-MAR-2014 | 61 | .0 | .0 | 95.7 |
|  | 29-MAR-2014 | 59 | .0 | .0 | 95.8 |
|  | 30-MAR-2014 | 55 | .0 | .0 | 95.8 |
|  | 31-MAR-2014 | 41 | .0 | .0 | 95.8 |
|  | 01-APR-2014 | 99 | .1 | .1 | 95.9 |
|  | 02-APR-2014 | 91 | .0 | .0 | 95.9 |
|  | 03-APR-2014 | 75 | .0 | .0 | 96.0 |
|  | 04-APR-2014 | 40 | .0 | .0 | 96.0 |
|  | 05-APR-2014 | 111 | .1 | .1 | 96.0 |
|  | 06-APR-2014 | 25 | .0 | .0 | 96.0 |
|  | 07-APR-2014 | 47 | .0 | .0 | 96.1 |
|  | 08-APR-2014 | 67 | .0 | .0 | 96.1 |
|  | 09-APR-2014 | 43 | .0 | .0 | 96.1 |
|  | 10-APR-2014 | 161 | .1 | .1 | 96.2 |
|  | 11-APR-2014 | 35 | .0 | .0 | 96.2 |
|  | 12-APR-2014 | 24 | .0 | .0 | 96.2 |
|  | 13-APR-2014 | 55 | .0 | .0 | 96.3 |
|  | 14-APR-2014 | 84 | .0 | .0 | 96.3 |
|  | 15-APR-2014 | 48 | .0 | .0 | 96.3 |
|  | 16-APR-2014 | 79 | .0 | .0 | 96.4 |
|  | 17-APR-2014 | 49 | .0 | .0 | 96.4 |
|  | 18-APR-2014 | 53 | .0 | .0 | 96.4 |
|  | 19-APR-2014 | 55 | .0 | .0 | 96.5 |
|  | 20-APR-2014 | 45 | .0 | .0 | 96.5 |
|  | 21-APR-2014 | 34 | .0 | .0 | 96.5 |
|  | 22-APR-2014 | 37 | .0 | .0 | 96.5 |
|  | 23-APR-2014 | 32 | .0 | .0 | 96.5 |
|  | 24-APR-2014 | 44 | .0 | .0 | 96.6 |
|  | 25-APR-2014 | 58 | .0 | .0 | 96.6 |
|  | 26-APR-2014 | 42 | .0 | .0 | 96.6 |
|  | 27-APR-2014 | 61 | .0 | .0 | 96.7 |
|  | 28-APR-2014 | 76 | .0 | .0 | 96.7 |
|  | 29-APR-2014 | 41 | .0 | .0 | 96.7 |
|  | 30-APR-2014 | 35 | .0 | .0 | 96.7 |
|  | 01-MAY-2014 | 82 | .0 | .0 | 96.8 |
|  | 02-MAY-2014 | 56 | .0 | .0 | 96.8 |
|  | 03-MAY-2014 | 27 | .0 | .0 | 96.8 |
|  | 04-MAY-2014 | 21 | .0 | .0 | 96.8 |
|  | 05-MAY-2014 | 82 | .0 | .0 | 96.9 |
|  | 06-MAY-2014 | 31 | .0 | .0 | 96.9 |
|  | 07-MAY-2014 | 53 | .0 | .0 | 96.9 |
|  | 08-MAY-2014 | 56 | .0 | .0 | 96.9 |
|  | 09-MAY-2014 | 63 | .0 | .0 | 97.0 |
|  | 10-MAY-2014 | 69 | .0 | .0 | 97.0 |
|  | 11-MAY-2014 | 68 | .0 | .0 | 97.0 |
|  | 12-MAY-2014 | 18 | .0 | .0 | 97.1 |
|  | 13-MAY-2014 | 78 | .0 | .0 | 97.1 |
|  | 14-MAY-2014 | 18 | .0 | .0 | 97.1 |
|  | 15-MAY-2014 | 35 | .0 | .0 | 97.1 |
|  | 16-MAY-2014 | 48 | .0 | .0 | 97.2 |
|  | 17-MAY-2014 | 39 | .0 | .0 | 97.2 |
|  | 18-MAY-2014 | 57 | .0 | .0 | 97.2 |
|  | 19-MAY-2014 | 56 | .0 | .0 | 97.2 |
|  | 20-MAY-2014 | 96 | .1 | .1 | 97.3 |
|  | 21-MAY-2014 | 41 | .0 | .0 | 97.3 |
|  | 22-MAY-2014 | 44 | .0 | .0 | 97.3 |
|  | 23-MAY-2014 | 22 | .0 | .0 | 97.3 |
|  | 24-MAY-2014 | 22 | .0 | .0 | 97.4 |
|  | 25-MAY-2014 | 53 | .0 | .0 | 97.4 |
|  | 26-MAY-2014 | 39 | .0 | .0 | 97.4 |
|  | 27-MAY-2014 | 34 | .0 | .0 | 97.4 |
|  | 28-MAY-2014 | 23 | .0 | .0 | 97.4 |
|  | 29-MAY-2014 | 32 | .0 | .0 | 97.4 |
|  | 30-MAY-2014 | 21 | .0 | .0 | 97.5 |
|  | 31-MAY-2014 | 19 | .0 | .0 | 97.5 |
|  | 01-JUN-2014 | 62 | .0 | .0 | 97.5 |
|  | 02-JUN-2014 | 32 | .0 | .0 | 97.5 |
|  | 03-JUN-2014 | 44 | .0 | .0 | 97.5 |
|  | 04-JUN-2014 | 22 | .0 | .0 | 97.6 |
|  | 05-JUN-2014 | 43 | .0 | .0 | 97.6 |
|  | 06-JUN-2014 | 14 | .0 | .0 | 97.6 |
|  | 07-JUN-2014 | 40 | .0 | .0 | 97.6 |
|  | 08-JUN-2014 | 35 | .0 | .0 | 97.6 |
|  | 09-JUN-2014 | 55 | .0 | .0 | 97.6 |
|  | 10-JUN-2014 | 63 | .0 | .0 | 97.7 |
|  | 11-JUN-2014 | 46 | .0 | .0 | 97.7 |
|  | 12-JUN-2014 | 30 | .0 | .0 | 97.7 |
|  | 13-JUN-2014 | 18 | .0 | .0 | 97.7 |
|  | 14-JUN-2014 | 56 | .0 | .0 | 97.8 |
|  | 15-JUN-2014 | 92 | .0 | .0 | 97.8 |
|  | 16-JUN-2014 | 43 | .0 | .0 | 97.8 |
|  | 17-JUN-2014 | 43 | .0 | .0 | 97.9 |
|  | 18-JUN-2014 | 58 | .0 | .0 | 97.9 |
|  | 19-JUN-2014 | 44 | .0 | .0 | 97.9 |
|  | 20-JUN-2014 | 47 | .0 | .0 | 97.9 |
|  | 21-JUN-2014 | 22 | .0 | .0 | 97.9 |
|  | 22-JUN-2014 | 34 | .0 | .0 | 98.0 |
|  | 23-JUN-2014 | 46 | .0 | .0 | 98.0 |
|  | 24-JUN-2014 | 25 | .0 | .0 | 98.0 |
|  | 25-JUN-2014 | 34 | .0 | .0 | 98.0 |
|  | 26-JUN-2014 | 38 | .0 | .0 | 98.0 |
|  | 27-JUN-2014 | 49 | .0 | .0 | 98.1 |
|  | 28-JUN-2014 | 44 | .0 | .0 | 98.1 |
|  | 29-JUN-2014 | 39 | .0 | .0 | 98.1 |
|  | 30-JUN-2014 | 23 | .0 | .0 | 98.1 |
|  | 01-JUL-2014 | 56 | .0 | .0 | 98.1 |
|  | 02-JUL-2014 | 44 | .0 | .0 | 98.2 |
|  | 03-JUL-2014 | 43 | .0 | .0 | 98.2 |
|  | 04-JUL-2014 | 25 | .0 | .0 | 98.2 |
|  | 05-JUL-2014 | 58 | .0 | .0 | 98.2 |
|  | 06-JUL-2014 | 34 | .0 | .0 | 98.3 |
|  | 07-JUL-2014 | 28 | .0 | .0 | 98.3 |
|  | 08-JUL-2014 | 50 | .0 | .0 | 98.3 |
|  | 09-JUL-2014 | 38 | .0 | .0 | 98.3 |
|  | 10-JUL-2014 | 82 | .0 | .0 | 98.4 |
|  | 11-JUL-2014 | 36 | .0 | .0 | 98.4 |
|  | 12-JUL-2014 | 46 | .0 | .0 | 98.4 |
|  | 13-JUL-2014 | 22 | .0 | .0 | 98.4 |
|  | 14-JUL-2014 | 38 | .0 | .0 | 98.4 |
|  | 15-JUL-2014 | 98 | .1 | .1 | 98.5 |
|  | 16-JUL-2014 | 16 | .0 | .0 | 98.5 |
|  | 17-JUL-2014 | 25 | .0 | .0 | 98.5 |
|  | 18-JUL-2014 | 30 | .0 | .0 | 98.5 |
|  | 19-JUL-2014 | 30 | .0 | .0 | 98.5 |
|  | 20-JUL-2014 | 63 | .0 | .0 | 98.6 |
|  | 21-JUL-2014 | 48 | .0 | .0 | 98.6 |
|  | 22-JUL-2014 | 31 | .0 | .0 | 98.6 |
|  | 23-JUL-2014 | 39 | .0 | .0 | 98.6 |
|  | 24-JUL-2014 | 21 | .0 | .0 | 98.6 |
|  | 25-JUL-2014 | 63 | .0 | .0 | 98.7 |
|  | 26-JUL-2014 | 35 | .0 | .0 | 98.7 |
|  | 27-JUL-2014 | 33 | .0 | .0 | 98.7 |
|  | 28-JUL-2014 | 41 | .0 | .0 | 98.7 |
|  | 29-JUL-2014 | 43 | .0 | .0 | 98.8 |
|  | 30-JUL-2014 | 27 | .0 | .0 | 98.8 |
|  | 31-JUL-2014 | 6 | .0 | .0 | 98.8 |
|  | 01-AUG-2014 | 64 | .0 | .0 | 98.8 |
|  | 02-AUG-2014 | 46 | .0 | .0 | 98.8 |
|  | 03-AUG-2014 | 41 | .0 | .0 | 98.9 |
|  | 04-AUG-2014 | 33 | .0 | .0 | 98.9 |
|  | 05-AUG-2014 | 36 | .0 | .0 | 98.9 |
|  | 06-AUG-2014 | 15 | .0 | .0 | 98.9 |
|  | 07-AUG-2014 | 13 | .0 | .0 | 98.9 |
|  | 08-AUG-2014 | 31 | .0 | .0 | 98.9 |
|  | 09-AUG-2014 | 47 | .0 | .0 | 98.9 |
|  | 10-AUG-2014 | 57 | .0 | .0 | 99.0 |
|  | 11-AUG-2014 | 39 | .0 | .0 | 99.0 |
|  | 12-AUG-2014 | 36 | .0 | .0 | 99.0 |
|  | 13-AUG-2014 | 12 | .0 | .0 | 99.0 |
|  | 14-AUG-2014 | 32 | .0 | .0 | 99.0 |
|  | 15-AUG-2014 | 49 | .0 | .0 | 99.1 |
|  | 16-AUG-2014 | 14 | .0 | .0 | 99.1 |
|  | 17-AUG-2014 | 8 | .0 | .0 | 99.1 |
|  | 18-AUG-2014 | 30 | .0 | .0 | 99.1 |
|  | 19-AUG-2014 | 14 | .0 | .0 | 99.1 |
|  | 20-AUG-2014 | 42 | .0 | .0 | 99.1 |
|  | 21-AUG-2014 | 11 | .0 | .0 | 99.1 |
|  | 22-AUG-2014 | 76 | .0 | .0 | 99.2 |
|  | 23-AUG-2014 | 20 | .0 | .0 | 99.2 |
|  | 24-AUG-2014 | 30 | .0 | .0 | 99.2 |
|  | 25-AUG-2014 | 34 | .0 | .0 | 99.2 |
|  | 26-AUG-2014 | 19 | .0 | .0 | 99.2 |
|  | 27-AUG-2014 | 15 | .0 | .0 | 99.2 |
|  | 28-AUG-2014 | 34 | .0 | .0 | 99.2 |
|  | 29-AUG-2014 | 22 | .0 | .0 | 99.3 |
|  | 30-AUG-2014 | 22 | .0 | .0 | 99.3 |
|  | 31-AUG-2014 | 12 | .0 | .0 | 99.3 |
|  | 01-SEP-2014 | 53 | .0 | .0 | 99.3 |
|  | 02-SEP-2014 | 3 | .0 | .0 | 99.3 |
|  | 03-SEP-2014 | 13 | .0 | .0 | 99.3 |
|  | 04-SEP-2014 | 12 | .0 | .0 | 99.3 |
|  | 05-SEP-2014 | 17 | .0 | .0 | 99.3 |
|  | 06-SEP-2014 | 22 | .0 | .0 | 99.3 |
|  | 07-SEP-2014 | 21 | .0 | .0 | 99.4 |
|  | 08-SEP-2014 | 17 | .0 | .0 | 99.4 |
|  | 09-SEP-2014 | 24 | .0 | .0 | 99.4 |
|  | 10-SEP-2014 | 47 | .0 | .0 | 99.4 |
|  | 11-SEP-2014 | 6 | .0 | .0 | 99.4 |
|  | 12-SEP-2014 | 44 | .0 | .0 | 99.4 |
|  | 13-SEP-2014 | 10 | .0 | .0 | 99.4 |
|  | 14-SEP-2014 | 1 | .0 | .0 | 99.4 |
|  | 15-SEP-2014 | 25 | .0 | .0 | 99.4 |
|  | 16-SEP-2014 | 9 | .0 | .0 | 99.4 |
|  | 17-SEP-2014 | 25 | .0 | .0 | 99.5 |
|  | 18-SEP-2014 | 11 | .0 | .0 | 99.5 |
|  | 19-SEP-2014 | 13 | .0 | .0 | 99.5 |
|  | 20-SEP-2014 | 16 | .0 | .0 | 99.5 |
|  | 21-SEP-2014 | 3 | .0 | .0 | 99.5 |
|  | 22-SEP-2014 | 14 | .0 | .0 | 99.5 |
|  | 23-SEP-2014 | 8 | .0 | .0 | 99.5 |
|  | 24-SEP-2014 | 16 | .0 | .0 | 99.5 |
|  | 25-SEP-2014 | 17 | .0 | .0 | 99.5 |
|  | 26-SEP-2014 | 22 | .0 | .0 | 99.5 |
|  | 27-SEP-2014 | 18 | .0 | .0 | 99.5 |
|  | 28-SEP-2014 | 14 | .0 | .0 | 99.5 |
|  | 29-SEP-2014 | 3 | .0 | .0 | 99.5 |
|  | 30-SEP-2014 | 11 | .0 | .0 | 99.5 |
|  | 01-OCT-2014 | 25 | .0 | .0 | 99.6 |
|  | 02-OCT-2014 | 11 | .0 | .0 | 99.6 |
|  | 03-OCT-2014 | 11 | .0 | .0 | 99.6 |
|  | 04-OCT-2014 | 10 | .0 | .0 | 99.6 |
|  | 05-OCT-2014 | 4 | .0 | .0 | 99.6 |
|  | 06-OCT-2014 | 10 | .0 | .0 | 99.6 |
|  | 07-OCT-2014 | 4 | .0 | .0 | 99.6 |
|  | 08-OCT-2014 | 17 | .0 | .0 | 99.6 |
|  | 09-OCT-2014 | 23 | .0 | .0 | 99.6 |
|  | 10-OCT-2014 | 28 | .0 | .0 | 99.6 |
|  | 11-OCT-2014 | 16 | .0 | .0 | 99.6 |
|  | 12-OCT-2014 | 9 | .0 | .0 | 99.6 |
|  | 13-OCT-2014 | 22 | .0 | .0 | 99.6 |
|  | 14-OCT-2014 | 11 | .0 | .0 | 99.7 |
|  | 15-OCT-2014 | 2 | .0 | .0 | 99.7 |
|  | 16-OCT-2014 | 18 | .0 | .0 | 99.7 |
|  | 17-OCT-2014 | 17 | .0 | .0 | 99.7 |
|  | 18-OCT-2014 | 1 | .0 | .0 | 99.7 |
|  | 19-OCT-2014 | 5 | .0 | .0 | 99.7 |
|  | 20-OCT-2014 | 34 | .0 | .0 | 99.7 |
|  | 21-OCT-2014 | 14 | .0 | .0 | 99.7 |
|  | 22-OCT-2014 | 7 | .0 | .0 | 99.7 |
|  | 23-OCT-2014 | 13 | .0 | .0 | 99.7 |
|  | 24-OCT-2014 | 5 | .0 | .0 | 99.7 |
|  | 25-OCT-2014 | 5 | .0 | .0 | 99.7 |
|  | 26-OCT-2014 | 4 | .0 | .0 | 99.7 |
|  | 27-OCT-2014 | 11 | .0 | .0 | 99.7 |
|  | 28-OCT-2014 | 15 | .0 | .0 | 99.7 |
|  | 29-OCT-2014 | 8 | .0 | .0 | 99.7 |
|  | 30-OCT-2014 | 4 | .0 | .0 | 99.7 |
|  | 01-NOV-2014 | 10 | .0 | .0 | 99.7 |
|  | 02-NOV-2014 | 4 | .0 | .0 | 99.7 |
|  | 03-NOV-2014 | 17 | .0 | .0 | 99.8 |
|  | 04-NOV-2014 | 4 | .0 | .0 | 99.8 |
|  | 05-NOV-2014 | 13 | .0 | .0 | 99.8 |
|  | 06-NOV-2014 | 18 | .0 | .0 | 99.8 |
|  | 08-NOV-2014 | 8 | .0 | .0 | 99.8 |
|  | 09-NOV-2014 | 13 | .0 | .0 | 99.8 |
|  | 10-NOV-2014 | 16 | .0 | .0 | 99.8 |
|  | 11-NOV-2014 | 3 | .0 | .0 | 99.8 |
|  | 12-NOV-2014 | 8 | .0 | .0 | 99.8 |
|  | 13-NOV-2014 | 7 | .0 | .0 | 99.8 |
|  | 14-NOV-2014 | 6 | .0 | .0 | 99.8 |
|  | 15-NOV-2014 | 26 | .0 | .0 | 99.8 |
|  | 16-NOV-2014 | 3 | .0 | .0 | 99.8 |
|  | 17-NOV-2014 | 8 | .0 | .0 | 99.8 |
|  | 18-NOV-2014 | 15 | .0 | .0 | 99.8 |
|  | 19-NOV-2014 | 2 | .0 | .0 | 99.8 |
|  | 20-NOV-2014 | 22 | .0 | .0 | 99.8 |
|  | 21-NOV-2014 | 3 | .0 | .0 | 99.8 |
|  | 22-NOV-2014 | 9 | .0 | .0 | 99.9 |
|  | 23-NOV-2014 | 2 | .0 | .0 | 99.9 |
|  | 24-NOV-2014 | 11 | .0 | .0 | 99.9 |
|  | 25-NOV-2014 | 4 | .0 | .0 | 99.9 |
|  | 26-NOV-2014 | 3 | .0 | .0 | 99.9 |
|  | 27-NOV-2014 | 4 | .0 | .0 | 99.9 |
|  | 28-NOV-2014 | 3 | .0 | .0 | 99.9 |
|  | 29-NOV-2014 | 3 | .0 | .0 | 99.9 |
|  | 01-DEC-2014 | 21 | .0 | .0 | 99.9 |
|  | 02-DEC-2014 | 1 | .0 | .0 | 99.9 |
|  | 04-DEC-2014 | 2 | .0 | .0 | 99.9 |
|  | 05-DEC-2014 | 3 | .0 | .0 | 99.9 |
|  | 06-DEC-2014 | 18 | .0 | .0 | 99.9 |
|  | 07-DEC-2014 | 14 | .0 | .0 | 99.9 |
|  | 08-DEC-2014 | 3 | .0 | .0 | 99.9 |
|  | 09-DEC-2014 | 2 | .0 | .0 | 99.9 |
|  | 10-DEC-2014 | 7 | .0 | .0 | 99.9 |
|  | 11-DEC-2014 | 5 | .0 | .0 | 99.9 |
|  | 12-DEC-2014 | 20 | .0 | .0 | 99.9 |
|  | 13-DEC-2014 | 4 | .0 | .0 | 99.9 |
|  | 14-DEC-2014 | 8 | .0 | .0 | 99.9 |
|  | 15-DEC-2014 | 7 | .0 | .0 | 99.9 |
|  | 16-DEC-2014 | 5 | .0 | .0 | 99.9 |
|  | 17-DEC-2014 | 4 | .0 | .0 | 99.9 |
|  | 18-DEC-2014 | 3 | .0 | .0 | 99.9 |
|  | 19-DEC-2014 | 2 | .0 | .0 | 99.9 |
|  | 20-DEC-2014 | 10 | .0 | .0 | 99.9 |
|  | 21-DEC-2014 | 9 | .0 | .0 | 99.9 |
|  | 22-DEC-2014 | 1 | .0 | .0 | 99.9 |
|  | 24-DEC-2014 | 3 | .0 | .0 | 99.9 |
|  | 25-DEC-2014 | 3 | .0 | .0 | 99.9 |
|  | 26-DEC-2014 | 2 | .0 | .0 | 100.0 |
|  | 27-DEC-2014 | 5 | .0 | .0 | 100.0 |
|  | 30-DEC-2014 | 2 | .0 | .0 | 100.0 |
|  | 31-DEC-2014 | 1 | .0 | .0 | 100.0 |
|  | 01-JAN-2015 | 6 | .0 | .0 | 100.0 |
|  | 02-JAN-2015 | 5 | .0 | .0 | 100.0 |
|  | 04-JAN-2015 | 1 | .0 | .0 | 100.0 |
|  | 05-JAN-2015 | 1 | .0 | .0 | 100.0 |
|  | 07-JAN-2015 | 3 | .0 | .0 | 100.0 |
|  | 08-JAN-2015 | 1 | .0 | .0 | 100.0 |
|  | 09-JAN-2015 | 4 | .0 | .0 | 100.0 |
|  | 10-JAN-2015 | 1 | .0 | .0 | 100.0 |
|  | 11-JAN-2015 | 3 | .0 | .0 | 100.0 |
|  | 12-JAN-2015 | 2 | .0 | .0 | 100.0 |
|  | 14-JAN-2015 | 1 | .0 | .0 | 100.0 |
|  | 15-JAN-2015 | 3 | .0 | .0 | 100.0 |
|  | 16-JAN-2015 | 3 | .0 | .0 | 100.0 |
|  | 17-JAN-2015 | 2 | .0 | .0 | 100.0 |
|  | 18-JAN-2015 | 1 | .0 | .0 | 100.0 |
|  | 20-JAN-2015 | 1 | .0 | .0 | 100.0 |
|  | 21-JAN-2015 | 1 | .0 | .0 | 100.0 |
|  | 22-JAN-2015 | 1 | .0 | .0 | 100.0 |
|  | 23-JAN-2015 | 1 | .0 | .0 | 100.0 |
|  | 25-JAN-2015 | 1 | .0 | .0 | 100.0 |
|  | 28-JAN-2015 | 2 | .0 | .0 | 100.0 |
|  | 30-JAN-2015 | 1 | .0 | .0 | 100.0 |
|  | 31-JAN-2015 | 1 | .0 | .0 | 100.0 |
|  | 01-FEB-2015 | 2 | .0 | .0 | 100.0 |
|  | 02-FEB-2015 | 3 | .0 | .0 | 100.0 |
|  | 04-FEB-2015 | 1 | .0 | .0 | 100.0 |
|  | 05-FEB-2015 | 2 | .0 | .0 | 100.0 |
|  | 06-FEB-2015 | 1 | .0 | .0 | 100.0 |
|  | 07-FEB-2015 | 1 | .0 | .0 | 100.0 |
|  | 08-FEB-2015 | 1 | .0 | .0 | 100.0 |
|  | 09-FEB-2015 | 1 | .0 | .0 | 100.0 |
|  | 10-FEB-2015 | 1 | .0 | .0 | 100.0 |
|  | 11-FEB-2015 | 1 | .0 | .0 | 100.0 |
|  | 13-FEB-2015 | 1 | .0 | .0 | 100.0 |
|  | 14-FEB-2015 | 2 | .0 | .0 | 100.0 |
|  | 16-FEB-2015 | 1 | .0 | .0 | 100.0 |
|  | 17-FEB-2015 | 1 | .0 | .0 | 100.0 |
|  | 18-FEB-2015 | 3 | .0 | .0 | 100.0 |
|  | 19-FEB-2015 | 2 | .0 | .0 | 100.0 |
|  | 20-FEB-2015 | 3 | .0 | .0 | 100.0 |
|  | 22-FEB-2015 | 1 | .0 | .0 | 100.0 |
|  | 24-FEB-2015 | 1 | .0 | .0 | 100.0 |
|  | 25-FEB-2015 | 1 | .0 | .0 | 100.0 |
|  | 26-FEB-2015 | 2 | .0 | .0 | 100.0 |
|  | 27-FEB-2015 | 1 | .0 | .0 | 100.0 |
|  | 28-FEB-2015 | 1 | .0 | .0 | 100.0 |
|  | 01-MAR-2015 | 3 | .0 | .0 | 100.0 |
|  | 02-MAR-2015 | 1 | .0 | .0 | 100.0 |
|  | 04-MAR-2015 | 1 | .0 | .0 | 100.0 |
|  | 10-MAR-2015 | 1 | .0 | .0 | 100.0 |
|  | Total | 190290 | 100.0 | 100.0 |  |
|  |  |  |  |  |  |

Child ageChild age, table, 1 levels of column headers and 2 levels of row headers, table with 6 columns and 56 rows

|  |  |  |  |  |  |
| --- | --- | --- | --- | --- | --- |
|  | | Frequency | Percent | Valid Percent | Cumulative Percent |
| Valid | 8.0 | 1317 | .7 | .7 | .7 |
|  | 9.0 | 2492 | 1.3 | 1.3 | 2.0 |
|  | 10.0 | 4648 | 2.4 | 2.4 | 4.4 |
|  | 11.0 | 6796 | 3.6 | 3.6 | 8.0 |
|  | 12.0 | 6063 | 3.2 | 3.2 | 11.2 |
|  | 13.0 | 5362 | 2.8 | 2.8 | 14.0 |
|  | 14.0 | 5127 | 2.7 | 2.7 | 16.7 |
|  | 15.0 | 4702 | 2.5 | 2.5 | 19.2 |
|  | 16.0 | 5183 | 2.7 | 2.7 | 21.9 |
|  | 17.0 | 5150 | 2.7 | 2.7 | 24.6 |
|  | 18.0 | 5480 | 2.9 | 2.9 | 27.5 |
|  | 19.0 | 5042 | 2.6 | 2.6 | 30.1 |
|  | 20.0 | 5615 | 3.0 | 3.0 | 33.1 |
|  | 21.0 | 5588 | 2.9 | 2.9 | 36.0 |
|  | 22.0 | 5819 | 3.1 | 3.1 | 39.1 |
|  | 23.0 | 5874 | 3.1 | 3.1 | 42.2 |
|  | 24.0 | 5790 | 3.0 | 3.0 | 45.2 |
|  | 25.0 | 5084 | 2.7 | 2.7 | 47.9 |
|  | 26.0 | 5073 | 2.7 | 2.7 | 50.6 |
|  | 27.0 | 5265 | 2.8 | 2.8 | 53.3 |
|  | 28.0 | 5548 | 2.9 | 2.9 | 56.2 |
|  | 29.0 | 5288 | 2.8 | 2.8 | 59.0 |
|  | 30.0 | 5319 | 2.8 | 2.8 | 61.8 |
|  | 31.0 | 4996 | 2.6 | 2.6 | 64.4 |
|  | 32.0 | 4843 | 2.5 | 2.5 | 67.0 |
|  | 33.0 | 5461 | 2.9 | 2.9 | 69.9 |
|  | 34.0 | 6335 | 3.3 | 3.3 | 73.2 |
|  | 35.0 | 5917 | 3.1 | 3.1 | 76.3 |
|  | 36.0 | 5734 | 3.0 | 3.0 | 79.3 |
|  | 37.0 | 4893 | 2.6 | 2.6 | 81.9 |
|  | 38.0 | 3884 | 2.0 | 2.0 | 83.9 |
|  | 39.0 | 3565 | 1.9 | 1.9 | 85.8 |
|  | 40.0 | 3015 | 1.6 | 1.6 | 87.4 |
|  | 41.0 | 2942 | 1.5 | 1.5 | 88.9 |
|  | 42.0 | 2226 | 1.2 | 1.2 | 90.1 |
|  | 43.0 | 1717 | .9 | .9 | 91.0 |
|  | 44.0 | 1608 | .8 | .8 | 91.8 |
|  | 45.0 | 1667 | .9 | .9 | 92.7 |
|  | 46.0 | 1962 | 1.0 | 1.0 | 93.7 |
|  | 47.0 | 1807 | .9 | .9 | 94.7 |
|  | 48.0 | 1520 | .8 | .8 | 95.5 |
|  | 49.0 | 1249 | .7 | .7 | 96.2 |
|  | 50.0 | 1215 | .6 | .6 | 96.8 |
|  | 51.0 | 935 | .5 | .5 | 97.3 |
|  | 52.0 | 1014 | .5 | .5 | 97.8 |
|  | 53.0 | 938 | .5 | .5 | 98.3 |
|  | 54.0 | 694 | .4 | .4 | 98.7 |
|  | 55.0 | 636 | .3 | .3 | 99.0 |
|  | 56.0 | 455 | .2 | .2 | 99.2 |
|  | 57.0 | 409 | .2 | .2 | 99.5 |
|  | 58.0 | 468 | .2 | .2 | 99.7 |
|  | 59.0 | 397 | .2 | .2 | 99.9 |
|  | 60.0 | 163 | .1 | .1 | 100.0 |
|  | Total | 190290 | 100.0 | 100.0 |  |
|  |  |  |  |  |  |

Interview start timeInterview start time, table, 1 levels of column headers and 2 levels of row headers, table with 6 columns and 992 rows

|  |  |  |  |  |  |
| --- | --- | --- | --- | --- | --- |
|  | | Frequency | Percent | Valid Percent | Cumulative Percent |
| Valid | 13:00 | 2772 | 1.5 | 1.5 | 1.5 |
|  | 13:02 | 65 | .0 | .0 | 1.5 |
|  | 13:03 | 50 | .0 | .0 | 1.5 |
|  | 13:04 | 48 | .0 | .0 | 1.5 |
|  | 13:05 | 1284 | .7 | .7 | 2.2 |
|  | 13:06 | 57 | .0 | .0 | 2.2 |
|  | 13:07 | 66 | .0 | .0 | 2.3 |
|  | 13:08 | 70 | .0 | .0 | 2.3 |
|  | 13:09 | 75 | .0 | .0 | 2.4 |
|  | 13:10 | 6434 | 3.4 | 3.4 | 5.7 |
|  | 13:11 | 594 | .3 | .3 | 6.1 |
|  | 13:12 | 1231 | .6 | .6 | 6.7 |
|  | 13:13 | 791 | .4 | .4 | 7.1 |
|  | 13:14 | 280 | .1 | .1 | 7.3 |
|  | 13:15 | 1873 | 1.0 | 1.0 | 8.2 |
|  | 13:16 | 68 | .0 | .0 | 8.3 |
|  | 13:17 | 57 | .0 | .0 | 8.3 |
|  | 13:18 | 88 | .0 | .0 | 8.4 |
|  | 13:19 | 35 | .0 | .0 | 8.4 |
|  | 13:20 | 5629 | 3.0 | 3.0 | 11.3 |
|  | 13:21 | 69 | .0 | .0 | 11.4 |
|  | 13:22 | 130 | .1 | .1 | 11.4 |
|  | 13:23 | 53 | .0 | .0 | 11.5 |
|  | 13:24 | 46 | .0 | .0 | 11.5 |
|  | 13:25 | 1925 | 1.0 | 1.0 | 12.5 |
|  | 13:26 | 88 | .0 | .0 | 12.5 |
|  | 13:27 | 59 | .0 | .0 | 12.6 |
|  | 13:28 | 79 | .0 | .0 | 12.6 |
|  | 13:29 | 28 | .0 | .0 | 12.6 |
|  | 13:3 | 3 | .0 | .0 | 12.6 |
|  | 13:30 | 30532 | 16.0 | 16.0 | 28.7 |
|  | 13:31 | 39 | .0 | .0 | 28.7 |
|  | 13:32 | 82 | .0 | .0 | 28.7 |
|  | 13:33 | 90 | .0 | .0 | 28.8 |
|  | 13:34 | 47 | .0 | .0 | 28.8 |
|  | 13:35 | 1544 | .8 | .8 | 29.6 |
|  | 13:36 | 91 | .0 | .0 | 29.7 |
|  | 13:37 | 45 | .0 | .0 | 29.7 |
|  | 13:38 | 284 | .1 | .1 | 29.8 |
|  | 13:39 | 36 | .0 | .0 | 29.9 |
|  | 13:4 | 2 | .0 | .0 | 29.9 |
|  | 13:40 | 4912 | 2.6 | 2.6 | 32.5 |
|  | 13:41 | 40 | .0 | .0 | 32.5 |
|  | 13:42 | 83 | .0 | .0 | 32.5 |
|  | 13:43 | 45 | .0 | .0 | 32.5 |
|  | 13:44 | 53 | .0 | .0 | 32.6 |
|  | 13:45 | 1704 | .9 | .9 | 33.5 |
|  | 13:46 | 54 | .0 | .0 | 33.5 |
|  | 13:47 | 42 | .0 | .0 | 33.5 |
|  | 13:48 | 58 | .0 | .0 | 33.5 |
|  | 13:49 | 26 | .0 | .0 | 33.6 |
|  | 13:5 | 2 | .0 | .0 | 33.6 |
|  | 13:50 | 4381 | 2.3 | 2.3 | 35.9 |
|  | 13:51 | 28 | .0 | .0 | 35.9 |
|  | 13:52 | 72 | .0 | .0 | 35.9 |
|  | 13:53 | 35 | .0 | .0 | 35.9 |
|  | 13:54 | 37 | .0 | .0 | 36.0 |
|  | 13:55 | 1191 | .6 | .6 | 36.6 |
|  | 13:56 | 61 | .0 | .0 | 36.6 |
|  | 13:57 | 42 | .0 | .0 | 36.6 |
|  | 13:58 | 98 | .1 | .1 | 36.7 |
|  | 13:59 | 182 | .1 | .1 | 36.8 |
|  | 13:60 | 524 | .3 | .3 | 37.1 |
|  | 13:61 | 5 | .0 | .0 | 37.1 |
|  | 13:62 | 2 | .0 | .0 | 37.1 |
|  | 13:63 | 1 | .0 | .0 | 37.1 |
|  | 13:64 | 8 | .0 | .0 | 37.1 |
|  | 13:65 | 13 | .0 | .0 | 37.1 |
|  | 13:66 | 1 | .0 | .0 | 37.1 |
|  | 13:68 | 1 | .0 | .0 | 37.1 |
|  | 13:70 | 8 | .0 | .0 | 37.1 |
|  | 13:80 | 10 | .0 | .0 | 37.1 |
|  | 13:90 | 8 | .0 | .0 | 37.1 |
|  | 13:94 | 1 | .0 | .0 | 37.1 |
|  | 13:95 | 2 | .0 | .0 | 37.1 |
|  | 13:L1 | 2 | .0 | .0 | 37.1 |
|  | 13:L3 | 1 | .0 | .0 | 37.1 |
|  | 13?:2 | 2 | .0 | .0 | 37.1 |
|  | 13.00 | 26 | .0 | .0 | 37.1 |
|  | 13.03 | 1 | .0 | .0 | 37.1 |
|  | 13.05 | 4 | .0 | .0 | 37.1 |
|  | 13.06 | 1 | .0 | .0 | 37.1 |
|  | 13.08 | 1 | .0 | .0 | 37.1 |
|  | 13.10 | 36 | .0 | .0 | 37.1 |
|  | 13.11 | 2 | .0 | .0 | 37.1 |
|  | 13.13 | 2 | .0 | .0 | 37.1 |
|  | 13.15 | 15 | .0 | .0 | 37.1 |
|  | 13.19 | 1 | .0 | .0 | 37.1 |
|  | 13.20 | 45 | .0 | .0 | 37.2 |
|  | 13.22 | 1 | .0 | .0 | 37.2 |
|  | 13.25 | 11 | .0 | .0 | 37.2 |
|  | 13.26 | 1 | .0 | .0 | 37.2 |
|  | 13.30 | 58 | .0 | .0 | 37.2 |
|  | 13.35 | 6 | .0 | .0 | 37.2 |
|  | 13.40 | 40 | .0 | .0 | 37.2 |
|  | 13.45 | 7 | .0 | .0 | 37.2 |
|  | 13.50 | 41 | .0 | .0 | 37.2 |
|  | 13.53 | 1 | .0 | .0 | 37.2 |
|  | 13.54 | 1 | .0 | .0 | 37.2 |
|  | 13.55 | 14 | .0 | .0 | 37.3 |
|  | 13.59 | 2 | .0 | .0 | 37.3 |
|  | 13':2 | 1 | .0 | .0 | 37.3 |
|  | 130 | 27 | .0 | .0 | 37.3 |
|  | 1300 | 268 | .1 | .1 | 37.4 |
|  | 1302 | 3 | .0 | .0 | 37.4 |
|  | 1303 | 2 | .0 | .0 | 37.4 |
|  | 1304 | 4 | .0 | .0 | 37.4 |
|  | 1305 | 118 | .1 | .1 | 37.5 |
|  | 1306 | 6 | .0 | .0 | 37.5 |
|  | 1307 | 6 | .0 | .0 | 37.5 |
|  | 1308 | 5 | .0 | .0 | 37.5 |
|  | 1309 | 5 | .0 | .0 | 37.5 |
|  | 1310 | 580 | .3 | .3 | 37.8 |
|  | 1311 | 5 | .0 | .0 | 37.8 |
|  | 1312 | 9 | .0 | .0 | 37.8 |
|  | 1313 | 8 | .0 | .0 | 37.8 |
|  | 1314 | 9 | .0 | .0 | 37.8 |
|  | 1315 | 210 | .1 | .1 | 37.9 |
|  | 1316 | 8 | .0 | .0 | 37.9 |
|  | 1317 | 2 | .0 | .0 | 37.9 |
|  | 1318 | 10 | .0 | .0 | 37.9 |
|  | 1319 | 2 | .0 | .0 | 37.9 |
|  | 1320 | 707 | .4 | .4 | 38.3 |
|  | 1321 | 1 | .0 | .0 | 38.3 |
|  | 1322 | 8 | .0 | .0 | 38.3 |
|  | 13220 | 1 | .0 | .0 | 38.3 |
|  | 1323 | 5 | .0 | .0 | 38.3 |
|  | 1324 | 5 | .0 | .0 | 38.3 |
|  | 1325 | 188 | .1 | .1 | 38.4 |
|  | 1326 | 5 | .0 | .0 | 38.4 |
|  | 1327 | 4 | .0 | .0 | 38.4 |
|  | 1328 | 4 | .0 | .0 | 38.4 |
|  | 1329 | 8 | .0 | .0 | 38.4 |
|  | 1330 | 767 | .4 | .4 | 38.8 |
|  | 13300 | 1 | .0 | .0 | 38.8 |
|  | 1331 | 2 | .0 | .0 | 38.8 |
|  | 1332 | 17 | .0 | .0 | 38.8 |
|  | 1333 | 8 | .0 | .0 | 38.8 |
|  | 1334 | 6 | .0 | .0 | 38.8 |
|  | 1335 | 199 | .1 | .1 | 38.9 |
|  | 1336 | 7 | .0 | .0 | 39.0 |
|  | 1337 | 3 | .0 | .0 | 39.0 |
|  | 1338 | 4 | .0 | .0 | 39.0 |
|  | 1339 | 4 | .0 | .0 | 39.0 |
|  | 1340 | 746 | .4 | .4 | 39.3 |
|  | 13400 | 1 | .0 | .0 | 39.4 |
|  | 1341 | 4 | .0 | .0 | 39.4 |
|  | 1342 | 9 | .0 | .0 | 39.4 |
|  | 1343 | 2 | .0 | .0 | 39.4 |
|  | 1344 | 3 | .0 | .0 | 39.4 |
|  | 1345 | 205 | .1 | .1 | 39.5 |
|  | 1346 | 10 | .0 | .0 | 39.5 |
|  | 1347 | 5 | .0 | .0 | 39.5 |
|  | 1348 | 7 | .0 | .0 | 39.5 |
|  | 1349 | 4 | .0 | .0 | 39.5 |
|  | 135 | 8 | .0 | .0 | 39.5 |
|  | 1350 | 566 | .3 | .3 | 39.8 |
|  | 1351 | 2 | .0 | .0 | 39.8 |
|  | 1352 | 11 | .0 | .0 | 39.8 |
|  | 1353 | 1 | .0 | .0 | 39.8 |
|  | 1354 | 3 | .0 | .0 | 39.8 |
|  | 1355 | 152 | .1 | .1 | 39.9 |
|  | 1356 | 9 | .0 | .0 | 39.9 |
|  | 1357 | 5 | .0 | .0 | 39.9 |
|  | 1358 | 6 | .0 | .0 | 39.9 |
|  | 1359 | 9 | .0 | .0 | 39.9 |
|  | 136 | 2 | .0 | .0 | 39.9 |
|  | 136:3 | 1 | .0 | .0 | 39.9 |
|  | 1360 | 100 | .1 | .1 | 39.9 |
|  | 1365 | 2 | .0 | .0 | 39.9 |
|  | 1369 | 1 | .0 | .0 | 39.9 |
|  | 1370 | 2 | .0 | .0 | 39.9 |
|  | 1375 | 1 | .0 | .0 | 39.9 |
|  | 138 | 3 | .0 | .0 | 39.9 |
|  | 1380 | 1 | .0 | .0 | 39.9 |
|  | 1390 | 1 | .0 | .0 | 39.9 |
|  | 13L:1 | 1 | .0 | .0 | 39.9 |
|  | 13L50 | 1 | .0 | .0 | 39.9 |
|  | 14 | 9 | .0 | .0 | 40.0 |
|  | 14;'1 | 1 | .0 | .0 | 40.0 |
|  | 14;00 | 40 | .0 | .0 | 40.0 |
|  | 14;02 | 1 | .0 | .0 | 40.0 |
|  | 14;03 | 1 | .0 | .0 | 40.0 |
|  | 14;05 | 12 | .0 | .0 | 40.0 |
|  | 14;09 | 2 | .0 | .0 | 40.0 |
|  | 14;10 | 47 | .0 | .0 | 40.0 |
|  | 14;11 | 2 | .0 | .0 | 40.0 |
|  | 14;12 | 2 | .0 | .0 | 40.0 |
|  | 14;15 | 10 | .0 | .0 | 40.0 |
|  | 14;19 | 1 | .0 | .0 | 40.0 |
|  | 14;20 | 43 | .0 | .0 | 40.0 |
|  | 14;24 | 1 | .0 | .0 | 40.0 |
|  | 14;25 | 5 | .0 | .0 | 40.0 |
|  | 14;28 | 2 | .0 | .0 | 40.0 |
|  | 14;30 | 40 | .0 | .0 | 40.1 |
|  | 14;33 | 1 | .0 | .0 | 40.1 |
|  | 14;35 | 10 | .0 | .0 | 40.1 |
|  | 14;36 | 1 | .0 | .0 | 40.1 |
|  | 14;37 | 1 | .0 | .0 | 40.1 |
|  | 14;38 | 1 | .0 | .0 | 40.1 |
|  | 14;40 | 25 | .0 | .0 | 40.1 |
|  | 14;42 | 2 | .0 | .0 | 40.1 |
|  | 14;45 | 15 | .0 | .0 | 40.1 |
|  | 14;50 | 20 | .0 | .0 | 40.1 |
|  | 14;55 | 3 | .0 | .0 | 40.1 |
|  | 14;57 | 1 | .0 | .0 | 40.1 |
|  | 14;60 | 3 | .0 | .0 | 40.1 |
|  | 14;70 | 1 | .0 | .0 | 40.1 |
|  | 14:.1 | 2 | .0 | .0 | 40.1 |
|  | 14:'0 | 1 | .0 | .0 | 40.1 |
|  | 14:'3 | 1 | .0 | .0 | 40.1 |
|  | 14:0 | 5 | .0 | .0 | 40.1 |
|  | 14:00 | 5955 | 3.1 | 3.1 | 43.2 |
|  | 14:02 | 100 | .1 | .1 | 43.3 |
|  | 14:03 | 60 | .0 | .0 | 43.3 |
|  | 14:04 | 97 | .1 | .1 | 43.4 |
|  | 14:05 | 1415 | .7 | .7 | 44.1 |
|  | 14:06 | 50 | .0 | .0 | 44.1 |
|  | 14:07 | 64 | .0 | .0 | 44.2 |
|  | 14:08 | 76 | .0 | .0 | 44.2 |
|  | 14:09 | 64 | .0 | .0 | 44.3 |
|  | 14:10 | 6011 | 3.2 | 3.2 | 47.4 |
|  | 14:11 | 96 | .1 | .1 | 47.5 |
|  | 14:12 | 156 | .1 | .1 | 47.5 |
|  | 14:13 | 176 | .1 | .1 | 47.6 |
|  | 14:14 | 92 | .0 | .0 | 47.7 |
|  | 14:15 | 2299 | 1.2 | 1.2 | 48.9 |
|  | 14:16 | 91 | .0 | .0 | 48.9 |
|  | 14:17 | 85 | .0 | .0 | 49.0 |
|  | 14:18 | 87 | .0 | .0 | 49.0 |
|  | 14:19 | 52 | .0 | .0 | 49.1 |
|  | 14:20 | 6626 | 3.5 | 3.5 | 52.5 |
|  | 14:21 | 61 | .0 | .0 | 52.6 |
|  | 14:22 | 152 | .1 | .1 | 52.7 |
|  | 14:23 | 64 | .0 | .0 | 52.7 |
|  | 14:24 | 35 | .0 | .0 | 52.7 |
|  | 14:25 | 1998 | 1.0 | 1.0 | 53.8 |
|  | 14:26 | 77 | .0 | .0 | 53.8 |
|  | 14:27 | 50 | .0 | .0 | 53.8 |
|  | 14:28 | 92 | .0 | .0 | 53.9 |
|  | 14:29 | 41 | .0 | .0 | 53.9 |
|  | 14:3 | 1 | .0 | .0 | 53.9 |
|  | 14:3. | 1 | .0 | .0 | 53.9 |
|  | 14:30 | 6442 | 3.4 | 3.4 | 57.3 |
|  | 14:31 | 35 | .0 | .0 | 57.3 |
|  | 14:32 | 108 | .1 | .1 | 57.4 |
|  | 14:33 | 71 | .0 | .0 | 57.4 |
|  | 14:34 | 39 | .0 | .0 | 57.4 |
|  | 14:35 | 1519 | .8 | .8 | 58.2 |
|  | 14:36 | 77 | .0 | .0 | 58.3 |
|  | 14:37 | 39 | .0 | .0 | 58.3 |
|  | 14:38 | 112 | .1 | .1 | 58.3 |
|  | 14:39 | 38 | .0 | .0 | 58.3 |
|  | 14:4 | 2 | .0 | .0 | 58.4 |
|  | 14:40 | 5154 | 2.7 | 2.7 | 61.1 |
|  | 14:41 | 52 | .0 | .0 | 61.1 |
|  | 14:42 | 84 | .0 | .0 | 61.1 |
|  | 14:43 | 58 | .0 | .0 | 61.2 |
|  | 14:44 | 46 | .0 | .0 | 61.2 |
|  | 14:45 | 1643 | .9 | .9 | 62.0 |
|  | 14:46 | 39 | .0 | .0 | 62.1 |
|  | 14:47 | 39 | .0 | .0 | 62.1 |
|  | 14:48 | 57 | .0 | .0 | 62.1 |
|  | 14:49 | 37 | .0 | .0 | 62.1 |
|  | 14:5 | 5 | .0 | .0 | 62.1 |
|  | 14:50 | 4623 | 2.4 | 2.4 | 64.6 |
|  | 14:51 | 28 | .0 | .0 | 64.6 |
|  | 14:52 | 77 | .0 | .0 | 64.6 |
|  | 14:53 | 42 | .0 | .0 | 64.6 |
|  | 14:54 | 35 | .0 | .0 | 64.7 |
|  | 14:55 | 1028 | .5 | .5 | 65.2 |
|  | 14:56 | 76 | .0 | .0 | 65.2 |
|  | 14:57 | 54 | .0 | .0 | 65.3 |
|  | 14:58 | 101 | .1 | .1 | 65.3 |
|  | 14:59 | 159 | .1 | .1 | 65.4 |
|  | 14:6 | 1 | .0 | .0 | 65.4 |
|  | 14:60 | 486 | .3 | .3 | 65.7 |
|  | 14:62 | 1 | .0 | .0 | 65.7 |
|  | 14:63 | 3 | .0 | .0 | 65.7 |
|  | 14:64 | 1 | .0 | .0 | 65.7 |
|  | 14:65 | 6 | .0 | .0 | 65.7 |
|  | 14:66 | 1 | .0 | .0 | 65.7 |
|  | 14:69 | 1 | .0 | .0 | 65.7 |
|  | 14:70 | 7 | .0 | .0 | 65.7 |
|  | 14:71 | 2 | .0 | .0 | 65.7 |
|  | 14:72 | 9 | .0 | .0 | 65.7 |
|  | 14:73 | 4 | .0 | .0 | 65.7 |
|  | 14:74 | 4 | .0 | .0 | 65.7 |
|  | 14:75 | 5 | .0 | .0 | 65.7 |
|  | 14:8 | 1 | .0 | .0 | 65.7 |
|  | 14:80 | 3 | .0 | .0 | 65.7 |
|  | 14:85 | 1 | .0 | .0 | 65.7 |
|  | 14:90 | 8 | .0 | .0 | 65.7 |
|  | 14:L0 | 2 | .0 | .0 | 65.7 |
|  | 14:L2 | 1 | .0 | .0 | 65.7 |
|  | 14:L3 | 1 | .0 | .0 | 65.7 |
|  | 14?:1 | 1 | .0 | .0 | 65.7 |
|  | 14?:3 | 1 | .0 | .0 | 65.7 |
|  | 14.:2 | 1 | .0 | .0 | 65.7 |
|  | 14.00 | 49 | .0 | .0 | 65.7 |
|  | 14.03 | 1 | .0 | .0 | 65.7 |
|  | 14.04 | 1 | .0 | .0 | 65.7 |
|  | 14.05 | 14 | .0 | .0 | 65.7 |
|  | 14.06 | 1 | .0 | .0 | 65.7 |
|  | 14.07 | 1 | .0 | .0 | 65.7 |
|  | 14.08 | 1 | .0 | .0 | 65.7 |
|  | 14.10 | 42 | .0 | .0 | 65.8 |
|  | 14.11 | 1 | .0 | .0 | 65.8 |
|  | 14.13 | 2 | .0 | .0 | 65.8 |
|  | 14.15 | 18 | .0 | .0 | 65.8 |
|  | 14.16 | 2 | .0 | .0 | 65.8 |
|  | 14.17 | 3 | .0 | .0 | 65.8 |
|  | 14.20 | 54 | .0 | .0 | 65.8 |
|  | 14.23 | 2 | .0 | .0 | 65.8 |
|  | 14.25 | 18 | .0 | .0 | 65.8 |
|  | 14.27 | 1 | .0 | .0 | 65.8 |
|  | 14.28 | 1 | .0 | .0 | 65.8 |
|  | 14.30 | 51 | .0 | .0 | 65.8 |
|  | 14.33 | 1 | .0 | .0 | 65.8 |
|  | 14.34 | 2 | .0 | .0 | 65.8 |
|  | 14.35 | 12 | .0 | .0 | 65.8 |
|  | 14.40 | 40 | .0 | .0 | 65.9 |
|  | 14.41 | 1 | .0 | .0 | 65.9 |
|  | 14.45 | 13 | .0 | .0 | 65.9 |
|  | 14.47 | 1 | .0 | .0 | 65.9 |
|  | 14.50 | 27 | .0 | .0 | 65.9 |
|  | 14.52 | 1 | .0 | .0 | 65.9 |
|  | 14.55 | 13 | .0 | .0 | 65.9 |
|  | 14.58 | 1 | .0 | .0 | 65.9 |
|  | 14.59 | 1 | .0 | .0 | 65.9 |
|  | 14.60 | 1 | .0 | .0 | 65.9 |
|  | 14"25 | 1 | .0 | .0 | 65.9 |
|  | 14"35 | 1 | .0 | .0 | 65.9 |
|  | 14"40 | 1 | .0 | .0 | 65.9 |
|  | 140 | 26 | .0 | .0 | 65.9 |
|  | 1400 | 572 | .3 | .3 | 66.2 |
|  | 1402 | 25 | .0 | .0 | 66.2 |
|  | 1403 | 11 | .0 | .0 | 66.2 |
|  | 1404 | 1 | .0 | .0 | 66.2 |
|  | 1405 | 142 | .1 | .1 | 66.3 |
|  | 1406 | 6 | .0 | .0 | 66.3 |
|  | 1407 | 2 | .0 | .0 | 66.3 |
|  | 1408 | 11 | .0 | .0 | 66.3 |
|  | 1409 | 11 | .0 | .0 | 66.3 |
|  | 141:1 | 1 | .0 | .0 | 66.3 |
|  | 1410 | 711 | .4 | .4 | 66.7 |
|  | 1411 | 12 | .0 | .0 | 66.7 |
|  | 14113 | 1 | .0 | .0 | 66.7 |
|  | 1412 | 13 | .0 | .0 | 66.7 |
|  | 1413 | 7 | .0 | .0 | 66.7 |
|  | 1414 | 2 | .0 | .0 | 66.7 |
|  | 1415 | 227 | .1 | .1 | 66.8 |
|  | 1416 | 9 | .0 | .0 | 66.8 |
|  | 1417 | 3 | .0 | .0 | 66.8 |
|  | 1418 | 8 | .0 | .0 | 66.8 |
|  | 1419 | 2 | .0 | .0 | 66.8 |
|  | 142 | 2 | .0 | .0 | 66.8 |
|  | 1420 | 742 | .4 | .4 | 67.2 |
|  | 14200 | 1 | .0 | .0 | 67.2 |
|  | 14202 | 1 | .0 | .0 | 67.2 |
|  | 1421 | 7 | .0 | .0 | 67.2 |
|  | 1422 | 14 | .0 | .0 | 67.3 |
|  | 1423 | 7 | .0 | .0 | 67.3 |
|  | 1424 | 1 | .0 | .0 | 67.3 |
|  | 1425 | 172 | .1 | .1 | 67.3 |
|  | 1426 | 14 | .0 | .0 | 67.4 |
|  | 1427 | 1 | .0 | .0 | 67.4 |
|  | 1428 | 13 | .0 | .0 | 67.4 |
|  | 1429 | 4 | .0 | .0 | 67.4 |
|  | 143 | 1 | .0 | .0 | 67.4 |
|  | 143:2 | 1 | .0 | .0 | 67.4 |
|  | 1430 | 637 | .3 | .3 | 67.7 |
|  | 1431 | 2 | .0 | .0 | 67.7 |
|  | 1432 | 19 | .0 | .0 | 67.7 |
|  | 1433 | 7 | .0 | .0 | 67.7 |
|  | 1434 | 3 | .0 | .0 | 67.7 |
|  | 1435 | 147 | .1 | .1 | 67.8 |
|  | 1436 | 12 | .0 | .0 | 67.8 |
|  | 1437 | 3 | .0 | .0 | 67.8 |
|  | 1438 | 7 | .0 | .0 | 67.8 |
|  | 1439 | 4 | .0 | .0 | 67.8 |
|  | 1440 | 542 | .3 | .3 | 68.1 |
|  | 1441 | 1 | .0 | .0 | 68.1 |
|  | 1442 | 13 | .0 | .0 | 68.1 |
|  | 1443 | 3 | .0 | .0 | 68.1 |
|  | 1444 | 6 | .0 | .0 | 68.1 |
|  | 1445 | 146 | .1 | .1 | 68.2 |
|  | 1446 | 6 | .0 | .0 | 68.2 |
|  | 1447 | 7 | .0 | .0 | 68.2 |
|  | 1448 | 6 | .0 | .0 | 68.2 |
|  | 1449 | 2 | .0 | .0 | 68.2 |
|  | 145 | 9 | .0 | .0 | 68.2 |
|  | 145: | 1 | .0 | .0 | 68.2 |
|  | 145:2 | 1 | .0 | .0 | 68.2 |
|  | 1450 | 486 | .3 | .3 | 68.5 |
|  | 1451 | 6 | .0 | .0 | 68.5 |
|  | 1452 | 10 | .0 | .0 | 68.5 |
|  | 1453 | 4 | .0 | .0 | 68.5 |
|  | 1454 | 3 | .0 | .0 | 68.5 |
|  | 1455 | 85 | .0 | .0 | 68.5 |
|  | 1456 | 5 | .0 | .0 | 68.5 |
|  | 1457 | 6 | .0 | .0 | 68.5 |
|  | 1458 | 5 | .0 | .0 | 68.5 |
|  | 1459 | 15 | .0 | .0 | 68.5 |
|  | 1460 | 92 | .0 | .0 | 68.6 |
|  | 147:0 | 1 | .0 | .0 | 68.6 |
|  | 147:3 | 1 | .0 | .0 | 68.6 |
|  | 1470 | 1 | .0 | .0 | 68.6 |
|  | 1473 | 1 | .0 | .0 | 68.6 |
|  | 1475 | 1 | .0 | .0 | 68.6 |
|  | 148 | 1 | .0 | .0 | 68.6 |
|  | 14L:1 | 1 | .0 | .0 | 68.6 |
|  | 14L:2 | 1 | .0 | .0 | 68.6 |
|  | 15 | 8 | .0 | .0 | 68.6 |
|  | 15;'0 | 2 | .0 | .0 | 68.6 |
|  | 15;'1 | 2 | .0 | .0 | 68.6 |
|  | 15;00 | 31 | .0 | .0 | 68.6 |
|  | 15;05 | 10 | .0 | .0 | 68.6 |
|  | 15;06 | 1 | .0 | .0 | 68.6 |
|  | 15;10 | 12 | .0 | .0 | 68.6 |
|  | 15;15 | 9 | .0 | .0 | 68.6 |
|  | 15;16 | 1 | .0 | .0 | 68.6 |
|  | 15;20 | 20 | .0 | .0 | 68.6 |
|  | 15;24 | 2 | .0 | .0 | 68.6 |
|  | 15;25 | 5 | .0 | .0 | 68.6 |
|  | 15;3 | 1 | .0 | .0 | 68.6 |
|  | 15;30 | 20 | .0 | .0 | 68.6 |
|  | 15;31 | 1 | .0 | .0 | 68.6 |
|  | 15;35 | 6 | .0 | .0 | 68.6 |
|  | 15;38 | 1 | .0 | .0 | 68.6 |
|  | 15;40 | 12 | .0 | .0 | 68.7 |
|  | 15;45 | 4 | .0 | .0 | 68.7 |
|  | 15;50 | 19 | .0 | .0 | 68.7 |
|  | 15;55 | 1 | .0 | .0 | 68.7 |
|  | 15;56 | 1 | .0 | .0 | 68.7 |
|  | 15::3 | 1 | .0 | .0 | 68.7 |
|  | 15:0 | 4 | .0 | .0 | 68.7 |
|  | 15:00 | 4620 | 2.4 | 2.4 | 71.1 |
|  | 15:02 | 72 | .0 | .0 | 71.1 |
|  | 15:03 | 30 | .0 | .0 | 71.2 |
|  | 15:04 | 37 | .0 | .0 | 71.2 |
|  | 15:05 | 1059 | .6 | .6 | 71.7 |
|  | 15:06 | 35 | .0 | .0 | 71.7 |
|  | 15:07 | 48 | .0 | .0 | 71.8 |
|  | 15:08 | 65 | .0 | .0 | 71.8 |
|  | 15:09 | 35 | .0 | .0 | 71.8 |
|  | 15:1 | 1 | .0 | .0 | 71.8 |
|  | 15:10 | 4053 | 2.1 | 2.1 | 74.0 |
|  | 15:11 | 39 | .0 | .0 | 74.0 |
|  | 15:12 | 84 | .0 | .0 | 74.0 |
|  | 15:13 | 118 | .1 | .1 | 74.1 |
|  | 15:14 | 67 | .0 | .0 | 74.1 |
|  | 15:15 | 1280 | .7 | .7 | 74.8 |
|  | 15:16 | 74 | .0 | .0 | 74.8 |
|  | 15:17 | 44 | .0 | .0 | 74.8 |
|  | 15:18 | 217 | .1 | .1 | 75.0 |
|  | 15:19 | 22 | .0 | .0 | 75.0 |
|  | 15:2 | 1 | .0 | .0 | 75.0 |
|  | 15:20 | 4239 | 2.2 | 2.2 | 77.2 |
|  | 15:21 | 51 | .0 | .0 | 77.2 |
|  | 15:22 | 80 | .0 | .0 | 77.3 |
|  | 15:23 | 32 | .0 | .0 | 77.3 |
|  | 15:24 | 28 | .0 | .0 | 77.3 |
|  | 15:26 | 49 | .0 | .0 | 77.3 |
|  | 15:27 | 41 | .0 | .0 | 77.4 |
|  | 15:28 | 52 | .0 | .0 | 77.4 |
|  | 15:29 | 28 | .0 | .0 | 77.4 |
|  | 15:3 | 3 | .0 | .0 | 77.4 |
|  | 15:30 | 5765 | 3.0 | 3.0 | 80.4 |
|  | 15:31 | 25 | .0 | .0 | 80.4 |
|  | 15:32 | 73 | .0 | .0 | 80.5 |
|  | 15:33 | 48 | .0 | .0 | 80.5 |
|  | 15:34 | 27 | .0 | .0 | 80.5 |
|  | 15:35 | 1008 | .5 | .5 | 81.0 |
|  | 15:36 | 59 | .0 | .0 | 81.1 |
|  | 15:37 | 40 | .0 | .0 | 81.1 |
|  | 15:38 | 71 | .0 | .0 | 81.1 |
|  | 15:39 | 17 | .0 | .0 | 81.1 |
|  | 15:4 | 1 | .0 | .0 | 81.1 |
|  | 15:40 | 3147 | 1.7 | 1.7 | 82.8 |
|  | 15:41 | 28 | .0 | .0 | 82.8 |
|  | 15:42 | 75 | .0 | .0 | 82.9 |
|  | 15:43 | 28 | .0 | .0 | 82.9 |
|  | 15:44 | 27 | .0 | .0 | 82.9 |
|  | 15:45 | 1029 | .5 | .5 | 83.4 |
|  | 15:46 | 35 | .0 | .0 | 83.4 |
|  | 15:47 | 47 | .0 | .0 | 83.5 |
|  | 15:48 | 49 | .0 | .0 | 83.5 |
|  | 15:49 | 27 | .0 | .0 | 83.5 |
|  | 15:5 | 1 | .0 | .0 | 83.5 |
|  | 15:50 | 2864 | 1.5 | 1.5 | 85.0 |
|  | 15:51 | 22 | .0 | .0 | 85.0 |
|  | 15:52 | 41 | .0 | .0 | 85.0 |
|  | 15:53 | 39 | .0 | .0 | 85.1 |
|  | 15:54 | 20 | .0 | .0 | 85.1 |
|  | 15:55 | 538 | .3 | .3 | 85.4 |
|  | 15:56 | 47 | .0 | .0 | 85.4 |
|  | 15:57 | 36 | .0 | .0 | 85.4 |
|  | 15:58 | 57 | .0 | .0 | 85.4 |
|  | 15:59 | 86 | .0 | .0 | 85.5 |
|  | 15:60 | 274 | .1 | .1 | 85.6 |
|  | 15:61 | 5 | .0 | .0 | 85.6 |
|  | 15:62 | 4 | .0 | .0 | 85.6 |
|  | 15:64 | 1 | .0 | .0 | 85.6 |
|  | 15:65 | 1 | .0 | .0 | 85.6 |
|  | 15:66 | 2 | .0 | .0 | 85.6 |
|  | 15:70 | 4 | .0 | .0 | 85.6 |
|  | 15:74 | 1 | .0 | .0 | 85.6 |
|  | 15:75 | 1 | .0 | .0 | 85.6 |
|  | 15:8 | 2 | .0 | .0 | 85.6 |
|  | 15:80 | 6 | .0 | .0 | 85.6 |
|  | 15:81 | 2 | .0 | .0 | 85.6 |
|  | 15:82 | 4 | .0 | .0 | 85.6 |
|  | 15:84 | 2 | .0 | .0 | 85.6 |
|  | 15:85 | 2 | .0 | .0 | 85.6 |
|  | 15:90 | 6 | .0 | .0 | 85.6 |
|  | 15:L0 | 3 | .0 | .0 | 85.6 |
|  | 15:L1 | 1 | .0 | .0 | 85.6 |
|  | 15:L2 | 1 | .0 | .0 | 85.6 |
|  | 15?:4 | 1 | .0 | .0 | 85.6 |
|  | 15.00 | 30 | .0 | .0 | 85.7 |
|  | 15.04 | 1 | .0 | .0 | 85.7 |
|  | 15.05 | 11 | .0 | .0 | 85.7 |
|  | 15.09 | 1 | .0 | .0 | 85.7 |
|  | 15.10 | 21 | .0 | .0 | 85.7 |
|  | 15.12 | 2 | .0 | .0 | 85.7 |
|  | 15.13 | 2 | .0 | .0 | 85.7 |
|  | 15.14 | 1 | .0 | .0 | 85.7 |
|  | 15.15 | 8 | .0 | .0 | 85.7 |
|  | 15.16 | 1 | .0 | .0 | 85.7 |
|  | 15.20 | 35 | .0 | .0 | 85.7 |
|  | 15.22 | 2 | .0 | .0 | 85.7 |
|  | 15.24 | 2 | .0 | .0 | 85.7 |
|  | 15.25 | 3 | .0 | .0 | 85.7 |
|  | 15.26 | 1 | .0 | .0 | 85.7 |
|  | 15.27 | 1 | .0 | .0 | 85.7 |
|  | 15.30 | 41 | .0 | .0 | 85.7 |
|  | 15.32 | 1 | .0 | .0 | 85.7 |
|  | 15.33 | 2 | .0 | .0 | 85.7 |
|  | 15.34 | 1 | .0 | .0 | 85.7 |
|  | 15.35 | 4 | .0 | .0 | 85.7 |
|  | 15.36 | 1 | .0 | .0 | 85.7 |
|  | 15.38 | 1 | .0 | .0 | 85.7 |
|  | 15.40 | 21 | .0 | .0 | 85.7 |
|  | 15.43 | 1 | .0 | .0 | 85.7 |
|  | 15.45 | 7 | .0 | .0 | 85.8 |
|  | 15.48 | 1 | .0 | .0 | 85.8 |
|  | 15.49 | 1 | .0 | .0 | 85.8 |
|  | 15.50 | 9 | .0 | .0 | 85.8 |
|  | 15.51 | 1 | .0 | .0 | 85.8 |
|  | 15.53 | 1 | .0 | .0 | 85.8 |
|  | 15.55 | 8 | .0 | .0 | 85.8 |
|  | 15.59 | 1 | .0 | .0 | 85.8 |
|  | 15"18 | 1 | .0 | .0 | 85.8 |
|  | 15"20 | 1 | .0 | .0 | 85.8 |
|  | 15"30 | 2 | .0 | .0 | 85.8 |
|  | 15"40 | 1 | .0 | .0 | 85.8 |
|  | 15"50 | 2 | .0 | .0 | 85.8 |
|  | 15"56 | 1 | .0 | .0 | 85.8 |
|  | 150 | 19 | .0 | .0 | 85.8 |
|  | 1500 | 446 | .2 | .2 | 86.0 |
|  | 1502 | 17 | .0 | .0 | 86.0 |
|  | 1503 | 11 | .0 | .0 | 86.0 |
|  | 1504 | 5 | .0 | .0 | 86.0 |
|  | 1505 | 87 | .0 | .0 | 86.1 |
|  | 1506 | 4 | .0 | .0 | 86.1 |
|  | 1507 | 5 | .0 | .0 | 86.1 |
|  | 1508 | 11 | .0 | .0 | 86.1 |
|  | 1509 | 4 | .0 | .0 | 86.1 |
|  | 1510 | 445 | .2 | .2 | 86.3 |
|  | 1511 | 3 | .0 | .0 | 86.3 |
|  | 1512 | 6 | .0 | .0 | 86.3 |
|  | 1513 | 13 | .0 | .0 | 86.3 |
|  | 1514 | 3 | .0 | .0 | 86.3 |
|  | 1515 | 99 | .1 | .1 | 86.4 |
|  | 1516 | 16 | .0 | .0 | 86.4 |
|  | 1517 | 8 | .0 | .0 | 86.4 |
|  | 1518 | 8 | .0 | .0 | 86.4 |
|  | 1519 | 4 | .0 | .0 | 86.4 |
|  | 1520 | 461 | .2 | .2 | 86.6 |
|  | 1521 | 6 | .0 | .0 | 86.7 |
|  | 1522 | 10 | .0 | .0 | 86.7 |
|  | 1523 | 2 | .0 | .0 | 86.7 |
|  | 1524 | 8 | .0 | .0 | 86.7 |
|  | 1525 | 89 | .0 | .0 | 86.7 |
|  | 1526 | 6 | .0 | .0 | 86.7 |
|  | 1527 | 4 | .0 | .0 | 86.7 |
|  | 1528 | 8 | .0 | .0 | 86.7 |
|  | 1529 | 1 | .0 | .0 | 86.7 |
|  | 1530 | 451 | .2 | .2 | 87.0 |
|  | 1531 | 4 | .0 | .0 | 87.0 |
|  | 1532 | 11 | .0 | .0 | 87.0 |
|  | 1533 | 5 | .0 | .0 | 87.0 |
|  | 1534 | 5 | .0 | .0 | 87.0 |
|  | 1535 | 66 | .0 | .0 | 87.0 |
|  | 1536 | 7 | .0 | .0 | 87.0 |
|  | 1537 | 3 | .0 | .0 | 87.0 |
|  | 1538 | 9 | .0 | .0 | 87.0 |
|  | 1539 | 6 | .0 | .0 | 87.0 |
|  | 154 | 1 | .0 | .0 | 87.0 |
|  | 1540 | 322 | .2 | .2 | 87.2 |
|  | 1542 | 8 | .0 | .0 | 87.2 |
|  | 1543 | 4 | .0 | .0 | 87.2 |
|  | 1545 | 74 | .0 | .0 | 87.2 |
|  | 1546 | 3 | .0 | .0 | 87.2 |
|  | 1547 | 8 | .0 | .0 | 87.2 |
|  | 1548 | 4 | .0 | .0 | 87.2 |
|  | 1549 | 3 | .0 | .0 | 87.2 |
|  | 155 | 3 | .0 | .0 | 87.2 |
|  | 1550 | 241 | .1 | .1 | 87.4 |
|  | 1551 | 1 | .0 | .0 | 87.4 |
|  | 1552 | 4 | .0 | .0 | 87.4 |
|  | 1553 | 3 | .0 | .0 | 87.4 |
|  | 1555 | 61 | .0 | .0 | 87.4 |
|  | 15550 | 1 | .0 | .0 | 87.4 |
|  | 1556 | 5 | .0 | .0 | 87.4 |
|  | 1557 | 4 | .0 | .0 | 87.4 |
|  | 1558 | 5 | .0 | .0 | 87.4 |
|  | 1559 | 10 | .0 | .0 | 87.4 |
|  | 156 | 1 | .0 | .0 | 87.4 |
|  | 156:3 | 1 | .0 | .0 | 87.4 |
|  | 1560 | 35 | .0 | .0 | 87.4 |
|  | 1564 | 1 | .0 | .0 | 87.4 |
|  | 158:3 | 1 | .0 | .0 | 87.4 |
|  | 158:4 | 1 | .0 | .0 | 87.4 |
|  | 16 | 2 | .0 | .0 | 87.4 |
|  | 16;00 | 9 | .0 | .0 | 87.4 |
|  | 16;05 | 4 | .0 | .0 | 87.4 |
|  | 16;10 | 17 | .0 | .0 | 87.5 |
|  | 16;12 | 2 | .0 | .0 | 87.5 |
|  | 16;15 | 1 | .0 | .0 | 87.5 |
|  | 16;16 | 1 | .0 | .0 | 87.5 |
|  | 16;20 | 13 | .0 | .0 | 87.5 |
|  | 16;25 | 3 | .0 | .0 | 87.5 |
|  | 16;30 | 11 | .0 | .0 | 87.5 |
|  | 16;35 | 3 | .0 | .0 | 87.5 |
|  | 16;40 | 7 | .0 | .0 | 87.5 |
|  | 16;45 | 1 | .0 | .0 | 87.5 |
|  | 16;50 | 3 | .0 | .0 | 87.5 |
|  | 16:0 | 1 | .0 | .0 | 87.5 |
|  | 16:00 | 2406 | 1.3 | 1.3 | 88.7 |
|  | 16:02 | 50 | .0 | .0 | 88.8 |
|  | 16:03 | 25 | .0 | .0 | 88.8 |
|  | 16:04 | 10 | .0 | .0 | 88.8 |
|  | 16:05 | 463 | .2 | .2 | 89.0 |
|  | 16:06 | 28 | .0 | .0 | 89.0 |
|  | 16:07 | 18 | .0 | .0 | 89.1 |
|  | 16:08 | 26 | .0 | .0 | 89.1 |
|  | 16:09 | 31 | .0 | .0 | 89.1 |
|  | 16:10 | 1983 | 1.0 | 1.0 | 90.1 |
|  | 16:11 | 30 | .0 | .0 | 90.1 |
|  | 16:12 | 50 | .0 | .0 | 90.2 |
|  | 16:13 | 94 | .0 | .0 | 90.2 |
|  | 16:14 | 42 | .0 | .0 | 90.2 |
|  | 16:15 | 631 | .3 | .3 | 90.6 |
|  | 16:16 | 24 | .0 | .0 | 90.6 |
|  | 16:17 | 32 | .0 | .0 | 90.6 |
|  | 16:18 | 46 | .0 | .0 | 90.6 |
|  | 16:19 | 15 | .0 | .0 | 90.6 |
|  | 16:20 | 2045 | 1.1 | 1.1 | 91.7 |
|  | 16:21 | 30 | .0 | .0 | 91.7 |
|  | 16:22 | 51 | .0 | .0 | 91.8 |
|  | 16:23 | 21 | .0 | .0 | 91.8 |
|  | 16:24 | 20 | .0 | .0 | 91.8 |
|  | 16:25 | 500 | .3 | .3 | 92.0 |
|  | 16:26 | 34 | .0 | .0 | 92.1 |
|  | 16:27 | 20 | .0 | .0 | 92.1 |
|  | 16:28 | 35 | .0 | .0 | 92.1 |
|  | 16:29 | 14 | .0 | .0 | 92.1 |
|  | 16:30 | 1709 | .9 | .9 | 93.0 |
|  | 16:31 | 19 | .0 | .0 | 93.0 |
|  | 16:32 | 46 | .0 | .0 | 93.0 |
|  | 16:33 | 24 | .0 | .0 | 93.0 |
|  | 16:34 | 24 | .0 | .0 | 93.0 |
|  | 16:35 | 399 | .2 | .2 | 93.3 |
|  | 16:36 | 40 | .0 | .0 | 93.3 |
|  | 16:37 | 23 | .0 | .0 | 93.3 |
|  | 16:38 | 39 | .0 | .0 | 93.3 |
|  | 16:39 | 19 | .0 | .0 | 93.3 |
|  | 16:40 | 1239 | .7 | .7 | 94.0 |
|  | 16:41 | 17 | .0 | .0 | 94.0 |
|  | 16:42 | 33 | .0 | .0 | 94.0 |
|  | 16:43 | 17 | .0 | .0 | 94.0 |
|  | 16:44 | 26 | .0 | .0 | 94.0 |
|  | 16:45 | 443 | .2 | .2 | 94.3 |
|  | 16:46 | 12 | .0 | .0 | 94.3 |
|  | 16:47 | 21 | .0 | .0 | 94.3 |
|  | 16:48 | 25 | .0 | .0 | 94.3 |
|  | 16:49 | 17 | .0 | .0 | 94.3 |
|  | 16:50 | 986 | .5 | .5 | 94.8 |
|  | 16:51 | 19 | .0 | .0 | 94.8 |
|  | 16:52 | 22 | .0 | .0 | 94.8 |
|  | 16:53 | 20 | .0 | .0 | 94.8 |
|  | 16:54 | 12 | .0 | .0 | 94.8 |
|  | 16:55 | 288 | .2 | .2 | 95.0 |
|  | 16:56 | 21 | .0 | .0 | 95.0 |
|  | 16:57 | 11 | .0 | .0 | 95.0 |
|  | 16:58 | 28 | .0 | .0 | 95.0 |
|  | 16:59 | 47 | .0 | .0 | 95.1 |
|  | 16:60 | 88 | .0 | .0 | 95.1 |
|  | 16:62 | 2 | .0 | .0 | 95.1 |
|  | 16:69 | 1 | .0 | .0 | 95.1 |
|  | 16:70 | 2 | .0 | .0 | 95.1 |
|  | 16:90 | 1 | .0 | .0 | 95.1 |
|  | 16:92 | 3 | .0 | .0 | 95.1 |
|  | 16:94 | 1 | .0 | .0 | 95.1 |
|  | 16?:2 | 1 | .0 | .0 | 95.1 |
|  | 16.00 | 9 | .0 | .0 | 95.1 |
|  | 16.02 | 1 | .0 | .0 | 95.1 |
|  | 16.03 | 1 | .0 | .0 | 95.1 |
|  | 16.05 | 2 | .0 | .0 | 95.1 |
|  | 16.06 | 2 | .0 | .0 | 95.1 |
|  | 16.08 | 1 | .0 | .0 | 95.1 |
|  | 16.09 | 1 | .0 | .0 | 95.1 |
|  | 16.10 | 11 | .0 | .0 | 95.1 |
|  | 16.12 | 3 | .0 | .0 | 95.1 |
|  | 16.15 | 6 | .0 | .0 | 95.1 |
|  | 16.16 | 1 | .0 | .0 | 95.1 |
|  | 16.17 | 1 | .0 | .0 | 95.1 |
|  | 16.18 | 1 | .0 | .0 | 95.1 |
|  | 16.20 | 13 | .0 | .0 | 95.1 |
|  | 16.22 | 2 | .0 | .0 | 95.1 |
|  | 16.24 | 1 | .0 | .0 | 95.1 |
|  | 16.25 | 7 | .0 | .0 | 95.1 |
|  | 16.27 | 1 | .0 | .0 | 95.1 |
|  | 16.28 | 1 | .0 | .0 | 95.1 |
|  | 16.30 | 10 | .0 | .0 | 95.1 |
|  | 16.34 | 1 | .0 | .0 | 95.1 |
|  | 16.35 | 3 | .0 | .0 | 95.2 |
|  | 16.38 | 1 | .0 | .0 | 95.2 |
|  | 16.40 | 5 | .0 | .0 | 95.2 |
|  | 16.41 | 1 | .0 | .0 | 95.2 |
|  | 16.42 | 1 | .0 | .0 | 95.2 |
|  | 16.45 | 1 | .0 | .0 | 95.2 |
|  | 16.49 | 1 | .0 | .0 | 95.2 |
|  | 16.50 | 6 | .0 | .0 | 95.2 |
|  | 16.55 | 2 | .0 | .0 | 95.2 |
|  | 16.58 | 1 | .0 | .0 | 95.2 |
|  | 160 | 2 | .0 | .0 | 95.2 |
|  | 1600 | 179 | .1 | .1 | 95.3 |
|  | 1602 | 9 | .0 | .0 | 95.3 |
|  | 1603 | 4 | .0 | .0 | 95.3 |
|  | 1605 | 29 | .0 | .0 | 95.3 |
|  | 1606 | 3 | .0 | .0 | 95.3 |
|  | 1607 | 4 | .0 | .0 | 95.3 |
|  | 1608 | 2 | .0 | .0 | 95.3 |
|  | 1609 | 5 | .0 | .0 | 95.3 |
|  | 1610 | 158 | .1 | .1 | 95.4 |
|  | 1611 | 3 | .0 | .0 | 95.4 |
|  | 1612 | 3 | .0 | .0 | 95.4 |
|  | 1613 | 5 | .0 | .0 | 95.4 |
|  | 1614 | 1 | .0 | .0 | 95.4 |
|  | 1615 | 44 | .0 | .0 | 95.4 |
|  | 1618 | 3 | .0 | .0 | 95.4 |
|  | 162 | 1 | .0 | .0 | 95.4 |
|  | 1620 | 147 | .1 | .1 | 95.5 |
|  | 1621 | 2 | .0 | .0 | 95.5 |
|  | 1622 | 1 | .0 | .0 | 95.5 |
|  | 1624 | 1 | .0 | .0 | 95.5 |
|  | 1625 | 27 | .0 | .0 | 95.5 |
|  | 1627 | 1 | .0 | .0 | 95.5 |
|  | 1629 | 2 | .0 | .0 | 95.5 |
|  | 1630 | 140 | .1 | .1 | 95.6 |
|  | 1631 | 4 | .0 | .0 | 95.6 |
|  | 1632 | 1 | .0 | .0 | 95.6 |
|  | 1633 | 3 | .0 | .0 | 95.6 |
|  | 1634 | 1 | .0 | .0 | 95.6 |
|  | 1635 | 21 | .0 | .0 | 95.6 |
|  | 1636 | 2 | .0 | .0 | 95.6 |
|  | 1638 | 3 | .0 | .0 | 95.6 |
|  | 1640 | 103 | .1 | .1 | 95.6 |
|  | 1641 | 1 | .0 | .0 | 95.6 |
|  | 1642 | 3 | .0 | .0 | 95.6 |
|  | 1644 | 2 | .0 | .0 | 95.6 |
|  | 1645 | 20 | .0 | .0 | 95.7 |
|  | 1646 | 1 | .0 | .0 | 95.7 |
|  | 1648 | 1 | .0 | .0 | 95.7 |
|  | 1650 | 63 | .0 | .0 | 95.7 |
|  | 1651 | 1 | .0 | .0 | 95.7 |
|  | 1652 | 2 | .0 | .0 | 95.7 |
|  | 1655 | 12 | .0 | .0 | 95.7 |
|  | 1656 | 2 | .0 | .0 | 95.7 |
|  | 1659 | 1 | .0 | .0 | 95.7 |
|  | 1660 | 13 | .0 | .0 | 95.7 |
|  | 17 | 1 | .0 | .0 | 95.7 |
|  | 17;10 | 1 | .0 | .0 | 95.7 |
|  | 17;15 | 2 | .0 | .0 | 95.7 |
|  | 17;20 | 7 | .0 | .0 | 95.7 |
|  | 17;30 | 2 | .0 | .0 | 95.7 |
|  | 17;35 | 2 | .0 | .0 | 95.7 |
|  | 17;40 | 1 | .0 | .0 | 95.7 |
|  | 17;42 | 1 | .0 | .0 | 95.7 |
|  | 17;45 | 1 | .0 | .0 | 95.7 |
|  | 17:00 | 898 | .5 | .5 | 96.2 |
|  | 17:02 | 14 | .0 | .0 | 96.2 |
|  | 17:03 | 12 | .0 | .0 | 96.2 |
|  | 17:04 | 12 | .0 | .0 | 96.2 |
|  | 17:05 | 117 | .1 | .1 | 96.3 |
|  | 17:06 | 8 | .0 | .0 | 96.3 |
|  | 17:07 | 5 | .0 | .0 | 96.3 |
|  | 17:08 | 10 | .0 | .0 | 96.3 |
|  | 17:09 | 6 | .0 | .0 | 96.3 |
|  | 17:10 | 953 | .5 | .5 | 96.8 |
|  | 17:11 | 8 | .0 | .0 | 96.8 |
|  | 17:12 | 22 | .0 | .0 | 96.8 |
|  | 17:13 | 22 | .0 | .0 | 96.8 |
|  | 17:14 | 5 | .0 | .0 | 96.8 |
|  | 17:15 | 135 | .1 | .1 | 96.9 |
|  | 17:16 | 5 | .0 | .0 | 96.9 |
|  | 17:17 | 13 | .0 | .0 | 96.9 |
|  | 17:18 | 12 | .0 | .0 | 96.9 |
|  | 17:19 | 4 | .0 | .0 | 96.9 |
|  | 17:20 | 624 | .3 | .3 | 97.2 |
|  | 17:21 | 7 | .0 | .0 | 97.2 |
|  | 17:22 | 11 | .0 | .0 | 97.2 |
|  | 17:23 | 9 | .0 | .0 | 97.2 |
|  | 17:24 | 7 | .0 | .0 | 97.2 |
|  | 17:25 | 112 | .1 | .1 | 97.3 |
|  | 17:26 | 5 | .0 | .0 | 97.3 |
|  | 17:27 | 5 | .0 | .0 | 97.3 |
|  | 17:28 | 18 | .0 | .0 | 97.3 |
|  | 17:29 | 4 | .0 | .0 | 97.3 |
|  | 17:3 | 1 | .0 | .0 | 97.3 |
|  | 17:30 | 496 | .3 | .3 | 97.6 |
|  | 17:31 | 5 | .0 | .0 | 97.6 |
|  | 17:32 | 8 | .0 | .0 | 97.6 |
|  | 17:33 | 7 | .0 | .0 | 97.6 |
|  | 17:34 | 6 | .0 | .0 | 97.6 |
|  | 17:35 | 64 | .0 | .0 | 97.6 |
|  | 17:36 | 9 | .0 | .0 | 97.6 |
|  | 17:37 | 3 | .0 | .0 | 97.6 |
|  | 17:38 | 7 | .0 | .0 | 97.6 |
|  | 17:39 | 5 | .0 | .0 | 97.6 |
|  | 17:40 | 413 | .2 | .2 | 97.9 |
|  | 17:41 | 6 | .0 | .0 | 97.9 |
|  | 17:42 | 16 | .0 | .0 | 97.9 |
|  | 17:43 | 5 | .0 | .0 | 97.9 |
|  | 17:44 | 6 | .0 | .0 | 97.9 |
|  | 17:45 | 94 | .0 | .0 | 97.9 |
|  | 17:46 | 3 | .0 | .0 | 97.9 |
|  | 17:47 | 4 | .0 | .0 | 97.9 |
|  | 17:48 | 4 | .0 | .0 | 97.9 |
|  | 17:49 | 5 | .0 | .0 | 97.9 |
|  | 17:50 | 181 | .1 | .1 | 98.0 |
|  | 17:51 | 2 | .0 | .0 | 98.0 |
|  | 17:52 | 2 | .0 | .0 | 98.0 |
|  | 17:53 | 3 | .0 | .0 | 98.0 |
|  | 17:54 | 10 | .0 | .0 | 98.0 |
|  | 17:55 | 48 | .0 | .0 | 98.1 |
|  | 17:56 | 8 | .0 | .0 | 98.1 |
|  | 17:57 | 15 | .0 | .0 | 98.1 |
|  | 17:58 | 8 | .0 | .0 | 98.1 |
|  | 17:59 | 8 | .0 | .0 | 98.1 |
|  | 17:60 | 24 | .0 | .0 | 98.1 |
|  | 17.0 | 1 | .0 | .0 | 98.1 |
|  | 17.00 | 3 | .0 | .0 | 98.1 |
|  | 17.05 | 1 | .0 | .0 | 98.1 |
|  | 17.07 | 1 | .0 | .0 | 98.1 |
|  | 17.10 | 2 | .0 | .0 | 98.1 |
|  | 17.11 | 1 | .0 | .0 | 98.1 |
|  | 17.19 | 1 | .0 | .0 | 98.1 |
|  | 17.20 | 6 | .0 | .0 | 98.1 |
|  | 17.25 | 2 | .0 | .0 | 98.1 |
|  | 17.28 | 1 | .0 | .0 | 98.1 |
|  | 17.30 | 3 | .0 | .0 | 98.1 |
|  | 17.40 | 4 | .0 | .0 | 98.1 |
|  | 17.41 | 1 | .0 | .0 | 98.1 |
|  | 17.45 | 1 | .0 | .0 | 98.1 |
|  | 17.50 | 2 | .0 | .0 | 98.1 |
|  | 1700 | 32 | .0 | .0 | 98.1 |
|  | 1702 | 3 | .0 | .0 | 98.1 |
|  | 1703 | 1 | .0 | .0 | 98.1 |
|  | 1705 | 5 | .0 | .0 | 98.1 |
|  | 1706 | 1 | .0 | .0 | 98.1 |
|  | 1707 | 2 | .0 | .0 | 98.1 |
|  | 1709 | 2 | .0 | .0 | 98.1 |
|  | 1710 | 24 | .0 | .0 | 98.2 |
|  | 1712 | 2 | .0 | .0 | 98.2 |
|  | 1713 | 3 | .0 | .0 | 98.2 |
|  | 1715 | 8 | .0 | .0 | 98.2 |
|  | 1720 | 22 | .0 | .0 | 98.2 |
|  | 1722 | 1 | .0 | .0 | 98.2 |
|  | 1725 | 3 | .0 | .0 | 98.2 |
|  | 1728 | 2 | .0 | .0 | 98.2 |
|  | 1729 | 1 | .0 | .0 | 98.2 |
|  | 1730 | 16 | .0 | .0 | 98.2 |
|  | 1732 | 1 | .0 | .0 | 98.2 |
|  | 1733 | 1 | .0 | .0 | 98.2 |
|  | 1735 | 7 | .0 | .0 | 98.2 |
|  | 1740 | 10 | .0 | .0 | 98.2 |
|  | 1741 | 2 | .0 | .0 | 98.2 |
|  | 1745 | 3 | .0 | .0 | 98.2 |
|  | 1748 | 2 | .0 | .0 | 98.2 |
|  | 1750 | 11 | .0 | .0 | 98.2 |
|  | 1755 | 1 | .0 | .0 | 98.2 |
|  | 1760 | 1 | .0 | .0 | 98.2 |
|  | 18;00 | 1 | .0 | .0 | 98.2 |
|  | 18;40 | 1 | .0 | .0 | 98.2 |
|  | 18:00 | 709 | .4 | .4 | 98.6 |
|  | 18:02 | 3 | .0 | .0 | 98.6 |
|  | 18:03 | 2 | .0 | .0 | 98.6 |
|  | 18:04 | 1 | .0 | .0 | 98.6 |
|  | 18:05 | 96 | .1 | .1 | 98.6 |
|  | 18:06 | 2 | .0 | .0 | 98.6 |
|  | 18:07 | 2 | .0 | .0 | 98.6 |
|  | 18:08 | 8 | .0 | .0 | 98.6 |
|  | 18:10 | 136 | .1 | .1 | 98.7 |
|  | 18:11 | 3 | .0 | .0 | 98.7 |
|  | 18:12 | 2 | .0 | .0 | 98.7 |
|  | 18:13 | 3 | .0 | .0 | 98.7 |
|  | 18:15 | 20 | .0 | .0 | 98.7 |
|  | 18:16 | 1 | .0 | .0 | 98.7 |
|  | 18:18 | 1 | .0 | .0 | 98.7 |
|  | 18:19 | 2 | .0 | .0 | 98.7 |
|  | 18:20 | 66 | .0 | .0 | 98.8 |
|  | 18:21 | 3 | .0 | .0 | 98.8 |
|  | 18:22 | 2 | .0 | .0 | 98.8 |
|  | 18:23 | 4 | .0 | .0 | 98.8 |
|  | 18:24 | 2 | .0 | .0 | 98.8 |
|  | 18:25 | 14 | .0 | .0 | 98.8 |
|  | 18:26 | 1 | .0 | .0 | 98.8 |
|  | 18:27 | 2 | .0 | .0 | 98.8 |
|  | 18:28 | 2 | .0 | .0 | 98.8 |
|  | 18:29 | 1 | .0 | .0 | 98.8 |
|  | 18:30 | 72 | .0 | .0 | 98.8 |
|  | 18:31 | 1 | .0 | .0 | 98.8 |
|  | 18:32 | 2 | .0 | .0 | 98.8 |
|  | 18:33 | 1 | .0 | .0 | 98.8 |
|  | 18:34 | 2 | .0 | .0 | 98.8 |
|  | 18:35 | 11 | .0 | .0 | 98.8 |
|  | 18:36 | 1 | .0 | .0 | 98.8 |
|  | 18:37 | 3 | .0 | .0 | 98.8 |
|  | 18:40 | 38 | .0 | .0 | 98.8 |
|  | 18:41 | 1 | .0 | .0 | 98.8 |
|  | 18:45 | 8 | .0 | .0 | 98.9 |
|  | 18:48 | 1 | .0 | .0 | 98.9 |
|  | 18:50 | 72 | .0 | .0 | 98.9 |
|  | 18:51 | 2 | .0 | .0 | 98.9 |
|  | 18:52 | 1 | .0 | .0 | 98.9 |
|  | 18:54 | 2 | .0 | .0 | 98.9 |
|  | 18:55 | 3 | .0 | .0 | 98.9 |
|  | 18:58 | 2 | .0 | .0 | 98.9 |
|  | 18:59 | 2 | .0 | .0 | 98.9 |
|  | 18:60 | 6 | .0 | .0 | 98.9 |
|  | 18:80 | 1 | .0 | .0 | 98.9 |
|  | 18:85 | 1 | .0 | .0 | 98.9 |
|  | 18.00 | 1 | .0 | .0 | 98.9 |
|  | 18.05 | 1 | .0 | .0 | 98.9 |
|  | 18.13 | 1 | .0 | .0 | 98.9 |
|  | 18.15 | 1 | .0 | .0 | 98.9 |
|  | 18.20 | 1 | .0 | .0 | 98.9 |
|  | 18.26 | 1 | .0 | .0 | 98.9 |
|  | 18.40 | 1 | .0 | .0 | 98.9 |
|  | 1800 | 4 | .0 | .0 | 98.9 |
|  | 1805 | 3 | .0 | .0 | 98.9 |
|  | 1810 | 5 | .0 | .0 | 98.9 |
|  | 1815 | 1 | .0 | .0 | 98.9 |
|  | 1820 | 3 | .0 | .0 | 98.9 |
|  | 1825 | 4 | .0 | .0 | 98.9 |
|  | 1830 | 2 | .0 | .0 | 98.9 |
|  | 1840 | 3 | .0 | .0 | 98.9 |
|  | 1850 | 3 | .0 | .0 | 98.9 |
|  | 1855 | 1 | .0 | .0 | 98.9 |
|  | 1860 | 1 | .0 | .0 | 98.9 |
|  | 19:00 | 280 | .1 | .1 | 99.1 |
|  | 19:02 | 1 | .0 | .0 | 99.1 |
|  | 19:03 | 1 | .0 | .0 | 99.1 |
|  | 19:04 | 2 | .0 | .0 | 99.1 |
|  | 19:05 | 60 | .0 | .0 | 99.1 |
|  | 19:06 | 1 | .0 | .0 | 99.1 |
|  | 19:07 | 2 | .0 | .0 | 99.1 |
|  | 19:08 | 1 | .0 | .0 | 99.1 |
|  | 19:10 | 225 | .1 | .1 | 99.2 |
|  | 19:11 | 1 | .0 | .0 | 99.2 |
|  | 19:12 | 2 | .0 | .0 | 99.2 |
|  | 19:13 | 2 | .0 | .0 | 99.2 |
|  | 19:15 | 71 | .0 | .0 | 99.3 |
|  | 19:20 | 300 | .2 | .2 | 99.4 |
|  | 19:21 | 1 | .0 | .0 | 99.4 |
|  | 19:22 | 1 | .0 | .0 | 99.4 |
|  | 19:25 | 88 | .0 | .0 | 99.5 |
|  | 19:26 | 4 | .0 | .0 | 99.5 |
|  | 19:30 | 330 | .2 | .2 | 99.6 |
|  | 19:34 | 1 | .0 | .0 | 99.6 |
|  | 19:35 | 81 | .0 | .0 | 99.7 |
|  | 19:40 | 231 | .1 | .1 | 99.8 |
|  | 19:45 | 106 | .1 | .1 | 99.9 |
|  | 19:50 | 196 | .1 | .1 | 100.0 |
|  | 19:55 | 70 | .0 | .0 | 100.0 |
|  | Total | 190290 | 100.0 | 100.0 |  |
|  |  |  |  |  |  |

Interview end timeInterview end time, table, 1 levels of column headers and 2 levels of row headers, table with 6 columns and 3 rows

|  |  |  |  |  |  |
| --- | --- | --- | --- | --- | --- |
|  | | Frequency | Percent | Valid Percent | Cumulative Percent |
| Valid |  | 190290 | 100.0 | 100.0 | 100.0 |
|  |  |  |  |  |  |

Sex of the individualSex of the individual, table, 1 levels of column headers and 2 levels of row headers, table with 6 columns and 5 rows

|  |  |  |  |  |  |
| --- | --- | --- | --- | --- | --- |
|  | | Frequency | Percent | Valid Percent | Cumulative Percent |
| Valid | Male | 95323 | 50.1 | 50.1 | 50.1 |
|  | Female | 94967 | 49.9 | 49.9 | 100.0 |
|  | Total | 190290 | 100.0 | 100.0 |  |
|  |  |  |  |  |  |

mother's serial numbermother's serial number, table, 1 levels of column headers and 2 levels of row headers, table with 6 columns and 13 rows

|  |  |  |  |  |  |
| --- | --- | --- | --- | --- | --- |
|  | | Frequency | Percent | Valid Percent | Cumulative Percent |
| Valid | 1 | 4948 | 2.6 | 2.6 | 2.6 |
|  | 2 | 131966 | 69.3 | 69.3 | 72.0 |
|  | 3 | 19900 | 10.5 | 10.5 | 82.4 |
|  | 4 | 22479 | 11.8 | 11.8 | 94.2 |
|  | 5 | 5500 | 2.9 | 2.9 | 97.1 |
|  | 6 | 3348 | 1.8 | 1.8 | 98.9 |
|  | 7 | 1247 | .7 | .7 | 99.5 |
|  | 8 | 576 | .3 | .3 | 99.8 |
|  | 9 | 225 | .1 | .1 | 99.9 |
|  | 10 | 101 | .1 | .1 | 100.0 |
|  | Total | 190290 | 100.0 | 100.0 |  |
|  |  |  |  |  |  |

relationship of primary caregiver with childrelationship of primary caregiver with child, table, 1 levels of column headers and 2 levels of row headers, table with 6 columns and 14 rows

|  |  |  |  |  |  |
| --- | --- | --- | --- | --- | --- |
|  | | Frequency | Percent | Valid Percent | Cumulative Percent |
| Valid | Paternal grandmother | 3308 | 1.7 | 72.1 | 72.1 |
|  | Paternal grandfather | 207 | .1 | 4.5 | 76.6 |
|  | Father | 266 | .1 | 5.8 | 82.4 |
|  | Maternal grandfather | 37 | .0 | .8 | 83.2 |
|  | Maternal grandmother | 369 | .2 | 8.0 | 91.3 |
|  | Sibling | 214 | .1 | 4.7 | 95.9 |
|  | Cousin | 43 | .0 | .9 | 96.9 |
|  | Aunt/uncle | 83 | .0 | 1.8 | 98.7 |
|  | Others(not coded) code start from 09 | 60 | .0 | 1.3 | 100.0 |
|  | Total | 4587 | 2.4 | 100.0 |  |
| Missing | System | 185703 | 97.6 |  |  |
| Total | | 190290 | 100.0 |  |  |
|  |  |  |  |  |  |

Age of the primary caregiverAge of the primary caregiver, table, 1 levels of column headers and 2 levels of row headers, table with 6 columns and 79 rows

|  |  |  |  |  |  |
| --- | --- | --- | --- | --- | --- |
|  | | Frequency | Percent | Valid Percent | Cumulative Percent |
| Valid | 7 | 2 | .0 | .0 | .0 |
|  | 8 | 19 | .0 | .4 | .5 |
|  | 9 | 6 | .0 | .1 | .6 |
|  | 10 | 82 | .0 | 1.8 | 2.4 |
|  | 11 | 41 | .0 | .9 | 3.3 |
|  | 12 | 48 | .0 | 1.0 | 4.3 |
|  | 13 | 33 | .0 | .7 | 5.0 |
|  | 14 | 2 | .0 | .0 | 5.1 |
|  | 15 | 31 | .0 | .7 | 5.8 |
|  | 16 | 12 | .0 | .3 | 6.0 |
|  | 17 | 1 | .0 | .0 | 6.0 |
|  | 18 | 10 | .0 | .2 | 6.3 |
|  | 19 | 9 | .0 | .2 | 6.5 |
|  | 20 | 20 | .0 | .4 | 6.9 |
|  | 21 | 6 | .0 | .1 | 7.0 |
|  | 22 | 7 | .0 | .2 | 7.2 |
|  | 23 | 9 | .0 | .2 | 7.4 |
|  | 24 | 6 | .0 | .1 | 7.5 |
|  | 25 | 63 | .0 | 1.4 | 8.9 |
|  | 26 | 3 | .0 | .1 | 8.9 |
|  | 27 | 1 | .0 | .0 | 9.0 |
|  | 28 | 6 | .0 | .1 | 9.1 |
|  | 29 | 2 | .0 | .0 | 9.1 |
|  | 30 | 96 | .1 | 2.1 | 11.2 |
|  | 32 | 10 | .0 | .2 | 11.4 |
|  | 33 | 4 | .0 | .1 | 11.5 |
|  | 34 | 1 | .0 | .0 | 11.6 |
|  | 35 | 134 | .1 | 2.9 | 14.5 |
|  | 36 | 76 | .0 | 1.7 | 16.1 |
|  | 37 | 5 | .0 | .1 | 16.2 |
|  | 38 | 50 | .0 | 1.1 | 17.3 |
|  | 39 | 75 | .0 | 1.6 | 19.0 |
|  | 40 | 451 | .2 | 9.8 | 28.8 |
|  | 41 | 43 | .0 | .9 | 29.7 |
|  | 42 | 89 | .0 | 1.9 | 31.7 |
|  | 43 | 184 | .1 | 4.0 | 35.7 |
|  | 44 | 60 | .0 | 1.3 | 37.0 |
|  | 45 | 289 | .2 | 6.3 | 43.3 |
|  | 46 | 64 | .0 | 1.4 | 44.7 |
|  | 47 | 87 | .0 | 1.9 | 46.6 |
|  | 48 | 113 | .1 | 2.5 | 49.1 |
|  | 49 | 19 | .0 | .4 | 49.5 |
|  | 50 | 471 | .2 | 10.3 | 59.7 |
|  | 51 | 27 | .0 | .6 | 60.3 |
|  | 52 | 302 | .2 | 6.6 | 66.9 |
|  | 53 | 15 | .0 | .3 | 67.2 |
|  | 54 | 12 | .0 | .3 | 67.5 |
|  | 55 | 105 | .1 | 2.3 | 69.8 |
|  | 56 | 85 | .0 | 1.9 | 71.6 |
|  | 57 | 106 | .1 | 2.3 | 73.9 |
|  | 58 | 18 | .0 | .4 | 74.3 |
|  | 59 | 67 | .0 | 1.5 | 75.8 |
|  | 60 | 213 | .1 | 4.6 | 80.4 |
|  | 61 | 12 | .0 | .3 | 80.7 |
|  | 62 | 100 | .1 | 2.2 | 82.9 |
|  | 63 | 118 | .1 | 2.6 | 85.5 |
|  | 64 | 11 | .0 | .2 | 85.7 |
|  | 65 | 39 | .0 | .9 | 86.5 |
|  | 66 | 35 | .0 | .8 | 87.3 |
|  | 67 | 47 | .0 | 1.0 | 88.3 |
|  | 68 | 1 | .0 | .0 | 88.4 |
|  | 69 | 6 | .0 | .1 | 88.5 |
|  | 70 | 159 | .1 | 3.5 | 92.0 |
|  | 71 | 3 | .0 | .1 | 92.0 |
|  | 72 | 1 | .0 | .0 | 92.0 |
|  | 75 | 39 | .0 | .9 | 92.9 |
|  | 77 | 125 | .1 | 2.7 | 95.6 |
|  | 78 | 48 | .0 | 1.0 | 96.7 |
|  | 80 | 84 | .0 | 1.8 | 98.5 |
|  | 82 | 29 | .0 | .6 | 99.1 |
|  | 83 | 28 | .0 | .6 | 99.7 |
|  | 85 | 3 | .0 | .1 | 99.8 |
|  | 90 | 8 | .0 | .2 | 100.0 |
|  | 94 | 1 | .0 | .0 | 100.0 |
|  | Total | 4587 | 2.4 | 100.0 |  |
| Missing | System | 185703 | 97.6 |  |  |
| Total | | 190290 | 100.0 |  |  |
|  |  |  |  |  |  |

Where the baby stay at the time of visitWhere the baby stay at the time of visit, table, 1 levels of column headers and 2 levels of row headers, table with 6 columns and 8 rows

|  |  |  |  |  |  |
| --- | --- | --- | --- | --- | --- |
|  | | Frequency | Percent | Valid Percent | Cumulative Percent |
| Valid | Inside the house | 80574 | 42.3 | 42.3 | 42.3 |
|  | Yard | 70625 | 37.1 | 37.1 | 79.5 |
|  | Outside the bari | 36180 | 19.0 | 19.0 | 98.5 |
|  | Anchal | 1640 | .9 | .9 | 99.3 |
|  | Others (not coded) code start from 04 | 1271 | .7 | .7 | 100.0 |
|  | Total | 190290 | 100.0 | 100.0 |  |
|  |  |  |  |  |  |

Whether the child was in the playpenWhether the child was in the playpen, table, 1 levels of column headers and 2 levels of row headers, table with 6 columns and 7 rows

|  |  |  |  |  |  |
| --- | --- | --- | --- | --- | --- |
|  | | Frequency | Percent | Valid Percent | Cumulative Percent |
| Valid | Yes | 91368 | 48.0 | 60.4 | 60.4 |
|  | No | 59831 | 31.4 | 39.6 | 100.0 |
|  | Total | 151199 | 79.5 | 100.0 |  |
| Missing | System | 39091 | 20.5 |  |  |
| Total | | 190290 | 100.0 |  |  |
|  |  |  |  |  |  |

What the child doing inside the playpenWhat the child doing inside the playpen, table, 1 levels of column headers and 2 levels of row headers, table with 6 columns and 9 rows

|  |  |  |  |  |  |
| --- | --- | --- | --- | --- | --- |
|  | | Frequency | Percent | Valid Percent | Cumulative Percent |
| Valid | Sleeping | 7600 | 4.0 | 8.3 | 8.3 |
|  | Playing | 78393 | 41.2 | 85.8 | 94.1 |
|  | Crying | 5054 | 2.7 | 5.5 | 99.6 |
|  | Others (not coded) Others code will be start from 04 | 321 | .2 | .4 | 100.0 |
|  | Total | 91368 | 48.0 | 100.0 |  |
| Missing | System | 98922 | 52.0 |  |  |
| Total | | 190290 | 100.0 |  |  |
|  |  |  |  |  |  |

Location of playpenLocation of playpen, table, 1 levels of column headers and 2 levels of row headers, table with 6 columns and 11 rows

|  |  |  |  |  |  |
| --- | --- | --- | --- | --- | --- |
|  | | Frequency | Percent | Valid Percent | Cumulative Percent |
| Valid | Inside the house | 84024 | 44.2 | 44.2 | 44.2 |
|  | In the cowshed | 3185 | 1.7 | 1.7 | 45.8 |
|  | Yard | 67086 | 35.3 | 35.3 | 81.1 |
|  | Kitchen | 5677 | 3.0 | 3.0 | 84.1 |
|  | Rooftop | 4119 | 2.2 | 2.2 | 86.2 |
|  | Not in the house | 499 | .3 | .3 | 86.5 |
|  | Porch | 25547 | 13.4 | 13.4 | 99.9 |
|  | Others (not coded) code start from 07 | 153 | .1 | .1 | 100.0 |
|  | Total | 190290 | 100.0 | 100.0 |  |
|  |  |  |  |  |  |

The physical condition of the playpenThe physical condition of the playpen, table, 1 levels of column headers and 2 levels of row headers, table with 6 columns and 9 rows

|  |  |  |  |  |  |
| --- | --- | --- | --- | --- | --- |
|  | | Frequency | Percent | Valid Percent | Cumulative Percent |
| Valid | Completely out of order/ Broken | 5418 | 2.8 | 2.8 | 2.8 |
|  | Cannot be assembeled | 607 | .3 | .3 | 3.2 |
|  | Some parts are broken | 4198 | 2.2 | 2.2 | 5.4 |
|  | In good / usable condition but not stable | 45411 | 23.9 | 23.9 | 29.2 |
|  | In good / usable condition and stable | 134421 | 70.6 | 70.6 | 99.9 |
|  | Others (not coded) code start from 06 | 235 | .1 | .1 | 100.0 |
|  | Total | 190290 | 100.0 | 100.0 |  |
|  |  |  |  |  |  |

What is the playpen being usedWhat is the playpen being used, table, 1 levels of column headers and 2 levels of row headers, table with 6 columns and 7 rows

|  |  |  |  |  |  |
| --- | --- | --- | --- | --- | --- |
|  | | Frequency | Percent | Valid Percent | Cumulative Percent |
| Valid | For safe keeping of the child | 182173 | 95.7 | 95.7 | 95.7 |
|  | For storing household goods | 4137 | 2.2 | 2.2 | 97.9 |
|  | not in use | 3831 | 2.0 | 2.0 | 99.9 |
|  | Others (not coded) code start from 03 | 149 | .1 | .1 | 100.0 |
|  | Total | 190290 | 100.0 | 100.0 |  |
|  |  |  |  |  |  |

No oneNo one, table, 1 levels of column headers and 2 levels of row headers, table with 6 columns and 5 rows

|  |  |  |  |  |  |
| --- | --- | --- | --- | --- | --- |
|  | | Frequency | Percent | Valid Percent | Cumulative Percent |
| Valid |  | 146186 | 76.8 | 76.8 | 76.8 |
|  | 1 | 44104 | 23.2 | 23.2 | 100.0 |
|  | Total | 190290 | 100.0 | 100.0 |  |
|  |  |  |  |  |  |

Mother/primary caregiverMother/primary caregiver, table, 1 levels of column headers and 2 levels of row headers, table with 6 columns and 5 rows

|  |  |  |  |  |  |
| --- | --- | --- | --- | --- | --- |
|  | | Frequency | Percent | Valid Percent | Cumulative Percent |
| Valid |  | 58738 | 30.9 | 30.9 | 30.9 |
|  | 1 | 131552 | 69.1 | 69.1 | 100.0 |
|  | Total | 190290 | 100.0 | 100.0 |  |
|  |  |  |  |  |  |

Paternal grandmotherPaternal grandmother, table, 1 levels of column headers and 2 levels of row headers, table with 6 columns and 5 rows

|  |  |  |  |  |  |
| --- | --- | --- | --- | --- | --- |
|  | | Frequency | Percent | Valid Percent | Cumulative Percent |
| Valid |  | 185650 | 97.6 | 97.6 | 97.6 |
|  | 1 | 4640 | 2.4 | 2.4 | 100.0 |
|  | Total | 190290 | 100.0 | 100.0 |  |
|  |  |  |  |  |  |

Age of grandmotherAge of grandmother, table, 1 levels of column headers and 2 levels of row headers, table with 6 columns and 49 rows

|  |  |  |  |  |  |
| --- | --- | --- | --- | --- | --- |
|  | | Frequency | Percent | Valid Percent | Cumulative Percent |
| Valid |  | 185650 | 97.6 | 97.6 | 97.6 |
|  | 34 | 2 | .0 | .0 | 97.6 |
|  | 35 | 42 | .0 | .0 | 97.6 |
|  | 36 | 4 | .0 | .0 | 97.6 |
|  | 37 | 2 | .0 | .0 | 97.6 |
|  | 38 | 66 | .0 | .0 | 97.6 |
|  | 39 | 8 | .0 | .0 | 97.6 |
|  | 40 | 591 | .3 | .3 | 97.9 |
|  | 41 | 14 | .0 | .0 | 97.9 |
|  | 42 | 85 | .0 | .0 | 98.0 |
|  | 43 | 25 | .0 | .0 | 98.0 |
|  | 44 | 9 | .0 | .0 | 98.0 |
|  | 45 | 667 | .4 | .4 | 98.4 |
|  | 46 | 39 | .0 | .0 | 98.4 |
|  | 47 | 22 | .0 | .0 | 98.4 |
|  | 48 | 96 | .1 | .1 | 98.4 |
|  | 49 | 34 | .0 | .0 | 98.5 |
|  | 50 | 1370 | .7 | .7 | 99.2 |
|  | 51 | 53 | .0 | .0 | 99.2 |
|  | 52 | 83 | .0 | .0 | 99.2 |
|  | 53 | 32 | .0 | .0 | 99.3 |
|  | 54 | 14 | .0 | .0 | 99.3 |
|  | 55 | 491 | .3 | .3 | 99.5 |
|  | 56 | 92 | .0 | .0 | 99.6 |
|  | 57 | 14 | .0 | .0 | 99.6 |
|  | 58 | 40 | .0 | .0 | 99.6 |
|  | 59 | 8 | .0 | .0 | 99.6 |
|  | 60 | 493 | .3 | .3 | 99.9 |
|  | 61 | 9 | .0 | .0 | 99.9 |
|  | 62 | 20 | .0 | .0 | 99.9 |
|  | 63 | 3 | .0 | .0 | 99.9 |
|  | 64 | 2 | .0 | .0 | 99.9 |
|  | 65 | 91 | .0 | .0 | 99.9 |
|  | 66 | 2 | .0 | .0 | 99.9 |
|  | 68 | 3 | .0 | .0 | 99.9 |
|  | 70 | 79 | .0 | .0 | 100.0 |
|  | 72 | 2 | .0 | .0 | 100.0 |
|  | 74 | 1 | .0 | .0 | 100.0 |
|  | 75 | 9 | .0 | .0 | 100.0 |
|  | 77 | 3 | .0 | .0 | 100.0 |
|  | 78 | 5 | .0 | .0 | 100.0 |
|  | 80 | 8 | .0 | .0 | 100.0 |
|  | 82 | 1 | .0 | .0 | 100.0 |
|  | 85 | 1 | .0 | .0 | 100.0 |
|  | 90 | 4 | .0 | .0 | 100.0 |
|  | 97 | 1 | .0 | .0 | 100.0 |
|  | Total | 190290 | 100.0 | 100.0 |  |
|  |  |  |  |  |  |

paternal grandfatherpaternal grandfather, table, 1 levels of column headers and 2 levels of row headers, table with 6 columns and 5 rows

|  |  |  |  |  |  |
| --- | --- | --- | --- | --- | --- |
|  | | Frequency | Percent | Valid Percent | Cumulative Percent |
| Valid |  | 189947 | 99.8 | 99.8 | 99.8 |
|  | 1 | 343 | .2 | .2 | 100.0 |
|  | Total | 190290 | 100.0 | 100.0 |  |
|  |  |  |  |  |  |

Age of paternal grandfatherAge of paternal grandfather, table, 1 levels of column headers and 2 levels of row headers, table with 6 columns and 32 rows

|  |  |  |  |  |  |
| --- | --- | --- | --- | --- | --- |
|  | | Frequency | Percent | Valid Percent | Cumulative Percent |
| Valid |  | 189947 | 99.8 | 99.8 | 99.8 |
|  | 40 | 21 | .0 | .0 | 99.8 |
|  | 45 | 36 | .0 | .0 | 99.8 |
|  | 48 | 11 | .0 | .0 | 99.9 |
|  | 49 | 1 | .0 | .0 | 99.9 |
|  | 50 | 68 | .0 | .0 | 99.9 |
|  | 51 | 1 | .0 | .0 | 99.9 |
|  | 52 | 4 | .0 | .0 | 99.9 |
|  | 53 | 1 | .0 | .0 | 99.9 |
|  | 54 | 6 | .0 | .0 | 99.9 |
|  | 55 | 29 | .0 | .0 | 99.9 |
|  | 56 | 7 | .0 | .0 | 99.9 |
|  | 57 | 5 | .0 | .0 | 99.9 |
|  | 58 | 1 | .0 | .0 | 99.9 |
|  | 59 | 3 | .0 | .0 | 99.9 |
|  | 60 | 79 | .0 | .0 | 100.0 |
|  | 61 | 2 | .0 | .0 | 100.0 |
|  | 62 | 8 | .0 | .0 | 100.0 |
|  | 63 | 1 | .0 | .0 | 100.0 |
|  | 65 | 14 | .0 | .0 | 100.0 |
|  | 66 | 1 | .0 | .0 | 100.0 |
|  | 67 | 1 | .0 | .0 | 100.0 |
|  | 69 | 1 | .0 | .0 | 100.0 |
|  | 70 | 32 | .0 | .0 | 100.0 |
|  | 73 | 1 | .0 | .0 | 100.0 |
|  | 76 | 1 | .0 | .0 | 100.0 |
|  | 80 | 6 | .0 | .0 | 100.0 |
|  | 85 | 1 | .0 | .0 | 100.0 |
|  | 90 | 1 | .0 | .0 | 100.0 |
|  | Total | 190290 | 100.0 | 100.0 |  |
|  |  |  |  |  |  |

FatherFather, table, 1 levels of column headers and 2 levels of row headers, table with 6 columns and 5 rows

|  |  |  |  |  |  |
| --- | --- | --- | --- | --- | --- |
|  | | Frequency | Percent | Valid Percent | Cumulative Percent |
| Valid |  | 189349 | 99.5 | 99.5 | 99.5 |
|  | 1 | 941 | .5 | .5 | 100.0 |
|  | Total | 190290 | 100.0 | 100.0 |  |
|  |  |  |  |  |  |

Age of fatherAge of father, table, 1 levels of column headers and 2 levels of row headers, table with 6 columns and 35 rows

|  |  |  |  |  |  |
| --- | --- | --- | --- | --- | --- |
|  | | Frequency | Percent | Valid Percent | Cumulative Percent |
| Valid |  | 189349 | 99.5 | 99.5 | 99.5 |
|  | 23 | 5 | .0 | .0 | 99.5 |
|  | 24 | 7 | .0 | .0 | 99.5 |
|  | 25 | 69 | .0 | .0 | 99.5 |
|  | 26 | 17 | .0 | .0 | 99.6 |
|  | 27 | 15 | .0 | .0 | 99.6 |
|  | 28 | 31 | .0 | .0 | 99.6 |
|  | 29 | 11 | .0 | .0 | 99.6 |
|  | 30 | 180 | .1 | .1 | 99.7 |
|  | 31 | 4 | .0 | .0 | 99.7 |
|  | 32 | 68 | .0 | .0 | 99.7 |
|  | 33 | 9 | .0 | .0 | 99.7 |
|  | 34 | 7 | .0 | .0 | 99.7 |
|  | 35 | 175 | .1 | .1 | 99.8 |
|  | 36 | 14 | .0 | .0 | 99.8 |
|  | 37 | 7 | .0 | .0 | 99.8 |
|  | 38 | 27 | .0 | .0 | 99.8 |
|  | 39 | 2 | .0 | .0 | 99.8 |
|  | 40 | 186 | .1 | .1 | 99.9 |
|  | 41 | 3 | .0 | .0 | 99.9 |
|  | 42 | 5 | .0 | .0 | 99.9 |
|  | 43 | 1 | .0 | .0 | 99.9 |
|  | 44 | 1 | .0 | .0 | 99.9 |
|  | 45 | 47 | .0 | .0 | 100.0 |
|  | 47 | 2 | .0 | .0 | 100.0 |
|  | 48 | 6 | .0 | .0 | 100.0 |
|  | 49 | 1 | .0 | .0 | 100.0 |
|  | 50 | 27 | .0 | .0 | 100.0 |
|  | 52 | 1 | .0 | .0 | 100.0 |
|  | 55 | 10 | .0 | .0 | 100.0 |
|  | 56 | 1 | .0 | .0 | 100.0 |
|  | 60 | 2 | .0 | .0 | 100.0 |
|  | Total | 190290 | 100.0 | 100.0 |  |
|  |  |  |  |  |  |

Maternal grandfatherMaternal grandfather, table, 1 levels of column headers and 2 levels of row headers, table with 6 columns and 5 rows

|  |  |  |  |  |  |
| --- | --- | --- | --- | --- | --- |
|  | | Frequency | Percent | Valid Percent | Cumulative Percent |
| Valid |  | 190226 | 100.0 | 100.0 | 100.0 |
|  | 1 | 64 | .0 | .0 | 100.0 |
|  | Total | 190290 | 100.0 | 100.0 |  |
|  |  |  |  |  |  |

Age of Maternal grandfatherAge of Maternal grandfather, table, 1 levels of column headers and 2 levels of row headers, table with 6 columns and 16 rows

|  |  |  |  |  |  |
| --- | --- | --- | --- | --- | --- |
|  | | Frequency | Percent | Valid Percent | Cumulative Percent |
| Valid |  | 190226 | 100.0 | 100.0 | 100.0 |
|  | 40 | 4 | .0 | .0 | 100.0 |
|  | 42 | 3 | .0 | .0 | 100.0 |
|  | 45 | 1 | .0 | .0 | 100.0 |
|  | 46 | 1 | .0 | .0 | 100.0 |
|  | 47 | 1 | .0 | .0 | 100.0 |
|  | 48 | 1 | .0 | .0 | 100.0 |
|  | 50 | 9 | .0 | .0 | 100.0 |
|  | 55 | 11 | .0 | .0 | 100.0 |
|  | 56 | 2 | .0 | .0 | 100.0 |
|  | 60 | 24 | .0 | .0 | 100.0 |
|  | 65 | 6 | .0 | .0 | 100.0 |
|  | 70 | 1 | .0 | .0 | 100.0 |
|  | Total | 190290 | 100.0 | 100.0 |  |
|  |  |  |  |  |  |

Maternal grandmotherMaternal grandmother, table, 1 levels of column headers and 2 levels of row headers, table with 6 columns and 5 rows

|  |  |  |  |  |  |
| --- | --- | --- | --- | --- | --- |
|  | | Frequency | Percent | Valid Percent | Cumulative Percent |
| Valid |  | 189569 | 99.6 | 99.6 | 99.6 |
|  | 1 | 721 | .4 | .4 | 100.0 |
|  | Total | 190290 | 100.0 | 100.0 |  |
|  |  |  |  |  |  |

Age of Maternal grandmotherAge of Maternal grandmother, table, 1 levels of column headers and 2 levels of row headers, table with 6 columns and 37 rows

|  |  |  |  |  |  |
| --- | --- | --- | --- | --- | --- |
|  | | Frequency | Percent | Valid Percent | Cumulative Percent |
| Valid |  | 189569 | 99.6 | 99.6 | 99.6 |
|  | 35 | 24 | .0 | .0 | 99.6 |
|  | 36 | 1 | .0 | .0 | 99.6 |
|  | 37 | 2 | .0 | .0 | 99.6 |
|  | 38 | 12 | .0 | .0 | 99.6 |
|  | 40 | 134 | .1 | .1 | 99.7 |
|  | 41 | 4 | .0 | .0 | 99.7 |
|  | 42 | 11 | .0 | .0 | 99.7 |
|  | 43 | 2 | .0 | .0 | 99.7 |
|  | 44 | 2 | .0 | .0 | 99.7 |
|  | 45 | 106 | .1 | .1 | 99.8 |
|  | 46 | 10 | .0 | .0 | 99.8 |
|  | 47 | 7 | .0 | .0 | 99.8 |
|  | 48 | 22 | .0 | .0 | 99.8 |
|  | 49 | 7 | .0 | .0 | 99.8 |
|  | 50 | 161 | .1 | .1 | 99.9 |
|  | 51 | 4 | .0 | .0 | 99.9 |
|  | 52 | 9 | .0 | .0 | 99.9 |
|  | 53 | 1 | .0 | .0 | 99.9 |
|  | 54 | 4 | .0 | .0 | 99.9 |
|  | 55 | 48 | .0 | .0 | 99.9 |
|  | 56 | 13 | .0 | .0 | 99.9 |
|  | 57 | 1 | .0 | .0 | 99.9 |
|  | 58 | 7 | .0 | .0 | 99.9 |
|  | 59 | 2 | .0 | .0 | 99.9 |
|  | 60 | 88 | .0 | .0 | 100.0 |
|  | 61 | 2 | .0 | .0 | 100.0 |
|  | 62 | 4 | .0 | .0 | 100.0 |
|  | 63 | 1 | .0 | .0 | 100.0 |
|  | 65 | 17 | .0 | .0 | 100.0 |
|  | 66 | 2 | .0 | .0 | 100.0 |
|  | 70 | 11 | .0 | .0 | 100.0 |
|  | 72 | 1 | .0 | .0 | 100.0 |
|  | 75 | 1 | .0 | .0 | 100.0 |
|  | Total | 190290 | 100.0 | 100.0 |  |
|  |  |  |  |  |  |

SiblingSibling, table, 1 levels of column headers and 2 levels of row headers, table with 6 columns and 5 rows

|  |  |  |  |  |  |
| --- | --- | --- | --- | --- | --- |
|  | | Frequency | Percent | Valid Percent | Cumulative Percent |
| Valid |  | 188747 | 99.2 | 99.2 | 99.2 |
|  | 1 | 1543 | .8 | .8 | 100.0 |
|  | Total | 190290 | 100.0 | 100.0 |  |
|  |  |  |  |  |  |

Age of siblingAge of sibling, table, 1 levels of column headers and 2 levels of row headers, table with 6 columns and 23 rows

|  |  |  |  |  |  |
| --- | --- | --- | --- | --- | --- |
|  | | Frequency | Percent | Valid Percent | Cumulative Percent |
| Valid | 3 | 5 | .0 | .3 | .3 |
|  | 4 | 9 | .0 | .6 | .9 |
|  | 5 | 115 | .1 | 7.5 | 8.4 |
|  | 6 | 116 | .1 | 7.5 | 15.9 |
|  | 7 | 134 | .1 | 8.7 | 24.6 |
|  | 8 | 194 | .1 | 12.6 | 37.1 |
|  | 9 | 114 | .1 | 7.4 | 44.5 |
|  | 10 | 245 | .1 | 15.9 | 60.4 |
|  | 11 | 78 | .0 | 5.1 | 65.5 |
|  | 12 | 203 | .1 | 13.2 | 78.6 |
|  | 13 | 55 | .0 | 3.6 | 82.2 |
|  | 14 | 66 | .0 | 4.3 | 86.5 |
|  | 15 | 101 | .1 | 6.5 | 93.0 |
|  | 16 | 49 | .0 | 3.2 | 96.2 |
|  | 17 | 8 | .0 | .5 | 96.7 |
|  | 18 | 33 | .0 | 2.1 | 98.8 |
|  | 19 | 3 | .0 | .2 | 99.0 |
|  | 20 | 15 | .0 | 1.0 | 100.0 |
|  | Total | 1543 | .8 | 100.0 |  |
| Missing | System | 188747 | 99.2 |  |  |
| Total | | 190290 | 100.0 |  |  |
|  |  |  |  |  |  |

CousinCousin, table, 1 levels of column headers and 2 levels of row headers, table with 6 columns and 5 rows

|  |  |  |  |  |  |
| --- | --- | --- | --- | --- | --- |
|  | | Frequency | Percent | Valid Percent | Cumulative Percent |
| Valid |  | 190118 | 99.9 | 99.9 | 99.9 |
|  | 1 | 172 | .1 | .1 | 100.0 |
|  | Total | 190290 | 100.0 | 100.0 |  |
|  |  |  |  |  |  |

Age of CousinAge of Cousin, table, 1 levels of column headers and 2 levels of row headers, table with 6 columns and 28 rows

|  |  |  |  |  |  |
| --- | --- | --- | --- | --- | --- |
|  | | Frequency | Percent | Valid Percent | Cumulative Percent |
| Valid | 4 | 2 | .0 | 1.2 | 1.2 |
|  | 5 | 9 | .0 | 5.2 | 6.4 |
|  | 6 | 2 | .0 | 1.2 | 7.6 |
|  | 7 | 4 | .0 | 2.3 | 9.9 |
|  | 8 | 11 | .0 | 6.4 | 16.3 |
|  | 9 | 7 | .0 | 4.1 | 20.3 |
|  | 10 | 23 | .0 | 13.4 | 33.7 |
|  | 11 | 2 | .0 | 1.2 | 34.9 |
|  | 12 | 37 | .0 | 21.5 | 56.4 |
|  | 13 | 6 | .0 | 3.5 | 59.9 |
|  | 14 | 8 | .0 | 4.7 | 64.5 |
|  | 15 | 27 | .0 | 15.7 | 80.2 |
|  | 16 | 5 | .0 | 2.9 | 83.1 |
|  | 17 | 1 | .0 | .6 | 83.7 |
|  | 18 | 9 | .0 | 5.2 | 89.0 |
|  | 19 | 3 | .0 | 1.7 | 90.7 |
|  | 20 | 7 | .0 | 4.1 | 94.8 |
|  | 21 | 2 | .0 | 1.2 | 95.9 |
|  | 22 | 1 | .0 | .6 | 96.5 |
|  | 24 | 1 | .0 | .6 | 97.1 |
|  | 25 | 3 | .0 | 1.7 | 98.8 |
|  | 27 | 1 | .0 | .6 | 99.4 |
|  | 30 | 1 | .0 | .6 | 100.0 |
|  | Total | 172 | .1 | 100.0 |  |
| Missing | System | 190118 | 99.9 |  |  |
| Total | | 190290 | 100.0 |  |  |
|  |  |  |  |  |  |

Aunt/uncleAunt/uncle, table, 1 levels of column headers and 2 levels of row headers, table with 6 columns and 5 rows

|  |  |  |  |  |  |
| --- | --- | --- | --- | --- | --- |
|  | | Frequency | Percent | Valid Percent | Cumulative Percent |
| Valid | 1 | 414 | .2 | 100.0 | 100.0 |
| Missing | System | 189876 | 99.8 |  |  |
| Total | | 190290 | 100.0 |  |  |
|  |  |  |  |  |  |

Age of Aunt/uncleAge of Aunt/uncle, table, 1 levels of column headers and 2 levels of row headers, table with 6 columns and 40 rows

|  |  |  |  |  |  |
| --- | --- | --- | --- | --- | --- |
|  | | Frequency | Percent | Valid Percent | Cumulative Percent |
| Valid | 7 | 1 | .0 | .2 | .2 |
|  | 8 | 2 | .0 | .5 | .7 |
|  | 9 | 4 | .0 | 1.0 | 1.7 |
|  | 10 | 6 | .0 | 1.4 | 3.1 |
|  | 11 | 5 | .0 | 1.2 | 4.3 |
|  | 12 | 15 | .0 | 3.6 | 8.0 |
|  | 13 | 14 | .0 | 3.4 | 11.4 |
|  | 14 | 11 | .0 | 2.7 | 14.0 |
|  | 15 | 21 | .0 | 5.1 | 19.1 |
|  | 16 | 15 | .0 | 3.6 | 22.7 |
|  | 17 | 8 | .0 | 1.9 | 24.6 |
|  | 18 | 32 | .0 | 7.7 | 32.4 |
|  | 19 | 2 | .0 | .5 | 32.9 |
|  | 20 | 57 | .0 | 13.8 | 46.6 |
|  | 21 | 3 | .0 | .7 | 47.3 |
|  | 22 | 12 | .0 | 2.9 | 50.2 |
|  | 23 | 2 | .0 | .5 | 50.7 |
|  | 24 | 8 | .0 | 1.9 | 52.7 |
|  | 25 | 45 | .0 | 10.9 | 63.5 |
|  | 26 | 6 | .0 | 1.4 | 65.0 |
|  | 27 | 1 | .0 | .2 | 65.2 |
|  | 28 | 10 | .0 | 2.4 | 67.6 |
|  | 29 | 7 | .0 | 1.7 | 69.3 |
|  | 30 | 45 | .0 | 10.9 | 80.2 |
|  | 31 | 1 | .0 | .2 | 80.4 |
|  | 32 | 3 | .0 | .7 | 81.2 |
|  | 34 | 2 | .0 | .5 | 81.6 |
|  | 35 | 31 | .0 | 7.5 | 89.1 |
|  | 36 | 1 | .0 | .2 | 89.4 |
|  | 38 | 2 | .0 | .5 | 89.9 |
|  | 40 | 22 | .0 | 5.3 | 95.2 |
|  | 45 | 11 | .0 | 2.7 | 97.8 |
|  | 50 | 7 | .0 | 1.7 | 99.5 |
|  | 55 | 1 | .0 | .2 | 99.8 |
|  | 56 | 1 | .0 | .2 | 100.0 |
|  | Total | 414 | .2 | 100.0 |  |
| Missing | System | 189876 | 99.8 |  |  |
| Total | | 190290 | 100.0 |  |  |
|  |  |  |  |  |  |

NeighbourNeighbour, table, 1 levels of column headers and 2 levels of row headers, table with 6 columns and 5 rows

|  |  |  |  |  |  |
| --- | --- | --- | --- | --- | --- |
|  | | Frequency | Percent | Valid Percent | Cumulative Percent |
| Valid | 1 | 1607 | .8 | 100.0 | 100.0 |
| Missing | System | 188683 | 99.2 |  |  |
| Total | | 190290 | 100.0 |  |  |
|  |  |  |  |  |  |

Age of NeighbourAge of Neighbour, table, 1 levels of column headers and 2 levels of row headers, table with 6 columns and 41 rows

|  |  |  |  |  |  |
| --- | --- | --- | --- | --- | --- |
|  | | Frequency | Percent | Valid Percent | Cumulative Percent |
| Valid | 8 | 18 | .0 | 1.1 | 1.1 |
|  | 10 | 96 | .1 | 6.0 | 7.1 |
|  | 12 | 20 | .0 | 1.2 | 8.3 |
|  | 13 | 9 | .0 | .6 | 8.9 |
|  | 14 | 2 | .0 | .1 | 9.0 |
|  | 15 | 86 | .0 | 5.4 | 14.4 |
|  | 16 | 3 | .0 | .2 | 14.6 |
|  | 17 | 34 | .0 | 2.1 | 16.7 |
|  | 18 | 108 | .1 | 6.7 | 23.4 |
|  | 19 | 14 | .0 | .9 | 24.3 |
|  | 20 | 91 | .0 | 5.7 | 29.9 |
|  | 21 | 7 | .0 | .4 | 30.4 |
|  | 22 | 102 | .1 | 6.3 | 36.7 |
|  | 23 | 25 | .0 | 1.6 | 38.3 |
|  | 24 | 17 | .0 | 1.1 | 39.3 |
|  | 25 | 214 | .1 | 13.3 | 52.6 |
|  | 26 | 46 | .0 | 2.9 | 55.5 |
|  | 27 | 16 | .0 | 1.0 | 56.5 |
|  | 28 | 9 | .0 | .6 | 57.1 |
|  | 29 | 23 | .0 | 1.4 | 58.5 |
|  | 30 | 408 | .2 | 25.4 | 83.9 |
|  | 31 | 5 | .0 | .3 | 84.2 |
|  | 32 | 64 | .0 | 4.0 | 88.2 |
|  | 34 | 34 | .0 | 2.1 | 90.3 |
|  | 35 | 54 | .0 | 3.4 | 93.7 |
|  | 36 | 15 | .0 | .9 | 94.6 |
|  | 39 | 3 | .0 | .2 | 94.8 |
|  | 40 | 51 | .0 | 3.2 | 97.9 |
|  | 45 | 3 | .0 | .2 | 98.1 |
|  | 48 | 1 | .0 | .1 | 98.2 |
|  | 50 | 3 | .0 | .2 | 98.4 |
|  | 51 | 1 | .0 | .1 | 98.4 |
|  | 55 | 22 | .0 | 1.4 | 99.8 |
|  | 60 | 1 | .0 | .1 | 99.9 |
|  | 80 | 1 | .0 | .1 | 99.9 |
|  | 84 | 1 | .0 | .1 | 100.0 |
|  | Total | 1607 | .8 | 100.0 |  |
| Missing | System | 188683 | 99.2 |  |  |
| Total | | 190290 | 100.0 |  |  |
|  |  |  |  |  |  |

OthersOthers, table, 1 levels of column headers and 2 levels of row headers, table with 6 columns and 5 rows

|  |  |  |  |  |  |
| --- | --- | --- | --- | --- | --- |
|  | | Frequency | Percent | Valid Percent | Cumulative Percent |
| Valid | 1 | 4190 | 2.2 | 100.0 | 100.0 |
| Missing | System | 186100 | 97.8 |  |  |
| Total | | 190290 | 100.0 |  |  |
|  |  |  |  |  |  |

Other specifyOther specify, table, 1 levels of column headers and 2 levels of row headers, table with 4 columns and 3 rows

|  |  |  |  |
| --- | --- | --- | --- |
|  | | Frequency | Percent |
| Missing | System | 190290 | 100.0 |
|  |  |  |  |

Age of other personAge of other person, table, 1 levels of column headers and 2 levels of row headers, table with 6 columns and 47 rows

|  |  |  |  |  |  |
| --- | --- | --- | --- | --- | --- |
|  | | Frequency | Percent | Valid Percent | Cumulative Percent |
| Valid | 10 | 16 | .0 | .4 | .4 |
|  | 12 | 1 | .0 | .0 | .4 |
|  | 13 | 5 | .0 | .1 | .5 |
|  | 15 | 29 | .0 | .7 | 1.2 |
|  | 16 | 37 | .0 | .9 | 2.1 |
|  | 17 | 86 | .0 | 2.1 | 4.2 |
|  | 18 | 109 | .1 | 2.6 | 6.8 |
|  | 19 | 174 | .1 | 4.2 | 10.9 |
|  | 20 | 448 | .2 | 10.7 | 21.6 |
|  | 21 | 31 | .0 | .7 | 22.3 |
|  | 22 | 230 | .1 | 5.5 | 27.8 |
|  | 23 | 180 | .1 | 4.3 | 32.1 |
|  | 24 | 78 | .0 | 1.9 | 34.0 |
|  | 25 | 458 | .2 | 10.9 | 44.9 |
|  | 26 | 107 | .1 | 2.6 | 47.5 |
|  | 27 | 246 | .1 | 5.9 | 53.3 |
|  | 28 | 94 | .0 | 2.2 | 55.6 |
|  | 29 | 22 | .0 | .5 | 56.1 |
|  | 30 | 507 | .3 | 12.1 | 68.2 |
|  | 31 | 1 | .0 | .0 | 68.2 |
|  | 32 | 143 | .1 | 3.4 | 71.6 |
|  | 33 | 52 | .0 | 1.2 | 72.9 |
|  | 34 | 76 | .0 | 1.8 | 74.7 |
|  | 35 | 245 | .1 | 5.8 | 80.5 |
|  | 36 | 73 | .0 | 1.7 | 82.3 |
|  | 37 | 16 | .0 | .4 | 82.7 |
|  | 38 | 18 | .0 | .4 | 83.1 |
|  | 39 | 1 | .0 | .0 | 83.1 |
|  | 40 | 169 | .1 | 4.0 | 87.2 |
|  | 42 | 8 | .0 | .2 | 87.4 |
|  | 43 | 15 | .0 | .4 | 87.7 |
|  | 45 | 182 | .1 | 4.3 | 92.1 |
|  | 47 | 3 | .0 | .1 | 92.1 |
|  | 49 | 1 | .0 | .0 | 92.1 |
|  | 50 | 256 | .1 | 6.1 | 98.3 |
|  | 54 | 1 | .0 | .0 | 98.3 |
|  | 55 | 34 | .0 | .8 | 99.1 |
|  | 57 | 15 | .0 | .4 | 99.5 |
|  | 60 | 12 | .0 | .3 | 99.7 |
|  | 80 | 4 | .0 | .1 | 99.8 |
|  | 90 | 6 | .0 | .1 | 100.0 |
|  | 97 | 1 | .0 | .0 | 100.0 |
|  | Total | 4190 | 2.2 | 100.0 |  |
| Missing | System | 186100 | 97.8 |  |  |
| Total | | 190290 | 100.0 |  |  |
|  |  |  |  |  |  |

Activites of mother/primary caregiver during that timeActivites of mother/primary caregiver during that time, table, 1 levels of column headers and 2 levels of row headers, table with 6 columns and 9 rows

|  |  |  |  |  |  |
| --- | --- | --- | --- | --- | --- |
|  | | Frequency | Percent | Valid Percent | Cumulative Percent |
| Valid | Not at home | 4527 | 2.4 | 7.4 | 7.4 |
|  | Doing household chores | 50246 | 26.4 | 81.7 | 89.1 |
|  | Taking rest | 5958 | 3.1 | 9.7 | 98.7 |
|  | others (not coded) code start from 04 | 771 | .4 | 1.3 | 100.0 |
|  | Total | 61502 | 32.3 | 100.0 |  |
| Missing | System | 128788 | 67.7 |  |  |
| Total | | 190290 | 100.0 |  |  |
|  |  |  |  |  |  |

Whether the mother used the playpen since the intervention worker's last visitWhether the mother used the playpen since the intervention worker's last visit, table, 1 levels of column headers and 2 levels of row headers, table with 6 columns and 5 rows

|  |  |  |  |  |  |
| --- | --- | --- | --- | --- | --- |
|  | | Frequency | Percent | Valid Percent | Cumulative Percent |
| Valid | Yes | 178868 | 94.0 | 94.0 | 94.0 |
|  | No | 11422 | 6.0 | 6.0 | 100.0 |
|  | Total | 190290 | 100.0 | 100.0 |  |
|  |  |  |  |  |  |

Number of days that the playpen used in last weekNumber of days that the playpen used in last week, table, 1 levels of column headers and 2 levels of row headers, table with 6 columns and 12 rows

|  |  |  |  |  |  |
| --- | --- | --- | --- | --- | --- |
|  | | Frequency | Percent | Valid Percent | Cumulative Percent |
| Valid | 1 | 11986 | 6.3 | 6.7 | 6.7 |
|  | 2 | 8881 | 4.7 | 5.0 | 11.7 |
|  | 3 | 15288 | 8.0 | 8.5 | 20.2 |
|  | 4 | 23618 | 12.4 | 13.2 | 33.4 |
|  | 5 | 31634 | 16.6 | 17.7 | 51.1 |
|  | 6 | 34751 | 18.3 | 19.4 | 70.5 |
|  | 7 | 52710 | 27.7 | 29.5 | 100.0 |
|  | Total | 178868 | 94.0 | 100.0 |  |
| Missing | System | 11422 | 6.0 |  |  |
| Total | | 190290 | 100.0 |  |  |
|  |  |  |  |  |  |

Average number of times the baby kept in the playpenAverage number of times the baby kept in the playpen, table, 1 levels of column headers and 2 levels of row headers, table with 6 columns and 16 rows

|  |  |  |  |  |  |
| --- | --- | --- | --- | --- | --- |
|  | | Frequency | Percent | Valid Percent | Cumulative Percent |
| Valid | 1 | 7011 | 3.7 | 3.9 | 3.9 |
|  | 2 | 35311 | 18.6 | 19.7 | 23.7 |
|  | 3 | 50473 | 26.5 | 28.2 | 51.9 |
|  | 4 | 36571 | 19.2 | 20.4 | 72.3 |
|  | 5 | 22677 | 11.9 | 12.7 | 85.0 |
|  | 6 | 11856 | 6.2 | 6.6 | 91.6 |
|  | 7 | 5283 | 2.8 | 3.0 | 94.6 |
|  | 8 | 5083 | 2.7 | 2.8 | 97.4 |
|  | 9 | 1757 | .9 | 1.0 | 98.4 |
|  | 10 | 2328 | 1.2 | 1.3 | 99.7 |
|  | 11 | 518 | .3 | .3 | 100.0 |
|  | Total | 178868 | 94.0 | 100.0 |  |
| Missing | System | 11422 | 6.0 |  |  |
| Total | | 190290 | 100.0 |  |  |
|  |  |  |  |  |  |

Wash dishes/clothWash dishes/cloth, table, 1 levels of column headers and 2 levels of row headers, table with 6 columns and 6 rows

|  |  |  |  |  |  |
| --- | --- | --- | --- | --- | --- |
|  | | Frequency | Percent | Valid Percent | Cumulative Percent |
| Valid |  | 11422 | 6.0 | 6.0 | 6.0 |
|  | Yes | 126371 | 66.4 | 66.4 | 72.4 |
|  | No | 52497 | 27.6 | 27.6 | 100.0 |
|  | Total | 190290 | 100.0 | 100.0 |  |
|  |  |  |  |  |  |

Taking care of the poultry/domestic animalsTaking care of the poultry/domestic animals, table, 1 levels of column headers and 2 levels of row headers, table with 6 columns and 6 rows

|  |  |  |  |  |  |
| --- | --- | --- | --- | --- | --- |
|  | | Frequency | Percent | Valid Percent | Cumulative Percent |
| Valid |  | 11422 | 6.0 | 6.0 | 6.0 |
|  | Yes | 81950 | 43.1 | 43.1 | 49.1 |
|  | No | 96918 | 50.9 | 50.9 | 100.0 |
|  | Total | 190290 | 100.0 | 100.0 |  |
|  |  |  |  |  |  |

Collect fuel/water/irrigation workCollect fuel/water/irrigation work, table, 1 levels of column headers and 2 levels of row headers, table with 6 columns and 6 rows

|  |  |  |  |  |  |
| --- | --- | --- | --- | --- | --- |
|  | | Frequency | Percent | Valid Percent | Cumulative Percent |
| Valid |  | 11422 | 6.0 | 6.0 | 6.0 |
|  | Yes | 39813 | 20.9 | 20.9 | 26.9 |
|  | No | 139055 | 73.1 | 73.1 | 100.0 |
|  | Total | 190290 | 100.0 | 100.0 |  |
|  |  |  |  |  |  |

Child Care-Bathing,teaching,feeding,taking to schoolChild Care-Bathing,teaching,feeding,taking to school, table, 1 levels of column headers and 2 levels of row headers, table with 6 columns and 6 rows

|  |  |  |  |  |  |
| --- | --- | --- | --- | --- | --- |
|  | | Frequency | Percent | Valid Percent | Cumulative Percent |
| Valid |  | 11422 | 6.0 | 6.0 | 6.0 |
|  | Yes | 31401 | 16.5 | 16.5 | 22.5 |
|  | No | 147467 | 77.5 | 77.5 | 100.0 |
|  | Total | 190290 | 100.0 | 100.0 |  |
|  |  |  |  |  |  |

Care of other HH membersCare of other HH members, table, 1 levels of column headers and 2 levels of row headers, table with 6 columns and 6 rows

|  |  |  |  |  |  |
| --- | --- | --- | --- | --- | --- |
|  | | Frequency | Percent | Valid Percent | Cumulative Percent |
| Valid |  | 11422 | 6.0 | 6.0 | 6.0 |
|  | Yes | 35930 | 18.9 | 18.9 | 24.9 |
|  | No | 142938 | 75.1 | 75.1 | 100.0 |
|  | Total | 190290 | 100.0 | 100.0 |  |
|  |  |  |  |  |  |

Other HH work e.g.Cleaning the HHOther HH work e.g.Cleaning the HH, table, 1 levels of column headers and 2 levels of row headers, table with 6 columns and 6 rows

|  |  |  |  |  |  |
| --- | --- | --- | --- | --- | --- |
|  | | Frequency | Percent | Valid Percent | Cumulative Percent |
| Valid |  | 11422 | 6.0 | 6.0 | 6.0 |
|  | Yes | 42930 | 22.6 | 22.6 | 28.6 |
|  | No | 135938 | 71.4 | 71.4 | 100.0 |
|  | Total | 190290 | 100.0 | 100.0 |  |
|  |  |  |  |  |  |

Working in others houseWorking in others house, table, 1 levels of column headers and 2 levels of row headers, table with 6 columns and 6 rows

|  |  |  |  |  |  |
| --- | --- | --- | --- | --- | --- |
|  | | Frequency | Percent | Valid Percent | Cumulative Percent |
| Valid |  | 11422 | 6.0 | 6.0 | 6.0 |
|  | Yes | 8607 | 4.5 | 4.5 | 10.5 |
|  | No | 170261 | 89.5 | 89.5 | 100.0 |
|  | Total | 190290 | 100.0 | 100.0 |  |
|  |  |  |  |  |  |

Leisure activities-chatting,sleeping,taking rest etc.Leisure activities-chatting,sleeping,taking rest etc., table, 1 levels of column headers and 2 levels of row headers, table with 6 columns and 6 rows

|  |  |  |  |  |  |
| --- | --- | --- | --- | --- | --- |
|  | | Frequency | Percent | Valid Percent | Cumulative Percent |
| Valid |  | 11422 | 6.0 | 6.0 | 6.0 |
|  | Yes | 16200 | 8.5 | 8.5 | 14.5 |
|  | No | 162668 | 85.5 | 85.5 | 100.0 |
|  | Total | 190290 | 100.0 | 100.0 |  |
|  |  |  |  |  |  |

CookingCooking, table, 1 levels of column headers and 2 levels of row headers, table with 6 columns and 6 rows

|  |  |  |  |  |  |
| --- | --- | --- | --- | --- | --- |
|  | | Frequency | Percent | Valid Percent | Cumulative Percent |
| Valid |  | 11422 | 6.0 | 6.0 | 6.0 |
|  | Yes | 115715 | 60.8 | 60.8 | 66.8 |
|  | No | 63153 | 33.2 | 33.2 | 100.0 |
|  | Total | 190290 | 100.0 | 100.0 |  |
|  |  |  |  |  |  |

OthersOthers, table, 1 levels of column headers and 2 levels of row headers, table with 6 columns and 6 rows

|  |  |  |  |  |  |
| --- | --- | --- | --- | --- | --- |
|  | | Frequency | Percent | Valid Percent | Cumulative Percent |
| Valid |  | 11422 | 6.0 | 6.0 | 6.0 |
|  | Yes | 3783 | 2.0 | 2.0 | 8.0 |
|  | No | 175085 | 92.0 | 92.0 | 100.0 |
|  | Total | 190290 | 100.0 | 100.0 |  |
|  |  |  |  |  |  |

Other(Specify)Other(Specify), table, 1 levels of column headers and 2 levels of row headers, table with 6 columns and 3 rows

|  |  |  |  |  |  |
| --- | --- | --- | --- | --- | --- |
|  | | Frequency | Percent | Valid Percent | Cumulative Percent |
| Valid |  | 190290 | 100.0 | 100.0 | 100.0 |
|  |  |  |  |  |  |

How long the child kept in the playpen during last use of the playpenHow long the child kept in the playpen during last use of the playpen, table, 1 levels of column headers and 2 levels of row headers, table with 6 columns and 154 rows

|  |  |  |  |  |  |
| --- | --- | --- | --- | --- | --- |
|  | | Frequency | Percent | Valid Percent | Cumulative Percent |
| Valid | 10 | 29629 | 15.6 | 16.6 | 16.6 |
|  | 11 | 1327 | .7 | .7 | 17.3 |
|  | 12 | 3864 | 2.0 | 2.2 | 19.5 |
|  | 13 | 984 | .5 | .6 | 20.0 |
|  | 14 | 793 | .4 | .4 | 20.5 |
|  | 15 | 19080 | 10.0 | 10.7 | 31.1 |
|  | 16 | 1051 | .6 | .6 | 31.7 |
|  | 17 | 549 | .3 | .3 | 32.0 |
|  | 18 | 850 | .4 | .5 | 32.5 |
|  | 19 | 449 | .2 | .3 | 32.7 |
|  | 20 | 39830 | 20.9 | 22.3 | 55.0 |
|  | 21 | 392 | .2 | .2 | 55.2 |
|  | 22 | 717 | .4 | .4 | 55.6 |
|  | 23 | 279 | .1 | .2 | 55.8 |
|  | 24 | 322 | .2 | .2 | 56.0 |
|  | 25 | 8485 | 4.5 | 4.7 | 60.7 |
|  | 26 | 370 | .2 | .2 | 60.9 |
|  | 27 | 181 | .1 | .1 | 61.0 |
|  | 28 | 266 | .1 | .1 | 61.2 |
|  | 29 | 80 | .0 | .0 | 61.2 |
|  | 30 | 28095 | 14.8 | 15.7 | 76.9 |
|  | 31 | 98 | .1 | .1 | 77.0 |
|  | 32 | 308 | .2 | .2 | 77.2 |
|  | 33 | 114 | .1 | .1 | 77.2 |
|  | 34 | 86 | .0 | .0 | 77.3 |
|  | 35 | 2645 | 1.4 | 1.5 | 78.7 |
|  | 36 | 147 | .1 | .1 | 78.8 |
|  | 37 | 42 | .0 | .0 | 78.8 |
|  | 38 | 57 | .0 | .0 | 78.9 |
|  | 39 | 30 | .0 | .0 | 78.9 |
|  | 40 | 9508 | 5.0 | 5.3 | 84.2 |
|  | 41 | 62 | .0 | .0 | 84.2 |
|  | 42 | 137 | .1 | .1 | 84.3 |
|  | 43 | 14 | .0 | .0 | 84.3 |
|  | 44 | 41 | .0 | .0 | 84.4 |
|  | 45 | 8272 | 4.3 | 4.6 | 89.0 |
|  | 46 | 27 | .0 | .0 | 89.0 |
|  | 47 | 16 | .0 | .0 | 89.0 |
|  | 48 | 21 | .0 | .0 | 89.0 |
|  | 49 | 12 | .0 | .0 | 89.0 |
|  | 50 | 3630 | 1.9 | 2.0 | 91.1 |
|  | 51 | 15 | .0 | .0 | 91.1 |
|  | 52 | 46 | .0 | .0 | 91.1 |
|  | 53 | 5 | .0 | .0 | 91.1 |
|  | 54 | 10 | .0 | .0 | 91.1 |
|  | 55 | 1200 | .6 | .7 | 91.8 |
|  | 56 | 36 | .0 | .0 | 91.8 |
|  | 57 | 6 | .0 | .0 | 91.8 |
|  | 58 | 48 | .0 | .0 | 91.8 |
|  | 59 | 19 | .0 | .0 | 91.8 |
|  | 60 | 5599 | 2.9 | 3.1 | 95.0 |
|  | 61 | 2 | .0 | .0 | 95.0 |
|  | 62 | 62 | .0 | .0 | 95.0 |
|  | 63 | 4 | .0 | .0 | 95.0 |
|  | 64 | 7 | .0 | .0 | 95.0 |
|  | 65 | 45 | .0 | .0 | 95.0 |
|  | 66 | 7 | .0 | .0 | 95.0 |
|  | 67 | 2 | .0 | .0 | 95.0 |
|  | 68 | 3 | .0 | .0 | 95.0 |
|  | 69 | 4 | .0 | .0 | 95.0 |
|  | 70 | 1086 | .6 | .6 | 95.6 |
|  | 71 | 1 | .0 | .0 | 95.6 |
|  | 72 | 12 | .0 | .0 | 95.6 |
|  | 73 | 2 | .0 | .0 | 95.6 |
|  | 74 | 1 | .0 | .0 | 95.6 |
|  | 75 | 141 | .1 | .1 | 95.7 |
|  | 76 | 6 | .0 | .0 | 95.7 |
|  | 77 | 3 | .0 | .0 | 95.7 |
|  | 78 | 3 | .0 | .0 | 95.7 |
|  | 80 | 1103 | .6 | .6 | 96.3 |
|  | 82 | 7 | .0 | .0 | 96.4 |
|  | 85 | 11 | .0 | .0 | 96.4 |
|  | 86 | 2 | .0 | .0 | 96.4 |
|  | 89 | 2 | .0 | .0 | 96.4 |
|  | 90 | 3008 | 1.6 | 1.7 | 98.0 |
|  | 92 | 6 | .0 | .0 | 98.0 |
|  | 93 | 1 | .0 | .0 | 98.0 |
|  | 95 | 19 | .0 | .0 | 98.1 |
|  | 96 | 1 | .0 | .0 | 98.1 |
|  | 97 | 2 | .0 | .0 | 98.1 |
|  | 100 | 205 | .1 | .1 | 98.2 |
|  | 101 | 11 | .0 | .0 | 98.2 |
|  | 102 | 49 | .0 | .0 | 98.2 |
|  | 103 | 9 | .0 | .0 | 98.2 |
|  | 104 | 1 | .0 | .0 | 98.2 |
|  | 105 | 55 | .0 | .0 | 98.2 |
|  | 106 | 6 | .0 | .0 | 98.2 |
|  | 107 | 3 | .0 | .0 | 98.2 |
|  | 108 | 3 | .0 | .0 | 98.3 |
|  | 109 | 2 | .0 | .0 | 98.3 |
|  | 110 | 260 | .1 | .1 | 98.4 |
|  | 111 | 4 | .0 | .0 | 98.4 |
|  | 112 | 18 | .0 | .0 | 98.4 |
|  | 113 | 8 | .0 | .0 | 98.4 |
|  | 114 | 6 | .0 | .0 | 98.4 |
|  | 115 | 43 | .0 | .0 | 98.4 |
|  | 116 | 4 | .0 | .0 | 98.4 |
|  | 117 | 1 | .0 | .0 | 98.4 |
|  | 118 | 1 | .0 | .0 | 98.4 |
|  | 119 | 3 | .0 | .0 | 98.4 |
|  | 120 | 1331 | .7 | .7 | 99.2 |
|  | 121 | 4 | .0 | .0 | 99.2 |
|  | 122 | 8 | .0 | .0 | 99.2 |
|  | 124 | 1 | .0 | .0 | 99.2 |
|  | 125 | 20 | .0 | .0 | 99.2 |
|  | 127 | 1 | .0 | .0 | 99.2 |
|  | 128 | 2 | .0 | .0 | 99.2 |
|  | 130 | 357 | .2 | .2 | 99.4 |
|  | 131 | 3 | .0 | .0 | 99.4 |
|  | 132 | 2 | .0 | .0 | 99.4 |
|  | 135 | 9 | .0 | .0 | 99.4 |
|  | 140 | 113 | .1 | .1 | 99.5 |
|  | 141 | 1 | .0 | .0 | 99.5 |
|  | 142 | 4 | .0 | .0 | 99.5 |
|  | 145 | 26 | .0 | .0 | 99.5 |
|  | 150 | 115 | .1 | .1 | 99.6 |
|  | 151 | 1 | .0 | .0 | 99.6 |
|  | 152 | 17 | .0 | .0 | 99.6 |
|  | 155 | 4 | .0 | .0 | 99.6 |
|  | 160 | 55 | .0 | .0 | 99.6 |
|  | 162 | 3 | .0 | .0 | 99.6 |
|  | 170 | 14 | .0 | .0 | 99.6 |
|  | 175 | 2 | .0 | .0 | 99.6 |
|  | 180 | 275 | .1 | .2 | 99.8 |
|  | 182 | 1 | .0 | .0 | 99.8 |
|  | 190 | 14 | .0 | .0 | 99.8 |
|  | 195 | 2 | .0 | .0 | 99.8 |
|  | 200 | 112 | .1 | .1 | 99.8 |
|  | 201 | 5 | .0 | .0 | 99.8 |
|  | 202 | 32 | .0 | .0 | 99.9 |
|  | 203 | 6 | .0 | .0 | 99.9 |
|  | 204 | 2 | .0 | .0 | 99.9 |
|  | 205 | 3 | .0 | .0 | 99.9 |
|  | 206 | 1 | .0 | .0 | 99.9 |
|  | 210 | 81 | .0 | .0 | 99.9 |
|  | 211 | 8 | .0 | .0 | 99.9 |
|  | 215 | 9 | .0 | .0 | 99.9 |
|  | 218 | 1 | .0 | .0 | 99.9 |
|  | 220 | 30 | .0 | .0 | 99.9 |
|  | 222 | 3 | .0 | .0 | 99.9 |
|  | 230 | 27 | .0 | .0 | 100.0 |
|  | 235 | 1 | .0 | .0 | 100.0 |
|  | 240 | 34 | .0 | .0 | 100.0 |
|  | 250 | 10 | .0 | .0 | 100.0 |
|  | 252 | 5 | .0 | .0 | 100.0 |
|  | 260 | 5 | .0 | .0 | 100.0 |
|  | 270 | 1 | .0 | .0 | 100.0 |
|  | 275 | 1 | .0 | .0 | 100.0 |
|  | 280 | 17 | .0 | .0 | 100.0 |
|  | Total | 178868 | 94.0 | 100.0 |  |
| Missing | System | 11422 | 6.0 |  |  |
| Total | | 190290 | 100.0 |  |  |
|  |  |  |  |  |  |

Whether the child got any injuries(fell out.cut,bruise)while the playpen being uWhether the child got any injuries(fell out.cut,bruise)while the playpen being u, table, 1 levels of column headers and 2 levels of row headers, table with 6 columns and 7 rows

|  |  |  |  |  |  |
| --- | --- | --- | --- | --- | --- |
|  | | Frequency | Percent | Valid Percent | Cumulative Percent |
| Valid | Yes | 1663 | .9 | .9 | .9 |
|  | No | 177207 | 93.1 | 99.1 | 100.0 |
|  | Total | 178870 | 94.0 | 100.0 |  |
| Missing | System | 11420 | 6.0 |  |  |
| Total | | 190290 | 100.0 |  |  |
|  |  |  |  |  |  |

Describe in detail how the child got injuredDescribe in detail how the child got injured, table, 1 levels of column headers and 2 levels of row headers, table with 6 columns and 6 rows

|  |  |  |  |  |  |
| --- | --- | --- | --- | --- | --- |
|  | | Frequency | Percent | Valid Percent | Cumulative Percent |
| Valid |  | 190288 | 100.0 | 100.0 | 100.0 |
|  | leg pain | 1 | .0 | .0 | 100.0 |
|  | pain | 1 | .0 | .0 | 100.0 |
|  | Total | 190290 | 100.0 | 100.0 |  |
|  |  |  |  |  |  |

Any difficulties faces during using the playpenAny difficulties faces during using the playpen, table, 1 levels of column headers and 2 levels of row headers, table with 6 columns and 8 rows

|  |  |  |  |  |  |
| --- | --- | --- | --- | --- | --- |
|  | | Frequency | Percent | Valid Percent | Cumulative Percent |
| Valid |  | 12051 | 6.3 | 6.3 | 6.3 |
|  | None | 175633 | 92.3 | 92.3 | 98.6 |
|  | Difficult to set up | 921 | .5 | .5 | 99.1 |
|  | Don't have enough space | 1529 | .8 | .8 | 99.9 |
|  | Others (Specify) | 156 | .1 | .1 | 100.0 |
|  | Total | 190290 | 100.0 | 100.0 |  |
|  |  |  |  |  |  |

InconvenientInconvenient, table, 1 levels of column headers and 2 levels of row headers, table with 6 columns and 5 rows

|  |  |  |  |  |  |
| --- | --- | --- | --- | --- | --- |
|  | | Frequency | Percent | Valid Percent | Cumulative Percent |
| Valid | Yes | 399 | .2 | .2 | .2 |
|  | No | 189891 | 99.8 | 99.8 | 100.0 |
|  | Total | 190290 | 100.0 | 100.0 |  |
|  |  |  |  |  |  |

Child does not want to stay in the playpenChild does not want to stay in the playpen, table, 1 levels of column headers and 2 levels of row headers, table with 6 columns and 5 rows

|  |  |  |  |  |  |
| --- | --- | --- | --- | --- | --- |
|  | | Frequency | Percent | Valid Percent | Cumulative Percent |
| Valid | Yes | 3731 | 2.0 | 2.0 | 2.0 |
|  | No | 186559 | 98.0 | 98.0 | 100.0 |
|  | Total | 190290 | 100.0 | 100.0 |  |
|  |  |  |  |  |  |

Child goes to crecheChild goes to creche, table, 1 levels of column headers and 2 levels of row headers, table with 6 columns and 5 rows

|  |  |  |  |  |  |
| --- | --- | --- | --- | --- | --- |
|  | | Frequency | Percent | Valid Percent | Cumulative Percent |
| Valid | Yes | 7427 | 3.9 | 3.9 | 3.9 |
|  | No | 182863 | 96.1 | 96.1 | 100.0 |
|  | Total | 190290 | 100.0 | 100.0 |  |
|  |  |  |  |  |  |

Have a caregiver to look after the childHave a caregiver to look after the child, table, 1 levels of column headers and 2 levels of row headers, table with 6 columns and 5 rows

|  |  |  |  |  |  |
| --- | --- | --- | --- | --- | --- |
|  | | Frequency | Percent | Valid Percent | Cumulative Percent |
| Valid | Yes | 308 | .2 | .2 | .2 |
|  | No | 189982 | 99.8 | 99.8 | 100.0 |
|  | Total | 190290 | 100.0 | 100.0 |  |
|  |  |  |  |  |  |

Don't think it is necessaryDon't think it is necessary, table, 1 levels of column headers and 2 levels of row headers, table with 6 columns and 5 rows

|  |  |  |  |  |  |
| --- | --- | --- | --- | --- | --- |
|  | | Frequency | Percent | Valid Percent | Cumulative Percent |
| Valid | Yes | 440 | .2 | .2 | .2 |
|  | No | 189850 | 99.8 | 99.8 | 100.0 |
|  | Total | 190290 | 100.0 | 100.0 |  |
|  |  |  |  |  |  |

Have other methods of supervision(specify)Have other methods of supervision(specify), table, 1 levels of column headers and 2 levels of row headers, table with 6 columns and 5 rows

|  |  |  |  |  |  |
| --- | --- | --- | --- | --- | --- |
|  | | Frequency | Percent | Valid Percent | Cumulative Percent |
| Valid | Yes | 54 | .0 | .0 | .0 |
|  | No | 190236 | 100.0 | 100.0 | 100.0 |
|  | Total | 190290 | 100.0 | 100.0 |  |
|  |  |  |  |  |  |

OthersOthers, table, 1 levels of column headers and 2 levels of row headers, table with 6 columns and 5 rows

|  |  |  |  |  |  |
| --- | --- | --- | --- | --- | --- |
|  | | Frequency | Percent | Valid Percent | Cumulative Percent |
| Valid | Yes | 725 | .4 | .4 | .4 |
|  | No | 189565 | 99.6 | 99.6 | 100.0 |
|  | Total | 190290 | 100.0 | 100.0 |  |
|  |  |  |  |  |  |

Other specifyOther specify, table, 1 levels of column headers and 2 levels of row headers, table with 6 columns and 3 rows

|  |  |  |  |  |  |
| --- | --- | --- | --- | --- | --- |
|  | | Frequency | Percent | Valid Percent | Cumulative Percent |
| Valid |  | 190290 | 100.0 | 100.0 | 100.0 |
|  |  |  |  |  |  |

Satisfaction level with the playpen interventionSatisfaction level with the playpen intervention, table, 1 levels of column headers and 2 levels of row headers, table with 6 columns and 8 rows

|  |  |  |  |  |  |
| --- | --- | --- | --- | --- | --- |
|  | | Frequency | Percent | Valid Percent | Cumulative Percent |
| Valid | Very dissatisfied | 4392 | 2.3 | 2.3 | 2.3 |
|  | Moderately dissatisfiwd | 1002 | .5 | .5 | 2.8 |
|  | Neigher satisfied or dissatisfied | 3271 | 1.7 | 1.7 | 4.6 |
|  | Moderately satisfied | 9328 | 4.9 | 4.9 | 9.5 |
|  | Very satisfied | 172297 | 90.5 | 90.5 | 100.0 |
|  | Total | 190290 | 100.0 | 100.0 |  |
|  |  |  |  |  |  |

Any suggestions to improve the playpen interventionAny suggestions to improve the playpen intervention, table, 1 levels of column headers and 2 levels of row headers, table with 6 columns and 6 rows

|  |  |  |  |  |  |
| --- | --- | --- | --- | --- | --- |
|  | | Frequency | Percent | Valid Percent | Cumulative Percent |
| Valid |  | 1745 | .9 | .9 | .9 |
|  | Yes (Specify) | 10816 | 5.7 | 5.7 | 6.6 |
|  | No | 177729 | 93.4 | 93.4 | 100.0 |
|  | Total | 190290 | 100.0 | 100.0 |  |
|  |  |  |  |  |  |

Code of suggestionCode of suggestion, table, 1 levels of column headers and 2 levels of row headers, table with 6 columns and 9 rows

|  |  |  |  |  |  |
| --- | --- | --- | --- | --- | --- |
|  | | Frequency | Percent | Valid Percent | Cumulative Percent |
| Valid |  | 190218 | 100.0 | 100.0 | 100.0 |
|  | 0 | 5 | .0 | .0 | 100.0 |
|  | 1 | 8 | .0 | .0 | 100.0 |
|  | 2 | 51 | .0 | .0 | 100.0 |
|  | 3 | 6 | .0 | .0 | 100.0 |
|  | 5 | 2 | .0 | .0 | 100.0 |
|  | Total | 190290 | 100.0 | 100.0 |  |
|  |  |  |  |  |  |

Currently participate in any income generating activitiesCurrently participate in any income generating activities, table, 1 levels of column headers and 2 levels of row headers, table with 6 columns and 5 rows

|  |  |  |  |  |  |
| --- | --- | --- | --- | --- | --- |
|  | | Frequency | Percent | Valid Percent | Cumulative Percent |
| Valid | Yes | 16256 | 8.5 | 8.5 | 8.5 |
|  | No | 174034 | 91.5 | 91.5 | 100.0 |
|  | Total | 190290 | 100.0 | 100.0 |  |
|  |  |  |  |  |  |

Number of hours per day devote to income generating activitiesNumber of hours per day devote to income generating activities, table, 1 levels of column headers and 2 levels of row headers, table with 6 columns and 12 rows

|  |  |  |  |  |  |
| --- | --- | --- | --- | --- | --- |
|  | | Frequency | Percent | Valid Percent | Cumulative Percent |
| Valid | None | 174034 | 91.5 | 91.5 | 91.5 |
|  | 1 | 404 | .2 | .2 | 91.7 |
|  | 2 | 1756 | .9 | .9 | 92.6 |
|  | 3 | 2316 | 1.2 | 1.2 | 93.8 |
|  | 4 | 5174 | 2.7 | 2.7 | 96.5 |
|  | 5 | 2356 | 1.2 | 1.2 | 97.8 |
|  | 6 | 2205 | 1.2 | 1.2 | 98.9 |
|  | 7 | 769 | .4 | .4 | 99.3 |
|  | 8 | 1276 | .7 | .7 | 100.0 |
|  | Total | 190290 | 100.0 | 100.0 |  |
|  |  |  |  |  |  |

Numder of hours per day devote to household choresNumder of hours per day devote to household chores, table, 1 levels of column headers and 2 levels of row headers, table with 6 columns and 14 rows

|  |  |  |  |  |  |
| --- | --- | --- | --- | --- | --- |
|  | | Frequency | Percent | Valid Percent | Cumulative Percent |
| Valid | None | 1665 | .9 | .9 | .9 |
|  | 1.00 | 625 | .3 | .3 | 1.2 |
|  | 2.00 | 5877 | 3.1 | 3.1 | 4.3 |
|  | 3.00 | 14178 | 7.5 | 7.5 | 11.8 |
|  | 4.00 | 31771 | 16.7 | 16.8 | 28.5 |
|  | 5.00 | 36022 | 18.9 | 19.0 | 47.5 |
|  | 6.00 | 54399 | 28.6 | 28.7 | 76.2 |
|  | 7.00 | 22526 | 11.8 | 11.9 | 88.1 |
|  | 8.00 | 22552 | 11.9 | 11.9 | 100.0 |
|  | Total | 189615 | 99.6 | 100.0 |  |
| Missing | System | 675 | .4 |  |  |
| Total | | 190290 | 100.0 |  |  |
|  |  |  |  |  |  |

Age category Age category, table, 1 levels of column headers and 2 levels of row headers, table with 6 columns and 6 rows

|  |  |  |  |  |  |
| --- | --- | --- | --- | --- | --- |
|  | | Frequency | Percent | Valid Percent | Cumulative Percent |
| Valid | 1 | 15253 | 8.0 | 8.0 | 8.0 |
|  | 2 | 129924 | 68.3 | 68.3 | 76.3 |
|  | 3 | 45113 | 23.7 | 23.7 | 100.0 |
|  | Total | 190290 | 100.0 | 100.0 |  |
|  |  |  |  |  |  |
