# Supplementary material for: Compliance to playpen usages to enhance parental supervision of under-five children in rural community of Bangladesh
Source: PLoS One. 2022 May 9;17(5):e0264902. doi: 10.1371/journal.pone.0264902 (PMC9084520; doi:10.1371/journal.pone.0264902)
Supplement: S3 File — (PDF) [file pone.0264902.s003.pdf]

Annexure

Annex 1: Specifications of a Wooden Playpen:

The following specifications have been maintained during production of Playpen as agreed with Johns Hopkins Bloomberg School of Public Health and CIPRB

I. It would be made of eucalyptus wood
II. Length of 30 inches; Breadth of 30 inches and Height of 30 inches
III. There were no sharp edges, corners or points and any cut end of metal tubing that could cause injury risk
IV. Local varnish was used
V. Each Playpen should last at least two years i.e. for the project period

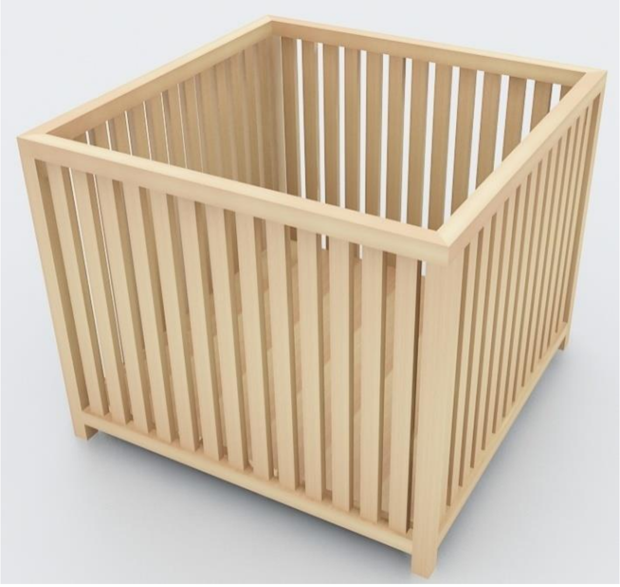

Figure 1: Prototype design of Playpen

Annex 2: Messages to be delivered during each household visit

**Household visit 1: Enrolment**<br>• **Introduce themselves and build rapport**<br>• **Discuss the importance of drowning, drowning prevention and the Playpen intervention.**<br>• **Read out the consent form and enroll child**	
**Discussion points**	Key messages for visit 1
**Drowning: When and at what age drowning deaths generally occur**	• Drowning is a significant cause of childhood death between the ages 1 to 4 years.<br>• All children age <5 years are at risk for drowning though the risks are highest when children first learn to walk and crawl and when children do not understand the danger of water.<br>• Children die throughout the year due to drowning.<br>• It most often occurs between late morning (around 9am) and early afternoon (1 pm) when mothers are busy with household activities.
**Places where small children are likely to drown**	• Most childhood drowning deaths occur in ponds and ditches.<br>• Surprisingly, many drowning deaths occur in low level water bodies, even on roads or paddy fields<br>• Some drowning deaths have occurred in a bucket full of water or in the container used to feed the cattle
**Importance of proper supervision**	• Drowning deaths can occur within a matter of a few minutes. Therefore, it is crucial to supervise the young child's movements at all times.

	• Appropriate supervision entails watching the child at all times. This means that young children should always be within eyesight of the supervising adult<br>• Never assume that another family member is taking charge of childcare or supervision
**What is a Playpen and how it can help?**	• A Playpen is a small, portable enclosure in which a young child can play alone safely<br>• If no adult is available to supervise the child's movements, use a protective measure such as the Playpen to ensure that the child's movement is restricted and he or she is thus safe from the danger of water.<br>• The Playpen should be used when the mother is busy in household activities or is unable to supervise the young child and an adult is not available to take care the Playpen can be used<br>• If all the family members are busy and the child is sleeping at that time, he or she needs to be in the Playpen during that time.<br>• The Playpen is to be used when the caregiver is busy and not at all times
**Discuss the visit schedule**	• We will come next day to distribute the Playpen.<br>• This was followed with visits on day 3 and day 7.<br>• Each visit was of 30-40 minutes where we will discuss with you whether you are facing in problems with the Playpen or not; and whether there has been any injury or not.<br>• We will also collect data on Playpen usage by the family using a short questionnaire

**Household visit 2: Playpen distribution**<br>• Distribute the Playpen to the enrolled children<br>• Demonstrate to the parent or caregiver how to set it up<br>• Deliver key messages to the parent or caregiver on how and when to use the Playpen<br>• Inform the parent or caregiver of the safety checklists that need to be followed.	
**Discussion points**	Key messages for visit 2
When to use the Playpen	• Use the Playpen, when there is no adult available to supervise the child's movements.<br>• That is, if the mother is busy in household activities or unable to supervise the young child and an adult is not available to take care of the child, use the Playpen.<br>• If all the family members are busy and the child is sleeping at that time, he or she needs to be in the Playpen during that time.<br>• The Playpen is to be used when the caregiver is busy and not at all times
Advantages of using the problem	• Keeping your child in the Playpen will stop him/her from moving around unsupervised while you focus on your own tasks,<br>• It will ensure that he or she is thus safe from the danger of water.<br>• You was able to easily see your child while they are playing and you are working
Challenges and how to solve it	• It may take a while for the child to adjust to the Playpen. However, if you make this a regular part of the daily routine, he or she will adapt quickly

	• Try to give some local toys in the Playpen for the child. This will help settle the child to adjust there. However, make sure there are no large toys that will enable the child to climb out.<br>• The Playpen may be new. It might take you some time to get used to it and most importantly the child may cry initially and take some time to get used to it. But it is important to use this tool for the sake of the child’s life
Others	• Keep the Playpen within eyesight, so that you can see him/her while you are working<br>• It is important that only one child is in the Playpen at any time; if you have twins, the child who needs the most supervision should be placed in the Playpen.

Follow-up visits

- Reinforce the importance of using the Playpen, for households who are using it.
- For mothers who are facing difficulties in using them, advise them on how to solve those. If you do not have any solutions inform your supervisor.
- For mothers who have stopped using the Playpen, ask them why and reinforce the importance of its use.
- Check the status of the Playpen (structural integrity and inform the supervisors if the Playpen is not in a good condition).
- Reiterate the messages in the safety checklist
- Use the compliance form to collect information about the Playpen usage.

Annex 3: Safety guidelines for the use of Playpens:

- *Place the Playpen in a safe location away from fire, heat sources or other hazardous environment*
- *Keep the Playpen away from cords, clothes line, and hanging ropes/drapes as these can pose strangulation hazard*
- *Place the Playpen at ground level and on an even surface at all times*
- *Do not use pillows, blankets, wraps, quilts or sheets when the child is placed in the Playpen as these can cause suffocation*
- *Do not place large toys, boxes or any large objects in the Playpen as these can be used by the child to climb out of the Playpen*
- *Toys and other items should not be tied at the corner or on the top of the Playpen railing as these can be a strangulation hazard*
- *Check the railing for holes and tears as children may bite the top railing during teething; discontinue the use of the Playpen if these holes cannot be repaired*
- *If a sleeping child is placed in the Playpen, put the child on its back and do not use bedding, cushion, pillow, blankets, wraps, sheets or soft toys as these may cause suffocation*
- *Do not give children plastic wrappings or plastic bags to play with when in Playpen or otherwise*
- *Do not tie anything around child's neck (e.g. threads, necklace, bibs, pacifiers) because if such an item catches somewhere in the Playpen it can lead to strangulation*
- *Do not cover the Playpen when it is in use, i.e. when a child is kept in it*
- *Do not use the Playpen if it is unstable*
- *Do not use the Playpen if there are problems with its structural integrity*
- *If the child is able to climb out of the Playpen, do not use it as it can cause fall injuries to the child*
- *Do not use the Playpen if you notice any splits, cracks or other defects*
- *Do not use the Playpen if its surface is rough; has sharp edges, corners or uneven planes, as these can cause injuries to the child*
- *Do not use the Playpen if it has a missing slats to avoid head entrapment and/or strangulation*
- *Place only one child in the Playpen at a time*
- *Keep the child in the Playpen for short periods of time*
- *Playpens assist in parental supervision efforts and are not meant to replace it. Hence, check the child frequently whenever s/he is placed in the Playpen*

Annex 4: Interviewer Manual – Playpen Compliance form:

Introduction

The primary form that was completed during household (HH) visits by the Anchal Maas (IW) is the *Playpen Compliance Form*. This requires the IWs to physically inspect the Playpen, its condition and whether/how it is being utilized. The mother/primary caregiver was questioned and will provide information on the Playpen's use.

If the interviewer discovers that the child has suffered from an injury since the previous visit an Injury Notification Form also be filled out and returned to the Anchal Supervisor.

Guidelines on household visit

General guidelines

A best and eligible informant is required to complete the compliance form. You should interview the mother or the primary caregiver in the household. If an appropriate respondent is not found in a household, go to the next one but plan to come back to this household at a later time. You might obtain all the necessary information by interviewing an appropriate respondent of the household; but often you may also want to talk to other members of the household to know about specific issues.

Responsibilities of the interviewer

The interviewer should enter into the household with proper greetings and then introduce themselves. Example of an introduction, "Hello, my name is (your name) and I am coming from CIPRB to collect necessary information for a project on prevention of childhood drowning. The information you provide was helpful in preventing children from drowning and injury related mortality and morbidity". Please tell the respondent that data was collected from

this household to evaluate the Playpens and that information was collected every 8 weeks during household visits. The interviewer should keep in his/her mind the following:

- As apologies can sometimes trigger a refusal, don't say "I am sorry to disturb you" or "are you busy now?" Rather say to the respondent that, "I'm here for the home visit" or "I would like to talk to you for a few minutes."

- If the respondent queries about the duration of the interview, please tell him/her that it will take about 40-50 minutes to complete this interview only.

- The presence of a third person during an interview session can hinder good responses. If a private and quiet environment is not available, the interview will have to be conducted in the same room but some form of privacy should be created to avoid other members of the household being tempted to eavesdrop.

- Most of the people are polite and will tend to provide answers that they think you might want to hear so try to be neutral when asking a question.

- The respondent might give an answer that seems illogical or irrelevant to the question. In such a circumstance, if you suggest an answer and then ask the respondent, "Is this what you wanted to say?" the respondent may accept your answer. So, please do not suggest answers; rather try to get the appropriate answer from the respondent.

- All questions must be read exactly as they appear in the questionnaire. If the respondent did not understand the question, repeat the question slowly and clearly.

- You must read slowly enough to allow the respondent time to understand everything you are asking; to do otherwise may lead to a "don't know" or incorrect answer.

- Situations may arise where a respondent may directly say “I don’t know” or give an irrelevant answer or may look displeased with the interview. In any of these circumstances, efforts should be there to make him/her interested in the discussion.

- Never be preconceived of the respondents’ level of knowledge and perceptions. Never presume that village people are unacquainted or unintelligent.

Annex 5: Playpen compliance form (Both Bangla and English)

Saving of Lives from Drowning (SoLiD) in Bangladesh
Compliance Form

	নাম Name	কোড Code
জেলা **DISTRICT**		☐
উপজেলা **UPAZILA**		☐
ইউনিয়ন **UNION**		☐☐
গ্রাম **VILLAGE**		☐☐☐
বাড়ি**BARI**		☐☐☐
খানার নম্বর **HOUSEHOLD NO**		☐☐☐☐
ঠিকানা **ADDRESS (Location)**		
ইন্টেরভেনশন কর্মী **INTERVENTION WORKER**		☐☐☐
শিশু**CHILD**		☐☐
মা **MOTHER**		☐☐
সেবাদানকারী(মা না হলে) **CAREGIVER (if not mother)**		☐☐

এই অংশে শিশুর পেন্ড-পেন ব্যবহার সম্পর্কিত তথ্য রয়েছেThis section contains information on the use of Playpens for the child

NO.	QUESTIONS AND FILTERS	CODING CATEGORIES	SKIP
W01	সাক্ষাতের তারিখ (দিন/মাস/বছর)<br>Date of visit (dd/mm/yy)	|___|___|/|___|___|/|___|___|	
W02	সাক্ষাতের সময়<br>Time of visit	|___|___|:|___|___|	
W03	ফলো-আপ সাক্ষাতের সংখ্যা<br>Follow-up visit number	1☐ 2☐ 3☐ 4☐ 5☐ 6☐ 7☐ 8☐ 9☐ 10☐ 11☐ 12☐ 13☐14☐15☐	
W04	শিশুর বয়স (পূর্ণ মাসে) এবং লিঙ্গ<br>Age of child (in completed months) and sex	|___|___|মাস month<br>পুরুষ Male ....1<br>মহিলা Female 2	
W05	প্রধান সেবাদানকারী ও শিশুর সম্পর্ক (যদি প্রধান সেবাদানকারী মা না হন) এবং প্রধান সেবাদানকারীর বয়স?<br>Relationship of primary caregiver with child (if primary caregiver is not mother) and what is the age of the primary caregiver?	Age<br>দাদি Paternal grandmother....................1..|___|___| দাদা Paternal grandfather ....2..|___|___| বাবা Father....................3..|___|___| নানা Maternal grandfather ....4..|___|___| নানি Maternal grandmother....................5..|___|___| ভাই-বোন Sibling ....6..|___|___| তুতো ভাই-বোন Cousin ....7..|___|___| চাচা/মামা/খালু/ফুপা/ চাচি/মামি/খালা/ফুপু Aunt/uncle....................8..|___|___| অন্যান্য (নির্দিষ্ট করুন) Others (Specify)____________________97.|___|___|	
W06	সাক্ষাতের সময় শিশু কোথায় আছে?<br>Where is the child at the time of the visit?	বাড়ির ভেতরে Inside the house ....1<br>উঠানে Yard ....2<br>বাড়ির বাইরে Outside the bari ....3<br>অন্যান্য (নির্দিষ্ট করুন) Others (specify)____________________ .97	W09 →<br>W09 →
W07	শিশুটি কি প্লে-পেন এর মধ্যে আছে?<br>Is the child in the Playpen?	হ্যাঁ Yes ....1<br>না No 2	W09 →
W08	প্লে-পেনে শিশুটি কি করছে?<br>What is the child doing in the Playpen?	ঘুমিয়ে আছে Sleeping ....1<br>খেলা করছে Playing....................2<br>কান্না করছে Crying....................3<br>অন্যান্য (নির্দিষ্ট করুন) Others (specify)_______________ 97	

NO.	QUESTIONS AND FILTERS	CODING CATEGORIES	SKIP
W09	প্লে-পেনটি কোথায় রাখা?<br>Where is the Playpen?	বাড়ির ভেতরে Inside the house .... 1<br>গোয়ালঘরে In the cowshed .... 2<br>উঠানে Yard.. 3<br>রান্নাঘরে Kitchen .... 4<br>ছাদে Rooftop .... 5<br>বাড়িতে নয় Not in the house.. 6<br>অন্যান্য (নির্দিষ্ট করুন) Others (specify)_______________ 97	
W10	প্লে-পেনটির বাহ্যিক অবস্থা কেমন?<br>What is the physical condition of the Playpen?	সম্পূর্ণ ভাঙ্গা Completely out of order/Broken .... 1<br>জোড়া লাগানো যাবে না এমন Cannot be assembeled .... 2<br>কিছু অংশ ভাঙ্গা Some parts are broken.. 3<br>ভাল/ব্যবহারযোগ্য অবস্থা কিন্তু মজবুত নয় In good/usable condition but not stable. 4<br>ভাল/ব্যবহারযোগ্য অবস্থা এবং মজবুত In good/usable condition and stable .... 5<br>অন্যান্য (নির্দিষ্ট করুন) Others (Specify) 97	
W11	প্লে-পেনটি কি কাজে ব্যবহৃত হচ্ছে?<br>What is the Playpen being used for?	শিশুকে নিরাপদ রাখার জন্য For safe keeping of the child................................. 1<br>খানার অন্যান্য জিনিস রাখার জন্য For storing household goods.......................... 2<br>অন্যান্য (নির্দিষ্ট করুন) Others (Specify) 97	
W12	এখন কে শিশুর দেখাশোনা করছে? (ভিসিটের সময়)<br>Who is supervising the child (at the time of visit)?	কেউ না No one .... A<br>মা/প্রধান সেবাদানকারী Mother/primary caregiver.. B<br>দাদি Paternal grandmother.. C..|___|___| দাদা Paternal grandfather .... D..|___|___| বাবা Father.. E..|___|___| নানা Maternal grandfather.. F..|___|___| নানি Maternal grandmother... G..|___|___| ভাই-বোন Sibling .... H..|___|___| তুতো ভাই-বোন Cousin .... I..|___|___| চাচা/মামা/খালু/ফুপা/ চাচি/মামি/খালা/ফুপু Aunt/uncle .... J..|___|___| প্রতিবেশী Neighbour .... K..|___|___| অন্যান্য (নির্দিষ্ট করুন) Others (Specify)____________________X..|___|___|	W14 →
W13	সেসময় মা/প্রধান সেবাদানকারী কি করছেন?<br>What is the mother/primary caregiver doing during that time?	বাড়িতে নয় Not at home .... 1<br>গৃহস্থালীর কাজ করছেন Doing household chores .... 2<br>বিশ্রাম নিচ্ছেন Taking rest .... 3<br>অন্যান্য (নির্দিষ্ট করুন) Others (specify)_______________ 97	
W14	আমার (কর্মীর) সর্বশেষ সাক্ষাতের পর আপনি কি প্লে-পেনটি ব্যবহার করেছেন?<br>Since my (intervention worker's) last visit, did you use the Playpen?	হ্যাঁ Yes .... 1<br>না No 2	W22 →
W15	গত সপ্তাহে (পূর্ণ সপ্তাহ) আপনি কতদিন প্লে-পেনটি ব্যবহার করেছেন?<br>In the last week, how many days did you use the Playpen?	|___||___| দিন days	
W16	প্লে-পেনটি ব্যবহার করলে একদিনে গড়ে কত ঘন্টা আপনি শিশুকে এতে রাখেন?<br>On average, how many times do you keep your child in the Playpen per day of use?	|___|___| দিনে এত বার (counts per day)	
W17	বাচ্চা যখন প্লে-পেনটি এ থাকে তখন আপনি কি কাজ করেন?<br>What activities do you do when the child is in the Playpen?	থালা-বাসন/কাপড় ধোয়া (Wash dishes/cloth)……………………………………………A<br>হাঁস-মুরগী/গবাদি পশুর যত্ন নেয়া (Taking care of the poultry/domestic animals)……………………………………………………………………………..B<br>জ্বালানী/পানি সংগ্রহ করা/সেচের কাজ (Collect fuel/water/ irrigation work)……………………C<br>শিশুর যত্ন নেয়া-গোসল করানো/পাঠদান/খাওয়ানো/স্কুলে নেয়া (Child Care-Bathing,teaching,feeding,taking to school)……………………………………………D<br>খানার অন্যান্য সদস্যদের যত্ন নেয়া (Care of other HH members)………………………………E<br>খানার অন্যান্য কাজ যেমন খানা পরিষ্কার পরিচ্ছন্ন করা (Other HH work e.g. Cleaning the HH)……F<br>অন্যের বাড়িতে কাজ করা(Working in others' house)……………………………………………………………………..…………G<br>অবসর যাপন-আড্ডা দেয়া, ঘুমানো, বিশ্রাম নেয়া ইত্যাদি (Leisure activities-chatting, sleeping, taking rest etc.)……………………………………………………………………………………H<br>অন্যান্য (নির্দিষ্ট করুন) Others (Specify) ___________________________________X	
W18	শেষবার ব্যবহারকালে আপনার শিশুকে কতক্ষন (মিনিট) প্লে-পেনে রেখেছিলেন?<br>During your last use, how long (minutes) did you keep your child in the Playpen?	|___|___|___| মিনিট Minutes	

NO.	QUESTIONS AND FILTERS	CODING CATEGORIES	SKIP
W19	আমার সর্বশেষ সাক্ষাতের পর কখনও কি আপনার শিশু প্লে-পেন ব্যবহারকালে কোন আঘাত পেয়েছিল?<br>Since my last visit, did your child fell out, cut, bruise or suffer from any injuries while the Playpen was being used?	হ্যাঁ Yes .... 1<br>না No 2	W21 →
W20	আপনার শিশু কিভাবে আঘাত পেয়েছিল তা বিশদভাবে বর্ণনা করুন?<br>Describe in detail how the child got injured:		Complete an injury notification form-1 and submit to supervisor
W21	প্লে-পেন ব্যবহারকালে আপনি কি ধরনের সমস্যার সম্মুখীন হয়েছিলেন?<br>What kind of difficulties did you encounter in using the Playpen?	একদমই না None........ 1<br>জোড়া লাগানো কঠিন Difficult to set up .... 2<br>যথেষ্ট জায়গার অভাব Don't have enough space .... 3<br>অন্যান্য (সুনির্দিষ্ট করুন) Others (specify)_______________ 97	
W22	আমার সর্বশেষ সাক্ষাতের পর আপনি যদি কখনও প্লে-পেন ব্যবহার না করে থাকেন বা কোন কারণে এটা ব্যবহার করা বন্ধ করে থাকেন- দয়া করে বলুন কি কি কারণে ।<br>If you have not used the Playpen since my last visit or stopped using it for any reason, please mention the reasons .	অসুবিধাজনক Inconvenient........ A<br>শিশু প্লে-পেনে থাকতে চায় না Child does not want to stay in the Playpen........ B<br>শিশু ক্রেশ এ যায় Child goes to crèche .... C<br>শিশুর দেখাশোনার জন্য সেবাদানকারী রয়েছে Have a caregiver to look after the child .... D<br>এটার প্রয়োজন আছে মনে করি না Don't think it is necessary ....E<br>শিশুর দেখাশোনার অন্য পদ্ধতি(নির্দিষ্ট করুন)<br>Have other methods of supervision (specify) ....E<br>অন্যান্য (নির্দিষ্ট করুন) Others (specify)_______________ X	
W23	প্লে-পেন পদ্ধতি ব্যবহার নিয়ে আপনি কতটুকু সন্তুষ্ট?<br>How satisfied are you with the Playpen intervention?	খুবই অসন্তুষ্ট Very dissatisfied........ 1<br>কিছুটাঅসন্তুষ্ট Moderately dissatisfied .... 2<br>সন্তুষ্ট নয় আবার অসন্তুষ্টও নয় Neither satisfied or dissatisfied .... 3<br>কিছুটা সন্তুষ্ট Moderately satisfied .... 4<br>খুবইসন্তুষ্ট Very satisfied 5	
W24	প্লে-পেন পদ্ধতি ব্যবহার উন্নত করার ব্যাপারে আমাদের প্রতি আপনার কোন পরামর্শ আছে কি?<br>Do you have any suggestions for us to improve the Playpen intervention?	হ্যাঁ (সুনির্দিষ্ট করুন) Yes (specify) .... 1<br>না No 2	
W26	আপনি কি বর্তমানে কোন আয়-উপার্জনের সাথে যুক্ত ?<br>Do you currently participate in any income generating activities?	হ্যাঁYes .... 1<br>নাNo 2	
W27	আপনি সারাদিনে কতক্ষন আয়-উপার্জনের সাথে যুক্ত থাকেন?<br>[ইন্টারভিউয়ারঃ আয়-উপার্জনের সাথে যুক্ত না থাকলে 00 লিখুন]প<br>How many hours per day do you devote to income generating activities? [INTERVIEWER: WRITE ZERO IF NONE.]	|_____|______|ঘন্টা(hours)	
W28	। আপনি সারাদিনে কতক্ষন গৃহস্থালির কাজের সাথে যুক্ত থাকেন?<br>[ইন্টারভিউয়ারঃ গৃহস্থালির কাজের সাথে যুক্ত না থাকলে 00 লিখুন]<br>How many hours per day do you devote to household chores? [INTERVIEWER: WRITE ZERO IF NONE.]	|_____|______|ঘন্টা (hours)	END
